# Pan-genome analysis of the genus *Finegoldia* identifies two distinct clades, strain-specific heterogeneity, and putative virulence factors

**DOI:** 10.1038/s41598-017-18661-8

**Published:** 2018-01-10

**Authors:** Holger Brüggemann, Anders Jensen, Seven Nazipi, Hüsnü Aslan, Rikke Louise Meyer, Anja Poehlein, Elzbieta Brzuszkiewicz, Munir A. Al-Zeer, Volker Brinkmann, Bo Söderquist

**Affiliations:** 1 Department of Biomedicine, Aarhus University, Aarhus, Denmark; 2 Department of Bioscience, Aarhus University, Aarhus, Denmark; 3 Department of Genomic and Applied Microbiology, Institute of Microbiology and Genetics, Georg-August University Göttingen, Göttingen, Germany; 4 Department of Applied Biochemistry, Institute of Biotechnology, TU Berlin, Germany; 5 Microscopy Core Facility, Max Planck Institute for Infection Biology, Berlin, Germany; 6 Department of Laboratory Medicine, Clinical Microbiology, Faculty of Medicine and Health, Örebro University, 70185 Örebro, Sweden

## Abstract

*Finegoldia magna*, a Gram-positive anaerobic coccus, is an opportunistic pathogen, associated with medical device-related infections. *F*. *magna* is the only described species of the genus *Finegoldia*. We report the analysis of 17 genomes of *Finegoldia* isolates. Phylogenomic analyses showed that the *Finegoldia* population can be divided into two distinct clades, with an average nucleotide identity of 90.7%. One clade contains strains of *F. magna*, whereas the other clade includes more heterogeneous strains, hereafter tentatively named “*Finegoldia nericia*”. The latter species appears to be more abundant in the human microbiome. Surface structure differences between strains of *F. magna* and *“F. nericia*” were detected by microscopy. Strain-specific heterogeneity is high and previously identified host-interacting factors are present only in subsets of *“F. nericia”* and *F*. *magna* strains. However, all genomes encode multiple host factor-binding proteins such as albumin-, collagen-, and immunoglobulin-binding proteins, and two to four copies of CAMP (Christie-Atkins-Munch-Petersen) factors; in accordance, most strains show a positive CAMP reaction for co-hemolysis. Our work sheds new light of the genus *Finegoldia* and its ability to bind host components. Future research should explore if the genomic differences identified here affect the potential of different *Finegoldia* species and strains to cause opportunistic infections.

## Introduction

*Finegoldia* is a genus of Gram-positive anaerobic cocci (GPAC) within the class Clostridia; the currently only described species of this genus is *Finegoldia magna*, formerly known as *Peptostreptococcus magnus* ^1^. *Finegoldia magna* is part of the human microbiota and present on the skin and mucosal surfaces of the oral cavity, the gastrointestinal and genitourinary tracts.

GPAC account for 25–30% of all isolated anaerobic bacteria from clinical specimens, and *F*. *magna* is commonly found in clinical materials and infection sites such as soft tissue and wound infections, including diabetic ulcers, bone and joint infections, prosthetic valve endocarditis, pneumonia, vaginosis, chronic balanitis, and others^2–5^. Thus, *F. magna* is regarded as an opportunistic pathogen with an elevated pathogenic potential^4,5^.

The identification of *F*. *magna* has been improved in recent years, mainly due to the implementation of MALDI-TOF mass spectrometry. However, the isolation and cultivation from blood or infection sites still remain challenging, due to elaborate growth requirements, including the sensitivity to oxygen, extended cultivation times, and growth media used^6^. In addition, in polymicrobial infections with fast-growing pathogens, the presence of *F*. *magna* may be neglected. Thus, it is assumed that the proportion of *F*. *magna* reported in clinical specimens is underestimated^4,5^.

Heterogeneity among *F*. *magna* isolates has been described, for example regarding growth behavior and biochemical reactions such as alkaline phosphatase and arginine dihydrolase^2,4,7^. Also, morphological heterogeneity has been reported, such as varied cell size (0.8 to 1.6 μm in diameter) and colony color (translucent, white, grey, yellow)^2,7^. Some virulence properties of *F*. *magna* have been identified, such as traits to allow efficient colonization and persistency in the host^4,5^. These traits are mediated by host-interacting factors, such as sortase-dependent pili, protein L (a surface protein with affinity for Ig L chains), PAB (peptostreptococcal albumin binding protein), SufA (a subtilisin-like proteinase) and FAF (*F. magna* adhesion factor)^8–13^. Interestingly, heterogeneity was observed regarding the presence of these factors in different *F. magna* strains; for example, protein L was only detected in about 10% of the *F. magna* isolates^4^. The first complete genome sequence of *F. magna* strain ATCC 29328, originally isolated from an abdominal wound, shed some light on genomic features, such as additional proteins with GA modules (protein G-related albumin-binding domain) and a plasmid that encoded several sortases^14^.

The aim of the present study was to investigate *Finegoldia* sp. isolates by comparing their genomes and performing additional investigations, including microscopy and co-hemolysis assays. Substantial heterogeneity among isolates was uncovered; a population distinct from *F*. *magna* was found that has features of a novel *Finegoldia* species, tentatively named “*Finegoldia nericia”*.

## Results

### Genomes of strains of *Finegoldia*

In total 17 genomes of *Finegoldia* sp. were analyzed and compared. Ten strains have recently been sequenced by our group^15^; they were isolated from patients with orthopedic joint implant-associated infections in Sweden. Seven genomes were previously sequenced by others, including the closed genome of the type strain ATCC 29328^14^. Genome features of all so far sequenced strains of *Finegoldia* are summarized in Table 1. The annotation using Prokka detected between 1570 and 1906 coding sequences (CDS) per genome, with an average of 1760 CDS per strain.

**Table 1:** Features of sequenced genomes of *Finegoldia* strains.

STRAIN NAME	SPECIES	GENOME SIZE (KB)	G+C (%)	CONTIGS (#)	CDS (PROKKA)
07T609	*F. magna*	1822	32.0	33	1698
08T492	*F. magna*	1904	31.9	34	1796
09T408	*F. magna*	1807	32.1	44	1677
09T494	*“F. nericia”*	1913	31.8	20	1827
12T272	*“F. nericia”*	1872	31.9	21	1773
12T273	*“F. nericia”*	1926	31.8	24	1840
12T306	*“F. nericia”*	1838	31.9	42	1712
CCUG54800	*“F. nericia”*	1990	31.8	67	1906
T151023	*“F. nericia”*	1679	32.1	40	1570
T160124	*“F. nericia”*	1761	32.1	35	1667
ATCC 29328	*F. magna*	1986	32.1	1 (+1 plasmid)	1842 (chromosome)
ATCC 53516	*“F. nericia”*	1908	32.3	3	1817
GED7760A	*F. magna*	1829	32.1	45	1669*
ACS-171-V-COL3	*F. magna*	1828	32.0	30	1682*
BVS033A4	*F. magna*	1995	31.7	50	1830*
SY403409CC001050417	*F. magna*	2032	32.0	39	1794*
ALB8	*F. magna*	1918	31.9	54	1700*

*Annotated with other tools than Prokka.

### Division of *Finegoldia* strains into two distinct clades based on genome comparison

**Figure 1:**
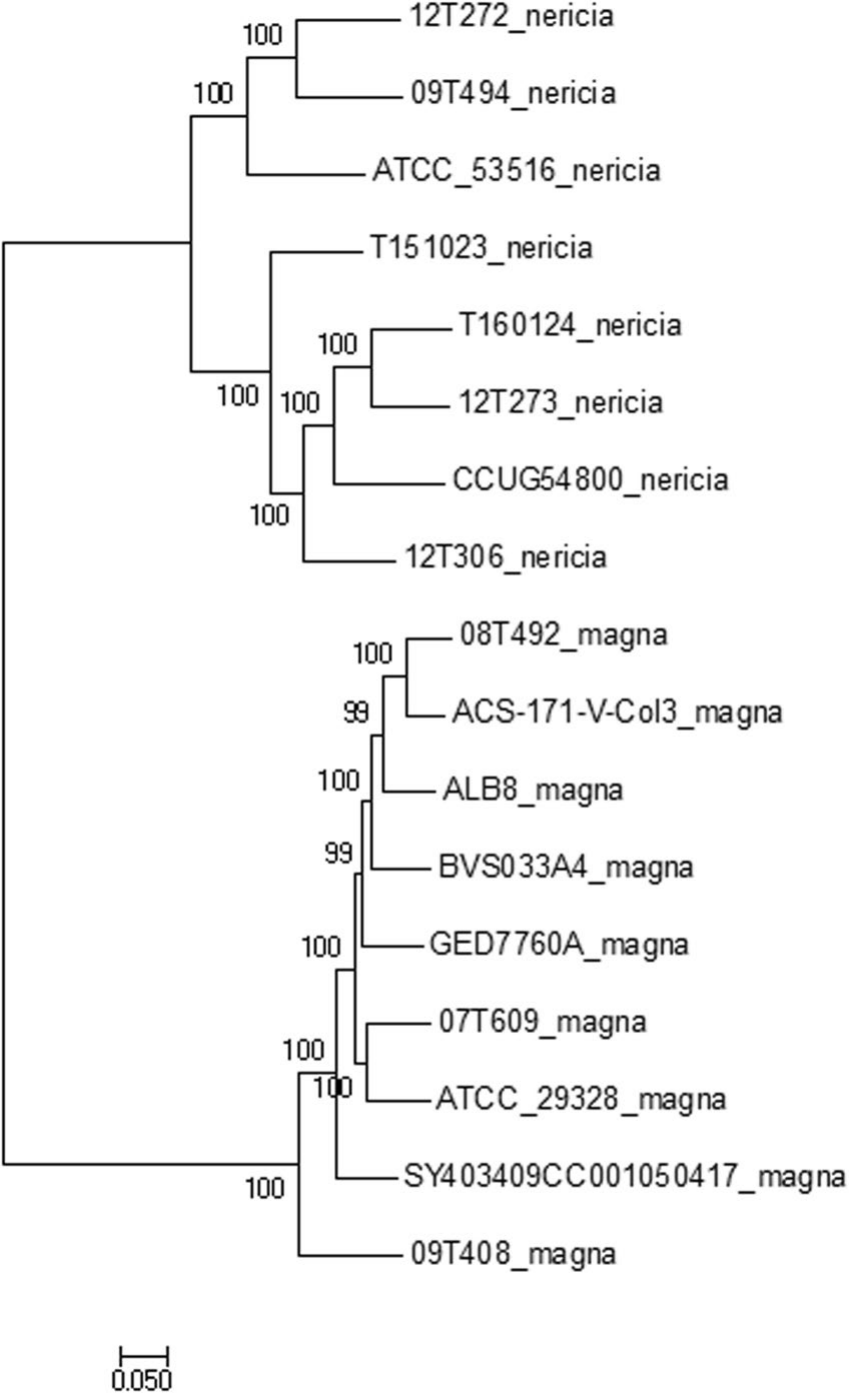
Phylogenomic tree based on core-genome SNPs of all 17 sequenced genomes of *Finegoldia* strains. The program Parsnp was used to align the core genome and call SNPs. The core genome is represented by 43% of the reference genome (ATCC29328); a total of 126,647 reliable core-genome SNPs were used to reconstruct a whole-genome phylogeny. Genomes can be assigned to two main clades; one clade represents *F. magna* strains and the other clade consists of strains of a novel species, here tentatively named *“F. nericia”*.

We previously noticed a large heterogeneity among genomes of *Finegoldia* isolates^15^. A core-genome alignment of all 17 genomes was done and single nucleotide polymorphisms (SNPs) in the core genome were called. In total, 126,647 core-genome SNPs were identified and used for phylogenomic reconstruction, revealing that the 17 genomes can be grouped into two distinct clades (Fig. 1). An additional analysis that determines the average nucleotide identify (ANI) of the core genome showed that the two clades exhibit an ANI of 90.7%, with a strain-specific ANI variation from 90.2% to 91.2%. Such a low ANI indicates that the two clades represent individual species^16^. One clade contains most previously sequenced and studied strains, including ATCC29328 and ALB8; it can be regarded as the classical *F. magna* clade, with an intraclade ANI of 96.6% (variation 95.1–97.4%). The other clade with an intraclade ANI of 94.8% (93.4–96.6%) contains another ATCC strain (ATCC 53516) and mainly strains isolated from patients at the Örebro University Hospital, Sweden^15^. Hereafter, strains of this clade are tentatively named *“Finegoldia nericia”* (Latin for Närke, the Swedish region, where most strains of this species were isolated).

A comparison of 16S rRNA sequences of all isolates revealed a high degree of overall similarity (>99%), but detected a separation of *F. magna* isolates from *“F. nericia”* isolates, due to 5 to 9 *“F. nericia”*-specific SNPs (Figure S1). It also revealed a higher diversification among *“F. nericia”* isolates compared to *F. magna*. Next, we analyzed additional 16S rRNA sequences belonging to *Finegoldia* sp. stored in GenBank (Figure S2). This revealed that most stored sequences cluster with *“F. nericia”*, in particular a group of *Finegoldia* sp. strains isolated from biliary stent biofilms^17^. Again, a higher diversification among *“F*. *nericia”* isolates is detected. Next, we analyzed human microbiome data stored in the IMG (Integrated Microbial Genomes and microbiome samples) database for the presence of *F. magna* and *“F. nericia”* sequences. 3287 and 7068 coding sequences of *F. magna* ATCC29328 and *“F. nericia”* ATCC 53516, respectively, could be identified in the 875 assembled human microbiome datasets present in IMG (data not shown); highest occurrences of *Finegoldia* were detected in retroauricular crease specimens. Taken together, these analyses suggest that among human-associated *Finegoldia* isolates *“F. nericia”* seems to be more prevalent than *F. magna*.

### Strain-specific heterogeneity of *Finegoldia* sp

**Figure 2:**
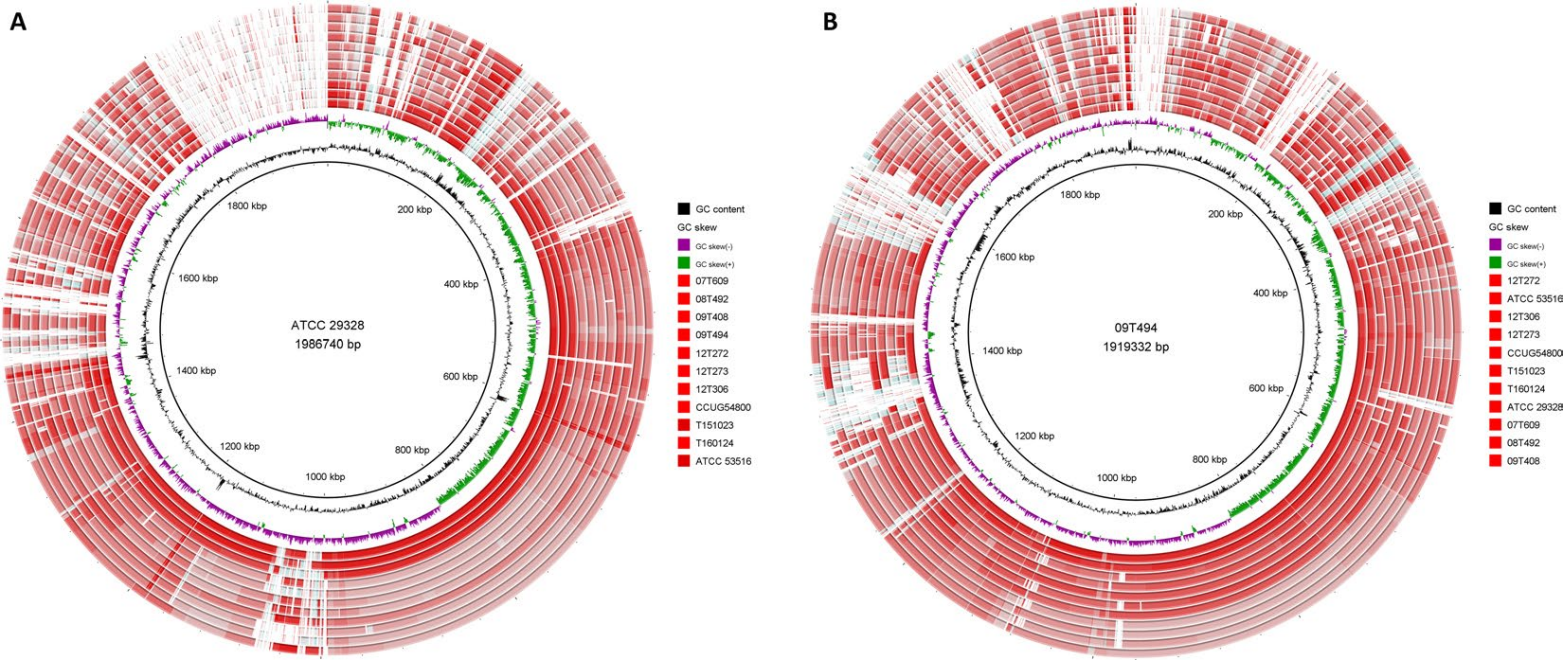
Genome comparison of 12 strains belonging to the genus *Finegoldia*. The two innermost rings represent the G + C-content (black) and the GC-skew (violet/green). (**A**) The reference strain is ATCC29328 (*F. magna*); note the high nucleotide identity (visualized by the dark red color) of the three innermost genomes (belonging all to *F. magna*), and the lower identify to eight strains belonging to *“F. nericia”* (pale red color of the eight outer rings); from inside to outside: strains 07T609, 08T492, 09T408, 09T494, 12T272, 12T273, 12T306, CCUG 54800, T151023, T160124 and ATCC 53516. The large ATCC29328-specific region in the upper left region represents the plasmid pFMC. (**B**) The reference strain is 09T494 (*“F. nericia”*). The seven *“F. nericia”* genomes (inner rings) have a higher nucleotide identify (represented by the darker red color), compared to the four *F. magna* genomes (outer rings); from inside to outside: strains 12T272, ATCC 53516, 12T306, 12T273, CCUG 54800, T151023, T160124, ATCC29328, 07T609, 08T492 and 09T408. There are no signs of *“F. nericia”*-specific genomic regions, but note the five large strain-specific clusters.

To shed further light on the population structure of the *Finegoldia* genus, comparative analyses of 12 genomes (all ten newly sequenced strains and the two ATCC strains) were carried out, including four strains of the *F. magna* clade and eight strains of the *“F. nericia”* clade. A visual comparison of the 12 genomes highlighted the nucleotide identity difference between the two clades (Fig. 2). Interestingly, the plasmid found in ATCC 29328 seems to be specific for this strain as no other sequenced strain contains a similar sequence (Fig. 2A).

No significant differences in genome size and number of CDS was detected between the two clades. In addition, no clade-specific (i.e. *F. magna* or *“F*. *nericia”*-specific) genomic regions were detected, as judged from the BRIG analysis (Fig. 2) and also confirmed by a bidirectional blast approach of all CDS using ProteinOrtho (Table S1). This approach detected 1202 orthologs shared by all 12 genomes, i.e. in average 68% of the CDS of each strain are part of the core proteome. Only very few clade-specific genes are found. Instead, many regions shared by only a few strains can be found, e.g. for strain 09T494 (see also Fig. 2B). In addition, strain-specific regions are present that encode in total 1016 strain-specific CDS, with a large range between the strains, e.g. 187 and 22 strain-specific CDS are detected in strain ATCC29328 and T151023, respectively (Table S1). Among the strain-specific functions are various transport functions (iron, efflux pumps, oligopeptide ABC transporters), surface proteins, restriction-modification systems, conjugative transfer functions, polyketide synthesis, bacteriocins, phage-related functions, antibiotic resistance determinants, and CRISPR/cas systems.

Interestingly, differences in the CRISPR/cas systems were detected between strains of *F. magna* and “*F*. *nericia”* (Table S2). Seven out of nine *F. magna* strains contained one to four CRISPR arrays per strain, containing in average 12 spacers (ranging from 3 to 38 spacers per strain). The repeat “GTTTGAGAATGATGTAATTTCATATAGGTATTAAAC” was specific to strains of *F*. *magna*. In contrast, “*F*. *nericia*” strains carried only one CRISPR array per strain that contained in average 53 spacers (range 14 to 87 per strain). Each strain contained an individual set of spacers in their CRISPR array(s), underling strain-specific heterogeneity, and indicating that each strain has an unique evolutionary history.

In order to evaluate if such strain-specific functions were horizontally acquired we looked at signs of their mobility/acquisition using the IslandViewer program (Figure S3). This program predicted 141 (strain 09T408) to 408 (strain 12T273) genes located in genomic islands that are likely to be horizontally acquired, including many of the above-mentioned strain-specific genes involved in specialization, fitness, survival and interspecies competition (Table S3).

### Host-interacting factors and (putative) virulence factors

**Figure 3:**
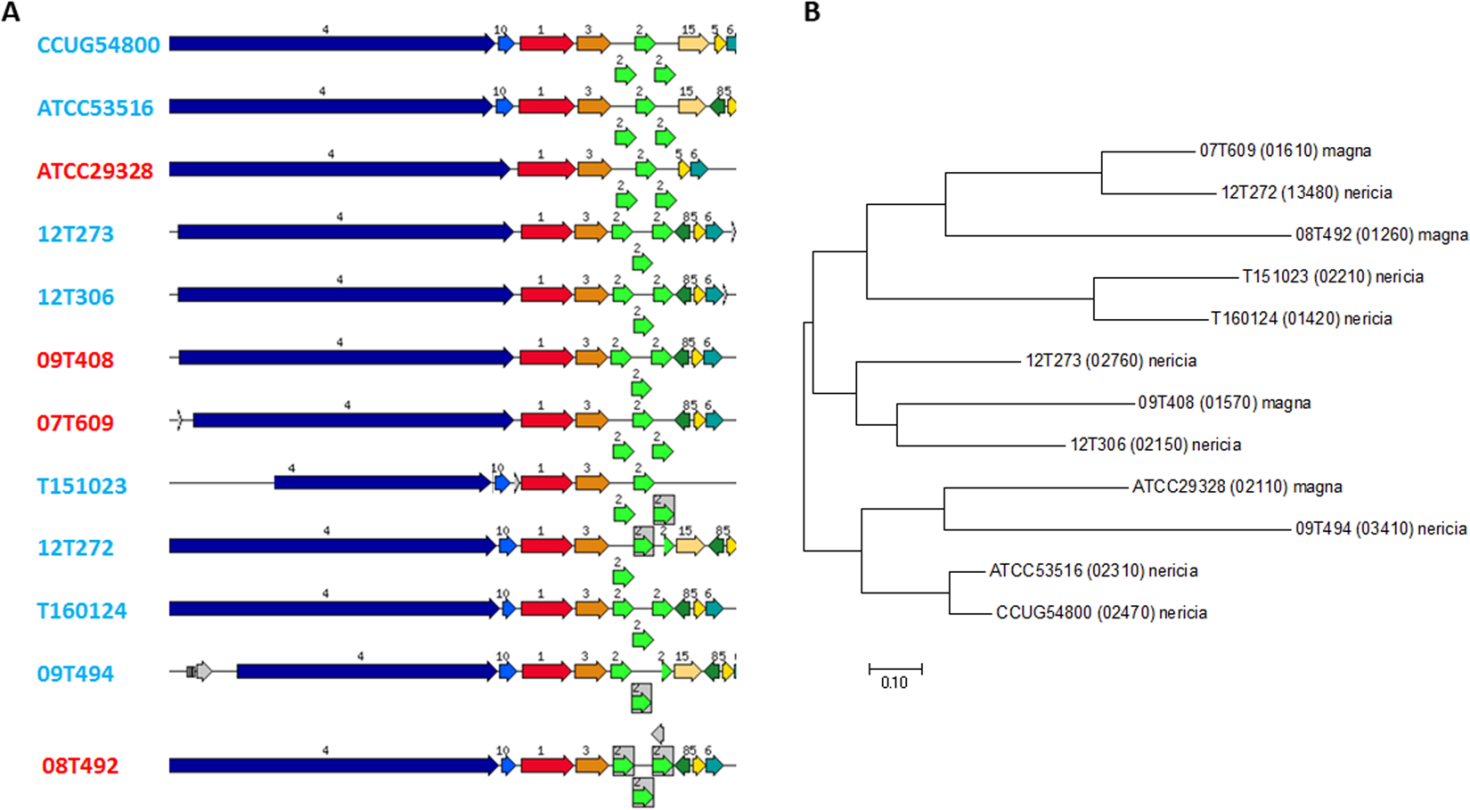
Sortase-dependent pili-encoding genomic loci in the genomes of *Finegoldia* strains. (**A**) Genomic organization of the loci in *F. magna* (red) and *“F. nericia”* (light blue) strains. The three sortases genes are depicted in light green (labelled “2”); the gene for the major pilus subunit Fmp1 is shown in red (labelled “1”) and *fmp*2 is depicted in orange (labelled “3”). Upstream of *fmp1* and downstream of the sortase genes in some strains additional genes (labelled “8”, hemolysin III-family protein and “10”, hypothetical protein) are present. A putative collagen-binding protein, Fmp3 (labelled “4”), is encoded upstream of *fmp*1. (**B**) Phylogenetic tree of Fmp1 of *Finegoldia* strains. Most strains possess an individual Fmp1 variant. The evolutionary history was inferred by using the Maximum Likelihood method, done in MEGA7.

Several host-interacting factors have been described for *F. magna* ^4,5^. Sortase-dependent pili have been identified that are important for adhesion and colonization^13^. A genomic locus that encodes the genes for the pilus subunit Fmp1, the putative subunit Fmp2 and three sortases is conserved among all sequenced genomes (Fig. 3A). There are strain-specific variations up- and downstream of this locus, i.e. some strains possess additional genes upstream of *fmp1* and downstream of the sortase locus, including a gene encoding a hemolysin III family protein. Interestingly, there are strain-specific differences regarding *fmp1*. Almost each strain carries an individual variant and a phylogenetic analysis of Fmp1 on protein level showed no correlation with the phylogenomic analysis of the core genome (Fig. 3B). In contrast, Fmp2 is highly conserved among all strains. Another protein, encoded upstream of *fmp1*, harbors collagen- and fibrinogen binding domains; the collagen-binding domain (COG4932) is also present in Fmp2. We propose that this protein also belongs to the pilus locus and name the corresponding gene *fmp*3.

**Figure 4:**
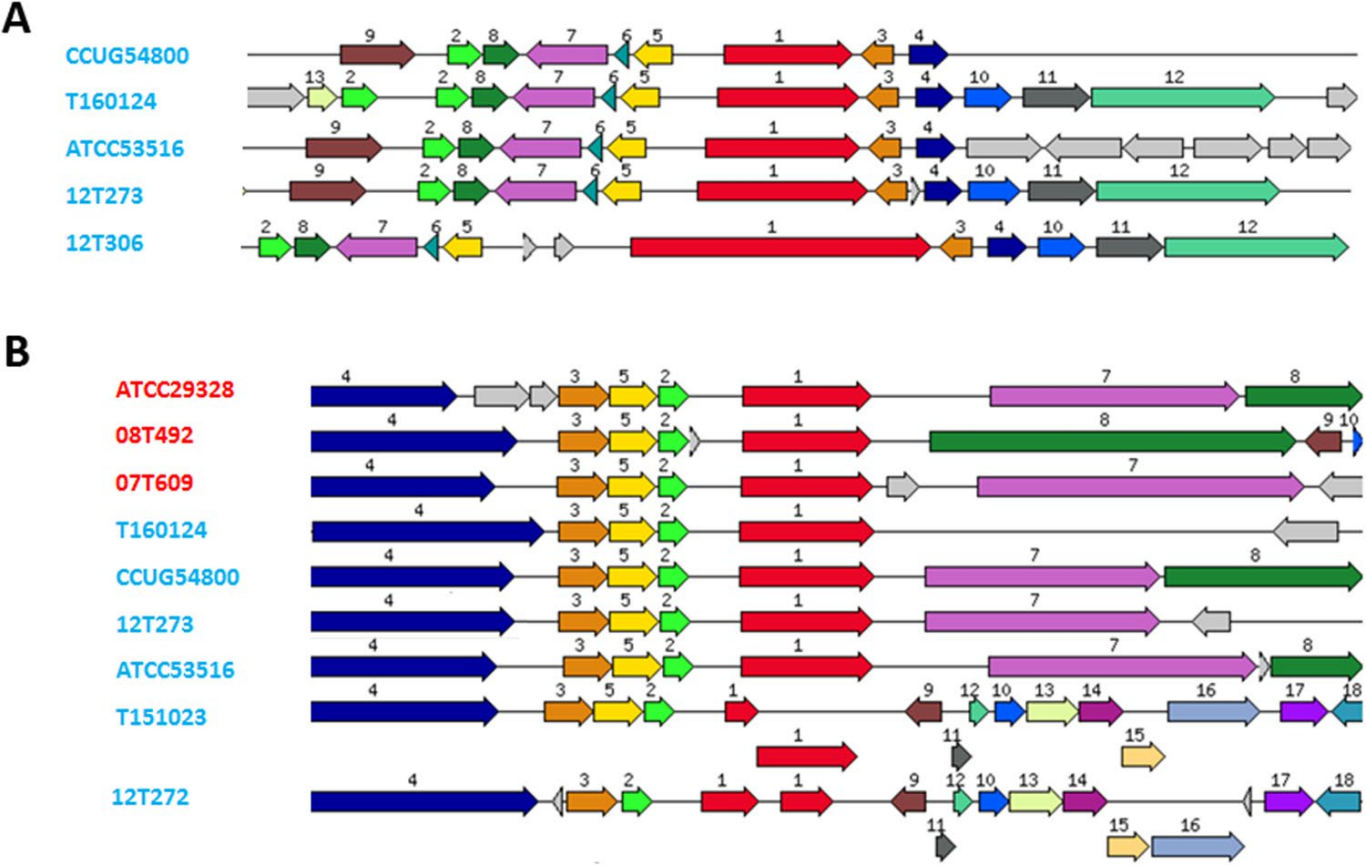
Genomic loci encoding protein L and FAF homologs in *Finegoldia* strains. (**A**) Blast searches with the characterized protein L from strain 312 (locus ID: M86697) was carried out in genome-sequenced *Finegoldia* strains, and homologs (in red, labelled “1”) with high similarity (>50% protein identity) are shown (see also Table S4A). Only *“F. nericia”* strains but not *F. magna* strains contain such homologs with varying lengths. (**B**) Blast searches with the studied FAF protein of strain ALB8 found full-length homologs (in red, labelled “1”) in three *F. magna* (red) and four *“F. nericia”* (light blue) strains. The FAF gene is frameshifted in the two *“F. nericia”* strains T151023 and 12T272.

Protein L is probably the best studied factor of *F. magna* ^8^. It is a superantigen that targets B cells and immunoglobulins. It has been detected in about 10% of the so far tested *Finegoldia* isolates, but varies in size, and numbers of antigen-binding fragment (Fab)-binding domains (B1 domain, pfam02246)^4,8,18^. A blast search with the studied protein L from strain 312 (locus ID: M86697, 719 aa) reveals a few highly similar (protein identity above 50%) proteins with varying sizes (603 to 1417 aa) in a few strains, i.e. strains CCUG54800, T161024, ATCC53516, 12T273 and 12T306 (Fig. 4A). All these are *“F*. *nericia”* strains, which indicates that the B-cell superantigen is more prevalent in this species. The exceptionally large (1417 aa) protein L-like factor in strain 12T306 possesses besides two B1 domains also two GA modules that are known from peptostreptococcal albumin-binding proteins (see below). Many additional proteins with low similarity to protein L, but without containing any apparent B1 domains are encoded in the genome-sequenced *Finegoldia* strains (Table S4A); again, strain-specific variations exist, and a few strains, i.e. 09T408, 09T494 and T151023 possess no such homologs.

*Finegoldia magna* was found to bind to human albumin^9^. A peptostreptococcal albumin-binding protein (PAB) has been studied in the strain ALB8; its activity is linked to an accelerated growth rate^9^. The PAB protein of strain ALB8 (locus ID: X77864) contains GA modules, the albumin-binding domain; the structure of the GA module has been resolved^19^. A Blast search revealed that a close PAB homolog cannot be found in other sequenced *Finegoldia* genomes (Table S4B). Instead, there are several homologs that share up to 50% protein identity; similarities of homologs in *F*. *magna* strains are in average higher compared with homologs in *“F. nericia”* strains. Regarding GA module-containing proteins, in total 16 proteins are encoded in the 12 *Finegoldia* genomes, indicating some redundancy of albumin-binding proteins (Table S4C). Most strains encode two proteins with GA modules, except from strain 09T408 (*F. magna*) and T151023 (*“F. nericia”*).

The cell wall-attached subtilisin-like protease SufA has been shown to degrade several human proteins, such as collagen IV, fibrinogen and antimicrobial peptides such as LL-37 and MIG/CXCL9^10,20,21^. Blast searches determined that most *Finegoldia* strains carry a SufA homolog that is highly similar (protein identity > 75%) to the studied SufA from strain ALB8 (locus ID: DQ679960). Exceptions are the strains 07T609, 09T408 and CCUG54800. A second SufA homolog is present in most strains; it has an average protein identity of 26% (Table S4D).

Another important host-interacting factor is FAF (*F. magna* adhesion factor). Around 90% of *F*. *magna* strains produce this factor, which is responsible for clumping of bacteria and mediates binding to the basement membrane by binding to BM-40^11^. We found that seven (three *F. magna* and four *“F. nericia”* strains) out of the 12 strains encode a FAF homolog similar to the one studied in the ALB8 strain (Fig. 4B). Strain-specific differences exist and frameshift mutations are seen in *“F. nericia”* strains T151023 and 12T272. No homolog can be found in strains 09T408, 09T494 and 12T306 (Table S4E).

### *Finegoldia* genomes encode CAMP factors that are functional

We searched the genome for other putative host-interacting factors and found genes coding for Christie-Atkins-Munch-Petersen (CAMP) factors. CAMP factors can act as co-hemolysins and account for the so-called CAMP reaction, the synergistic lysis of sheep erythrocytes by *Staphylococcus aureus* sphingomyelinase C (beta-toxin) and a CAMP factor^22^.

Genome mining identified two genes encoding CAMP factors in every genome-sequenced *Finegoldia* strain (CAMP1 and CAMP2; Figure S4); each homolog harbors the CAMP factor family domain (Pfam07373). Interestingly, one homolog (CAMP2) per strain harbors an additional bacterial Ig-like domain (Pfam02368). In addition to CAMP1 and CAMP2, four *“F. nericia”* strains contain two additional CAMP factors that substantially differ from the other homologs (CAMP3 and CAMP4; Figure S4). Thus, four out of seven *“F. nericia”* strains harbor four CAMP factor genes.

**Figure 5:**
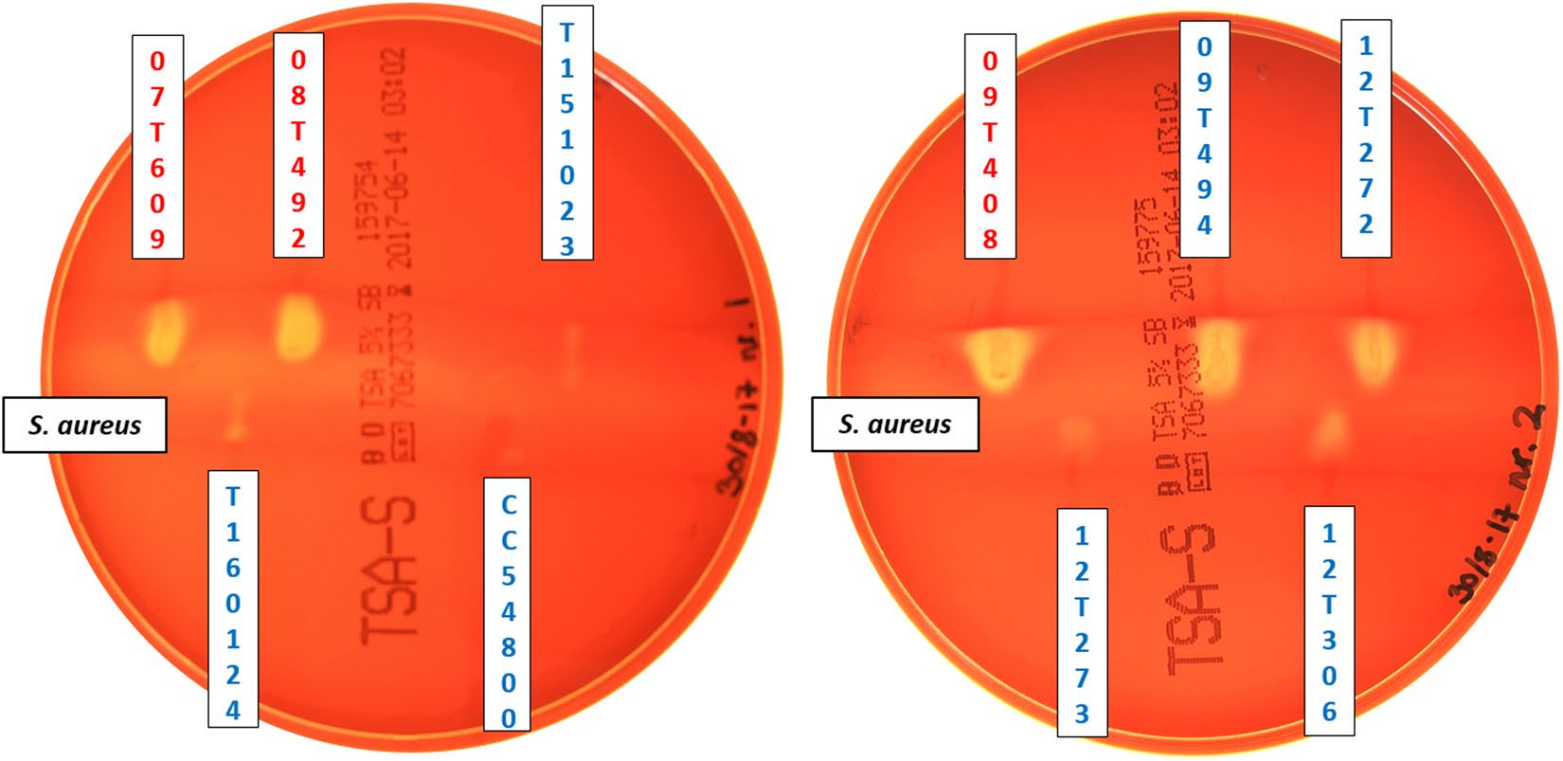
CAMP reaction of *Finegoldia* strains. TSA agar plates with 5% sheep blood were used. *Staphylococcus aureus* is inoculated in the middle streak. A positive CAMP test is indicated by complete erythrocyte lysis at the interface of the *Finegoldia* sp. and the *S. aureus* streaks. All three *F. magna* (in red) strains and two out of seven *“F. nericia”* (in blue) strains showed a strong positive CAMP reaction after two days of anaerobic incubation.

Next, we wanted to know if *Finegoldia* strains produce a positive CAMP reaction. A CAMP agar plate assay shows that some strains are strongly CAMP reaction-positive while for other strains only little co-hemolysis can be detected under the applied cultivation conditions at two and five days of incubation (Fig. 5, Figure S5). All three tested *F. magna* strains were strongly CAMP reaction-positive. Among *“F. nericia”* strains we detected some heterogeneity; two strains were strongly CAMP reaction-positive, four strains were only slightly co-hemolytic, and one strain, CCUG54800, was clearly CAMP reaction-negative. Genomic analysis found that in strain CCUG54800 the CAMP1-encoding gene contained an insertion of a transposase-encoding locus in the 5’-end of the gene, which disrupts the gene (Figure S6).

Our results indicate that CAMP factors of *Finegoldia* sp. can be functional and that there are strain-specific differences in CAMP factor activity.

### Two biochemical tests systems cannot unambiguously differentiate *F. magna* from “*F. nericia*”

Biochemical reactions of GPAC including strains of *Finegoldia* sp. have been described; only little biochemical variation among *Finegoldia* sp. strains was previously reported^2,7^. We tested two commercially available biochemical test systems that are used for the identification of anaerobes. The kit “API® 20A” contains 21 tests, among them 16 tests for carbohydrate utilization. As reported previously^7^, none of the carbohydrates could be metabolized by any *Finegoldia* sp. strain (data not shown). The other reactions (indole, urease, catalase, esculin hydrolysis and gelatin liquefaction) did also not reveal any difference between strains of *F. magna* and “*F. nericia*” (data not shown). The kit “RapID^TM^ ANA II” contains 18 tests for enzymatic activities. Whereas saccharolytic enzymes are lacking, all *Finegoldia* sp. strains produce proteolytic enzymes (Table S5). The reaction detecting phosphatase activity was the only reaction that differed among the strains, with all *F. magna* strains apparently negative and most “*F*. *nericia*” strains positive. However, the interpretation of this test result is limited since the color change was on the border of significance. In conclusion, two existing commercial biochemical test systems, API® 20 A and RapID^TM^ ANA II, cannot unambiguously differentiate *F. magna* from “*F. nericia*”. The biochemical properties of *Finegoldia* sp. need to be further explored with additional biochemical tests.

### Microscopy reveals surface differences between *F. magna* and “*F. nericia*”

**Figure 6:**
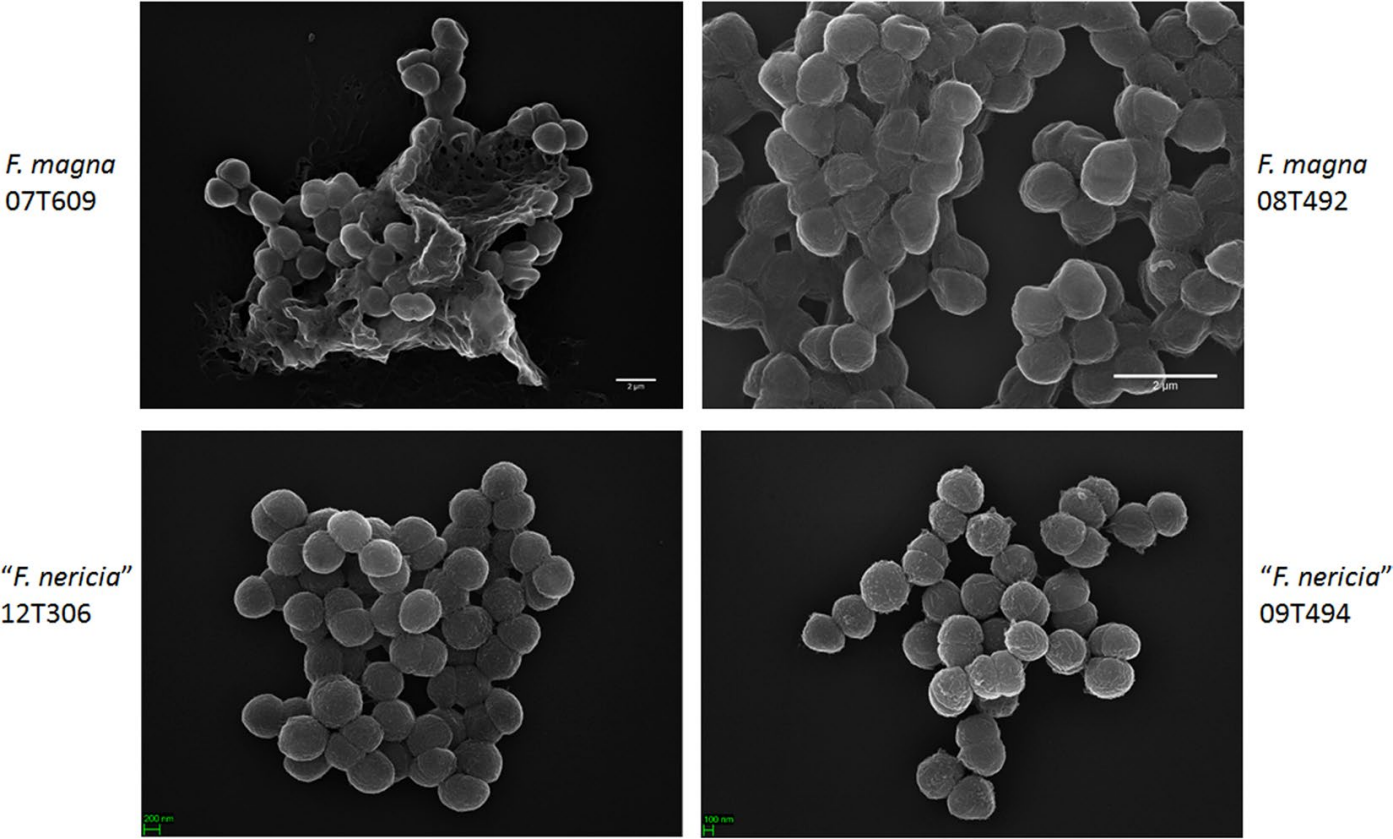
Scanning electron microscopic comparison of *F. magna* and *“F. nericia”*. The upper and lower panels show representative images of *F. magna* 07T609 and 08T492 and *“F. nericia”* 09T494 and 12T306, respectively. Cells of *F. magna* strains are more adherent/aggregative and seem to produce a polymer matrix, in particular strain 07T609; in contrast, cells of “*F. nericia*” are less aggregative.

In an attempt to visualize the morphology and surface structure of *F. magna* and *“F. nericia”* we used scanning electron microscopy (SEM) and atomic force microscopy (AFM) on strains of *F. magna* (07T609, 08T492, and 09T408) and *“F. nericia”* (09T494, 12T272 and 12T306). SEM detected surface differences: cells of *F. magna* were more adherent or aggregative, seemingly producing an extracellular polymer matrix (Fig. 6). This confirms previous work that has visualized an exopolysaccharide matrix in biofilms of *F. magna* (strain FmBs12) by scanning electron microscopy as well as confocal laser scanning microscopy^23^. In the tested *“F. nericia”* strains we could not identify such a matrix; cells seem less aggregative.

**Figure 7:**
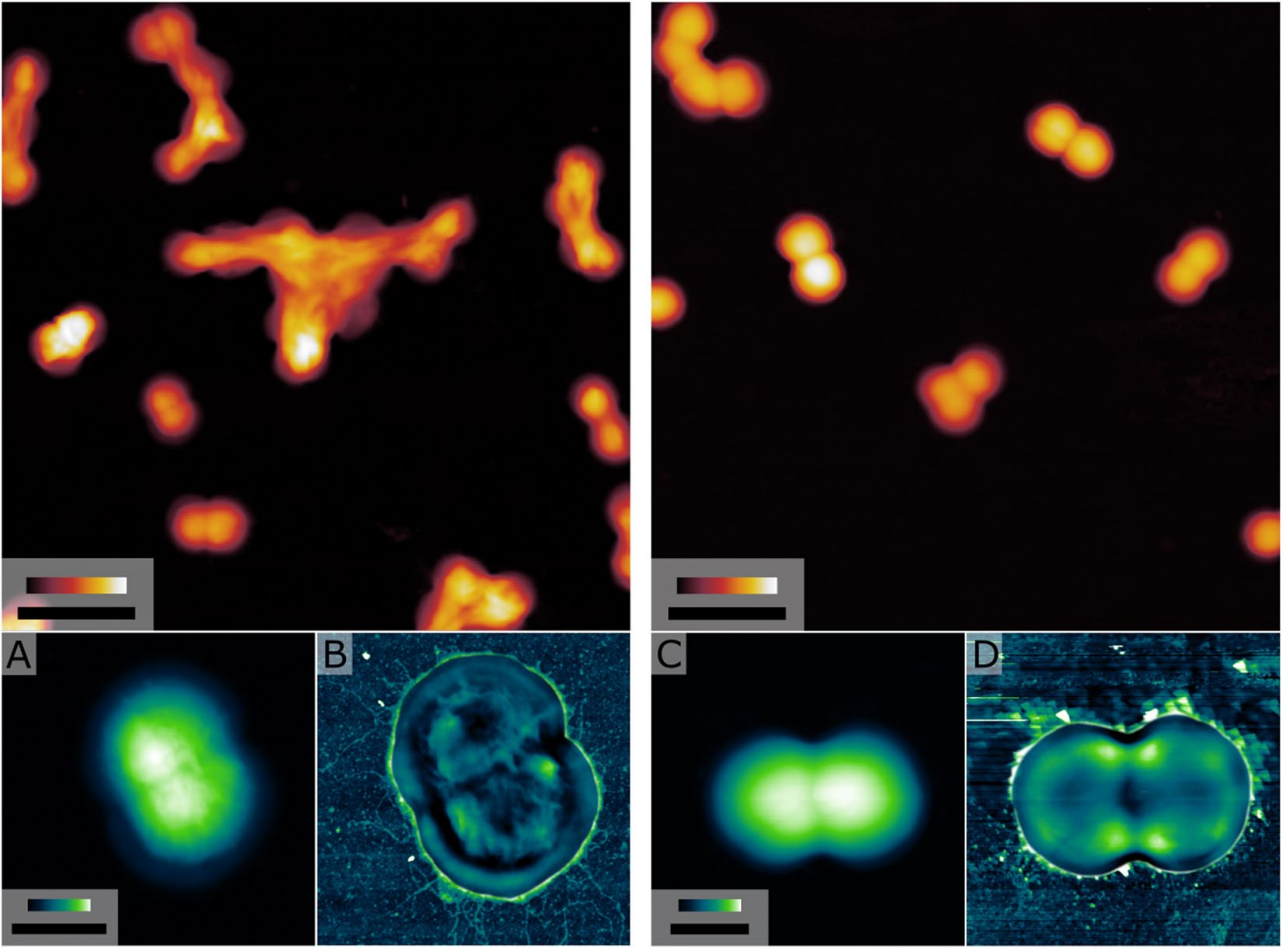
Atomic force microscopic comparison of *F. magna* and *“F. nericia”*. The left and right panels represent images of *F. magna* 07T609 and *“F. nericia”* 12T306, respectively. (**A** and **C**) High-resolution images with assisted advanced soft-touch method with Z scale bars up to 0.3 µm and XY bars indicating 500 nm. (**B** and **D**) Overlay images of high-contrast images of (**A** and **C**), combined with independent horizontal and vertical polynomial removal of (**A** and **C**), solved to the 11^th^ degree. Experiments were performed on three samples from each strain.

AFM confirmed these observations. Moreover, we observed differences regarding cell appendages. Cells of *F. magna* produced longer filamentous appendages that protruded from the entire cell surface; these structures could represent pili or fimbriae (Fig. 7). In contrast, cells of *“F. nericia”* strains had a smoother surface without long protruding appendages. It cannot be ruled out, however, that these AFM observations depend on the bacterial cultivation conditions and on the sample processing steps.

## Discussion

The present work identified substantial genomic diversity among isolates of the genus *Finegoldia*. Until now, only one species has been assigned to this genus, i.e. *F*. *magna*. We here propose to differentiate strains of this genus into two species, *F. magna* and a newly assigned species, tentatively named *“F. nericia”*. This proposal is based on the observation of distinct core genomes, with an ANI of 90.7% between the two species. According to recommendations to define species based on DNA similarity, an ANI below 95% indicates species separation^16^.

Other observations do not support a strict species separation of *F. magna* and *“F. nericia”*. To date no biochemical test was found that can clearly separate *F. magna* from *“F. nericia”*. Moreover, the average genome size and the G+C content are similar in both species, and the 16 S rRNA sequences, albeit different, show relatively little variation. No species-specific genomic regions or islands were identified (with the exception of CRISPR sequences) and the total number of *F. magna*- and *“F. nericia”*-specific genes is very low. In addition, previously studied virulence determinants of *Finegoldia* strains, such as FAF, SufA and PAB are found in some but not all strains of both, *F. magna* and *“F. nericia”*. An exception is protein L, a B-cell superantigen: close homologs are only found in *“F. nericia”* strains. This raises interesting questions regarding distinct virulence potentials of *F. magna and “F. nericia”*.

Microscopic observations support the existence of distinct species among *Finegoldia* isolates. The investigated *F. magna* strains showed cell appendages, possibly pili, and produced an extracellular polymer matrix, whereas no such structures were detected in the tested *“F. nericia”* strains. It cannot be ruled out, however, that there are atypical strains in both species. All genomes of both species contain a pili-encoding locus, and it is not obvious why some strains are apparently not producing pili. Further analyses are needed to clarify differences on the transcriptional level. Previously, sortase-dependent pili have been detected in the *F. magna* strain ALB8 by transmission electron microscopy^13^; the sortases were assigned to class C sortases due to their similarity with *Streptococcus pneumoniae*, and the major pilus subunit, Fmp1, was identified. Here, we showed that this protein is present in all sequenced *Finegoldia* strains, but we detected substantial strain-specific genetic differences, which indicates a strong selective pressure, possibly to introduce antigenic variation. Interestingly, it was previously noted that the antiserum raised against Fmp1 from strain ALB8 was unable to bind to any surface protein of other *F. magna* isolates, underlining the strain-specific nature of Fmp1^13^. This strain-specific variation is also seen for the newly assigned Fmp3, a protein with a fibrinogen-binding domain and multiple copies of a collagen-binding domain; the latter is also found in the minor pilus subunit Fmp2. The *fmp*3 gene is located immediately upstream of *fmp*1. These findings suggest a functional link of Fmp3 with the pilus, and could indicate that the pilus has strong adhesive properties. In fact, pili of *F. magna* were found to bind to keratinocytes in the epidermal layer of human skin^13^.

We also identified host-interacting factors that were previously not described for *Finegoldia*, such as CAMP factors. Such proteins have been described in a few other species, i.e. group A and group B streptococci (GAS, GBS) and *Propionibacterium acnes* ^24–26^. All genomes of *Finegoldia* strains encoded multiple CAMP factors, two (*F. magna*) to four (*“F. nericia”*) homologs per genome. This CAMP factor redundancy is also seen in *P. acnes* that possesses five CAMP factor genes^25,26^, and indicates a crucial role for survival in and/or colonization of host tissue. Interestingly, both *P. acnes* and *Finegoldia* sp. are often isolated from human skin as well as soft and deep tissue sites. The precise role of CAMP factors in virulence is not known but several lines of evidence suggest that they have a role in escaping macrophage-mediated host immunity, thus allowing the bacterium to grow and spread in human tissues. A recent study showed that the CAMP factor of GAS induced vacuolation and reduced the phagocytic activity of macrophages without causing cell death^27^. Another study suggested that one of the CAMP factors of *P. acnes* acts together with a membrane-associated mammalian sphingomyelinase and facilitates phagosomal escape of *P. acnes* in macrophages^28^. It was also suggested that CAMP factors have Ig-binding capabilities, since sequence comparison identified similarities between the Fc-binding region of *S. aureus* protein A and *P. acnes* CAMP factors^26^. This is reminiscent of a study that reported unspecific Ig-binding activity of CAMP factor of streptococci^29^; however, in a later study this could not be confirmed^30^. Interestingly, the here identified CAMP2 protein that is present in all *Finegoldia* strains, contains an Ig-like domain, which might indicate a role in immunoglobulin interaction. Diversity among strains of *Finegoldia* was found regarding the CAMP reaction, with strong (*F. magna* and *“F. nericia”* strains), weak (*“F. nericia”* strains) and no (one *“F. nericia”* strain) CAMP reaction-positive strains. Analyses of the CAMP genes in the genome of the CAMP reaction-negative strain *“F. nericia”* CCUG54800 revealed an inactivation of the *camp*1 gene by an insertion of a mobile element that encodes transposases. This strongly suggests that CAMP1 is responsible for the co-hemolytic effect on sheep erythrocytes. Less clear is the reason for the difference between strains that exhibited either strong or weak CAMP reactions. A possible reason could be related to the transcriptional control of CAMP factor expression. It was shown in GBS that CAMP gene expression is controlled by the two-component system CsrRS (or CovRS) that controls the expression of multiple virulence factors in GBS^31^. Future work needs to be done to unravel the role of CAMP factors in *Finegoldia* and to assess the significance of CAMP reaction differences among strains.

Many open questions regarding species-specific traits remain. Currently, it is not clear if a single human individual is colonized with both, *F. magna* and *“F. nericia”*, and if a specific clone is predominating. The analyses of 16 S rRNA sequences and microbiome databases showed that *“F. nericia”* is more commonly found than *F. magna* in human-associated microbiological samples. Interestingly, a cohort of *“F. nericia”* strains were obtained from biliary stent biofilms^17^, and we mainly isolated *“F. nericia”* from orthopedic joint-implant associated infections^15^. This indicates that *“F. nericia”* might be more prevalent in nature and/or has a higher virulence potential compared to *F. magna*.

Taken together, the genomes of *Finegoldia* strains encode arrays of host-interacting factors, including factors involved in host molecule-binding and recognition of adhesive matrix molecules. A multitude of proteins with albumin- and immunoglobulin-binding domains were found and other host factor-binding proteins, such as collagen- and fibrinogen-binding proteins. These factors serve important functions in colonization of and survival in human tissue. They can also be part of a camouflage strategy to evade the host immune responses by covering *Finegoldia* with host-derived components rendering it into “a wolf in sheep skin”. In addition, we detected a large heterogeneity in the genomes of the *Finegoldia* population and propose to differentiate two species. This might reflect different ecological niches of *Finegoldia*: they are found at several different body sites, including skin, oral cavity and the urogenital and gastrointestinal tracts. As such, although universal residents of the human body, they likely evolved tissue-specific colonization strategies, while avoiding the human defense systems. Future work needs to investigate species- and strain-specific host-interaction profiles, and needs to map and compare *Finegoldia* populations from different tissue sites.

## Methods

### Bacterial strains

Ten *Finegoldia* strains have been used in this study. Nine isolates (07T609, 08T492, 09T408, 09T494, 12T272, 12T273, 12T306, T151023, T160124) were obtained from orthopedic implant infections collected at the Department of Laboratory Medicine, Clinical Microbiology, Örebro University Hospital, Sweden from 2004 to 2016^15^. One strain, CCUG 54800, originally isolated from human synovial fluid, was obtained from a public strain collection in Sweden (CCUG: http://www.ccug.se/) for comparative reasons.

All ten strains were identified as *F*. *magna* by using MALDI-TOF mass spectrometry (MicroflexLT and Biotyper 3.1; Bruker Daltonics) with identification scores of 1.8 or higher. All strains were grown under anaerobic conditions on FAA plates (4.6% LAB 90 Fastidious Anaerobe Agar, LAB M, Heywood, UK) supplemented with 5% horse blood (v/v) and incubated at 37 °C in anaerobic conditions (10% H2, 10% CO2, 80% N2) for 4 to 7 days.

### Genome sequences

Information about the genomes used in this study can be found in Table 1. The GenBank accession numbers of the draft genome sequences of the ten strains isolated in Sweden are: NDYJ00000000 (strain 07T609), NDYI00000000 (strain 08T492), NDYH00000000 (strain 09T408), NDYG00000000 (strain 09T494), NDYF00000000 (strain 12T272), NDYE00000000 (strain 12T273), NDYD00000000 (strain 12T306), NDYC00000000 (strain CCUG54800), NDYB00000000 (strain T151023), NDYA00000000 (strain T160124). Seven genomes of *F. magna* strains that have been sequenced previously by others have been used for comparative purposes. Their names and GenBank accession numbers are: ACS-171-V-Col3 (AECM01), ATCC 29328 (AP008971, AP008972), ATCC 53516 (ACHM02), GED7760A (LRPW01), BVS033A4 (AEDP01), SY403409CC001050417 (AFUI01), ALB8 (JDVC01).

### Phylogenomic and other bioinformatic analyses

For phylogenomic analyses, the core genome was identified and aligned with Parsnp, a program that is part of the Harvest software package^32^. Parsnp aligns microbial genomes based on a suffix graph data structure; the output is a core-genome alignment that contains all SNPs, Indels, and structural variation within the core genome. Parsnp is further quality-filtering SNPs; only reliable core-genome SNPs are considered for reconstruction of the whole-genome phylogeny that can be visualized with Gingr, another program of the Harvest software package^32^.

To calculate the average nucleotide identity (ANI) between genomes the program JSpecies was used^16^. Jspecies calculates the ANI between the genomes in a pairwise comparison using BLAST.

Gene prediction and annotation of all genomes were done with RAST and Prokka^33,34^. Phylogenetic trees were built in Mega v7^35^. For comparative genome analyses and visualization, the programs BRIG and RAST were used^33,36^. To predict genomic island and horizontally acquired genes the tool IslandViewer 4 was used, a computational tool that integrates four different genomic island prediction methods: IslandPick, IslandPath-DIMOB, SIGI-HMM, and Islander^37^. To determine orthologous genes among the *Finegoldia* strains we used the tool ProteinOrtho^38^. For the analysis of human microbiome datasets the IMG database and the IMG/MER tools were used (https://img.jgi.doe.gov/cgi-bin/mer/main.cgi). Only assembled human microbiome datasets were search for the presence of coding sequences of *F. magna* ATCC29328 and *“F. nericia”* ATCC 53516 using the “Genome versus Metagenomes” search function. The threshold was set to 90% amino acid identity.

### CAMP reaction

The CAMP (Christie-Atkins-Munch-Petersen) plate assay was used as previously described^22^, with small modifications. Trypticase soy agar plates with 5% sheep blood were used. Plates were incubated for two and five days under anaerobic conditions at 37 °C.

### Biochemical tests

Two test systems, API® 20A (Biomerieux) and RapID^TM^ ANA II (Remel/Thermo Fisher), were used according to the instructions of the manufacturers. In brief, *Finegoldia* strains were grown on blood agar plates for 4 days under anaerobic conditions; cells were harvested and resuspended in the test system’s recommended inoculation fluids in the desired densities. For the RapID^TM^ ANA II kit the bacterial suspension had a visual turbidity equal to a no. 4 McFarland turbidity standard. After inoculation, the RapID^TM^ ANA II panel was incubated at 37 °C for 5 h. The inoculated API 20 A kit panel was incubated for 24 h. Additional substances were added after inoculation, and results were interpreted as described in the instructions of the manufacturers.

### Scanning electron microscopy

*Finegoldia* sp. strains were incubated on blood agar plates for four days under anaerobic conditions. Cells were harvested and resuspended in 1 mL PBS, and washed twice in PBS with gentle centrifugation (1000 rpm, 5 min). Bacterial cells were then fixed with 2.5% glutaraldehyde, post-fixed using repeated incubations with 1% osmium tetroxide/1% tannic acid, dehydrated with a graded ethanol series, critical point dried and coated with 3 nm platinum/carbon. Specimens were analyzed in a Leo 1550 scanning electron microscope.

### Atomic force microscopy

*Finegoldia* sp. strains were incubated on blood agar plates for four days under anaerobic conditions. Cells were harvested and resuspended in 1 mL PBS, and washed twice in PBS with gentle centrifugation (1000 rpm, 5 min) to avoid shredding of possible cell appendages. Cells were then diluted and transferred to Superfrost Plus Microscope slides (ThermoFisher Scientific), and cells were left to adhere onto the surface of the sildes for at least 30 min. The slides were afterwards washed vigorously with water to remove any loosely attached cells, and finally air-dried before microscopy.

A NanoWizard IV (JPK Instruments, Germany) combined with an inverted optical microscope (Zeiss Axiovert 200M, Zeiss, Germany) and a DimensionIcon (Bruker, USA) atomic force microscope (AFM) were used to record raw AFM data at 512 pixels per line, with 1 Hz scanning speed. Dynamic Nanomechanical Mapping^39^ in air was performed for soft-touch imaging with direct force control, using ScanAsyst Air cantilevers (Bruker) with nominal spring constant of 0.4 N/m. Raw AFM data was processed using Gwyddion^40^ and images made with GIMP and Inkscape.

### Data Availability

The datasets analysed during the current study are available in the GenBank repository (https://www.ncbi.nlm.nih.gov/genbank/).

## Electronic supplementary material

Supplementary information
