# Supplementary material for: Pan-genome analysis of the genus Finegoldia identifies two distinct clades, strain-specific heterogeneity, and putative virulence factors
Source: Sci Rep. 2018 Jan 10;8:266. doi: 10.1038/s41598-017-18661-8 (PMC5762925; doi:10.1038/s41598-017-18661-8)
Supplement: Supplementary file 1 — Supplementary information [file 41598_2017_18661_MOESM1_ESM.pdf]

Supplementary Information

Pan-genome analysis of the genus *Finegoldia* identifies two distinct clades, strain-specific heterogeneity, and putative virulence factors

Holger Brüggemann, Anders Jensen, Seven Nazipi, Hüsnü Aslan, Rikke Louise Meyer, Anja Poehlein, Elzbieta Brzuszkiewicz, Munir Al-Zeer, Volker Brinkmann, Bo Söderquist

Content:

Figure S1: Phylogenetic tree of 16S rDNA sequences extracted from draft genomes of *Finegoldia*

Figure S2: Phylogenetic tree of 16S rDNA sequences of *Finegoldia* isolates extracted from GenBank

Figure S3: Predicted genomic islands in *Finegoldia* genomes

Figure S4: Phylogenetic tree of all putative CAMP factors of *Fingoldia* strains

Figure S5: CAMP reaction of *Finegoldia* strains at elongated cultivation time

Figure S6: Insertional inactivation of *camp*1 in strain CCUG54800

Table S1: Bidirectional blast of all coding sequences of 12 *Finegoldia* genomes

Table S2: CRISPR/cas systems in *Finegoldia* sp.

Tables S3A-S3L: Predicted genomic islands in the genomes of *F. magna* and *"F. nericia"*

Table S4A-S4E: Blast results showing homologs of host-interacting factors in 12 *Finegoldia* genomes

Table S5: *Finegoldia* sp. strains tested in the RapID^{TM} ANA II system

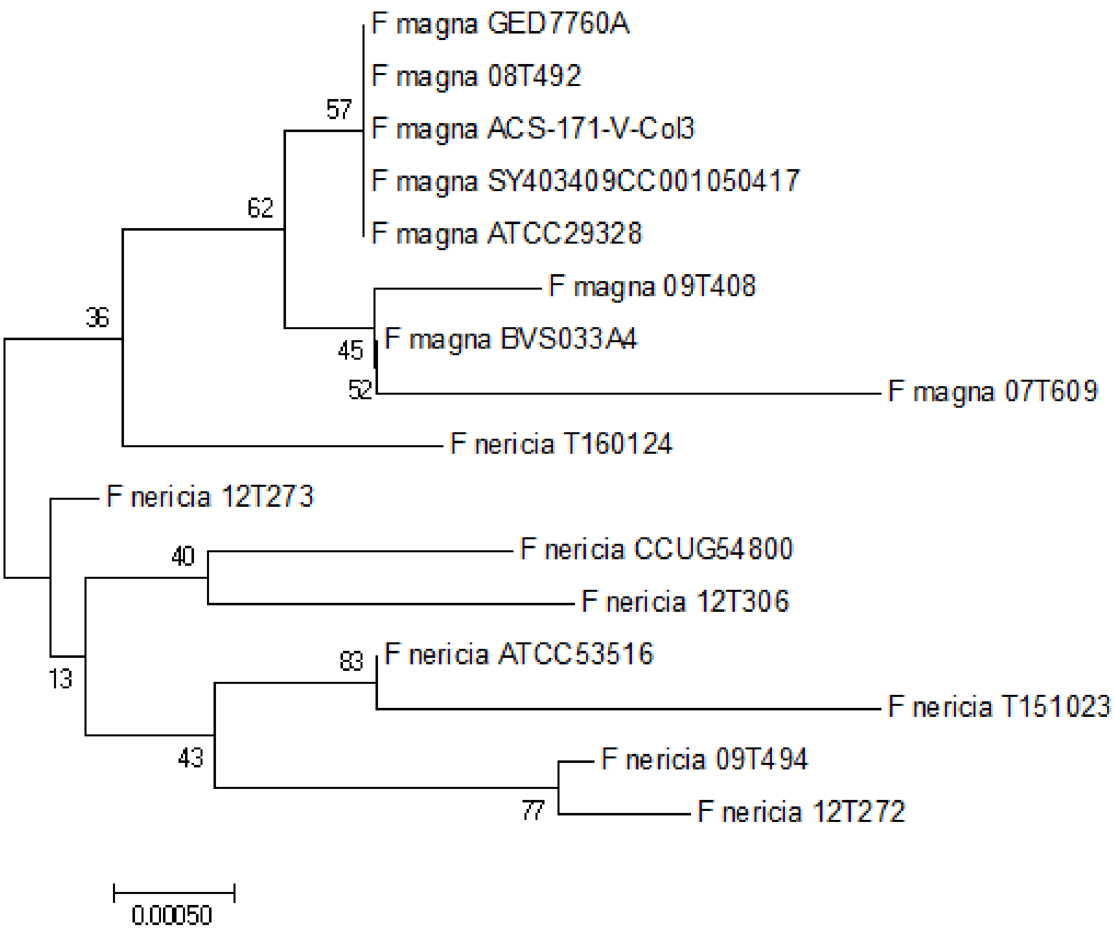

Figure S1: Phylogenetic tree of 16S rDNA sequences extracted from draft genomes of *Finegoldia*
The analysis involved 16 nucleotide sequences. For one genome, *F. magna* ALB8, the 16S rDNA sequence is incomplete. There is an overall high similarity (in average 99%) between the 16S rDNA sequence of strains of *F. magna* and "*F. nericia*": there are 5 to 9 SNPs in the 16S rDNA of "*F. nericia*" strains compared to the cluster of *F. magna* strains, including the reference strain *F. magna* ATCC29328. The phylogenetic analysis was done with MEGA7. The evolutionary history was inferred using the Neighbor-Joining method. The percentage of replicate trees in which the associated taxa clustered together in the bootstrap test (250 replicates) are shown next to the branches. The tree is drawn to scale, with branch lengths in the same units as those of the evolutionary distances used to infer the phylogenetic tree. The evolutionary distances were computed using the Maximum Composite Likelihood method and are in the units of the number of base substitutions per site.

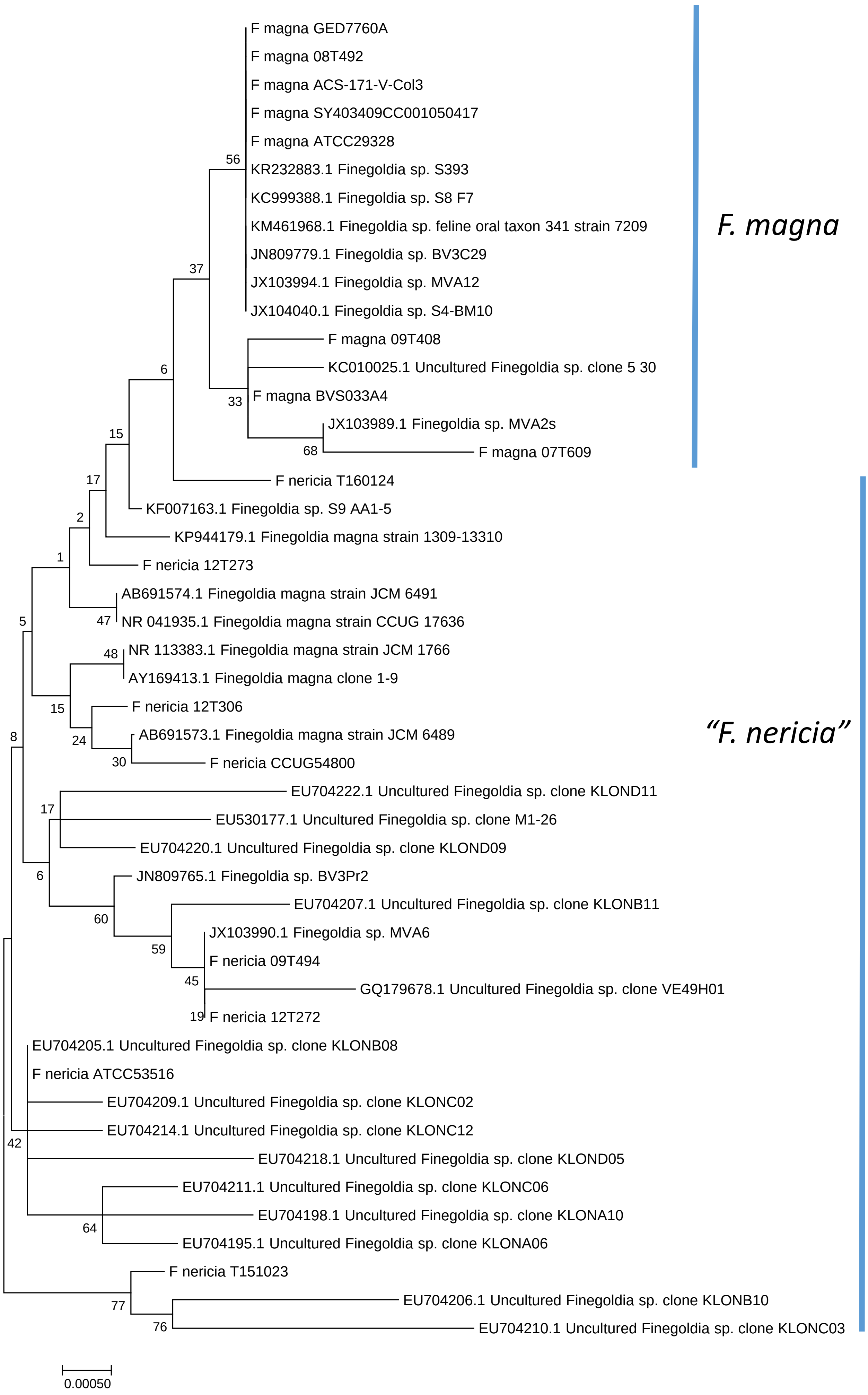

Figure S2: Phylogenetic tree of 16S rDNA sequences of *Finegoldia* isolates extracted from GenBank
All deposited 16S rDNA sequences assigned to *Finegoldia* were taken from GenBank that had a >85% overlap with the 16S rDNA sequence (*rrnA*) of ATCC29328. 47 sequences were compared. The phylogenetic analysis was done with MEGA7. The evolutionary history was inferred using the Neighbor-Joining method. The percentage of replicate trees in which the associated taxa clustered together in the bootstrap test (250 replicates) are shown next to the branches. The tree is drawn to scale, with branch lengths in the same units as those of the evolutionary distances used to infer the phylogenetic tree. The evolutionary distances were computed using the Maximum Composite Likelihood method and are in the units of the number of base substitutions per site.

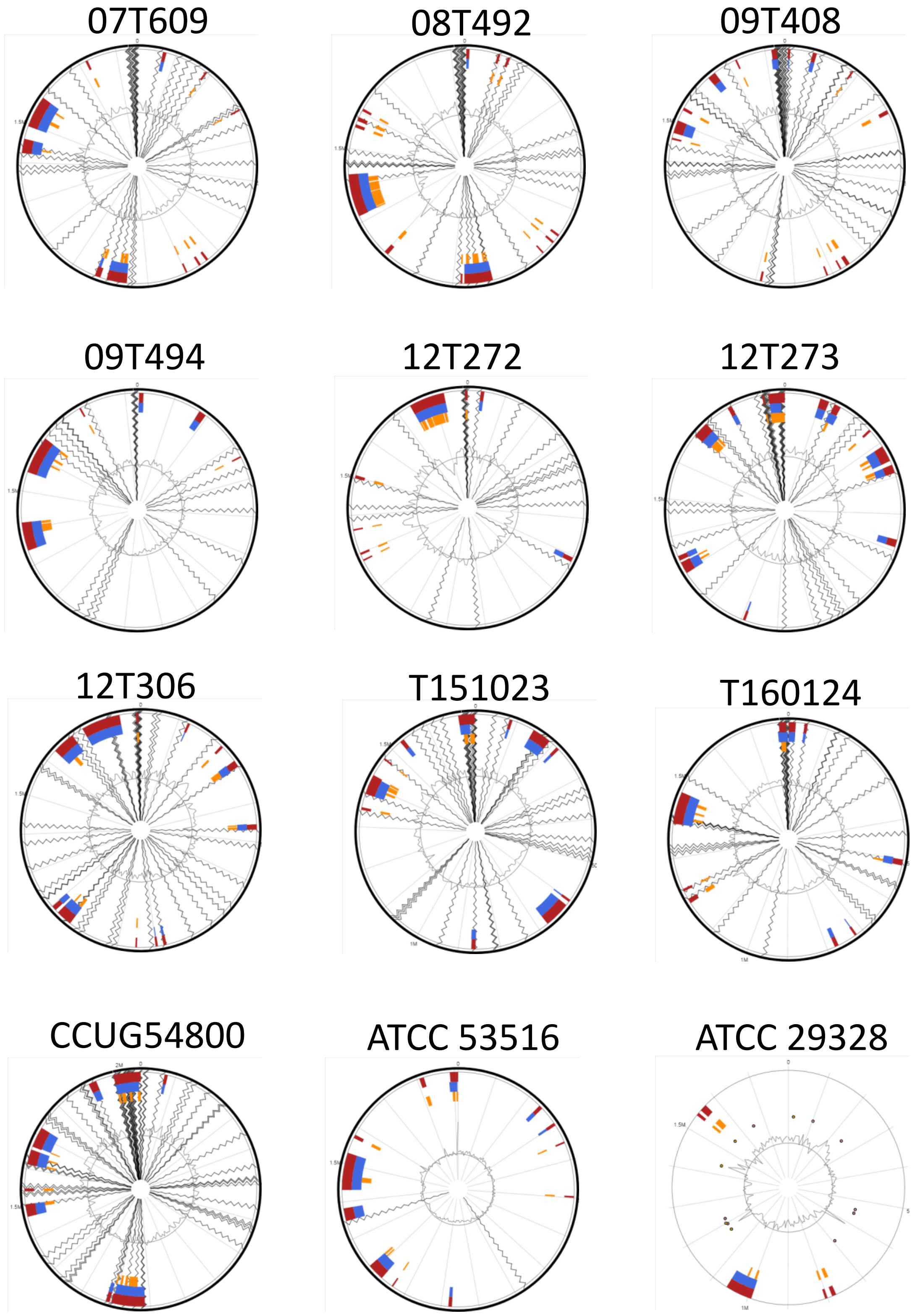

Figure S3: Predicted genomic islands in *Finegoldia* genomes

All analyzed 12 *Finegoldia* genomes contain predicted genomic islands, but with strain-specific differences. The color depicts the prediction method: blue, IslandPath-DIMOB; orange, SIGI-HMM and dark red, integrated. All genes found in these islands are shown in table S3.

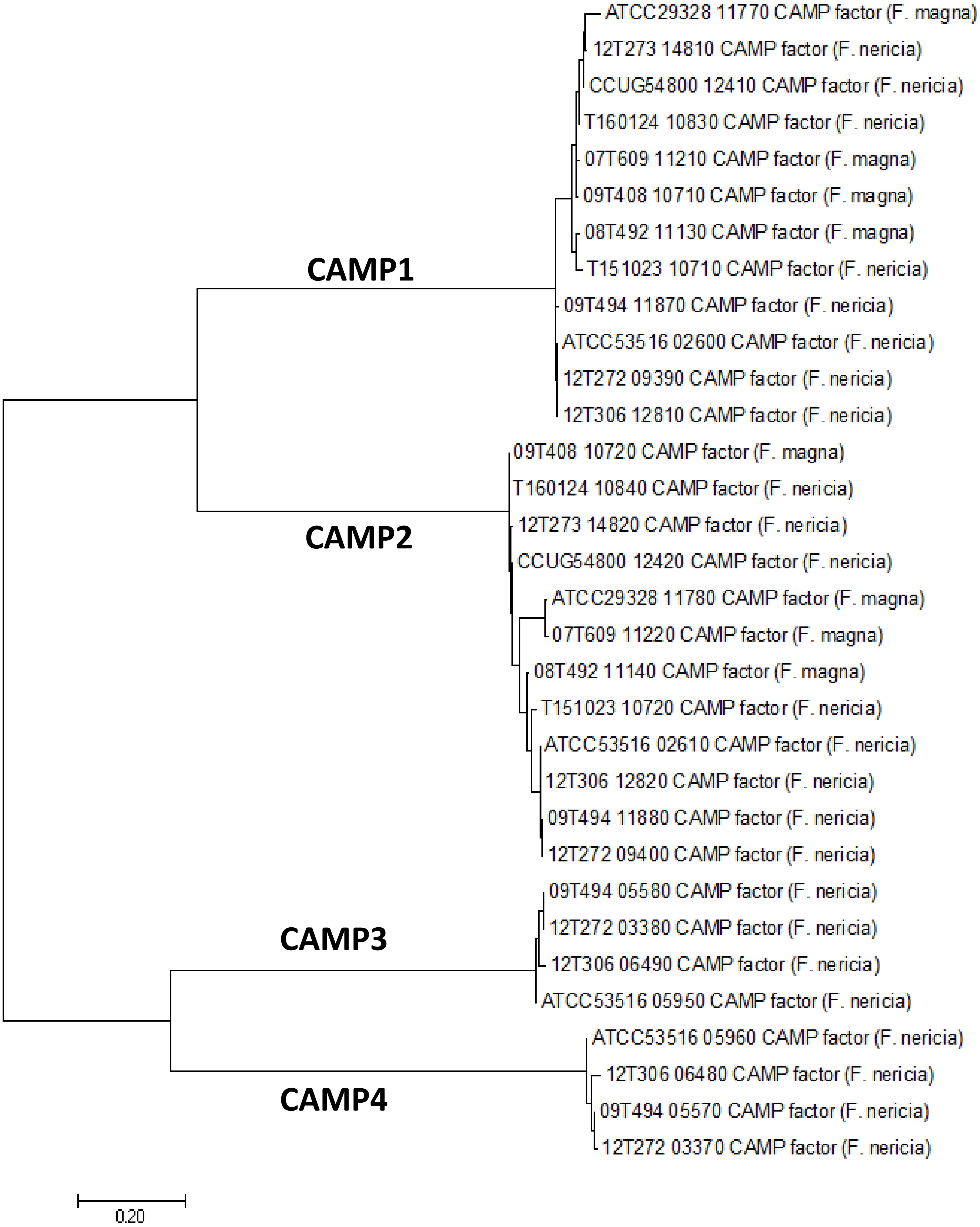

Figure S4: Phylogenetic tree of all putative CAMP factors of *Finegoldia* strains

Each strain possesses a least two distinct CAMP factors whose genes are in direct vicinity. Four out of seven *"F. nericia"* strains possess two additional CAMP factors; again, the genes are located next to each other. The tree was created in MEGA7, using the Maximum Likelihood method.

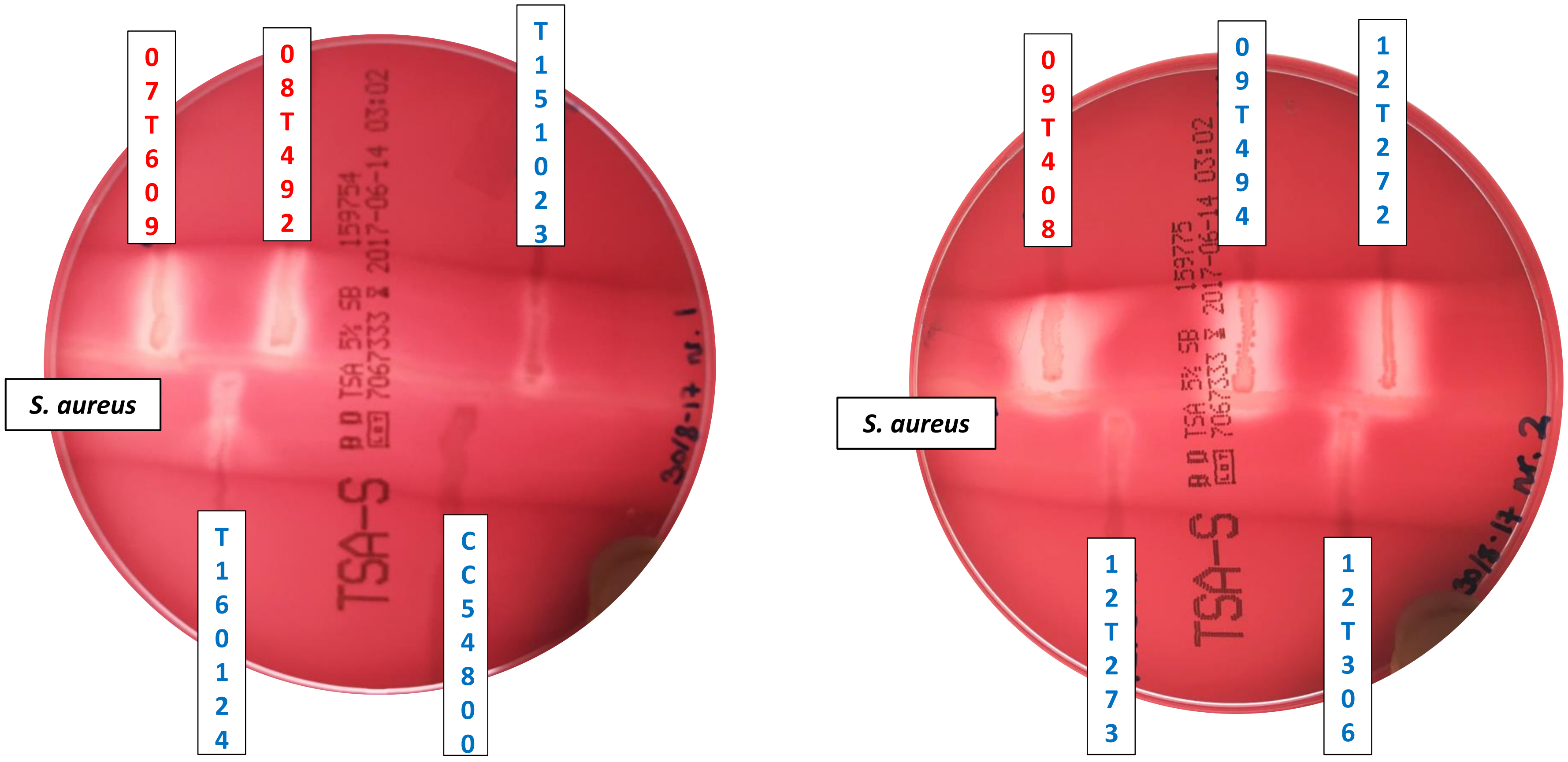

Figure S5: CAMP reaction of *Finegoldia* sp. strains at elongated cultivation time

TSA agar plates with 5% sheep blood was used. *Staphylococcus aureus* is inoculated in the middle streak. A positive CAMP test is indicated by complete erythrocyte lysis at the interface of the *Finegoldia* sp. and the *S. aureus* streaks. All three *F. magna* (in red) strains and two out of seven *"F. nericia"* (in blue) strains showed a strong positive CAMP reaction after five days of anaerobic incubation. One strain (CCUG54800) is negative in the CAMP reaction (see also Figure S6).

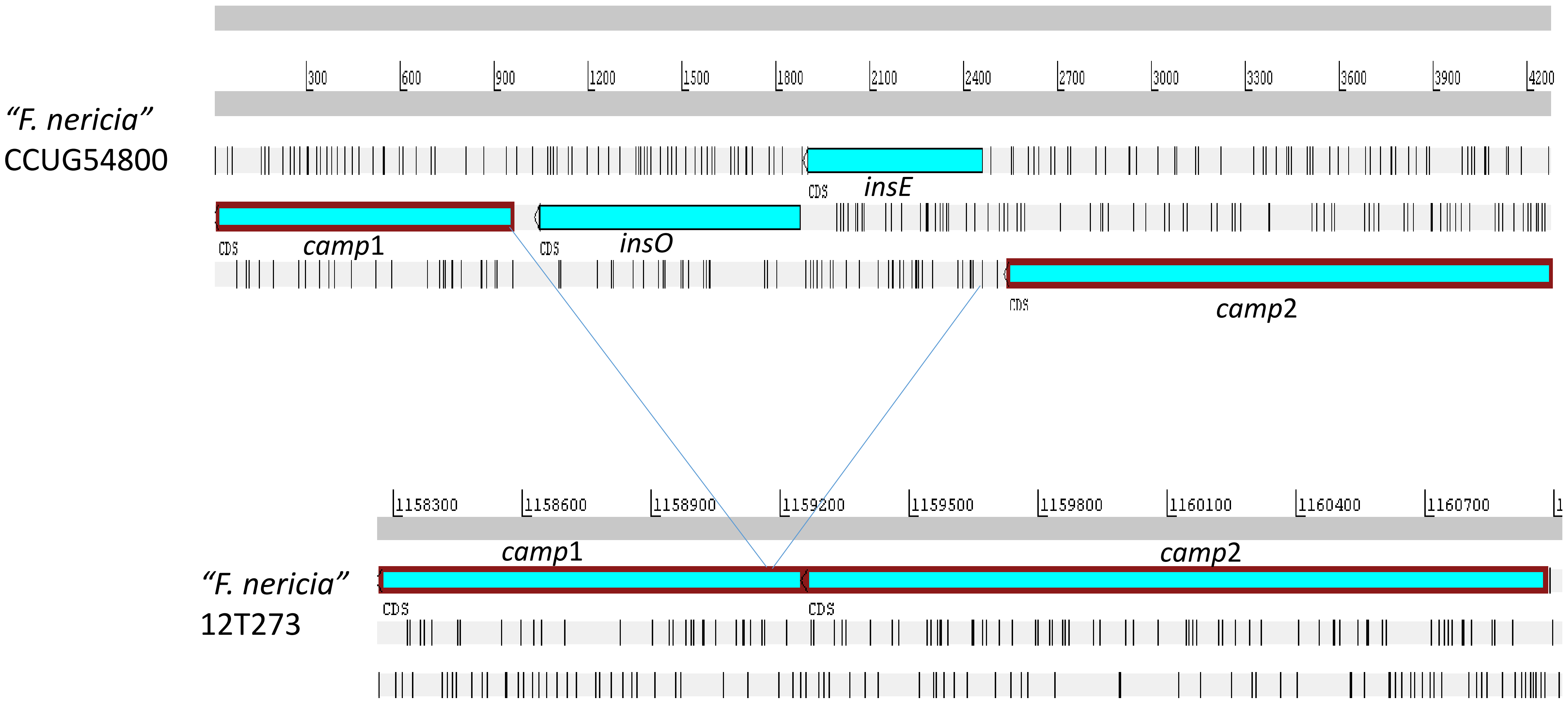

Figure S6: Insertional inactivation of *camp*1 in strain CCUG54800
The genomic regions containing the *camp*1 and the *camp*2 gene in the *"F. nericia"* strains CCUG54800 and 12T273 are shown.
A transposase-encoding mobile element is inserted in the 5' end of *camp*1 in strain CCUG54800.

Table S1: Bidirectional blast of all coding sequences of 12 *Finegoldia* genomes

The program ProteinOrtho (version 4.26) was used with the following parameters: blast=blastp v2.2.24, E-value=1e-10, alg.-conn.=0.1, coverage=0.5, percent_identity=25, adaptive_similarity=0.95, inc_pairs=1, inc_singles=1, selfblast=1, unambiguous=0)
The Prokka annotation is shown. Highlighted in grey are the CDS with orthologs in all 12 genomes.

#species	proteins	alg.-conn.	Annotation	ATCC29328	ATCC53516	07T609	08T492	09T408	09T494	12T272	12T273	12T306	CCUG54800	T151023	T160124
#12	21125	-		1842 CDS	1817 CDS	1698 CDS	1796 CDS	1677 CDS	1827 CDS	1773 CDS	1840 CDS	1712 CDS	1906 CDS	1570 CDS	1667 CDS
12	12	1	Chromosomal replication initiator protein Dna	ATCC29328_00010	ATCC53516_11280	07T609_16840	08T492_17540	09T408_16640	09T494_01290	12T272_17700	12T273_00730	12T306_00150	CCUG54800_18780	T151023_00340	T160124_16570
12	12	1	DNA polymerase III subunit beta	ATCC29328_00020	ATCC53516_11270	07T609_16850	08T492_17550	09T408_16650	09T494_01300	12T272_17710	12T273_00740	12T306_00160	CCUG54800_18790	T151023_00350	T160124_16580
12	12	1	ribosome-associated protein	ATCC29328_00030	ATCC53516_11260	07T609_16860	08T492_17560	09T408_16660	09T494_01310	12T272_17720	12T273_00750	12T306_00170	CCUG54800_18800	T151023_00360	T160124_16590
12	12	1	DNA replication and repair protein RecF	ATCC29328_00040	ATCC53516_11250	07T609_16870	08T492_17570	09T408_16670	09T494_01320	12T272_17730	12T273_00760	12T306_00180	CCUG54800_18810	T151023_00370	T160124_16600
12	12	1	hypothetical protein	ATCC29328_00050	ATCC53516_11240	07T609_16880	08T492_17580	09T408_16680	09T494_01330	12T272_17740	12T273_00770	12T306_00190	CCUG54800_18820	T151023_00380	T160124_16610
12	12	1	DNA gyrase subunit B	ATCC29328_00060	ATCC53516_11230	07T609_16890	08T492_17590	09T408_16690	09T494_01340	12T272_17750	12T273_00780	12T306_00200	CCUG54800_18830	T151023_00390	T160124_16620
12	12	1	DNA gyrase subunit A	ATCC29328_00070	ATCC53516_11220	07T609_16900	08T492_17600	09T408_16700	09T494_01350	12T272_17760	12T273_00790	12T306_00210	CCUG54800_18840	T151023_00400	T160124_16630
12	12	1	Putative anti-sigma factor antagonist	ATCC29328_00080	ATCC53516_11210	07T609_16910	08T492_17610	09T408_16710	09T494_01360	12T272_17770	12T273_00800	12T306_00220	CCUG54800_18850	T151023_00410	T160124_16640
12	12	1	serine-protein kinase RsbW	ATCC29328_00090	ATCC53516_11200	07T609_16920	08T492_17620	09T408_16720	09T494_01370	12T272_17780	12T273_00810	12T306_00230	CCUG54800_18860	T151023_00420	T160124_16650
12	24	0.167	RNA polymerase sigma factor SigF	ATCC29328_00100,ATCC2932	ATCC53516_16000,ATCC5351	07T609_08200,07T609_	08T492_08050,08T492_17	09T408_08280,09T408_	09T494_09240,09T494_	12T272_07000,12T272_	12T273_11890,12T273_	12T306_10120,12T306_	CCUG54800_18870,CCUG548	T151023_08580,T151023_	T160124_08490,T160124_16660
12	12	1	Glutamine transport ATP-binding protein GlnQ	ATCC29328_00110	ATCC53516_11180	07T609_16940	08T492_17640	09T408_16740	09T494_01390	12T272_17800	12T273_00830	12T306_00250	CCUG54800_18880	T151023_00440	T160124_16670
12	12	1	hypothetical protein	ATCC29328_00120	ATCC53516_11170	07T609_16950	08T492_17650	09T408_16750	09T494_01400	12T272_17810	12T273_00840	12T306_00260	CCUG54800_18890	T151023_00450	T160124_16680
12	24	0.167	Fumarate hydratase class II	ATCC29328_00130,ATCC2932	ATCC53516_05620,ATCC5351	07T609_05000,07T609_	08T492_04690,08T492_17	09T408_05110,09T408_	09T494_05880,09T494_	12T272_03680,12T272_	12T273_08570,12T273_	12T306_06780,12T306_	CCUG54800_18900,CCUG548	T151023_05340,T151023_	T160124_05120,T160124_16690
12	12	1	hypothetical protein	ATCC29328_00140	ATCC53516_11150	07T609_16970	08T492_17670	09T408_16770	09T494_01420	12T272_17830	12T273_00860	12T306_00280	CCUG54800_18910	T151023_00470	T160124_16700
6	7	0.241	Acetyltransferase (GNAT) family protein	ATCC29328_00150	*	*	*	*	09T494_01430	12T272_17840,12T272_	12T273_00870	*	CCUG54800_18920	*	T160124_16710
12	12	1	hypothetical protein	ATCC29328_00160	ATCC53516_11140	07T609_16980	08T492_17680	09T408_16780	09T494_01440	12T272_17850	12T273_00880	12T306_00290	CCUG54800_18930	T151023_00480	T160124_16720
12	12	1	hypothetical protein	ATCC29328_00170	ATCC53516_11130	07T609_16990	08T492_17690	09T408_16790	09T494_01450	12T272_17860	12T273_00890	12T306_00300	CCUG54800_18940	T151023_00490	T160124_16730
7	7	1	YcfA-like protein	ATCC29328_00180	ATCC53516_11120	07T609_17000	08T492_17720	09T408_16800	*	12T272_17890	*	*	CCUG54800_18950	*	*
7	7	1	hypothetical protein	ATCC29328_00190	ATCC53516_11110	07T609_17010	08T492_17730	09T408_16810	*	12T272_17900	*	*	CCUG54800_18960	*	*
11	11	1	hypothetical protein	ATCC29328_00200	ATCC53516_11100	07T609_17020	08T492_17740	09T408_16820	09T494_01480	12T272_17910	12T273_00920	*	CCUG54800_18970	T151023_00500	T160124_16760
11	11	1	hypothetical protein	ATCC29328_00210	ATCC53516_11090	07T609_17030	08T492_17750	09T408_16830	09T494_01490	12T272_17920	12T273_00930	*	CCUG54800_18980	T151023_00510	T160124_16770
12	12	1	hypothetical protein	ATCC29328_00220	ATCC53516_11080	07T609_17040	08T492_17760	09T408_16840	09T494_01500	12T272_17930	12T273_00940	12T306_00310	CCUG54800_18990	T151023_00520	T160124_16780
12	12	1	hypothetical protein	ATCC29328_00230	ATCC53516_11070	07T609_17050	08T492_17770	09T408_16850	09T494_01510	12T272_17940	12T273_00950	12T306_00320	CCUG54800_19000	T151023_00530	T160124_16790
12	12	1	YvrJ protein family protein	ATCC29328_00240	ATCC53516_11050	07T609_17060	08T492_17780	09T408_16860	09T494_01520	12T272_17950	12T273_00960	12T306_00330	CCUG54800_19010	T151023_00540	T160124_16800
12	19	0.123	High-affinity zinc uptake system binding-protei	ATCC29328_00250,ATCC2932	ATCC53516_17010,ATCC5351	07T609_09050,07T609_	08T492_08920,08T492_17	09T408_09240,09T408_	09T494_10200,09T494_	12T272_07950	12T273_12830	12T306_00340,12T306_	CCUG54800_10270	T151023_09460	T160124_09440
7	7	1	High-affinity zinc uptake system ATP-binding p	ATCC29328_00260	ATCC53516_11010	07T609_17080	08T492_17800	09T408_16880	09T494_01540	*	*	12T306_00350	*	*	*
7	7	1	High-affinity zinc uptake system membrane pr	ATCC29328_00270	ATCC53516_11000	07T609_17090	08T492_17810	09T408_16890	09T494_01550	*	*	12T306_00360	*	*	*
7	7	1	hypothetical protein	ATCC29328_00280	ATCC53516_10990	07T609_17100	08T492_17820	09T408_16900	09T494_01560	*	*	12T306_00370	*	*	*
4	4	1	hypothetical protein	ATCC29328_00290	*	07T609_17110	*	*	*	*	12T273_00970	*	CCUG54800_00010	*	*
12	12	1	Transglutaminase-like superfamily protein	ATCC29328_00300	ATCC53516_10980	07T609_17120	08T492_17860	09T408_16920	09T494_01570	12T272_17960	12T273_00980	12T306_00390	CCUG54800_00020	T151023_00550	T160124_16810
12	12	1	molybdopterin biosynthesis-like protein MoeZ	ATCC29328_00310	ATCC53516_10970	07T609_17130	08T492_17870	09T408_16930	09T494_01580	12T272_17970	12T273_00990	12T306_00400	CCUG54800_00030	T151023_00560	T160124_16820
12	12	1	K(+)/H(+) antiporter NhaP2	ATCC29328_00320	ATCC53516_10960	07T609_17140	08T492_17880	09T408_16940	09T494_01600	12T272_17980	12T273_01000	12T306_00410	CCUG54800_00040	T151023_00570	T160124_16830
12	17	0.148	Ferrous-iron efflux pump FieF	ATCC29328_00330	ATCC53516_18210,ATCC5351	07T609_17150	08T492_17890	09T408_16950	09T494_11290,09T494_	12T272_17990	12T273_14220,12T273_	12T306_00420	CCUG54800_00050,CCUG548	T151023_00580	T160124_10240,T160124_16840
12	12	1	PAP2 superfamily protein	ATCC29328_00340	ATCC53516_10940	07T609_17160	08T492_17900	09T408_16960	09T494_01620	12T272_18000	12T273_01020	12T306_00430	CCUG54800_00060	T151023_00590	T160124_16850
8	8	1	hypothetical protein	ATCC29328_00350	ATCC53516_07850	07T609_03000	08T492_02680	09T408_03130	*	*	12T273_06530	12T306_00440	*	T151023_03310	*
12	12	1	Thiamine precursor transporter HmpT	ATCC29328_00360	ATCC53516_10870	07T609_17170	08T492_17910	09T408_16970	09T494_01640	12T272_18020	12T273_01040	12T306_00460	CCUG54800_00080	T151023_00610	T160124_16870
9	9	1	PII-type proteinase precursor	ATCC29328_00370	ATCC53516_10860	*	08T492_17920	*	09T494_01650	12T272_18030	12T273_01050	12T306_00480	*	T151023_00620	T160124_16880
1	1	1	N-acetylmuramoyl-L-alanine amidase LytC pre	ATCC29328_00380	*	*	*	*	*	*	*	*	*	*	*
12	12	1	Cna protein B-type domain protein	ATCC29328_00390	ATCC53516_10850	07T609_00010	08T492_17930	09T408_00010	09T494_01660	12T272_18040	12T273_01060	12T306_00500	CCUG54800_00100	T151023_00630	T160124_16890
12	12	1	Putative small multi-drug export protein	ATCC29328_00400	ATCC53516_10820	07T609_00020	08T492_17950	09T408_00020	09T494_01670	12T272_18070	12T273_01080	12T306_00510	CCUG54800_00120	T151023_00650	T160124_16910
12	12	1	Phosphorylated carbohydrates phosphatase	ATCC29328_00410	ATCC53516_10810	07T609_00030	08T492_17960	09T408_00030	09T494_01680	12T272_18080	12T273_01090	12T306_00520	CCUG54800_00130	T151023_00660	T160124_16920
12	12	1	Aminopeptidase 2	ATCC29328_00420	ATCC53516_10800	07T609_00040	08T492_17970	09T408_00040	09T494_01690	12T272_18090	12T273_01100	12T306_00530	CCUG54800_00140	T151023_00670	T160124_16930
12	24	0.147	Oligoendopeptidase F, plasmid	ATCC29328_00430,ATCC2932	ATCC53516_03170,ATCC5351	07T609_11770,07T609_	08T492_11680,08T492_17	09T408_11260,09T408_	09T494_12410,09T494_	12T272_09940,12T272_	12T273_15370,12T273_	12T306_13370,12T306_	CCUG54800_00150,CCUG548	T151023_11260,T151023_	T160124_16940,T160124_11400
12	12	1	Glutamine transport ATP-binding protein GlnQ	ATCC29328_00440	ATCC53516_10780	07T609_00060	08T492_17990	09T408_00060	09T494_01710	12T272_18110	12T273_01120	12T306_00550	CCUG54800_00160	T151023_00690	T160124_16950
12	12	1	hypothetical protein	ATCC29328_00450	ATCC53516_10770	07T609_00070	08T492_18000	09T408_00070	09T494_01720	12T272_18120	12T273_01130	12T306_00560	CCUG54800_00170	T151023_00700	T160124_16960
12	12	1	hypothetical protein	ATCC29328_00460	ATCC53516_10760	07T609_00080	08T492_18010	09T408_00080	09T494_01730	12T272_18130	12T273_01140	12T306_00570	CCUG54800_00180	T151023_00710	T160124_16970
9	9	1	enterobactin exporter EntS	ATCC29328_00470	ATCC53516_10750	*	*	*	09T494_01740	12T272_18140	12T273_01150	12T306_00580	CCUG54800_00190	T151023_00720	T160124_16980
12	12	1	Putative superoxide reductase	ATCC29328_00480	ATCC53516_10710	07T609_00090	08T492_18020	09T408_00100	09T494_01750	12T272_18150	12T273_01160	12T306_00650	CCUG54800_00260	T151023_00730	T160124_16990
2	2	1	Fic/DOC family protein	ATCC29328_00490	*	*	*	*	*	*	*	*	CCUG54800_16290	*	*
5	5	0.2	Listeria-Bacteroides repeat domain (List_Bact_	ATCC29328_00500	*	07T609_00100	08T492_18030	09T408_00110	*	*	*	*	*	T151023_00760	*
1	1	1	N-acetylmuramoyl-L-alanine amidase LytC pre	ATCC29328_00510	*	*	*	*	*	*	*	*	*	*	*
12	12	1	Radical SAM superfamily protein	ATCC29328_00520	ATCC53516_10700	07T609_00110	08T492_18040	09T408_00120	09T494_01760	12T272_18180	12T273_01220	12T306_00670	CCUG54800_00290	T151023_00770	T160124_00030
12	12	1	Bifunctional ligase/repressor BirA	ATCC29328_00530	ATCC53516_10690	07T609_00120	08T492_18050	09T408_00130	09T494_01770	12T272_18190	12T273_01230	12T306_00680	CCUG54800_00300	T151023_00780	T160124_00040
7	7	1	hypothetical protein	ATCC29328_00540	*	07T609_00130	08T492_18060	*	*	*	12T273_01240	12T306_00700	CCUG54800_00320	*	T160124_00050
12	22	0.119	hypothetical protein	ATCC29328_00550,ATCC2932	ATCC53516_11300,ATCC5351	07T609_16820,07T609_	08T492_17520,08T492_18	09T408_16620,09T408_	09T494_01780	12T272_17680,12T272_	12T273_00710,12T273_	12T306_00130,12T306_	CCUG54800_00330,CCUG548	T151023_00790	T160124_16550,T160124_00060
12	12	1	Cadmium, zinc and cobalt-transporting ATPase	ATCC29328_00560	ATCC53516_10670	07T609_00150	08T492_18080	09T408_00150	09T494_01790	12T272_00020	12T273_01260	12T306_00720	CCUG54800_00340	T151023_00800	T160124_00070
12	24	0.167	Cadmium, zinc and cobalt-transporting ATPase	ATCC29328_00570,ATCC2932	ATCC53516_07010,ATCC5351	07T609_03760,07T609_	08T492_03460,08T492_18	09T408_03910,09T408_	09T494_04600,09T494_	12T272_02400,12T272_	12T273_07340,12T273_	12T306_05510,12T306_	CCUG54800_00350,CCUG548	T151023_04100,T151023_	T160124_03800,T160124_00080
12	12	1	hypothetical protein	ATCC29328_00580	ATCC53516_10650	07T609_00170	08T492_18100	09T408_00170	09T494_01810	12T272_00040	12T273_01280	12T306_00740	CCUG54800_00360	T151023_00820	T160124_00090
12	12	1	hypothetical protein	ATCC29328_00590	ATCC53516_10610	07T609_00200	08T492_18130	09T408_00200	09T494_01820	12T272_00070	12T273_01310	12T306_00770	CCUG54800_00390	T151023_00860	T160124_00120
3	3	1	CRISPR-associated endonuclease Cas9 2	ATCC29328_00600	*	07T609_00210	*	09T408_00210	*	*	*	*	*	*	*
3	3	1	CRISPR-associated endonuclease Cas1	ATCC29328_00610	*	07T609_00220	*	09T408_00220	*	*	*	*	*	*	*
3	3	1	CRISPR-associated endoribonuclease Cas2	ATCC29328_00620	*	07T609_00230	*	09T408_00230	*	*	*	*	*	*	*
3	3	1	hypothetical protein	ATCC29328_00630	*	07T609_00240	*	09T408_00240	*	*	*	*	*	*	*
8	8	1	hypothetical protein	ATCC29328_00640	ATCC53516_10600	07T609_00250	08T492_13750	09T408_00250	*	*	*	12T306_00790	CCUG54800_00430	T151023_00890	*
2	2	1	hypothetical protein	ATCC29328_00650	*	07T609_00260	*	*	*	*	*	*	*	*	*
12	15	0.145	2-iminoacetate synthase	ATCC29328_00660	ATCC53516_10590	07T609_00270	08T492_18140	09T408_00260	09T494_13500,09T494_	12T272_11000,12T272_	12T273_01320	12T306_00800	CCUG54800_00440	T151023_00900,T151023_	T160124_00140
12	12	1	Thiazole synthase	ATCC29328_00670	ATCC53516_10580	07T609_00280	08T492_18150	09T408_00270	09T494_01850	12T272_00100	12T273_01330	12T306_00810	CCUG54800_00450	T151023_00910	T160124_00150
12	12	1	putative adenylyltransferase/sulfurtransferase	ATCC29328_00680	ATCC53516_10570	07T609_00290	08T492_18160	09T408_00280	09T494_01860	12T272_00110	12T273_01340	12T306_00820	CCUG54800_00460	T151023_00920	T160124_00160
12	12	1	sulfur carrier protein ThiS	ATCC29328_00690	ATCC53516_10560	07T609_00300	08T492_18170	09T408_00290	09T494_01870	12T272_00120	12T273_01350	12T306_00830	CCUG54800_00470	T151023_00930	T160124_00170
12	12	1	Glutathione S-transferase GST-4.5	ATCC29328_00700	ATCC53516_10550	07T609_00320	08T492_18180	09T408_00300	09T494_01880	12T272_00130	12T273_01360	12T306_00840	CCUG54800_00480	T151023_00940	T160124_00180
12	12	1	Competence protein ComM	ATCC29328_00710	ATCC53516_10530	07T609_00330	08T492_18190	09T408_00310	09T494_01900	12T272_00150	12T273_01380	12T306_00860	CCUG54800_00490	T151023_00960	T160124_00200
9	9	1	hypothetical protein	ATCC29328_00720	ATCC53516_10520	07T609_00340	08T492_18200	09T408_00320	09T494_02040	12T272_00160	*	12T306_00870	CCUG54800_00500	*	*
12	12	1	L-carnitine dehydrogenase	ATCC29328_00730	ATCC53516_10470	07T609_00350	08T492_18210	09T408_00360	09T494_02080	12T272_00210	12T273_01450	12T306_00910	CCUG54800_00540	T151023_01030	T160124_00260
9	9	1	hypothetical protein	ATCC29328_00740	ATCC53516_10460	07T609_00360	08T492_18220	09T408_00370	09T494_02090	12T272_00230	*	12T306_00920	CCUG54800_00550	*	*
9	9	1	hypothetical protein	ATCC29328_00750	ATCC53516_10450	07T609_00370	08T492_18230	09T408_00380	09T494_02100	12T272_00240	*	12T306_00930	CCUG54800_00560	*	*
2	2	1	N-6 DNA Methylase	ATCC29328_00760	*	*	*	*	*	*	*	*	*	T151023_01040	*
2	2	1	hypothetical protein	ATCC29328_00770	*	*	*	*	*	*	*	*	*	T151023_01050	*
7	8	0.499	putative type I restriction enzymeP M protein	ATCC29328_00780	ATCC53516_01870	07T609_00400	08T492_16940	*	*	*	12T273_03110	*	*	T151023_15500,T151023_	T160124_15940
5	5	1	hypothetical protein	ATCC29328_00790	*	07T609_00410	*	*	09T494_15860	*	*	*	CCUG54800_16840	T151023_01070	*
3	3	1	Putative type-1 restriction enzyme specificity p	ATCC29328_00800	*	07T609_00420	*	*	*	*	*	*	*	T151023_01080	*
4	4	0.5	Type-1 restriction enzyme R protein	ATCC29328_00810	ATCC53516_01850	07T609_00430	*	*	*	*	*	*	*	T151023_01090	*
3	3	1	hypothetical protein	ATCC29328_00820	*	07T609_00440	*	*	*	*	*	*	*	T151023_01100	*
4	8	1	Elongation factor Tu-B	ATCC29328_00830,ATCC2932	ATCC53516_10440,ATCC5351	*	*	*	09T494_05560,09T494_	*	*	*	*	*	T160124_04820,T160124_00310
12	12	1	anaerobic ribonucleoside triphosphate reducta	ATCC29328_00840	ATCC53516_10430	07T609_00460	08T492_00020	09T408_00430	09T494_02150	12T272_00290	12T273_01470	12T306_00950	CCUG54800_00580	T151023_01120	T160124_00320
3	3	1	N-acetylmuramoyl-L-alanine amidase LytC pre	ATCC29328_00850	*	*	*	*	*	*	*	*	CCUG54800_00590	*	T160124_00330
11	19	0.2	N-acetylmuramoyl-L-alanine amidase LytC pre	ATCC29328_00860,ATCC2932	ATCC53516_10390,ATCC5351	07T609_00500,07T609_	08T492_00050,08T492_00	*	09T494_02180,09T494_	12T272_00310	12T273_01500,12T273_	12T306_00980,12T306_	CCUG54800_00610	T151023_01140	T160124_00360,T160124_00340
12	12	1	hypothetical protein	ATCC29328_00870	ATCC53516_10400	07T609_00490	08T492_00040	09T408_00450	09T494_02170	12T272_00300	12T273_01490	12T306_00970	CCUG54800_00600	T151023_01130	T160124_00350
12	12	1	hypothetical protein	ATCC29328_00880	ATCC53516_10380	07T609_00510	08T492_00060	09T408_00460	09T494_02190	12T272_00320	12T273_01610	12T306_00990	CCUG54800_00620	T151023_01150	T160124_00370
12	12	1	hypothetical protein	ATCC29328_00890	ATCC53516_10370	07T609_00520	08T492_00070	09T408_00470	09T494_02200	12T272_00330	12T273_01620	12T306_01000	CCUG54800_00630	T151023_01160	T160124_00380
12	12	1	Bifunctional oligoribonuclease and PAP phosph	ATCC29328_00900	ATCC53516_10360	07T609_00530	08T492_00080	09T408_00480	09T494_02210	12T272_00340	12T273_01630	12T306_01010	CCUG54800_00640	T151023_01170	T160124_00390
12	12	1	50S ribosomal protein L9	ATCC29328_00910	ATCC53516_10350	07T609_00540	08T492_00090	09T408_00490	09T494_02220	12T272_00350	12T273_01640	12T306_01020	CCUG54800_00650	T151023_01180	T160124_00400
12	12	1	Replicative DNA helicase	ATCC29328_00920	ATCC53516_10340	07T609_00550	08T492_00100	09T408_00500	09T494_02230	12T272_00360	12T273_01650	12T306_01030	CCUG54800_00660	T151023_01190	T160124_00410
12	12	1	Acetyltransferase (GNAT) family protein	ATCC29328_00930	ATCC53516_10330	07T609_00560	08T492_00110	09T408_00510	09T494_02240	12T272_00370	12T273_01660	12T306_01040	CCUG54800_00670	T151023_01200	T160124_00420
12	12	1	Replication initiation and membrane attachme	ATCC29328_00940	ATCC53516_10320	07T609_00570	08T492_00120	09T408_00520	09T494_02250	12T272_00380	12T273_01670	12T306_01050	CCUG54800_00680	T151023_01210	T160124_00430
12	12	1	Primosomal protein DnaI	ATCC29328_00950	ATCC53516_10310	07T609_00580	08T492_00130	09T408_00530	09T494_02260	12T272_00390	12T273_01680	12T306_01060	CCUG54800_00690	T151023_01220	T160124_00440
12	12	1	Bifunctional NAD(P)H-hydrate repair enzyme N	ATCC29328_00960	ATCC53516_10300	07T609_00590	08T492_00140	09T408_00540	09T494_02270	12T272_00400	12T273_01690	12T306_01070	CCUG54800_00700	T151023_01230	T160124_00450
12	12	1	hypothetical protein	ATCC29328_00970	ATCC53516_10290	07T609_00600	08T492_00150	09T408_00550	09T494_02280	12T272_00410	12T273_01700	12T306_01080	CCUG54800_00710	T151023_01240	T160124_00460
12	12	1	hypothetical protein	ATCC29328_00980	ATCC53516_10280	07T609_00610	08T492_00160	09T408_00560	09T494_02290	12T272_00420	12T273_01710	12T306_01090	CCUG54800_00720	T151023_01250	T160124_00470
12	23	0.163	hypothetical protein	ATCC29328_00990,ATCC2932	ATCC53516_04200,ATCC5351	07T609_12760,07T609_	08T492_12830,08T492_00	09T408_12210,09T408_	09T494_13420,09T494_	12T272_10920,12T272_	12T273_01720	12T306_14380,12T306_	CCUG54800_00730,CCUG548	T151023_12270,T151023_	T160124_12570,T160124_00480
8	8	1	hypothetical protein	ATCC29328_01000	*	07T609_00630	08T492_00180	09T408_00580	*	12T272_00440	12T273_01730	*	CCUG54800_00740	*	T160124_00490
1	1	1	hypothetical protein	ATCC29328_01010	*	*	*	*	*	*	*	*	*	*	*
6	6	1	D-alanyl-D-alanine carboxypeptidase precurso	ATCC29328_01020	ATCC53516_10240	07T609_00650	08T492_00200	09T408_00600	09T494_02330	*	*	*	*	*	*
12	12	1	Serine--tRNA ligase	ATCC29328_01030	ATCC53516_10230	07T609_00660	08T492_00210	09T408_00610	09T494_02340	12T272_00460	12T273_01750	12T306_01130	CCUG54800_00760	T151023_01290	T160124_00510
12	12	1	Putative teichuronic acid biosynthesis glycosylt	ATCC29328_01040	ATCC53516_10220	07T609_00670	08T492_00220	09T408_00620	09T494_02350	12T272_00470	12T273_01760	12T306_01140	CCUG54800_00770	T151023_01300	T160124_00520
12	24	0.167	D-alanyl-D-alanine carboxypeptidase DacF pre	ATCC29328_01050,ATCC2932	ATCC53516_09490,ATCC5351	07T609_15250,07T609_	08T492_16270,08T492_00	09T408_15120,09T408_	09T494_17930,09T494_	12T272_15460,12T272_	12T273_04290,12T273_	12T306_02900,12T306_	CCUG54800_00780,CCUG548	T151023_14790,T151023_	T160124_15170,T160124_00530
4	4	1	hypothetical protein	ATCC29328_01070	*	07T609_00700	08T492_00250	*	*	*	*	*	CCUG54800_01440	*	*
12	12	1	hypothetical protein	ATCC29328_01080	ATCC53516_10190	07T609_00710	08T492_00260	09T408_00650	09T494_02380	12T272_00510	12T273_01790	12T306_01170	CCUG54800_01450	T151023_01330	T160124_00550
12	12	1	Helix-hairpin-helix motif protein	ATCC29328_01090	ATCC53516_10180	07T609_00720	08T492_00270	09T408_00660	09T494_02390	12T272_00520	12T273_01800	12T306_01180	CCUG54800_01460	T151023_01340	T160124_00560
12	24	0.167	Branched-chain amino acid transport system 2	ATCC29328_01100,ATCC2932	ATCC53516_08810,ATCC5351	07T609_02070,07T609_	08T492_01800,08T492_00	09T408_02110,09T408_	09T494_15360,09T494_	12T272_12360,12T272_	12T273_05050,12T273_	12T306_03630,12T306_	CCUG54800_01470,CCUG548	T151023_13600,T151023_	T160124_01620,T160124_00570
12	12	1	hypothetical protein	ATCC29328_01110	ATCC53516_10160	07T609_00740	08T492_00290	09T408_00680	09T494_02410	12T272_00540	12T273_01820	12T306_01200	CCUG54800_01480	T151023_01360	T160124_00580
12	12	1	Isochorismatase family protein	ATCC29328_01120	ATCC53516_10150	07T609_00780	08T492_00300	09T408_00690	09T494_02460	12T272_00580	12T273_01860	12T306_01270	CCUG54800_01520	T151023_01400	T160124_00590
12	12	1	hypothetical protein	ATCC29328_01130	ATCC53516_10140	07T609_00790	08T492_00310	09T408_00700	09T494_02470	12T272_00590	12T273_01870	12T306_01280	CCUG54800_01530	T151023_01410	T160124_00600
12	24	0.147	TraX protein	ATCC29328_01140,ATCC2932	ATCC53516_11500,ATCC5351	07T609_16600,07T609_	08T492_17340,08T492_00	09T408_16420,09T408_	09T494_01100,09T494_	12T272_16790,12T272_	12T273_00520,12T273_	12T306_17410,12T306_	CCUG54800_01540,CCUG548	T151023_00140,T151023_	T160124_16370,T160124_00610
12	12	1	Arsenical resistance operon trans-acting repre	ATCC29328_01150	ATCC53516_10120	07T609_00810	08T492_00330	09T408_00720	09T494_02490	12T272_00610	12T273_01890	12T306_01300	CCUG54800_01550	T151023_01430	T160124_00620
12	13	0.141	Putative glutamine amidotransferase	ATCC29328_01160,ATCC2932	ATCC53516_10110	07T609_00820	08T492_00340	09T408_00730	09T494_02500	12T272_00620	12T273_01900	12T306_01310	CCUG54800_01560	T151023_01440	T160124_00630
12	12	1	putative DMT superfamily transporter inner m	ATCC29328_01180	ATCC53516_10090	07T609_00840	08T492_00360	09T408_00750	09T494_02520	12T272_00760	12T273_01920	12T306_01330	CCUG54800_01580	T151023_01460	T160124_00650
12	12	1	Peptide methionine sulfoxide reductase msrA/	ATCC29328_01190	ATCC53516_10080	07T609_00850	08T492_00370	09T408_00760	09T494_02530	12T272_00770	12T273_01930	12T306_01340	CCUG54800_01590	T151023_01470	T160124_00660
12	12	1	pyrroloquinoline quinone biosynthesis protein	ATCC29328_01200	ATCC53516_10070	07T609_00860	08T492_00380	09T408_00770	09T494_02540	12T272_00780	12T273_01940	12T306_01350	CCUG54800_01600	T151023_01480	T160124_00670
12	12	1	DNA-binding transcriptional repressor AcrR	ATCC29328_01210	ATCC53516_10060	07T609_00870	08T492_00390	09T408_00780	09T494_02550	12T272_00790	12T273_01950	12T306_01360	CCUG54800_01610	T151023_01490	T160124_00680
12	12	1	Carboxymuconolactone decarboxylase family	ATCC29328_01220	ATCC53516_10050	07T609_00880	08T492_00400	09T408_00790	09T494_02560	12T272_00800	12T273_01960	12T306_01370	CCUG54800_01630	T151023_01500	T160124_00690
9	9	1	Phosphate-binding protein PstS precursor	ATCC29328_01230	ATCC53516_10040	07T609_00890	08T492_00410	09T408_00800	09T494_02580	12T272_00810	*	12T306_01380	*	T151023_01510	*
12	21	0.158	Phosphate transport system permease protein	ATCC29328_01240,ATCC2932	ATCC53516_13580,ATCC5351	07T609_05800,07T609_	08T492_05650,08T492_00	09T408_05910,09T408_	09T494_06830,09T494_	12T272_04600,12T272_	12T273_09470	12T306_07690,12T306_	CCUG54800_06930	T151023_01520,T151023_	T160124_06090
9	9	1	Phosphate transport system permease protein	ATCC29328_01250	ATCC53516_10020	07T609_00910	08T492_00430	09T408_00820	09T494_02600	12T272_00830	*	12T306_01400	*	T151023_01530	*
12	21	0.158	Phosphate import ATP-binding protein PstB	ATCC29328_01260,ATCC2932	ATCC53516_13560,ATCC5351	07T609_05780,07T609_	08T492_05630,08T492_00	09T408_05890,09T408_	09T494_06810,09T494_	12T272_04580,12T272_	12T273_09450	12T306_07670,12T306_	CCUG54800_06910	T151023_01540,T151023_	T160124_06070
11	11	1	Transcriptional regulatory protein SrrA	ATCC29328_01270	*	07T609_00930	08T492_00450	09T408_00840	09T494_02620	12T272_00850	12T273_01990	12T306_01420	CCUG54800_01650	T151023_01550	T160124_00720
12	22	0.149	Sensor kinase CusS	ATCC29328_01280,ATCC2932	ATCC53516_16860	07T609_00940	08T492_08810,08T492_00	09T408_09110,09T408_	09T494_10060,09T494_	12T272_07820,12T272_	12T273_12720,12T273_	12T306_10870,12T306_	CCUG54800_01660,CCUG548	T151023_09350,T151023_	T160124_09330,T160124_00730
11	11	1	hypothetical protein	ATCC29328_01290	*	07T609_00950	08T492_00480	09T408_00860	09T494_02650	12T272_00880	12T273_02010	12T306_01440	CCUG54800_01670	T151023_01570	T160124_00740
11	12	0.152	Lipoprotein-releasing system ATP-binding prot	ATCC29328_01300	*	07T609_00960	08T492_00490	09T408_00870	09T494_02660,09T494_	12T272_00890	12T273_02020	12T306_01450	CCUG54800_01680	T151023_01580	T160124_00750
12	12	1	Glycosyl transferases group 1	ATCC29328_01310	ATCC53516_09950	07T609_01000	08T492_00530	09T408_00910	09T494_02680	12T272_00900	12T273_02050	12T306_01460	CCUG54800_01710	T151023_01600	T160124_00780
12	24	0.167	UDP-N-acetyl-D-glucosamine 6-dehydrogenase	ATCC29328_01320,ATCC2932	ATCC53516_13440,ATCC5351	07T609_05690,07T609_	08T492_05540,08T492_00	09T408_05790,09T408_	09T494_06710,09T494_	12T272_04480,12T272_	12T273_09340,12T273_	12T306_07570,12T306_	CCUG54800_01720,CCUG548	T151023_06050,T151023_	T160124_05960,T160124_00790
12	18	0.151	UDP-N-acetylglucosamine 2-epimerase	ATCC29328_01330,ATCC2932	ATCC53516_09930	07T609_04800,07T609_	08T492_04490,08T492_00	09T408_00930	09T494_05680,09T494_	12T272_03480,12T272_	12T273_02070	12T306_01480	CCUG54800_01730	T151023_01620,T151023_	T160124_00800
12	12	1	hypothetical protein	ATCC29328_01340	ATCC53516_09920	07T609_01030	08T492_00560	09T408_00940	09T494_02710	12T272_00930	12T273_02080	12T306_01490	CCUG54800_01740	T151023_01630	T160124_00810
12	12	1	hypothetical protein	ATCC29328_01350	ATCC53516_09910	07T609_01040	08T492_00570	09T408_00950	09T494_02720	12T272_00940	12T273_02090	12T306_01500	CCUG54800_01750	T151023_01640	T160124_00820
12	12	1	hypothetical protein	ATCC29328_01360	ATCC53516_09900	07T609_01050	08T492_00580	09T408_00960	09T494_02730	12T272_00950	12T273_02100	12T306_01510	CCUG54800_01760	T151023_01650	T160124_00830
12	12	1	Aminopeptidase YpdE	ATCC29328_01370	ATCC53516_09880	07T609_01060	08T492_00590	09T408_00980	09T494_02760	12T272_01000	12T273_02130	12T306_01540	CCUG54800_01780	T151023_01680	T160124_00860
7	7	1	Signal transduction histidine-protein kinase Ba	ATCC29328_01380	ATCC53516_09870	*	08T492_00600	09T408_00990	09T494_02770	*	*	*	CCUG54800_01790	*	T160124_00870
7	7	1	Phosphate regulon transcriptional regulatory p	ATCC29328_01390	ATCC53516_09860	*	08T492_00610	09T408_01000	09T494_02780	*	*	*	CCUG54800_01800	*	T160124_00880
7	7	1	ABC-2 family transporter protein	ATCC29328_01400	ATCC53516_09850	*	08T492_00620	09T408_01010	09T494_02790	*	*	*	CCUG54800_01810	*	T160124_00890
7	7	1	Thiamine import ATP-binding protein ThiQ	ATCC29328_01410	ATCC53516_09840	*	08T492_00630	09T408_01020	09T494_02800	*	*	*	CCUG54800_01820	*	T160124_00900
12	12	1	Nucleoside 2-deoxyribosyltransferase	ATCC29328_01420	ATCC53516_09830	07T609_01070	08T492_00640	09T408_01030	09T494_02810	12T272_01010	12T273_02140	12T306_01550	CCUG54800_01830	T151023_01690	T160124_00910
12	12	0.667	hypothetical protein	ATCC29328_01430	ATCC53516_09820	07T609_01080	08T492_00650	09T408_01040	09T494_02820	12T272_01020	12T273_02150	12T306_01560	CCUG54800_01840	T151023_01700	T160124_00930
12	12	1	Adenylosuccinate synthetase	ATCC29328_01440	ATCC53516_09810	07T609_01090	08T492_00660	09T408_01050	09T494_02830	12T272_01030	12T273_02160	12T306_01570	CCUG54800_01850	T151023_01710	T160124_00940
1	1	1	hypothetical protein	ATCC29328_01450	*	*	*	*	*	*	*	*	*	*	*
9	10	0.171	sn-glycerol-3-phosphate import ATP-binding p	ATCC29328_01460,ATCC2932	ATCC53516_16820	07T609_16050	*	09T408_09090	09T494_10040	12T272_07800	12T273_12700	*	CCUG54800_10140	*	T160124_09310
1	1	1	Putative ABC transporter substrate-binding pr	ATCC29328_01470	*	*	*	*	*	*	*	*	*	*	*
9	10	0.178	Trehalose transport system permease protein	ATCC29328_01480,ATCC2932	ATCC53516_16810	07T609_16070	*	09T408_09080	09T494_10030	12T272_07790	12T273_12690	*	CCUG54800_10130	*	T160124_09300
9	10	0.274	Trehalose transport system permease protein	ATCC29328_01490,ATCC2932	ATCC53516_16800	07T609_16060	*	09T408_09070	09T494_10020	12T272_07780	12T273_12680	*	CCUG54800_10120	*	T160124_09290
12	12	1	DNA-binding transcriptional repressor AcrR	ATCC29328_01500	ATCC53516_09800	07T609_01100	08T492_00670	09T408_01060	09T494_02840	12T272_01040	12T273_02170	12T306_01580	CCUG54800_01860	T151023_01720	T160124_00950
12	12	1	Membrane protein YdfJ	ATCC29328_01510	ATCC53516_09790	07T609_01110	08T492_00680	09T408_01070	09T494_02850	12T272_01050	12T273_02180	12T306_01590	CCUG54800_01870	T151023_01730	T160124_00960
12	12	1	Chromosome partition protein Smc	ATCC29328_01520	ATCC53516_09780	07T609_01120	08T492_00690	09T408_01080	09T494_02860	12T272_01060	12T273_02190	12T306_01600	CCUG54800_01880	T151023_01740	T160124_00970
12	12	1	Proline--tRNA ligase	ATCC29328_01530	ATCC53516_09770	07T609_01130	08T492_00700	09T408_01090	09T494_02870	12T272_01070	12T273_02200	12T306_01610	CCUG54800_01890	T151023_01750	T160124_00980
8	8	1	antitoxin HipB	ATCC29328_01540	ATCC53516_09760	07T609_01140	08T492_00710	*	09T494_02880	12T272_01080	12T273_02210	12T306_01620	*	*	*
8	8	1	hypothetical protein	ATCC29328_01550	ATCC53516_09750	07T609_01150	08T492_00720	*	09T494_02890	12T272_01090	12T273_02220	12T306_01630	*	*	*
7	7	1	Fic/DOC family protein	ATCC29328_01560	ATCC53516_12540	07T609_01160	08T492_00730	*	*	*	*	*	CCUG54800_01900	T151023_01760	T160124_00990
12	22	0.16	Sarcosine/dimethylglycine N-methyltransferas	ATCC29328_01570,ATCC2932	ATCC53516_11290,ATCC5351	07T609_16830,07T609_	08T492_17530,08T492_00	09T408_16630,09T408_	09T494_02900	12T272_17690,12T272_	12T273_00720,12T273_	12T306_00140,12T306_	CCUG54800_01910,CCUG548	T151023_01770	T160124_16560,T160124_01000
11	11	1	Glycerol uptake facilitator protein	ATCC29328_01580	ATCC53516_12560	07T609_01180	08T492_00750	09T408_01120	09T494_02910	12T272_01110	12T273_02250	12T306_01650	CCUG54800_01920	T151023_01780	*
12	12	1	ribosomal-protein-L7/L12-serine acetyltransfe	ATCC29328_01590	ATCC53516_12570	07T609_01190	08T492_00760	09T408_01130	09T494_02990	12T272_01180	12T273_02270	12T306_01660	CCUG54800_01930	T151023_01790	T160124_01010
12	12	1	Multidrug resistance protein NorM	ATCC29328_01600	ATCC53516_12590	07T609_01200	08T492_00770	09T408_01140	09T494_03000	12T272_01190	12T273_02280	12T306_01670	CCUG54800_01940	T151023_01800	T160124_01020
11	11	1	acid-resistance membrane protein	ATCC29328_01610	ATCC53516_12600	07T609_01220	08T492_00780	09T408_01150	09T494_03010	12T272_01210	12T273_02290	12T306_01680	CCUG54800_01950	*	T160124_01030
12	12	1	30S ribosomal protein S10	ATCC29328_01620	ATCC53516_12610	07T609_01230	08T492_00790	09T408_01160	09T494_03020	12T272_01220	12T273_02300	12T306_01690	CCUG54800_01960	T151023_01810	T160124_01040
12	12	1	50S ribosomal protein L3	ATCC29328_01630	ATCC53516_12620	07T609_01240	08T492_00800	09T408_01170	09T494_03030	12T272_01230	12T273_02310	12T306_01700	CCUG54800_01970	T151023_01820	T160124_01050
12	12	1	50S ribosomal protein L4	ATCC29328_01640	ATCC53516_12630	07T609_01250	08T492_00810	09T408_01180	09T494_03040	12T272_01240	12T273_02320	12T306_01710	CCUG54800_01980	T151023_01830	T160124_01060
12	12	1	50S ribosomal protein L23	ATCC29328_01650	ATCC53516_12640	07T609_01260	08T492_00820	09T408_01190	09T494_03050	12T272_01250	12T273_02330	12T306_01720	CCUG54800_01990	T151023_01840	T160124_01070
12	12	1	50S ribosomal protein L2	ATCC29328_01660	ATCC53516_12650	07T609_01270	08T492_00830	09T408_01200	09T494_03060	12T272_01260	12T273_02340	12T306_01730	CCUG54800_02000	T151023_01850	T160124_01080
12	12	1	30S ribosomal protein S19	ATCC29328_01670	ATCC53516_12660	07T609_01280	08T492_00840	09T408_01210	09T494_03070	12T272_01270	12T273_02350	12T306_01740	CCUG54800_02010	T151023_01860	T160124_01090
12	12	1	50S ribosomal protein L22	ATCC29328_01680	ATCC53516_12670	07T609_01290	08T492_00850	09T408_01220	09T494_03080	12T272_01280	12T273_02360	12T306_01750	CCUG54800_02020	T151023_01870	T160124_01100
12	12	1	30S ribosomal protein S3	ATCC29328_01690	ATCC53516_12680	07T609_01300	08T492_00860	09T408_01230	09T494_03090	12T272_01290	12T273_02370	12T306_01760	CCUG54800_02030	T151023_01880	T160124_01110
12	12	1	50S ribosomal protein L16	ATCC29328_01700	ATCC53516_12690	07T609_01310	08T492_00870	09T408_01240	09T494_03100	12T272_01300	12T273_02380	12T306_01770	CCUG54800_02040	T151023_01890	T160124_01120
12	12	1	50S ribosomal protein L29	ATCC29328_01710	ATCC53516_12700	07T609_01320	08T492_00880	09T408_01250	09T494_03110	12T272_01310	12T273_02390	12T306_01780	CCUG54800_02050	T151023_01900	T160124_01130
12	12	1	30S ribosomal protein S17	ATCC29328_01720	ATCC53516_12710	07T609_01330	08T492_00890	09T408_01260	09T494_03120	12T272_01320	12T273_02400	12T306_01790	CCUG54800_02060	T151023_01910	T160124_01140
12	12	1	50S ribosomal protein L14	ATCC29328_01730	ATCC53516_12720	07T609_01340	08T492_00900	09T408_01270	09T494_03130	12T272_01330	12T273_02410	12T306_01800	CCUG54800_02070	T151023_01920	T160124_01150
12	12	1	50S ribosomal protein L24	ATCC29328_01740	ATCC53516_12730	07T609_01350	08T492_00910	09T408_01280	09T494_03140	12T272_01340	12T273_02420	12T306_01810	CCUG54800_02080	T151023_01930	T160124_01160
12	12	1	50S ribosomal protein L5	ATCC29328_01750	ATCC53516_12740	07T609_01360	08T492_00920	09T408_01290	09T494_03150	12T272_01350	12T273_02430	12T306_01820	CCUG54800_02090	T151023_01940	T160124_01170
12	12	1	30S ribosomal protein S14	ATCC29328_01760	ATCC53516_12750	07T609_01370	08T492_00930	09T408_01300	09T494_03160	12T272_01360	12T273_02440	12T306_01830	CCUG54800_02100	T151023_01950	T160124_01180
12	12	1	30S ribosomal protein S8	ATCC29328_01770	ATCC53516_12760	07T609_01380	08T492_00940	09T408_01310	09T494_03170	12T272_01370	12T273_02450	12T306_01840	CCUG54800_02110	T151023_01960	T160124_01190
12	12	1	50S ribosomal protein L6	ATCC29328_01780	ATCC53516_12770	07T609_01390	08T492_00950	09T408_01320	09T494_03180	12T272_01380	12T273_02460	12T306_01850	CCUG54800_02120	T151023_01970	T160124_01200
12	12	1	50S ribosomal protein L18	ATCC29328_01790	ATCC53516_12780	07T609_01400	08T492_00960	09T408_01330	09T494_03190	12T272_01390	12T273_02470	12T306_01860	CCUG54800_02130	T151023_01980	T160124_01210
12	12	1	30S ribosomal protein S5	ATCC29328_01800	ATCC53516_12790	07T609_01410	08T492_00970	09T408_01340	09T494_03200	12T272_01400	12T273_02480	12T306_01870	CCUG54800_02140	T151023_01990	T160124_01220
12	12	1	50S ribosomal protein L30	ATCC29328_01810	ATCC53516_12800	07T609_01420	08T492_00980	09T408_01350	09T494_03210	12T272_01410	12T273_02490	12T306_01880	CCUG54800_02150	T151023_02000	T160124_01230
12	12	1	50S ribosomal protein L15	ATCC29328_01820	ATCC53516_12810	07T609_01430	08T492_00990	09T408_01360	09T494_03220	12T272_01420	12T273_02500	12T306_01890	CCUG54800_02160	T151023_02010	T160124_01240
12	12	1	preprotein translocase subunit SecY	ATCC29328_01830	ATCC53516_12820	07T609_01440	08T492_01000	09T408_01370	09T494_03230	12T272_01430	12T273_02510	12T306_01900	CCUG54800_02170	T151023_02020	T160124_01250
12	12	1	Adenylate kinase	ATCC29328_01840	ATCC53516_12830	07T609_01450	08T492_01010	09T408_01380	09T494_03240	12T272_01440	12T273_02520	12T306_01910	CCUG54800_02180	T151023_02030	T160124_01260
12	12	1	50S ribosomal protein L14e	ATCC29328_01850	ATCC53516_12840	07T609_01460	08T492_01020	09T408_01390	09T494_03250	12T272_01450	12T273_02530	12T306_01920	CCUG54800_02190	T151023_02040	T160124_01270
12	12	1	Translation initiation factor IF-1	ATCC29328_01860	ATCC53516_12850	07T609_01470	08T492_01030	09T408_01400	09T494_03260	12T272_01460	12T273_02540	12T306_01930	CCUG54800_02200	T151023_02050	T160124_01280
12	12	1	50S ribosomal protein L36	ATCC29328_01870	ATCC53516_12860	07T609_01480	08T492_01040	09T408_01410	09T494_03270	12T272_01470	12T273_02550	12T306_01940	CCUG54800_02210	T151023_02060	T160124_01290
12	12	1	30S ribosomal protein S13	ATCC29328_01880	ATCC53516_12870	07T609_01490	08T492_01050	09T408_01420	09T494_03280	12T272_01480	12T273_02560	12T306_01950	CCUG54800_02220	T151023_02070	T160124_01300
12	12	1	30S ribosomal protein S11	ATCC29328_01890	ATCC53516_12880	07T609_01500	08T492_01060	09T408_01430	09T494_03290	12T272_01490	12T273_02570	12T306_01960	CCUG54800_02230	T151023_02080	T160124_01310
12	12	1	DNA-directed RNA polymerase subunit alpha	ATCC29328_01900	ATCC53516_12890	07T609_01510	08T492_01070	09T408_01440	09T494_03300	12T272_01500	12T273_02580	12T306_01970	CCUG54800_02240	T151023_02090	T160124_01320
12	12	1	50S ribosomal protein L17	ATCC29328_01910	ATCC53516_12900	07T609_01520	08T492_01080	09T408_01450	09T494_03310	12T272_01510	12T273_02590	12T306_01980	CCUG54800_02250	T151023_02100	T160124_01330
10	10	1	N-acetylmuramoyl-L-alanine amidase LytC pre	ATCC29328_01920	ATCC53516_12910	07T609_01530	08T492_01090	*	09T494_03320	12T272_01530	12T273_02600	*	CCUG54800_02270	T151023_02120	T160124_01340
12	12	1	30S ribosomal protein S4	ATCC29328_01930	ATCC53516_12920	07T609_01540	08T492_01100	09T408_01470	09T494_03330	12T272_01540	12T273_02610	12T306_02000	CCUG54800_02280	T151023_02130	T160124_01350
12	24	0.167	Putative peptidoglycan binding domain protein	ATCC29328_01940,ATCC2932	ATCC53516_15720,ATCC5351	07T609_07940,07T609_	08T492_07790,08T492_01	09T408_08020,09T408_	09T494_08980,09T494_	12T272_06740,12T272_	12T273_11640,12T273_	12T306_09850,12T306_	CCUG54800_02290,CCUG548	T151023_08320,T151023_	T160124_08240,T160124_01360
2	2	1	Cna protein B-type domain protein	ATCC29328_02100	*	*	08T492_01240	*	*	*	*	*	*	*	*
12	12	0.833	Trypsin-resistant surface T6 protein precursor	ATCC29328_02110	ATCC53516_02310	07T609_01610	08T492_01260	09T408_01570	09T494_03410	12T272_13480	12T273_02760	12T306_02150	CCUG54800_02470	T151023_02210	T160124_01420
12	12	0.403	Cna protein B-type domain protein	ATCC29328_02120	ATCC53516_02300	07T609_01620	08T492_01270	09T408_01580	09T494_03420	12T272_13470	12T273_02770	12T306_02160	CCUG54800_02480	T151023_02220	T160124_01430
12	24	0.167	Sortase family protein	ATCC29328_02130,ATCC2932	ATCC53516_02280,ATCC5351	07T609_01640,07T609_	08T492_01290,08T492_01	09T408_01600,09T408_	09T494_03440,09T494_	12T272_13450,12T272_	12T273_02790,12T273_	12T306_02180,12T306_	CCUG54800_02490,CCUG548	T151023_02240,T151023_	T160124_01450,T160124_01440
12	12	1	Sortase family protein	ATCC29328_02150	ATCC53516_02270	07T609_01650	08T492_01300	09T408_01610	09T494_03450	12T272_13440	12T273_02800	12T306_02190	CCUG54800_02510	T151023_02250	T160124_01460
12	12	1	hypothetical protein	ATCC29328_02160	ATCC53516_02240	07T609_01670	08T492_01320	09T408_01630	09T494_03480	12T272_13410	12T273_02820	12T306_02210	CCUG54800_02530	T151023_02270	T160124_01480
12	12	1	(R)-stereoselective amidase	ATCC29328_02170	ATCC53516_02230	07T609_01680	08T492_01330	09T408_01640	09T494_03490	12T272_13400	12T273_02830	12T306_02220	CCUG54800_02540	T151023_02280	T160124_01490
10	10	1	Adenosine monophosphate-protein transferas	ATCC29328_02180	ATCC53516_02220	07T609_01690	08T492_01340	*	09T494_03500	12T272_13390	12T273_02850	12T306_02230	CCUG54800_02550	T151023_02300	*
12	12	1	hypothetical protein	ATCC29328_02190	ATCC53516_02170	07T609_01720	08T492_01370	09T408_01680	09T494_03520	12T272_13370	12T273_02860	12T306_02250	CCUG54800_02560	T151023_02330	T160124_14510
12	12	1	hypothetical protein	ATCC29328_02200	ATCC53516_02160	07T609_01730	08T492_01380	09T408_01690	09T494_03530	12T272_13360	12T273_02870	12T306_02260	CCUG54800_02570	T151023_02340	T160124_14500
12	12	1	hypothetical protein	ATCC29328_02210	ATCC53516_02150	07T609_01740	08T492_01390	09T408_01700	09T494_03540	12T272_13350	12T273_02880	12T306_02270	CCUG54800_02580	T151023_02350	T160124_14490
12	12	1	hypothetical protein	ATCC29328_02220	ATCC53516_02140	07T609_01750	08T492_01400	09T408_01710	09T494_03550	12T272_13340	12T273_02890	12T306_02280	CCUG54800_02590	T151023_02360	T160124_14480

12	24	0.167	putative multidrug resistance ABC transporter	ATCC29328_02230,ATCC2932	ATCC53516_14650,ATCC5351	07T609_06870,07T609_	08T492_06720,08T492_01	09T408_06940,09T408_	09T494_07910,09T494_	12T272_05670,12T272_	12T273_10540,12T273_	12T306_08760,12T306_	CCUG54800_02600,CCUG548	T151023_07250,T151023_	T160124_07170,T160124_14470
12	24	0.167	putative multidrug resistance ABC transporter	ATCC29328_02240,ATCC2932	ATCC53516_14660,ATCC5351	07T609_06880,07T609_	08T492_06730,08T492_01	09T408_06950,09T408_	09T494_07920,09T494_	12T272_05680,12T272_	12T273_10550,12T273_	12T306_08770,12T306_	CCUG54800_02610,CCUG548	T151023_07260,T151023_	T160124_07180,T160124_14460
12	12	1	4-alpha-glucanotransferase	ATCC29328_02250	ATCC53516_02110	07T609_01780	08T492_01430	09T408_01740	09T494_03580	12T272_13310	12T273_02920	12T306_02310	CCUG54800_02620	T151023_02390	T160124_14450
12	12	1	Hydroxymethylpyrimidine/phosphomethylpyri	ATCC29328_02260	ATCC53516_02100	07T609_01790	08T492_01440	09T408_01750	09T494_03590	12T272_13300	12T273_02930	12T306_02320	CCUG54800_02630	T151023_02400	T160124_14440
12	24	0.167	Thiamine-phosphate synthase	ATCC29328_02270,ATCC2932	ATCC53516_07260,ATCC5351	07T609_03550,07T609_	08T492_03230,08T492_01	09T408_03680,09T408_	09T494_04360,09T494_	12T272_02160,12T272_	12T273_07110,12T273_	12T306_05270,12T306_	CCUG54800_02640,CCUG548	T151023_03860,T151023_	T160124_03590,T160124_14430
6	6	1	HTH-type transcriptional regulator MhqR	ATCC29328_02280	ATCC53516_02080	07T609_01810	08T492_01460	*	09T494_03610	*	*	12T306_02340	*	*	*
6	6	1	Quercetin 2,3-dioxygenase	ATCC29328_02290	ATCC53516_02070	07T609_01820	08T492_01470	*	09T494_03620	*	*	12T306_02350	*	*	*
6	6	1	Nitroreductase family protein	ATCC29328_02300	ATCC53516_02060	07T609_01830	08T492_01490	*	09T494_03630	*	*	12T306_02360	*	*	*
6	6	1	Multidrug export protein MepA	ATCC29328_02310	ATCC53516_02050	07T609_01850	08T492_01510	09T408_01770	*	*	*	12T306_02370	*	*	*
12	12	1	Uracil DNA glycosylase superfamily protein	ATCC29328_02320	ATCC53516_02040	07T609_01860	08T492_01520	09T408_01780	09T494_03640	12T272_13270	12T273_02950	12T306_02380	CCUG54800_02650	T151023_02420	T160124_14420
12	12	1	Serine dehydratase alpha chain	ATCC29328_02330	ATCC53516_02030	07T609_01870	08T492_01550	09T408_01790	09T494_03650	12T272_13260	12T273_02960	12T306_02390	CCUG54800_02660	T151023_02430	T160124_14410
1	1	1	Serine/threonine exchanger SteT	ATCC29328_02350	*	*	*	*	*	*	*	*	*	*	*
12	13	0.141	hypothetical protein	ATCC29328_02360	ATCC53516_02020	07T609_01880	08T492_01560	09T408_01800	09T494_03660	12T272_13250	12T273_02970	12T306_02400	CCUG54800_02670	T151023_02440,T151023_	T160124_14400
12	24	0.147	Cytosol non-specific dipeptidase	ATCC29328_02370,ATCC2932	ATCC53516_12380,ATCC5351	07T609_15640,07T609_	08T492_16660,08T492_01	09T408_15470,09T408_	09T494_18330,09T494_	12T272_15830,12T272_	12T273_18230,12T273_	12T306_16550,12T306_	CCUG54800_02680,CCUG548	T151023_15110,T151023_	T160124_15550,T160124_14390
12	20	0.155	Methionine aminotransferase	ATCC29328_02380,ATCC2932	ATCC53516_15240	07T609_07460	08T492_07310,08T492_01	09T408_07540,09T408_	09T494_08500,09T494_	12T272_06260,12T272_	12T273_11160,12T273_	12T306_09370,12T306_	CCUG54800_08600	T151023_07840	T160124_14380,T160124_07760
8	8	1	Phenylacetate-coenzyme A ligase	ATCC29328_02390	*	*	08T492_01590	09T408_01830	09T494_03690	12T272_13220	12T273_03000	12T306_02440	*	*	T160124_14370
8	8	1	Pyruvate synthase subunit PorB	ATCC29328_02400	*	*	08T492_01600	09T408_01840	09T494_03700	12T272_13210	12T273_03010	12T306_02450	*	*	T160124_14360
8	8	1	NADH-dependent phenylglyoxylate dehydroge	ATCC29328_02410	*	*	08T492_01610	09T408_01850	09T494_03710	12T272_13200	12T273_03020	12T306_02460	*	*	T160124_14350
8	8	1	Pyruvate synthase subunit PorD	ATCC29328_02420	*	*	08T492_01620	09T408_01860	09T494_03720	12T272_13190	12T273_03030	12T306_02470	*	*	T160124_14340
8	8	1	Pyruvate synthase subunit PorC	ATCC29328_02430	*	*	08T492_01630	09T408_01870	09T494_03730	12T272_13180	12T273_03040	12T306_02480	*	*	T160124_14330
12	12	1	hypothetical protein	ATCC29328_02440	ATCC53516_02000	07T609_01900	08T492_01640	09T408_01880	09T494_03740	12T272_13170	12T273_03050	12T306_02490	CCUG54800_02690	T151023_02460	T160124_14320
12	12	1	hypothetical protein	ATCC29328_02450	ATCC53516_01990	07T609_01910	08T492_01650	09T408_01890	09T494_03750	12T272_13160	12T273_03060	12T306_02500	CCUG54800_02700	T151023_02470	T160124_14310
11	11	1	DNA polymerase IV	ATCC29328_02460	ATCC53516_01980	07T609_01920	08T492_01660	09T408_01900	*	12T272_13150	12T273_03070	12T306_02510	CCUG54800_02710	T151023_02480	T160124_14300
12	14	0.143	hypothetical protein	ATCC29328_02470	ATCC53516_01970	07T609_01930	08T492_01670	09T408_01910	09T494_15840,09T494_	12T272_13140	12T273_03080	12T306_02520	CCUG54800_02720,CCUG548	T151023_02490	T160124_14290
12	14	0.143	HTH-type transcriptional regulator ImmR	ATCC29328_02480	ATCC53516_01960	07T609_01940	08T492_01680	09T408_01920	09T494_15830,09T494_	12T272_13110	12T273_03090	12T306_02530	CCUG54800_02730,CCUG548	T151023_02500	T160124_14280
11	11	1	hypothetical protein	ATCC29328_02490	ATCC53516_01950	07T609_01950	08T492_01690	09T408_01930	*	12T272_13100	12T273_03100	12T306_02540	CCUG54800_02740	T151023_02510	T160124_14270
12	12	1	zinc/cadmium-binding protein	ATCC29328_02500	ATCC53516_01940	07T609_01960	08T492_01700	09T408_01940	09T494_17170	12T272_14610	12T273_04880	12T306_03450	CCUG54800_02790	T151023_02520	T160124_14570
12	12	1	High-affinity zinc uptake system binding-protei	ATCC29328_02510	ATCC53516_01930	07T609_01970	08T492_01710	09T408_01950	09T494_17160	12T272_14600	12T273_04890	12T306_03460	CCUG54800_02800	T151023_02530	T160124_14560
12	24	0.167	High-affinity zinc uptake system ATP-binding p	ATCC29328_02520,ATCC2932	ATCC53516_17000,ATCC5351	07T609_09040,07T609_	08T492_08910,08T492_01	09T408_09230,09T408_	09T494_10190,09T494_	12T272_07940,12T272_	12T273_12820,12T273_	12T306_10970,12T306_	CCUG54800_02810,CCUG548	T151023_09450,T151023_	T160124_09430,T160124_14550
12	24	0.147	High-affinity zinc uptake system membrane pr	ATCC29328_02530,ATCC2932	ATCC53516_16990,ATCC5351	07T609_09030,07T609_	08T492_08900,08T492_01	09T408_09220,09T408_	09T494_10180,09T494_	12T272_07930,12T272_	12T273_12810,12T273_	12T306_10960,12T306_	CCUG54800_02820,CCUG548	T151023_09440,T151023_	T160124_09420,T160124_14540
12	12	1	hypothetical protein	ATCC29328_02540	ATCC53516_01900	07T609_02000	08T492_01740	09T408_01980	09T494_17130	12T272_14570	12T273_04920	12T306_03500	CCUG54800_02830	T151023_02560	T160124_14530
4	4	1	C protein alpha-antigen precursor	ATCC29328_02550	*	07T609_02010	08T492_01750	09T408_01990	*	*	*	*	*	*	*
11	11	0.182	Putative metal chaperone YciC	ATCC29328_02560	ATCC53516_08900	*	08T492_01530	09T408_02020	09T494_15450	12T272_12450	12T273_04960	12T306_03540	CCUG54800_02870	T151023_13690	T160124_01530
10	19	0.111	methylcobalamin:coenzyme M methyltransfer	ATCC29328_02570	ATCC53516_08870,ATCC5351	*	*	09T408_02050,09T408_	09T494_15420,09T494_	12T272_12420,12T272_	12T273_04990,12T273_	12T306_03570,12T306_	CCUG54800_02880,CCUG548	T151023_13660,T151023_	T160124_01560,T160124_01540
10	10	1	Methionine synthase	ATCC29328_02580	ATCC53516_08880	*	*	09T408_02040	09T494_15430	12T272_12430	12T273_04980	12T306_03560	CCUG54800_02890	T151023_13670	T160124_01550
10	10	1	Na(+)-translocating NADH-quinone reductase s	ATCC29328_02590	ATCC53516_08860	*	*	09T408_02060	09T494_15410	12T272_12410	12T273_05000	12T306_03580	CCUG54800_02910	T151023_13650	T160124_01570
10	10	1	hypothetical protein	ATCC29328_02600	ATCC53516_08850	*	*	09T408_02070	09T494_15400	12T272_12400	12T273_05010	12T306_03590	CCUG54800_02920	T151023_13640	T160124_01580
10	10	1	hypothetical protein	ATCC29328_02610	ATCC53516_08840	*	*	09T408_02080	09T494_15390	12T272_12390	12T273_05020	12T306_03600	CCUG54800_02930	T151023_13630	T160124_01590
12	24	0.167	hypothetical protein	ATCC29328_02620,ATCC2932	ATCC53516_11570,ATCC5351	07T609_16460,07T609_	08T492_17260,08T492_01	09T408_16290,09T408_	09T494_01020,09T494_	12T272_16570,12T272_	12T273_00280,12T273_	12T306_17200,12T306_	CCUG54800_02940,CCUG548	T151023_15890,T151023_	T160124_16290,T160124_01600
12	12	1	TVP38/TMEM64 family inner membrane prote	ATCC29328_02630	ATCC53516_08820	07T609_02060	08T492_01790	09T408_02100	09T494_15370	12T272_12370	12T273_05040	12T306_03620	CCUG54800_02950	T151023_13610	T160124_01610
12	12	1	Daunorubicin/doxorubicin resistance ATP-binc	ATCC29328_02650	ATCC53516_08800	07T609_02080	08T492_01810	09T408_02120	09T494_15350	12T272_12350	12T273_05060	12T306_03640	CCUG54800_02970	T151023_13590	T160124_01630
11	11	1	ABC-2 family transporter protein	ATCC29328_02660	ATCC53516_08790	07T609_02090	*	09T408_02130	09T494_15340	12T272_12340	12T273_05070	12T306_03650	CCUG54800_02980	T151023_13580	T160124_01640
12	12	1	ABC-2 family transporter protein	ATCC29328_02670	ATCC53516_08780	07T609_02100	08T492_01840	09T408_02140	09T494_15330	12T272_12330	12T273_05080	12T306_03660	CCUG54800_02990	T151023_13570	T160124_01650
12	12	1	High-affinity gluconate transporter	ATCC29328_02680	ATCC53516_08770	07T609_02110	08T492_01850	09T408_02150	09T494_15320	12T272_12320	12T273_05090	12T306_03670	CCUG54800_03000	T151023_13560	T160124_01660
12	12	1	Glycerate kinase	ATCC29328_02690	ATCC53516_08760	07T609_02120	08T492_01860	09T408_02160	09T494_15310	12T272_12310	12T273_05100	12T306_03680	CCUG54800_03010	T151023_13550	T160124_01670
12	12	1	Carbohydrate diacid regulator	ATCC29328_02700	ATCC53516_08750	07T609_02130	08T492_01870	09T408_02170	09T494_15300	12T272_12300	12T273_05110	12T306_03690	CCUG54800_03020	T151023_13540	T160124_01680
12	12	1	hypothetical protein	ATCC29328_02710	ATCC53516_08740	07T609_02140	08T492_01880	09T408_02180	09T494_15290	12T272_12290	12T273_05120	12T306_03700	CCUG54800_03030	T151023_13530	T160124_01690
12	12	1	Prolyl oligopeptidase family protein	ATCC29328_02720	ATCC53516_08730	07T609_02150	08T492_01890	09T408_02190	09T494_15280	12T272_12280	12T273_05130	12T306_03710	CCUG54800_03040	T151023_13520	T160124_01700
12	12	1	hypothetical protein	ATCC29328_02730	ATCC53516_08720	07T609_02160	08T492_01900	09T408_02200	09T494_15270	12T272_12260	12T273_05140	12T306_03720	CCUG54800_03050	T151023_13510	T160124_01710
12	24	0.167	Putative multidrug export ATP-binding/permea	ATCC29328_02740,ATCC2932	ATCC53516_01720,ATCC5351	07T609_14590,07T609_	08T492_15570,08T492_01	09T408_14210,09T408_	09T494_11140,09T494_	12T272_12980,12T272_	12T273_03260,12T273_	12T306_02680,12T306_	CCUG54800_03060,CCUG548	T151023_02700,T151023_	T160124_14120,T160124_01720
4	4	1	hypothetical protein	ATCC29328_02750	*	07T609_02180	08T492_01920	09T408_02220	*	*	*	*	*	*	*
12	12	1	Putative type II secretion system protein E	ATCC29328_02760	ATCC53516_08700	07T609_02190	08T492_01930	09T408_02230	09T494_15250	12T272_12240	12T273_05160	12T306_03740	CCUG54800_03070	T151023_13490	T160124_01730
12	12	1	Type II secretion system protein F	ATCC29328_02770	ATCC53516_08690	07T609_02200	08T492_01940	09T408_02240	09T494_15240	12T272_12230	12T273_05170	12T306_03750	CCUG54800_03080	T151023_13480	T160124_01740
1	1	1	Maltose/maltodextrin-binding protein precurs	ATCC29328_02790	*	*	*	*	*	*	*	*	*	*	*
1	1	1	hypothetical protein	ATCC29328_02820	*	*	*	*	*	*	*	*	*	*	*
12	12	1	Peptidase M16C associated	ATCC29328_02830	ATCC53516_08680	07T609_02220	08T492_01950	09T408_02250	09T494_15230	12T272_12200	12T273_05180	12T306_03760	CCUG54800_03090	T151023_13470	T160124_01750
12	12	1	Exodeoxyribonuclease	ATCC29328_02840	ATCC53516_08670	07T609_02230	08T492_01960	09T408_02260	09T494_15220	12T272_12190	12T273_05190	12T306_03770	CCUG54800_03100	T151023_13440	T160124_01780
12	12	1	dTDP-4-dehydrorhamnose reductase	ATCC29328_02850	ATCC53516_08660	07T609_02240	08T492_01970	09T408_02270	09T494_15210	12T272_12180	12T273_05200	12T306_03780	CCUG54800_03110	T151023_13430	T160124_01790
12	12	1	Proton glutamate symport protein	ATCC29328_02860	ATCC53516_08650	07T609_02250	08T492_01980	09T408_02280	09T494_15200	12T272_12170	12T273_05210	12T306_03790	CCUG54800_03120	T151023_13420	T160124_01800
12	12	1	TVP38/TMEM64 family inner membrane prote	ATCC29328_02870	ATCC53516_08640	07T609_02260	08T492_01990	09T408_02290	09T494_15190	12T272_12160	12T273_05220	12T306_03800	CCUG54800_03130	T151023_13410	T160124_01810
12	24	0.167	UDP-N-acetylmuramoyl-tripeptide--D-alanyl-D	ATCC29328_02880,ATCC2932	ATCC53516_12140,ATCC5351	07T609_15760,07T609_	08T492_16780,08T492_02	09T408_15700,09T408_	09T494_00510,09T494_	12T272_16050,12T272_	12T273_18500,12T273_	12T306_16660,12T306_	CCUG54800_03180,CCUG548	T151023_15220,T151023_	T160124_15770,T160124_01860
12	12	1	D-alanine--D-alanine ligase	ATCC29328_02890	ATCC53516_08580	07T609_02280	08T492_02010	09T408_02310	09T494_15170	12T272_12100	12T273_05240	12T306_03820	CCUG54800_03190	T151023_13390	T160124_01870
12	12	1	Maltodextrin phosphorylase	ATCC29328_02900	ATCC53516_08570	07T609_02290	08T492_02020	09T408_02320	09T494_15160	12T272_12090	12T273_05250	12T306_03830	CCUG54800_03200	T151023_13380	T160124_01880
12	12	1	hypothetical protein	ATCC29328_02910	ATCC53516_08560	07T609_02300	08T492_02030	09T408_02330	09T494_15150	12T272_12080	12T273_05260	12T306_03840	CCUG54800_03210	T151023_13370	T160124_01890
12	12	1	Magnesium transport protein CorA	ATCC29328_02920	ATCC53516_08550	07T609_02310	08T492_02040	09T408_02340	09T494_15140	12T272_12070	12T273_05270	12T306_03850	CCUG54800_03220	T151023_13360	T160124_01900
12	12	1	hypothetical protein	ATCC29328_02930	ATCC53516_08540	07T609_02320	08T492_02050	09T408_02350	09T494_15130	12T272_12060	12T273_05280	12T306_03860	CCUG54800_03230	T151023_13350	T160124_01910
12	12	1	Dihydrofolate reductase	ATCC29328_02940	ATCC53516_08530	07T609_02330	08T492_02060	09T408_02360	09T494_15120	12T272_12050	12T273_05290	12T306_03870	CCUG54800_03240	T151023_13340	T160124_01920
12	12	1	hypothetical protein	ATCC29328_02950	ATCC53516_08520	07T609_02340	08T492_02070	09T408_02370	09T494_15110	12T272_12040	12T273_05300	12T306_03880	CCUG54800_03250	T151023_13330	T160124_01930
12	24	0.167	Tagatose-6-phosphate kinase	ATCC29328_02960,ATCC2932	ATCC53516_04650,ATCC5351	07T609_13040,07T609_	08T492_14110,08T492_02	09T408_12470,09T408_	09T494_14290,09T494_	12T272_11280,12T272_	12T273_06250,12T273_	12T306_14650,12T306_	CCUG54800_03260,CCUG548	T151023_12580,T151023_	T160124_02810,T160124_01940
12	22	0.16	Oligopeptide transport ATP-binding protein Op	ATCC29328_02970	ATCC53516_04070,ATCC5351	07T609_12660,07T609_	08T492_12720,08T492_02	09T408_12110,09T408_	09T494_13290,09T494_	12T272_10780,12T272_	12T273_05320	12T306_14250,12T306_	CCUG54800_13950,CCUG548	T151023_13310,T151023_	T160124_12440,T160124_01950
12	12	1	Oligopeptide transport ATP-binding protein Op	ATCC29328_02980	ATCC53516_08490	07T609_02370	08T492_02100	09T408_02400	09T494_15080	12T272_12010	12T273_05330	12T306_03910	CCUG54800_03280	T151023_13300	T160124_01960
12	12	1	Glutathione transport system permease protei	ATCC29328_02990	ATCC53516_08480	07T609_02380	08T492_02110	09T408_02410	09T494_15070	12T272_12000	12T273_05340	12T306_03920	CCUG54800_03290	T151023_13290	T160124_01970
12	12	1	Oligopeptide transport system permease prot	ATCC29328_03000	ATCC53516_08460	07T609_02390	08T492_02120	09T408_02420	09T494_15060	12T272_11990	12T273_05350	12T306_03930	CCUG54800_03300	T151023_13280	T160124_01980
12	12	1	Oligopeptide-binding protein AppA precursor	ATCC29328_03010	ATCC53516_08450	07T609_02400	08T492_02130	09T408_02430	09T494_15050	12T272_11980	12T273_05360	12T306_03940	CCUG54800_03310	T151023_13270	T160124_01990
12	12	1	hypothetical protein	ATCC29328_03020	ATCC53516_08440	07T609_02410	08T492_02140	09T408_02440	09T494_15040	12T272_11970	12T273_05370	12T306_03950	CCUG54800_03320	T151023_13260	T160124_02000
12	12	1	DNA polymerase IV	ATCC29328_03030	ATCC53516_08430	07T609_02420	08T492_02150	09T408_02450	09T494_15030	12T272_11960	12T273_05380	12T306_03960	CCUG54800_03330	T151023_13250	T160124_02010
12	12	1	lineage-specific thermal regulator protein	ATCC29328_03040	ATCC53516_08420	07T609_02430	08T492_02160	09T408_02460	09T494_15020	12T272_11950	12T273_05390	12T306_03970	CCUG54800_03340	T151023_13240	T160124_02020
12	12	1	hypothetical protein	ATCC29328_03050	ATCC53516_08410	07T609_02440	08T492_02170	09T408_02470	09T494_15010	12T272_11940	12T273_05400	12T306_03980	CCUG54800_03350	T151023_13230	T160124_02030
12	12	1	Ribosomal RNA large subunit methyltransferas	ATCC29328_03060	ATCC53516_08400	07T609_02450	08T492_02180	09T408_02480	09T494_15000	12T272_11930	12T273_05410	12T306_03990	CCUG54800_03360	T151023_13220	T160124_02040
12	12	1	Phosphoserine phosphatase RsbU	ATCC29328_03070	ATCC53516_08390	07T609_02460	08T492_02190	09T408_02490	09T494_14990	12T272_11920	12T273_05420	12T306_04000	CCUG54800_03370	T151023_13210	T160124_02050
12	12	1	Glucose-6-phosphate isomerase	ATCC29328_03080	ATCC53516_08380	07T609_02470	08T492_02200	09T408_02500	09T494_14980	12T272_11910	12T273_05430	12T306_04010	CCUG54800_03380	T151023_13200	T160124_02060
12	12	1	hypothetical protein	ATCC29328_03090	ATCC53516_08370	07T609_02480	08T492_02210	09T408_02510	09T494_14970	12T272_11900	12T273_05440	12T306_04020	CCUG54800_03390	T151023_13190	T160124_02070
12	12	1	AAA-like domain protein	ATCC29328_03100	ATCC53516_08360	07T609_02490	08T492_02220	09T408_02520	09T494_14960	12T272_11890	12T273_05450	12T306_04030	CCUG54800_03400	T151023_13180	T160124_02080
12	12	1	Glycine/sarcosine N-methyltransferase	ATCC29328_03110	ATCC53516_08320	07T609_02500	08T492_02230	09T408_02530	09T494_14950	12T272_11880	12T273_05460	12T306_04040	CCUG54800_03410	T151023_13170	T160124_02090
12	13	0.141	hypothetical protein	ATCC29328_03120,ATCC2932	ATCC53516_08310	07T609_02510	08T492_02240	09T408_02540	09T494_14940	12T272_11870	12T273_05470	12T306_04050	CCUG54800_03420	T151023_13160	T160124_02100
12	24	0.167	23S rRNA methyltransferase	ATCC29328_03130,ATCC2932	ATCC53516_16480,ATCC5351	07T609_08660,07T609_	08T492_08510,08T492_02	09T408_08740,09T408_	09T494_09700,09T494_	12T272_07460,12T272_	12T273_12350,12T273_	12T306_10580,12T306_	CCUG54800_03430,CCUG548	T151023_09040,T151023_	T160124_08960,T160124_02110
12	12	1	Phosphoglycolate phosphatase	ATCC29328_03140	ATCC53516_08290	07T609_02530	08T492_02260	09T408_02560	09T494_14920	12T272_11850	12T273_05490	12T306_04070	CCUG54800_03440	T151023_13140	T160124_02120
12	12	1	hypothetical protein	ATCC29328_03150	ATCC53516_08280	07T609_02540	08T492_02270	09T408_02570	09T494_14910	12T272_11840	12T273_05500	12T306_04080	CCUG54800_03450	T151023_13130	T160124_02130
12	12	1	formate dehydrogenase accessory protein	ATCC29328_03160	ATCC53516_08270	07T609_02550	08T492_02280	09T408_02580	09T494_14900	12T272_11830	12T273_05510	12T306_04090	CCUG54800_03460	T151023_13120	T160124_02140
12	12	1	Nitrite transporter NirC	ATCC29328_03170	ATCC53516_08260	07T609_02560	08T492_02290	09T408_02590	09T494_14890	12T272_11820	12T273_05520	12T306_04100	CCUG54800_03470	T151023_13110	T160124_02150
12	12	1	Molybdopterin molybdenumtransferase	ATCC29328_03180	ATCC53516_08250	07T609_02570	08T492_02300	09T408_02600	09T494_14880	12T272_11810	12T273_05530	12T306_04110	CCUG54800_03480	T151023_13100	T160124_02160
12	12	1	Molybdenum cofactor guanylyltransferase	ATCC29328_03190	ATCC53516_08240	07T609_02580	08T492_02310	09T408_02610	09T494_14870	12T272_11800	12T273_05540	12T306_04120	CCUG54800_03490	T151023_13090	T160124_02170
12	12	1	NADH pyrophosphatase	ATCC29328_03200	ATCC53516_08230	07T609_02590	08T492_02320	09T408_02620	09T494_14860	12T272_11790	12T273_05550	12T306_04130	CCUG54800_03500	T151023_13080	T160124_02180
12	12	1	EamA-like transporter family protein	ATCC29328_03210	ATCC53516_08220	07T609_02600	08T492_02330	09T408_02630	09T494_14850	12T272_11780	12T273_05560	12T306_04140	CCUG54800_03510	T151023_13070	T160124_02190
12	12	1	NAD-dependent protein deacetylase	ATCC29328_03220	ATCC53516_08210	07T609_02610	08T492_02340	09T408_02640	09T494_14840	12T272_11770	12T273_05570	12T306_04150	CCUG54800_03520	T151023_13060	T160124_02200
12	12	1	hypothetical protein	ATCC29328_03230	ATCC53516_08200	07T609_02620	08T492_02350	09T408_02650	09T494_14830	12T272_11760	12T273_05580	12T306_04160	CCUG54800_03530	T151023_13050	T160124_02210
12	19	0.153	DegV domain-containing protein	ATCC29328_03240,ATCC2932	ATCC53516_14080	07T609_06300,07T609_	08T492_06150,08T492_02	09T408_06390,09T408_	09T494_07340	12T272_05100	12T273_09970,12T273_	12T306_08170	CCUG54800_03540,CCUG548	T151023_06680,T151023_	T160124_06600
12	12	1	Thiol-disulfide oxidoreductase ResA	ATCC29328_03250	ATCC53516_08190	07T609_02640	08T492_02370	09T408_02670	09T494_14820	12T272_11750	12T273_05600	12T306_04170	CCUG54800_03550	T151023_13030	T160124_02220
12	12	1	Putative electron transport protein YccM	ATCC29328_03260	ATCC53516_08170	07T609_02650	08T492_02380	09T408_02680	09T494_14810	12T272_11740	12T273_05620	12T306_04190	CCUG54800_03570	T151023_13010	T160124_02240
12	12	1	Thiosulfate sulfurtransferase PspE precursor	ATCC29328_03270	ATCC53516_08160	07T609_02660	08T492_02390	09T408_02690	09T494_14800	12T272_11730	12T273_05630	12T306_04200	CCUG54800_03580	T151023_13000	T160124_02250
12	12	1	2-phospho-L-lactate guanylyltransferase	ATCC29328_03280	ATCC53516_08150	07T609_02670	08T492_02400	09T408_02700	09T494_14790	12T272_11720	12T273_05640	12T306_04210	CCUG54800_03590	T151023_12990	T160124_02260
12	12	1	Glycosyl transferase family 2	ATCC29328_03290	ATCC53516_08140	07T609_02680	08T492_02410	09T408_02710	09T494_14780	12T272_11710	12T273_05650	12T306_04220	CCUG54800_03600	T151023_12980	T160124_02270
12	12	1	succinate dehydrogenase/fumarate reductase	ATCC29328_03300	ATCC53516_08130	07T609_02690	08T492_02420	09T408_02720	09T494_14770	12T272_11700	12T273_05660	12T306_04230	CCUG54800_03610	T151023_12970	T160124_02280
12	12	1	Antilisterial bacteriocin subtilosin biosynthesis	ATCC29328_03310	ATCC53516_08120	07T609_02700	08T492_02430	09T408_02730	09T494_14760	12T272_11690	12T273_05670	12T306_04240	CCUG54800_03620	T151023_12960	T160124_02290
12	12	1	Putative redox-active protein (C_GCAxxG_C_C)	ATCC29328_03320	ATCC53516_08110	07T609_02710	08T492_02440	09T408_02740	09T494_14750	12T272_11680	12T273_05680	12T306_04250	CCUG54800_03630	T151023_12950	T160124_02300
12	12	1	D-amino acid dehydrogenase small subunit	ATCC29328_03330	ATCC53516_08080	07T609_02720	08T492_02450	09T408_02750	09T494_14740	12T272_11670	12T273_05690	12T306_04260	CCUG54800_03640	T151023_12940	T160124_02310
12	12	1	Tagatose-6-phosphate kinase	ATCC29328_03340	ATCC53516_08070	07T609_02730	08T492_02460	09T408_02760	09T494_14730	12T272_11660	12T273_05700	12T306_04270	CCUG54800_03650	T151023_12930	T160124_02320
12	12	1	Inosine-5'-monophosphate dehydrogenase	ATCC29328_03350	ATCC53516_08060	07T609_02740	08T492_02470	09T408_02770	09T494_14720	12T272_11650	12T273_05710	12T306_04280	CCUG54800_03660	T151023_12920	T160124_02330
12	12	1	N5-carboxyaminoimidazole ribonucleotide mu	ATCC29328_03360	ATCC53516_08050	07T609_02750	08T492_02480	09T408_02780	09T494_14710	12T272_11640	12T273_05720	12T306_04290	CCUG54800_03670	T151023_12910	T160124_02340
12	12	1	Phosphoribosylformylglycinamidine cyclo-ligas	ATCC29328_03370	ATCC53516_08040	07T609_02760	08T492_02490	09T408_02790	09T494_14700	12T272_11630	12T273_05730	12T306_04300	CCUG54800_03680	T151023_12900	T160124_02350
12	15	0.212	methylcobalamin:coenzyme M methyltransfer	ATCC29328_03380	ATCC53516_08910	07T609_02020	08T492_01760	09T408_02010	09T494_15460	12T272_12460	12T273_04950,12T273_	12T306_03530,12T306_	CCUG54800_02860	T151023_13700	T160124_01520,T160124_02360
12	24	0.167	putative ABC transporter ATP-binding protein	ATCC29328_03390,ATCC2932	ATCC53516_03620,ATCC5351	07T609_12210,07T609_	08T492_12260,08T492_02	09T408_11650,09T408_	09T494_12840,09T494_	12T272_10330,12T272_	12T273_15810,12T273_	12T306_13820,12T306_	CCUG54800_03690,CCUG548	T151023_11700,T151023_	T160124_11990,T160124_02370
12	12	1	GMP synthase [glutamine-hydrolyzing]	ATCC29328_03400	ATCC53516_08010	07T609_02780	08T492_02510	09T408_02810	09T494_14680	12T272_11610	12T273_05760	12T306_04340	CCUG54800_03700	T151023_12880	T160124_02380
1	1	1	hypothetical protein	ATCC29328_03410	*	*	*	*	*	*	*	*	*	*	*
2	2	1	hypothetical protein	ATCC29328_03420	*	*	*	*	09T494_14600	*	*	*	*	*	*
9	9	1	hypothetical protein	ATCC29328_03430	*	07T609_02860	08T492_02550	09T408_02930	*	*	12T273_05820	12T306_15030	CCUG54800_14900	T151023_12840	T160124_02440
4	4	1	Aspartokinase 3	ATCC29328_03440	*	07T609_02870	08T492_02560	09T408_02940	*	*	*	*	*	*	*
4	4	1	Aspartate-semialdehyde dehydrogenase	ATCC29328_03450	*	07T609_02880	08T492_02570	09T408_02950	*	*	*	*	*	*	*
4	4	1	Homoserine dehydrogenase	ATCC29328_03460	*	07T609_02890	08T492_02580	09T408_02960	*	*	*	*	*	*	*
12	16	0.147	Threonine synthase	ATCC29328_03470,ATCC2932	ATCC53516_04260	07T609_12830,07T609_	08T492_02590,08T492_12	09T408_12280,09T408_	09T494_13530	12T272_11030	12T273_16450	12T306_14430	CCUG54800_14130	T151023_12380	T160124_12630
12	16	0.147	Magnesium-transporting ATPase, P-type 1	ATCC29328_03480,ATCC2932	ATCC53516_11970	07T609_16120,07T609_	08T492_02600,08T492_16	09T408_15930,09T408_	09T494_00660	12T272_16220	12T273_18720	12T306_16840	CCUG54800_18150	T151023_15530	T160124_15960
10	10	0.3	HTH-type transcriptional regulator ImmR	ATCC29328_03490	ATCC53516_07940	07T609_02920	08T492_02610	09T408_03000	*	12T272_11590	12T273_05830	12T306_15020	*	T151023_12830	T160124_02460
10	13	0.268	N-acyl-L-amino acid amidohydrolase	ATCC29328_03500	ATCC53516_07910	07T609_00390,07T609_	08T492_02620	09T408_15880,09T408_	*	12T272_13690,12T272_	12T273_06460	12T306_04640	CCUG54800_18100	*	T160124_03000
8	8	1	2-keto-3-deoxygluconate permease	ATCC29328_03510	ATCC53516_07900	07T609_02940	08T492_02630	09T408_03080	*	*	12T273_06480	12T306_04650	*	*	T160124_03010
2	2	1	hypothetical protein	ATCC29328_03520	*	*	*	*	*	*	*	12T306_04660	*	*	*
2	2	1	hypothetical protein	ATCC29328_03530	*	*	*	*	*	*	*	12T306_04680	*	*	*
2	2	1	hypothetical protein	ATCC29328_03540	*	*	*	*	*	*	*	12T306_04690	*	*	*
11	12	0.116	N-6 DNA Methylase	ATCC29328_03550	ATCC53516_07890	07T609_02950	08T492_02640	09T408_03090	09T494_16370	*	12T273_06490	12T306_04700	CCUG54800_03890,CCUG548	T151023_03280	T160124_03030
9	9	1	hypothetical protein	ATCC29328_03560	ATCC53516_07880	07T609_02960	08T492_02650	09T408_03100	*	*	12T273_06500	12T306_04710	CCUG54800_03900	*	T160124_03040
11	12	0.152	BFD-like [2Fe-2S] binding domain protein	ATCC29328_03570	ATCC53516_07870	07T609_02970	08T492_02660	09T408_03110	09T494_16360	*	12T273_06510	12T306_04720	CCUG54800_03910	T151023_03290,T151023_	T160124_03050
7	8	0.232	Type I restriction enzyme EcoR124II R protein	ATCC29328_03580	ATCC53516_07860	07T609_02980,07T609_	08T492_02670	09T408_03120	*	*	12T273_06520	*	CCUG54800_03920	*	*
12	12	1	Transcriptional regulator CtsR	ATCC29328_03590	ATCC53516_07830	07T609_03020	08T492_02700	09T408_03140	09T494_16340	12T272_13670	12T273_06550	12T306_04740	CCUG54800_03940	T151023_03320	T160124_03070
12	12	1	hypothetical protein	ATCC29328_03600	ATCC53516_07820	07T609_03030	08T492_02710	09T408_03150	09T494_16330	12T272_13660	12T273_06560	12T306_04750	CCUG54800_03950	T151023_03330	T160124_03080
12	12	1	Putative ATP:guanido phosphotransferase	ATCC29328_03610	ATCC53516_07810	07T609_03040	08T492_02720	09T408_03160	09T494_16320	12T272_13650	12T273_06570	12T306_04760	CCUG54800_03960	T151023_03340	T160124_03090
12	12	1	ATP-dependent Clp protease ATP-binding subu	ATCC29328_03620	ATCC53516_07800	07T609_03050	08T492_02730	09T408_03170	09T494_16310	12T272_13640	12T273_06580	12T306_04770	CCUG54800_03970	T151023_03350	T160124_03100
12	12	1	hypothetical protein	ATCC29328_03630	ATCC53516_07790	07T609_03060	08T492_02740	09T408_03180	09T494_16300	12T272_13630	12T273_06590	12T306_04780	CCUG54800_03980	T151023_03360	T160124_03110
12	12	1	hypothetical protein	ATCC29328_03640	ATCC53516_07780	07T609_03070	08T492_02750	09T408_03190	09T494_03830	12T272_01560	12T273_06600	12T306_04790	CCUG54800_03990	T151023_03370	T160124_03120
12	12	1	hypothetical protein	ATCC29328_03650	ATCC53516_07770	07T609_03080	08T492_02760	09T408_03200	09T494_03840	12T272_01570	12T273_06610	12T306_04800	CCUG54800_04000	T151023_03380	T160124_03130
12	12	1	ADP-ribosyl-[dinitrogen reductase] glycohydro	ATCC29328_03660	ATCC53516_07760	07T609_03100	08T492_02780	09T408_03210	09T494_03860	12T272_01590	12T273_06630	12T306_04820	CCUG54800_04010	T151023_03390	T160124_03140
12	24	0.167	Aldehyde-alcohol dehydrogenase	ATCC29328_03670,ATCC2932	ATCC53516_04270,ATCC5351	07T609_12840,07T609_	08T492_12900,08T492_02	09T408_12290,09T408_	09T494_13540,09T494_	12T272_11040,12T272_	12T273_16460,12T273_	12T306_14440,12T306_	CCUG54800_04070,CCUG548	T151023_12390,T151023_	T160124_12640,T160124_03180
12	12	1	hypothetical protein	ATCC29328_03680	ATCC53516_07690	07T609_03150	08T492_02830	09T408_03270	09T494_03930	12T272_01650	12T273_06690	12T306_04870	CCUG54800_04080	T151023_03410	T160124_03190
12	12	1	Ribose-5-phosphate isomerase B	ATCC29328_03700	ATCC53516_07670	07T609_03170	08T492_02850	09T408_03300	09T494_03950	12T272_01670	12T273_06730	12T306_04890	CCUG54800_04100	T151023_03430	T160124_03210
12	12	1	DNA polymerase I	ATCC29328_03710	ATCC53516_07660	07T609_03180	08T492_02860	09T408_03310	09T494_03960	12T272_01680	12T273_06740	12T306_04900	CCUG54800_04110	T151023_03440	T160124_03220
12	12	1	Dephospho-CoA kinase	ATCC29328_03720	ATCC53516_07650	07T609_03190	08T492_02870	09T408_03320	09T494_03970	12T272_01690	12T273_06750	12T306_04910	CCUG54800_04120	T151023_03450	T160124_03230
12	12	1	Soluble lytic murein transglycosylase precursor	ATCC29328_03730	ATCC53516_07640	07T609_03200	08T492_02880	09T408_03330	09T494_03980	12T272_01700	12T273_06760	12T306_04920	CCUG54800_04130	T151023_03460	T160124_03240
12	12	1	Electron transport complex protein RnfC	ATCC29328_03750	ATCC53516_07620	07T609_03220	08T492_02900	09T408_03350	09T494_04000	12T272_01720	12T273_06780	12T306_04940	CCUG54800_04150	T151023_03480	T160124_03260
12	24	0.167	Electron transport complex protein RnfD	ATCC29328_03760,ATCC2932	ATCC53516_02560,ATCC5351	07T609_11160,07T609_	08T492_11080,08T492_02	09T408_10660,09T408_	09T494_11830,09T494_	12T272_09300,12T272_	12T273_14760,12T273_	12T306_12770,12T306_	CCUG54800_04160,CCUG548	T151023_10660,T151023_	T160124_10780,T160124_03270
12	12	1	Electron transport complex protein RnfG	ATCC29328_03770	ATCC53516_07600	07T609_03240	08T492_02920	09T408_03370	09T494_04020	12T272_01740	12T273_06800	12T306_04960	CCUG54800_04170	T151023_03500	T160124_03280
12	24	0.167	Na(+)-translocating NADH-quinone reductase s	ATCC29328_03780,ATCC2932	ATCC53516_02540,ATCC5351	07T609_11140,07T609_	08T492_11060,08T492_02	09T408_10640,09T408_	09T494_11810,09T494_	12T272_09280,12T272_	12T273_14740,12T273_	12T306_12750,12T306_	CCUG54800_04180,CCUG548	T151023_10640,T151023_	T160124_10760,T160124_03290
12	24	0.167	Electron transport complex protein RnfA	ATCC29328_03790,ATCC2932	ATCC53516_02530,ATCC5351	07T609_11130,07T609_	08T492_11050,08T492_02	09T408_10630,09T408_	09T494_11800,09T494_	12T272_09270,12T272_	12T273_14730,12T273_	12T306_12740,12T306_	CCUG54800_04190,CCUG548	T151023_10630,T151023_	T160124_10750,T160124_03300
12	12	1	Electron transport complex protein rnfB	ATCC29328_03800	ATCC53516_07570	07T609_03270	08T492_02950	09T408_03400	09T494_04050	12T272_01770	12T273_06830	12T306_04990	CCUG54800_04200	T151023_03530	T160124_03310
12	12	1	hypothetical protein	ATCC29328_03810	ATCC53516_07560	07T609_03280	08T492_02960	09T408_03410	09T494_04090	12T272_01820	12T273_06840	12T306_05000	CCUG54800_04240	T151023_03570	T160124_03320
12	12	1	hypothetical protein	ATCC29328_03820	ATCC53516_07550	07T609_03290	08T492_02970	09T408_03420	09T494_04100	12T272_01830	12T273_06850	12T306_05010	CCUG54800_04250	T151023_03580	T160124_03330
12	12	1	Pyrrolidone-carboxylate peptidase	ATCC29328_03830	ATCC53516_07540	07T609_03300	08T492_02980	09T408_03430	09T494_04110	12T272_01840	12T273_06860	12T306_05020	CCUG54800_04260	T151023_03590	T160124_03340
12	12	1	hypothetical protein	ATCC29328_03840	ATCC53516_07530	07T609_03310	08T492_02990	09T408_03440	09T494_04120	12T272_01850	12T273_06870	12T306_05030	CCUG54800_04270	T151023_03600	T160124_03350
12	12	1	Heptaprenyl diphosphate synthase componen	ATCC29328_03850	ATCC53516_07520	07T609_03320	08T492_03000	09T408_03450	09T494_04130	12T272_01860	12T273_06880	12T306_05040	CCUG54800_04280	T151023_03610	T160124_03360
12	12	1	hypothetical protein	ATCC29328_03860	ATCC53516_07510	07T609_03330	08T492_03010	09T408_03460	09T494_04140	12T272_01870	12T273_06890	12T306_05050	CCUG54800_04290	T151023_03620	T160124_03370
12	24	0.167	Rod shape-determining protein MreB	ATCC29328_03870,ATCC2932	ATCC53516_03870,ATCC5351	07T609_12460,07T609_	08T492_12520,08T492_03	09T408_11910,09T408_	09T494_13090,09T494_	12T272_10580,12T272_	12T273_16060,12T273_	12T306_14050,12T306_	CCUG54800_04300,CCUG548	T151023_11940,T151023_	T160124_12240,T160124_03380
12	12	1	Cell shape-determining protein MreC precurso	ATCC29328_03880	ATCC53516_07490	07T609_03350	08T492_03030	09T408_03480	09T494_04160	12T272_01890	12T273_06910	12T306_05070	CCUG54800_04310	T151023_03640	T160124_03390
12	12	1	rod shape-determining protein MreD	ATCC29328_03890	ATCC53516_07480	07T609_03360	08T492_03040	09T408_03490	09T494_04170	12T272_01900	12T273_06920	12T306_05080	CCUG54800_04320	T151023_03650	T160124_03400
12	12	1	Stage V sporulation protein D	ATCC29328_03900	ATCC53516_07470	07T609_03370	08T492_03050	09T408_03500	09T494_04180	12T272_01910	12T273_06930	12T306_05090	CCUG54800_04330	T151023_03660	T160124_03410
12	12	1	hypothetical protein	ATCC29328_03910	ATCC53516_07460	07T609_03380	08T492_03060	09T408_03510	09T494_04190	12T272_01920	12T273_06940	12T306_05100	CCUG54800_04340	T151023_03670	T160124_03420
12	12	1	ribonuclease G	ATCC29328_03920	ATCC53516_07450	07T609_03390	08T492_03070	09T408_03520	09T494_04200	12T272_01930	12T273_06950	12T306_05110	CCUG54800_04350	T151023_03680	T160124_03430
12	12	1	50S ribosomal protein L21	ATCC29328_03930	ATCC53516_07440	07T609_03400	08T492_03080	09T408_03530	09T494_04210	12T272_01940	12T273_06960	12T306_05120	CCUG54800_04360	T151023_03690	T160124_03440
12	12	1	hypothetical protein	ATCC29328_03940	ATCC53516_07430	07T609_03410	08T492_03090	09T408_03540	09T494_04220	12T272_01950	12T273_06970	12T306_05130	CCUG54800_04370	T151023_03700	T160124_03450
12	12	1	50S ribosomal protein L27	ATCC29328_03950	ATCC53516_07420	07T609_03420	08T492_03100	09T408_03550	09T494_04230	12T272_01960	12T273_06980	12T306_05140	CCUG54800_04380	T151023_03710	T160124_03460
12	12	1	GTPase Obg	ATCC29328_03960	ATCC53516_07410	07T609_03430	08T492_03110	09T408_03560	09T494_04240	12T272_01970	12T273_06990	12T306_05150	CCUG54800_04390	T151023_03720	T160124_03470
12	12	1	RNA-binding protein	ATCC29328_03970	ATCC53516_07400	07T609_03440	08T492_03120	09T408_03570	09T494_04250	12T272_01980	12T273_07000	12T306_05160	CCUG54800_04400	T151023_03730	T160124_03480
12	12	1	putative nicotinate-nucleotide adenylyltransfe	ATCC29328_03980	ATCC53516_07390	07T609_03450	08T492_03130	09T408_03580	09T494_04260	12T272_01990	12T273_07010	12T306_05170	CCUG54800_04410	T151023_03740	T160124_03490
12	12	1	putative nicotinate-nucleotide adenylyltransfe	ATCC29328_03990	ATCC53516_07380	07T609_03460	08T492_03140	09T408_03590	09T494_04270	12T272_02000	12T273_07020	12T306_05180	CCUG54800_04420	T151023_03750	T160124_03500
12	12	1	Ribosomal silencing factor RsfS	ATCC29328_04000	ATCC53516_07370	07T609_03470	08T492_03150	09T408_03600	09T494_04280	12T272_02010	12T273_07030	12T306_05190	CCUG54800_04430	T151023_03760	T160124_03510
12	12	1	Enamine/imine deaminase	ATCC29328_04010	ATCC53516_07360	07T609_03480	08T492_03160	09T408_03610	09T494_04290	12T272_02020	12T273_07040	12T306_05200	CCUG54800_04440	T151023_03770	T160124_03520
12	12	1	N-acetylmuramoyl-L-alanine amidase LytC pre	ATCC29328_04020	ATCC53516_07350	07T609_03490	08T492_03170	09T408_03620	09T494_04300	12T272_02090	12T273_07050	12T306_05210	CCUG54800_04450	T151023_03780	T160124_03530
12	12	1	Ribosome-binding ATPase YchF	ATCC29328_04030	ATCC53516_07310	07T609_03500	08T492_03180	09T408_03630	09T494_04310	12T272_02100	12T273_07060	12T306_05220	CCUG54800_04460	T151023_03810	T160124_03540
12	12	1	Xaa-Pro dipeptidase	ATCC29328_04040	ATCC53516_07300	07T609_03510	08T492_03190	09T408_03640	09T494_04320	12T272_02120	12T273_07070	12T306_05230	CCUG54800_04470	T151023_03820	T160124_03550
12	12	1	UvrABC system protein C	ATCC29328_04050	ATCC53516_07290	07T609_03520	08T492_03200	09T408_03650	09T494_04330	12T272_02130	12T273_07080	12T306_05240	CCUG54800_04480	T151023_03830	T160124_03560
12	12	1	Thiamine-precursor transporter protein (ThiW	ATCC29328_04060	ATCC53516_07280	07T609_03530	08T492_03210	09T408_03660	09T494_04340	12T272_02140	12T273_07090	12T306_05250	CCUG54800_04490	T151023_03840	T160124_03570
12	12	1	Hydroxyethylthiazole kinase	ATCC29328_04070	ATCC53516_07270	07T609_03540	08T492_03220	09T408_03670	09T494_04350	12T272_02150	12T273_07100	12T306_05260	CCUG54800_04500	T151023_03850	T160124_03580
12	12	1	hypothetical protein	ATCC29328_04090	ATCC53516_07250	07T609_03560	08T492_03240	09T408_03690	09T494_04370	12T272_02170	12T273_07120	12T306_05280	CCUG54800_04520	T151023_03870	T160124_03600
12	12	1	HTH-type transcriptional repressor YtrA	ATCC29328_04100	ATCC53516_07240	07T609_03570	08T492_03250	09T408_03700	09T494_04380	12T272_02180	12T273_07130	12T306_05290	CCUG54800_04530	T151023_03880	T160124_03610
12	12	1	hypothetical protein	ATCC29328_04110	ATCC53516_07230	07T609_03580	08T492_03260	09T408_03710	09T494_04390	12T272_02190	12T273_07140	12T306_05300	CCUG54800_04540	T151023_03890	T160124_03620
12	24	0.167	N-acetylmuramoyl-L-alanine amidase LytC pre	ATCC29328_04120,ATCC2932	ATCC53516_07200,ATCC5351	07T609_03600,07T609_	08T492_03300,08T492_03	09T408_03730,09T408_	09T494_04420,09T494_	12T272_02220,12T272_	12T273_07170,12T273_	12T306_05330,12T306_	CCUG54800_04550,CCUG548	T151023_03920,T151023_	T160124_03640,T160124_03630
12	12	1	HPr kinase/phosphorylase	ATCC29328_04140	ATCC53516_07190	07T609_03610	08T492_03310	09T408_03740	09T494_04430	12T272_02230	12T273_07180	12T306_05340	CCUG54800_04580	T151023_03930	T160124_03650
12	12	1	Pyruvate-flavodoxin oxidoreductase	ATCC29328_04150	ATCC53516_07180	07T609_03620	08T492_03320	09T408_03750	09T494_04440	12T272_02240	12T273_07190	12T306_05350	CCUG54800_04590	T151023_03940	T160124_03660
12	12	1	Acyl-CoA dehydrogenase	ATCC29328_04160	ATCC53516_07170	07T609_03630	08T492_03330	09T408_03760	09T494_04450	12T272_02250	12T273_07200	12T306_05360	CCUG54800_04600	T151023_03950	T160124_03670
12	12	1	Phosphate-import protein PhnD precursor	ATCC29328_04170	ATCC53516_07140	07T609_03640	08T492_03340	09T408_03770	09T494_04480	12T272_02280	12T273_07220	12T306_05390	CCUG54800_04620	T151023_03980	T160124_03680
12	12	1	5-methyltetrahydropteroyltriglutamate--homo	ATCC29328_04180	ATCC53516_07130	07T609_03650	08T492_03350	09T408_03780	09T494_04490	12T272_02290	12T273_07230	12T306_05400	CCUG54800_04630	T151023_03990	T160124_03690
12	12	1	OsmC-like protein	ATCC29328_04190	ATCC53516_07120	07T609_03660	08T492_03360	09T408_03790	09T494_04500	12T272_02300	12T273_07240	12T306_05410	CCUG54800_04640	T151023_04000	T160124_03700
12	12	1	putative tryptophan transport protein	ATCC29328_04200	ATCC53516_07110	07T609_03670	08T492_03370	09T408_03800	09T494_04510	12T272_02310	12T273_07250	12T306_05420	CCUG54800_04650	T151023_04010	T160124_03710
12	24	0.167	Uridine phosphorylase	ATCC29328_04210,ATCC2932	ATCC53516_06960,ATCC5351	07T609_03810,07T609_	08T492_03510,08T492_03	09T408_03960,09T408_	09T494_04650,09T494_	12T272_02440,12T272_	12T273_07390,12T273_	12T306_05560,12T306_	CCUG54800_04660,CCUG548	T151023_04140,T151023_	T160124_03850,T160124_03720
12	12	1	YacP-like NYN domain protein	ATCC29328_04220	ATCC53516_07090	07T609_03690	08T492_03390	09T408_03820	09T494_04530	12T272_02330	12T273_07270	12T306_05440	CCUG54800_04670	T151023_04030	T160124_03730
12	12	1	23S rRNA (guanosine-2'-O-)-methyltransferase	ATCC29328_04230	ATCC53516_07080	07T609_03700	08T492_03400	09T408_03830	09T494_04540	12T272_02340	12T273_07280	12T306_05450	CCUG54800_04680	T151023_04040	T160124_03740
12	12	1	Alpha-amylase precursor	ATCC29328_04240	ATCC53516_07070	07T609_03710	08T492_03410	09T408_03840	09T494_04550	12T272_02350	12T273_07290	12T306_05460	CCUG54800_04690	T151023_04050	T160124_03750
12	12	1	Ribonuclease H	ATCC29328_04250	ATCC53516_07060	07T609_03720	08T492_03420	09T408_03870	09T494_04560	12T272_02360	12T273_07300	12T306_05470	CCUG54800_04700	T151023_04060	T160124_03760
12	12	1	Hemolysin C	ATCC29328_04260	ATCC53516_07050	07T609_03730	08T492_03430	09T408_03880	09T494_04570	12T272_02370	12T273_07310	12T306_05480	CCUG54800_04710	T151023_04070	T160124_03770
12	12	1	Adenine phosphoribosyltransferase	ATCC29328_04270	ATCC53516_07040	07T609_03740	08T492_03440	09T408_03890	09T494_04580	12T272_02380	12T273_07320	12T306_05490	CCUG54800_04720	T151023_04080	T160124_03780
12	12	1	Nicotinate phosphoribosyltransferase pncB2	ATCC29328_04280	ATCC53516_07020	07T609_03750	08T492_03450	09T408_03900	09T494_04590	12T272_02390	12T273_07330	12T306_05500	CCUG54800_04730	T151023_04090	T160124_03790
12	12	1	Copper chaperone CopZ	ATCC29328_04300	ATCC53516_07000	07T609_03770	08T492_03470	09T408_03920	09T494_04610	12T272_02410	12T273_07350	12T306_05520	CCUG54800_04750	T151023_04110	T160124_03810
12	12	1	Copper-sensing transcriptional repressor CsoR	ATCC29328_04310	ATCC53516_06990	07T609_03780	08T492_03480	09T408_03930	09T494_04620	12T272_02420	12T273_07360	12T306_05530	CCUG54800_04760	T151023_04120	T160124_03820
12	12	1	Ribosome-associated factor Y	ATCC29328_04320	ATCC53516_06980	07T609_03790	08T492_03490	09T408_03940	09T494_04630	12T272_02430	12T273_07370	12T306_05540	CCUG54800_04770	T151023_04130	T160124_03830
10	10	1	hypothetical protein	ATCC29328_04330	ATCC53516_06970	07T609_03800	08T492_03500	09T408_03950	09T494_04640	*	12T273_07380	12T306_05550	CCUG54800_04780	*	T160124_03840
12	12	1	Deoxyribose-phosphate aldolase 1	ATCC29328_04350	ATCC53516_06950	07T609_03820	08T492_03520	09T408_03970	09T494_04660	12T272_02450	12T273_07400	12T306_05570	CCUG54800_04800	T151023_04150	T160124_03860
12	12	1	Histidinol-phosphatase	ATCC29328_04360	ATCC53516_06940	07T609_03830	08T492_03530	09T408_03980	09T494_04670	12T272_02460	12T273_07410	12T306_05580	CCUG54800_04810	T151023_04160	T160124_03870
12	12	1	Putative 2-hydroxyacid dehydrogenase	ATCC29328_04370	ATCC53516_06930	07T609_03840	08T492_03540	09T408_03990	09T494_04680	12T272_02470	12T273_07420	12T306_05590	CCUG54800_04820	T151023_04170	T160124_03880
11	11	1	hypothetical protein	ATCC29328_04380	ATCC53516_06920	07T609_03850	08T492_03550	09T408_04000	09T494_04690	12T272_02480	12T273_07430	*	CCUG54800_04830	T151023_04180	T160124_03890
11	11	1	Capsular polysaccharide type 8 biosynthesis pr	ATCC29328_04390	ATCC53516_06910	07T609_03860	08T492_03560	09T408_04010	09T494_04700	12T272_02490	12T273_07440	*	CCUG54800_04840	T151023_04190	T160124_03900
4	8	0.5	DNA-binding transcriptional dual regulator Crp	ATCC29328_04400,ATCC2932	*	07T609_09200,07T609_	08T492_09060,08T492_03	09T408_04020,09T408_	*	*	*	*	*	*	*
12	12	1	Hydroxylamine reductase	ATCC29328_04410	ATCC53516_06890	07T609_03880	08T492_03580	09T408_04030	09T494_04720	12T272_02510	12T273_07460	12T306_05620	CCUG54800_04860	T151023_04210	T160124_03920
12	12	1	ribosomal-protein-L7/L12-serine acetyltransfe	ATCC29328_04420	ATCC53516_06850	07T609_03890	08T492_03590	09T408_04040	09T494_04730	12T272_02520	12T273_07470	12T306_05630	CCUG54800_04870	T151023_04220	T160124_03930
12	12	1	Multidrug resistance protein MdtK	ATCC29328_04430	ATCC53516_06840	07T609_03900	08T492_03600	09T408_04050	09T494_04740	12T272_02530	12T273_07480	12T306_05640	CCUG54800_04880	T151023_04230	T160124_03940

12	12	1	Glutamate--tRNA ligase	ATCC29328_04440	ATCC53516_06830	07T609_03910	08T492_03610	09T408_04060	09T494_04750	12T272_02540	12T273_07490	12T306_05650	CCUG54800_04890	T151023_04240	T160124_03950
12	12	1	Cysteine--tRNA ligase	ATCC29328_04450	ATCC53516_06820	07T609_03920	08T492_03620	09T408_04070	09T494_04760	12T272_02550	12T273_07500	12T306_05660	CCUG54800_04900	T151023_04250	T160124_03960
12	12	1	Thymidylate synthase ThyX	ATCC29328_04460	ATCC53516_06810	07T609_03930	08T492_03630	09T408_04080	09T494_04770	12T272_02560	12T273_07510	12T306_05670	CCUG54800_04910	T151023_04260	T160124_03970
12	24	0.167	Fatty acid-binding protein	ATCC29328_04470,ATCC2932	ATCC53516_13520,ATCC5351	07T609_05740,07T609_	08T492_05590,08T492_03	09T408_05850,09T408_	09T494_06770,09T494_	12T272_04540,12T272_	12T273_09410,12T273_	12T306_07630,12T306_	CCUG54800_04920,CCUG548	T151023_06110,T151023_	T160124_06030,T160124_03980
12	12	1	Multidrug export protein MepA	ATCC29328_04480	ATCC53516_06790	07T609_03950	08T492_03650	09T408_04100	09T494_04790	12T272_02580	12T273_07530	12T306_05690	CCUG54800_04940	T151023_04280	T160124_03990
12	24	0.167	Proton/sodium-glutamate symport protein	ATCC29328_04490,ATCC2932	ATCC53516_05820,ATCC5351	07T609_04850,07T609_	08T492_04540,08T492_03	09T408_04970,09T408_	09T494_05730,09T494_	12T272_03530,12T272_	12T273_08430,12T273_	12T306_06620,12T306_	CCUG54800_04950,CCUG548	T151023_05190,T151023_	T160124_04970,T160124_04000
12	12	1	Choloylglycine hydrolase	ATCC29328_04500	ATCC53516_06770	07T609_03970	08T492_03670	09T408_04120	09T494_04810	12T272_02600	12T273_07550	12T306_05710	CCUG54800_04960	T151023_04310	T160124_04010
12	12	1	UDP-N-acetylmuramate--L-alanine ligase	ATCC29328_04510	ATCC53516_06760	07T609_03980	08T492_03680	09T408_04130	09T494_04820	12T272_02610	12T273_07560	12T306_05720	CCUG54800_04970	T151023_04320	T160124_04020
12	12	1	Pyridoxine kinase	ATCC29328_04520	ATCC53516_06750	07T609_03990	08T492_03690	09T408_04140	09T494_04830	12T272_02620	12T273_07570	12T306_05730	CCUG54800_04980	T151023_04330	T160124_04030
12	24	0.167	hypothetical protein	ATCC29328_04530,ATCC2932	ATCC53516_16980,ATCC5351	07T609_09020,07T609_	08T492_08890,08T492_03	09T408_09210,09T408_	09T494_10170,09T494_	12T272_07920,12T272_	12T273_12800,12T273_	12T306_10950,12T306_	CCUG54800_04990,CCUG548	T151023_09430,T151023_	T160124_09410,T160124_04040
12	12	1	Glycine/sarcosine/betaine reductase complex	ATCC29328_04540	ATCC53516_06730	07T609_04010	08T492_03710	09T408_04160	09T494_04850	12T272_02640	12T273_07590	12T306_05750	CCUG54800_05000	T151023_04350	T160124_04050
12	12	1	Glycine/sarcosine/betaine reductase complex	ATCC29328_04550	ATCC53516_06720	07T609_04020	08T492_03720	09T408_04170	09T494_04860	12T272_02650	12T273_07600	12T306_05760	CCUG54800_05010	T151023_04360	T160124_04060
12	19	0.123	Glycine reductase complex component B subu	ATCC29328_04560,ATCC2932	ATCC53516_06710	07T609_16550,07T609_	08T492_03730	09T408_16380,09T408_	09T494_04870	12T272_16670,12T272_	12T273_00400,12T273_	12T306_17290,12T306_	CCUG54800_05020	T151023_04370,T151023_	T160124_04070
3	6	0.5	Glycine reductase complex component B subu	ATCC29328_04570,ATCC2932	*	07T609_04040,07T609_	*	*	*	12T272_15170,12T272_	*	*	*	*	*
11	18	1	Glycine/sarcosine/betaine reductase complex	ATCC29328_04580,ATCC2932	ATCC53516_06690,ATCC5351	*	08T492_03750	09T408_04260	09T494_04950	12T272_02750,12T272_	12T273_07700,12T273_	12T306_05860,12T306_	CCUG54800_05100	T151023_04460,T151023_	T160124_04160,T160124_04090
12	24	1	Glycine/sarcosine/betaine reductase complex	ATCC29328_04590,ATCC2932	ATCC53516_06680,ATCC5351	07T609_04130,07T609_	08T492_03830,08T492_03	09T408_04270,09T408_	09T494_04960,09T494_	12T272_02760,12T272_	12T273_07710,12T273_	12T306_05870,12T306_	CCUG54800_05110,CCUG548	T151023_04470,T151023_	T160124_04170,T160124_04100
12	19	0.153	Glycine reductase complex component B subu	ATCC29328_04600,ATCC2932	ATCC53516_06670	07T609_16570,07T609_	08T492_03770	09T408_16400,09T408_	09T494_04900	12T272_16690,12T272_	12T273_00420,12T273_	12T306_17310,12T306_	CCUG54800_05050	T151023_04410,T151023_	T160124_04110
12	24	0.167	Thioredoxin	ATCC29328_04610,ATCC2932	ATCC53516_02880,ATCC5351	07T609_11490,07T609_	08T492_11410,08T492_03	09T408_10990,09T408_	09T494_12130,09T494_	12T272_09650,12T272_	12T273_15090,12T273_	12T306_13090,12T306_	CCUG54800_05060,CCUG548	T151023_10990,T151023_	T160124_11110,T160124_04120
12	12	1	hypothetical protein	ATCC29328_04620	ATCC53516_06650	07T609_04090	08T492_03790	09T408_04230	09T494_04920	12T272_02720	12T273_07670	12T306_05830	CCUG54800_05070	T151023_04430	T160124_04130
12	12	1	L-seryl-tRNA(Sec) selenium transferase	ATCC29328_04630	ATCC53516_06640	07T609_04100	08T492_03800	09T408_04240	09T494_04930	12T272_02730	12T273_07680	12T306_05840	CCUG54800_05080	T151023_04440	T160124_04140
12	12	1	Sodium:alanine symporter family protein	ATCC29328_04640	ATCC53516_06630	07T609_04110	08T492_03810	09T408_04250	09T494_04940	12T272_02740	12T273_07690	12T306_05850	CCUG54800_05090	T151023_04450	T160124_04150
12	12	1	Thioredoxin reductase	ATCC29328_04670	ATCC53516_06600	07T609_04140	08T492_03840	09T408_04280	09T494_04970	12T272_02770	12T273_07720	12T306_05880	CCUG54800_05120	T151023_04480	T160124_04180
12	12	1	Amino-acid carrier protein AlsT	ATCC29328_04680	ATCC53516_06590	07T609_04150	08T492_03850	09T408_04290	09T494_04980	12T272_02780	12T273_07730	12T306_05890	CCUG54800_05130	T151023_04490	T160124_04190
12	12	1	hypothetical protein	ATCC29328_04700	ATCC53516_06550	07T609_04170	08T492_03870	09T408_04310	09T494_05010	12T272_02810	12T273_07750	12T306_05920	CCUG54800_05160	T151023_04520	T160124_04210
12	12	1	Exopolyphosphatase	ATCC29328_04710	ATCC53516_06540	07T609_04180	08T492_03880	09T408_04320	09T494_05020	12T272_02820	12T273_07760	12T306_05930	CCUG54800_05170	T151023_04530	T160124_04220
12	12	1	Polyphosphate kinase	ATCC29328_04720	ATCC53516_06530	07T609_04190	08T492_03890	09T408_04330	09T494_05030	12T272_02830	12T273_07770	12T306_05940	CCUG54800_05180	T151023_04540	T160124_04230
12	12	1	antiporter inner membrane protein	ATCC29328_04730	ATCC53516_06520	07T609_04200	08T492_03900	09T408_04340	09T494_05040	12T272_02840	12T273_07780	12T306_05950	CCUG54800_05190	T151023_04550	T160124_04240
12	24	0.167	ATP-dependent zinc metalloprotease FtsH 4	ATCC29328_04740,ATCC2932	ATCC53516_17580,ATCC5351	07T609_09990,07T609_	08T492_09990,08T492_03	09T408_09480,09T408_	09T494_10340,09T494_	12T272_08130,12T272_	12T273_13590,12T273_	12T306_11700,12T306_	CCUG54800_05210,CCUG548	T151023_09600,T151023_	T160124_09620,T160124_04260
12	12	1	Organic hydroperoxide resistance transcriptio	ATCC29328_04750	ATCC53516_06490	07T609_04220	08T492_03920	09T408_04370	09T494_05070	12T272_02870	12T273_07810	12T306_05970	CCUG54800_05220	T151023_04580	T160124_04270
12	12	1	hypothetical protein	ATCC29328_04760	ATCC53516_06480	07T609_04230	08T492_03930	09T408_04380	09T494_05080	12T272_02880	12T273_07820	12T306_05980	CCUG54800_05230	T151023_04590	T160124_04280
12	12	1	H(+)/Cl(-) exchange transporter ClcA	ATCC29328_04770	ATCC53516_06470	07T609_04240	08T492_03940	09T408_04390	09T494_05090	12T272_02890	12T273_07830	12T306_05990	CCUG54800_05240	T151023_04600	T160124_04290
12	12	1	hypothetical protein	ATCC29328_04780	ATCC53516_06460	07T609_04250	08T492_03950	09T408_04400	09T494_05100	12T272_02900	12T273_07840	12T306_06000	CCUG54800_05250	T151023_04610	T160124_04300
12	12	1	SirA-like protein	ATCC29328_04790	ATCC53516_06450	07T609_04260	08T492_03960	09T408_04410	09T494_05110	12T272_02910	12T273_07850	12T306_06010	CCUG54800_05260	T151023_04620	T160124_04310
12	12	1	hypothetical protein	ATCC29328_04800	ATCC53516_06440	07T609_04270	08T492_03970	09T408_04420	09T494_05120	12T272_02920	12T273_07860	12T306_06020	CCUG54800_05270	T151023_04630	T160124_04320
12	12	1	putative MscS family protein YkuT	ATCC29328_04810	ATCC53516_06430	07T609_04280	08T492_03980	09T408_04430	09T494_05130	12T272_02930	12T273_07870	12T306_06030	CCUG54800_05280	T151023_04640	T160124_04330
12	12	1	hypothetical protein	ATCC29328_04820	ATCC53516_06420	07T609_04290	08T492_03990	09T408_04440	09T494_05140	12T272_02940	12T273_07880	12T306_06040	CCUG54800_05290	T151023_04650	T160124_04340
12	12	1	GtrA-like protein	ATCC29328_04830	ATCC53516_06410	07T609_04300	08T492_04000	09T408_04450	09T494_05150	12T272_02950	12T273_07890	12T306_06050	CCUG54800_05300	T151023_04660	T160124_04350
12	12	1	hypothetical protein	ATCC29328_04840	ATCC53516_06400	07T609_04310	08T492_04010	09T408_04460	09T494_05160	12T272_02960	12T273_07900	12T306_06060	CCUG54800_05310	T151023_04670	T160124_04360
12	12	1	Acetyltransferase YpeA	ATCC29328_04850	ATCC53516_06390	07T609_04320	08T492_04020	09T408_04470	09T494_05180	12T272_02980	12T273_07920	12T306_06080	CCUG54800_05330	T151023_04690	T160124_04380
12	12	1	Arginine deiminase	ATCC29328_04860	ATCC53516_06380	07T609_04330	08T492_04030	09T408_04480	09T494_05190	12T272_02990	12T273_07930	12T306_06090	CCUG54800_05340	T151023_04700	T160124_04390
12	12	1	Ornithine carbamoyltransferase	ATCC29328_04870	ATCC53516_06370	07T609_04340	08T492_04040	09T408_04490	09T494_05200	12T272_03000	12T273_07940	12T306_06100	CCUG54800_05350	T151023_04710	T160124_04400
12	12	1	O-Antigen ligase	ATCC29328_04880	ATCC53516_06360	07T609_04350	08T492_04050	09T408_04500	09T494_05210	12T272_03010	12T273_07950	12T306_06110	CCUG54800_05360	T151023_04720	T160124_04410
12	12	1	Putative metallo-hydrolase YycJ	ATCC29328_04890	ATCC53516_06350	07T609_04360	08T492_04060	09T408_04510	09T494_05220	12T272_03020	12T273_07960	12T306_06120	CCUG54800_05370	T151023_04730	T160124_04420
12	12	1	tRNA 2-thiocytidine biosynthesis protein TtcA	ATCC29328_04900	ATCC53516_06340	07T609_04370	08T492_04070	09T408_04520	09T494_05230	12T272_03030	12T273_07970	12T306_06140	CCUG54800_05380	T151023_04740	T160124_04430
12	12	1	Nitric oxide reductase	ATCC29328_04910	ATCC53516_06330	07T609_04380	08T492_04080	09T408_04530	09T494_05240	12T272_03040	12T273_07980	12T306_06150	CCUG54800_05390	T151023_04750	T160124_04440
12	12	1	Formate--tetrahydrofolate ligase	ATCC29328_04920	ATCC53516_06320	07T609_04390	08T492_04090	09T408_04540	09T494_05250	12T272_03050	12T273_07990	12T306_06160	CCUG54800_05400	T151023_04760	T160124_04450
12	12	1	Aminomethyltransferase	ATCC29328_04930	ATCC53516_06310	07T609_04400	08T492_04100	09T408_04550	09T494_05260	12T272_03060	12T273_08000	12T306_06170	CCUG54800_05410	T151023_04770	T160124_04460
12	12	1	Glycine cleavage system H protein	ATCC29328_04940	ATCC53516_06300	07T609_04410	08T492_04110	09T408_04560	09T494_05270	12T272_03070	12T273_08010	12T306_06180	CCUG54800_05420	T151023_04780	T160124_04470
12	12	1	putative glycine dehydrogenase (decarboxylati	ATCC29328_04950	ATCC53516_06290	07T609_04420	08T492_04120	09T408_04570	09T494_05280	12T272_03080	12T273_08020	12T306_06190	CCUG54800_05430	T151023_04790	T160124_04480
12	12	1	putative glycine dehydrogenase (decarboxylati	ATCC29328_04960	ATCC53516_06280	07T609_04430	08T492_04130	09T408_04580	09T494_05290	12T272_03090	12T273_08030	12T306_06200	CCUG54800_05440	T151023_04800	T160124_04490
12	24	0.147	Dihydrolipoyl dehydrogenase	ATCC29328_04970,ATCC2932	ATCC53516_05780,ATCC5351	07T609_04880,07T609_	08T492_04570,08T492_04	09T408_05000,09T408_	09T494_05760,09T494_	12T272_03560,12T272_	12T273_08460,12T273_	12T306_06650,12T306_	CCUG54800_05450,CCUG548	T151023_05220,T151023_	T160124_05000,T160124_04500
12	12	1	Serine protease Do-like HtrB	ATCC29328_04980	ATCC53516_06260	07T609_04460	08T492_04150	09T408_04600	09T494_05310	12T272_03110	12T273_08050	12T306_06220	CCUG54800_05460	T151023_04820	T160124_04510
12	12	1	hypothetical protein	ATCC29328_04990	ATCC53516_06250	07T609_04470	08T492_04160	09T408_04610	09T494_05320	12T272_03120	12T273_08060	12T306_06230	CCUG54800_05470	T151023_04830	T160124_04520
12	12	1	Vegetative protein 296	ATCC29328_05000	ATCC53516_06240	07T609_04480	08T492_04170	09T408_04620	09T494_05330	12T272_03130	12T273_08070	12T306_06240	CCUG54800_05480	T151023_04840	T160124_04530
12	12	1	FeS cluster assembly protein SufB	ATCC29328_05010	ATCC53516_06230	07T609_04490	08T492_04180	09T408_04630	09T494_05340	12T272_03140	12T273_08080	12T306_06250	CCUG54800_05490	T151023_04850	T160124_04540
12	12	1	FeS cluster assembly protein SufB	ATCC29328_05020	ATCC53516_06220	07T609_04500	08T492_04190	09T408_04640	09T494_05350	12T272_03150	12T273_08090	12T306_06260	CCUG54800_05500	T151023_04860	T160124_04550
12	24	0.167	putative cysteine desulfurase	ATCC29328_05030,ATCC2932	ATCC53516_11440,ATCC5351	07T609_16660,07T609_	08T492_17400,08T492_04	09T408_16490,09T408_	09T494_01170,09T494_	12T272_16860,12T272_	12T273_00590,12T273_	12T306_17470,12T306_	CCUG54800_05510,CCUG548	T151023_00210,T151023_	T160124_16430,T160124_04560
12	12	1	NifU-like protein	ATCC29328_05040	ATCC53516_06200	07T609_04520	08T492_04210	09T408_04660	09T494_05370	12T272_03170	12T273_08110	12T306_06280	CCUG54800_05520	T151023_04880	T160124_04570
3	3	1	cofactor-independent phosphoglycerate muta	ATCC29328_05050	ATCC53516_06190	*	*	*	*	*	*	*	*	*	T160124_04580
3	3	1	Citrate carrier	ATCC29328_05060	ATCC53516_06180	*	*	*	*	*	*	*	*	*	T160124_04590
3	3	1	HTH-type transcriptional activator CmpR	ATCC29328_05070	ATCC53516_06170	*	*	*	*	*	*	*	*	*	T160124_04610
12	12	1	hypothetical protein	ATCC29328_05080	ATCC53516_06160	07T609_04530	08T492_04220	09T408_04670	09T494_05380	12T272_03180	12T273_08120	12T306_06290	CCUG54800_05530	T151023_04890	T160124_04640
12	12	1	Thiamine biosynthesis lipoprotein ApbE precu	ATCC29328_05090	ATCC53516_06150	07T609_04540	08T492_04230	09T408_04680	09T494_05390	12T272_03190	12T273_08130	12T306_06300	CCUG54800_05540	T151023_04900	T160124_04650
12	12	1	Bifunctional protein GlmU	ATCC29328_05100	ATCC53516_06140	07T609_04550	08T492_04240	09T408_04690	09T494_05400	12T272_03200	12T273_08140	12T306_06310	CCUG54800_05550	T151023_04910	T160124_04660
12	12	1	Peptidyl-tRNA hydrolase	ATCC29328_05110	ATCC53516_06130	07T609_04560	08T492_04250	09T408_04700	09T494_05410	12T272_03210	12T273_08150	12T306_06320	CCUG54800_05560	T151023_04920	T160124_04670
12	12	1	Transcription-repair-coupling factor	ATCC29328_05120	ATCC53516_06120	07T609_04570	08T492_04260	09T408_04710	09T494_05420	12T272_03220	12T273_08160	12T306_06330	CCUG54800_05570	T151023_04930	T160124_04680
12	12	1	Foldase protein PrsA 1 precursor	ATCC29328_05130	ATCC53516_06110	07T609_04580	08T492_04270	09T408_04720	09T494_05430	12T272_03230	12T273_08170	12T306_06340	CCUG54800_05580	T151023_04940	T160124_04690
12	12	1	DNA-binding protein HU	ATCC29328_05140	ATCC53516_06090	07T609_04590	08T492_04280	09T408_04730	09T494_05440	12T272_03240	12T273_08180	12T306_06350	CCUG54800_05590	T151023_04950	T160124_04700
12	12	1	hypothetical protein	ATCC29328_05150	ATCC53516_06080	07T609_04600	08T492_04290	09T408_04740	09T494_05450	12T272_03250	12T273_08190	12T306_06360	CCUG54800_05600	T151023_04960	T160124_04710
12	12	1	hypothetical protein	ATCC29328_05160	ATCC53516_06070	07T609_04610	08T492_04300	09T408_04750	09T494_05460	12T272_03260	12T273_08200	12T306_06370	CCUG54800_05610	T151023_04970	T160124_04720
12	12	1	Pyrophosphate--fructose 6-phosphate 1-phos	ATCC29328_05170	ATCC53516_06060	07T609_04620	08T492_04310	09T408_04760	09T494_05470	12T272_03270	12T273_08210	12T306_06380	CCUG54800_05620	T151023_04980	T160124_04730
12	12	1	putative metallophosphoesterase	ATCC29328_05190	ATCC53516_06040	07T609_04640	08T492_04330	09T408_04780	09T494_05490	12T272_03290	12T273_08230	12T306_06400	CCUG54800_05640	T151023_05000	T160124_04750
12	12	1	hypothetical protein	ATCC29328_05200	ATCC53516_06030	07T609_04650	08T492_04340	09T408_04790	09T494_05500	12T272_03300	12T273_08240	12T306_06410	CCUG54800_05650	T151023_05010	T160124_04760
12	12	1	ABC-2 family transporter protein	ATCC29328_05210	ATCC53516_06020	07T609_04660	08T492_04350	09T408_04800	09T494_05510	12T272_03310	12T273_08250	12T306_06420	CCUG54800_05670	T151023_05020	T160124_04770
12	23	0.163	SkfA peptide export ATP-binding protein SkfE	ATCC29328_05220,ATCC2932	ATCC53516_16910,ATCC5351	07T609_12030,07T609_	08T492_12080,08T492_04	09T408_09160,09T408_	09T494_10110,09T494_	12T272_07870,12T272_	12T273_08260	12T306_13620,12T306_	CCUG54800_05680,CCUG548	T151023_11520,T151023_	T160124_11800,T160124_04780
12	12	1	30S ribosomal protein S12	ATCC29328_05230	ATCC53516_06000	07T609_04680	08T492_04370	09T408_04820	09T494_05530	12T272_03330	12T273_08270	12T306_06440	CCUG54800_05690	T151023_05040	T160124_04790
12	12	1	30S ribosomal protein S7	ATCC29328_05240	ATCC53516_05990	07T609_04690	08T492_04380	09T408_04830	09T494_05540	12T272_03340	12T273_08280	12T306_06450	CCUG54800_05700	T151023_05050	T160124_04800
12	24	0.167	Elongation factor G	ATCC29328_05250,ATCC2932	ATCC53516_04990,ATCC5351	07T609_13310,07T609_	08T492_14410,08T492_04	09T408_12740,09T408_	09T494_14560,09T494_	12T272_11540,12T272_	12T273_05870,12T273_	12T306_14980,12T306_	CCUG54800_05710,CCUG548	T151023_12800,T151023_	T160124_02490,T160124_04810
11	11	1	hypothetical protein	ATCC29328_05270	ATCC53516_05930	07T609_04720	08T492_04410	09T408_04860	09T494_05600	12T272_03400	12T273_08320	12T306_06510	CCUG54800_05730	*	T160124_04860
12	12	1	ABC-2 family transporter protein	ATCC29328_05280	ATCC53516_05920	07T609_04730	08T492_04420	09T408_04870	09T494_05610	12T272_03410	12T273_08330	12T306_06520	CCUG54800_05740	T151023_05070	T160124_04870
12	12	1	ABC-type transporter ATP-binding protein Ecs	ATCC29328_05290	ATCC53516_05910	07T609_04740	08T492_04430	09T408_04880	09T494_05620	12T272_03420	12T273_08340	12T306_06530	CCUG54800_05750	T151023_05080	T160124_04880
12	12	1	Membrane transport protein	ATCC29328_05300	ATCC53516_05900	07T609_04750	08T492_04440	09T408_04890	09T494_05630	12T272_03430	12T273_08350	12T306_06540	CCUG54800_05760	T151023_05090	T160124_04890
12	12	1	Citrate-sodium symporter	ATCC29328_05310	ATCC53516_05890	07T609_04760	08T492_04450	09T408_04900	09T494_05640	12T272_03440	12T273_08360	12T306_06550	CCUG54800_05770	T151023_05100	T160124_04900
12	12	1	NAD-dependent malic enzyme	ATCC29328_05320	ATCC53516_05880	07T609_04770	08T492_04460	09T408_04910	09T494_05650	12T272_03450	12T273_08370	12T306_06560	CCUG54800_05780	T151023_05110	T160124_04910
12	14	0.143	Peptidase T	ATCC29328_05330	ATCC53516_05870	07T609_04780	08T492_04470	09T408_15890,09T408_	09T494_05660	12T272_03460	12T273_08380	12T306_06570	CCUG54800_05790,CCUG548	T151023_05120	T160124_04920
12	12	1	Inner membrane transporter YcaM	ATCC29328_05340	ATCC53516_05860	07T609_04790	08T492_04480	09T408_04930	09T494_05670	12T272_03470	12T273_08390	12T306_06580	CCUG54800_05800	T151023_05130	T160124_04930
12	12	1	putative glycosyl transferase	ATCC29328_05360	ATCC53516_13410	07T609_04810	08T492_04500	09T408_05760	09T494_05690	12T272_03490	12T273_09310	12T306_07540	CCUG54800_06780	T151023_05150	T160124_05930
12	12	1	Nicotinate dehydrogenase FAD-subunit	ATCC29328_05370	ATCC53516_05850	07T609_04820	08T492_04510	09T408_04940	09T494_05700	12T272_03500	12T273_08400	12T306_06590	CCUG54800_05810	T151023_05160	T160124_04940
12	12	1	Carbon monoxide dehydrogenase small chain	ATCC29328_05380	ATCC53516_05840	07T609_04830	08T492_04520	09T408_04950	09T494_05710	12T272_03510	12T273_08410	12T306_06600	CCUG54800_05820	T151023_05170	T160124_04950
12	12	1	Xanthine dehydrogenase molybdenum-bindin	ATCC29328_05390	ATCC53516_05830	07T609_04840	08T492_04530	09T408_04960	09T494_05720	12T272_03520	12T273_08420	12T306_06610	CCUG54800_05830	T151023_05180	T160124_04960
12	24	0.167	Signal peptidase I P	ATCC29328_05410,ATCC2932	ATCC53516_15590,ATCC5351	07T609_07810,07T609_	08T492_07660,08T492_04	09T408_07890,09T408_	09T494_08850,09T494_	12T272_06610,12T272_	12T273_11510,12T273_	12T306_09720,12T306_	CCUG54800_05850,CCUG548	T151023_08190,T151023_	T160124_08110,T160124_04980
12	12	1	Fructose-1,6-bisphosphatase class 3	ATCC29328_05420	ATCC53516_05790	07T609_04870	08T492_04560	09T408_04990	09T494_05750	12T272_03550	12T273_08450	12T306_06640	CCUG54800_05860	T151023_05210	T160124_04990
12	12	1	Zinc transporter ZupT	ATCC29328_05440	ATCC53516_05750	07T609_04890	08T492_04580	09T408_05010	09T494_05770	12T272_03570	12T273_08470	12T306_06660	CCUG54800_05900	T151023_05230	T160124_05010
12	12	1	DNA polymerase III subunit tau	ATCC29328_05450	ATCC53516_05740	07T609_04900	08T492_04590	09T408_05020	09T494_05780	12T272_03580	12T273_08480	12T306_06670	CCUG54800_05910	T151023_05240	T160124_05020
12	12	1	Nucleoid-associated protein	ATCC29328_05460	ATCC53516_05730	07T609_04910	08T492_04600	09T408_05030	09T494_05790	12T272_03590	12T273_08490	12T306_06680	CCUG54800_05920	T151023_05250	T160124_05030
12	12	1	Recombination protein RecR	ATCC29328_05470	ATCC53516_05720	07T609_04920	08T492_04610	09T408_05040	09T494_05800	12T272_03600	12T273_08500	12T306_06690	CCUG54800_05930	T151023_05260	T160124_05040
12	12	1	Inner membrane protein YgaZ	ATCC29328_05480	ATCC53516_05710	07T609_04930	08T492_04620	09T408_05050	09T494_05810	12T272_03610	12T273_08510	12T306_06700	CCUG54800_05940	T151023_05270	T160124_05050
12	12	1	Branched-chain amino acid transport protein (	ATCC29328_05490	ATCC53516_05700	07T609_04940	08T492_04630	09T408_05060	09T494_05820	12T272_03620	12T273_08520	12T306_06710	CCUG54800_05950	T151023_05280	T160124_05060
12	12	1	Putative GTP cyclohydrolase 1 type 2	ATCC29328_05500	ATCC53516_05680	07T609_04960	08T492_04650	09T408_05070	09T494_05840	12T272_03640	12T273_08530	12T306_06740	CCUG54800_05960	T151023_05300	T160124_05070
12	12	1	Glutaredoxin-3	ATCC29328_05510	ATCC53516_05670	07T609_04970	08T492_04660	09T408_05080	09T494_05850	12T272_03650	12T273_08540	12T306_06750	CCUG54800_05970	T151023_05310	T160124_05080
12	24	0.167	D-amino acid dehydrogenase	ATCC29328_05520,ATCC2932	ATCC53516_16060,ATCC5351	07T609_08260,07T609_	08T492_08110,08T492_04	09T408_08340,09T408_	09T494_09300,09T494_	12T272_07060,12T272_	12T273_11950,12T273_	12T306_10180,12T306_	CCUG54800_05980,CCUG548	T151023_08640,T151023_	T160124_08550,T160124_05100
12	12	1	hypothetical protein	ATCC29328_05530	ATCC53516_05630	07T609_04990	08T492_04680	09T408_05100	09T494_05870	12T272_03670	12T273_08560	12T306_06770	CCUG54800_05990	T151023_05330	T160124_05110
12	12	1	hypothetical protein	ATCC29328_05550	ATCC53516_05610	07T609_05010	08T492_04700	09T408_05120	09T494_05890	12T272_03690	12T273_08580	12T306_06790	CCUG54800_06010	T151023_05350	T160124_05130
12	12	1	Glycerol-3-phosphate dehydrogenase [NAD(P)	ATCC29328_05560	ATCC53516_05600	07T609_05020	08T492_04710	09T408_05130	09T494_05900	12T272_03700	12T273_08590	12T306_06800	CCUG54800_06020	T151023_05360	T160124_05140
12	12	1	putative DNA-binding protein	ATCC29328_05570	ATCC53516_05590	07T609_05030	08T492_04720	09T408_05140	09T494_05910	12T272_03710	12T273_08600	12T306_06810	CCUG54800_06030	T151023_05370	T160124_05150
12	12	1	Signal recognition particle protein	ATCC29328_05580	ATCC53516_05580	07T609_05040	08T492_04730	09T408_05150	09T494_05920	12T272_03720	12T273_08610	12T306_06820	CCUG54800_06040	T151023_05380	T160124_05160
12	12	1	30S ribosomal protein S16	ATCC29328_05590	ATCC53516_05570	07T609_05050	08T492_04740	09T408_05160	09T494_05930	12T272_03730	12T273_08620	12T306_06830	CCUG54800_06050	T151023_05390	T160124_05170
12	12	1	hypothetical protein	ATCC29328_05600	ATCC53516_05560	07T609_05060	08T492_04750	09T408_05170	09T494_05940	12T272_03740	12T273_08630	12T306_06840	CCUG54800_06060	T151023_05400	T160124_05180
12	12	1	Ribosome maturation factor RimM	ATCC29328_05610	ATCC53516_05550	07T609_05070	08T492_04760	09T408_05180	09T494_05950	12T272_03750	12T273_08640	12T306_06850	CCUG54800_06070	T151023_05410	T160124_05190
12	12	1	tRNA (guanine-N(1)-)-methyltransferase	ATCC29328_05620	ATCC53516_05540	07T609_05080	08T492_04770	09T408_05190	09T494_05960	12T272_03760	12T273_08650	12T306_06860	CCUG54800_06080	T151023_05420	T160124_05200
12	12	1	50S ribosomal protein L19	ATCC29328_05630	ATCC53516_05530	07T609_05090	08T492_04780	09T408_05200	09T494_05970	12T272_03770	12T273_08660	12T306_06870	CCUG54800_06090	T151023_05430	T160124_05210
12	12	1	Ribosome biogenesis GTPase A	ATCC29328_05640	ATCC53516_05520	07T609_05100	08T492_04790	09T408_05210	09T494_05980	12T272_03780	12T273_08670	12T306_06880	CCUG54800_06100	T151023_05440	T160124_05220
12	12	1	Ribonuclease HII	ATCC29328_05650	ATCC53516_05510	07T609_05110	08T492_04800	09T408_05220	09T494_05990	12T272_03790	12T273_08680	12T306_06890	CCUG54800_06110	T151023_05450	T160124_05230
12	12	1	hypothetical protein	ATCC29328_05660	ATCC53516_05500	07T609_05120	08T492_04810	09T408_05230	09T494_06000	12T272_03800	12T273_08690	12T306_06900	CCUG54800_06120	T151023_05460	T160124_05240
12	12	1	Molybdopterin adenylyltransferase	ATCC29328_05670	ATCC53516_05490	07T609_05130	08T492_04820	09T408_05240	09T494_06010	12T272_03810	12T273_08700	12T306_06910	CCUG54800_06130	T151023_05470	T160124_05250
12	12	1	Elongation factor P	ATCC29328_05680	ATCC53516_05480	07T609_05140	08T492_04830	09T408_05250	09T494_06020	12T272_03820	12T273_08710	12T306_06920	CCUG54800_06140	T151023_05480	T160124_05260
12	12	1	50S ribosomal protein L33	ATCC29328_05690	ATCC53516_05470	07T609_05150	08T492_04840	09T408_05260	09T494_06030	12T272_03830	12T273_08720	12T306_06930	CCUG54800_06150	T151023_05490	T160124_05270
12	12	1	preprotein translocase subunit SecE	ATCC29328_05700	ATCC53516_05460	07T609_05160	08T492_04850	09T408_05270	09T494_06040	12T272_03840	12T273_08730	12T306_06940	CCUG54800_06160	T151023_05500	T160124_05280
12	12	1	hypothetical protein	ATCC29328_05710	ATCC53516_05450	07T609_05170	08T492_04860	09T408_05280	09T494_06050	12T272_03850	12T273_08740	12T306_06950	CCUG54800_06170	T151023_05510	T160124_05290
12	12	1	50S ribosomal protein L11	ATCC29328_05720	ATCC53516_05440	07T609_05180	08T492_04870	09T408_05290	09T494_06060	12T272_03860	12T273_08750	12T306_06960	CCUG54800_06180	T151023_05520	T160124_05300
12	12	1	50S ribosomal protein L1	ATCC29328_05730	ATCC53516_05430	07T609_05190	08T492_04880	09T408_05300	09T494_06070	12T272_03870	12T273_08760	12T306_06970	CCUG54800_06190	T151023_05530	T160124_05310
12	12	1	hypothetical protein	ATCC29328_05740	ATCC53516_05420	07T609_05200	08T492_04890	09T408_05310	09T494_06080	12T272_03880	12T273_08770	12T306_06980	CCUG54800_06200	T151023_05540	T160124_05320
12	12	1	50S ribosomal protein L10	ATCC29328_05750	ATCC53516_05410	07T609_05210	08T492_04900	09T408_05320	09T494_06090	12T272_03890	12T273_08780	12T306_06990	CCUG54800_06210	T151023_05550	T160124_05330
12	12	1	50S ribosomal protein L7/L12	ATCC29328_05760	ATCC53516_05400	07T609_05220	08T492_04910	09T408_05330	09T494_06100	12T272_03900	12T273_08790	12T306_07000	CCUG54800_06220	T151023_05560	T160124_05340
12	12	1	Methionine--tRNA ligase	ATCC29328_05770	ATCC53516_05390	07T609_05230	08T492_04920	09T408_05340	09T494_06110	12T272_03910	12T273_08800	12T306_07010	CCUG54800_06230	T151023_05570	T160124_05350
12	12	1	putative deoxyribonuclease YcfH	ATCC29328_05780	ATCC53516_05380	07T609_05240	08T492_04930	09T408_05350	09T494_06120	12T272_03920	12T273_08810	12T306_07020	CCUG54800_06240	T151023_05580	T160124_05360
12	12	1	Ribonuclease M5	ATCC29328_05790	ATCC53516_05370	07T609_05250	08T492_04940	09T408_05360	09T494_06130	12T272_03930	12T273_08820	12T306_07030	CCUG54800_06250	T151023_05590	T160124_05370
12	13	0.141	Ribosomal RNA small subunit methyltransfera	ATCC29328_05800	ATCC53516_05360	07T609_05260	08T492_04950	09T408_05370	09T494_06140	12T272_03940	12T273_08830	12T306_07040	CCUG54800_06260,CCUG548	T151023_05600	T160124_05380
12	12	1	Glutaredoxin-3	ATCC29328_05810	ATCC53516_05350	07T609_05270	08T492_04960	09T408_05380	09T494_06150	12T272_03950	12T273_08840	12T306_07050	CCUG54800_06270	T151023_05610	T160124_05390
12	12	1	hypothetical protein	ATCC29328_05820	ATCC53516_05340	07T609_05280	08T492_04970	09T408_05390	09T494_06160	12T272_03960	12T273_08850	12T306_07060	CCUG54800_06280	T151023_05620	T160124_05400
12	12	1	Magnesium transporter MgtE	ATCC29328_05830	ATCC53516_05330	07T609_05290	08T492_04980	09T408_05400	09T494_06170	12T272_03970	12T273_08860	12T306_07070	CCUG54800_06290	T151023_05630	T160124_05410
12	12	1	hypothetical protein	ATCC29328_05840	ATCC53516_05320	07T609_05300	08T492_04990	09T408_05410	09T494_06180	12T272_03980	12T273_08870	12T306_07080	CCUG54800_06300	T151023_05640	T160124_05420
12	12	1	Glutaredoxin	ATCC29328_05850	ATCC53516_05310	07T609_05310	08T492_05000	09T408_05420	09T494_06190	12T272_03990	12T273_08880	12T306_07090	CCUG54800_06310	T151023_05650	T160124_05430
12	12	1	4-diphosphocytidyl-2-C-methyl-D-erythritol ki	ATCC29328_05860	ATCC53516_05300	07T609_05320	08T492_05010	09T408_05430	09T494_06200	12T272_04000	12T273_08890	12T306_07100	CCUG54800_06320	T151023_05660	T160124_05440
12	12	1	Glutamate racemase	ATCC29328_05870	ATCC53516_05290	07T609_05330	08T492_05020	09T408_05440	09T494_06210	12T272_04010	12T273_08900	12T306_07110	CCUG54800_06330	T151023_05670	T160124_05450
12	12	1	putative M18 family aminopeptidase 1	ATCC29328_05880	ATCC53516_05280	07T609_05340	08T492_05030	09T408_05450	09T494_06220	12T272_04020	12T273_08910	12T306_07120	CCUG54800_06340	T151023_05680	T160124_05460
12	12	1	Fimbrial protein precursor	ATCC29328_06110	ATCC53516_13140	07T609_05400	08T492_05250	09T408_05490	09T494_06420	12T272_04190	12T273_09040	12T306_07270	CCUG54800_06510	T151023_05760	T160124_05660
12	12	1	hypothetical protein	ATCC29328_06120	ATCC53516_13150	07T609_05410	08T492_05260	09T408_05500	09T494_06430	12T272_04200	12T273_09050	12T306_07280	CCUG54800_06520	T151023_05770	T160124_05670
12	12	1	hypothetical protein	ATCC29328_06130	ATCC53516_13160	07T609_05420	08T492_05270	09T408_05510	09T494_06440	12T272_04210	12T273_09060	12T306_07290	CCUG54800_06530	T151023_05780	T160124_05680
12	24	0.167	Cysteine desulfurase	ATCC29328_06140,ATCC2932	ATCC53516_11670,ATCC5351	07T609_16360,07T609_	08T492_17160,08T492_05	09T408_16200,09T408_	09T494_00910,09T494_	12T272_16480,12T272_	12T273_00220,12T273_	12T306_17090,12T306_	CCUG54800_06540,CCUG548	T151023_15790,T151023_	T160124_16230,T160124_05690
12	12	1	putative tRNA sulfurtransferase	ATCC29328_06150	ATCC53516_13180	07T609_05440	08T492_05290	09T408_05530	09T494_06460	12T272_04230	12T273_09080	12T306_07310	CCUG54800_06550	T151023_05800	T160124_05700
12	17	0.148	LemA family protein	ATCC29328_06160	ATCC53516_13190	07T609_05450	08T492_05300	09T408_05540	09T494_06470	12T272_18170,12T272_	12T273_01210,12T273_	12T306_07320	CCUG54800_06560,CCUG548	T151023_00750,T151023_	T160124_00020,T160124_05710
12	12	1	hypothetical protein	ATCC29328_06170	ATCC53516_13200	07T609_05460	08T492_05310	09T408_05550	09T494_06480	12T272_04250	12T273_09100	12T306_07330	CCUG54800_06570	T151023_05820	T160124_05720
12	12	1	HTH-type transcriptional regulator PuuR	ATCC29328_06180	ATCC53516_13210	07T609_05470	08T492_05320	09T408_05560	09T494_06490	12T272_04260	12T273_09110	12T306_07340	CCUG54800_06580	T151023_05830	T160124_05730
12	20	0.155	Spermidine/putrescine import ATP-binding pr	ATCC29328_06190	ATCC53516_04170,ATCC5351	07T609_05480	08T492_05330	09T408_05570	09T494_13390,09T494_	12T272_10890,12T272_	12T273_16360,12T273_	12T306_14350,12T306_	CCUG54800_06590,CCUG548	T151023_12240,T151023_	T160124_12540,T160124_05740
12	12	1	Spermidine/putrescine transport system perm	ATCC29328_06200	ATCC53516_13230	07T609_05490	08T492_05340	09T408_05580	09T494_06510	12T272_04280	12T273_09130	12T306_07360	CCUG54800_06600	T151023_05850	T160124_05750
12	12	1	Inner membrane ABC transporter permease p	ATCC29328_06210	ATCC53516_13240	07T609_05500	08T492_05350	09T408_05590	09T494_06520	12T272_04290	12T273_09140	12T306_07370	CCUG54800_06610	T151023_05860	T160124_05760
12	12	1	Spermidine/putrescine-binding periplasmic pr	ATCC29328_06220	ATCC53516_13250	07T609_05510	08T492_05360	09T408_05600	09T494_06530	12T272_04300	12T273_09150	12T306_07380	CCUG54800_06620	T151023_05870	T160124_05770
12	12	1	Undecaprenyl-diphosphatase	ATCC29328_06230	ATCC53516_13260	07T609_05520	08T492_05370	09T408_05610	09T494_06540	12T272_04310	12T273_09160	12T306_07390	CCUG54800_06630	T151023_05880	T160124_05780
12	24	0.167	ATP-dependent DNA helicase PcrA	ATCC29328_06240,ATCC2932	ATCC53516_14170,ATCC5351	07T609_06390,07T609_	08T492_06240,08T492_05	09T408_06460,09T408_	09T494_07430,09T494_	12T272_05190,12T272_	12T273_10060,12T273_	12T306_08260,12T306_	CCUG54800_06640,CCUG548	T151023_06770,T151023_	T160124_06690,T160124_05790
12	12	1	DNA ligase	ATCC29328_06250	ATCC53516_13280	07T609_05540	08T492_05390	09T408_05630	09T494_06560	12T272_04330	12T273_09180	12T306_07410	CCUG54800_06650	T151023_05900	T160124_05800
12	12	1	Aspartyl/glutamyl-tRNA(Asn/Gln) amidotransf	ATCC29328_06260	ATCC53516_13290	07T609_05550	08T492_05400	09T408_05640	09T494_06570	12T272_04340	12T273_09190	12T306_07420	CCUG54800_06660	T151023_05910	T160124_05810
12	12	1	Glutamyl-tRNA(Gln) amidotransferase subunit	ATCC29328_06270	ATCC53516_13300	07T609_05560	08T492_05410	09T408_05650	09T494_06580	12T272_04350	12T273_09200	12T306_07430	CCUG54800_06670	T151023_05920	T160124_05820
12	12	1	Aspartyl/glutamyl-tRNA(Asn/Gln) amidotransf	ATCC29328_06280	ATCC53516_13310	07T609_05570	08T492_05420	09T408_05660	09T494_06590	12T272_04360	12T273_09210	12T306_07440	CCUG54800_06680	T151023_05930	T160124_05830
12	12	1	Non-canonical purine NTP pyrophosphatase	ATCC29328_06290	ATCC53516_13320	07T609_05580	08T492_05430	09T408_05670	09T494_06600	12T272_04370	12T273_09220	12T306_07450	CCUG54800_06690	T151023_05940	T160124_05840
12	24	0.147	hypothetical protein	ATCC29328_06300,ATCC2932	ATCC53516_03630,ATCC5351	07T609_12220,07T609_	08T492_12270,08T492_05	09T408_11660,09T408_	09T494_12850,09T494_	12T272_10340,12T272_	12T273_15820,12T273_	12T306_13830,12T306_	CCUG54800_06700,CCUG548	T151023_11710,T151023_	T160124_12000,T160124_05850
12	12	1	Bifunctional protein FolD protein	ATCC29328_06310	ATCC53516_13340	07T609_05600	08T492_05450	09T408_05690	09T494_06620	12T272_04390	12T273_09240	12T306_07470	CCUG54800_06710	T151023_05960	T160124_05860
12	12	1	Purine catabolism protein PucB	ATCC29328_06320	ATCC53516_13350	07T609_05610	08T492_05460	09T408_05700	09T494_06630	12T272_04400	12T273_09250	12T306_07480	CCUG54800_06720	T151023_05970	T160124_05870
12	12	1	hypothetical protein	ATCC29328_06330	ATCC53516_13360	07T609_05620	08T492_05470	09T408_05710	09T494_06640	12T272_04410	12T273_09260	12T306_07490	CCUG54800_06730	T151023_05980	T160124_05880
12	12	1	putative xanthine dehydrogenase subunit A	ATCC29328_06340	ATCC53516_13370	07T609_05630	08T492_05480	09T408_05720	09T494_06650	12T272_04420	12T273_09270	12T306_07500	CCUG54800_06740	T151023_05990	T160124_05890
12	24	0.167	Zinc uptake regulation protein	ATCC29328_06350,ATCC2932	ATCC53516_13950,ATCC5351	07T609_06170,07T609_	08T492_06020,08T492_05	09T408_06260,09T408_	09T494_07200,09T494_	12T272_04970,12T272_	12T273_09840,12T273_	12T306_08040,12T306_	CCUG54800_06750,CCUG548	T151023_06550,T151023_	T160124_06460,T160124_05900
12	21	0.152	UDP-4-amino-4-deoxy-L-arabinose--oxoglutar	ATCC29328_06360,ATCC2932	ATCC53516_13420,ATCC5351	07T609_05670,07T609_	08T492_05520,08T492_05	09T408_05770	09T494_06670	12T272_04460	12T273_09320,12T273_	12T306_07550,12T306_	CCUG54800_06790,CCUG548	T151023_06010,T151023_	T160124_05940,T160124_05910
12	15	0.145	UDP-N-acetylgalactosamine-undecaprenyl-ph	ATCC29328_06370	ATCC53516_13400	07T609_05660	08T492_05510	09T408_14460,09T408_	09T494_17310,09T494_	12T272_04450,12T272_	12T273_09300	12T306_07530	CCUG54800_06770	T151023_06020	T160124_05920
12	12	1	UDP-2-acetamido-3-amino-2,3-dideoxy-D-gluc	ATCC29328_06390	ATCC53516_13430	07T609_05680	08T492_05530	09T408_05780	09T494_06700	12T272_04470	12T273_09330	12T306_07560	CCUG54800_06800	T151023_06040	T160124_05950
12	12	1	Inositol 2-dehydrogenase	ATCC29328_06410	ATCC53516_13450	07T609_05700	08T492_05550	09T408_05800	09T494_06720	12T272_04490	12T273_09350	12T306_07580	CCUG54800_06820	T151023_06060	T160124_05970
12	12	1	hypothetical protein	ATCC29328_06420	ATCC53516_13470	07T609_05710	08T492_05560	09T408_05820	09T494_06740	12T272_04510	12T273_09380	12T306_07600	CCUG54800_06840	T151023_06080	T160124_06000
12	12	1	hypothetical protein	ATCC29328_06430	ATCC53516_13480	07T609_05720	08T492_05570	09T408_05830	09T494_06750	12T272_04520	12T273_09390	12T306_07610	CCUG54800_06850	T151023_06090	T160124_06010
12	12	1	Alkaline shock protein 23	ATCC29328_06440	ATCC53516_13490	07T609_05730	08T492_05580	09T408_05840	09T494_06760	12T272_04530	12T273_09400	12T306_07620	CCUG54800_06860	T151023_06100	T160124_06020
12	12	1	Signal-transduction histidine kinase senX3	ATCC29328_06460	ATCC53516_13530	07T609_05750	08T492_05600	09T408_05860	09T494_06780	12T272_04550	12T273_09420	12T306_07640	CCUG54800_06880	T151023_06120	T160124_06040
12	12	1	Transcriptional regulatory protein WalR	ATCC29328_06470	ATCC53516_13540	07T609_05760	08T492_05610	09T408_05870	09T494_06790	12T272_04560	12T273_09430	12T306_07650	CCUG54800_06890	T151023_06130	T160124_06050
12	12	1	hypothetical protein	ATCC29328_06480	ATCC53516_13550	07T609_05770	08T492_05620	09T408_05880	09T494_06800	12T272_04570	12T273_09440	12T306_07660	CCUG54800_06900	T151023_06140	T160124_06060
12	12	1	Phosphate transport system permease protein	ATCC29328_06500	ATCC53516_13570	07T609_05790	08T492_05640	09T408_05900	09T494_06820	12T272_04590	12T273_09460	12T306_07680	CCUG54800_06920	T151023_06160	T160124_06080
12	12	1	Phosphate-binding protein PstS 2 precursor	ATCC29328_06520	ATCC53516_13590	07T609_05810	08T492_05660	09T408_05920	09T494_06840	12T272_04610	12T273_09480	12T306_07700	CCUG54800_06940	T151023_06190	T160124_06100
12	12	1	L-asparaginase 1	ATCC29328_06530	ATCC53516_13600	07T609_05820	08T492_05670	09T408_05930	09T494_06850	12T272_04620	12T273_09490	12T306_07710	CCUG54800_06950	T151023_06200	T160124_06110
12	12	1	hypothetical protein	ATCC29328_06540	ATCC53516_13610	07T609_05830	08T492_05680	09T408_05940	09T494_06860	12T272_04630	12T273_09500	12T306_07720	CCUG54800_06960	T151023_06210	T160124_06120
12	12	1	YceG-like family protein	ATCC29328_06550	ATCC53516_13620	07T609_05840	08T492_05690	09T408_05950	09T494_06870	12T272_04640	12T273_09510	12T306_07730	CCUG54800_06970	T151023_06220	T160124_06130
12	24	0.167	Ribosomal large subunit pseudouridine syntha	ATCC29328_06560,ATCC2932	ATCC53516_14070,ATCC5351	07T609_06290,07T609_	08T492_06140,08T492_05	09T408_06380,09T408_	09T494_07330,09T494_	12T272_05090,12T272_	12T273_09960,12T273_	12T306_08160,12T306_	CCUG54800_07430,CCUG548	T151023_06230,T151023_	T160124_06590,T160124_06140
12	12	1	Uridine kinase	ATCC29328_06570	ATCC53516_13640	07T609_05860	08T492_05710	09T408_05970	09T494_06890	12T272_04660	12T273_09530	12T306_07750	CCUG54800_06990	T151023_06240	T160124_06150
12	12	1	ATP-dependent helicase/deoxyribonuclease su	ATCC29328_06580	ATCC53516_13650	07T609_05870	08T492_05720	09T408_05980	09T494_06900	12T272_04670	12T273_09540	12T306_07760	CCUG54800_07000	T151023_06250	T160124_06160
12	12	1	ATP-dependent helicase/nuclease subunit A	ATCC29328_06590	ATCC53516_13660	07T609_05880	08T492_05730	09T408_05990	09T494_06910	12T272_04680	12T273_09550	12T306_07770	CCUG54800_07010	T151023_06260	T160124_06170
12	13	0.141	Alkaline phosphatase synthesis transcriptional	ATCC29328_06600	ATCC53516_13670	07T609_05890	08T492_05740,08T492_13	09T408_06000	09T494_06920	12T272_04690	12T273_09560	12T306_07780	CCUG54800_07020	T151023_06270	T160124_06180
12	12	1	Alkaline phosphatase synthesis sensor protein	ATCC29328_06610	ATCC53516_13680	07T609_05900	08T492_05750	09T408_06010	09T494_06930	12T272_04700	12T273_09570	12T306_07790	CCUG54800_07030	T151023_06280	T160124_06190
12	12	1	hypothetical protein	ATCC29328_06620	ATCC53516_13690	07T609_05910	08T492_05760	09T408_06020	09T494_06940	12T272_04710	12T273_09580	12T306_07800	CCUG54800_07040	T151023_06290	T160124_06200
12	12	1	hypothetical protein	ATCC29328_06650	ATCC53516_13720	07T609_05940	08T492_05790	09T408_06030	09T494_06970	12T272_04740	12T273_09610	12T306_07810	CCUG54800_07080	T151023_06320	T160124_06230
12	12	1	haloacid dehalogenase-like hydrolase	ATCC29328_06660	ATCC53516_13730	07T609_05950	08T492_05800	09T408_06040	09T494_06980	12T272_04750	12T273_09620	12T306_07820	CCUG54800_07090	T151023_06330	T160124_06240
12	12	1	Sortase family protein	ATCC29328_06670	ATCC53516_13740	07T609_05960	08T492_05810	09T408_06050	09T494_06990	12T272_04760	12T273_09630	12T306_07830	CCUG54800_07100	T151023_06340	T160124_06250
12	12	1	cell division protein MraZ	ATCC29328_06680	ATCC53516_13750	07T609_05970	08T492_05820	09T408_06060	09T494_07000	12T272_04770	12T273_09640	12T306_07840	CCUG54800_07110	T151023_06350	T160124_06260
12	12	1	Ribosomal RNA small subunit methyltransfera	ATCC29328_06690	ATCC53516_13760	07T609_05980	08T492_05830	09T408_06070	09T494_07010	12T272_04780	12T273_09650	12T306_07850	CCUG54800_07120	T151023_06360	T160124_06270
12	12	1	Cell division protein FtsL	ATCC29328_06700	ATCC53516_13770	07T609_05990	08T492_05840	09T408_06080	09T494_07020	12T272_04790	12T273_09660	12T306_07860	CCUG54800_07130	T151023_06370	T160124_06280
12	12	1	Stage V sporulation protein D	ATCC29328_06710	ATCC53516_13780	07T609_06000	08T492_05850	09T408_06090	09T494_07030	12T272_04800	12T273_09670	12T306_07870	CCUG54800_07140	T151023_06380	T160124_06290
12	12	1	Phospho-N-acetylmuramoyl-pentapeptide-tra	ATCC29328_06720	ATCC53516_13790	07T609_06010	08T492_05860	09T408_06100	09T494_07040	12T272_04810	12T273_09680	12T306_07880	CCUG54800_07150	T151023_06390	T160124_06300
12	12	1	UDP-N-acetylmuramoylalanine--D-glutamate li	ATCC29328_06730	ATCC53516_13800	07T609_06020	08T492_05870	09T408_06110	09T494_07050	12T272_04820	12T273_09690	12T306_07890	CCUG54800_07160	T151023_06400	T160124_06310
12	24	0.167	Lipid II flippase FtsW	ATCC29328_06740,ATCC2932	ATCC53516_14910,ATCC5351	07T609_07130,07T609_	08T492_06980,08T492_05	09T408_07200,09T408_	09T494_08170,09T494_	12T272_05930,12T272_	12T273_10820,12T273_	12T306_09040,12T306_	CCUG54800_07170,CCUG548	T151023_07510,T151023_	T160124_07430,T160124_06320
12	12	1	UDP-N-acetylglucosamine--N-acetylmuramyl-(	ATCC29328_06750	ATCC53516_13820	07T609_06040	08T492_05890	09T408_06130	09T494_07070	12T272_04840	12T273_09710	12T306_07910	CCUG54800_07180	T151023_06420	T160124_06330
12	12	1	cell division protein FtsQ	ATCC29328_06760	ATCC53516_13830	07T609_06050	08T492_05900	09T408_06140	09T494_07080	12T272_04850	12T273_09720	12T306_07920	CCUG54800_07190	T151023_06430	T160124_06340
12	12	1	Cell division protein FtsZ	ATCC29328_06770	ATCC53516_13840	07T609_06060	08T492_05910	09T408_06150	09T494_07090	12T272_04860	12T273_09730	12T306_07930	CCUG54800_07200	T151023_06440	T160124_06350
12	12	1	Transcriptional repressor NrdR	ATCC29328_06780	ATCC53516_13850	07T609_06070	08T492_05920	09T408_06160	09T494_07100	12T272_04870	12T273_09740	12T306_07940	CCUG54800_07210	T151023_06450	T160124_06360
12	12	1	Replication-associated recombination protein	ATCC29328_06790	ATCC53516_13860	07T609_06080	08T492_05930	09T408_06170	09T494_07110	12T272_04880	12T273_09750	12T306_07950	CCUG54800_07220	T151023_06460	T160124_06370
12	12	1	Asparagine--tRNA ligase	ATCC29328_06800	ATCC53516_13870	07T609_06090	08T492_05940	09T408_06180	09T494_07120	12T272_04890	12T273_09760	12T306_07960	CCUG54800_07230	T151023_06470	T160124_06380
12	12	1	Leucine--tRNA ligase	ATCC29328_06810	ATCC53516_13880	07T609_06100	08T492_05950	09T408_06190	09T494_07130	12T272_04900	12T273_09770	12T306_07970	CCUG54800_07240	T151023_06480	T160124_06390
12	12	1	HTH-type transcriptional regulator CymR	ATCC29328_06820	ATCC53516_13890	07T609_06110	08T492_05960	09T408_06200	09T494_07140	12T272_04910	12T273_09780	12T306_07980	CCUG54800_07250	T151023_06490	T160124_06400
12	12	1	AI-2 transport protein TqsA	ATCC29328_06830	ATCC53516_13900	07T609_06120	08T492_05970	09T408_06210	09T494_07150	12T272_04920	12T273_09790	12T306_07990	CCUG54800_07260	T151023_06500	T160124_06410
12	12	1	Alanine--tRNA ligase	ATCC29328_06840	ATCC53516_13910	07T609_06130	08T492_05980	09T408_06220	09T494_07160	12T272_04930	12T273_09800	12T306_08000	CCUG54800_07270	T151023_06510	T160124_06420
12	12	1	hypothetical protein	ATCC29328_06850	ATCC53516_13920	07T609_06140	08T492_05990	09T408_06230	09T494_07170	12T272_04940	12T273_09810	12T306_08010	CCUG54800_07280	T151023_06520	T160124_06430
12	12	1	Putative Holliday junction resolvase	ATCC29328_06860	ATCC53516_13930	07T609_06150	08T492_06000	09T408_06240	09T494_07180	12T272_04950	12T273_09820	12T306_08020	CCUG54800_07290	T151023_06530	T160124_06440
12	12	1	hypothetical protein	ATCC29328_06870	ATCC53516_13940	07T609_06160	08T492_06010	09T408_06250	09T494_07190	12T272_04960	12T273_09830	12T306_08030	CCUG54800_07300	T151023_06540	T160124_06450
12	12	1	Ribonuclease J 1	ATCC29328_06890	ATCC53516_13960	07T609_06180	08T492_06030	09T408_06270	09T494_07210	12T272_04980	12T273_09850	12T306_08050	CCUG54800_07320	T151023_06560	T160124_06470
12	12	1	hypothetical protein	ATCC29328_06900	ATCC53516_13970	07T609_06190	08T492_06040	09T408_06280	09T494_07220	12T272_04990	12T273_09860	12T306_08060	CCUG54800_07330	T151023_06570	T160124_06480
12	12	1	Putative O-methyltransferase/MSMEI_4947	ATCC29328_06910	ATCC53516_13980	07T609_06200	08T492_06050	09T408_06290	09T494_07230	12T272_05000	12T273_09870	12T306_08070	CCUG54800_07340	T151023_06580	T160124_06490
12	12	1	putative protease YhbU precursor	ATCC29328_06920	ATCC53516_13990	07T609_06210	08T492_06060	09T408_06300	09T494_07240	12T272_05010	12T273_09880	12T306_08080	CCUG54800_07350	T151023_06590	T160124_06500

12	12	1	HlyD family secretion protein	ATCC29328_06930	ATCC53516_14000	07T609_06220	08T492_06070	09T408_06310	09T494_07250	12T272_05020	12T273_09890	12T306_08090	CCUG54800_07360	T151023_06600	T160124_06510
12	12	1	hypothetical protein	ATCC29328_06940	ATCC53516_14010	07T609_06230	08T492_06080	09T408_06320	09T494_07260	12T272_05030	12T273_09900	12T306_08100	CCUG54800_07370	T151023_06610	T160124_06520
12	12	1	Cell division protein SepF	ATCC29328_06950	ATCC53516_14020	07T609_06240	08T492_06090	09T408_06330	09T494_07270	12T272_05040	12T273_09910	12T306_08110	CCUG54800_07380	T151023_06620	T160124_06530
12	12	1	YGGT family protein	ATCC29328_06960	ATCC53516_14030	07T609_06250	08T492_06100	09T408_06340	09T494_07280	12T272_05050	12T273_09920	12T306_08120	CCUG54800_07390	T151023_06630	T160124_06540
12	12	1	hypothetical protein	ATCC29328_06970	ATCC53516_14040	07T609_06260	08T492_06110	09T408_06350	09T494_07290	12T272_05060	12T273_09930	12T306_08130	CCUG54800_07400	T151023_06640	T160124_06550
12	12	1	Lipoprotein signal peptidase	ATCC29328_06980	ATCC53516_14050	07T609_06270	08T492_06120	09T408_06360	09T494_07310	12T272_05070	12T273_09940	12T306_08140	CCUG54800_07410	T151023_06650	T160124_06570
12	12	1	Uracil-DNA glycosylase	ATCC29328_06990	ATCC53516_14060	07T609_06280	08T492_06130	09T408_06370	09T494_07320	12T272_05080	12T273_09950	12T306_08150	CCUG54800_07420	T151023_06660	T160124_06580
12	13	0.141	Putative endo-beta-N-acetylglucosaminidase p	ATCC29328_07030,ATCC2932ł	ATCC53516_14100	07T609_06320	08T492_06170	09T408_06410	09T494_07360	12T272_05120	12T273_09990	12T306_08190	CCUG54800_07460	T151023_06700	T160124_06620
12	14	0.143	Thermonuclease precursor	ATCC29328_07040	ATCC53516_14110	07T609_06330	08T492_06180	09T408_06420	09T494_02010,09T494_	12T272_05130	12T273_10000	12T306_08200	CCUG54800_07470,CCUG548	T151023_06710	T160124_06630
12	12	1	hypothetical protein	ATCC29328_07080	ATCC53516_14150	07T609_06370	08T492_06220	09T408_06430	09T494_07410	12T272_05170	12T273_10040	12T306_08240	CCUG54800_07510	T151023_06750	T160124_06670
12	12	1	Alkyl hydroperoxide reductase subunit C	ATCC29328_07090	ATCC53516_14160	07T609_06380	08T492_06230	09T408_06440	09T494_07420	12T272_05180	12T273_10050	12T306_08250	CCUG54800_07520	T151023_06760	T160124_06680
12	12	1	Nitroreductase family protein	ATCC29328_07110	ATCC53516_14180	07T609_06400	08T492_06250	09T408_06470	09T494_07440	12T272_05200	12T273_10070	12T306_08270	CCUG54800_07540	T151023_06780	T160124_06700
12	12	1	Molybdate-binding periplasmic protein precur	ATCC29328_07120	ATCC53516_14190	07T609_06410	08T492_06260	09T408_06480	09T494_07450	12T272_05210	12T273_10080	12T306_08280	CCUG54800_07550	T151023_06790	T160124_06710
12	12	1	Molybdenum transport system permease prot	ATCC29328_07130	ATCC53516_14200	07T609_06420	08T492_06270	09T408_06490	09T494_07460	12T272_05220	12T273_10090	12T306_08290	CCUG54800_07560	T151023_06800	T160124_06720
12	12	1	Fe(3+) ions import ATP-binding protein FbpC 2	ATCC29328_07140	ATCC53516_14210	07T609_06430	08T492_06280	09T408_06500	09T494_07470	12T272_05230	12T273_10100	12T306_08300	CCUG54800_07570	T151023_06810	T160124_06730
12	12	1	Ribonuclease R	ATCC29328_07150	ATCC53516_14220	07T609_06440	08T492_06290	09T408_06510	09T494_07480	12T272_05240	12T273_10110	12T306_08310	CCUG54800_07580	T151023_06820	T160124_06740
12	12	1	SsrA-binding protein	ATCC29328_07160	ATCC53516_14230	07T609_06450	08T492_06300	09T408_06520	09T494_07490	12T272_05250	12T273_10120	12T306_08320	CCUG54800_07590	T151023_06830	T160124_06750
12	12	1	hypothetical protein	ATCC29328_07170	ATCC53516_14240	07T609_06460	08T492_06310	09T408_06530	09T494_07500	12T272_05260	12T273_10130	12T306_08330	CCUG54800_07600	T151023_06840	T160124_06760
12	12	1	hypothetical protein	ATCC29328_07180	ATCC53516_14250	07T609_06470	08T492_06320	09T408_06540	09T494_07510	12T272_05270	12T273_10140	12T306_08340	CCUG54800_07610	T151023_06850	T160124_06770
12	12	1	hypothetical protein	ATCC29328_07190	ATCC53516_14260	07T609_06480	08T492_06330	09T408_06550	09T494_07520	12T272_05280	12T273_10150	12T306_08350	CCUG54800_07620	T151023_06860	T160124_06780
12	12	1	Guanylate kinase	ATCC29328_07200	ATCC53516_14270	07T609_06490	08T492_06340	09T408_06560	09T494_07530	12T272_05290	12T273_10160	12T306_08360	CCUG54800_07630	T151023_06870	T160124_06790
12	12	1	DNA-directed RNA polymerase subunit omega	ATCC29328_07210	ATCC53516_14280	07T609_06500	08T492_06350	09T408_06570	09T494_07540	12T272_05300	12T273_10170	12T306_08370	CCUG54800_07640	T151023_06880	T160124_06800
12	12	1	Coenzyme A biosynthesis bifunctional protein	ATCC29328_07220	ATCC53516_14290	07T609_06510	08T492_06360	09T408_06580	09T494_07550	12T272_05310	12T273_10180	12T306_08380	CCUG54800_07650	T151023_06890	T160124_06810
12	12	1	Primosomal protein N'	ATCC29328_07230	ATCC53516_14300	07T609_06520	08T492_06370	09T408_06590	09T494_07560	12T272_05320	12T273_10190	12T306_08390	CCUG54800_07660	T151023_06900	T160124_06820
12	12	1	Peptide deformylase 1	ATCC29328_07240	ATCC53516_14310	07T609_06530	08T492_06380	09T408_06600	09T494_07570	12T272_05330	12T273_10200	12T306_08400	CCUG54800_07670	T151023_06910	T160124_06830
12	12	1	Methionyl-tRNA formyltransferase	ATCC29328_07250	ATCC53516_14320	07T609_06540	08T492_06390	09T408_06610	09T494_07580	12T272_05340	12T273_10210	12T306_08410	CCUG54800_07680	T151023_06920	T160124_06840
12	12	1	Putative neutral zinc metallopeptidase	ATCC29328_07260	ATCC53516_14330	07T609_06550	08T492_06400	09T408_06620	09T494_07590	12T272_05350	12T273_10220	12T306_08420	CCUG54800_07690	T151023_06930	T160124_06850
12	12	1	Ribosomal RNA small subunit methyltransfera:	ATCC29328_07270	ATCC53516_14340	07T609_06560	08T492_06410	09T408_06630	09T494_07600	12T272_05360	12T273_10230	12T306_08430	CCUG54800_07700	T151023_06940	T160124_06860
12	12	1	putative dual-specificity RNA methyltransferas	ATCC29328_07280	ATCC53516_14350	07T609_06570	08T492_06420	09T408_06640	09T494_07610	12T272_05370	12T273_10240	12T306_08440	CCUG54800_07710	T151023_06950	T160124_06870
12	24	0.167	Serine/threonine phosphatase stp	ATCC29328_07290,ATCC2932ł	ATCC53516_15150,ATCC5351(	07T609_07370,07T609_(	08T492_07220,08T492_06	09T408_07440,09T408_(	09T494_08410,09T494_	12T272_06170,12T272_	12T273_11070,12T273_	12T306_09280,12T306_	CCUG54800_07720,CCUG548	T151023_07750,T151023_	T160124_07670,T160124_06880
12	12	1	Serine/threonine-protein kinase PrkC	ATCC29328_07300	ATCC53516_14370	07T609_06590	08T492_06440	09T408_06660	09T494_07630	12T272_05390	12T273_10260	12T306_08460	CCUG54800_07730	T151023_06970	T160124_06890
12	12	1	Putative ribosome biogenesis GTPase RsgA	ATCC29328_07310	ATCC53516_14380	07T609_06600	08T492_06450	09T408_06670	09T494_07640	12T272_05400	12T273_10270	12T306_08470	CCUG54800_07740	T151023_06980	T160124_06900
12	12	1	Ribulose-phosphate 3-epimerase	ATCC29328_07320	ATCC53516_14390	07T609_06610	08T492_06460	09T408_06680	09T494_07650	12T272_05410	12T273_10280	12T306_08480	CCUG54800_07750	T151023_06990	T160124_06910
12	12	1	Thiamine transporter ThiT	ATCC29328_07330	ATCC53516_14400	07T609_06620	08T492_06470	09T408_06690	09T494_07660	12T272_05420	12T273_10290	12T306_08490	CCUG54800_07760	T151023_07000	T160124_06920
12	12	1	Thiamine pyrophosphokinase	ATCC29328_07340	ATCC53516_14410	07T609_06630	08T492_06480	09T408_06700	09T494_07670	12T272_05430	12T273_10300	12T306_08500	CCUG54800_07770	T151023_07010	T160124_06930
12	12	1	50S ribosomal protein L28	ATCC29328_07350	ATCC53516_14420	07T609_06640	08T492_06490	09T408_06710	09T494_07680	12T272_05440	12T273_10310	12T306_08510	CCUG54800_07780	T151023_07020	T160124_06940
12	12	1	Alkaline shock protein 23	ATCC29328_07360	ATCC53516_14430	07T609_06650	08T492_06500	09T408_06720	09T494_07690	12T272_05450	12T273_10320	12T306_08520	CCUG54800_07790	T151023_07030	T160124_06950
12	12	1	DAK2 domain protein	ATCC29328_07370	ATCC53516_14440	07T609_06660	08T492_06510	09T408_06730	09T494_07700	12T272_05460	12T273_10330	12T306_08530	CCUG54800_07800	T151023_07040	T160124_06960
12	12	1	ATP-dependent DNA helicase RecG	ATCC29328_07380	ATCC53516_14450	07T609_06670	08T492_06520	09T408_06740	09T494_07710	12T272_05470	12T273_10340	12T306_08540	CCUG54800_07810	T151023_07050	T160124_06970
12	12	1	Ribosomal RNA small subunit methyltransfera:	ATCC29328_07390	ATCC53516_14460	07T609_06680	08T492_06530	09T408_06750	09T494_07720	12T272_05480	12T273_10350	12T306_08550	CCUG54800_07820	T151023_07060	T160124_06980
12	12	1	Phosphopantetheine adenylyltransferase	ATCC29328_07400	ATCC53516_14470	07T609_06690	08T492_06540	09T408_06760	09T494_07730	12T272_05490	12T273_10360	12T306_08560	CCUG54800_07830	T151023_07070	T160124_06990
12	12	1	hypothetical protein	ATCC29328_07410	ATCC53516_14480	07T609_06700	08T492_06550	09T408_06770	09T494_07740	12T272_05500	12T273_10370	12T306_08570	CCUG54800_07840	T151023_07080	T160124_07000
12	12	1	Flavodoxin	ATCC29328_07420	ATCC53516_14490	07T609_06710	08T492_06560	09T408_06780	09T494_07750	12T272_05510	12T273_10380	12T306_08600	CCUG54800_07850	T151023_07090	T160124_07010
12	12	1	hypothetical protein	ATCC29328_07430	ATCC53516_14500	07T609_06720	08T492_06570	09T408_06790	09T494_07760	12T272_05520	12T273_10390	12T306_08610	CCUG54800_07860	T151023_07100	T160124_07020
12	12	1	preprotein translocase subunit SecG	ATCC29328_07440	ATCC53516_14510	07T609_06730	08T492_06580	09T408_06800	09T494_07770	12T272_05530	12T273_10400	12T306_08620	CCUG54800_07870	T151023_07110	T160124_07030
12	12	1	Putative K(+)-stimulated pyrophosphate-energ	ATCC29328_07450	ATCC53516_14520	07T609_06740	08T492_06590	09T408_06810	09T494_07780	12T272_05540	12T273_10410	12T306_08630	CCUG54800_07880	T151023_07120	T160124_07040
12	13	0.141	Magnesium and cobalt efflux protein CorC	ATCC29328_07460	ATCC53516_14530	07T609_06750	08T492_06600	09T408_06820	09T494_07790	12T272_05550	12T273_10420	12T306_08640	CCUG54800_07890	T151023_07130,T151023_	T160124_07050
12	12	1	Methionine aminopeptidase 1	ATCC29328_07470	ATCC53516_14540	07T609_06760	08T492_06610	09T408_06830	09T494_07800	12T272_05560	12T273_10430	12T306_08650	CCUG54800_07900	T151023_07140	T160124_07060
12	12	1	hypothetical protein	ATCC29328_07480	ATCC53516_14550	07T609_06770	08T492_06620	09T408_06840	09T494_07810	12T272_05570	12T273_10440	12T306_08660	CCUG54800_07910	T151023_07150	T160124_07070
12	12	1	hypothetical protein	ATCC29328_07490	ATCC53516_14560	07T609_06780	08T492_06630	09T408_06850	09T494_07820	12T272_05580	12T273_10450	12T306_08670	CCUG54800_07920	T151023_07160	T160124_07080
12	12	1	hypothetical protein	ATCC29328_07500	ATCC53516_14570	07T609_06790	08T492_06640	09T408_06860	09T494_07830	12T272_05590	12T273_10460	12T306_08680	CCUG54800_07930	T151023_07170	T160124_07090
12	12	1	hypothetical protein	ATCC29328_07510	ATCC53516_14580	07T609_06800	08T492_06650	09T408_06870	09T494_07840	12T272_05600	12T273_10470	12T306_08690	CCUG54800_07940	T151023_07180	T160124_07100
12	12	1	hypothetical protein	ATCC29328_07550	ATCC53516_14620	07T609_06840	08T492_06690	09T408_06910	09T494_07880	12T272_05640	12T273_10510	12T306_08730	CCUG54800_07980	T151023_07220	T160124_07140
12	12	1	Histidine--tRNA ligase	ATCC29328_07560	ATCC53516_14630	07T609_06850	08T492_06700	09T408_06920	09T494_07890	12T272_05650	12T273_10520	12T306_08740	CCUG54800_07990	T151023_07230	T160124_07150
12	12	1	Threonine--tRNA ligase 2	ATCC29328_07570	ATCC53516_14640	07T609_06860	08T492_06710	09T408_06930	09T494_07900	12T272_05660	12T273_10530	12T306_08750	CCUG54800_08000	T151023_07240	T160124_07160
12	12	1	Phosphatidylglycerol lysyltransferase	ATCC29328_07600	ATCC53516_14670	07T609_06890	08T492_06740	09T408_06960	09T494_07930	12T272_05690	12T273_10560	12T306_08780	CCUG54800_08030	T151023_07270	T160124_07190
12	12	1	Translation initiation factor IF-3	ATCC29328_07610	ATCC53516_14680	07T609_06900	08T492_06750	09T408_06970	09T494_07940	12T272_05700	12T273_10570	12T306_08790	CCUG54800_08040	T151023_07280	T160124_07200
12	12	1	50S ribosomal protein L35	ATCC29328_07620	ATCC53516_14690	07T609_06910	08T492_06760	09T408_06980	09T494_07950	12T272_05710	12T273_10580	12T306_08800	CCUG54800_08050	T151023_07290	T160124_07210
12	12	1	50S ribosomal protein L20	ATCC29328_07630	ATCC53516_14700	07T609_06920	08T492_06770	09T408_06990	09T494_07960	12T272_05720	12T273_10590	12T306_08810	CCUG54800_08060	T151023_07300	T160124_07220
12	12	1	GIY-YIG nuclease superfamily protein	ATCC29328_07640	ATCC53516_14710	07T609_06930	08T492_06780	09T408_07000	09T494_07970	12T272_05730	12T273_10600	12T306_08820	CCUG54800_08070	T151023_07310	T160124_07230
12	12	1	Regulatory protein RecX	ATCC29328_07650	ATCC53516_14720	07T609_06940	08T492_06790	09T408_07010	09T494_07980	12T272_05740	12T273_10610	12T306_08830	CCUG54800_08080	T151023_07320	T160124_07240
5	5	0.4	hypothetical protein	ATCC29328_07660	*	*	08T492_06800	09T408_07020	09T494_07990	12T272_05750	*	*	*	*	*
12	12	1	hypothetical protein	ATCC29328_07670	ATCC53516_14740	07T609_06960	08T492_06810	09T408_07030	09T494_08000	12T272_05760	12T273_10630	12T306_08850	CCUG54800_08100	T151023_07340	T160124_07260
12	12	1	putative metallophosphoesterase YhaO	ATCC29328_07680	ATCC53516_14750	07T609_06970	08T492_06820	09T408_07040	09T494_08010	12T272_05770	12T273_10640	12T306_08860	CCUG54800_08110	T151023_07350	T160124_07270
12	12	1	chromosome segregation protein	ATCC29328_07690	ATCC53516_14760	07T609_06980	08T492_06830	09T408_07050	09T494_08020	12T272_05780	12T273_10650	12T306_08870	CCUG54800_08120	T151023_07360	T160124_07280
12	12	1	3',5'-cyclic adenosine monophosphate phosph	ATCC29328_07700	ATCC53516_14770	07T609_06990	08T492_06840	09T408_07060	09T494_08030	12T272_05790	12T273_10660	12T306_08880	CCUG54800_08130	T151023_07370	T160124_07290
12	12	1	CYTH domain protein	ATCC29328_07710	ATCC53516_14780	07T609_07000	08T492_06850	09T408_07070	09T494_08040	12T272_05800	12T273_10670	12T306_08890	CCUG54800_08140	T151023_07380	T160124_07300
12	12	1	DNA topoisomerase 1	ATCC29328_07720	ATCC53516_14790	07T609_07010	08T492_06860	09T408_07080	09T494_08050	12T272_05810	12T273_10680	12T306_08900	CCUG54800_08150	T151023_07390	T160124_07310
12	12	1	30S ribosomal protein S2	ATCC29328_07730	ATCC53516_14800	07T609_07020	08T492_06870	09T408_07090	09T494_08060	12T272_05820	12T273_10690	12T306_08910	CCUG54800_08160	T151023_07400	T160124_07320
12	12	1	Elongation factor Ts	ATCC29328_07740	ATCC53516_14810	07T609_07030	08T492_06880	09T408_07100	09T494_08070	12T272_05830	12T273_10700	12T306_08920	CCUG54800_08170	T151023_07410	T160124_07330
12	12	1	Uridylate kinase	ATCC29328_07750	ATCC53516_14820	07T609_07040	08T492_06890	09T408_07110	09T494_08080	12T272_05840	12T273_10710	12T306_08930	CCUG54800_08180	T151023_07420	T160124_07340
12	12	1	Ribosome-recycling factor	ATCC29328_07760	ATCC53516_14830	07T609_07050	08T492_06900	09T408_07120	09T494_08090	12T272_05850	12T273_10720	12T306_08940	CCUG54800_08190	T151023_07430	T160124_07350
12	12	1	Murein DD-endopeptidase MepH precursor	ATCC29328_07770	ATCC53516_14840	07T609_07060	08T492_06910	09T408_07130	09T494_08100	12T272_05860	12T273_10750	12T306_08950	CCUG54800_08200	T151023_07440	T160124_07360
12	12	1	ComE operon protein 1	ATCC29328_07780	ATCC53516_14850	07T609_07070	08T492_06920	09T408_07140	09T494_08110	12T272_05870	12T273_10760	12T306_08980	CCUG54800_08210	T151023_07450	T160124_07370
12	12	1	Selenide, water dikinase	ATCC29328_07790	ATCC53516_14860	07T609_07080	08T492_06930	09T408_07150	09T494_08120	12T272_05880	12T273_10770	12T306_08990	CCUG54800_08220	T151023_07460	T160124_07380
12	12	1	Transcriptional regulatory protein YycF	ATCC29328_07800	ATCC53516_14870	07T609_07090	08T492_06940	09T408_07160	09T494_08130	12T272_05890	12T273_10780	12T306_09000	CCUG54800_08230	T151023_07470	T160124_07390
12	12	1	Putative peptidyl-prolyl cis-trans isomerase	ATCC29328_07810	ATCC53516_14880	07T609_07100	08T492_06950	09T408_07170	09T494_08140	12T272_05900	12T273_10790	12T306_09010	CCUG54800_08240	T151023_07480	T160124_07400
12	12	1	Thymidine kinase	ATCC29328_07820	ATCC53516_14890	07T609_07110	08T492_06960	09T408_07180	09T494_08150	12T272_05910	12T273_10800	12T306_09020	CCUG54800_08250	T151023_07490	T160124_07410
12	12	1	S-ribosylhomocysteine lyase	ATCC29328_07830	ATCC53516_14900	07T609_07120	08T492_06970	09T408_07190	09T494_08160	12T272_05920	12T273_10810	12T306_09030	CCUG54800_08260	T151023_07500	T160124_07420
12	12	1	putative endonuclease 4	ATCC29328_07850	ATCC53516_14920	07T609_07140	08T492_06990	09T408_07210	09T494_08180	12T272_05940	12T273_10840	12T306_09050	CCUG54800_08280	T151023_07520	T160124_07440
12	12	1	Endonuclease III	ATCC29328_07860	ATCC53516_14930	07T609_07150	08T492_07000	09T408_07220	09T494_08190	12T272_05950	12T273_10850	12T306_09060	CCUG54800_08290	T151023_07530	T160124_07450
12	12	1	Aspartate carbamoyltransferase	ATCC29328_07870	ATCC53516_14940	07T609_07160	08T492_07010	09T408_07230	09T494_08200	12T272_05960	12T273_10860	12T306_09070	CCUG54800_08300	T151023_07540	T160124_07460
12	12	1	Aspartate carbamoyltransferase regulatory chı	ATCC29328_07880	ATCC53516_14950	07T609_07170	08T492_07020	09T408_07240	09T494_08210	12T272_05970	12T273_10870	12T306_09080	CCUG54800_08310	T151023_07550	T160124_07470
12	12	1	Dihydroorotase	ATCC29328_07890	ATCC53516_14960	07T609_07180	08T492_07030	09T408_07250	09T494_08220	12T272_05980	12T273_10880	12T306_09090	CCUG54800_08320	T151023_07560	T160124_07480
12	12	1	orotidine 5'-phosphate decarboxylase	ATCC29328_07900	ATCC53516_14970	07T609_07190	08T492_07040	09T408_07260	09T494_08230	12T272_05990	12T273_10890	12T306_09100	CCUG54800_08330	T151023_07570	T160124_07490
12	12	1	Dihydroorotate dehydrogenase B (NAD(+)), ele	ATCC29328_07910	ATCC53516_14980	07T609_07200	08T492_07050	09T408_07270	09T494_08240	12T272_06000	12T273_10900	12T306_09110	CCUG54800_08340	T151023_07580	T160124_07500
12	12	1	Dihydroorotate dehydrogenase B (NAD(+)), cat	ATCC29328_07920	ATCC53516_14990	07T609_07210	08T492_07060	09T408_07280	09T494_08250	12T272_06010	12T273_10910	12T306_09120	CCUG54800_08350	T151023_07590	T160124_07510
12	12	1	Orotate phosphoribosyltransferase	ATCC29328_07930	ATCC53516_15000	07T609_07220	08T492_07070	09T408_07290	09T494_08260	12T272_06020	12T273_10920	12T306_09130	CCUG54800_08360	T151023_07600	T160124_07520
12	12	1	Ditrans,polycis-undecaprenyl-diphosphate syn	ATCC29328_07940	ATCC53516_15010	07T609_07230	08T492_07080	09T408_07300	09T494_08270	12T272_06030	12T273_10930	12T306_09140	CCUG54800_08370	T151023_07610	T160124_07530
12	12	1	Phosphatidate cytidylyltransferase	ATCC29328_07950	ATCC53516_15020	07T609_07240	08T492_07090	09T408_07310	09T494_08280	12T272_06040	12T273_10940	12T306_09150	CCUG54800_08380	T151023_07620	T160124_07540
12	12	1	Regulator of sigma-W protease RasP	ATCC29328_07960	ATCC53516_15030	07T609_07250	08T492_07100	09T408_07320	09T494_08290	12T272_06050	12T273_10950	12T306_09160	CCUG54800_08390	T151023_07630	T160124_07550
12	12	1	4-hydroxy-3-methylbut-2-en-1-yl diphosphate	ATCC29328_07970	ATCC53516_15040	07T609_07260	08T492_07110	09T408_07330	09T494_08300	12T272_06060	12T273_10960	12T306_09170	CCUG54800_08400	T151023_07640	T160124_07560
12	12	1	DNA polymerase III PolC-type	ATCC29328_07980	ATCC53516_15050	07T609_07270	08T492_07120	09T408_07340	09T494_08310	12T272_06070	12T273_10970	12T306_09180	CCUG54800_08410	T151023_07650	T160124_07570
12	12	1	PhoH-like protein	ATCC29328_07990	ATCC53516_15060	07T609_07280	08T492_07130	09T408_07350	09T494_08320	12T272_06080	12T273_10980	12T306_09190	CCUG54800_08420	T151023_07660	T160124_07580
12	12	1	Endoribonuclease YbeY	ATCC29328_08000	ATCC53516_15070	07T609_07290	08T492_07140	09T408_07360	09T494_08330	12T272_06090	12T273_10990	12T306_09200	CCUG54800_08430	T151023_07670	T160124_07590
12	12	1	Undecaprenol kinase	ATCC29328_08010	ATCC53516_15080	07T609_07300	08T492_07150	09T408_07370	09T494_08340	12T272_06100	12T273_11000	12T306_09210	CCUG54800_08440	T151023_07680	T160124_07600
12	12	1	Cytidine deaminase	ATCC29328_08020	ATCC53516_15090	07T609_07310	08T492_07160	09T408_07380	09T494_08350	12T272_06110	12T273_11010	12T306_09220	CCUG54800_08450	T151023_07690	T160124_07610
12	12	1	GTPase Era	ATCC29328_08030	ATCC53516_15100	07T609_07320	08T492_07170	09T408_07390	09T494_08360	12T272_06120	12T273_11020	12T306_09230	CCUG54800_08460	T151023_07700	T160124_07620
12	12	1	DNA repair protein RecO	ATCC29328_08040	ATCC53516_15110	07T609_07330	08T492_07180	09T408_07400	09T494_08370	12T272_06130	12T273_11030	12T306_09240	CCUG54800_08470	T151023_07710	T160124_07630
12	12	1	Glycine--tRNA ligase beta subunit	ATCC29328_08050	ATCC53516_15120	07T609_07340	08T492_07190	09T408_07410	09T494_08380	12T272_06140	12T273_11040	12T306_09250	CCUG54800_08480	T151023_07720	T160124_07640
12	24	0.167	Putative pyruvate, phosphate dikinase regulatı	ATCC29328_08060,ATCC2932ł	ATCC53516_15730,ATCC5351(	07T609_07950,07T609_(	08T492_07800,08T492_07	09T408_08030,09T408_(	09T494_08990,09T494_	12T272_06750,12T272_	12T273_11650,12T273_	12T306_09860,12T306_	CCUG54800_08490,CCUG548	T151023_08330,T151023_	T160124_08250,T160124_07650
12	12	1	Serine/threonine-protein kinase PK-1	ATCC29328_08070	ATCC53516_15140	07T609_07360	08T492_07210	09T408_07430	09T494_08400	12T272_06160	12T273_11060	12T306_09270	CCUG54800_08500	T151023_07740	T160124_07660
12	24	0.167	(Dimethylallyl)adenosine tRNA methylthiotran:	ATCC29328_08090,ATCC2932ł	ATCC53516_15500,ATCC5351(	07T609_07720,07T609_(	08T492_07570,08T492_07	09T408_07800,09T408_(	09T494_08760,09T494_	12T272_06520,12T272_	12T273_11420,12T273_	12T306_09630,12T306_	CCUG54800_08520,CCUG548	T151023_08100,T151023_	T160124_08020,T160124_07680
12	12	1	DNA mismatch repair protein MutS	ATCC29328_08100	ATCC53516_15170	07T609_07390	08T492_07240	09T408_07460	09T494_08430	12T272_06190	12T273_11090	12T306_09300	CCUG54800_08530	T151023_07770	T160124_07690
12	12	1	DNA mismatch repair protein MutL	ATCC29328_08110	ATCC53516_15180	07T609_07400	08T492_07250	09T408_07470	09T494_08440	12T272_06200	12T273_11100	12T306_09310	CCUG54800_08540	T151023_07780	T160124_07700
12	12	1	tRNA dimethylallyltransferase	ATCC29328_08120	ATCC53516_15190	07T609_07410	08T492_07260	09T408_07490	09T494_08450	12T272_06210	12T273_11110	12T306_09320	CCUG54800_08550	T151023_07790	T160124_07710
12	12	1	methionine gamma-lyase	ATCC29328_08130	ATCC53516_15200	07T609_07420	08T492_07270	09T408_07500	09T494_08460	12T272_06220	12T273_11120	12T306_09330	CCUG54800_08560	T151023_07800	T160124_07720
12	14	0.143	LexA repressor	ATCC29328_08140	ATCC53516_15210	07T609_07430	08T492_07280	09T408_07510	09T494_00460,09T494_	12T272_06230	12T273_11130	12T306_09340	CCUG54800_08570,CCUG548	T151023_07810	T160124_07730
12	12	1	Cell division suppressor protein YneA	ATCC29328_08150	ATCC53516_15220	07T609_07440	08T492_07290	09T408_07520	09T494_08480	12T272_06240	12T273_11140	12T306_09350	CCUG54800_08580	T151023_07820	T160124_07740
12	13	0.141	Tyrosine recombinase XerD	ATCC29328_08160,ATCC2932ł	ATCC53516_15230	07T609_07450	08T492_07300	09T408_07530	09T494_08490	12T272_06250	12T273_11150	12T306_09360	CCUG54800_08590	T151023_07830	T160124_07750
12	12	1	PHP domain protein	ATCC29328_08180	ATCC53516_15250	07T609_07470	08T492_07320	09T408_07550	09T494_08510	12T272_06270	12T273_11170	12T306_09380	CCUG54800_08610	T151023_07850	T160124_07770
12	12	1	Ribonuclease Y	ATCC29328_08190	ATCC53516_15260	07T609_07480	08T492_07330	09T408_07560	09T494_08520	12T272_06280	12T273_11180	12T306_09390	CCUG54800_08620	T151023_07860	T160124_07780
12	12	1	hypothetical protein	ATCC29328_08200	ATCC53516_15270	07T609_07490	08T492_07340	09T408_07570	09T494_08530	12T272_06290	12T273_11190	12T306_09400	CCUG54800_08630	T151023_07870	T160124_07790
12	12	1	hypothetical protein	ATCC29328_08210	ATCC53516_15280	07T609_07500	08T492_07350	09T408_07580	09T494_08540	12T272_06300	12T273_11200	12T306_09410	CCUG54800_08640	T151023_07880	T160124_07800
12	12	1	chromosome segregation protein	ATCC29328_08220	ATCC53516_15290	07T609_07510	08T492_07360	09T408_07590	09T494_08550	12T272_06310	12T273_11210	12T306_09420	CCUG54800_08650	T151023_07890	T160124_07810
12	12	1	3',5'-cyclic adenosine monophosphate phosph	ATCC29328_08230	ATCC53516_15300	07T609_07520	08T492_07370	09T408_07600	09T494_08560	12T272_06320	12T273_11220	12T306_09430	CCUG54800_08660	T151023_07900	T160124_07820
12	12	1	recombinase A	ATCC29328_08240	ATCC53516_15310	07T609_07530	08T492_07380	09T408_07610	09T494_08570	12T272_06330	12T273_11230	12T306_09440	CCUG54800_08670	T151023_07910	T160124_07830
12	12	1	CDP-diacylglycerol--glycerol-3-phosphate 3-ph	ATCC29328_08250	ATCC53516_15320	07T609_07540	08T492_07390	09T408_07620	09T494_08580	12T272_06340	12T273_11240	12T306_09450	CCUG54800_08680	T151023_07920	T160124_07840
12	12	1	DNA translocase SpoIIIE	ATCC29328_08260	ATCC53516_15330	07T609_07550	08T492_07400	09T408_07630	09T494_08590	12T272_06350	12T273_11250	12T306_09460	CCUG54800_08690	T151023_07930	T160124_07850
12	13	0.141	Deoxyuridine 5'-triphosphate nucleotidohydro	ATCC29328_08270,ATCC2932ł	ATCC53516_15340	07T609_07560	08T492_07410	09T408_07640	09T494_08600	12T272_06360	12T273_11260	12T306_09470	CCUG54800_08700	T151023_07940	T160124_07860
12	12	1	Polyribonucleotide nucleotidyltransferase	ATCC29328_08280	ATCC53516_15350	07T609_07570	08T492_07420	09T408_07650	09T494_08610	12T272_06370	12T273_11270	12T306_09480	CCUG54800_08710	T151023_07950	T160124_07870
12	12	1	30S ribosomal protein S15	ATCC29328_08290	ATCC53516_15360	07T609_07580	08T492_07430	09T408_07660	09T494_08620	12T272_06380	12T273_11280	12T306_09490	CCUG54800_08720	T151023_07960	T160124_07880
12	12	1	Riboflavin biosynthesis protein RibF	ATCC29328_08300	ATCC53516_15370	07T609_07590	08T492_07440	09T408_07670	09T494_08630	12T272_06390	12T273_11290	12T306_09500	CCUG54800_08730	T151023_07970	T160124_07890
12	12	1	tRNA pseudouridine synthase B	ATCC29328_08310	ATCC53516_15380	07T609_07600	08T492_07450	09T408_07680	09T494_08640	12T272_06400	12T273_11300	12T306_09510	CCUG54800_08740	T151023_07980	T160124_07900
12	12	1	Bifunctional oligoribonuclease and PAP phospl	ATCC29328_08320	ATCC53516_15390	07T609_07610	08T492_07460	09T408_07690	09T494_08650	12T272_06410	12T273_11310	12T306_09520	CCUG54800_08750	T151023_07990	T160124_07910
12	12	1	Ribosome-binding factor A	ATCC29328_08330	ATCC53516_15400	07T609_07620	08T492_07470	09T408_07700	09T494_08660	12T272_06420	12T273_11320	12T306_09530	CCUG54800_08760	T151023_08000	T160124_07920
12	12	1	Translation initiation factor IF-2	ATCC29328_08340	ATCC53516_15410	07T609_07630	08T492_07480	09T408_07710	09T494_08670	12T272_06430	12T273_11330	12T306_09540	CCUG54800_08770	T151023_08010	T160124_07930
12	12	1	hypothetical protein	ATCC29328_08350	ATCC53516_15420	07T609_07640	08T492_07490	09T408_07720	09T494_08680	12T272_06440	12T273_11340	12T306_09550	CCUG54800_08780	T151023_08020	T160124_07940
12	12	1	hypothetical protein	ATCC29328_08360	ATCC53516_15430	07T609_07650	08T492_07500	09T408_07730	09T494_08690	12T272_06450	12T273_11350	12T306_09560	CCUG54800_08790	T151023_08030	T160124_07950
12	12	1	Ribosome maturation factor RimP	ATCC29328_08370	ATCC53516_15440	07T609_07660	08T492_07510	09T408_07740	09T494_08700	12T272_06460	12T273_11360	12T306_09570	CCUG54800_08800	T151023_08040	T160124_07960
12	12	0.667	hypothetical protein	ATCC29328_08380	ATCC53516_15450	07T609_07670	08T492_07520	09T408_07750	09T494_08710	12T272_06470	12T273_11370	12T306_09580	CCUG54800_08810	T151023_08050	T160124_07970
12	12	1	SigmaW regulon antibacterial	ATCC29328_08390	ATCC53516_15460	07T609_07680	08T492_07530	09T408_07760	09T494_08720	12T272_06480	12T273_11380	12T306_09590	CCUG54800_08820	T151023_08060	T160124_07980
12	12	1	hypothetical protein	ATCC29328_08400	ATCC53516_15470	07T609_07690	08T492_07540	09T408_07770	09T494_08730	12T272_06490	12T273_11390	12T306_09600	CCUG54800_08830	T151023_08070	T160124_07990
12	12	1	30S ribosomal protein S21	ATCC29328_08410	ATCC53516_15480	07T609_07700	08T492_07550	09T408_07780	09T494_08740	12T272_06500	12T273_11400	12T306_09610	CCUG54800_08840	T151023_08080	T160124_08000
12	12	1	HIT-like protein	ATCC29328_08420	ATCC53516_15490	07T609_07710	08T492_07560	09T408_07790	09T494_08750	12T272_06510	12T273_11410	12T306_09620	CCUG54800_08850	T151023_08090	T160124_08010
12	12	1	Ribosomal RNA small subunit methyltransfera:	ATCC29328_08440	ATCC53516_15510	07T609_07730	08T492_07580	09T408_07810	09T494_08770	12T272_06530	12T273_11430	12T306_09640	CCUG54800_08870	T151023_08110	T160124_08030
12	12	1	Ribosomal protein L11 methyltransferase	ATCC29328_08450	ATCC53516_15520	07T609_07740	08T492_07590	09T408_07820	09T494_08780	12T272_06540	12T273_11440	12T306_09650	CCUG54800_08880	T151023_08120	T160124_08040
12	12	1	Chaperone protein DnaJ	ATCC29328_08460	ATCC53516_15530	07T609_07750	08T492_07600	09T408_07830	09T494_08790	12T272_06550	12T273_11450	12T306_09660	CCUG54800_08890	T151023_08130	T160124_08050
12	12	1	Chaperone protein DnaK	ATCC29328_08470	ATCC53516_15540	07T609_07760	08T492_07610	09T408_07840	09T494_08800	12T272_06560	12T273_11460	12T306_09670	CCUG54800_08900	T151023_08140	T160124_08060
12	12	1	heat shock protein GrpE	ATCC29328_08480	ATCC53516_15550	07T609_07770	08T492_07620	09T408_07850	09T494_08810	12T272_06570	12T273_11470	12T306_09680	CCUG54800_08910	T151023_08150	T160124_08070
12	12	1	Heat-inducible transcription repressor HrcA	ATCC29328_08490	ATCC53516_15560	07T609_07780	08T492_07630	09T408_07860	09T494_08820	12T272_06580	12T273_11480	12T306_09690	CCUG54800_08920	T151023_08160	T160124_08080
12	12	1	Oxygen-independent coproporphyrinogen-III (	ATCC29328_08500	ATCC53516_15570	07T609_07790	08T492_07640	09T408_07870	09T494_08830	12T272_06590	12T273_11490	12T306_09700	CCUG54800_08930	T151023_08170	T160124_08090
12	24	0.167	Elongation factor 4	ATCC29328_08510,ATCC2932ł	ATCC53516_18060,ATCC5351(	07T609_10470,07T609_(	08T492_10470,08T492_07	09T408_09980,09T408_(	09T494_10820,09T494_	12T272_08630,12T272_	12T273_14070,12T273_	12T306_12190,12T306_	CCUG54800_08940,CCUG548	T151023_10080,T151023_	T160124_10090,T160124_08100
12	12	1	30S ribosomal protein S20	ATCC29328_08530	ATCC53516_15600	07T609_07820	08T492_07670	09T408_07900	09T494_08860	12T272_06620	12T273_11520	12T306_09730	CCUG54800_08960	T151023_08200	T160124_08120
12	12	1	DNA polymerase III subunit delta	ATCC29328_08540	ATCC53516_15610	07T609_07830	08T492_07680	09T408_07910	09T494_08870	12T272_06630	12T273_11530	12T306_09740	CCUG54800_08970	T151023_08210	T160124_08130
12	12	1	ComEC family competence protein	ATCC29328_08550	ATCC53516_15620	07T609_07840	08T492_07690	09T408_07920	09T494_08880	12T272_06640	12T273_11540	12T306_09750	CCUG54800_08980	T151023_08220	T160124_08140
12	12	1	Enolase	ATCC29328_08560	ATCC53516_15630	07T609_07850	08T492_07700	09T408_07930	09T494_08890	12T272_06650	12T273_11550	12T306_09760	CCUG54800_08990	T151023_08230	T160124_08150
12	12	1	2,3-bisphosphoglycerate-independent phosph	ATCC29328_08570	ATCC53516_15640	07T609_07860	08T492_07710	09T408_07940	09T494_08900	12T272_06660	12T273_11560	12T306_09770	CCUG54800_09000	T151023_08240	T160124_08160
12	12	1	Triosephosphate isomerase	ATCC29328_08580	ATCC53516_15650	07T609_07870	08T492_07720	09T408_07950	09T494_08910	12T272_06670	12T273_11570	12T306_09780	CCUG54800_09010	T151023_08250	T160124_08170
12	12	1	Bifunctional PGK/TIM	ATCC29328_08590	ATCC53516_15660	07T609_07880	08T492_07730	09T408_07960	09T494_08920	12T272_06680	12T273_11580	12T306_09790	CCUG54800_09020	T151023_08260	T160124_08180
12	12	1	Glyceraldehyde-3-phosphate dehydrogenase	ATCC29328_08600	ATCC53516_15670	07T609_07890	08T492_07740	09T408_07970	09T494_08930	12T272_06690	12T273_11590	12T306_09800	CCUG54800_09030	T151023_08270	T160124_08190
12	12	1	transcriptional regulator PhoU	ATCC29328_08610	ATCC53516_15680	07T609_07900	08T492_07750	09T408_07980	09T494_08940	12T272_06700	12T273_11600	12T306_09810	CCUG54800_09040	T151023_08280	T160124_08200
12	12	1	tRNA (cytidine(34)-2'-O)-methyltransferase	ATCC29328_08620	ATCC53516_15690	07T609_07910	08T492_07760	09T408_07990	09T494_08950	12T272_06710	12T273_11610	12T306_09820	CCUG54800_09050	T151023_08290	T160124_08210
12	12	1	Transketolase	ATCC29328_08630	ATCC53516_15700	07T609_07920	08T492_07770	09T408_08000	09T494_08960	12T272_06720	12T273_11620	12T306_09830	CCUG54800_09060	T151023_08300	T160124_08220
12	12	1	hypothetical protein	ATCC29328_08640	ATCC53516_15710	07T609_07930	08T492_07780	09T408_08010	09T494_08970	12T272_06730	12T273_11630	12T306_09840	CCUG54800_09070	T151023_08310	T160124_08230
12	24	0.129	Pyruvate, phosphate dikinase	ATCC29328_08670,ATCC2932ł	ATCC53516_17830,ATCC5351(	07T609_10240,07T609_(	08T492_10240,08T492_07	09T408_09740,09T408_(	09T494_10590,09T494_	12T272_08390,12T272_	12T273_13840,12T273_	12T306_11960,12T306_	CCUG54800_09100,CCUG548	T151023_09850,T151023_	T160124_09860,T160124_08260
12	12	1	Lipid II:glycine glycyltransferase	ATCC29328_08680	ATCC53516_15750	07T609_07970	08T492_07820	09T408_08050	09T494_09010	12T272_06770	12T273_11670	12T306_09880	CCUG54800_09110	T151023_08350	T160124_08270
12	12	1	Multidrug export protein MepA	ATCC29328_08690	ATCC53516_15760	07T609_07980	08T492_07830	09T408_08060	09T494_09020	12T272_06780	12T273_11680	12T306_09890	CCUG54800_09120	T151023_08360	T160124_08280
12	12	1	33 kDa chaperonin	ATCC29328_08700	ATCC53516_15770	07T609_07990	08T492_07840	09T408_08070	09T494_09030	12T272_06790	12T273_11690	12T306_09900	CCUG54800_09130	T151023_08370	T160124_08290
12	24	0.167	putative cold shock protein A	ATCC29328_08710,ATCC2932ł	ATCC53516_00280,ATCC5351(	07T609_13660,07T609_(	08T492_14740,08T492_07	09T408_13120,09T408_(	09T494_16690,09T494_	12T272_14100,12T272_	12T273_17280,12T273_	12T306_15300,12T306_	CCUG54800_09140,CCUG548	T151023_14250,T151023_	T160124_13040,T160124_08300
12	12	1	trans-aconitate 2-methyltransferase	ATCC29328_08720	ATCC53516_15790	07T609_08010	08T492_07860	09T408_08090	09T494_09050	12T272_06810	12T273_11710	12T306_09920	CCUG54800_09150	T151023_08390	T160124_08310
12	24	0.167	Hypoxanthine phosphoribosyltransferase	ATCC29328_08730,ATCC2932ł	ATCC53516_17590,ATCC5351(	07T609_10000,07T609_(	08T492_10000,08T492_07	09T408_09490,09T408_(	09T494_10350,09T494_	12T272_08140,12T272_	12T273_13600,12T273_	12T306_11710,12T306_	CCUG54800_09160,CCUG548	T151023_09610,T151023_	T160124_09630,T160124_08320
1	1	1	hypothetical protein	ATCC29328_08740	*	*	*	*	*	*	*	*	*	*	*
12	24	0.167	Valine--tRNA ligase	ATCC29328_08750,ATCC2932ł	ATCC53516_02580,ATCC5351(	07T609_11180,07T609_(	08T492_11100,08T492_07	09T408_10680,09T408_(	09T494_11850,09T494_	12T272_09320,12T272_	12T273_14780,12T273_	12T306_12790,12T306_	CCUG54800_09180,CCUG548	T151023_10680,T151023_	T160124_10800,T160124_08330
12	12	1	putative metallo-hydrolase	ATCC29328_08760	ATCC53516_15830	07T609_08050	08T492_07900	09T408_08130	09T494_09090	12T272_06850	12T273_11740	12T306_09960	CCUG54800_09190	T151023_08430	T160124_08340
12	12	1	D-tyrosyl-tRNA(Tyr) deacylase	ATCC29328_08770	ATCC53516_15840	07T609_08060	08T492_07910	09T408_08140	09T494_09100	12T272_06860	12T273_11750	12T306_09970	CCUG54800_09200	T151023_08440	T160124_08350
12	12	1	GTP pyrophosphokinase	ATCC29328_08780	ATCC53516_15850	07T609_08070	08T492_07920	09T408_08150	09T494_09110	12T272_06870	12T273_11760	12T306_09980	CCUG54800_09210	T151023_08450	T160124_08360
12	12	1	Single-stranded-DNA-specific exonuclease Rec.	ATCC29328_08790	ATCC53516_15860	07T609_08080	08T492_07930	09T408_08160	09T494_09120	12T272_06880	12T273_11770	12T306_09990	CCUG54800_09220	T151023_08460	T160124_08370
12	12	1	preprotein translocase subunit YajC	ATCC29328_08800	ATCC53516_15870	07T609_08090	08T492_07940	09T408_08170	09T494_09130	12T272_06890	12T273_11780	12T306_10000	CCUG54800_09230	T151023_08470	T160124_08380
12	12	1	Queuine tRNA-ribosyltransferase	ATCC29328_08810	ATCC53516_15900	07T609_08100	08T492_07950	09T408_08180	09T494_09140	12T272_06900	12T273_11790	12T306_10020	CCUG54800_09240	T151023_08480	T160124_08390
12	12	1	S-adenosylmethionine:tRNA ribosyltransferase	ATCC29328_08820	ATCC53516_15910	07T609_08110	08T492_07960	09T408_08190	09T494_09150	12T272_06910	12T273_11800	12T306_10030	CCUG54800_09250	T151023_08490	T160124_08400
12	12	1	Holliday junction ATP-dependent DNA helicase	ATCC29328_08830	ATCC53516_15920	07T609_08120	08T492_07970	09T408_08200	09T494_09160	12T272_06920	12T273_11810	12T306_10040	CCUG54800_09260	T151023_08500	T160124_08410
12	12	1	Holliday junction ATP-dependent DNA helicase	ATCC29328_08840	ATCC53516_15930	07T609_08130	08T492_07980	09T408_08210	09T494_09170	12T272_06930	12T273_11820	12T306_10050	CCUG54800_09270	T151023_08510	T160124_08420
12	12	1	Crossover junction endodeoxyribonuclease Ru	ATCC29328_08850	ATCC53516_15940	07T609_08140	08T492_07990	09T408_08220	09T494_09180	12T272_06940	12T273_11830	12T306_10060	CCUG54800_09280	T151023_08520	T160124_08430
12	15	0.145	3-oxoacyl-[acyl-carrier-protein] reductase Fab(	ATCC29328_08860	ATCC53516_15950	07T609_08150	08T492_08000	09T408_08230	09T494_09190	12T272_16740,12T272_	12T273_00470,12T273_	12T306_10070,12T306_	CCUG54800_09290	T151023_08530	T160124_08440
12	12	1	Transcriptional regulatory protein OmpR	ATCC29328_08870	ATCC53516_15960	07T609_08160	08T492_08010	09T408_08240	09T494_09200	12T272_06960	12T273_11850	12T306_10080	CCUG54800_09300	T151023_08540	T160124_08450
12	13	0.141	Alkaline phosphatase synthesis sensor protein	ATCC29328_08880	ATCC53516_15970	07T609_08170	08T492_08020,08T492_13	09T408_08250	09T494_09210	12T272_06970	12T273_11860	12T306_10090	CCUG54800_09310	T151023_08550	T160124_08460
12	12	1	Putative GTP cyclohydrolase 1 type 2	ATCC29328_08890	ATCC53516_15980	07T609_08180	08T492_08030	09T408_08260	09T494_09220	12T272_06980	12T273_11870	12T306_10100	CCUG54800_09320	T151023_08560	T160124_08470
12	12	1	tRNA (adenine(22)-N(1))-methyltransferase	ATCC29328_08900	ATCC53516_15990	07T609_08190	08T492_08040	09T408_08270	09T494_09230	12T272_06990	12T273_11880	12T306_10110	CCUG54800_09330	T151023_08570	T160124_08480
12	12	1	DNA primase	ATCC29328_08920	ATCC53516_16010	07T609_08210	08T492_08060	09T408_08290	09T494_09250	12T272_07010	12T273_11900	12T306_10130	CCUG54800_09350	T151023_08590	T160124_08500
12	12	1	Deoxyguanosinetriphosphate triphosphohydr(	ATCC29328_08930	ATCC53516_16020	07T609_08220	08T492_08070	09T408_08300	09T494_09260	12T272_07020	12T273_11910	12T306_10140	CCUG54800_09360	T151023_08600	T160124_08510
12	12	1	30S ribosomal protein S1	ATCC29328_08940	ATCC53516_16030	07T609_08230	08T492_08080	09T408_08310	09T494_09270	12T272_07030	12T273_11920	12T306_10150	CCUG54800_09370	T151023_08610	T160124_08520
12	12	1	1-acyl-sn-glycerol-3-phosphate acyltransferase	ATCC29328_08950	ATCC53516_16040	07T609_08240	08T492_08090	09T408_08320	09T494_09280	12T272_07040	12T273_11930	12T306_10160	CCUG54800_09380	T151023_08620	T160124_08530
12	12	1	Cytidylate kinase	ATCC29328_08960	ATCC53516_16050	07T609_08250	08T492_08100	09T408_08330	09T494_09290	12T272_07050	12T273_11940	12T306_10170	CCUG54800_09390	T151023_08630	T160124_08540
12	12	1	16S rRNA m(4)C1402 methyltranserfase	ATCC29328_08980	ATCC53516_16070	07T609_08270	08T492_08120	09T408_08350	09T494_09310	12T272_07070	12T273_11960	12T306_10190	CCUG54800_09410	T151023_08650	T160124_08560
12	12	1	hypothetical protein	ATCC29328_08990	ATCC53516_16080	07T609_08280	08T492_08130	09T408_08360	09T494_09320	12T272_07080	12T273_11970	12T306_10200	CCUG54800_09420	T151023_08660	T160124_08570
12	24	0.167	Ribosomal large subunit pseudouridine syntha	ATCC29328_09000,ATCC2932ł	ATCC53516_00020,ATCC5351(	07T609_13450,07T609_(	08T492_14560,08T492_08	09T408_12910,09T408_(	09T494_16530,09T494_	12T272_13900,12T272_	12T273_17090,12T273_	12T306_15100,12T306_	CCUG54800_09430,CCUG548	T151023_14050,T151023_	T160124_12850,T160124_08580
12	12	1	Segregation and condensation protein B	ATCC29328_09010	ATCC53516_16100	07T609_08300	08T492_08150	09T408_08380	09T494_09340	12T272_07100	12T273_11990	12T306_10220	CCUG54800_09440	T151023_08680	T160124_08590
12	12	1	Segregation and condensation protein A	ATCC29328_09020	ATCC53516_16110	07T609_08310	08T492_08160	09T408_08390	09T494_09350	12T272_07110	12T273_12000	12T306_10230	CCUG54800_09450	T151023_08690	T160124_08600
12	12	1	Peptidase family M50	ATCC29328_09030	ATCC53516_16120	07T609_08320	08T492_08170	09T408_08400	09T494_09360	12T272_07120	12T273_12010	12T306_10240	CCUG54800_09460	T151023_08700	T160124_08610
12	12	1	ADP-ribose pyrophosphatase	ATCC29328_09040	ATCC53516_16130	07T609_08330	08T492_08180	09T408_08410	09T494_09370	12T272_07130	12T273_12020	12T306_10250	CCUG54800_09470	T151023_08710	T160124_08620
12	12	1	DNA repair protein RecN	ATCC29328_09050	ATCC53516_16140	07T609_08340	08T492_08190	09T408_08420	09T494_09380	12T272_07140	12T273_12030	12T306_10260	CCUG54800_09480	T151023_08720	T160124_08630
12	12	1	Arginine repressor	ATCC29328_09060	ATCC53516_16150	07T609_08350	08T492_08200	09T408_08430	09T494_09390	12T272_07150	12T273_12040	12T306_10270	CCUG54800_09490	T151023_08730	T160124_08640
12	12	1	Hemolysin A	ATCC29328_09070	ATCC53516_16160	07T609_08360	08T492_08210	09T408_08440	09T494_09400	12T272_07160	12T273_12050	12T306_10280	CCUG54800_09500	T151023_08740	T160124_08660
12	12	1	Farnesyl diphosphate synthase	ATCC29328_09080	ATCC53516_16170	07T609_08370	08T492_08220	09T408_08450	09T494_09410	12T272_07170	12T273_12060	12T306_10290	CCUG54800_09510	T151023_08750	T160124_08670
12	12	1	exodeoxyribonuclease VII small subunit	ATCC29328_09090	ATCC53516_16180	07T609_08380	08T492_08230	09T408_08460	09T494_09420	12T272_07180	12T273_12070	12T306_10300	CCUG54800_09520	T151023_08760	T160124_08680
12	12	1	Exodeoxyribonuclease 7 large subunit	ATCC29328_09100	ATCC53516_16190	07T609_08390	08T492_08240	09T408_08470	09T494_09430	12T272_07190	12T273_12080	12T306_10310	CCUG54800_09530	T151023_08770	T160124_08690
12	12	1	hypothetical protein	ATCC29328_09110	ATCC53516_16200	07T609_08400	08T492_08250	09T408_08480	09T494_09440	12T272_07200	12T273_12090	12T306_10320	CCUG54800_09540	T151023_08780	T160124_08700
12	12	1	hypothetical protein	ATCC29328_09120	ATCC53516_16210	07T609_08410	08T492_08260	09T408_08490	09T494_09450	12T272_07210	12T273_12100	12T306_10330	CCUG54800_09550	T151023_08790	T160124_08710
12	12	1	Signal recognition particle receptor FtsY	ATCC29328_09130	ATCC53516_16220	07T609_08420	08T492_08270	09T408_08500	09T494_09460	12T272_07220	12T273_12110	12T306_10340	CCUG54800_09560	T151023_08800	T160124_08720
12	12	1	Chromosome partition protein Smc	ATCC29328_09140	ATCC53516_16230	07T609_08430	08T492_08280	09T408_08510	09T494_09470	12T272_07230	12T273_12120	12T306_10350	CCUG54800_09570	T151023_08810	T160124_08730
12	12	1	coproporphyrinogen III oxidase	ATCC29328_09150	ATCC53516_16240	07T609_08440	08T492_08290	09T408_08520	09T494_09480	12T272_07240	12T273_12130	12T306_10360	CCUG54800_09580	T151023_08820	T160124_08740
12	12	1	Ribonuclease 3	ATCC29328_09160	ATCC53516_16250	07T609_08450	08T492_08300	09T408_08530	09T494_09490	12T272_07250	12T273_12140	12T306_10370	CCUG54800_09590	T151023_08830	T160124_08750
12	12	1	Acyl carrier protein	ATCC29328_09170	ATCC53516_16260	07T609_08460	08T492_08310	09T408_08540	09T494_09500	12T272_07260	12T273_12150	12T306_10380	CCUG54800_09600	T151023_08840	T160124_08760
12	12	1	Phosphate acyltransferase	ATCC29328_09180	ATCC53516_16270	07T609_08470	08T492_08320	09T408_08550	09T494_09510	12T272_07270	12T273_12160	12T306_10390	CCUG54800_09610	T151023_08850	T160124_08770
12	12	1	50S ribosomal protein L32	ATCC29328_09190	ATCC53516_16280	07T609_08480	08T492_08330	09T408_08560	09T494_09520	12T272_07280	12T273_12170	12T306_10400	CCUG54800_09620	T151023_08860	T160124_08780
12	12	1	Acetate kinase	ATCC29328_09200	ATCC53516_16290	07T609_08490	08T492_08340	09T408_08570	09T494_09530	12T272_07290	12T273_12180	12T306_10410	CCUG54800_09630	T151023_08870	T160124_08790
12	12	1	Glycerol-3-phosphate acyltransferase	ATCC29328_09210	ATCC53516_16300	07T609_08500	08T492_08350	09T408_08580	09T494_09540	12T272_07300	12T273_12190	12T306_10420	CCUG54800_09640	T151023_08880	T160124_08800
12	12	1	GTPase Der	ATCC29328_09220	ATCC53516_16310	07T609_08510	08T492_08360	09T408_08590	09T494_09550	12T272_07310	12T273_12200	12T306_10430	CCUG54800_09650	T151023_08890	T160124_08810
12	12	1	Serine hydroxymethyltransferase	ATCC29328_09230	ATCC53516_16320	07T609_08520	08T492_08370	09T408_08600	09T494_09560	12T272_07320	12T273_12210	12T306_10440	CCUG54800_09660	T151023_08900	T160124_08820

12	12	1	Positive regulator of sigma(E), RseC/MucC	ATCC29328_09240	ATCC53516_16330	07T609_08530	08T492_08380	09T408_08610	09T494_09570	12T272_07330	12T273_12220	12T306_10450	CCUG54800_09670	T151023_08910	T160124_08830
12	12	1	tRNA threonylcarbamoyladenosine dehydratas	ATCC29328_09250	ATCC53516_16340	07T609_08540	08T492_08390	09T408_08620	09T494_09580	12T272_07340	12T273_12230	12T306_10460	CCUG54800_09680	T151023_08920	T160124_08840
12	12	1	Aspartate--tRNA ligase	ATCC29328_09260	ATCC53516_16350	07T609_08550	08T492_08400	09T408_08630	09T494_09590	12T272_07350	12T273_12240	12T306_10470	CCUG54800_09690	T151023_08930	T160124_08850
12	12	1	putative peptidoglycan biosynthesis protein M	ATCC29328_09270	ATCC53516_16360	07T609_08560	08T492_08410	09T408_08640	09T494_09600	12T272_07360	12T273_12250	12T306_10480	CCUG54800_09700	T151023_08940	T160124_08860
12	12	1	carbohydrate diacid transcriptional activator C	ATCC29328_09280	ATCC53516_16370	07T609_08570	08T492_08420	09T408_08650	09T494_09610	12T272_07370	12T273_12260	12T306_10490	CCUG54800_09710	T151023_08950	T160124_08870
12	12	1	Tetratricopeptide repeat protein	ATCC29328_09290	ATCC53516_16380	07T609_08580	08T492_08430	09T408_08660	09T494_09620	12T272_07380	12T273_12270	12T306_10500	CCUG54800_09720	T151023_08960	T160124_08880
12	12	1	hypothetical protein	ATCC29328_09300	ATCC53516_16390	07T609_08590	08T492_08440	09T408_08670	09T494_09630	12T272_07390	12T273_12280	12T306_10510	CCUG54800_09730	T151023_08970	T160124_08890
12	12	1	Arginine--tRNA ligase	ATCC29328_09310	ATCC53516_16400	07T609_08600	08T492_08450	09T408_08680	09T494_09640	12T272_07400	12T273_12290	12T306_10520	CCUG54800_09740	T151023_08980	T160124_08900
12	12	1	Endonuclease MutS2	ATCC29328_09320	ATCC53516_16410	07T609_08610	08T492_08460	09T408_08690	09T494_09650	12T272_07410	12T273_12300	12T306_10530	CCUG54800_09750	T151023_08990	T160124_08910
12	12	1	putative protease YhbU precursor	ATCC29328_09330	ATCC53516_16440	07T609_08620	08T492_08470	09T408_08700	09T494_09660	12T272_07420	12T273_12310	12T306_10540	CCUG54800_09760	T151023_09000	T160124_08920
12	12	1	chromosome segregation protein	ATCC29328_09340	ATCC53516_16450	07T609_08630	08T492_08480	09T408_08710	09T494_09670	12T272_07430	12T273_12320	12T306_10550	CCUG54800_09770	T151023_09010	T160124_08930
12	12	1	Phenylalanine--tRNA ligase beta subunit	ATCC29328_09350	ATCC53516_16460	07T609_08640	08T492_08490	09T408_08720	09T494_09680	12T272_07440	12T273_12330	12T306_10560	CCUG54800_09780	T151023_09020	T160124_08940
12	12	1	Phenylalanine--tRNA ligase alpha subunit	ATCC29328_09360	ATCC53516_16470	07T609_08650	08T492_08500	09T408_08730	09T494_09690	12T272_07450	12T273_12340	12T306_10570	CCUG54800_09790	T151023_09030	T160124_08950
12	12	1	Ktr system potassium uptake protein A	ATCC29328_09380	ATCC53516_16490	07T609_08670	08T492_08520	09T408_08750	09T494_09710	12T272_07470	12T273_12360	12T306_10590	CCUG54800_09810	T151023_09050	T160124_08970
12	12	1	Ktr system potassium uptake protein B	ATCC29328_09390	ATCC53516_16500	07T609_08680	08T492_08530	09T408_08760	09T494_09720	12T272_07480	12T273_12370	12T306_10600	CCUG54800_09820	T151023_09060	T160124_08980
12	12	1	Aspartate--ammonia ligase	ATCC29328_09400	ATCC53516_16510	07T609_08690	08T492_08540	09T408_08770	09T494_09730	12T272_07490	12T273_12380	12T306_10610	CCUG54800_09830	T151023_09070	T160124_08990
12	12	1	Putative septation protein SpoVG	ATCC29328_09410	ATCC53516_16520	07T609_08700	08T492_08550	09T408_08780	09T494_09740	12T272_07500	12T273_12390	12T306_10620	CCUG54800_09840	T151023_09080	T160124_09000
12	12	1	Processive diacylglycerol alpha-glucosyltransfe	ATCC29328_09420	ATCC53516_16530	07T609_08710	08T492_08560	09T408_08790	09T494_09750	12T272_07510	12T273_12400	12T306_10630	CCUG54800_09850	T151023_09090	T160124_09010
12	12	1	TVP38/TMEM64 family inner membrane prote	ATCC29328_09430	ATCC53516_16540	07T609_08720	08T492_08570	09T408_08800	09T494_09760	12T272_07520	12T273_12410	12T306_10640	CCUG54800_09860	T151023_09100	T160124_09020
12	12	1	Alpha-monoglucosyldiacylglycerol synthase	ATCC29328_09440	ATCC53516_16550	07T609_08730	08T492_08580	09T408_08810	09T494_09770	12T272_07530	12T273_12420	12T306_10650	CCUG54800_09870	T151023_09110	T160124_09030
12	12	1	hypothetical protein	ATCC29328_09450	ATCC53516_16560	07T609_08740	08T492_08590	09T408_08820	09T494_09780	12T272_07540	12T273_12430	12T306_10660	CCUG54800_09880	T151023_09120	T160124_09040
12	12	1	Redox-sensing transcriptional repressor Rex	ATCC29328_09460	ATCC53516_16570	07T609_08750	08T492_08600	09T408_08830	09T494_09790	12T272_07550	12T273_12440	12T306_10670	CCUG54800_09890	T151023_09140	T160124_09050
11	11	1	Carbon starvation protein A	ATCC29328_09470	ATCC53516_16580	07T609_08760	08T492_08610	09T408_08840	09T494_09800	12T272_07560	12T273_12450	12T306_10680	CCUG54800_09900	*	T160124_09070
12	12	1	Bifunctional folate synthesis protein	ATCC29328_09480	ATCC53516_16590	07T609_08770	08T492_08630	09T408_08850	09T494_09810	12T272_07570	12T273_12460	12T306_10690	CCUG54800_09910	T151023_09150	T160124_09080
12	12	1	Dihydropteroate synthase	ATCC29328_09490	ATCC53516_16600	07T609_08780	08T492_08640	09T408_08860	09T494_09820	12T272_07580	12T273_12470	12T306_10700	CCUG54800_09920	T151023_09160	T160124_09090
12	12	1	phosphodiesterase	ATCC29328_09500	ATCC53516_16610	07T609_08790	08T492_08650	09T408_08870	09T494_09830	12T272_07590	12T273_12480	12T306_10710	CCUG54800_09930	T151023_09170	T160124_09100
12	12	1	GTP cyclohydrolase 1	ATCC29328_09510	ATCC53516_16620	07T609_08800	08T492_08660	09T408_08880	09T494_09840	12T272_07600	12T273_12490	12T306_10720	CCUG54800_09940	T151023_09180	T160124_09110
12	12	1	hypothetical protein	ATCC29328_09520	ATCC53516_16630	07T609_08810	08T492_08670	09T408_08890	09T494_09850	12T272_07610	12T273_12500	12T306_10730	CCUG54800_09950	T151023_09190	T160124_09120
12	12	1	GTPase YlqF	ATCC29328_09530	ATCC53516_16640	07T609_08820	08T492_08680	09T408_08900	09T494_09860	12T272_07620	12T273_12510	12T306_10740	CCUG54800_09960	T151023_09200	T160124_09130
12	12	1	60 kDa chaperonin	ATCC29328_09540	ATCC53516_16650	07T609_08830	08T492_08690	09T408_08910	09T494_09870	12T272_07630	12T273_12520	12T306_10750	CCUG54800_09970	T151023_09210	T160124_09140
12	12	1	10 kDa chaperonin	ATCC29328_09550	ATCC53516_16660	07T609_08840	08T492_08700	09T408_08920	09T494_09880	12T272_07640	12T273_12530	12T306_10760	CCUG54800_09980	T151023_09220	T160124_09150
12	12	1	DNA-3-methyladenine glycosylase	ATCC29328_09560	ATCC53516_16670	07T609_08850	08T492_08710	09T408_08930	09T494_09890	12T272_07650	12T273_12540	12T306_10770	CCUG54800_09990	T151023_09230	T160124_09160
12	12	1	NAD-specific glutamate dehydrogenase	ATCC29328_09570	ATCC53516_16680	07T609_08860	08T492_08720	09T408_08940	09T494_09900	12T272_07660	12T273_12550	12T306_10780	CCUG54800_10000	T151023_09240	T160124_09170
12	12	1	Glycine--tRNA ligase	ATCC29328_09580	ATCC53516_16690	07T609_08870	08T492_08730	09T408_08950	09T494_09910	12T272_07670	12T273_12560	12T306_10790	CCUG54800_10010	T151023_09250	T160124_09180
12	12	1	hypothetical protein	ATCC29328_09590	ATCC53516_16700	07T609_08880	08T492_08740	09T408_08960	09T494_09920	12T272_07680	12T273_12570	12T306_10800	CCUG54800_10020	T151023_09260	T160124_09190
12	12	1	Prolipoprotein diacylglyceryl transferase	ATCC29328_09600	ATCC53516_16710	07T609_08890	08T492_08750	09T408_08970	09T494_09930	12T272_07690	12T273_12580	12T306_10810	CCUG54800_10030	T151023_09270	T160124_09200
12	12	1	putative transcriptional regulatory protein	ATCC29328_09610	ATCC53516_16720	07T609_08900	08T492_08760	09T408_08980	09T494_09940	12T272_07700	12T273_12590	12T306_10820	CCUG54800_10040	T151023_09280	T160124_09210
12	12	1	NH(3)-dependent NAD(+) synthetase	ATCC29328_09620	ATCC53516_16730	07T609_08910	08T492_08770	09T408_08990	09T494_09950	12T272_07710	12T273_12600	12T306_10830	CCUG54800_10050	T151023_09290	T160124_09220
12	12	1	IMPACT family member YigZ	ATCC29328_09630	ATCC53516_16740	07T609_08920	08T492_08780	09T408_09000	09T494_09960	12T272_07720	12T273_12610	12T306_10840	CCUG54800_10060	T151023_09300	T160124_09230
12	12	1	Penicillin-binding protein 1A	ATCC29328_09650	ATCC53516_16760	07T609_08940	08T492_08800	09T408_09020	09T494_09980	12T272_07740	12T273_12630	12T306_10860	CCUG54800_10080	T151023_09320	T160124_09250
5	6	0.271	Alkaline phosphatase synthesis transcriptional	ATCC29328_09670	*	*	08T492_08820	*	*	*	12T273_12730	12T306_10880,12T306_	*	T151023_09360	*
12	12	1	UvrABC system protein A	ATCC29328_09680	ATCC53516_16880	07T609_08960	08T492_08830	09T408_09130	09T494_10080	12T272_07840	12T273_12740	12T306_10890	CCUG54800_10180	T151023_09370	T160124_09350
12	12	1	UvrABC system protein B	ATCC29328_09690	ATCC53516_16890	07T609_08970	08T492_08840	09T408_09140	09T494_10090	12T272_07850	12T273_12750	12T306_10900	CCUG54800_10190	T151023_09380	T160124_09360
12	12	1	Serine/threonine transporter SstT	ATCC29328_09700	ATCC53516_16900	07T609_08980	08T492_08850	09T408_09150	09T494_10100	12T272_07860	12T273_12760	12T306_10910	CCUG54800_10200	T151023_09390	T160124_09370
12	12	1	N-acetylmuramoyl-L-alanine amidase LytC pre	ATCC29328_09710	ATCC53516_16950	07T609_08990	08T492_08860	09T408_09180	09T494_10140	12T272_07890	12T273_12770	12T306_10920	CCUG54800_10210	T151023_09400	T160124_09380
12	12	1	Putative peptidyl-prolyl cis-trans isomerase Cb	ATCC29328_09720	ATCC53516_16960	07T609_09000	08T492_08870	09T408_09190	09T494_10150	12T272_07900	12T273_12780	12T306_10930	CCUG54800_10220	T151023_09410	T160124_09390
12	12	1	hypothetical protein	ATCC29328_09730	ATCC53516_16970	07T609_09010	08T492_08880	09T408_09200	09T494_10160	12T272_07910	12T273_12790	12T306_10940	CCUG54800_10230	T151023_09420	T160124_09400
12	12	1	hypothetical protein	ATCC29328_09780	ATCC53516_17030	07T609_09070	08T492_08930	09T408_09260	09T494_10210	12T272_07970	12T273_12850	12T306_10990	CCUG54800_10290	T151023_09470	T160124_09470
12	12	1	Phosphate regulon sensor protein PhoR	ATCC29328_09790	ATCC53516_17040	07T609_09080	08T492_08940	09T408_09270	09T494_10220	12T272_07980	12T273_12860	12T306_11000	CCUG54800_10300	T151023_09480	T160124_09480
12	12	1	Sensory transduction protein regX3	ATCC29328_09800	ATCC53516_17050	07T609_09090	08T492_08950	09T408_09280	09T494_10230	12T272_07990	12T273_12870	12T306_11010	CCUG54800_10310	T151023_09490	T160124_09490
12	24	0.167	FtsX-like permease family protein	ATCC29328_09810,ATCC2932	ATCC53516_17080,ATCC5351	07T609_09120,07T609_	08T492_08980,08T492_08	09T408_09310,09T408_	09T494_10260,09T494_	12T272_08020,12T272_	12T273_12900,12T273_	12T306_11040,12T306_	CCUG54800_10320,CCUG548	T151023_09520,T151023_	T160124_09520,T160124_09500
12	12	1	putative ABC transporter ATP-binding protein/	ATCC29328_09820	ATCC53516_17070	07T609_09110	08T492_08970	09T408_09300	09T494_10250	12T272_08010	12T273_12890	12T306_11030	CCUG54800_10330	T151023_09510	T160124_09510
10	10	1	hypothetical protein	ATCC29328_09840	*	07T609_09130	08T492_08990	09T408_09340	*	12T272_11560	12T273_05850	12T306_15000	CCUG54800_14880	T151023_12810	T160124_02480
10	10	1	Uracil DNA glycosylase superfamily protein	ATCC29328_09850	*	07T609_09140	08T492_09000	09T408_09350	*	12T272_11570	12T273_05840	12T306_15010	CCUG54800_14890	T151023_12820	T160124_02470
4	4	1	hypothetical protein	ATCC29328_09860	*	07T609_09150	08T492_09010	09T408_09360	*	*	*	*	*	*	*
4	4	0.5	putative ABC transporter ATP-binding protein	ATCC29328_09870	*	07T609_09170	08T492_09020	09T408_09370	*	*	*	*	*	*	*
3	3	1	putative ABC transporter permease protein	ATCC29328_09880	*	07T609_09180	08T492_09030	*	*	*	*	*	*	*	*
2	2	1	hypothetical protein	ATCC29328_09890	*	*	08T492_09040	*	*	*	*	*	*	*	*
4	4	1	Demethylrebeccamycin-D-glucose O-methyltra	ATCC29328_09900	*	07T609_09190	08T492_09050	09T408_09390	*	*	*	*	*	*	*
1	1	1	hypothetical protein	ATCC29328_09920	*	*	*	*	*	*	*	*	*	*	*
11	22	0.164	Integrase core domain protein	ATCC29328_09930	ATCC53516_09990,ATCC5351	07T609_09220	08T492_03290	09T408_14870,09T408_	09T494_18700	12T272_12220,12T272_	12T273_18820,12T273_	*	CCUG54800_10400,CCUG548	T151023_09340	T160124_17060,T160124_17100
11	14	0.156	FtsX-like permease family protein	ATCC29328_09940,ATCC2932	ATCC53516_00420	07T609_13740,07T609_	08T492_09090,08T492_14	09T408_13200	09T494_16800	12T272_14230	*	12T306_15410	CCUG54800_15270	T151023_14340	T160124_13180
3	3	1	FMN-binding domain protein	ATCC29328_09950	*	07T609_09240	08T492_09100	*	*	*	*	*	*	*	*
6	6	1	Methionine--tRNA ligase	ATCC29328_09960	ATCC53516_17090	*	08T492_09230	*	*	*	12T273_12930	12T306_11050	CCUG54800_10350	*	*
7	9	0.333	Transposase from transposon Tn916	ATCC29328_09970	ATCC53516_04440,ATCC5351	*	08T492_11940,08T492_09	*	*	*	12T273_12940	12T306_11060	CCUG54800_10360	*	T160124_11510
6	6	1	Excisionase from transposon Tn916	ATCC29328_09980	ATCC53516_17110	*	08T492_09250	*	*	*	12T273_12950	12T306_11070	CCUG54800_10370	*	*
10	16	0.111	hypothetical protein	ATCC29328_09990,ATCC2932	ATCC53516_17120	07T609_09670	08T492_09800	09T408_14060,09T408_	09T494_15810,09T494_	12T272_17000	12T273_13380,12T273_	12T306_11460,12T306_	CCUG54800_10380,CCUG548	*	*
3	3	1	Multidrug export protein MepA	ATCC29328_10000	ATCC53516_17130	*	08T492_09280	*	*	*	*	*	*	*	*
5	5	1	Putative multidrug export ATP-binding/permea	ATCC29328_10010	ATCC53516_17140	*	08T492_09290	*	*	*	*	12T306_11120	CCUG54800_10440	*	*
6	6	0.667	Putative multidrug export ATP-binding/permea	ATCC29328_10020	ATCC53516_17150	*	08T492_09300	09T408_13830	*	*	*	12T306_11130	CCUG54800_10450	*	*
4	4	1	Putative HMP/thiamine import ATP-binding pr	ATCC29328_10030	ATCC53516_17160	07T609_14410	08T492_09310	*	*	*	*	*	*	*	*
4	4	1	Energy-coupling factor transporter transmemb	ATCC29328_10040	ATCC53516_17170	07T609_14420	08T492_09320	*	*	*	*	*	*	*	*
4	4	1	hypothetical protein	ATCC29328_10050	ATCC53516_17180	07T609_14430	08T492_09330	*	*	*	*	*	*	*	*
10	19	0.16	putative HTH-type transcriptional regulator Yfi	ATCC29328_10060,ATCC2932	ATCC53516_01220,ATCC5351	07T609_14310,07T609_	08T492_15460,08T492_09	*	09T494_15640	12T272_17070,12T272_	12T273_03740	12T306_16150,12T306_	CCUG54800_15880,CCUG548	*	T160124_14010
11	19	0.14	hypothetical protein	ATCC29328_10070,ATCC2932	*	07T609_14320,07T609_	08T492_15470,08T492_09	09T408_13850	09T494_15870,09T494_	12T272_12660,12T272_	12T273_13040	12T306_15890,12T306_	CCUG54800_10470,CCUG548	T151023_02780	T160124_14020
9	15	0.125	Relaxase/Mobilisation nuclease domain protei	ATCC29328_10080	ATCC53516_17210	*	08T492_13310,08T492_09	09T408_13860	09T494_13930,09T494_	12T272_17170,12T272_	12T273_13050,12T273_	*	CCUG54800_19260,CCUG548	*	T160124_14030
3	3	1	FtsX-like permease family protein	ATCC29328_10090	*	*	*	*	*	*	*	12T306_16010	*	*	T160124_13790
5	10	0.262	Lipoprotein-releasing system ATP-binding prot	ATCC29328_10100,ATCC2932	*	*	*	*	09T494_11450,09T494_	*	*	12T306_16020	CCUG54800_11990,CCUG548	*	T160124_13800,T160124_10400
3	4	0.36	Sensor kinase CusS	ATCC29328_10110	*	*	*	*	*	*	*	12T306_16030,12T306_	*	*	T160124_13810
3	4	0.36	Phosphate regulon transcriptional regulatory p	ATCC29328_10120	*	*	*	*	*	*	*	12T306_16040,12T306_	*	*	T160124_13820
1	1	1	ATP-dependent DNA helicase Rep	ATCC29328_10130	*	*	*	*	*	*	*	*	*	*	*
1	1	1	chromosome segregation protein	ATCC29328_10140	*	*	*	*	*	*	*	*	*	*	*
5	5	1	Helix-turn-helix domain protein	ATCC29328_10150	ATCC53516_17250	*	08T492_15520	*	*	*	*	*	CCUG54800_14950	*	T160124_14080
9	11	0.182	hypothetical protein	ATCC29328_10160	ATCC53516_17260	07T609_09420	*	09T408_13870	09T494_16220	12T272_12740	12T273_13060	12T306_11240	CCUG54800_10490,CCUG548	*	*
10	14	0.101	hypothetical protein	ATCC29328_10170	ATCC53516_17270	07T609_09430	08T492_13550	09T408_13880	09T494_13950,09T494_	12T272_17420	12T273_13090,12T273_	12T306_11250	CCUG54800_10760,CCUG548	*	*
4	4	1	hypothetical protein	ATCC29328_10180	ATCC53516_17280	07T609_09440	*	*	*	*	*	*	CCUG54800_10770	*	*
8	9	1	Modification methylase HhaI	ATCC29328_10190	ATCC53516_17290	07T609_09450	08T492_09440	*	09T494_16240	*	12T273_13110	12T306_11260	CCUG54800_10780,CCUG548	*	*
10	16	0.107	DNA topoisomerase 3	ATCC29328_10200,ATCC2932	ATCC53516_17300	07T609_09460	08T492_13570,08T492_09	09T408_13900	09T494_13970,09T494_	12T272_17440	12T273_13120,12T273_	12T306_11270	CCUG54800_10790,CCUG548	*	*
9	13	0.265	hypothetical protein	ATCC29328_10210	ATCC53516_17310	07T609_09470	08T492_13600,08T492_09	*	09T494_13980,09T494_	12T272_17450	12T273_16840,12T273_	12T306_11290	CCUG54800_10820,CCUG548	*	*
8	8	0.75	chromosome segregation protein	ATCC29328_10220	ATCC53516_17330	07T609_09480	08T492_13610	*	09T494_13990	12T272_17460	12T273_16850	*	CCUG54800_10640	*	*
8	8	1	hypothetical protein	ATCC29328_10230	ATCC53516_17340	07T609_09490	08T492_09500	*	09T494_10940	*	12T273_13170	12T306_11310	CCUG54800_10840	*	*
9	9	0.778	putative endopeptidase p60 precursor	ATCC29328_10240	ATCC53516_17380	07T609_09500	08T492_09510	09T408_13930	09T494_10950	*	12T273_13180	12T306_11320	CCUG54800_10850	*	*
10	15	0.112	AAA-like domain protein	ATCC29328_10250,ATCC2932	ATCC53516_17390	07T609_09510	08T492_13650,08T492_09	09T408_13940	09T494_14030,09T494_	12T272_17500	12T273_16890,12T273_	12T306_11330	CCUG54800_10860,CCUG548	*	*
8	8	1	hypothetical protein	ATCC29328_10260	ATCC53516_17400	07T609_09530	08T492_09530	*	09T494_10970	*	12T273_13200	12T306_11340	CCUG54800_10870	*	*
10	14	0.243	hypothetical protein	ATCC29328_10270	ATCC53516_17410	07T609_09540	08T492_13680,08T492_09	09T408_13960	09T494_14060,09T494_	12T272_17530	12T273_16920,12T273_	12T306_11350	CCUG54800_10880,CCUG548	*	*
10	15	0.183	Maff2 family protein	ATCC29328_10280	ATCC53516_17420	07T609_14550,07T609_	08T492_09690,08T492_13	09T408_13970	09T494_14070,09T494_	12T272_17540	12T273_16930,12T273_	12T306_11360	CCUG54800_10890,CCUG548	*	*
10	15	0.208	hypothetical protein	ATCC29328_10290	ATCC53516_17430	07T609_14560,07T609_	08T492_13700,08T492_09	09T408_13980	09T494_14080,09T494_	12T272_17550	12T273_16940,12T273_	12T306_11370	CCUG54800_10900,CCUG548	*	*
4	4	0.5	hypothetical protein	ATCC29328_10300	*	07T609_09570	08T492_09710	*	*	*	*	*	CCUG54800_10920	*	*
5	6	0.271	hypothetical protein	ATCC29328_10310	*	07T609_09590,07T609_	08T492_09720	*	*	*	12T273_13260	*	CCUG54800_10930	*	*
7	10	0.158	TraM recognition site of TraD and TraG	ATCC29328_10320,ATCC2932	*	07T609_09600	08T492_13720,08T492_09	09T408_13990	*	*	12T273_13310	12T306_11390	CCUG54800_10940,CCUG548	*	*
7	8	0.448	hypothetical protein	ATCC29328_10330	*	07T609_09610	08T492_13730,08T492_09	09T408_14000	*	*	12T273_13320	12T306_11400	CCUG54800_10950	*	*
7	8	0.335	ORF6N domain protein	ATCC29328_10340	*	07T609_09620	08T492_09750,08T492_09	09T408_14010	*	*	12T273_13330	12T306_11410	CCUG54800_10960	*	*
7	7	1	hypothetical protein	ATCC29328_10350	*	07T609_09630	08T492_09760	09T408_14020	*	*	12T273_13340	12T306_11420	CCUG54800_10970	*	*
2	4	0.191	hypothetical protein	ATCC29328_10360,ATCC2932	*	*	08T492_09570,08T492_13	*	*	*	*	*	*	*	*
7	9	0.221	Mobilization protein A	ATCC29328_10370	*	07T609_09650,07T609_	08T492_09780,08T492_18	09T408_14040	*	*	12T273_13360	12T306_11440	CCUG54800_10990	*	*
4	4	1	hypothetical protein	ATCC29328_10380	*	*	*	*	*	*	12T273_06360	12T306_11450	CCUG54800_03790	*	*
3	3	1	hypothetical protein	ATCC29328_10400	*	*	*	09T408_13790	*	*	12T273_16640	*	*	*	*
12	15	0.145	Ferrous iron transport protein B	ATCC29328_10410,ATCC2932	ATCC53516_03700	07T609_12290	08T492_12340	09T408_11730,09T408_	09T494_12920	12T272_10410	12T273_16650,12T273_	12T306_13900	CCUG54800_13570	T151023_11780	T160124_12070
3	3	1	FeoA domain protein	ATCC29328_10420	*	*	*	09T408_13810	*	*	12T273_16660	*	*	*	*
7	7	0.143	putative ABC transporter ATP-binding protein	ATCC29328_10430	*	07T609_09730	*	09T408_13820	09T494_15510	12T272_12510	12T273_16670	12T306_16100	*	*	*
1	1	1	Putative multidrug export ATP-binding/permea	ATCC29328_10440	*	*	*	*	*	*	*	*	*	*	*
3	3	1	Regulatory protein PchR	ATCC29328_10450	*	*	*	09T408_13840	*	*	12T273_16690	*	*	*	*
7	9	0.184	Recombinase	ATCC29328_10460	*	07T609_09760,07T609_	08T492_13930,08T492_09	09T408_14140	*	*	12T273_13450	12T306_11560	CCUG54800_11070	*	*
10	14	0.24	Type IV secretory system Conjugative DNA trar	ATCC29328_10470	ATCC53516_17450	07T609_09870	08T492_13940,08T492_09	09T408_14150	09T494_14100,09T494_	12T272_17570	12T273_16960,12T273_	12T306_11570	CCUG54800_11080,CCUG548	*	*
8	9	0.195	hypothetical protein	ATCC29328_10480	ATCC53516_17460	07T609_09880	08T492_09870	*	09T494_11070,09T494_	*	12T273_13470	12T306_11580	CCUG54800_11090	*	*
7	10	0.147	hypothetical protein	ATCC29328_10490	ATCC53516_17470	07T609_14480,07T609_	08T492_09880,08T492_18	09T408_14160	*	*	*	12T306_11590	CCUG54800_11100,CCUG548	*	*
10	15	0.104	Primosomal protein DnaI	ATCC29328_10500	ATCC53516_17480	07T609_09900	08T492_13970,08T492_09	09T408_14170	09T494_14130,09T494_	12T272_17600	12T273_13490,12T273_	12T306_11600	CCUG54800_11110,CCUG548	*	*
6	7	1	hypothetical protein	ATCC29328_10520	ATCC53516_17500	*	08T492_09910,08T492_09	*	*	*	12T273_13510	12T306_11620	CCUG54800_11130	*	*
12	12	1	Lysine--tRNA ligase	ATCC29328_10530	ATCC53516_17510	07T609_09920	08T492_09920	09T408_09410	09T494_10270	12T272_08060	12T273_13520	12T306_11630	CCUG54800_11140	T151023_09530	T160124_09550
12	12	1	Transcription elongation factor GreA	ATCC29328_10540	ATCC53516_17520	07T609_09930	08T492_09930	09T408_09420	09T494_10280	12T272_08070	12T273_13530	12T306_11640	CCUG54800_11150	T151023_09540	T160124_09560
12	12	1	putative tRNA-dihydrouridine synthase	ATCC29328_10550	ATCC53516_17530	07T609_09940	08T492_09940	09T408_09430	09T494_10290	12T272_08080	12T273_13540	12T306_11650	CCUG54800_11160	T151023_09550	T160124_09570
12	12	1	Type III pantothenate kinase	ATCC29328_10560	ATCC53516_17540	07T609_09950	08T492_09950	09T408_09440	09T494_10300	12T272_08090	12T273_13550	12T306_11660	CCUG54800_11170	T151023_09560	T160124_09580
12	12	1	CTP synthase	ATCC29328_10570	ATCC53516_17550	07T609_09960	08T492_09960	09T408_09450	09T494_10310	12T272_08100	12T273_13560	12T306_11670	CCUG54800_11180	T151023_09570	T160124_09590
12	12	1	Glutamine synthetase	ATCC29328_10580	ATCC53516_17560	07T609_09970	08T492_09970	09T408_09460	09T494_10320	12T272_08110	12T273_13570	12T306_11680	CCUG54800_11190	T151023_09580	T160124_09600
12	12	1	hypothetical protein	ATCC29328_10590	ATCC53516_17570	07T609_09980	08T492_09980	09T408_09470	09T494_10330	12T272_08120	12T273_13580	12T306_11690	CCUG54800_11200	T151023_09590	T160124_09610
12	12	1	tRNA(Ile)-lysidine synthase	ATCC29328_10620	ATCC53516_17600	07T609_10010	08T492_10010	09T408_09500	09T494_10360	12T272_08150	12T273_13610	12T306_11720	CCUG54800_11230	T151023_09620	T160124_09640
12	12	1	General stress protein 13	ATCC29328_10630	ATCC53516_17610	07T609_10020	08T492_10020	09T408_09510	09T494_10370	12T272_08160	12T273_13620	12T306_11730	CCUG54800_11240	T151023_09630	T160124_09650
12	12	1	Cell division protein FtsL	ATCC29328_10640	ATCC53516_17620	07T609_10030	08T492_10030	09T408_09520	09T494_10380	12T272_08170	12T273_13630	12T306_11740	CCUG54800_11250	T151023_09640	T160124_09660
12	12	1	S4 domain protein	ATCC29328_10650	ATCC53516_17630	07T609_10040	08T492_10040	09T408_09530	09T494_10390	12T272_08180	12T273_13640	12T306_11750	CCUG54800_11260	T151023_09650	T160124_09670
12	12	1	Phosphorylated carbohydrates phosphatase	ATCC29328_10660	ATCC53516_17640	07T609_10050	08T492_10050	09T408_09540	09T494_10400	12T272_08190	12T273_13650	12T306_11760	CCUG54800_11270	T151023_09660	T160124_09680
12	12	1	Pyruvate kinase	ATCC29328_10670	ATCC53516_17650	07T609_10060	08T492_10060	09T408_09550	09T494_10410	12T272_08200	12T273_13660	12T306_11770	CCUG54800_11280	T151023_09670	T160124_09690
12	12	1	6-phosphofructokinase	ATCC29328_10680	ATCC53516_17660	07T609_10070	08T492_10070	09T408_09560	09T494_10420	12T272_08210	12T273_13670	12T306_11780	CCUG54800_11290	T151023_09680	T160124_09700
12	12	1	DNA polymerase III subunit alpha	ATCC29328_10690	ATCC53516_17670	07T609_10080	08T492_10080	09T408_09570	09T494_10430	12T272_08220	12T273_13680	12T306_11790	CCUG54800_11300	T151023_09690	T160124_09710
12	12	1	Putative sporulation transcription regulator W	ATCC29328_10700	ATCC53516_17680	07T609_10090	08T492_10090	09T408_09580	09T494_10440	12T272_08230	12T273_13690	12T306_11800	CCUG54800_11310	T151023_09700	T160124_09720
12	12	1	Diadenosine hexaphosphate hydrolase	ATCC29328_10710	ATCC53516_17690	07T609_10100	08T492_10100	09T408_09590	09T494_10450	12T272_08240	12T273_13700	12T306_11810	CCUG54800_11320	T151023_09710	T160124_09730
12	12	1	hypothetical protein	ATCC29328_10720	ATCC53516_17700	07T609_10110	08T492_10110	09T408_09600	09T494_10460	12T272_08250	12T273_13710	12T306_11820	CCUG54800_11330	T151023_09720	T160124_09740
12	12	1	TelA-like protein	ATCC29328_10730	ATCC53516_17710	07T609_10120	08T492_10120	09T408_09610	09T494_10470	12T272_08260	12T273_13720	12T306_11830	CCUG54800_11340	T151023_09730	T160124_09750
12	12	1	hypothetical protein	ATCC29328_10740	ATCC53516_17720	07T609_10130	08T492_10130	09T408_09620	09T494_10480	12T272_08270	12T273_13730	12T306_11840	CCUG54800_11350	T151023_09740	T160124_09760
11	11	1	hypothetical protein	ATCC29328_10750	ATCC53516_17730	07T609_10140	08T492_10140	09T408_09630	09T494_10490	12T272_08290	12T273_13740	12T306_11850	CCUG54800_11360	T151023_09750	*
12	12	1	Branched-chain amino acid transport system /	ATCC29328_10760	ATCC53516_17740	07T609_10150	08T492_10150	09T408_09640	09T494_10500	12T272_08300	12T273_13750	12T306_11870	CCUG54800_11370	T151023_09760	T160124_09770
12	12	1	beta-methylgalactoside transporter inner mem	ATCC29328_10770	ATCC53516_17750	07T609_10160	08T492_10160	09T408_09650	09T494_10510	12T272_08310	12T273_13760	12T306_11880	CCUG54800_11380	T151023_09770	T160124_09780
12	12	1	Galactose/methyl galactoside import ATP-bind	ATCC29328_10780	ATCC53516_17760	07T609_10170	08T492_10170	09T408_09660	09T494_10520	12T272_08320	12T273_13770	12T306_11890	CCUG54800_11390	T151023_09780	T160124_09790
12	12	1	Membrane lipoprotein TmpC precursor	ATCC29328_10790	ATCC53516_17770	07T609_10180	08T492_10180	09T408_09670	09T494_10530	12T272_08330	12T273_13780	12T306_11900	CCUG54800_11400	T151023_09790	T160124_09800
12	12	1	NADH dehydrogenase-like protein	ATCC29328_10800	ATCC53516_17780	07T609_10190	08T492_10190	09T408_09680	09T494_10540	12T272_08340	12T273_13790	12T306_11910	CCUG54800_11410	T151023_09800	T160124_09810
12	24	0.167	Uric acid permease PucK	ATCC29328_10810,ATCC2932	ATCC53516_18400,ATCC5351	07T609_10780,07T609_	08T492_10700,08T492_10	09T408_10290,09T408_	09T494_11480,09T494_	12T272_08940,12T272_	12T273_14410,12T273_	12T306_12420,12T306_	CCUG54800_11420,CCUG548	T151023_10310,T151023_	T160124_10430,T160124_09820
12	12	1	Carbamate kinase 1	ATCC29328_10820	ATCC53516_17800	07T609_10210	08T492_10210	09T408_09710	09T494_10560	12T272_08360	12T273_13810	12T306_11930	CCUG54800_11430	T151023_09820	T160124_09830
12	12	1	DNA-binding transcriptional regulator ModE	ATCC29328_10830	ATCC53516_17810	07T609_10220	08T492_10220	09T408_09720	09T494_10570	12T272_08370	12T273_13820	12T306_11940	CCUG54800_11440	T151023_09830	T160124_09840
12	12	1	hypothetical protein	ATCC29328_10840	ATCC53516_17820	07T609_10230	08T492_10230	09T408_09730	09T494_10580	12T272_08380	12T273_13830	12T306_11950	CCUG54800_11450	T151023_09840	T160124_09850
12	12	1	HPr-like protein Crh	ATCC29328_10860	ATCC53516_17840	07T609_10250	08T492_10250	09T408_09750	09T494_10600	12T272_08400	12T273_13850	12T306_11970	CCUG54800_11470	T151023_09860	T160124_09870
12	12	1	Catabolite control protein A	ATCC29328_10870	ATCC53516_17850	07T609_10260	08T492_10260	09T408_09760	09T494_10610	12T272_08410	12T273_13860	12T306_11980	CCUG54800_11480	T151023_09870	T160124_09880
12	12	1	Diacylglycerol kinase	ATCC29328_10880	ATCC53516_17860	07T609_10270	08T492_10270	09T408_09770	09T494_10620	12T272_08420	12T273_13870	12T306_11990	CCUG54800_11490	T151023_09880	T160124_09890
12	12	1	hypothetical protein	ATCC29328_10890	ATCC53516_17870	07T609_10280	08T492_10280	09T408_09780	09T494_10630	12T272_08430	12T273_13880	12T306_12000	CCUG54800_11500	T151023_09890	T160124_09900
12	12	1	YtxH-like protein	ATCC29328_10900	ATCC53516_17880	07T609_10290	08T492_10290	09T408_09790	09T494_10640	12T272_08440	12T273_13890	12T306_12010	CCUG54800_11510	T151023_09900	T160124_09910
12	12	1	hypothetical protein	ATCC29328_10910	ATCC53516_17890	07T609_10300	08T492_10300	09T408_09800	09T494_10650	12T272_08450	12T273_13900	12T306_12020	CCUG54800_11520	T151023_09910	T160124_09920
12	12	1	Ribosomal large subunit pseudouridine syntha	ATCC29328_10920	ATCC53516_17900	07T609_10310	08T492_10310	09T408_09810	09T494_10660	12T272_08460	12T273_13910	12T306_12030	CCUG54800_11530	T151023_09920	T160124_09930
12	12	1	putative inorganic polyphosphate/ATP-NAD ki	ATCC29328_10930	ATCC53516_17910	07T609_10320	08T492_10320	09T408_09820	09T494_10670	12T272_08470	12T273_13920	12T306_12040	CCUG54800_11540	T151023_09930	T160124_09940
12	12	1	Penicillin-binding protein A	ATCC29328_10940	ATCC53516_17920	07T609_10330	08T492_10330	09T408_09830	09T494_10680	12T272_08480	12T273_13930	12T306_12050	CCUG54800_11550	T151023_09940	T160124_09950
12	12	1	Lipid II flippase FtsW	ATCC29328_10950	ATCC53516_17930	07T609_10340	08T492_10340	09T408_09840	09T494_10690	12T272_08490	12T273_13940	12T306_12060	CCUG54800_11560	T151023_09950	T160124_09960
12	12	1	FHA domain-containing protein FhaB	ATCC29328_10960	ATCC53516_17940	07T609_10350	08T492_10350	09T408_09850	09T494_10700	12T272_08500	12T273_13950	12T306_12070	CCUG54800_11570	T151023_09960	T160124_09970
12	12	1	Xanthine phosphoribosyltransferase	ATCC29328_10970	ATCC53516_17950	07T609_10360	08T492_10360	09T408_09860	09T494_10710	12T272_08510	12T273_13960	12T306_12080	CCUG54800_11580	T151023_09970	T160124_09980
12	12	1	Phosphoglucomutase	ATCC29328_10980	ATCC53516_17960	07T609_10370	08T492_10370	09T408_09870	09T494_10720	12T272_08520	12T273_13970	12T306_12090	CCUG54800_11590	T151023_09980	T160124_09990
12	12	1	hypothetical protein	ATCC29328_10990	ATCC53516_17970	07T609_10380	08T492_10380	09T408_09880	09T494_10730	12T272_08530	12T273_13980	12T306_12100	CCUG54800_11600	T151023_09990	T160124_10000
12	12	1	Pullulanase precursor	ATCC29328_11000	ATCC53516_17980	07T609_10390	08T492_10390	09T408_09890	09T494_10740	12T272_08550	12T273_13990	12T306_12110	CCUG54800_11610	T151023_10000	T160124_10010
12	12	1	Glycogen phosphorylase	ATCC29328_11010	ATCC53516_17990	07T609_10400	08T492_10400	09T408_09900	09T494_10750	12T272_08560	12T273_14000	12T306_12120	CCUG54800_11620	T151023_10010	T160124_10020
12	12	1	Glycogen synthase	ATCC29328_11020	ATCC53516_18000	07T609_10410	08T492_10410	09T408_09920	09T494_10760	12T272_08570	12T273_14010	12T306_12130	CCUG54800_11630	T151023_10020	T160124_10030
12	12	1	Glycogen biosynthesis protein GlgD	ATCC29328_11030	ATCC53516_18010	07T609_10420	08T492_10420	09T408_09930	09T494_10770	12T272_08580	12T273_14020	12T306_12140	CCUG54800_11640	T151023_10030	T160124_10040
12	12	1	Glucose-1-phosphate adenylyltransferase	ATCC29328_11040	ATCC53516_18020	07T609_10430	08T492_10430	09T408_09940	09T494_10780	12T272_08590	12T273_14030	12T306_12150	CCUG54800_11650	T151023_10040	T160124_10050
12	12	1	1,4-alpha-glucan branching enzyme GlgB	ATCC29328_11050	ATCC53516_18030	07T609_10440	08T492_10440	09T408_09950	09T494_10790	12T272_08600	12T273_14040	12T306_12160	CCUG54800_11660	T151023_10050	T160124_10060
12	12	1	Ribonuclease	ATCC29328_11060	ATCC53516_18040	07T609_10450	08T492_10450	09T408_09960	09T494_10800	12T272_08610	12T273_14050	12T306_12170	CCUG54800_11670	T151023_10060	T160124_10070
12	12	1	Trp repressor protein	ATCC29328_11070	ATCC53516_18050	07T609_10460	08T492_10460	09T408_09970	09T494_10810	12T272_08620	12T273_14060	12T306_12180	CCUG54800_11680	T151023_10070	T160124_10080
12	12	1	anti-sigma F factor	ATCC29328_11090	ATCC53516_18070	07T609_10480	08T492_10480	09T408_09990	09T494_10830	12T272_08640	12T273_14080	12T306_12200	CCUG54800_11700	T151023_10090	T160124_10100
12	12	1	Rubredoxin	ATCC29328_11100	ATCC53516_18080	07T609_10490	08T492_10490	09T408_10000	09T494_10840	12T272_08650	12T273_14090	12T306_12210	CCUG54800_11710	T151023_10100	T160124_10110
12	12	1	putative GTP-binding protein EngB	ATCC29328_11110	ATCC53516_18090	07T609_10500	08T492_10500	09T408_10010	09T494_10850	12T272_08660	12T273_14100	12T306_12220	CCUG54800_11720	T151023_10110	T160124_10120
12	12	1	Ferritin-1	ATCC29328_11120	ATCC53516_18180	07T609_10590	08T492_10510	09T408_10100	09T494_11260	12T272_08750	12T273_14190	12T306_12230	CCUG54800_11810	T151023_10120	T160124_10210
12	12	1	hypothetical protein	ATCC29328_11130	ATCC53516_18190	07T609_10600	08T492_10520	09T408_10110	09T494_11270	12T272_08760	12T273_14200	12T306_12240	CCUG54800_11820	T151023_10130	T160124_10220
12	12	1	Peptidase family S41	ATCC29328_11140	ATCC53516_18200	07T609_10610	08T492_10530	09T408_10120	09T494_11280	12T272_08770	12T273_14210	12T306_12250	CCUG54800_11830	T151023_10140	T160124_10230
12	12	1	Organic hydroperoxide resistance transcriptior	ATCC29328_11150	ATCC53516_18230	07T609_10620	08T492_10540	09T408_10130	09T494_11310	12T272_08780	12T273_14250	12T306_12260	CCUG54800_11860	T151023_10150	T160124_10270
12	12	1	Branched-chain-amino-acid aminotransferase	ATCC29328_11160	ATCC53516_18240	07T609_10630	08T492_10550	09T408_10140	09T494_11320	12T272_08790	12T273_14260	12T306_12270	CCUG54800_11870	T151023_10160	T160124_10280
12	12	1	Aminodeoxychorismate synthase component :	ATCC29328_11170	ATCC53516_18250	07T609_10640	08T492_10560	09T408_10150	09T494_11330	12T272_08800	12T273_14270	12T306_12280	CCUG54800_11880	T151023_10170	T160124_10290
12	12	1	Aminodeoxychorismate/anthranilate synthase	ATCC29328_11180	ATCC53516_18260	07T609_10650	08T492_10570	09T408_10160	09T494_11340	12T272_08810	12T273_14280	12T306_12290	CCUG54800_11890	T151023_10180	T160124_10300
12	12	1	Macrolide export ATP-binding/permease prote	ATCC29328_11190	ATCC53516_18270	07T609_10660	08T492_10580	09T408_10170	09T494_11350	12T272_08820	12T273_14290	12T306_12300	CCUG54800_11900	T151023_10190	T160124_10310
12	17	0.149	Macrolide export ATP-binding/permease prote	ATCC29328_11200	ATCC53516_18360,ATCC5351	07T609_10670	08T492_10590	09T408_10260,09T408_	09T494_11360	12T272_08910,12T272_	12T273_14380,12T273_	12T306_12310	CCUG54800_11910	T151023_10200,T151023_	T160124_10320
12	12	1	Putative efflux system component YknX	ATCC29328_11210	ATCC53516_18290	07T609_10680	08T492_10600	09T408_10190	09T494_11370	12T272_08840	12T273_14310	12T306_12320	CCUG54800_11920	T151023_10210	T160124_10330
12	12	1	Cytosol non-specific dipeptidase	ATCC29328_11220	ATCC53516_18300	07T609_10690	08T492_10610	09T408_10200	09T494_11380	12T272_08850	12T273_14320	12T306_12330	CCUG54800_11930	T151023_10220	T160124_10340
12	12	1	Yqey-like protein	ATCC29328_11230	ATCC53516_18310	07T609_10700	08T492_10620	09T408_10210	09T494_11390	12T272_08860	12T273_14330	12T306_12340	CCUG54800_11940	T151023_10230	T160124_10350
12	24	0.167	phosphatidylglycerophosphatase B	ATCC29328_11240,ATCC2932	ATCC53516_18330,ATCC5351	07T609_10720,07T609_	08T492_10640,08T492_10	09T408_10230,09T408_	09T494_11410,09T494_	12T272_08880,12T272_	12T273_14350,12T273_	12T306_12360,12T306_	CCUG54800_11950,CCUG548	T151023_10250,T151023_	T160124_10370,T160124_10360
12	12	1	bifunctional molybdenum cofactor biosynthesi	ATCC29328_11260	ATCC53516_18340	07T609_10730	08T492_10650	09T408_10240	09T494_11420	12T272_08890	12T273_14360	12T306_12370	CCUG54800_11970	T151023_10260	T160124_10380
12	12	1	ABC transporter permease protein YxdM	ATCC29328_11270	ATCC53516_18350	07T609_10740	08T492_10660	09T408_10250	09T494_11440	12T272_08900	12T273_14370	12T306_12380	CCUG54800_11980	T151023_10270	T160124_10390
12	12	1	Sensor histidine kinase GraS	ATCC29328_11290	ATCC53516_18370	07T609_10760	08T492_10680	09T408_10270	09T494_11460	12T272_08920	12T273_14390	12T306_12400	CCUG54800_12000	T151023_10290	T160124_10410
12	12	1	Response regulator protein GraR	ATCC29328_11300	ATCC53516_18380	07T609_10770	08T492_10690	09T408_10280	09T494_11470	12T272_08930	12T273_14400	12T306_12410	CCUG54800_12010	T151023_10300	T160124_10420
12	12	1	Peroxide operon regulator	ATCC29328_11320	ATCC53516_18410	07T609_10790	08T492_10710	09T408_10300	09T494_11490	12T272_08950	12T273_14420	12T306_12430	CCUG54800_12030	T151023_10320	T160124_10440
12	12	1	Regulatory protein SoxS	ATCC29328_11330	ATCC53516_18420	07T609_10800	08T492_10720	09T408_10310	09T494_11500	12T272_08960	12T273_14430	12T306_12440	CCUG54800_12040	T151023_10330	T160124_10450
11	11	1	hypothetical protein	ATCC29328_11340	ATCC53516_18430	07T609_10810	08T492_10730	09T408_10320	09T494_11510	*	12T273_14440	12T306_12450	CCUG54800_12050	T151023_10340	T160124_10460
7	7	1	putative ABC transporter ATP-binding protein	ATCC29328_11350	ATCC53516_18440	07T609_10820	08T492_10740	09T408_10330	*	*	*	12T306_00620	CCUG54800_00230	*	*
7	7	1	Multidrug resistance ABC transporter ATP-binc	ATCC29328_11360	ATCC53516_18450	07T609_10830	08T492_10750	09T408_10340	*	*	*	12T306_00610	CCUG54800_00220	*	*
7	7	1	hypothetical protein	ATCC29328_11370	ATCC53516_18460	07T609_10840	08T492_10760	09T408_10350	*	*	*	12T306_00630	CCUG54800_00240	*	*
12	12	1	V-type sodium ATPase subunit D	ATCC29328_11380	ATCC53516_18470	07T609_10850	08T492_10770	09T408_10360	09T494_11520	12T272_08980	12T273_14450	12T306_12460	CCUG54800_12060	T151023_10350	T160124_10470
12	24	0.167	V-type sodium ATPase subunit B	ATCC29328_11390,ATCC2932	ATCC53516_03200,ATCC5351	07T609_11800,07T609_	08T492_11710,08T492_10	09T408_11290,09T408_	09T494_12440,09T494_	12T272_09970,12T272_	12T273_15400,12T273_	12T306_13400,12T306_	CCUG54800_12070,CCUG548	T151023_11290,T151023_	T160124_11430,T160124_10480
12	24	0.167	V-type sodium ATPase catalytic subunit A	ATCC29328_11400,ATCC2932	ATCC53516_03210,ATCC5351	07T609_11810,07T609_	08T492_11720,08T492_10	09T408_11300,09T408_	09T494_12450,09T494_	12T272_09980,12T272_	12T273_15410,12T273_	12T306_13410,12T306_	CCUG54800_12080,CCUG548	T151023_11300,T151023_	T160124_11440,T160124_10490
12	12	1	V-type sodium ATPase subunit G	ATCC29328_11410	ATCC53516_18500	07T609_10880	08T492_10800	09T408_10390	09T494_11550	12T272_09010	12T273_14480	12T306_12490	CCUG54800_12090	T151023_10380	T160124_10500
12	12	1	V-type sodium ATPase subunit C	ATCC29328_11420	ATCC53516_18510	07T609_10890	08T492_10810	09T408_10400	09T494_11560	12T272_09020	12T273_14490	12T306_12500	CCUG54800_12100	T151023_10390	T160124_10510
12	12	1	V-type ATP synthase subunit E	ATCC29328_11430	ATCC53516_18520	07T609_10900	08T492_10820	09T408_10410	09T494_11570	12T272_09030	12T273_14500	12T306_12510	CCUG54800_12110	T151023_10400	T160124_10520
12	12	1	V-type sodium ATPase subunit K	ATCC29328_11440	ATCC53516_18530	07T609_10910	08T492_10830	09T408_10420	09T494_11580	12T272_09040	12T273_14510	12T306_12520	CCUG54800_12120	T151023_10410	T160124_10530
12	12	1	V-type ATP synthase subunit I	ATCC29328_11450	ATCC53516_18540	07T609_10920	08T492_10840	09T408_10430	09T494_11590	12T272_09050	12T273_14520	12T306_12530	CCUG54800_12130	T151023_10420	T160124_10540
12	12	1	hypothetical protein	ATCC29328_11460	ATCC53516_18550	07T609_10930	08T492_10850	09T408_10440	09T494_11600	12T272_09060	12T273_14530	12T306_12540	CCUG54800_12140	T151023_10430	T160124_10550
12	12	1	Uracil phosphoribosyltransferase	ATCC29328_11470	ATCC53516_18560	07T609_10940	08T492_10860	09T408_10450	09T494_11610	12T272_09070	12T273_14540	12T306_12550	CCUG54800_12150	T151023_10440	T160124_10560
12	12	1	Threonylcarbamoyl-AMP synthase	ATCC29328_11480	ATCC53516_18570	07T609_10950	08T492_10870	09T408_10460	09T494_11620	12T272_09080	12T273_14550	12T306_12560	CCUG54800_12160	T151023_10450	T160124_10570
12	12	1	Gluconeogenesis factor	ATCC29328_11490	ATCC53516_18580	07T609_10960	08T492_10880	09T408_10470	09T494_11630	12T272_09090	12T273_14560	12T306_12570	CCUG54800_12170	T151023_10460	T160124_10580
12	12	1	glmZ(sRNA)-inactivating NTPase	ATCC29328_11500	ATCC53516_18590	07T609_10970	08T492_10890	09T408_10480	09T494_11640	12T272_09100	12T273_14570	12T306_12580	CCUG54800_12180	T151023_10470	T160124_10590
12	12	1	putative phosphatase YcdX	ATCC29328_11510	ATCC53516_18600	07T609_10980	08T492_10900	09T408_10490	09T494_11650	12T272_09110	12T273_14580	12T306_12590	CCUG54800_12190	T151023_10480	T160124_10600

12	12	1	UDP-N-acetylenolpyruvoylglucosamine reducta	ATCC29328_11520	ATCC53516_18610	07T609_10990	08T492_10910	09T408_10500	09T494_11660	12T272_09120	12T273_14590	12T306_12600	CCUG54800_12200	T151023_10490	T160124_10610
12	12	1	Folylpolyglutamate synthase	ATCC29328_11530	ATCC53516_18620	07T609_11000	08T492_10920	09T408_10510	09T494_11670	12T272_09130	12T273_14600	12T306_12610	CCUG54800_12210	T151023_10500	T160124_10620
12	24	0.167	Peptide chain release factor 1	ATCC29328_11540,ATCC2932	ATCC53516_12360,ATCC5351	07T609_15650,07T609_	08T492_16670,08T492_1	09T408_15490,09T408_	09T494_18350,09T494_	12T272_15850,12T272	12T273_18250,12T273	12T306_16570,12T306	CCUG54800_12220,CCUG548	T151023_15130,T151023_	T160124_15570,T160124_10630
12	12	1	Release factor glutamine methyltransferase	ATCC29328_11550	ATCC53516_18640	07T609_11020	08T492_10940	09T408_10530	09T494_11690	12T272_09150	12T273_14620	12T306_12630	CCUG54800_12230	T151023_10520	T160124_10640
12	12	1	hypothetical protein	ATCC29328_11560	ATCC53516_18650	07T609_11030	08T492_10950	09T408_10540	09T494_11700	12T272_09160	12T273_14630	12T306_12640	CCUG54800_12240	T151023_10530	T160124_10650
12	12	1	50S ribosomal protein L31	ATCC29328_11570	ATCC53516_18660	07T609_11040	08T492_10960	09T408_10550	09T494_11710	12T272_09170	12T273_14640	12T306_12650	CCUG54800_12250	T151023_10540	T160124_10660
12	12	1	hypothetical protein	ATCC29328_11580	ATCC53516_18670	07T609_11050	08T492_10970	09T408_10560	09T494_11720	12T272_09180	12T273_14650	12T306_12660	CCUG54800_12260	T151023_10550	T160124_10670
12	12	1	Thiol:disulfide interchange protein DsbD precu	ATCC29328_11590	ATCC53516_18680	07T609_11060	08T492_10980	09T408_10570	09T494_11730	12T272_09190	12T273_14660	12T306_12670	CCUG54800_12270	T151023_10560	T160124_10680
12	12	1	Thioredoxin	ATCC29328_11600	ATCC53516_18690	07T609_11070	08T492_10990	09T408_10580	09T494_11740	12T272_09200	12T273_14670	12T306_12680	CCUG54800_12280	T151023_10570	T160124_10690
12	12	1	hypothetical protein	ATCC29328_11650	ATCC53516_02490	07T609_11090	08T492_11010	09T408_10590	09T494_11760	12T272_09230	12T273_14690	12T306_12700	CCUG54800_12290	T151023_10590	T160124_10710
12	12	1	hypothetical protein	ATCC29328_11660	ATCC53516_02500	07T609_11100	08T492_11020	09T408_10600	09T494_11770	12T272_09240	12T273_14700	12T306_12710	CCUG54800_12300	T151023_10600	T160124_10720
12	12	1	nitroreductase A	ATCC29328_11670	ATCC53516_02510	07T609_11110	08T492_11030	09T408_10610	09T494_11780	12T272_09250	12T273_14710	12T306_12720	CCUG54800_12310	T151023_10610	T160124_10730
12	12	1	Na(+)-translocating NADH-quinone reductase s	ATCC29328_11680	ATCC53516_02520	07T609_11120	08T492_11040	09T408_10620	09T494_11790	12T272_09260	12T273_14720	12T306_12730	CCUG54800_12320	T151023_10620	T160124_10740
12	12	1	Electron transport complex protein RnfG	ATCC29328_11710	ATCC53516_02550	07T609_11150	08T492_11070	09T408_10650	09T494_11820	12T272_09290	12T273_14750	12T306_12760	CCUG54800_12350	T151023_10650	T160124_10770
12	12	1	hypothetical protein	ATCC29328_11730	ATCC53516_02570	07T609_11170	08T492_11090	09T408_10670	09T494_11840	12T272_09310	12T273_14770	12T306_12780	CCUG54800_12370	T151023_10670	T160124_10790
8	8	1	hypothetical protein	ATCC29328_11750	*	07T609_11190	08T492_11110	09T408_10690	*	*	12T273_14790	*	CCUG54800_12390	T151023_10690	T160124_10810
12	12	1	N-acetylmuramoyl-L-alanine amidase LytC pre	ATCC29328_11760	ATCC53516_02590	07T609_11200	08T492_11120	09T408_10700	09T494_11860	12T272_09330	12T273_14800	12T306_12800	CCUG54800_12400	T151023_10700	T160124_10820
12	12	1	CAMP factor (Cfa)	ATCC29328_11770	ATCC53516_02600	07T609_11210	08T492_11130	09T408_10710	09T494_11870	12T272_09390	12T273_14810	12T306_12810	CCUG54800_12410	T151023_10710	T160124_10830
12	12	1	CAMP factor (Cfa)	ATCC29328_11780	ATCC53516_02610	07T609_11220	08T492_11140	09T408_10720	09T494_11880	12T272_09400	12T273_14820	12T306_12820	CCUG54800_12420	T151023_10720	T160124_10840
12	24	0.147	Papain family cysteine protease	ATCC29328_11790,ATCC2932	ATCC53516_02630,ATCC5351	07T609_11240,07T609_	08T492_11160,08T492_11	09T408_10740,09T408_	09T494_11900,09T494_	12T272_09420,12T272	12T273_14840,12T273	12T306_12840,12T306	CCUG54800_12430,CCUG548	T151023_10740,T151023_	T160124_10860,T160124_10850
12	12	1	Arginine transport ATP-binding protein ArtM	ATCC29328_11810	ATCC53516_02640	07T609_11250	08T492_11170	09T408_10750	09T494_11910	12T272_09430	12T273_14850	12T306_12850	CCUG54800_12450	T151023_10750	T160124_10870
12	12	1	Inner membrane amino-acid ABC transporter p	ATCC29328_11820	ATCC53516_02650	07T609_11260	08T492_11180	09T408_10760	09T494_11920	12T272_09440	12T273_14860	12T306_12860	CCUG54800_12460	T151023_10760	T160124_10880
12	12	1	tRNA pseudouridine synthase A	ATCC29328_11830	ATCC53516_02660	07T609_11270	08T492_11190	09T408_10770	09T494_11930	12T272_09450	12T273_14870	12T306_12870	CCUG54800_12470	T151023_10770	T160124_10890
12	12	1	Energy-coupling factor transporter transmemb	ATCC29328_11840	ATCC53516_02670	07T609_11280	08T492_11200	09T408_10780	09T494_11940	12T272_09460	12T273_14880	12T306_12880	CCUG54800_12480	T151023_10780	T160124_10900
12	24	0.167	Energy-coupling factor transporter ATP-bindin	ATCC29328_11850,ATCC2932	ATCC53516_02690,ATCC5351	07T609_11300,07T609_	08T492_11220,08T492_11	09T408_10800,09T408_	09T494_11960,09T494_	12T272_09480,12T272	12T273_14900,12T273	12T306_12900,12T306	CCUG54800_12490,CCUG548	T151023_10800,T151023_	T160124_10920,T160124_10910
12	12	1	DNA-directed RNA polymerase subunit beta'	ATCC29328_11870	ATCC53516_02700	07T609_11310	08T492_11230	09T408_10810	09T494_11970	12T272_09490	12T273_14910	12T306_12910	CCUG54800_12510	T151023_10810	T160124_10930
12	12	1	DNA-directed RNA polymerase subunit beta	ATCC29328_11880	ATCC53516_02710	07T609_11320	08T492_11240	09T408_10820	09T494_11980	12T272_09500	12T273_14920	12T306_12920	CCUG54800_12520	T151023_10820	T160124_10940
12	12	1	Acyl-ACP thioesterase	ATCC29328_11890	ATCC53516_02720	07T609_11330	08T492_11250	09T408_10830	09T494_11990	12T272_09510	12T273_14930	12T306_12930	CCUG54800_12530	T151023_10830	T160124_10950
12	12	1	AAA-like domain protein	ATCC29328_11900	ATCC53516_02730	07T609_11340	08T492_11260	09T408_10840	09T494_12000	12T272_09520	12T273_14940	12T306_12940	CCUG54800_12540	T151023_10840	T160124_10960
12	12	1	NurA domain protein	ATCC29328_11910	ATCC53516_02740	07T609_11350	08T492_11270	09T408_10850	09T494_12010	12T272_09530	12T273_14950	12T306_12950	CCUG54800_12550	T151023_10850	T160124_10970
12	12	1	Ribosomal RNA small subunit methyltransfera	ATCC29328_11920	ATCC53516_02750	07T609_11360	08T492_11280	09T408_10860	09T494_12020	12T272_09540	12T273_14960	12T306_12960	CCUG54800_12560	T151023_10860	T160124_10980
12	12	1	N5-glutamine S-adenosyl-L-methionine-depen	ATCC29328_11930	ATCC53516_02760	07T609_11370	08T492_11290	09T408_10870	09T494_12030	12T272_09550	12T273_14970	12T306_12970	CCUG54800_12570	T151023_10870	T160124_10990
12	12	1	Ferredoxin	ATCC29328_11940	ATCC53516_02770	07T609_11380	08T492_11300	09T408_10880	09T494_12040	12T272_09560	12T273_14980	12T306_12980	CCUG54800_12580	T151023_10880	T160124_11000
12	12	1	hypothetical protein	ATCC29328_11950	ATCC53516_02780	07T609_11390	08T492_11310	09T408_10890	09T494_12050	12T272_09570	12T273_14990	12T306_12990	CCUG54800_12590	T151023_10890	T160124_11010
12	12	1	DNA polymerase III subunit tau	ATCC29328_11960	ATCC53516_02790	07T609_11400	08T492_11320	09T408_10900	09T494_12060	12T272_09580	12T273_15000	12T306_13000	CCUG54800_12600	T151023_10900	T160124_11020
12	12	1	hypothetical protein	ATCC29328_11970	ATCC53516_02800	07T609_11410	08T492_11330	09T408_10910	09T494_12070	12T272_09590	12T273_15010	12T306_13010	CCUG54800_12610	T151023_10910	T160124_11030
12	12	1	hypothetical protein	ATCC29328_11980	ATCC53516_02810	07T609_11420	08T492_11340	09T408_10920	09T494_12080	12T272_09600	12T273_15020	12T306_13020	CCUG54800_12620	T151023_10920	T160124_11040
12	12	1	Thymidylate kinase	ATCC29328_11990	ATCC53516_02820	07T609_11430	08T492_11350	09T408_10930	09T494_12090	12T272_09610	12T273_15030	12T306_13030	CCUG54800_12630	T151023_10930	T160124_11050
12	12	1	50S ribosomal protein L25	ATCC29328_12000	ATCC53516_02830	07T609_11440	08T492_11360	09T408_10940	09T494_12100	12T272_09620	12T273_15040	12T306_13040	CCUG54800_12640	T151023_10940	T160124_11060
9	9	1	SigmaK-factor processing regulatory protein Bo	ATCC29328_12010	ATCC53516_02840	07T609_11450	08T492_11370	09T408_10950	*	12T272_09630	12T273_15050	*	CCUG54800_12650	*	T160124_11070
12	12	1	putative thiol peroxidase	ATCC29328_12020	ATCC53516_02850	07T609_11460	08T492_11380	09T408_10960	09T494_12120	12T272_09640	12T273_15060	12T306_13060	CCUG54800_12660	T151023_10960	T160124_11080
10	10	1	putative copper-exporting P-type ATPase V	ATCC29328_12030	ATCC53516_02860	07T609_11470	08T492_11390	09T408_10970	*	*	12T273_15070	12T306_13070	CCUG54800_12670	T151023_10970	T160124_11090
1	1	1	hypothetical protein	ATCC29328_12040	*	*	*	*	*	*	*	*	*	*	*
12	12	1	D-alanyl-D-alanine carboxypeptidase	ATCC29328_12060	ATCC53516_02890	07T609_11500	08T492_11420	09T408_11000	09T494_12140	12T272_09660	12T273_15100	12T306_13100	CCUG54800_12700	T151023_11000	T160124_11120
12	12	1	UDP-N-acetyl-alpha-D-glucosamine C6 dehydra	ATCC29328_12070	ATCC53516_02900	07T609_11510	08T492_11430	09T408_11010	09T494_12150	12T272_09670	12T273_15110	12T306_13110	CCUG54800_12710	T151023_11010	T160124_11130
12	12	1	Branched-chain-amino-acid aminotransferase	ATCC29328_12080	ATCC53516_02910	07T609_11520	08T492_11440	09T408_11020	09T494_12160	12T272_09680	12T273_15120	12T306_13120	CCUG54800_12720	T151023_11020	T160124_11140
12	12	1	30S ribosomal protein S18	ATCC29328_12090	ATCC53516_02920	07T609_11530	08T492_11450	09T408_11030	09T494_12170	12T272_09690	12T273_15130	12T306_13130	CCUG54800_12730	T151023_11030	T160124_11150
12	13	0.141	Single-stranded DNA-binding protein ssb	ATCC29328_12100	ATCC53516_02930	07T609_11540	08T492_11460	09T408_11040	09T494_12180	12T272_09700	12T273_15140	12T306_13140	CCUG54800_12740,CCUG548	T151023_11040	T160124_11160
12	12	1	30S ribosomal protein S6	ATCC29328_12110	ATCC53516_02940	07T609_11550	08T492_11470	09T408_11050	09T494_12190	12T272_09710	12T273_15150	12T306_13150	CCUG54800_12750	T151023_11050	T160124_11170
12	12	1	hypothetical protein	ATCC29328_12120	ATCC53516_02950	07T609_11560	08T492_11480	09T408_11060	09T494_12200	12T272_09720	12T273_15160	12T306_13160	CCUG54800_12760	T151023_11060	T160124_11180
12	12	1	Biotin transporter BioY	ATCC29328_12130	ATCC53516_02960	07T609_11570	08T492_11490	09T408_11070	09T494_12210	12T272_09730	12T273_15170	12T306_13170	CCUG54800_12770	T151023_11070	T160124_11190
12	12	1	Isopentenyl-diphosphate delta-isomerase	ATCC29328_12140	ATCC53516_02970	07T609_11580	08T492_11500	09T408_11080	09T494_12220	12T272_09740	12T273_15180	12T306_13180	CCUG54800_12780	T151023_11080	T160124_11200
12	12	1	Putative transcriptional regulator YvhJ	ATCC29328_12150	ATCC53516_02980	07T609_11590	08T492_11510	09T408_11090	09T494_12230	12T272_09750	12T273_15190	12T306_13190	CCUG54800_12790	T151023_11090	T160124_11210
12	12	1	R-phenyllactate dehydratase activator	ATCC29328_12160	ATCC53516_02990	07T609_11600	08T492_11520	09T408_11100	09T494_12240	12T272_09760	12T273_15200	12T306_13200	CCUG54800_12800	T151023_11100	T160124_11220
12	24	0.147	coproporphyrinogen III oxidase	ATCC29328_12170,ATCC2932	ATCC53516_12420,ATCC5351	07T609_15600,07T609_	08T492_16620,08T492_11	09T408_15430,09T408_	09T494_18290,09T494_	12T272_15790,12T272	12T273_18190,12T273	12T306_16510,12T306	CCUG54800_12810,CCUG548	T151023_15070,T151023_	T160124_15510,T160124_11240
12	12	1	tRNA-specific adenosine deaminase	ATCC29328_12180	ATCC53516_03010	07T609_11620	08T492_11540	09T408_11120	09T494_12260	12T272_09790	12T273_15220	12T306_13220	CCUG54800_12820	T151023_11120	T160124_11250
12	12	1	Ribose-phosphate pyrophosphokinase	ATCC29328_12190	ATCC53516_03030	07T609_11630	08T492_11550	09T408_11130	09T494_12270	12T272_09800	12T273_15230	12T306_13230	CCUG54800_12830	T151023_11130	T160124_11260
12	12	1	Threonine-phosphate decarboxylase	ATCC29328_12200	ATCC53516_03040	07T609_11640	08T492_11560	09T408_11140	09T494_12280	12T272_09810	12T273_15240	12T306_13240	CCUG54800_12840	T151023_11140	T160124_11270
12	12	1	Formate dehydrogenase H	ATCC29328_12210	ATCC53516_03050	07T609_11650	08T492_11570	09T408_11150	09T494_12290	12T272_09820	12T273_15250	12T306_13250	CCUG54800_12850	T151023_11150	T160124_11280
12	24	0.167	Putative formate dehydrogenase	ATCC29328_12220,ATCC2932	ATCC53516_03080,ATCC5351	07T609_11680,07T609_	08T492_11600,08T492_11	09T408_11180,09T408_	09T494_12320,09T494_	12T272_09850,12T272	12T273_15280,12T273	12T306_13280,12T306	CCUG54800_12860,CCUG548	T151023_11180,T151023_	T160124_11310,T160124_11290
12	12	1	Rubrerythrin	ATCC29328_12230	ATCC53516_03070	07T609_11670	08T492_11590	09T408_11170	09T494_12310	12T272_09840	12T273_15270	12T306_13270	CCUG54800_12870	T151023_11170	T160124_11300
12	15	0.145	NADP-reducing hydrogenase subunit HndC	ATCC29328_12250	ATCC53516_03090	07T609_11690	08T492_11610	09T408_11190	09T494_13470,09T494_	12T272_10970,12T272	12T273_15290	12T306_13290	CCUG54800_12890	T151023_11190,T151023_	T160124_11320
12	15	0.145	NADP-reducing hydrogenase subunit HndA	ATCC29328_12260	ATCC53516_03100	07T609_11700	08T492_11620	09T408_11200	09T494_13480,09T494_	12T272_10980,12T272	12T273_15300	12T306_13300	CCUG54800_12900	T151023_11200,T151023_	T160124_11330
12	12	1	Selenocysteine-specific elongation factor	ATCC29328_12270	ATCC53516_03110	07T609_11710	08T492_11630	09T408_11210	09T494_12350	12T272_09880	12T273_15310	12T306_13310	CCUG54800_12910	T151023_11210	T160124_11340
12	12	1	S-adenosylmethionine synthase	ATCC29328_12290	ATCC53516_03130	07T609_11730	08T492_11640	09T408_11220	09T494_12370	12T272_09900	12T273_15330	12T306_13330	CCUG54800_12930	T151023_11220	T160124_11360
12	12	1	ABC-2 family transporter protein	ATCC29328_12300	ATCC53516_03140	07T609_11740	08T492_11650	09T408_11230	09T494_12380	12T272_09910	12T273_15340	12T306_13340	CCUG54800_12940	T151023_11230	T160124_11370
12	12	1	putative ABC transporter ATP-binding protein Y	ATCC29328_12310	ATCC53516_03150	07T609_11750	08T492_11660	09T408_11240	09T494_12390	12T272_09920	12T273_15350	12T306_13350	CCUG54800_12950	T151023_11240	T160124_11380
12	12	1	Lactoylglutathione lyase	ATCC29328_12320	ATCC53516_03160	07T609_11760	08T492_11670	09T408_11250	09T494_12400	12T272_09930	12T273_15360	12T306_13360	CCUG54800_12960	T151023_11250	T160124_11390
12	12	1	hypothetical protein	ATCC29328_12340	ATCC53516_03180	07T609_11780	08T492_11690	09T408_11270	09T494_12420	12T272_09950	12T273_15380	12T306_13380	CCUG54800_12980	T151023_11270	T160124_11410
12	12	1	V-type sodium ATPase subunit D	ATCC29328_12350	ATCC53516_03190	07T609_11790	08T492_11700	09T408_11280	09T494_12430	12T272_09960	12T273_15390	12T306_13390	CCUG54800_12990	T151023_11280	T160124_11420
12	12	1	V-type ATP synthase subunit E	ATCC29328_12380	ATCC53516_03220	07T609_11820	08T492_11730	09T408_11310	09T494_12460	12T272_09990	12T273_15420	12T306_13420	CCUG54800_13020	T151023_11310	T160124_11450
12	12	1	V-type ATP synthase subunit F	ATCC29328_12390	ATCC53516_03230	07T609_11830	08T492_11740	09T408_11320	09T494_12470	12T272_10000	12T273_15430	12T306_13430	CCUG54800_13030	T151023_11320	T160124_11460
12	12	1	V-type ATP synthase subunit K	ATCC29328_12400	ATCC53516_03240	07T609_11840	08T492_11750	09T408_11330	09T494_12480	12T272_10010	12T273_15440	12T306_13440	CCUG54800_13040	T151023_11330	T160124_11470
12	12	1	V-type ATP synthase subunit I	ATCC29328_12410	ATCC53516_03250	07T609_11850	08T492_11760	09T408_11340	09T494_12490	12T272_10020	12T273_15450	12T306_13450	CCUG54800_13050	T151023_11340	T160124_11480
12	12	1	V-type ATP synthase subunit C	ATCC29328_12420	ATCC53516_03260	07T609_11860	08T492_11770	09T408_11350	09T494_12500	12T272_10030	12T273_15460	12T306_13460	CCUG54800_13060	T151023_11350	T160124_11490
12	12	1	hypothetical protein	ATCC29328_12430	ATCC53516_03270	07T609_11870	08T492_11780	09T408_11360	09T494_12510	12T272_10040	12T273_15470	12T306_13470	CCUG54800_13070	T151023_11360	T160124_11500
7	7	1	hypothetical protein	ATCC29328_12440	ATCC53516_03290	07T609_11880	08T492_11950	*	*	*	*	12T306_13480	CCUG54800_13090	*	T160124_11670
8	8	1	Chaperone protein DnaJ	ATCC29328_12450	*	07T609_11890	08T492_11960	09T408_11370	*	*	12T273_15480	12T306_13490	*	T151023_11370	T160124_11680
12	12	1	Denitrification regulatory protein NirQ	ATCC29328_12460	ATCC53516_03310	07T609_11900	08T492_11970	09T408_11380	09T494_12530	12T272_10060	12T273_15490	12T306_13500	CCUG54800_13110	T151023_11380	T160124_11690
12	12	1	hypothetical protein	ATCC29328_12470	ATCC53516_03320	07T609_11910	08T492_11980	09T408_11390	09T494_12540	12T272_10070	12T273_15500	12T306_13510	CCUG54800_13120	T151023_11390	T160124_11700
12	12	1	anaerobic ribonucleotide reductase-activating	ATCC29328_12480	ATCC53516_03330	07T609_11920	08T492_11990	09T408_11400	09T494_12550	12T272_10080	12T273_15510	12T306_13520	CCUG54800_13130	T151023_11400	T160124_11710
12	12	1	Anaerobic ribonucleoside-triphosphate reduct	ATCC29328_12490	ATCC53516_03340	07T609_11930	08T492_12000	09T408_11410	09T494_12560	12T272_10090	12T273_15520	12T306_13530	CCUG54800_13140	T151023_11410	T160124_11720
12	12	1	Bifunctional xylanase/deacetylase precursor	ATCC29328_12500	ATCC53516_03350	07T609_11940	08T492_12010	09T408_11420	09T494_12570	12T272_10100	12T273_15530	12T306_13540	CCUG54800_13150	T151023_11420	T160124_11730
12	12	1	putative CtpA-like serine protease	ATCC29328_12510	ATCC53516_03360	07T609_11950	08T492_12020	09T408_11430	09T494_12580	12T272_10110	12T273_15540	12T306_13550	CCUG54800_13160	T151023_11430	T160124_11740
7	7	1	Peptidase family S41	ATCC29328_12520	ATCC53516_03370	07T609_11960	*	*	*	12T272_10130	*	12T306_13560	CCUG54800_13170	T151023_11440	*
11	13	0.154	Peptidase family S41	ATCC29328_12530	ATCC53516_03480,ATCC5351	*	08T492_12030	09T408_11450	09T494_12600	12T272_10140	12T273_15550,12T273	12T306_13570	CCUG54800_13180	T151023_11470	T160124_11750
12	12	1	Murein DD-endopeptidase MepM	ATCC29328_12540	ATCC53516_03390	07T609_11990	08T492_12040	09T408_11460	09T494_12610	12T272_10150	12T273_15560	12T306_13580	CCUG54800_13190	T151023_11480	T160124_11760
12	12	1	Cell division protein FtsX	ATCC29328_12550	ATCC53516_03400	07T609_12000	08T492_12050	09T408_11470	09T494_12620	12T272_10160	12T273_15570	12T306_13590	CCUG54800_13200	T151023_11490	T160124_11770
12	12	1	Cell division ATP-binding protein FtsE	ATCC29328_12560	ATCC53516_03410	07T609_12010	08T492_12060	09T408_11480	09T494_12630	12T272_10170	12T273_15580	12T306_13600	CCUG54800_13210	T151023_11500	T160124_11780
12	12	1	putative 5-formyltetrahydrofolate cyclo-ligase	ATCC29328_12570	ATCC53516_03420	07T609_12020	08T492_12070	09T408_11490	09T494_12640	12T272_10180	12T273_15590	12T306_13610	CCUG54800_13220	T151023_11510	T160124_11790
11	11	1	hypothetical protein	ATCC29328_12590	ATCC53516_16920	07T609_12040	08T492_12090	09T408_09170	09T494_10120	12T272_07880	*	12T306_13630	CCUG54800_13240	T151023_11530	T160124_11810
10	13	0.141	Peptidase family S41	ATCC29328_12600	ATCC53516_03490,ATCC5351	07T609_11980,07T609_	08T492_12100	09T408_11500	09T494_12650	*	12T273_15680	12T306_13640	CCUG54800_13260	T151023_11460,T151023_	*
11	13	0.252	Peptidase family S41	ATCC29328_12610	ATCC53516_03470	07T609_12060,07T609_	08T492_12110	09T408_11440	09T494_12590	12T272_10120	*	12T306_13680	CCUG54800_13280	T151023_11550,T151023_	T160124_11820
6	7	0.175	hypothetical protein	ATCC29328_12620	*	07T609_12070	*	*	*	*	12T273_15630	*	CCUG54800_13290,CCUG548	T151023_11560	T160124_11850
5	6	0.24	hypothetical protein	ATCC29328_12630	*	07T609_12080	*	*	09T494_12700	*	12T273_15650,12T273	*	*	*	T160124_11830
12	12	1	hypothetical protein	ATCC29328_12640	ATCC53516_03500	07T609_12090	08T492_12140	09T408_11530	09T494_12720	12T272_10210	12T273_15690	12T306_13700	CCUG54800_13370	T151023_11580	T160124_11870
12	12	1	mRNA interferase EndoA	ATCC29328_12650	ATCC53516_03510	07T609_12100	08T492_12150	09T408_11540	09T494_12730	12T272_10220	12T273_15700	12T306_13710	CCUG54800_13380	T151023_11590	T160124_11880
12	12	1	Alanine racemase 1	ATCC29328_12660	ATCC53516_03520	07T609_12110	08T492_12160	09T408_11550	09T494_12740	12T272_10230	12T273_15710	12T306_13720	CCUG54800_13390	T151023_11600	T160124_11890
12	12	1	Bifunctional NAD(P)H-hydrate repair enzyme N	ATCC29328_12670	ATCC53516_03530	07T609_12120	08T492_12170	09T408_11560	09T494_12750	12T272_10240	12T273_15720	12T306_13730	CCUG54800_13400	T151023_11610	T160124_11900
12	12	1	Holo-[acyl-carrier-protein] synthase	ATCC29328_12680	ATCC53516_03540	07T609_12130	08T492_12180	09T408_11570	09T494_12760	12T272_10250	12T273_15730	12T306_13740	CCUG54800_13410	T151023_11620	T160124_11910
12	12	1	Region found in RelA / SpoT proteins	ATCC29328_12690	ATCC53516_03550	07T609_12140	08T492_12190	09T408_11580	09T494_12770	12T272_10260	12T273_15740	12T306_13750	CCUG54800_13420	T151023_11630	T160124_11920
12	12	1	hypothetical protein	ATCC29328_12700	ATCC53516_03560	07T609_12150	08T492_12200	09T408_11590	09T494_12780	12T272_10270	12T273_15750	12T306_13760	CCUG54800_13430	T151023_11640	T160124_11930
12	24	0.167	R-phenyllactate dehydratase beta subunit	ATCC29328_12710,ATCC2932	ATCC53516_12150,ATCC5351	07T609_15750,07T609_	08T492_16770,08T492_12	09T408_15690,09T408_	09T494_00500,09T494_	12T272_16040,12T272	12T273_18490,12T273	12T306_16650,12T306	CCUG54800_13440,CCUG548	T151023_15210,T151023_	T160124_15760,T160124_11940
12	12	1	2-hydroxyglutaryl-CoA dehydratase, D-compo	ATCC29328_12720	ATCC53516_03580	07T609_12170	08T492_12220	09T408_11610	09T494_12800	12T272_10290	12T273_15770	12T306_13780	CCUG54800_13450	T151023_11660	T160124_11950
12	12	1	Activator of (R)-2-hydroxyglutaryl-CoA dehydra	ATCC29328_12730	ATCC53516_03590	07T609_12180	08T492_12230	09T408_11620	09T494_12810	12T272_10300	12T273_15780	12T306_13790	CCUG54800_13460	T151023_11670	T160124_11960
12	12	1	hypothetical protein	ATCC29328_12740	ATCC53516_03600	07T609_12190	08T492_12240	09T408_11630	09T494_12820	12T272_10310	12T273_15790	12T306_13800	CCUG54800_13470	T151023_11680	T160124_11970
12	12	1	putative adenine permease PurP	ATCC29328_12750	ATCC53516_03610	07T609_12200	08T492_12250	09T408_11640	09T494_12830	12T272_10320	12T273_15800	12T306_13810	CCUG54800_13480	T151023_11690	T160124_11980
12	12	1	Lon protease 1	ATCC29328_12780	ATCC53516_03640	07T609_12230	08T492_12280	09T408_11670	09T494_12860	12T272_10350	12T273_15830	12T306_13840	CCUG54800_13510	T151023_11720	T160124_12010
12	12	1	ATP-dependent Clp protease ATP-binding subu	ATCC29328_12790	ATCC53516_03650	07T609_12240	08T492_12290	09T408_11680	09T494_12870	12T272_10360	12T273_15840	12T306_13850	CCUG54800_13520	T151023_11730	T160124_12020
12	12	1	ATP-dependent Clp protease proteolytic subur	ATCC29328_12800	ATCC53516_03660	07T609_12250	08T492_12300	09T408_11690	09T494_12880	12T272_10370	12T273_15850	12T306_13860	CCUG54800_13530	T151023_11740	T160124_12030
12	12	1	Trigger factor	ATCC29328_12810	ATCC53516_03670	07T609_12260	08T492_12310	09T408_11700	09T494_12890	12T272_10380	12T273_15860	12T306_13870	CCUG54800_13540	T151023_11750	T160124_12040
12	12	1	N-acetylmuramoyl-L-alanine amidase LytC pre	ATCC29328_12820	ATCC53516_03680	07T609_12270	08T492_12320	09T408_11710	09T494_12900	12T272_10390	12T273_15870	12T306_13880	CCUG54800_13550	T151023_11760	T160124_12050
12	12	1	HTH-type transcriptional regulator YesS	ATCC29328_12830	ATCC53516_03690	07T609_12280	08T492_12330	09T408_11720	09T494_12910	12T272_10400	12T273_15880	12T306_13890	CCUG54800_13560	T151023_11770	T160124_12060
12	12	1	ferrous iron transport protein A	ATCC29328_12850	ATCC53516_03710	07T609_12300	08T492_12350	09T408_11740	09T494_12930	12T272_10420	12T273_15900	12T306_13910	CCUG54800_13590	T151023_11790	T160124_12080
12	12	1	FeoA domain protein	ATCC29328_12860	ATCC53516_03720	07T609_12310	08T492_12360	09T408_11750	09T494_12940	12T272_10430	12T273_15910	12T306_13920	CCUG54800_13600	T151023_11800	T160124_12090
12	12	1	Glutamine--fructose-6-phosphate aminotransf	ATCC29328_12870	ATCC53516_03730	07T609_12320	08T492_12380	09T408_11770	09T494_12950	12T272_10440	12T273_15920	12T306_13930	CCUG54800_13610	T151023_11810	T160124_12100
12	12	1	tRNA N6-adenosine threonylcarbamoyltransfe	ATCC29328_12880	ATCC53516_03740	07T609_12330	08T492_12390	09T408_11780	09T494_12960	12T272_10450	12T273_15930	12T306_13940	CCUG54800_13620	T151023_11820	T160124_12110
12	12	1	tRNA threonylcarbamoyladenosine biosynthes	ATCC29328_12890	ATCC53516_03750	07T609_12340	08T492_12400	09T408_11790	09T494_12970	12T272_10460	12T273_15940	12T306_13950	CCUG54800_13630	T151023_11830	T160124_12120
12	12	1	tRNA threonylcarbamoyladenosine biosynthes	ATCC29328_12900	ATCC53516_03760	07T609_12350	08T492_12410	09T408_11800	09T494_12980	12T272_10470	12T273_15950	12T306_13960	CCUG54800_13640	T151023_11840	T160124_12130
12	12	1	Riboflavin transporter RibU	ATCC29328_12910	ATCC53516_03770	07T609_12360	08T492_12420	09T408_11810	09T494_12990	12T272_10480	12T273_15960	12T306_13970	CCUG54800_13650	T151023_11850	T160124_12140
12	12	1	putative M18 family aminopeptidase 2	ATCC29328_12920	ATCC53516_03780	07T609_12370	08T492_12430	09T408_11820	09T494_13000	12T272_10490	12T273_15970	12T306_13980	CCUG54800_13660	T151023_11860	T160124_12150
12	12	1	5'-methylthioadenosine/S-adenosylhomocyste	ATCC29328_12940	ATCC53516_03800	07T609_12390	08T492_12450	09T408_11840	09T494_13020	12T272_10510	12T273_15990	12T306_13990	CCUG54800_13680	T151023_11880	T160124_12170
12	12	1	Phosphoglucosamine mutase	ATCC29328_12950	ATCC53516_03810	07T609_12400	08T492_12460	09T408_11850	09T494_13030	12T272_10520	12T273_16000	12T306_14000	CCUG54800_13690	T151023_11890	T160124_12180
10	10	1	hypothetical protein	ATCC29328_12960	ATCC53516_03820	07T609_12410	08T492_12470	09T408_11860	09T494_13040	12T272_10530	12T273_16010	*	CCUG54800_13700	*	T160124_12190
12	12	1	YbbR-like protein	ATCC29328_12970	ATCC53516_03830	07T609_12420	08T492_12480	09T408_11870	09T494_13050	12T272_10540	12T273_16020	12T306_14010	CCUG54800_13710	T151023_11900	T160124_12200
12	12	1	DNA integrity scanning protein DisA	ATCC29328_12980	ATCC53516_03840	07T609_12430	08T492_12490	09T408_11880	09T494_13060	12T272_10550	12T273_16030	12T306_14020	CCUG54800_13720	T151023_11910	T160124_12210
12	12	1	HPr-like protein Crh	ATCC29328_12990	ATCC53516_03850	07T609_12440	08T492_12500	09T408_11890	09T494_13070	12T272_10560	12T273_16040	12T306_14030	CCUG54800_13730	T151023_11920	T160124_12220
12	24	0.167	Rubrerythrin-2	ATCC29328_13000,ATCC2932	ATCC53516_11590,ATCC5351	07T609_16440,07T609_	08T492_17240,08T492_12	09T408_16270,09T408_	09T494_01000,09T494_	12T272_16550,12T272	12T273_00260,12T273	12T306_17180,12T306	CCUG54800_13740,CCUG548	T151023_15870,T151023_	T160124_16270,T160124_12230
12	12	1	2-C-methyl-D-erythritol 2,4-cyclodiphosphate s	ATCC29328_13020	ATCC53516_03880	07T609_12470	08T492_12530	09T408_11920	09T494_13100	12T272_10590	12T273_16070	12T306_14060	CCUG54800_13760	T151023_11950	T160124_12250
12	12	1	2-C-methyl-D-erythritol 4-phosphate cytidylylt	ATCC29328_13030	ATCC53516_03890	07T609_12480	08T492_12540	09T408_11930	09T494_13110	12T272_10600	12T273_16080	12T306_14070	CCUG54800_13770	T151023_11960	T160124_12260
12	12	1	putative PIN and TRAM-domain containing pro	ATCC29328_13040	ATCC53516_03900	07T609_12490	08T492_12550	09T408_11940	09T494_13120	12T272_10610	12T273_16090	12T306_14080	CCUG54800_13780	T151023_11970	T160124_12270
12	12	1	RNA polymerase-binding transcription factor C	ATCC29328_13050	ATCC53516_03910	07T609_12500	08T492_12560	09T408_11950	09T494_13130	12T272_10620	12T273_16100	12T306_14090	CCUG54800_13790	T151023_11980	T160124_12280
12	12	1	Cell wall-binding protein YocH precursor	ATCC29328_13060	ATCC53516_03920	07T609_12510	08T492_12570	09T408_11960	09T494_13140	12T272_10630	12T273_16110	12T306_14100	CCUG54800_13800	T151023_11990	T160124_12290
12	12	1	hypothetical protein	ATCC29328_13070	ATCC53516_03930	07T609_12520	08T492_12580	09T408_11970	09T494_13150	12T272_10640	12T273_16120	12T306_14110	CCUG54800_13810	T151023_12000	T160124_12300
12	12	1	Oxygen-independent coproporphyrinogen-III o	ATCC29328_13080	ATCC53516_03940	07T609_12530	08T492_12590	09T408_11980	09T494_13160	12T272_10650	12T273_16130	12T306_14120	CCUG54800_13820	T151023_12010	T160124_12310
12	12	1	Ribonucleoside-diphosphate reductase subuni	ATCC29328_13090	ATCC53516_03950	07T609_12540	08T492_12600	09T408_11990	09T494_13170	12T272_10660	12T273_16140	12T306_14130	CCUG54800_13830	T151023_12020	T160124_12320
12	14	0.143	3-dehydroquinate dehydratase	ATCC29328_13100	ATCC53516_03960	07T609_12550	08T492_13230,08T492_12	09T408_12000	09T494_13180	12T272_10670	12T273_16150,12T273	12T306_14140	CCUG54800_13840	T151023_12030	T160124_12330
12	13	0.141	Shikimate dehydrogenase	ATCC29328_13110	ATCC53516_03970	07T609_12560	08T492_12620,08T492_13	09T408_12010	09T494_13190	12T272_10680	12T273_16160	12T306_14150	CCUG54800_13850	T151023_12040	T160124_12340
12	12	1	hypothetical protein	ATCC29328_13120	ATCC53516_03980	07T609_12570	08T492_12630	09T408_12020	09T494_13200	12T272_10690	12T273_16170	12T306_14160	CCUG54800_13860	T151023_12050	T160124_12350
12	13	0.141	Chorismate synthase	ATCC29328_13130	ATCC53516_03990	07T609_12580	08T492_12640,08T492_13	09T408_12030	09T494_13210	12T272_10700	12T273_16180	12T306_14170	CCUG54800_13870	T151023_12060	T160124_12360
12	14	0.143	3-phosphoshikimate 1-carboxyvinyltransferase	ATCC29328_13140	ATCC53516_04000	07T609_12590	08T492_13260,08T492_12	09T408_12040	09T494_13220	12T272_10710	12T273_16190,12T273	12T306_14180	CCUG54800_13880	T151023_12070	T160124_12370
12	14	0.143	3-dehydroquinate synthase	ATCC29328_13150	ATCC53516_04010	07T609_12600	08T492_13270,08T492_12	09T408_12050	09T494_13230	12T272_10720	12T273_16200,12T273	12T306_14190	CCUG54800_13890	T151023_12080	T160124_12380
12	14	0.143	Phospho-2-dehydro-3-deoxyheptonate aldolas	ATCC29328_13160	ATCC53516_04020	07T609_12610	08T492_13220,08T492_12	09T408_12060	09T494_13240	12T272_10730	12T273_16210,12T273	12T306_14200	CCUG54800_13900	T151023_12090	T160124_12390
12	12	1	hypothetical protein	ATCC29328_13170	ATCC53516_04030	07T609_12620	08T492_12680	09T408_12070	09T494_13250	12T272_10740	12T273_16220	12T306_14210	CCUG54800_13910	T151023_12100	T160124_12400
12	12	1	Aminoglycoside 6-adenylyltransferase	ATCC29328_13180	ATCC53516_04040	07T609_12630	08T492_12690	09T408_12080	09T494_13260	12T272_10750	12T273_16230	12T306_14220	CCUG54800_13920	T151023_12110	T160124_12410
12	12	1	5'-nucleotidase	ATCC29328_13190	ATCC53516_04050	07T609_12640	08T492_12700	09T408_12090	09T494_13270	12T272_10760	12T273_16240	12T306_14230	CCUG54800_13930	T151023_12120	T160124_12420
12	15	0.113	Oligopeptide transport ATP-binding protein Op	ATCC29328_13200,ATCC2932	ATCC53516_04060,ATCC5351	07T609_12650	08T492_12710	09T408_12100	09T494_13280	12T272_10770	12T273_16250,12T273	12T306_14240	CCUG54800_13940	T151023_12130	T160124_12430
3	5	0.401	Oligopeptide transport ATP-binding protein Op	ATCC29328_13210,ATCC2932	ATCC53516_10900	*	*	*	*	*	12T273_18700,12T273	*	*	*	*
12	16	0.147	Dipeptide transport system permease protein	ATCC29328_13220,ATCC2932	ATCC53516_10910,ATCC5351	07T609_12670	08T492_12730	09T408_12120	09T494_13300	12T272_10790	12T273_18690,12T273	12T306_14260	CCUG54800_13960	T151023_12150,T151023_	T160124_12450
12	16	0.147	Oligopeptide transport system permease prote	ATCC29328_13230,ATCC2932	ATCC53516_10920,ATCC5351	07T609_12680	08T492_12740	09T408_12130	09T494_13310	12T272_10800	12T273_18680,12T273	12T306_14270	CCUG54800_13970	T151023_12160,T151023_	T160124_12460
12	12	0.833	Dipeptide-binding protein DppE precursor	ATCC29328_13240	ATCC53516_04100	07T609_12690	08T492_12750	09T408_12140	09T494_13320	12T272_10810	12T273_16290	12T306_14280	CCUG54800_13980	T151023_12170	T160124_12470
12	12	1	Low specificity L-threonine aldolase	ATCC29328_13250	ATCC53516_04110	07T609_12700	08T492_12770	09T408_12150	09T494_13330	12T272_10820	12T273_16300	12T306_14290	CCUG54800_13990	T151023_12180	T160124_12480
12	12	1	hypothetical protein	ATCC29328_13260	ATCC53516_04120	07T609_12710	08T492_12780	09T408_12160	09T494_13340	12T272_10830	12T273_16310	12T306_14300	CCUG54800_14000	T151023_12190	T160124_12490
12	12	1	Methylated-DNA--protein-cysteine methyltran	ATCC29328_13270	ATCC53516_04130	07T609_12720	08T492_12790	09T408_12170	09T494_13350	12T272_10850	12T273_16320	12T306_14310	CCUG54800_14010	T151023_12200	T160124_12500
12	12	1	hypothetical protein	ATCC29328_13280	ATCC53516_04140	07T609_12730	08T492_12800	09T408_12180	09T494_13360	12T272_10860	12T273_16330	12T306_14320	CCUG54800_14020	T151023_12210	T160124_12510
12	12	1	hypothetical protein	ATCC29328_13290	ATCC53516_04150	07T609_12740	08T492_12810	09T408_12190	09T494_13370	12T272_10870	12T273_16340	12T306_14330	CCUG54800_14030	T151023_12220	T160124_12520
12	12	1	Phosphatidylglycerophosphatase A	ATCC29328_13300	ATCC53516_04160	07T609_12750	08T492_12820	09T408_12200	09T494_13380	12T272_10880	12T273_16350	12T306_14340	CCUG54800_14040	T151023_12230	T160124_12530
12	12	1	N-acetylmuramoyl-L-alanine amidase LytC pre	ATCC29328_13320	ATCC53516_04210	07T609_12770	08T492_12840	09T408_12220	09T494_13430	12T272_10930	12T273_16400	12T306_14390	CCUG54800_14090	T151023_12280	T160124_12580
12	24	0.167	YibE/F-like protein	ATCC29328_13330,ATCC2932	ATCC53516_04230,ATCC5351	07T609_12790,07T609_	08T492_12860,08T492_12	09T408_12240,09T408_	09T494_13450,09T494_	12T272_10950,12T272	12T273_16420,12T273	12T306_14410,12T306	CCUG54800_14100,CCUG548	T151023_12300,T151023_	T160124_12600,T160124_12590
5	5	0.4	NADP-reducing hydrogenase subunit HndC	ATCC29328_13350	ATCC53516_04240	07T609_12810	*	09T408_12260	*	*	12T273_16430	*	*	*	*
12	12	1	Lactococcin-G-processing and transport ATP-bi	ATCC29328_13360	ATCC53516_04250	07T609_12820	08T492_12880	09T408_12270	09T494_13520	12T272_11020	12T273_16440	12T306_14420	CCUG54800_14120	T151023_12370	T160124_12620
11	11	1	putative ABC transporter ATP-binding protein	ATCC29328_13390	ATCC53516_04450	07T609_12850	08T492_12910	*	09T494_13550	12T272_11050	12T273_16470	12T306_14450	CCUG54800_14150	T151023_12400	T160124_12650
11	11	1	Bacitracin export ATP-binding protein BceA	ATCC29328_13400	ATCC53516_04460	07T609_12860	08T492_12920	*	09T494_13560	12T272_11060	12T273_16480	12T306_14460	CCUG54800_14160	T151023_12410	T160124_12660
12	12	1	EamA-like transporter family protein	ATCC29328_13410	ATCC53516_04470	07T609_12870	08T492_12940	09T408_12300	09T494_13570	12T272_11070	12T273_16490	12T306_14470	CCUG54800_14170	T151023_12420	T160124_12670
8	8	1	putative multidrug resistance ABC transporter	ATCC29328_13420	ATCC53516_04480	*	*	*	09T494_13580	12T272_11080	12T273_16500	12T306_14480	CCUG54800_14180	T151023_12430	*
5	5	1	hypothetical protein	ATCC29328_13430	*	*	*	*	*	*	12T273_16510	12T306_14490	CCUG54800_14190	*	T160124_12720
12	12	1	hypothetical protein	ATCC29328_13440	ATCC53516_04490	07T609_12880	08T492_12950	09T408_12310	09T494_13600	12T272_11110	12T273_16520	12T306_14500	CCUG54800_14200	T151023_12450	T160124_12730
11	11	1	hypothetical protein	ATCC29328_13450	ATCC53516_04500	07T609_12890	08T492_12960	09T408_12320	09T494_13610	12T272_11120	12T273_16530	12T306_14510	CCUG54800_14210	*	T160124_12740
11	11	1	Methionine import ATP-binding protein MetN	ATCC29328_13460	ATCC53516_04510	07T609_12900	08T492_12970	09T408_12330	09T494_13620	12T272_11130	12T273_16540	12T306_14520	CCUG54800_14220	*	T160124_12750
12	12	1	hypothetical protein	ATCC29328_13470	ATCC53516_04520	07T609_12910	08T492_12980	09T408_12340	09T494_13630	12T272_11140	12T273_16550	12T306_14530	CCUG54800_14230	T151023_12460	T160124_12760
11	11	0.262	Putative multidrug export ATP-binding/permea	ATCC29328_13480	ATCC53516_04530	07T609_12920	08T492_14000	09T408_12350	09T494_14170	12T272_11150	12T273_17010	12T306_14540	CCUG54800_14530	*	T160124_12770
12	12	1	hypothetical protein	ATCC29328_13490	ATCC53516_04540	07T609_12930	08T492_14010	09T408_12360	09T494_14180	12T272_11170	12T273_17020	12T306_14550	CCUG54800_14540	T151023_12480	T160124_12780
12	13	0.141	Anaerobic sulfatase-maturating enzyme	ATCC29328_13500	ATCC53516_04550	07T609_12940	08T492_14020	09T408_12370	09T494_14190	12T272_11180	12T273_17030	12T306_14560	CCUG54800_14550,CCUG548	T151023_12490	T160124_12790
12	12	1	Arginine decarboxylase	ATCC29328_13510	ATCC53516_04560	07T609_12950	08T492_14030	09T408_12380	09T494_14200	12T272_11190	12T273_17040	12T306_14570	CCUG54800_14560	T151023_12500	T160124_12800
12	12	1	HTH-type transcriptional repressor YtrA	ATCC29328_13520	ATCC53516_04570	07T609_12960	08T492_14040	09T408_12390	09T494_14210	12T272_11200	12T273_17050	12T306_14580	CCUG54800_14570	T151023_12510	T160124_12810
12	12	1	ABC transporter ATP-binding protein YtrB	ATCC29328_13530	ATCC53516_04580	07T609_12970	08T492_14050	09T408_12400	09T494_14220	12T272_11210	12T273_17060	12T306_14590	CCUG54800_14580	T151023_12520	T160124_12820
12	12	1	ABC-2 family transporter protein	ATCC29328_13540	ATCC53516_04600	07T609_12980	08T492_14060	09T408_12410	09T494_14230	12T272_11220	12T273_17070	12T306_14600	CCUG54800_14590	T151023_12530	T160124_02860
12	24	0.129	Chromate transport protein	ATCC29328_13550,ATCC2932	ATCC53516_04620,ATCC5351	07T609_13000,07T609_	08T492_14080,08T492_14	09T408_12430,09T408_	09T494_14250,09T494_	12T272_11240,12T272	12T273_06280,12T273	12T306_14620,12T306	CCUG54800_14600,CCUG548	T151023_12550,T151023_	T160124_02840,T160124_02850
5	5	1	Branched-chain amino acid transport system 2	ATCC29328_13570	*	07T609_13010	*	09T408_12440	09T494_14260	12T272_11250	*	*	*	*	*
12	12	1	Fructose-bisphosphate aldolase	ATCC29328_13580	ATCC53516_04630	07T609_13020	08T492_14090	09T408_12450	09T494_14270	12T272_11260	12T273_06270	12T306_14630	CCUG54800_14620	T151023_12560	T160124_02830
12	12	1	PTS system fructose-specific EIIABC componen	ATCC29328_13590	ATCC53516_04640	07T609_13030	08T492_14100	09T408_12460	09T494_14280	12T272_11270	12T273_06260	12T306_14640	CCUG54800_14630	T151023_12570	T160124_02820
12	12	1	Septum formation protein Maf	ATCC29328_13610	ATCC53516_04660	07T609_13050	08T492_14120	09T408_12480	09T494_14300	12T272_11290	12T273_06240	12T306_14660	CCUG54800_14650	T151023_12590	T160124_02800
12	12	1	hypothetical protein	ATCC29328_13620	ATCC53516_04670	07T609_13060	08T492_14130	09T408_12490	09T494_14310	12T272_11300	12T273_06230	12T306_14670	CCUG54800_14660	T151023_12600	T160124_02790
12	12	1	hypothetical protein	ATCC29328_13630	ATCC53516_04680	07T609_13070	08T492_14140	09T408_12500	09T494_14330	12T272_11310	12T273_06220	12T306_14680	CCUG54800_14670	T151023_12610	T160124_02780
12	12	1	hypothetical protein	ATCC29328_13640	ATCC53516_04690	07T609_13080	08T492_14170	09T408_12510	09T494_14340	12T272_11320	12T273_06210	12T306_14690	CCUG54800_14680	T151023_12620	T160124_02770
12	12	1	Lipoyl synthase	ATCC29328_13650	ATCC53516_04700	07T609_13090	08T492_14180	09T408_12520	09T494_14350	12T272_11330	12T273_06200	12T306_14700	CCUG54800_14690	T151023_12630	T160124_02760
12	12	1	Lipoate-protein ligase LplJ	ATCC29328_13660	ATCC53516_04710	07T609_13100	08T492_14190	09T408_12530	09T494_14360	12T272_11340	12T273_06190	12T306_14710	CCUG54800_14700	T151023_12640	T160124_02750
12	12	1	hypothetical protein	ATCC29328_13670	ATCC53516_04720	07T609_13110	08T492_14200	09T408_12540	09T494_14370	12T272_11350	12T273_06180	12T306_14720	CCUG54800_14710	T151023_12650	T160124_02740
12	12	1	Thioesterase superfamily protein	ATCC29328_13680	ATCC53516_04730	07T609_13120	08T492_14210	09T408_12550	09T494_14380	12T272_11360	12T273_06170	12T306_14730	CCUG54800_14720	T151023_12660	T160124_02730
12	12	1	bifunctional ATP-dependent DNA helicase/DN	ATCC29328_13690	ATCC53516_04740	07T609_13130	08T492_14220	09T408_12560	09T494_14390	12T272_11370	12T273_06160	12T306_14740	CCUG54800_14730	T151023_12670	T160124_02720
12	12	1	hypothetical protein	ATCC29328_13700	ATCC53516_04750	07T609_13140	08T492_14230	09T408_12570	09T494_14400	12T272_11380	12T273_06150	12T306_14750	CCUG54800_14740	T151023_12680	T160124_02710
12	12	1	23S rRNA (uracil-C(5))-methyltransferase RlmC	ATCC29328_13710	ATCC53516_04760	07T609_13150	08T492_14240	09T408_12580	09T494_14410	12T272_11390	12T273_06140	12T306_14760	CCUG54800_14750	T151023_12690	T160124_02700
9	9	1	Microcin C7 self-immunity protein MccF	ATCC29328_13720	*	07T609_13160	08T492_14250	09T408_13710	09T494_14420	12T272_11400	12T273_06130	12T306_02700	*	*	T160124_02690
11	11	1	hypothetical protein	ATCC29328_13730	ATCC53516_04770	07T609_13170	08T492_14260	*	09T494_14430	12T272_11410	12T273_06120	12T306_14800	CCUG54800_14760	T151023_12700	T160124_02680
8	8	1	Rubrerythrin	ATCC29328_13740	ATCC53516_04800	07T609_13180	08T492_14270	09T408_12590	*	*	12T273_06110	12T306_14810	*	*	T160124_02670
8	8	1	hypothetical protein	ATCC29328_13750	ATCC53516_04810	07T609_13190	08T492_14280	09T408_12600	*	*	12T273_06090	12T306_14830	*	*	T160124_02650
12	12	1	Acetyltransferase (GNAT) family protein	ATCC29328_13760	ATCC53516_04840	07T609_13200	08T492_14300	09T408_12610	09T494_14440	12T272_11420	12T273_06060	12T306_14840	CCUG54800_14770	T151023_12710	T160124_02620
6	6	1	Archaeal ATPase	ATCC29328_13770	ATCC53516_04850	07T609_13210	*	*	*	*	12T273_06050	12T306_14850	*	*	T160124_02610
4	4	1	FtsX-like permease family protein	ATCC29328_13780	*	07T609_13220	08T492_14340	09T408_12630	*	*	*	*	*	*	*
12	16	0.147	Bacitracin export ATP-binding protein BceA	ATCC29328_13790,ATCC2932	ATCC53516_00200	07T609_13620,07T609_	08T492_14350,08T492_14	09T408_13080,09T408_	09T494_16630	12T272_14040	12T273_17230	12T306_15250	CCUG54800_15110	T151023_14200	T160124_12990
12	13	1	hypothetical protein	ATCC29328_13800	ATCC53516_04880	07T609_13240	08T492_14360,08T492_14	09T408_12650	09T494_14470	12T272_11450	12T273_06020	12T306_14880	CCUG54800_14780	T151023_12720	T160124_02580
12	12	1	hypothetical protein	ATCC29328_13810	ATCC53516_04890	07T609_13250	08T492_14370	09T408_12660	09T494_14480	12T272_11460	12T273_06010	12T306_14890	CCUG54800_14790	T151023_12730	T160124_02570
12	13	0.141	ABC-type transporter ATP-binding protein EcsA	ATCC29328_13820	ATCC53516_04900	07T609_13260	08T492_14380	09T408_12670	09T494_14490	12T272_11470	12T273_06000	12T306_14900	CCUG54800_14800,CCUG548	T151023_12740	T160124_02560
12	12	1	hypothetical protein	ATCC29328_13830	ATCC53516_04910	07T609_13270	08T492_14390	09T408_12680	09T494_14500	12T272_11480	12T273_05990	12T306_14910	CCUG54800_14810	T151023_12750	T160124_02550
11	11	1	hypothetical protein	ATCC29328_13840	ATCC53516_04950	07T609_13280	08T492_14400	09T408_12690	09T494_14520	12T272_11500	*	12T306_14930	CCUG54800_14830	T151023_12760	T160124_02530
2	2	1	hypothetical protein	ATCC29328_13850	*	07T609_13290	*	*	*	*	*	*	*	*	*
12	12	1	5-methyltetrahydropteroyltriglutamate--homo	ATCC29328_13870	ATCC53516_05000	07T609_13320	08T492_14420	09T408_12750	09T494_16380	12T272_13740	12T273_06450	12T306_04630	CCUG54800_03880	T151023_03270	T160124_02990
12	15	0.145	D-methionine-binding lipoprotein MetQ precu	ATCC29328_13880	ATCC53516_08600,ATCC5351	07T609_13330	08T492_14430	09T408_12770	09T494_16400	12T272_13750	12T273_06440	12T306_04620	CCUG54800_03870,CCUG548	T151023_03260	T160124_01850,T160124_02980
12	15	0.145	D-methionine transport system permease prot	ATCC29328_13890	ATCC53516_08610,ATCC5351	07T609_13340	08T492_14440	09T408_12780	09T494_16410	12T272_13760	12T273_06430	12T306_04610	CCUG54800_03860,CCUG548	T151023_03250	T160124_01840,T160124_02970
12	15	0.145	Methionine import ATP-binding protein MetN	ATCC29328_13900	ATCC53516_05030,ATCC5351	07T609_13350	08T492_14450	09T408_12790	09T494_16420	12T272_13770	12T273_06420	12T306_04600	CCUG54800_03150,CCUG548	T151023_03240	T160124_01830,T160124_02960

12	12	1	hypothetical protein	ATCC29328_13910	ATCC53516_05040	07T609_13360	08T492_14460	09T408_12800	09T494_16430	12T272_13780	12T273_06410	12T306_04590	CCUG54800_03840	T151023_03230	T160124_02950
12	12	1	Ribonucleoside-diphosphate reductase subuni	ATCC29328_13920	ATCC53516_05050	07T609_13370	08T492_14470	09T408_12810	09T494_16440	12T272_13790	12T273_06400	12T306_04580	CCUG54800_03830	T151023_03220	T160124_02940
12	12	1	maltose/maltodextrin transporter ATP-binding	ATCC29328_13930	ATCC53516_05060	07T609_13380	08T492_14480	09T408_12820	09T494_16450	12T272_13800	12T273_06390	12T306_04570	CCUG54800_03820	T151023_03210	T160124_02930
12	12	1	hypothetical protein	ATCC29328_13940	ATCC53516_05070	07T609_13390	08T492_14490	09T408_12830	09T494_16460	12T272_13810	12T273_06380	12T306_04560	CCUG54800_03810	T151023_03200	T160124_02920
12	12	1	putative membrane protein YdfK	ATCC29328_13950	ATCC53516_05080	07T609_13400	08T492_14500	09T408_12840	09T494_16470	12T272_13820	12T273_06370	12T306_04550	CCUG54800_03800	T151023_03190	T160124_02910
7	9	0.445	hypothetical protein	ATCC29328_13960	ATCC53516_05090	*	*	09T408_16430,09T408_	09T494_01110	*	12T273_00530	*	CCUG54800_03750,CCUG548	T151023_00150	*
3	4	0.36	hypothetical protein	ATCC29328_13970	*	07T609_14510	08T492_13850,08T492_13	*	*	*	*	*	*	*	*
1	1	1	hypothetical protein	ATCC29328_13980	*	*	*	*	*	*	*	*	*	*	*
12	12	1	Coenzyme PQQ synthesis protein D	ATCC29328_13990	ATCC53516_05100	07T609_13410	08T492_14510	09T408_12860	09T494_16480	12T272_13850	12T273_06330	12T306_04540	CCUG54800_03740	T151023_03180	T160124_02900
12	12	1	OPT oligopeptide transporter protein	ATCC29328_14000	ATCC53516_05110	07T609_13420	08T492_14520	09T408_12870	09T494_16490	12T272_13860	12T273_06320	12T306_04530	CCUG54800_03730	T151023_03170	T160124_02890
12	12	1	hypothetical protein	ATCC29328_14010	ATCC53516_05120	07T609_13430	08T492_14530	09T408_12880	09T494_16500	12T272_13870	12T273_06310	12T306_04520	CCUG54800_03720	T151023_03160	T160124_02880
12	12	1	PTS-dependent dihydroxyacetone kinase, ADP-	ATCC29328_14050	ATCC53516_00010	07T609_13440	08T492_14550	09T408_12900	09T494_16520	12T272_13890	12T273_17080	12T306_15090	CCUG54800_14960	T151023_14040	T160124_12840
12	12	1	Aldehyde dehydrogenase	ATCC29328_14070	ATCC53516_00030	07T609_13460	08T492_14580	09T408_12920	09T494_16540	12T272_13910	12T273_17100	12T306_15110	CCUG54800_14980	T151023_14060	T160124_12860
12	15	0.145	Tyrosine-protein kinase YwqD	ATCC29328_14080	ATCC53516_00040	07T609_13470	08T492_14590	09T408_14510,09T408_	09T494_17410,09T494_	12T272_13920,12T272_	12T273_17110	12T306_15120	CCUG54800_14990	T151023_14070	T160124_12870
12	12	1	Capsular polysaccharide type 8 biosynthesis pr	ATCC29328_14090	ATCC53516_00050	07T609_13480	08T492_14600	09T408_12940	09T494_16560	12T272_13930	12T273_17120	12T306_15130	CCUG54800_15000	T151023_14080	T160124_12880
12	15	0.145	Tyrosine-protein phosphatase YwqE	ATCC29328_14100	ATCC53516_00060	07T609_13490	08T492_14610	09T408_14500,09T408_	09T494_17400,09T494_	12T272_13940,12T272_	12T273_17130	12T306_15140	CCUG54800_15010	T151023_14090	T160124_12890
12	12	1	Bacterial SH3 domain protein	ATCC29328_14110	ATCC53516_00070	07T609_13500	08T492_14620	09T408_12960	09T494_16580	12T272_13950	12T273_17140	12T306_15150	CCUG54800_15020	T151023_14100	T160124_12900
3	3	1	hypothetical protein	ATCC29328_14120	*	*	*	*	*	12T272_13960	*	*	*	T151023_14110	*
3	3	1	hypothetical protein	ATCC29328_14130	*	*	*	*	*	12T272_13970	*	*	*	T151023_14120	*
12	12	1	topology modulation protein	ATCC29328_14140	ATCC53516_00080	07T609_13510	08T492_14630	09T408_12970	09T494_16590	12T272_13980	12T273_17150	12T306_15160	CCUG54800_15030	T151023_14130	T160124_12910
1	1	1	hypothetical protein	ATCC29328_14150	*	*	*	*	*	*	*	*	*	*	*
6	6	0.167	hypothetical protein	ATCC29328_14160	ATCC53516_00140	07T609_13550	*	*	*	*	12T273_17170	12T306_15180	CCUG54800_15050	*	*
8	8	1	Cna protein B-type domain protein	ATCC29328_14170	ATCC53516_00150	07T609_13570	08T492_14650	*	*	*	12T273_17180	*	CCUG54800_15060	T151023_14140	T160124_12940
12	12	1	putative acyltransferase	ATCC29328_14180	ATCC53516_00160	07T609_13580	08T492_14660	09T408_13040	09T494_16600	12T272_14010	12T273_17190	12T306_15190	CCUG54800_15070	T151023_14160	T160124_12950
10	10	1	Integral membrane protein (intg_mem_TP038	ATCC29328_14190	ATCC53516_00170	07T609_13590	08T492_14670	09T408_13050	*	*	12T273_17200	12T306_15200	CCUG54800_15080	T151023_14170	T160124_12960
12	12	1	Peptidase family S51	ATCC29328_14200	ATCC53516_00180	07T609_13600	08T492_14680	09T408_13060	09T494_16610	12T272_14020	12T273_17210	12T306_15210	CCUG54800_15090	T151023_14180	T160124_12970
5	5	1	HIRAN domain protein	ATCC29328_14210	*	*	*	*	*	*	12T273_01400	12T306_15220	*	T151023_00980	T160124_00230
2	2	1	DnaJ domain protein	ATCC29328_14220	*	*	*	*	*	*	*	12T306_15230	*	*	*
12	12	1	outer membrane-specific lipoprotein transpor	ATCC29328_14230	ATCC53516_00190	07T609_13610	08T492_14690	09T408_13070	09T494_16620	12T272_14030	12T273_17220	12T306_15240	CCUG54800_15100	T151023_14190	T160124_12980
12	12	1	Bacterial regulatory proteins, tetR family	ATCC29328_14250	ATCC53516_00210	07T609_13630	08T492_14710	09T408_13090	09T494_16640	12T272_14050	12T273_17240	12T306_15260	CCUG54800_15120	T151023_14210	T160124_13000
11	11	1	Bifunctional AAC/APH	ATCC29328_14260	ATCC53516_00250	07T609_13640	08T492_14720	09T408_13100	09T494_16660	12T272_14070	12T273_17250	12T306_15270	*	T151023_14220	T160124_13010
12	12	1	dihydropteridine reductase	ATCC29328_14270	ATCC53516_00260	07T609_13650	08T492_14730	09T408_13110	09T494_16670	12T272_14080	12T273_17260	12T306_15280	CCUG54800_15140	T151023_14230	T160124_13020
12	12	1	Pyridoxamine 5'-phosphate oxidase	ATCC29328_14290	ATCC53516_00290	07T609_13670	08T492_14750	09T408_13130	09T494_16700	12T272_14110	12T273_17290	12T306_15310	CCUG54800_15170	T151023_14260	T160124_13050
12	12	1	DNA alkylation repair enzyme	ATCC29328_14300	ATCC53516_00300	07T609_13680	08T492_14760	09T408_13140	09T494_16710	12T272_14120	12T273_17300	12T306_15320	CCUG54800_15180	T151023_14270	T160124_13060
12	12	1	hypothetical protein	ATCC29328_14310	ATCC53516_00310	07T609_13690	08T492_14770	09T408_13150	09T494_16720	12T272_14130	12T273_17310	12T306_15330	CCUG54800_15190	T151023_14280	T160124_13070
12	12	1	Putative lipid kinase YtlR	ATCC29328_14320	ATCC53516_00320	07T609_13700	08T492_14780	09T408_13160	09T494_16730	12T272_14140	12T273_17320	12T306_15340	CCUG54800_15200	T151023_14290	T160124_13080
5	6	1	N-acetylmuramoyl-L-alanine amidase LytC prec	ATCC29328_14330	*	07T609_13710	08T492_14810	*	*	*	*	12T306_15360,12T306_	CCUG54800_15210	*	*
4	4	1	hypothetical protein	ATCC29328_14340	*	07T609_13720	08T492_14820	09T408_13180	*	*	*	*	*	*	*
11	13	0.154	ABC transporter ATP-binding protein YtrE	ATCC29328_14350	ATCC53516_00410	07T609_09310,07T609_	08T492_14830,08T492_09	09T408_13190	09T494_16790	12T272_14220	*	12T306_15400	CCUG54800_15260	T151023_14330	T160124_13170
11	11	1	hypothetical protein	ATCC29328_14370	ATCC53516_00430	07T609_13750	08T492_14850	09T408_13210	09T494_16810	12T272_14240	*	12T306_15420	CCUG54800_15280	T151023_14350	T160124_13190
12	12	1	HTH-type transcriptional activator CmpR	ATCC29328_14380	ATCC53516_00470	07T609_13760	08T492_14860	09T408_13250	09T494_16850	12T272_14280	12T273_17420	12T306_15430	CCUG54800_15290	T151023_14360	T160124_13230
12	12	1	TVP38/TMEM64 family inner membrane prote	ATCC29328_14390	ATCC53516_00480	07T609_13770	08T492_14870	09T408_13260	09T494_16860	12T272_14290	12T273_17430	12T306_15440	CCUG54800_15300	T151023_14370	T160124_13240
12	12	1	Adenylosuccinate lyase	ATCC29328_14400	ATCC53516_00490	07T609_13780	08T492_14880	09T408_13270	09T494_16870	12T272_14300	12T273_17440	12T306_15450	CCUG54800_15310	T151023_14380	T160124_13250
12	12	1	Phosphoribosylamine--glycine ligase	ATCC29328_14410	ATCC53516_00500	07T609_13790	08T492_14890	09T408_13280	09T494_16880	12T272_14310	12T273_17450	12T306_15460	CCUG54800_15320	T151023_14390	T160124_13260
12	12	1	Bifunctional purine biosynthesis protein PurH	ATCC29328_14420	ATCC53516_00510	07T609_13800	08T492_14900	09T408_13290	09T494_16890	12T272_14320	12T273_17460	12T306_15470	CCUG54800_15330	T151023_14400	T160124_13270
12	12	1	Phosphoribosylglycinamide formyltransferase	ATCC29328_14430	ATCC53516_00520	07T609_13810	08T492_14910	09T408_13300	09T494_16900	12T272_14330	12T273_17470	12T306_15480	CCUG54800_15340	T151023_14410	T160124_13280
12	12	1	Amidophosphoribosyltransferase precursor	ATCC29328_14440	ATCC53516_00530	07T609_13820	08T492_14920	09T408_13310	09T494_16910	12T272_14340	12T273_17480	12T306_15490	CCUG54800_15350	T151023_14420	T160124_13290
12	12	1	Phosphoribosylaminoimidazole-succinocarbox	ATCC29328_14450	ATCC53516_00540	07T609_13830	08T492_14930	09T408_13320	09T494_16920	12T272_14350	12T273_17490	12T306_15500	CCUG54800_15360	T151023_14430	T160124_13300
12	12	1	hypothetical protein	ATCC29328_14460	ATCC53516_00550	07T609_13840	08T492_14940	09T408_13330	09T494_16930	12T272_14360	12T273_17500	12T306_15510	CCUG54800_15370	T151023_14440	T160124_13310
9	9	1	Voltage-gated potassium channel Kch	ATCC29328_14470	ATCC53516_00570	07T609_13850	08T492_14950	09T408_13340	*	*	12T273_17530	12T306_15530	CCUG54800_15390	*	T160124_13320
8	8	1	NADH oxidase	ATCC29328_14480	*	07T609_13860	08T492_14960	09T408_13370	*	*	12T273_17560	12T306_15560	CCUG54800_15420	*	T160124_13350
4	4	1	Putative aminoacrylate hydrolase RutD	ATCC29328_14490	ATCC53516_00610	07T609_13870	08T492_14970	*	*	*	*	*	*	*	*
12	12	1	NADP-reducing hydrogenase subunit HndA	ATCC29328_14500	ATCC53516_00620	07T609_13880	08T492_14980	09T408_13380	09T494_16970	12T272_14400	12T273_17570	12T306_15590	CCUG54800_15430	T151023_14460	T160124_13360
11	11	0.364	hypothetical protein	ATCC29328_14510	ATCC53516_00630	07T609_13890	08T492_14990	09T408_13390	09T494_16980	12T272_14410	*	12T306_15600	CCUG54800_15440	T151023_14470	T160124_13370
4	4	1	hypothetical protein	ATCC29328_14520	ATCC53516_00640	*	*	*	*	*	*	*	*	T151023_03140	T160124_13450
10	10	1	hypothetical protein	ATCC29328_14530	ATCC53516_00650	07T609_13910	08T492_15010	09T408_13410	*	12T272_14430	*	12T306_15630	CCUG54800_15470	T151023_03130	T160124_13460
9	9	0.667	putative Mg(2+) transport ATPase	ATCC29328_14540	*	07T609_13920	08T492_15020	09T408_13420	09T494_17010	12T272_14440	*	12T306_15640	*	T151023_03120	T160124_13470
1	1	1	hypothetical protein	ATCC29328_14550	*	*	*	*	*	*	*	*	*	*	*
2	2	1	hypothetical protein	ATCC29328_14560	*	*	*	*	*	*	*	*	*	T151023_03060	*
1	2	1	Type-1 restriction enzyme R protein	ATCC29328_14570,ATCC2932(	*	*	*	*	*	*	*	*	*	*	*
1	1	1	hypothetical protein	ATCC29328_14580	*	*	*	*	*	*	*	*	*	*	*
1	1	1	Type I restriction modification DNA specificity (	ATCC29328_14590	*	*	*	*	*	*	*	*	*	*	*
1	1	1	Tyrosine recombinase XerC	ATCC29328_14600	*	*	*	*	*	*	*	*	*	*	*
1	1	1	Putative type-1 restriction enzyme specificity p	ATCC29328_14610	*	*	*	*	*	*	*	*	*	*	*
3	4	0.36	putative type I restriction enzymeP M protein	ATCC29328_14620,ATCC2932(	*	*	*	09T408_00390	*	*	*	*	*	*	T160124_00280
1	1	1	hypothetical protein	ATCC29328_14630	*	*	*	*	*	*	*	*	*	*	*
10	10	1	hypothetical protein	ATCC29328_14640	ATCC53516_00710	07T609_13960	08T492_15060	09T408_13480	*	12T272_14470	12T273_17670	12T306_15680	CCUG54800_15540	*	T160124_13540
10	10	1	alpha/beta hydrolase fold protein	ATCC29328_14650	ATCC53516_00720	07T609_13970	08T492_15070	09T408_13490	*	*	12T273_17680	12T306_15690	CCUG54800_15550	T151023_03030	T160124_13550
10	10	1	hypothetical protein	ATCC29328_14660	ATCC53516_00730	07T609_13980	08T492_15080	09T408_13500	*	*	12T273_17690	12T306_15700	CCUG54800_15560	T151023_03020	T160124_13560
10	12	0.233	putative methyltransferase YcgJ	ATCC29328_14670	ATCC53516_00740	07T609_14470,07T609_	08T492_15320,08T492_15	09T408_13510	*	*	12T273_17700	12T306_15710	CCUG54800_15570	T151023_03010	T160124_13570
1	1	1	hypothetical protein	ATCC29328_14680	*	*	*	*	*	*	*	*	*	*	*
1	1	1	hypothetical protein	ATCC29328_14690	*	*	*	*	*	*	*	*	*	*	*
1	1	1	hypothetical protein	ATCC29328_14700	*	*	*	*	*	*	*	*	*	*	*
1	1	1	hypothetical protein	ATCC29328_14710	*	*	*	*	*	*	*	*	*	*	*
1	1	1	hypothetical protein	ATCC29328_14720	*	*	*	*	*	*	*	*	*	*	*
1	1	1	sensory histidine kinase UhpB	ATCC29328_14730	*	*	*	*	*	*	*	*	*	*	*
1	1	1	Response regulator protein VraR	ATCC29328_14740	*	*	*	*	*	*	*	*	*	*	*
4	4	1	hypothetical protein	ATCC29328_14750	ATCC53516_04830	*	*	*	*	*	12T273_06070	*	*	*	T160124_02630
4	4	1	LOG family protein ORF6 in fasciation locus	ATCC29328_14760	*	*	*	09T408_13530	09T494_17070	*	*	*	*	*	T160124_13500
1	1	1	enterobactin exporter EntS	ATCC29328_14770	*	*	*	*	*	*	*	*	*	*	*
1	1	1	hypothetical protein	ATCC29328_14780	*	*	*	*	*	*	*	*	*	*	*
10	14	0.132	AMP nucleosidase	ATCC29328_14790	ATCC53516_01040,ATCC5351(	*	08T492_15100,08T492_15	09T408_13690	*	12T272_14490	12T273_17710,12T273_	12T306_15720,12T306_	CCUG54800_15700	T151023_03000	T160124_13580
10	11	0.252	putative ABC transporter ATP-binding protein	ATCC29328_14800	ATCC53516_00780	*	08T492_15110	09T408_13540	09T494_17080	12T272_14510	*	12T306_15670	CCUG54800_15710,CCUG548	T151023_02990	T160124_13510
11	11	1	hypothetical protein	ATCC29328_14810	ATCC53516_00790	*	08T492_15120	09T408_13550	09T494_17090	12T272_14520	12T273_03900	12T306_15730	CCUG54800_15720	T151023_02980	T160124_13600
11	11	1	Putative HMP/thiamine import ATP-binding pr	ATCC29328_14820	ATCC53516_00800	07T609_14050	08T492_15130	09T408_13560	09T494_02970	12T272_01170	12T273_17740	12T306_15740	CCUG54800_15730	*	T160124_13610
10	14	0.141	Energy-coupling factor transporter transmemb	ATCC29328_14830	*	07T609_14060	08T492_09400,08T492_15	09T408_14090,09T408_	09T494_02960	12T272_01160	12T273_13010,12T273_	12T306_11530,12T306_	*	T151023_02960	T160124_13620
11	15	0.182	hypothetical protein	ATCC29328_14840	ATCC53516_00820	07T609_14070	08T492_13120,08T492_15	09T408_13580	09T494_13760,09T494_	12T272_12900,12T272_	12T273_17760,12T273_	12T306_15760	*	T151023_02950	T160124_13630
12	19	0.141	Multidrug export protein MepA	ATCC29328_14850,ATCC2932(	ATCC53516_01090,ATCC5351(	07T609_14080	08T492_15160,08T492_13	09T408_13590	09T494_13710	12T272_17030	12T273_03870,12T273_	12T306_15770	CCUG54800_15760,CCUG548	T151023_02860	T160124_13970,T160124_13840,T160124_13640
12	16	0.107	Heterocyst differentiation ATP-binding protein	ATCC29328_14860	ATCC53516_00840	07T609_14090,07T609_	08T492_13070,08T492_15	09T408_13600	09T494_02940	12T272_01140	12T273_04090,12T273_	12T306_15780,12T306_	CCUG54800_15770	T151023_02940	T160124_13650
12	13	0.293	Iron import ATP-binding/permease protein Irt/	ATCC29328_14870	ATCC53516_00850	07T609_14100	08T492_13080,08T492_15	09T408_13610	09T494_02930	12T272_01130	12T273_04080	12T306_15790	CCUG54800_15780	T151023_02930	T160124_13660
11	12	0.313	Regulatory protein SoxS	ATCC29328_14880	ATCC53516_00860	07T609_14110	08T492_13090,08T492_15	09T408_13620	09T494_02920	12T272_01120	12T273_04070	12T306_15800	*	T151023_02920	T160124_13670
4	4	1	ABC-2 family transporter protein	ATCC29328_14890	*	07T609_14120	08T492_15200	09T408_13630	*	*	*	*	*	*	*
4	4	1	ABC-2 family transporter protein	ATCC29328_14900	*	07T609_14130	08T492_15210	09T408_13640	*	*	*	*	*	*	*
10	16	0.17	putative ABC transporter ATP-binding protein '	ATCC29328_14910	ATCC53516_07960	07T609_14140	08T492_13340,08T492_13	09T408_13650	*	12T272_17310,12T272_	12T273_17910,12T273_	12T306_02840,12T306_	CCUG54800_19210,CCUG548	*	T160124_02430
4	4	1	Sensory transduction protein regX3	ATCC29328_14920	*	07T609_14150	08T492_15230	09T408_13660	*	*	*	*	*	*	*
4	4	1	Sensor protein BasS	ATCC29328_14930	*	07T609_14160	08T492_15240	09T408_13670	*	*	*	*	*	*	*
1	1	1	hypothetical protein	ATCC29328_14940	*	*	*	*	*	*	*	*	*	*	*
10	15	0.167	Putative multidrug export ATP-binding/permea	ATCC29328_14950	ATCC53516_01200,ATCC5351(	07T609_14290,07T609_	08T492_15440	*	09T494_15590	12T272_17010,12T272_	12T273_03760	12T306_16130,12T306_	CCUG54800_16070,CCUG548	*	T160124_13950
11	16	0.219	Multidrug resistance ABC transporter ATP-binc	ATCC29328_14960	ATCC53516_01210,ATCC5351(	07T609_14300,07T609_	08T492_15450	09T408_14120	09T494_15600	12T272_12610,12T272_	12T273_03750	12T306_15830,12T306_	CCUG54800_16080,CCUG548	*	T160124_13960
8	10	0.264	Putative HMP/thiamine import ATP-binding pr	ATCC29328_14980	ATCC53516_01120	*	*	*	09T494_15610	12T272_17040,12T272_	12T273_03840	12T306_11090	CCUG54800_14350	*	T160124_13870,T160124_13980
8	10	0.34	Cobalt transport protein	ATCC29328_14990	ATCC53516_01130	*	*	*	09T494_15620	12T272_12630,12T272_	12T273_03830	12T306_11100	CCUG54800_14360	*	T160124_13880,T160124_13990
8	10	0.365	hypothetical protein	ATCC29328_15000	ATCC53516_01140	*	*	*	09T494_15630	12T272_12640,12T272_	12T273_03820	12T306_11110	CCUG54800_14370	*	T160124_13890,T160124_14000
1	1	1	hypothetical protein	ATCC29328_15030	*	*	*	*	*	*	*	*	*	*	*
1	1	1	hypothetical protein	ATCC29328_15040	*	*	*	*	*	*	*	*	*	*	*
1	1	1	hypothetical protein	ATCC29328_15050	*	*	*	*	*	*	*	*	*	*	*
2	3	1	hypothetical protein	ATCC29328_15060	*	*	08T492_15540,08T492_17	*	*	*	*	*	*	*	*
10	16	0.156	hypothetical protein	ATCC29328_15070,ATCC2932(	ATCC53516_11600	07T609_16390,07T609_	08T492_13590,08T492_17	*	09T494_00980	12T272_08050	12T273_12920	12T306_17160	*	T151023_15860,T151023_	T160124_14100,T160124_09540
12	13	0.141	23S rRNA (uracil-C(5))-methyltransferase RlmC	ATCC29328_15080	ATCC53516_01710	07T609_14580	08T492_15560	09T408_14200	09T494_11110	12T272_12950	12T273_03270	12T306_02690,12T306_	CCUG54800_16580	T151023_02730	T160124_14110
12	12	1	Putative multidrug export ATP-binding/permea	ATCC29328_15100	ATCC53516_01730	07T609_14600	08T492_15580	09T408_14220	09T494_11150	12T272_12990	12T273_03250	12T306_02670	CCUG54800_16600	T151023_02690	T160124_14130
12	12	1	Transcriptional repressor MprA	ATCC29328_15110	ATCC53516_01740	07T609_14610	08T492_15590	09T408_14230	09T494_11160	12T272_13000	12T273_03240	12T306_02660	CCUG54800_16610	T151023_02680	T160124_14140
12	12	1	Phosphomethylpyrimidine synthase	ATCC29328_15120	ATCC53516_01750	07T609_14620	08T492_15600	09T408_14240	09T494_11170	12T272_13010	12T273_03230	12T306_02650	CCUG54800_16620	T151023_02670	T160124_14150
12	12	1	Cyclic pyranopterin monophosphate synthase	ATCC29328_15130	ATCC53516_01760	07T609_14630	08T492_15610	09T408_14250	09T494_11180	12T272_13020	12T273_03220	12T306_02640	CCUG54800_16630	T151023_02660	T160124_14160
12	12	1	Cyclic pyranopterin monophosphate synthase	ATCC29328_15140	ATCC53516_01770	07T609_14640	08T492_15620	09T408_14260	09T494_11190	12T272_13030	12T273_03210	12T306_02630	CCUG54800_16640	T151023_02650	T160124_14170
12	12	1	hypothetical protein	ATCC29328_15150	ATCC53516_01780	07T609_14650	08T492_15630	09T408_14270	09T494_11200	12T272_13040	12T273_03200	12T306_02620	CCUG54800_16650	T151023_02640	T160124_14180
12	12	1	hypothetical protein	ATCC29328_15160	ATCC53516_01790	07T609_14660	08T492_15640	09T408_14280	09T494_11210	12T272_13050	12T273_03190	12T306_02610	CCUG54800_16660	T151023_02630	T160124_14190
12	12	1	hypothetical protein	ATCC29328_15170	ATCC53516_01800	07T609_14670	08T492_15650	09T408_14290	09T494_11220	12T272_13060	12T273_03180	12T306_02600	CCUG54800_16670	T151023_02620	T160124_14200
12	12	1	hypothetical protein	ATCC29328_15180	ATCC53516_01810	07T609_14680	08T492_15660	09T408_14300	09T494_11230	12T272_13070	12T273_03170	12T306_02590	CCUG54800_16680	T151023_02610	T160124_14210
12	14	0.143	Nucleoside recognition	ATCC29328_15190	ATCC53516_01820	07T609_16760,07T609_	08T492_15670	09T408_14310	09T494_11240	12T272_13080	12T273_03160	12T306_02580,12T306_	CCUG54800_16690	T151023_02600	T160124_14220
11	11	1	hypothetical protein	ATCC29328_15200	ATCC53516_01830	07T609_14700	08T492_15680	09T408_14320	09T494_03820	12T272_13090	12T273_03150	12T306_02570	CCUG54800_16700	*	T160124_14230
9	9	1	26 kDa periplasmic immunogenic protein prec	ATCC29328_15210	ATCC53516_01840	07T609_14710	08T492_15690	09T408_14330	*	*	12T273_03140	*	CCUG54800_16710	T151023_02590	T160124_14240
9	10	0.5	KilA-N domain protein	ATCC29328_15220	*	07T609_14720	08T492_13000	*	09T494_13650,09T494_	12T272_16950	12T273_16570	*	CCUG54800_14250	T151023_02580	T160124_14260
3	3	1	hypothetical protein	ATCC29328_15230	ATCC53516_01880	07T609_14730	*	*	*	*	*	*	*	*	*
12	12	1	Dipeptidase	ATCC29328_15240	ATCC53516_01890	07T609_14740	08T492_15700	09T408_14530	09T494_17120	12T272_14560	12T273_04930	12T306_03510	CCUG54800_02840	T151023_02570	T160124_14520
12	12	1	Non-specific ribonucleoside hydrolase RihC	ATCC29328_15250	ATCC53516_08930	07T609_14750	08T492_15710	09T408_14540	09T494_17430	12T272_14830	12T273_04870	12T306_03440	CCUG54800_16850	T151023_13720	T160124_14580
12	12	1	Cobalt import ATP-binding protein CbiO	ATCC29328_15260	ATCC53516_08940	07T609_14760	08T492_15720	09T408_14550	09T494_17440	12T272_14840	12T273_04860	12T306_03430	CCUG54800_16860	T151023_13730	T160124_14590
12	12	1	Energy-coupling factor transporter ATP-bindin	ATCC29328_15270	ATCC53516_08950	07T609_14770	08T492_15730	09T408_14560	09T494_17450	12T272_14850	12T273_04840	12T306_03420	CCUG54800_16870	T151023_13740	T160124_14600
12	12	1	Energy-coupling factor transporter transmemb	ATCC29328_15280	ATCC53516_08960	07T609_14780	08T492_15740	09T408_14570	09T494_17460	12T272_14860	12T273_04830	12T306_03410	CCUG54800_16880	T151023_13750	T160124_14610
12	17	0.149	hypothetical protein	ATCC29328_15290	ATCC53516_09450,ATCC5351(	07T609_15210,07T609_	08T492_15750	09T408_14580	09T494_17470	12T272_14870	12T273_04340,12T273_	12T306_03400	CCUG54800_16890,CCUG548	T151023_13760	T160124_15120,T160124_14620
12	12	1	Acetyltransferase (GNAT) family protein	ATCC29328_15300	ATCC53516_08980	07T609_14800	08T492_15760	09T408_14590	09T494_17480	12T272_14880	12T273_04810	12T306_03390	CCUG54800_16900	T151023_13770	T160124_14630
12	12	1	30S ribosomal protein S1	ATCC29328_15310	ATCC53516_09000	07T609_14830	08T492_15790	09T408_14610	09T494_17490	12T272_14890	12T273_04800	12T306_03370	CCUG54800_16920	T151023_13790	T160124_14660
12	12	1	hypothetical protein	ATCC29328_15320	ATCC53516_09010	07T609_14840	08T492_15800	09T408_14620	09T494_17500	12T272_14900	12T273_04790	12T306_03360	CCUG54800_16930	T151023_13800	T160124_14670
12	12	1	Uridine kinase	ATCC29328_15330	ATCC53516_09020	07T609_14850	08T492_15810	09T408_14630	09T494_17510	12T272_14910	12T273_04780	12T306_03350	CCUG54800_16940	T151023_13810	T160124_14680
12	12	1	Tyrosine--tRNA ligase	ATCC29328_15340	ATCC53516_09030	07T609_14860	08T492_15820	09T408_14640	09T494_17520	12T272_14920	12T273_04770	12T306_03340	CCUG54800_16950	T151023_13820	T160124_14690
12	13	0.141	Cardiolipin synthase	ATCC29328_15350	ATCC53516_09040	07T609_14870,07T609_(	08T492_15830	09T408_14650	09T494_17530	12T272_14940	12T273_04760	12T306_03330	CCUG54800_16960	T151023_13830	T160124_14700
12	12	1	Acyltransferase family protein	ATCC29328_15360	ATCC53516_09050	07T609_14880	08T492_15840	09T408_14660	09T494_17540	12T272_14950	12T273_04750	12T306_03320	CCUG54800_16970	T151023_13840	T160124_14710
12	12	1	Putative lipid kinase	ATCC29328_15370	ATCC53516_09060	07T609_14890	08T492_15850	09T408_14670	09T494_17550	12T272_14960	12T273_04740	12T306_03310	CCUG54800_16980	T151023_13850	T160124_14720
9	9	1	N-acetylmuramoyl-L-alanine amidase LytC prec	ATCC29328_15380	ATCC53516_09080	*	08T492_15860	*	09T494_17560	12T272_14970	12T273_04730	12T306_03300	*	T151023_13860	T160124_14730
12	12	1	Glycogen synthase	ATCC29328_15390	ATCC53516_09090	07T609_14900	08T492_15880	09T408_14690	09T494_17570	12T272_14980	12T273_04720	12T306_03290	CCUG54800_16990	T151023_13920	T160124_14740
12	13	0.141	Multidrug export protein MepA	ATCC29328_15400	ATCC53516_09100	07T609_14910	08T492_15890,08T492_09	09T408_14700	09T494_17580	12T272_14990	12T273_04710	12T306_03280	CCUG54800_17000	T151023_13930	T160124_14750
12	12	1	hypothetical protein	ATCC29328_15410	ATCC53516_09110	07T609_14920	08T492_15900	09T408_14710	09T494_17590	12T272_15000	12T273_04700	12T306_03270	CCUG54800_17010	T151023_13940	T160124_14760
12	12	1	Putative modulator of DNA gyrase	ATCC29328_15420	ATCC53516_09120	07T609_14930	08T492_15910	09T408_14720	09T494_17600	12T272_15010	12T273_04690	12T306_03260	CCUG54800_17020	T151023_13950	T160124_14770
12	12	1	protease TldD	ATCC29328_15430	ATCC53516_09130	07T609_14940	08T492_15920	09T408_14730	09T494_17610	12T272_15020	12T273_04680	12T306_03250	CCUG54800_17030	T151023_13960	T160124_14780
12	12	1	Pyridoxal biosynthesis lyase PdxS	ATCC29328_15440	ATCC53516_09150	07T609_14960	08T492_15940	09T408_14740	09T494_17620	12T272_15030	12T273_04660	12T306_03240	CCUG54800_17040	T151023_13980	T160124_14790
12	12	1	HTH-type transcriptional regulatory protein Ga	ATCC29328_15450	ATCC53516_09160	07T609_14970	08T492_15990	09T408_14750	09T494_17630	12T272_15070	12T273_04650	12T306_03230	CCUG54800_17050	T151023_14020	T160124_14800
12	12	1	Cytosol non-specific dipeptidase	ATCC29328_15460	ATCC53516_09170	07T609_14980	08T492_16000	09T408_14760	09T494_17640	12T272_15080	12T273_04640	12T306_03220	CCUG54800_17060	T151023_14030	T160124_14810
10	10	1	hypothetical protein	ATCC29328_15470	ATCC53516_09180	07T609_14990	08T492_16010	*	*	12T272_15090	12T273_04630	12T306_03210	CCUG54800_17070	T151023_14480	T160124_14820
12	12	1	hypothetical protein	ATCC29328_15480	ATCC53516_09190	07T609_15000	08T492_16030	09T408_14770	09T494_17650	12T272_15100	12T273_04620	12T306_03200	CCUG54800_17080	T151023_14490	T160124_14830
12	12	1	Cystathionine beta-lyase PatB	ATCC29328_15490	ATCC53516_09200	07T609_15010	08T492_16040	09T408_14780	09T494_17660	12T272_15110	12T273_04610	12T306_03190	CCUG54800_17090	T151023_14500	T160124_14840
12	12	1	Large-conductance mechanosensitive channel	ATCC29328_15500	ATCC53516_09210	07T609_15020	08T492_16050	09T408_14790	09T494_17670	12T272_15120	12T273_04600	12T306_03180	CCUG54800_17100	T151023_14510	T160124_14850
12	12	1	hypothetical protein	ATCC29328_15510	ATCC53516_09220	07T609_15030	08T492_16060	09T408_14800	09T494_17680	12T272_15130	12T273_04590	12T306_03170	CCUG54800_17110	T151023_14520	T160124_14860
3	3	1	Bacterial regulatory proteins, tetR family	ATCC29328_15520	*	07T609_15040	*	*	*	12T272_15140	*	*	*	*	*
3	3	1	Glycine betaine transporter OpuD	ATCC29328_15530	*	07T609_15050	*	*	*	12T272_15150	*	*	*	*	*
3	3	1	Betaine reductase complex component B subu	ATCC29328_15540	*	07T609_15060	*	*	*	12T272_15160	*	*	*	*	*
3	3	1	Betaine reductase complex component B subu	ATCC29328_15560	*	07T609_15080	*	*	*	12T272_15180	*	*	*	*	*
12	12	1	Tryptophan--tRNA ligase 2	ATCC29328_15570	ATCC53516_09230	07T609_15090	08T492_16070	09T408_14810	09T494_17690	12T272_15190	12T273_04580	12T306_03160	CCUG54800_17120	T151023_14530	T160124_14870
12	12	1	Capsule biosynthesis protein CapA	ATCC29328_15580	ATCC53516_09240	07T609_15100	08T492_16080	09T408_14820	09T494_17700	12T272_15200	12T273_04570	12T306_03150	CCUG54800_17130	T151023_14540	T160124_14880
12	12	1	Ribosomal RNA large subunit methyltransferas	ATCC29328_15590	ATCC53516_09250	07T609_15110	08T492_16090	09T408_14830	09T494_17710	12T272_15210	12T273_04560	12T306_03140	CCUG54800_17140	T151023_14550	T160124_14890
9	11	0.364	hypothetical protein	ATCC29328_15600,ATCC2932(	*	07T609_16200	08T492_17030	*	09T494_17720	12T272_16300	12T273_00040	12T306_03130	*	T151023_14560,T151023_	T160124_16040
3	3	1	YcfA-like protein	ATCC29328_15610	*	*	*	*	09T494_17730	*	*	*	*	T151023_14570	*
12	12	1	30S ribosomal protein S9	ATCC29328_15620	ATCC53516_09270	07T609_15120	08T492_16100	09T408_14890	09T494_17740	12T272_15230	12T273_04440	12T306_03120	CCUG54800_17150	T151023_14580	T160124_14900
12	12	1	50S ribosomal protein L13	ATCC29328_15630	ATCC53516_09280	07T609_15130	08T492_16110	09T408_14900	09T494_17750	12T272_15240	12T273_04430	12T306_03110	CCUG54800_17160	T151023_14590	T160124_14910
12	12	1	Cupin domain protein	ATCC29328_15640	ATCC53516_09290	07T609_15140	08T492_16120	09T408_14910	09T494_17760	12T272_15250	12T273_04420	12T306_03100	CCUG54800_17170	T151023_14600	T160124_14920
12	12	1	hypothetical protein	ATCC29328_15650	ATCC53516_09300	07T609_15150	08T492_16130	09T408_14920	09T494_17770	12T272_15260	12T273_04410	12T306_03090	CCUG54800_17180	T151023_14610	T160124_14930
12	12	1	hypothetical protein	ATCC29328_15660	ATCC53516_09310	07T609_15160	08T492_16140	09T408_14930	09T494_17780	12T272_15270	12T273_04390	12T306_03080	CCUG54800_17190	T151023_14620	T160124_14940
12	12	1	hypothetical protein	ATCC29328_15670	ATCC53516_09320	07T609_15170	08T492_16150	09T408_14940	09T494_17790	12T272_15290	12T273_04380	12T306_03070	CCUG54800_17200	T151023_14630	T160124_14950
12	12	1	hypothetical protein	ATCC29328_15680	ATCC53516_09420	07T609_15180	08T492_16210	09T408_15060	09T494_17880	12T272_15400	12T273_04370	12T306_02950	CCUG54800_17210	T151023_14720	T160124_15090
12	12	1	putative ABC transporter ATP-binding protein '	ATCC29328_15690	ATCC53516_09430	07T609_15190	08T492_16220	09T408_15070	09T494_17890	12T272_15410	12T273_04360	12T306_02940	CCUG54800_17220	T151023_14730	T160124_15100
12	12	1	hypothetical protein	ATCC29328_15700	ATCC53516_09440	07T609_15200	08T492_16230	09T408_15080	09T494_17900	12T272_15420	12T273_04350	12T306_02930	CCUG54800_17230	T151023_14740	T160124_15110
9	9	1	N-acetylmuramoyl-L-alanine amidase LytC prec	ATCC29328_15710	ATCC53516_09460	07T609_15220	08T492_16240	*	*	12T272_15430	12T273_04330	*	CCUG54800_17250	T151023_14750	T160124_15130
12	12	1	hypothetical protein	ATCC29328_15720	ATCC53516_09470	07T609_15230	08T492_16250	09T408_15100	09T494_17910	12T272_15440	12T273_04310	12T306_02920	CCUG54800_17270	T151023_14770	T160124_15150
12	12	1	hypothetical protein	ATCC29328_15730	ATCC53516_09480	07T609_15240	08T492_16260	09T408_15110	09T494_17920	12T272_15450	12T273_04300	12T306_02910	CCUG54800_17280	T151023_14780	T160124_15160
9	9	1	hypothetical protein	ATCC29328_15750	ATCC53516_09500	07T609_15260	08T492_16280	09T408_15130	09T494_17940	*	*	12T306_02890	CCUG54800_17300	T151023_14800	*
12	12	1	K(+)/H(+) antiporter NhaP2	ATCC29328_15760	ATCC53516_09510	07T609_15270	08T492_16290	09T408_15140	09T494_17950	12T272_15470	12T273_04280	12T306_16160	CCUG54800_17310	T151023_14810	T160124_15180
12	12	1	hypothetical protein	ATCC29328_15770	ATCC53516_09520	07T609_15280	08T492_16300	09T408_15150	09T494_17960	12T272_15480	12T273_04270	12T306_16170	CCUG54800_17320	T151023_14820	T160124_15190
11	11	1	Pyruvate formate-lyase-activating enzyme	ATCC29328_15780	ATCC53516_09530	07T609_15290	08T492_16310	09T408_15160	09T494_17970	12T272_15490	*	12T306_16180	CCUG54800_17330	T151023_14830	T160124_15200
11	11	1	Formate acetyltransferase	ATCC29328_15790	ATCC53516_09540	07T609_15300	08T492_16320	09T408_15170	09T494_17980	12T272_15500	*	12T306_16190	CCUG54800_17340	T151023_14840	T160124_15210
12	12	1	hypothetical protein	ATCC29328_15800	ATCC53516_09550	07T609_15310	08T492_16330	09T408_15180	09T494_17990	12T272_15510	12T273_04260	12T306_16200	CCUG54800_17350	T151023_14850	T160124_15220
12	12	1	Putative neutral zinc metallopeptidase	ATCC29328_15810	ATCC53516_09560	07T609_15320	08T492_16340	09T408_15190	09T494_18000	12T272_15520	12T273_04250	12T306_16210	CCUG54800_17360	T151023_14860	T160124_15230
12	12	1	DNA utilization protein GntX	ATCC29328_15820	ATCC53516_09570	07T609_15330	08T492_16350	09T408_15200	09T494_18010	12T272_15530	12T273_04240	12T306_16220	CCUG54800_17370	T151023_14870	T160124_15240
12	12	1	ATP-dependent RecD-like DNA helicase	ATCC29328_15830	ATCC53516_09580	07T609_15340	08T492_16360	09T408_15210	09T494_18020	12T272_15540	12T273_04230	12T306_16230	CCUG54800_17380	T151023_14880	T160124_15250
12	12	1	UDP-N-acetylglucosamine 1-carboxyvinyltransf	ATCC29328_15840	ATCC53516_09590	07T609_15350	08T492_16370	09T408_15220	09T494_18030	12T272_15550	12T273_04220	12T306_16240	CCUG54800_17390	T151023_14890	T160124_15260
8	8	1	N-acetylmuramoyl-L-alanine amidase LytC prec	ATCC29328_15850	ATCC53516_09600	07T609_15360	08T492_16380	*	09T494_18050	12T272_15560	12T273_04210	*	*	*	T160124_15270
12	12	1	Pantothenic acid transporter PanT	ATCC29328_15860	ATCC53516_09610	07T609_15370	08T492_16390	09T408_15240	09T494_18060	12T272_15570	12T273_04200	12T306_16260	CCUG54800_17410	T151023_14900	T160124_15280
12	12	1	L-serine dehydratase, alpha chain	ATCC29328_15870	ATCC53516_09630	07T609_15380	08T492_16400	09T408_15250	09T494_18070	12T272_15580	12T273_04190	12T306_16270	CCUG54800_17420	T151023_14910	T160124_15290
12	12	1	L-serine dehydratase, beta chain	ATCC29328_15880	ATCC53516_09640	07T609_15390	08T492_16410	09T408_15260	09T494_18080	12T272_15590	12T273_04180	12T306_16280	CCUG54800_17430	T151023_14920	T160124_15300
10	12	0.137	N-acetylmuramoyl-L-alanine amidase LytC prec	ATCC29328_15890,ATCC2932(	ATCC53516_09650	07T609_15400	08T492_16420	09T408_15750	*	*	12T273_04170,12T273_	12T306_16290	CCUG54800_17440	T151023_14930	T160124_15310
12	12	1	N-acetylmuramoyl-L-alanine amidase LytC prec	ATCC29328_15900	ATCC53516_09660	07T609_15410	08T492_16430	09T408_15270	09T494_18090	12T272_15600	12T273_04160	12T306_16300	CCUG54800_17450	T151023_14940	T160124_15320
12	12	1	Lipid A export ATP-binding/permease protein I	ATCC29328_15910	ATCC53516_09670	07T609_15420	08T492_16440	09T408_15280	09T494_18100	12T272_15610	12T273_04150	12T306_16310	CCUG54800_17460	T151023_14950	T160124_15330
12	12	1	Lipid A export ATP-binding/permease protein I	ATCC29328_15920	ATCC53516_09680	07T609_15430	08T492_16450	09T408_15290	09T494_18110	12T272_15620	12T273_04140	12T306_16320	CCUG54800_17470	T151023_14960	T160124_15340
12	12	1	Sensor histidine kinase DpiB	ATCC29328_15930	ATCC53516_09690	07T609_15440	08T492_16460	09T408_15300	09T494_18120	12T272_15630	12T273_04130	12T306_16330	CCUG54800_17480	T151023_14970	T160124_15350
12	19	0.153	Transcriptional regulatory protein YpdB	ATCC29328_15940	ATCC53516_12170,ATCC5351(	07T609_15450	08T492_16470	09T408_15670,09T408_	09T494_18530,09T494_	12T272_16020,12T272_	12T273_18470,12T273_	12T306_16340	CCUG54800_17490,CCUG548	T151023_14980	T160124_15740,T160124_15360
12	13	0.141	UTP--glucose-1-phosphate uridylyltransferase	ATCC29328_15950	ATCC53516_09710	07T609_15460	08T492_16480	09T408_15320	09T494_18140,09T494_	12T272_15650	12T273_04110	12T306_16350	CCUG54800_17500	T151023_14990	T160124_15370
12	12	1	Chaperone protein ClpB	ATCC29328_15960	ATCC53516_09720	07T609_15470	08T492_16490	09T408_15330	09T494_18150	12T272_15660	12T273_18050	12T306_16360	CCUG54800_17510	T151023_15000	T160124_15380
12	12	1	Curved DNA-binding protein	ATCC29328_15970	ATCC53516_09730	07T609_15480	08T492_16500	09T408_15340	09T494_18160	12T272_15670	12T273_18060	12T306_16370	CCUG54800_17520	T151023_15010	T160124_15390
12	12	1	Nitroreductase family protein	ATCC29328_15980	ATCC53516_09740	07T609_15490	08T492_16510	09T408_15350	09T494_18170	12T272_15680	12T273_18080	12T306_16380	CCUG54800_17530	T151023_15020	T160124_15400
12	13	0.141	RNA polymerase sigma factor	ATCC29328_15990	ATCC53516_12530	07T609_15500	08T492_16520	09T408_14340	09T494_18180,09T494_	12T272_15690	12T273_18090	12T306_16390	CCUG54800_17540	T151023_15030	T160124_15410
12	12	1	hypothetical protein	ATCC29328_16000	ATCC53516_12520	07T609_15510	08T492_16530	09T408_14350	09T494_18190	12T272_15700	12T273_18100	12T306_16400	CCUG54800_17550	T151023_15040	T160124_15420
1	1	1	Peptostreptococcal albumin-binding protein pr	ATCC29328_16010	*	*	*	*	*	*	*	*	*	*	*
11	11	1	hypothetical protein	ATCC29328_16020	ATCC53516_12500	07T609_15530	08T492_16550	09T408_15370	09T494_18220	12T272_15730	12T273_18120	12T306_16440	CCUG54800_17570	*	T160124_15440
11	11	1	hypothetical protein	ATCC29328_16030	ATCC53516_12490	07T609_15540	08T492_16560	09T408_15380	09T494_18230	12T272_15740	12T273_18130	12T306_16450	CCUG54800_17580	*	T160124_15450
10	10	1	Major Facilitator Superfamily protein	ATCC29328_16040	ATCC53516_12480	07T609_15550	08T492_16570	09T408_15390	*	12T272_15750	12T273_18140	12T306_16460	CCUG54800_17590	*	T160124_15460
11	11	1	Peptidase family M50	ATCC29328_16050	ATCC53516_12470	07T609_15560	08T492_16580	09T408_15400	09T494_18260	12T272_15760	12T273_18150	12T306_16470	CCUG54800_17600	*	T160124_15470
12	14	0.171	Putative zinc metalloprotease	ATCC29328_16060	ATCC53516_12440,ATCC5351(	07T609_15570	08T492_16590	09T408_15410	09T494_18270	12T272_15770	12T273_18160	12T306_16480	CCUG54800_17610	T151023_03550	T160124_15480,T160124_15490
8	8	0.25	hypothetical protein	ATCC29328_16070	ATCC53516_12450	07T609_15580	08T492_16600	*	*	*	12T273_18170	12T306_16490	CCUG54800_17620	T151023_15050	*
12	12	1	hypothetical protein	ATCC29328_16080	ATCC53516_12430	07T609_15590	08T492_16610	09T408_15420	09T494_18280	12T272_15780	12T273_18180	12T306_16500	CCUG54800_17630	T151023_15060	T160124_15500
12	12	1	Putative peroxiredoxin bcp	ATCC29328_16100	ATCC53516_12410	07T609_15610	08T492_16630	09T408_15440	09T494_18300	12T272_15800	12T273_18200	12T306_16520	CCUG54800_17650	T151023_15080	T160124_15520
12	12	1	Putative phosphatase	ATCC29328_16110	ATCC53516_12400	07T609_15620	08T492_16640	09T408_15450	09T494_18310	12T272_15810	12T273_18210	12T306_16530	CCUG54800_17660	T151023_15090	T160124_15530
12	12	1	hypothetical protein	ATCC29328_16120	ATCC53516_12390	07T609_15630	08T492_16650	09T408_15460	09T494_18320	12T272_15820	12T273_18220	12T306_16540	CCUG54800_17670	T151023_15100	T160124_15540
12	12	1	preprotein translocase subunit SecA	ATCC29328_16150	ATCC53516_12350	07T609_15660	08T492_16680	09T408_15500	09T494_18360	12T272_15860	12T273_18260	12T306_16580	CCUG54800_17710	T151023_15140	T160124_15580
6	6	1	putative transcriptional regulator	ATCC29328_16160	*	*	08T492_16950	09T408_15540	*	*	12T273_18310	*	*	T151023_15510	T160124_15950

1	1	1	Type-1 restriction enzyme EcoKI specificity pro	ATCC29328_16180	*	*	*	*	*	*	*	*	*	*	*
1	1	1	Histidine kinase-, DNA gyrase B-, and HSP90-lik	ATCC29328_16200	*	*	*	*	*	*	*	*	*	*	*
6	6	1	RelB antitoxin	ATCC29328_16210	ATCC53516_12340	07T609_15670	08T492_16690	*	*	12T272_15870	*	*	CCUG54800_17720	*	*
12	12	1	putative transaldolase	ATCC29328_16220	ATCC53516_12320	07T609_15690	08T492_16710	09T408_15550	09T494_18380	12T272_15890	12T273_18320	12T306_16590	CCUG54800_17740	T151023_15160	T160124_15590
12	12	1	Ascorbate-specific permease IIC component U	ATCC29328_16230	ATCC53516_12310	07T609_15700	08T492_16720	09T408_15560	09T494_18390	12T272_15900	12T273_18330	12T306_16600	CCUG54800_17750	T151023_15170	T160124_15600
12	12	1	Ascorbate-specific phosphotransferase enzym	ATCC29328_16240	ATCC53516_12300	07T609_15710	08T492_16730	09T408_15570	09T494_18400	12T272_15910	12T273_18340	12T306_16610	CCUG54800_17760	T151023_15180	T160124_15610
12	12	1	Ascorbate-specific phosphotransferase enzym	ATCC29328_16250	ATCC53516_12290	07T609_15720	08T492_16740	09T408_15580	09T494_18410	12T272_15920	12T273_18350	12T306_16620	CCUG54800_17770	T151023_15190	T160124_15620
11	11	1	Membrane transport protein	ATCC29328_16260	ATCC53516_12280	07T609_15730	08T492_16750	09T408_15590	09T494_18420	12T272_15930	12T273_18360	12T306_16630	CCUG54800_17780	*	T160124_15630
12	12	1	colanic acid exporter	ATCC29328_16270	ATCC53516_12160	07T609_15740	08T492_16760	09T408_15680	09T494_18540	12T272_16030	12T273_18480	12T306_16640	CCUG54800_17900	T151023_15200	T160124_15750
12	24	0.167	hypothetical protein	ATCC29328_16300,ATCC2932	ATCC53516_12090,ATCC5351	07T609_15820,07T609_	08T492_16840,08T492_16	09T408_15780,09T408_	09T494_00580,09T494_	12T272_16110,12T272_	12T273_18560,12T273_	12T306_16730,12T306_	CCUG54800_17930,CCUG548	T151023_15290,T151023_	T160124_15830,T160124_15780
12	12	1	Polyphosphate kinase 2 (PPK2)	ATCC29328_16310	ATCC53516_12120	07T609_15790	08T492_16810	09T408_15720	09T494_00530	12T272_16070	12T273_18520	12T306_16680	CCUG54800_17940	T151023_15240	T160124_15790
12	12	1	Limonene 1,2-monooxygenase	ATCC29328_16320	ATCC53516_12110	07T609_15800	08T492_16820	09T408_15730	09T494_00550	12T272_16080	12T273_18530	12T306_16690	CCUG54800_17950	T151023_15250	T160124_15800
12	12	1	Ribokinase	ATCC29328_16330	ATCC53516_12100	07T609_15810	08T492_16830	09T408_15760	09T494_00560	12T272_16090	12T273_18540	12T306_16700	CCUG54800_17980	T151023_15260	T160124_15810
8	8	1	Immunoglobulin G-binding protein A precurso	ATCC29328_16350	ATCC53516_12080	*	08T492_16850	*	*	12T272_16120	12T273_18580	12T306_16740	*	T151023_15300	T160124_15840
12	12	1	hypothetical protein	ATCC29328_16360	ATCC53516_12070	07T609_15830	08T492_16860	09T408_15790	09T494_00590	12T272_16130	12T273_18590	12T306_16750	CCUG54800_18010	T151023_15310	T160124_15850
12	12	1	N-acetylmuramoyl-L-alanine amidase LytC pre	ATCC29328_16370	ATCC53516_12060	07T609_15840	08T492_16870	09T408_15800	09T494_00600	12T272_16140	12T273_18600	12T306_16760	CCUG54800_18020	T151023_15320	T160124_15860
12	12	1	hypothetical protein	ATCC29328_16380	ATCC53516_12030	07T609_15850	08T492_16880	09T408_15810	09T494_00610	12T272_16170	12T273_18610	12T306_16790	CCUG54800_18030	T151023_15330	T160124_15870
10	10	1	putative ABC transporter ATP-binding protein	ATCC29328_16390	ATCC53516_12020	07T609_15860	*	09T408_15820	09T494_00620	12T272_16180	12T273_18620	12T306_16800	CCUG54800_18040	*	T160124_15880
10	10	1	hypothetical protein	ATCC29328_16400	ATCC53516_12010	07T609_15870	*	09T408_15830	09T494_00630	12T272_16190	12T273_18630	12T306_16810	CCUG54800_18050	*	T160124_15890
10	10	1	hypothetical protein	ATCC29328_16410	ATCC53516_12000	07T609_15880	*	09T408_15840	09T494_00640	12T272_16200	12T273_18640	12T306_16820	CCUG54800_18060	*	T160124_15900
12	14	0.129	hypothetical protein	ATCC29328_16420	ATCC53516_11990	07T609_15890,07T609_	08T492_16910	09T408_15870	09T494_00650	12T272_16210	12T273_18650	12T306_16830	CCUG54800_18090	T151023_15470,T151023_	T160124_15910
4	4	1	Oligopeptide-binding protein AppA precursor	ATCC29328_16430	ATCC53516_10930	*	*	*	*	*	12T273_18670	*	*	T151023_13910	*
12	12	1	Beta-lactamase superfamily domain protein	ATCC29328_16490	ATCC53516_11960	07T609_16130	08T492_16970	09T408_15940	09T494_00670	12T272_16230	12T273_18730	12T306_16850	CCUG54800_18160	T151023_15540	T160124_15970
12	12	1	Cys-tRNA(Pro)/Cys-tRNA(Cys) deacylase YbaK	ATCC29328_16500	ATCC53516_11950	07T609_16140	08T492_16980	09T408_15950	09T494_00680	12T272_16240	12T273_18740	12T306_16860	CCUG54800_18170	T151023_15550	T160124_15980
12	12	1	Glutamate--cysteine ligase GshA	ATCC29328_16510	ATCC53516_11940	07T609_16150	08T492_16990	09T408_15960	09T494_00690	12T272_16250	12T273_18750	12T306_16870	CCUG54800_18180	T151023_15560	T160124_15990
12	12	1	hypothetical protein	ATCC29328_16520	ATCC53516_11930	07T609_16160	08T492_17000	09T408_15970	09T494_00700	12T272_16260	12T273_18760	12T306_16880	CCUG54800_18190	T151023_15570	T160124_16000
9	9	1	Toxin RelG	ATCC29328_16530	*	07T609_16170	08T492_17010	*	*	12T272_16270	12T273_00010	12T306_00690	CCUG54800_00310	T151023_15580	T160124_16010
7	8	0.216	hypothetical protein	ATCC29328_16540,ATCC2932	*	07T609_16180	08T492_17020	*	*	12T272_16280	12T273_00020	*	*	T151023_15590	T160124_16020
2	2	1	hypothetical protein	ATCC29328_16550	*	07T609_16190	*	*	*	*	*	*	*	*	*
12	12	1	tRNA-specific adenosine deaminase	ATCC29328_16570	ATCC53516_11880	07T609_16210	08T492_17040	09T408_16020	09T494_00730	12T272_16320	12T273_00050	12T306_16910	CCUG54800_18220	T151023_15630	T160124_16080
12	12	1	2-oxoglutarate carboxylase small subunit	ATCC29328_16580	ATCC53516_11870	07T609_16220	08T492_17050	09T408_16030	09T494_00740	12T272_16330	12T273_00060	12T306_16920	CCUG54800_18230	T151023_15640	T160124_16090
8	8	1	hypothetical protein	ATCC29328_16590	ATCC53516_11860	*	08T492_17060	*	09T494_00750	12T272_16340	*	12T306_16930	CCUG54800_18250	T151023_15650	*
9	9	0.667	Antitoxin MazE	ATCC29328_16600	ATCC53516_11850	*	*	09T408_16080	09T494_00760	12T272_16350	12T273_00080	12T306_16940	CCUG54800_18260	T151023_15660	*
11	16	0.22	hypothetical protein	ATCC29328_16610	ATCC53516_11840	07T609_16240	08T492_17940	*	09T494_00770	12T272_18050,12T272_	12T273_01070,12T273_	12T306_16950	CCUG54800_18270,CCUG548	T151023_00640,T151023_	T160124_16900,T160124_16110
12	13	0.141	hypothetical protein	ATCC29328_16620	ATCC53516_11830	07T609_16250,07T609_	08T492_17070	09T408_16090	09T494_00780	12T272_16370	12T273_00110	12T306_16960	CCUG54800_18280	T151023_15680	T160124_16120
11	11	1	hypothetical protein	ATCC29328_16630	ATCC53516_11820	*	08T492_17080	09T408_16100	09T494_00790	12T272_16380	12T273_00120	12T306_16970	CCUG54800_18290	T151023_15690	T160124_16130
10	10	1	putative sulfite oxidase subunit YedZ	ATCC29328_16640	ATCC53516_11810	*	*	09T408_16110	09T494_00800	12T272_16390	12T273_00130	12T306_16980	CCUG54800_18300	T151023_15700	T160124_16140
9	9	1	Na(+)-translocating NADH-quinone reductase s	ATCC29328_16650	ATCC53516_11800	*	*	09T408_16120	09T494_00810	*	12T273_00140	12T306_16990	CCUG54800_18310	T151023_15710	T160124_16150
12	12	1	2-dehydropantoate 2-reductase	ATCC29328_16660	ATCC53516_11790	07T609_16290	08T492_17090	09T408_16130	09T494_00820	12T272_16420	12T273_00150	12T306_17000	CCUG54800_18320	T151023_15720	T160124_16160
12	12	1	Cobalt-dependent inorganic pyrophosphatase	ATCC29328_16670	ATCC53516_11780	07T609_16300	08T492_17100	09T408_16140	09T494_00830	12T272_16430	12T273_00160	12T306_17010	CCUG54800_18330	T151023_15730	T160124_16170
12	12	1	nicotinamidase/pyrazinamidase	ATCC29328_16680	ATCC53516_11770	07T609_16310	08T492_17110	09T408_16150	09T494_00840	12T272_16440	12T273_00170	12T306_17020	CCUG54800_18340	T151023_15740	T160124_16180
12	12	1	hypothetical protein	ATCC29328_16690	ATCC53516_11720	07T609_16330	08T492_17130	09T408_16170	09T494_00860	12T272_16450	12T273_00190	12T306_17060	CCUG54800_18360	T151023_15750	T160124_16200
12	12	1	hypothetical protein	ATCC29328_16700	ATCC53516_11710	07T609_16340	08T492_17140	09T408_16180	09T494_00870	12T272_16460	12T273_00200	12T306_17070	CCUG54800_18370	T151023_15770	T160124_16210
4	4	1	RelB antitoxin	ATCC29328_16710	ATCC53516_11690	*	*	*	09T494_00890	*	*	*	CCUG54800_18380	*	*
12	12	1	tRNA-specific 2-thiouridylase MnmA	ATCC29328_16720	ATCC53516_11680	07T609_16350	08T492_17150	09T408_16190	09T494_00900	12T272_16470	12T273_00210	12T306_17080	CCUG54800_18390	T151023_15780	T160124_16220
12	12	1	CAAX amino terminal protease self- immunity	ATCC29328_16740	ATCC53516_11660	07T609_16370	08T492_17170	09T408_16210	09T494_00920	12T272_16490	12T273_00230	12T306_17100	CCUG54800_18410	T151023_15800	T160124_16240
8	8	1	Macrolide export ATP-binding/permease prote	ATCC29328_16750	ATCC53516_11650	*	*	09T408_16220	09T494_00930	12T272_16500	*	12T306_17110	CCUG54800_18420	T151023_15810	*
8	8	1	hypothetical protein	ATCC29328_16760	ATCC53516_11640	*	*	09T408_16230	09T494_00940	12T272_16510	*	12T306_17120	CCUG54800_18430	T151023_15820	*
8	8	1	hypothetical protein	ATCC29328_16770	ATCC53516_11630	*	*	09T408_16240	09T494_00950	12T272_16520	*	12T306_17130	CCUG54800_18440	T151023_15830	*
8	8	1	hypothetical protein	ATCC29328_16780	ATCC53516_11620	*	*	09T408_16250	09T494_00960	12T272_16530	*	12T306_17140	CCUG54800_18450	T151023_15840	*
12	12	1	hypothetical protein	ATCC29328_16790	ATCC53516_11610	07T609_16380	08T492_17180	09T408_16260	09T494_00970	12T272_16540	12T273_00240	12T306_17150	CCUG54800_18460	T151023_15850	T160124_16250
12	12	1	G5 domain protein	ATCC29328_16820	ATCC53516_11580	07T609_16450	08T492_17250	09T408_16280	09T494_01010	12T272_16560	12T273_00270	12T306_17190	CCUG54800_18480	T151023_15880	T160124_16280
12	12	1	hypothetical protein	ATCC29328_16840	ATCC53516_11560	07T609_16470	08T492_17270	09T408_16300	09T494_01030	12T272_16580	12T273_00290	12T306_17210	CCUG54800_18500	T151023_00010	T160124_16300
12	12	1	hypothetical protein	ATCC29328_16850	ATCC53516_11550	07T609_16480	08T492_17280	09T408_16310	09T494_01040	12T272_16590	12T273_00300	12T306_17220	CCUG54800_18510	T151023_00020	T160124_16310
12	12	1	hypothetical protein	ATCC29328_16860	ATCC53516_11540	07T609_16490	08T492_17290	09T408_16320	09T494_01050	12T272_16600	12T273_00310	12T306_17230	CCUG54800_18520	T151023_00030	T160124_16320
12	12	1	hypothetical protein	ATCC29328_16870	ATCC53516_11530	07T609_16500	08T492_17300	09T408_16330	09T494_01060	12T272_16610	12T273_00320	12T306_17240	CCUG54800_18530	T151023_00040	T160124_16330
12	12	1	hypothetical protein	ATCC29328_16880	ATCC53516_11520	07T609_16510	08T492_17310	09T408_16340	09T494_01070	12T272_16620	12T273_00330	12T306_17250	CCUG54800_18540	T151023_00050	T160124_16340
12	12	1	hypothetical protein	ATCC29328_16890	ATCC53516_11510	07T609_16520	08T492_17320	09T408_16350	09T494_01080	12T272_16630	12T273_00340	12T306_17260	CCUG54800_18550	T151023_00060	T160124_16350
7	7	1	transcriptional regulator FixK	ATCC29328_16900	*	07T609_16530	*	09T408_16360	*	12T272_16640	12T273_00380	12T306_17270	*	T151023_00070	*
7	7	1	Sodium:neurotransmitter symporter family pr	ATCC29328_16910	*	07T609_16540	*	09T408_16370	*	12T272_16650	12T273_00390	12T306_17280	*	T151023_00080	*
7	7	1	Glycine reductase complex component B subu	ATCC29328_16930	*	07T609_16560	*	09T408_16390	*	12T272_16680	12T273_00410	12T306_17300	*	T151023_00100	*
7	7	1	hypothetical protein	ATCC29328_16950	*	07T609_16580	*	09T408_16410	*	12T272_16700	12T273_00430	12T306_17320	*	T151023_00120	*
12	12	1	tRNA 2-selenouridine synthase	ATCC29328_16970	ATCC53516_11490	07T609_16610	08T492_17350	09T408_16440	09T494_01120	12T272_16800	12T273_00540	12T306_17420	CCUG54800_18580	T151023_00160	T160124_16380
2	2	1	hypothetical protein	ATCC29328_16980	*	*	*	*	*	12T272_16810	*	*	*	*	*
12	12	1	hypothetical protein	ATCC29328_16990	ATCC53516_11480	07T609_16620	08T492_17360	09T408_16450	09T494_01130	12T272_16820	12T273_00550	12T306_17430	CCUG54800_18590	T151023_00170	T160124_16390
12	12	1	hypothetical protein	ATCC29328_17000	ATCC53516_11470	07T609_16630	08T492_17370	09T408_16460	09T494_01140	12T272_16830	12T273_00560	12T306_17440	CCUG54800_18600	T151023_00180	T160124_16400
12	12	1	Phosphate acetyltransferase	ATCC29328_17010	ATCC53516_11460	07T609_16640	08T492_17380	09T408_16470	09T494_01150	12T272_16840	12T273_00570	12T306_17450	CCUG54800_18610	T151023_00190	T160124_16410
12	12	1	hypothetical protein	ATCC29328_17020	ATCC53516_11450	07T609_16650	08T492_17390	09T408_16480	09T494_01160	12T272_16850	12T273_00580	12T306_17460	CCUG54800_18620	T151023_00200	T160124_16420
12	12	1	Methenyltetrahydrofolate cyclohydrolase	ATCC29328_17040	ATCC53516_11430	07T609_16670	08T492_17410	09T408_16500	09T494_01180	12T272_16870	12T273_00600	12T306_17480	CCUG54800_18640	T151023_00220	T160124_16440
12	12	1	Chromosome-partitioning protein Spo0J	ATCC29328_17050	ATCC53516_11420	07T609_16680	08T492_17420	09T408_16510	09T494_01190	12T272_16880	12T273_00610	12T306_17490	CCUG54800_18650	T151023_00230	T160124_16450
12	13	0.141	Sporulation initiation inhibitor protein Soj	ATCC29328_17060,ATCC2932	ATCC53516_11410	07T609_16690	08T492_17430	09T408_16520	09T494_01200	12T272_16890	12T273_00620	12T306_17500	CCUG54800_18660	T151023_00240	T160124_16460
12	12	1	Putative competence-damage inducible protei	ATCC29328_17070	ATCC53516_11400	07T609_16700	08T492_17440	09T408_16530	09T494_01210	12T272_16900	12T273_00630	12T306_17510	CCUG54800_18670	T151023_00250	T160124_16470
12	12	1	Ribosomal RNA small subunit methyltransfera	ATCC29328_17080	ATCC53516_11390	07T609_16710	08T492_17450	09T408_16540	09T494_01220	12T272_16910	12T273_00640	12T306_00010	CCUG54800_18680	T151023_00260	T160124_16480
12	12	1	tRNA uridine 5-carboxymethylaminomethyl m	ATCC29328_17090	ATCC53516_11380	07T609_16720	08T492_17460	09T408_16550	09T494_01230	12T272_16920	12T273_00650	12T306_00020	CCUG54800_18690	T151023_00270	T160124_16490
12	12	1	tRNA modification GTPase MnmE	ATCC29328_17100	ATCC53516_11370	07T609_16730	08T492_17470	09T408_16560	09T494_01240	12T272_16930	12T273_00660	12T306_00030	CCUG54800_18700	T151023_00280	T160124_16500
11	11	0.636	R3H domain protein	ATCC29328_17110	ATCC53516_11350	*	08T492_17480	09T408_16580	09T494_01250	12T272_17640	12T273_00670	12T306_00080	CCUG54800_18710	T151023_00300	T160124_16510
12	12	1	Membrane protein insertase YidC	ATCC29328_17120	ATCC53516_11340	07T609_16790	08T492_17490	09T408_16590	09T494_01260	12T272_17650	12T273_00680	12T306_00090	CCUG54800_18720	T151023_00310	T160124_16520
12	12	1	Ribonuclease P protein component	ATCC29328_17130	ATCC53516_11320	07T609_16800	08T492_17500	09T408_16600	09T494_01270	12T272_17660	12T273_00690	12T306_00110	CCUG54800_18740	T151023_00320	T160124_16530
12	12	1	50S ribosomal protein L34	ATCC29328_17140	ATCC53516_11310	07T609_16810	08T492_17510	09T408_16610	09T494_01280	12T272_17670	12T273_00700	12T306_00120	CCUG54800_18750	T151023_00330	T160124_16540
1	1	1	hypothetical protein	ATCC29328_17170	*	*	*	*	*	*	*	*	*	*	*
1	1	1	Relaxase/Mobilisation nuclease domain protei	ATCC29328_17180	*	*	*	*	*	*	*	*	*	*	*
4	4	1	Initiator Replication protein	ATCC29328_17190	*	07T609_17180	08T492_18280	*	09T494_02020	*	*	*	*	*	*
1	1	1	hypothetical protein	ATCC29328_17200	*	*	*	*	*	*	*	*	*	*	*
1	3	1	putative transposase	ATCC29328_17210,ATCC2932	*	*	*	*	*	*	*	*	*	*	*
1	2	1	Transposase IS200 like protein	ATCC29328_17220,ATCC2932	*	*	*	*	*	*	*	*	*	*	*
1	1	1	hypothetical protein	ATCC29328_17230	*	*	*	*	*	*	*	*	*	*	*
1	1	1	hypothetical protein	ATCC29328_17240	*	*	*	*	*	*	*	*	*	*	*
1	1	1	hypothetical protein	ATCC29328_17250	*	*	*	*	*	*	*	*	*	*	*
1	1	1	hypothetical protein	ATCC29328_17260	*	*	*	*	*	*	*	*	*	*	*
1	2	1	hypothetical protein	ATCC29328_17270,ATCC2932	*	*	*	*	*	*	*	*	*	*	*
1	1	1	hypothetical protein	ATCC29328_17280	*	*	*	*	*	*	*	*	*	*	*
1	2	1	Single-stranded DNA-binding protein ssb	ATCC29328_17290,ATCC2932	*	*	*	*	*	*	*	*	*	*	*
1	1	1	hypothetical protein	ATCC29328_17300	*	*	*	*	*	*	*	*	*	*	*
1	1	1	hypothetical protein	ATCC29328_17310	*	*	*	*	*	*	*	*	*	*	*
1	1	1	hypothetical protein	ATCC29328_17320	*	*	*	*	*	*	*	*	*	*	*
1	1	1	hypothetical protein	ATCC29328_17330	*	*	*	*	*	*	*	*	*	*	*
1	1	1	DNA primase TraC	ATCC29328_17340	*	*	*	*	*	*	*	*	*	*	*
1	1	1	hypothetical protein	ATCC29328_17350	*	*	*	*	*	*	*	*	*	*	*
1	1	1	hypothetical protein	ATCC29328_17360	*	*	*	*	*	*	*	*	*	*	*
1	1	1	hypothetical protein	ATCC29328_17370	*	*	*	*	*	*	*	*	*	*	*
1	1	1	hypothetical protein	ATCC29328_17380	*	*	*	*	*	*	*	*	*	*	*
1	1	1	hypothetical protein	ATCC29328_17390	*	*	*	*	*	*	*	*	*	*	*
1	1	1	hypothetical protein	ATCC29328_17400	*	*	*	*	*	*	*	*	*	*	*
1	1	1	hypothetical protein	ATCC29328_17410	*	*	*	*	*	*	*	*	*	*	*
1	1	1	hypothetical protein	ATCC29328_17420	*	*	*	*	*	*	*	*	*	*	*
1	1	1	hypothetical protein	ATCC29328_17450	*	*	*	*	*	*	*	*	*	*	*
1	1	1	hypothetical protein	ATCC29328_17460	*	*	*	*	*	*	*	*	*	*	*
1	1	1	hypothetical protein	ATCC29328_17470	*	*	*	*	*	*	*	*	*	*	*
1	1	1	hypothetical protein	ATCC29328_17490	*	*	*	*	*	*	*	*	*	*	*
1	1	1	Putative transposase DNA-binding domain pro	ATCC29328_17500	*	*	*	*	*	*	*	*	*	*	*
1	1	1	hypothetical protein	ATCC29328_17510	*	*	*	*	*	*	*	*	*	*	*
1	2	1	hypothetical protein	ATCC29328_17520,ATCC2932	*	*	*	*	*	*	*	*	*	*	*
1	1	1	hypothetical protein	ATCC29328_17530	*	*	*	*	*	*	*	*	*	*	*
1	1	1	hypothetical protein	ATCC29328_17540	*	*	*	*	*	*	*	*	*	*	*
1	2	1	hypothetical protein	ATCC29328_17550,ATCC2932	*	*	*	*	*	*	*	*	*	*	*
1	1	1	hypothetical protein	ATCC29328_17570	*	*	*	*	*	*	*	*	*	*	*
1	1	1	hypothetical protein	ATCC29328_17580	*	*	*	*	*	*	*	*	*	*	*
1	1	1	hypothetical protein	ATCC29328_17600	*	*	*	*	*	*	*	*	*	*	*
1	1	1	hypothetical protein	ATCC29328_17610	*	*	*	*	*	*	*	*	*	*	*
1	1	1	hypothetical protein	ATCC29328_17620	*	*	*	*	*	*	*	*	*	*	*
2	3	1	hypothetical protein	ATCC29328_17630,ATCC2932	*	*	*	*	*	*	12T273_00350	*	*	*	*
1	1	1	CAAX amino terminal protease self- immunity	ATCC29328_17640	*	*	*	*	*	*	*	*	*	*	*
2	3	1	hypothetical protein	ATCC29328_17660,ATCC2932	*	*	*	*	*	*	*	*	CCUG54800_01430	*	*
1	2	1	hypothetical protein	ATCC29328_17670,ATCC2932	*	*	*	*	*	*	*	*	*	*	*
1	1	1	hypothetical protein	ATCC29328_17700	*	*	*	*	*	*	*	*	*	*	*
1	1	1	hypothetical protein	ATCC29328_17710	*	*	*	*	*	*	*	*	*	*	*
1	1	1	hypothetical protein	ATCC29328_17720	*	*	*	*	*	*	*	*	*	*	*
1	1	1	hypothetical protein	ATCC29328_17730	*	*	*	*	*	*	*	*	*	*	*
1	1	1	hypothetical protein	ATCC29328_17740	*	*	*	*	*	*	*	*	*	*	*
1	1	1	hypothetical protein	ATCC29328_17750	*	*	*	*	*	*	*	*	*	*	*
2	2	1	CRISPR-associated endoribonuclease Cse3	ATCC29328_17760	*	*	*	09T408_02900	*	*	*	*	*	*	*
2	2	1	CRISPR system Cascade subunit CasD	ATCC29328_17770	*	*	*	09T408_02890	*	*	*	*	*	*	*
2	2	1	CRISPR system Cascade subunit CasC	ATCC29328_17780	*	*	*	09T408_02880	*	*	*	*	*	*	*
2	2	1	CRISPR-associated protein Cse1 (CRISPR_cse1)	ATCC29328_17790	*	*	*	09T408_02860	*	*	*	*	*	*	*
1	1	1	Sortase family protein	ATCC29328_17800	*	*	*	*	*	*	*	*	*	*	*
1	1	1	hypothetical protein	ATCC29328_17810	*	*	*	*	*	*	*	*	*	*	*
1	1	1	hypothetical protein	ATCC29328_17820	*	*	*	*	*	*	*	*	*	*	*
1	1	1	Single-stranded DNA-binding protein ssb	ATCC29328_17830	*	*	*	*	*	*	*	*	*	*	*
1	1	1	hypothetical protein	ATCC29328_17840	*	*	*	*	*	*	*	*	*	*	*
1	1	1	hypothetical protein	ATCC29328_17850	*	*	*	*	*	*	*	*	*	*	*
1	1	1	hypothetical protein	ATCC29328_17860	*	*	*	*	*	*	*	*	*	*	*
1	1	1	hypothetical protein	ATCC29328_17870	*	*	*	*	*	*	*	*	*	*	*
1	1	1	hypothetical protein	ATCC29328_17880	*	*	*	*	*	*	*	*	*	*	*
1	1	1	hypothetical protein	ATCC29328_17890	*	*	*	*	*	*	*	*	*	*	*
1	1	1	hypothetical protein	ATCC29328_17900	*	*	*	*	*	*	*	*	*	*	*
4	6	0.221	Modification methylase HaeIII	ATCC29328_17910,ATCC2932	*	07T609_02830	*	*	*	*	*	*	CCUG54800_14940,CCUG548	*	T160124_14050
1	1	1	hypothetical protein	ATCC29328_17920	*	*	*	*	*	*	*	*	*	*	*
1	1	1	hypothetical protein	ATCC29328_17940	*	*	*	*	*	*	*	*	*	*	*
1	1	1	hypothetical protein	ATCC29328_17950	*	*	*	*	*	*	*	*	*	*	*
1	1	1	hypothetical protein	ATCC29328_17960	*	*	*	*	*	*	*	*	*	*	*
1	1	1	hypothetical protein	ATCC29328_17970	*	*	*	*	*	*	*	*	*	*	*
1	1	1	hypothetical protein	ATCC29328_17980	*	*	*	*	*	*	*	*	*	*	*
1	1	1	hypothetical protein	ATCC29328_17990	*	*	*	*	*	*	*	*	*	*	*
1	1	1	hypothetical protein	ATCC29328_18000	*	*	*	*	*	*	*	*	*	*	*
2	2	1	Siphovirus Gp157	ATCC29328_18010	*	*	*	*	09T494_00320	*	*	*	*	*	*
1	1	1	hypothetical protein	ATCC29328_18020	*	*	*	*	*	*	*	*	*	*	*
1	1	1	hypothetical protein	ATCC29328_18030	*	*	*	*	*	*	*	*	*	*	*
1	1	1	hypothetical protein	ATCC29328_18040	*	*	*	*	*	*	*	*	*	*	*
1	1	1	hypothetical protein	ATCC29328_18050	*	*	*	*	*	*	*	*	*	*	*
1	1	1	hypothetical protein	ATCC29328_18060	*	*	*	*	*	*	*	*	*	*	*
10	18	0.158	Transposase IS200 like protein	ATCC29328_18070	ATCC53516_11740,ATCC5351	07T609_01840,07T609_	08T492_18400,08T492_18	*	09T494_18710,09T494_	12T272_18230	12T273_00370	*	CCUG54800_01620	T151023_09130	T160124_03020
1	1	1	hypothetical protein	ATCC29328_18090	*	*	*	*	*	*	*	*	*	*	*
1	1	1	hypothetical protein	ATCC29328_18100	*	*	*	*	*	*	*	*	*	*	*
1	1	1	hypothetical protein	ATCC29328_18110	*	*	*	*	*	*	*	*	*	*	*
1	1	1	hypothetical protein	ATCC29328_18120	*	*	*	*	*	*	*	*	*	*	*
1	1	1	hypothetical protein	ATCC29328_18130	*	*	*	*	*	*	*	*	*	*	*
1	1	1	hypothetical protein	ATCC29328_18140	*	*	*	*	*	*	*	*	*	*	*
1	1	1	hypothetical protein	ATCC29328_18150	*	*	*	*	*	*	*	*	*	*	*
1	1	1	hypothetical protein	ATCC29328_18160	*	*	*	*	*	*	*	*	*	*	*
1	1	1	Helix-turn-helix domain protein	ATCC29328_18170	*	*	*	*	*	*	*	*	*	*	*
1	1	1	hypothetical protein	ATCC29328_18190	*	*	*	*	*	*	*	*	*	*	*
1	1	1	RNA polymerase sigma-D factor	ATCC29328_18200	*	*	*	*	*	*	*	*	*	*	*
1	1	1	SpoVT / AbrB like domain protein	ATCC29328_18210	*	*	*	*	*	*	*	*	*	*	*
1	2	1	putative transposase	ATCC29328_18240,ATCC2932	*	*	*	*	*	*	*	*	*	*	*
1	1	1	hypothetical protein	ATCC29328_18250	*	*	*	*	*	*	*	*	*	*	*
1	1	1	hypothetical protein	ATCC29328_18260	*	*	*	*	*	*	*	*	*	*	*
1	1	1	hypothetical protein	ATCC29328_18270	*	*	*	*	*	*	*	*	*	*	*
1	1	1	hypothetical protein	ATCC29328_18280	*	*	*	*	*	*	*	*	*	*	*
1	1	1	hypothetical protein	ATCC29328_18290	*	*	*	*	*	*	*	*	*	*	*
1	1	1	hypothetical protein	ATCC29328_18300	*	*	*	*	*	*	*	*	*	*	*
1	1	1	hypothetical protein	ATCC29328_18310	*	*	*	*	*	*	*	*	*	*	*
1	1	1	hypothetical protein	ATCC29328_18320	*	*	*	*	*	*	*	*	*	*	*
1	1	1	hypothetical protein	ATCC29328_18340	*	*	*	*	*	*	*	*	*	*	*
1	1	1	hypothetical protein	ATCC29328_18360	*	*	*	*	*	*	*	*	*	*	*
1	1	1	hypothetical protein	ATCC29328_18370	*	*	*	*	*	*	*	*	*	*	*
1	1	1	hypothetical protein	ATCC29328_18380	*	*	*	*	*	*	*	*	*	*	*
1	1	1	Peptostreptococcal albumin-binding protein p	ATCC29328_18390	*	*	*	*	*	*	*	*	*	*	*
1	1	1	G5 domain protein	ATCC29328_18400	*	*	*	*	*	*	*	*	*	*	*
1	1	1	Sortase family protein	ATCC29328_18410	*	*	*	*	*	*	*	*	*	*	*
1	1	1	hypothetical protein	ATCC29328_18420	*	*	*	*	*	*	*	*	*	*	*
1	2	1	Sortase family protein	ATCC29328_18430,ATCC2932	*	*	*	*	*	*	*	*	*	*	*
1	2	1	Cna protein B-type domain protein	ATCC29328_18440,ATCC2932	*	*	*	*	*	*	*	*	*	*	*
1	1	1	Collagen adhesin precursor	ATCC29328_18470	*	*	*	*	*	*	*	*	*	*	*
1	1	1	Thermonuclease precursor	ATCC29328_18480	*	*	*	*	*	*	*	*	*	*	*
1	1	1	hypothetical protein	ATCC29328_18490	*	*	*	*	*	*	*	*	*	*	*
1	1	1	hypothetical protein	ATCC29328_18500	*	*	*	*	*	*	*	*	*	*	*
1	1	1	hypothetical protein	ATCC29328_18510	*	*	*	*	*	*	*	*	*	*	*
1	1	1	Signal peptidase I P	ATCC29328_18520	*	*	*	*	*	*	*	*	*	*	*
1	1	1	hypothetical protein	ATCC29328_18530	*	*	*	*	*	*	*	*	*	*	*
1	1	1	hypothetical protein	ATCC29328_18540	*	*	*	*	*	*	*	*	*	*	*
1	1	1	hypothetical protein	ATCC29328_18550	*	*	*	*	*	*	*	*	*	*	*
1	1	1	ESX-1 secretion system protein EccA1	ATCC29328_18570	*	*	*	*	*	*	*	*	*	*	*
1	1	1	hypothetical protein	ATCC29328_18580	*	*	*	*	*	*	*	*	*	*	*
1	1	1	hypothetical protein	ATCC29328_18590	*	*	*	*	*	*	*	*	*	*	*
1	1	1	hypothetical protein	ATCC29328_18600	*	*	*	*	*	*	*	*	*	*	*
4	4	1	putative transposase	ATCC29328_18610	*	*	08T492_16900	09T408_15860	*	*	*	*	CCUG54800_18080	*	*
1	1	1	hypothetical protein	ATCC29328_18620	*	*	*	*	*	*	*	*	*	*	*
1	1	1	NgoFVII restriction endonuclease	ATCC29328_18640	*	*	*	*	*	*	*	*	*	*	*
1	1	1	N-acetylmuramoyl-L-alanine amidase LytC pre	ATCC29328_18650	*	*	*	*	*	*	*	*	*	*	*
1	1	1	Serine-aspartate repeat-containing protein D p	ATCC29328_18660	*	*	*	*	*	*	*	*	*	*	*

1	1	1	hypothetical protein	ATCC29328_18670	*	*	*	*	*	*	*	*	*	*	*
1	1	1	Sortase family protein	ATCC29328_18680	*	*	*	*	*	*	*	*	*	*	*
1	1	1	Cna protein B-type domain protein	ATCC29328_18690	*	*	*	*	*	*	*	*	*	*	*
1	1	1	Sortase family protein	ATCC29328_18700	*	*	*	*	*	*	*	*	*	*	*
1	1	1	hypothetical protein	ATCC29328_18710	*	*	*	*	*	*	*	*	*	*	*
1	1	1	hypothetical protein	ATCC29328_18720	*	*	*	*	*	*	*	*	*	*	*
1	1	1	hypothetical protein	ATCC29328_18740	*	*	*	*	*	*	*	*	*	*	*
1	1	1	hypothetical protein	ATCC29328_18750	*	*	*	*	*	*	*	*	*	*	*
7	13	1	Integrase core domain protein	ATCC29328_18760	ATCC53516_11910,ATCC5351	*	08T492_14160	*	*	*	12T273_10740,12T273_	*	CCUG54800_05880	T151023_15900	T160124_17010
7	12	1	Transposase	ATCC29328_18770	ATCC53516_11920,ATCC5351	*	08T492_14150	*	*	*	12T273_18780,12T273_	*	CCUG54800_05890	T151023_15910	T160124_17020
6	7	0.241	Nuclease-related domain protein	ATCC29328_18780,ATCC2932	ATCC53516_11760	07T609_16320	08T492_17120	*	09T494_00850	*	*	*	CCUG54800_18350	*	*
1	1	1	hypothetical protein	ATCC29328_18790	*	*	*	*	*	*	*	*	*	*	*
1	1	1	hypothetical protein	ATCC29328_18800	*	*	*	*	*	*	*	*	*	*	*
1	1	1	Restriction endonuclease	ATCC29328_18820	*	*	*	*	*	*	*	*	*	*	*
1	1	1	hypothetical protein	ATCC29328_18830	*	*	*	*	*	*	*	*	*	*	*
1	1	1	hypothetical protein	ATCC29328_18840	*	*	*	*	*	*	*	*	*	*	*
1	1	1	hypothetical protein	ATCC29328_18850	*	*	*	*	*	*	*	*	*	*	*
1	1	1	hypothetical protein	ATCC29328_18860	*	*	*	*	*	*	*	*	*	*	*
1	1	1	hypothetical protein	ATCC29328_18870	*	*	*	*	*	*	*	*	*	*	*
1	1	1	hypothetical protein	ATCC29328_18890	*	*	*	*	*	*	*	*	*	*	*
1	1	1	HNH endonuclease	ATCC29328_18900	*	*	*	*	*	*	*	*	*	*	*
1	1	1	hypothetical protein	ATCC29328_18910	*	*	*	*	*	*	*	*	*	*	*
1	1	1	hypothetical protein	ATCC29328_18940	*	*	*	*	*	*	*	*	*	*	*
1	1	1	hypothetical protein	ATCC29328_18950	*	*	*	*	*	*	*	*	*	*	*
10	10	1	hypothetical protein	ATCC29328_18960	ATCC53516_09970	07T609_00990	08T492_00520	09T408_00900	09T494_02670	*	12T273_02030	*	CCUG54800_01690	T151023_01590	T160124_00760
1	1	1	N-acetylmuramoyl-L-alanine amidase domain-	ATCC29328_18970	*	*	*	*	*	*	*	*	*	*	*
1	1	1	PrgI family protein	ATCC29328_18990	*	*	*	*	*	*	*	*	*	*	*
1	1	1	hypothetical protein	ATCC29328_19000	*	*	*	*	*	*	*	*	*	*	*
1	1	1	hypothetical protein	ATCC29328_19010	*	*	*	*	*	*	*	*	*	*	*
1	1	1	hypothetical protein	ATCC29328_19020	*	*	*	*	*	*	*	*	*	*	*
1	1	1	hypothetical protein	ATCC29328_19030	*	*	*	*	*	*	*	*	*	*	*
9	21	0.157	Transposase, Mutator family	*	ATCC53516_00090,ATCC5351	*	08T492_02810	09T408_03240	09T494_03890	12T272_01620	12T273_06650	12T306_17540	CCUG54800_00410,CCUG548	*	T160124_03170
1	1	1	Extracellular matrix-binding protein ebh precu	*	ATCC53516_00100	*	*	*	*	*	*	*	*	*	*
1	1	1	Immunoglobulin G-binding protein A precurso	*	ATCC53516_00110	*	*	*	*	*	*	*	*	*	*
1	1	1	hypothetical protein	*	ATCC53516_00120	*	*	*	*	*	*	*	*	*	*
1	1	1	G5 domain protein	*	ATCC53516_00130	*	*	*	*	*	*	*	*	*	*
1	1	1	hypothetical protein	*	ATCC53516_00230	*	*	*	*	*	*	*	*	*	*
4	4	1	Flavoredoxin	*	ATCC53516_00240	*	*	*	09T494_16650	12T272_14060	*	*	CCUG54800_15130	*	*
8	8	1	hypothetical protein	*	ATCC53516_00270	*	*	*	09T494_16680	12T272_14090	12T273_17270	12T306_15290	CCUG54800_15150	T151023_14240	T160124_13030
8	10	0.172	hypothetical protein	*	ATCC53516_00330	07T609_16040	08T492_14790,08T492_09	*	09T494_16270	12T272_14150	12T273_13140,12T273_	*	CCUG54800_10810	*	T160124_13090
6	6	1	hypothetical protein	*	ATCC53516_00340	07T609_16030	08T492_09460	*	09T494_16260	*	12T273_13130	*	CCUG54800_10800	*	*
5	5	1	Peroxyureidoacrylate/ureidoacrylate amidohy	*	ATCC53516_00350	*	08T492_14800	*	*	12T272_14160	12T273_17340	*	*	*	T160124_13100
8	8	1	LemA family protein	*	ATCC53516_00360	*	*	*	09T494_16740	12T272_14180	12T273_17350	12T306_15370	CCUG54800_15220	T151023_14300	T160124_13110
8	8	1	hypothetical protein	*	ATCC53516_00370	*	*	*	09T494_16750	12T272_14190	12T273_17370	12T306_15380	CCUG54800_15230	T151023_14310	T160124_13120
3	3	1	hypothetical protein	*	ATCC53516_00380	*	*	*	09T494_16760	12T272_14200	*	*	*	*	*
3	3	1	Aminoglycoside N(6')-acetyltransferase type 1	*	ATCC53516_00390	*	*	*	*	12T272_14210	*	*	*	*	T160124_13160
9	13	0.324	Aliphatic sulfonates import ATP-binding protei	*	ATCC53516_00440	07T609_00750,07T609_	*	09T408_13220	09T494_02420,09T494_	12T272_00550,12T272	12T273_01830	*	CCUG54800_01490	T151023_15420,T151023_	T160124_13200
9	11	0.495	Putative aliphatic sulfonates transport permea	*	ATCC53516_00450	07T609_00770	*	09T408_13230	09T494_02450,09T494_	12T272_00570,12T272	12T273_01850	*	CCUG54800_01510	T151023_01390	T160124_13210
5	5	1	NMT1/THI5 like protein	*	ATCC53516_00460	*	*	09T408_13240	09T494_16840	12T272_14270	*	*	*	*	T160124_13220
6	6	1	Pyrroline-5-carboxylate reductase	*	ATCC53516_00560	*	*	*	09T494_16960	12T272_14390	*	12T306_15520	CCUG54800_15380	T151023_14450	*
1	1	1	NADPH dehydrogenase	*	ATCC53516_00580	*	*	*	*	*	*	*	*	*	*
1	1	1	NADH oxidase	*	ATCC53516_00590	*	*	*	*	*	*	*	*	*	*
1	1	1	hypothetical protein	*	ATCC53516_00600	*	*	*	*	*	*	*	*	*	*
3	3	1	putative Mg(2+) transport ATPase	*	ATCC53516_00660	*	*	*	*	*	*	12T306_15650	CCUG54800_15490	*	*
2	2	1	Type-1 restriction enzyme EcoKI specificity pro	*	ATCC53516_00670	*	*	09T408_13430	*	*	*	*	*	*	*
2	2	1	putative type I restriction enzymeP M protein	*	ATCC53516_00680	*	*	09T408_13440	*	*	*	*	*	*	*
2	2	1	Type-1 restriction enzyme R protein	*	ATCC53516_00690	*	*	09T408_13450	*	*	*	*	*	*	*
1	1	1	hypothetical protein	*	ATCC53516_00700	*	*	*	*	*	*	*	*	*	*
1	1	1	Integrase core domain protein	*	ATCC53516_00750	*	*	*	*	*	*	*	*	*	*
1	1	1	hypothetical protein	*	ATCC53516_00760	*	*	*	*	*	*	*	*	*	*
5	8	0.211	Energy-coupling factor transporter transmemb	*	ATCC53516_00810,ATCC5351	07T609_14210	*	*	*	*	*	12T306_15850	CCUG54800_15610,CCUG548	T151023_02820	*
7	17	0.141	Transposase	*	ATCC53516_00870,ATCC5351	*	08T492_03280	09T408_17020,09T408_	*	12T272_12210	12T273_18850,12T273_	12T306_17550	*	*	T160124_17080,T160124_17090,T160124_17050
4	4	1	hypothetical protein	*	ATCC53516_00900	07T609_14170	*	*	*	*	*	12T306_15810	CCUG54800_15810	*	*
6	8	0.168	Putative HMP/thiamine import ATP-binding pr	*	ATCC53516_00930	*	*	*	09T494_13740	12T272_12880	12T273_13410	12T306_15840,12T306_	*	T151023_02970,T151023_	*
5	6	0.414	hypothetical protein	*	ATCC53516_00950	07T609_14220	*	*	*	*	*	12T306_15860	CCUG54800_15620,CCUG548	T151023_02810	*
5	7	0.209	Virulence regulon transcriptional activator VirF	*	ATCC53516_00960	07T609_14230,07T609_	*	*	*	*	*	12T306_15870	CCUG54800_15630,CCUG548	T151023_02800	*
1	2	1	hypothetical protein	*	ATCC53516_00980,ATCC5351	*	*	*	*	*	*	*	*	*	*
1	1	1	Caspase domain protein	*	ATCC53516_00990	*	*	*	*	*	*	*	*	*	*
1	1	1	hypothetical protein	*	ATCC53516_01000	*	*	*	*	*	*	*	*	*	*
8	12	0.131	hypothetical protein	*	ATCC53516_01010	07T609_16410,07T609_	08T492_17210,08T492_15	*	09T494_00990	*	12T273_03940	12T306_17170,12T306_	CCUG54800_15940	T151023_02750	*
2	2	1	CAAX amino terminal protease self- immunity	*	ATCC53516_01020	*	*	*	*	*	*	12T306_02740	*	*	*
5	5	1	hypothetical protein	*	ATCC53516_01030	*	08T492_15280	09T408_13680	*	*	12T273_03920	12T306_02730	*	*	*
1	1	1	hypothetical protein	*	ATCC53516_01050	*	*	*	*	*	*	*	*	*	*
2	2	1	hypothetical protein	*	ATCC53516_01060	*	*	*	*	*	*	*	*	T151023_02890	*
4	4	1	hypothetical protein	*	ATCC53516_01070	*	08T492_09270	*	*	*	12T273_03880	*	*	*	T160124_13830
3	7	1	Integrase core domain protein	*	ATCC53516_01080,ATCC5351	07T609_17260	08T492_08620	*	*	*	*	*	*	*	*
5	5	1	Putative multidrug export ATP-binding/permea	*	ATCC53516_01100	*	*	09T408_14110	*	*	12T273_03860	*	CCUG54800_14330	*	T160124_13850
3	3	1	Iron import ATP-binding/permease protein Irt	*	ATCC53516_01110	*	*	*	*	*	12T273_03850	*	CCUG54800_14340	*	*
6	8	0.157	HTH-type transcriptional regulator AcrR	*	ATCC53516_01150	*	08T492_15370	*	*	*	12T273_12990,12T273_	12T306_11140	CCUG54800_14380,CCUG548	*	T160124_13900
11	15	0.126	Transposon gamma-delta resolvase	*	ATCC53516_01160,ATCC5351	07T609_14380	08T492_15380	09T408_13760	09T494_15790	12T272_12840	12T273_03780,12T273_	12T306_16050	CCUG54800_16050,CCUG548	T151023_02880	T160124_13910,T160124_13930
4	4	1	hypothetical protein	*	ATCC53516_01170	*	*	*	*	*	12T273_03790	*	CCUG54800_16040	*	T160124_13920
4	4	1	hypothetical protein	*	ATCC53516_01190	*	*	*	*	*	12T273_03770	*	CCUG54800_16060	*	T160124_13940
3	3	1	hypothetical protein	*	ATCC53516_01230	*	*	*	*	*	12T273_03730	*	CCUG54800_16100	*	*
3	3	1	Holin family protein	*	ATCC53516_01240	*	*	*	*	*	12T273_03720	*	CCUG54800_16110	*	*
3	3	1	hypothetical protein	*	ATCC53516_01250	*	*	*	*	*	12T273_03710	*	CCUG54800_16120	*	*
3	3	1	hypothetical protein	*	ATCC53516_01260	*	*	*	*	*	12T273_03700	*	CCUG54800_16130	*	*
3	3	1	hypothetical protein	*	ATCC53516_01270	*	*	*	*	*	12T273_03690	*	CCUG54800_16140	*	*
1	1	1	hypothetical protein	*	ATCC53516_01280	*	*	*	*	*	*	*	*	*	*
1	1	1	hypothetical protein	*	ATCC53516_01290	*	*	*	*	*	*	*	*	*	*
3	3	1	Phage-related minor tail protein	*	ATCC53516_01300	*	*	*	*	*	12T273_03680	*	CCUG54800_16150	*	*
1	1	1	hypothetical protein	*	ATCC53516_01310	*	*	*	*	*	*	*	*	*	*
2	2	1	hypothetical protein	*	ATCC53516_01320	*	*	*	*	*	*	*	CCUG54800_16180	*	*
3	3	1	hypothetical protein	*	ATCC53516_01330	*	*	*	*	*	12T273_03650	*	CCUG54800_16190	*	*
3	3	1	hypothetical protein	*	ATCC53516_01340	*	*	*	*	*	12T273_03640	*	CCUG54800_16200	*	*
3	3	1	hypothetical protein	*	ATCC53516_01350	*	*	*	*	*	12T273_03620	*	CCUG54800_16220	*	*
3	3	1	hypothetical protein	*	ATCC53516_01360	*	*	*	*	*	12T273_03610	*	CCUG54800_16230	*	*
3	3	1	Phage head-tail joining protein	*	ATCC53516_01370	*	*	*	*	*	12T273_03600	*	CCUG54800_16240	*	*
3	3	1	Phage gp6-like head-tail connector protein	*	ATCC53516_01380	*	*	*	*	*	12T273_03590	*	CCUG54800_16250	*	*
3	3	1	Phage capsid family protein	*	ATCC53516_01390	*	*	*	*	*	12T273_03580	*	CCUG54800_16260	*	*
4	4	1	ATP-dependent Clp protease proteolytic subur	*	ATCC53516_01400	*	*	*	09T494_00100	*	12T273_03570	*	CCUG54800_16270	*	*
3	3	1	Phage portal protein	*	ATCC53516_01410	*	*	*	*	*	12T273_03560	*	CCUG54800_16280	*	*
9	11	0.273	hypothetical protein	*	ATCC53516_01420,ATCC5351	07T609_09820	08T492_14310	09T408_12620	09T494_14450	12T272_11430	12T273_03550,12T273_	12T306_14860	*	*	T160124_02600
3	3	1	Phage Terminase	*	ATCC53516_01430	*	*	*	*	*	12T273_03540	*	CCUG54800_16300	*	*
1	1	1	hypothetical protein	*	ATCC53516_01440	*	*	*	*	*	*	*	*	*	*
1	1	1	hypothetical protein	*	ATCC53516_01450	*	*	*	*	*	*	*	*	*	*
3	3	1	hypothetical protein	*	ATCC53516_01460	*	*	*	*	*	12T273_03510	*	CCUG54800_16330	*	*
3	4	0.36	Modification methylase DpnIIB	*	ATCC53516_01470	*	*	*	*	*	12T273_03480	*	CCUG54800_16350,CCUG548	*	*
3	3	1	S-adenosylmethionine synthetase	*	ATCC53516_01480	*	*	*	*	*	12T273_03470	*	CCUG54800_16360	*	*
1	1	1	NUMOD4 motif protein	*	ATCC53516_01490	*	*	*	*	*	*	*	*	*	*
3	3	1	Phage terminase, small subunit	*	ATCC53516_01500	*	*	*	*	*	12T273_03460	*	CCUG54800_16370	*	*
3	3	1	HNH endonuclease	*	ATCC53516_01510	*	*	*	*	*	12T273_03450	*	CCUG54800_16380	*	*
9	10	0.274	SpoVT / AbrB like domain protein	*	ATCC53516_01520,ATCC5351	07T609_00190	08T492_18120	09T408_00190	*	12T272_00060	12T273_01300	*	CCUG54800_16390	T151023_00850	T160124_00110
4	4	1	hypothetical protein	*	ATCC53516_01530	*	*	*	*	12T272_00050	12T273_01290	*	CCUG54800_16400	*	*
3	3	1	hypothetical protein	*	ATCC53516_01540	*	*	*	*	*	12T273_03440	*	CCUG54800_16410	*	*
3	3	1	ATP-dependent helicase HepA	*	ATCC53516_01550	*	*	*	*	*	12T273_03430	*	CCUG54800_16420	*	*
3	3	1	VRR-NUC domain protein	*	ATCC53516_01560	*	*	*	*	*	12T273_03420	*	CCUG54800_16430	*	*
3	3	1	hypothetical protein	*	ATCC53516_01570	*	*	*	*	*	12T273_03410	*	CCUG54800_16440	*	*
3	3	1	hypothetical protein	*	ATCC53516_01580	*	*	*	*	*	12T273_03400	*	CCUG54800_16450	*	*
3	3	1	hypothetical protein	*	ATCC53516_01590	*	*	*	*	*	12T273_03390	*	CCUG54800_16460	*	*
3	3	1	hypothetical protein	*	ATCC53516_01600	*	*	*	*	*	12T273_03380	*	CCUG54800_16470	*	*
1	1	1	DNA polymerase I, thermostable	*	ATCC53516_01610	*	*	*	*	*	*	*	*	*	*
3	3	1	hypothetical protein	*	ATCC53516_01620	*	*	*	*	*	12T273_03360	*	CCUG54800_16490	*	*
3	3	1	hypothetical protein	*	ATCC53516_01630	*	*	*	*	*	12T273_03350	*	CCUG54800_16500	*	*
3	3	1	hypothetical protein	*	ATCC53516_01640	*	*	*	*	*	12T273_03340	*	CCUG54800_16510	*	*
3	3	1	hypothetical protein	*	ATCC53516_01650	*	*	*	*	*	12T273_03330	*	CCUG54800_16520	*	*
1	1	1	hypothetical protein	*	ATCC53516_01660	*	*	*	*	*	*	*	*	*	*
2	2	1	flagellar biosynthesis sigma factor	*	ATCC53516_01670	*	*	*	*	*	*	*	CCUG54800_16540	*	*
2	2	1	hypothetical protein	*	ATCC53516_01680	*	*	*	*	*	*	*	CCUG54800_16550	*	*
2	2	1	hypothetical protein	*	ATCC53516_01690	*	*	*	*	*	*	*	CCUG54800_16560	*	*
3	3	1	Helix-turn-helix domain protein	*	ATCC53516_01700	*	*	*	09T494_15680	*	*	*	CCUG54800_16570	*	*
1	1	1	EcoKI restriction-modification system protein H	*	ATCC53516_01860	*	*	*	*	*	*	*	*	*	*
3	5	0.2	hypothetical protein	*	ATCC53516_02180,ATCC5351	*	*	*	*	*	*	12T306_08960	*	T151023_03790	*
3	5	0.26	Transposase IS116/IS110/IS902 family protein	*	ATCC53516_02190,ATCC5351	*	*	*	*	*	*	12T306_08970	*	T151023_03800	*
7	7	1	Phd_YefM	*	ATCC53516_02200	07T609_01710	08T492_01360	09T408_01670	*	*	*	12T306_02240	*	T151023_02320	T160124_01500
10	10	1	Haemolysin-III related	*	ATCC53516_02250	07T609_01660	08T492_01310	09T408_01620	09T494_03470	12T272_13420	12T273_02810	12T306_02200	*	T151023_02260	T160124_01470
4	4	1	hypothetical protein	*	ATCC53516_02260	*	*	*	09T494_03460	12T272_13430	*	*	CCUG54800_02520	*	*
7	7	1	hypothetical protein	*	ATCC53516_02320	*	08T492_01250	*	09T494_03400	12T272_13490	*	*	CCUG54800_02460	T151023_02200	T160124_01410
10	10	0.153	Serine-aspartate repeat-containing protein E p	*	ATCC53516_02330	07T609_01600	*	09T408_01560	09T494_03390	12T272_13500	12T273_02750	12T306_02140	CCUG54800_02450	T151023_02190	T160124_01400
8	8	1	hypothetical protein	*	ATCC53516_02870	07T609_11480	08T492_11400	09T408_10980	*	*	12T273_15080	12T306_13080	*	T151023_10980	T160124_11100
6	8	0.216	Transposase IS116/IS110/IS902 family protein	*	ATCC53516_03020,ATCC5351	07T609_17240	08T492_18360	*	09T494_00540	*	*	*	*	T151023_15970,T151023_	T160124_04850
3	3	1	Chaperone protein DnaJ	*	ATCC53516_03300	*	*	*	09T494_12520	*	*	*	CCUG54800_13100	*	*
4	4	1	hypothetical protein	*	ATCC53516_03450	*	*	*	09T494_12660	*	*	12T306_13650	CCUG54800_13270	*	*
10	12	0.21	hypothetical protein	*	ATCC53516_03460	*	08T492_12130	09T408_11510	09T494_12690	12T272_10190,12T272	12T273_15640	12T306_13660	CCUG54800_13320	T151023_11570	T160124_11840,T160124_11860
8	9	0.195	Putative 2-aminoethylphosphonate transport s	*	ATCC53516_04180	*	*	*	09T494_13400	12T272_10900,12T272	12T273_16370	12T306_14360	CCUG54800_14060	T151023_12250	T160124_12550
8	8	1	Phosphoglycerate transport regulatory protein	*	ATCC53516_04190	*	*	*	09T494_13410	12T272_10910	12T273_16380	12T306_14370	CCUG54800_14070	T151023_12260	T160124_12560
1	1	1	ABC transporter ATP-binding protein uup	*	ATCC53516_04280	*	*	*	*	*	*	*	*	*	*
3	3	1	hypothetical protein	*	ATCC53516_04290	*	08T492_11790	*	*	*	*	*	*	*	T160124_11660
3	3	1	hypothetical protein	*	ATCC53516_04300	*	08T492_11800	*	*	*	*	*	*	*	T160124_11650
3	3	1	DNA translocase FtsK	*	ATCC53516_04310	*	08T492_11810	*	*	*	*	*	*	*	T160124_11640
3	3	1	Replication initiation factor	*	ATCC53516_04320	*	08T492_11820	*	*	*	*	*	*	*	T160124_11630
3	3	1	hypothetical protein	*	ATCC53516_04330	*	08T492_11830	*	*	*	*	*	*	*	T160124_11620
3	3	1	Antirestriction protein (ArdA)	*	ATCC53516_04340	*	08T492_11840	*	*	*	*	*	*	*	T160124_11610
3	3	1	AAA-like domain protein	*	ATCC53516_04350	*	08T492_11850	*	*	*	*	*	*	*	T160124_11600
3	3	1	hypothetical protein	*	ATCC53516_04360	*	08T492_11860	*	*	*	*	*	*	*	T160124_11590
3	3	1	putative endopeptidase p60 precursor	*	ATCC53516_04370	*	08T492_11870	*	*	*	*	*	*	*	T160124_11580
3	3	1	Conjugative transposon protein TcpC	*	ATCC53516_04380	*	08T492_11880	*	*	*	*	*	*	*	T160124_11570
3	3	1	Tetracycline resistance protein TetM from tran	*	ATCC53516_04390	*	08T492_11890	*	*	*	*	*	*	*	T160124_11560
3	3	1	anaerobic benzoate catabolism transcriptional	*	ATCC53516_04400	*	08T492_11900	*	*	*	*	*	*	*	T160124_11550
3	3	1	RNA polymerase sigma factor	*	ATCC53516_04410	*	08T492_11910	*	*	*	*	*	*	*	T160124_11540
3	3	1	Helix-turn-helix domain protein	*	ATCC53516_04420	*	08T492_11920	*	*	*	*	*	*	*	T160124_11530
3	3	1	Excisionase from transposon Tn916	*	ATCC53516_04430	*	08T492_11930	*	*	*	*	*	*	*	T160124_11520
1	1	1	hypothetical protein	*	ATCC53516_04590	*	*	*	*	*	*	*	*	*	*
1	1	1	Abi-like protein	*	ATCC53516_04780	*	*	*	*	*	*	*	*	*	*
1	1	1	Abi-like protein	*	ATCC53516_04790	*	*	*	*	*	*	*	*	*	*
4	4	0.5	hypothetical protein	*	ATCC53516_04820	*	08T492_14290	*	*	*	12T273_06080	*	*	*	T160124_02640
7	7	1	hypothetical protein	*	ATCC53516_04870	*	08T492_14320	*	09T494_14460	12T272_11440	12T273_06030	12T306_14870	*	*	T160124_02590
7	7	1	Glycerol dehydrogenase	*	ATCC53516_04920	*	*	*	09T494_14510	12T272_11490	12T273_05980	12T306_14920	CCUG54800_14820	*	T160124_02540
9	9	1	Serine acetyltransferase	*	ATCC53516_04960	*	*	09T408_12700	09T494_14530	12T272_11510	12T273_05900	12T306_14940	CCUG54800_14840	T151023_12770	T160124_02520
9	9	1	Cysteine synthase	*	ATCC53516_04970	*	*	09T408_12710	09T494_14540	12T272_11520	12T273_05890	12T306_14950	CCUG54800_14850	T151023_12780	T160124_02510
9	17	0.213	transcriptional activator FtrB	*	ATCC53516_04980,ATCC5351	*	*	09T408_12720	09T494_04710,09T494_	12T272_02500,12T272	12T273_07450,12T273_	12T306_05610,12T306_	CCUG54800_04850,CCUG548	T151023_04200,T151023_	T160124_02500,T160124_03910
7	9	0.299	hypothetical protein	*	ATCC53516_05140,ATCC5351	07T609_03120	08T492_02800	09T408_03230	09T494_15910,09T494_	*	*	*	CCUG54800_04030	*	T160124_03160
1	1	1	hypothetical protein	*	ATCC53516_05150	*	*	*	*	*	*	*	*	*	*
1	1	1	Type IV secretion-system coupling protein DN/	*	ATCC53516_05160	*	*	*	*	*	*	*	*	*	*
1	1	1	hypothetical protein	*	ATCC53516_05170	*	*	*	*	*	*	*	*	*	*
1	1	1	hypothetical protein	*	ATCC53516_05180	*	*	*	*	*	*	*	*	*	*
1	1	1	hypothetical protein	*	ATCC53516_05190	*	*	*	*	*	*	*	*	*	*
1	1	1	von Willebrand factor type A domain protein	*	ATCC53516_05200	*	*	*	*	*	*	*	*	*	*
1	1	1	Protein kinase domain protein	*	ATCC53516_05210	*	*	*	*	*	*	*	*	*	*
1	2	1	Internalin-A precursor	*	ATCC53516_05220,ATCC5351	*	*	*	*	*	*	*	*	*	*
2	2	1	SOCS box	*	ATCC53516_05240	*	*	*	09T494_18040	*	*	*	*	*	*
1	1	1	hypothetical protein	*	ATCC53516_05250	*	*	*	*	*	*	*	*	*	*
1	2	1	hypothetical protein	*	ATCC53516_05270,ATCC5351	*	*	*	*	*	*	*	*	*	*
2	2	1	hypothetical protein	*	ATCC53516_05650	*	*	*	*	*	*	*	*	*	T160124_00920
2	2	1	hypothetical protein	*	ATCC53516_05660	*	*	*	*	*	*	*	*	*	T160124_05090
7	8	0.216	Acetylene hydratase	*	ATCC53516_05690	07T609_04950	08T492_04640	*	09T494_05830	12T272_03630	*	12T306_06730,12T306_	*	T151023_05290	*
2	3	1	Transposase IS200 like protein	*	ATCC53516_05800,ATCC5351	*	*	*	*	*	*	*	*	T151023_16030	*
4	4	1	Thiosulfate sulfurtransferase GlpE	*	ATCC53516_05940	*	*	*	09T494_05590	12T272_03390	*	12T306_06500	*	*	*
4	8	0.5	CAMP factor (Cfa)	*	ATCC53516_05950,ATCC5351	*	*	*	09T494_05570,09T494_	12T272_03370,12T272	*	12T306_06490,12T306_	*	*	*
8	8	1	HTH-type transcriptional regulator Xre	*	ATCC53516_06510	*	*	09T408_04350	09T494_05050	12T272_02850	12T273_07790	*	CCUG54800_05200	T151023_04560	T160124_04250
6	6	1	hypothetical protein	*	ATCC53516_06580	*	*	*	09T494_04990	12T272_02790	*	12T306_05900	CCUG54800_05140	T151023_04500	*
9	9	1	Glycine reductase complex component B subu	*	ATCC53516_06700	*	08T492_03740	09T408_04190	09T494_04880	*	12T273_07620	12T306_05780	CCUG54800_05030	T151023_04380	T160124_04080
1	2	1	hypothetical protein	*	ATCC53516_06860,ATCC5351	*	*	*	*	*	*	*	*	*	*
7	12	0.48	Acryloyl-CoA reductase (NADH)	*	ATCC53516_07150,ATCC5351	*	*	*	09T494_04460,09T494_	12T272_02260,12T272	12T273_07210	12T306_05370,12T306_	CCUG54800_04610	T151023_03970,T151023_	*
5	5	1	hypothetical protein	*	ATCC53516_07220	*	*	*	09T494_04400	12T272_02200	*	12T306_05310	*	T151023_03900	*
1	1	1	hypothetical protein	*	ATCC53516_07710	*	*	*	*	*	*	*	*	*	*
3	4	0.36	hypothetical protein	*	ATCC53516_07720	*	*	*	09T494_03900	*	12T273_06660,12T273_	*	*	*	*
10	10	1	PII-type proteinase precursor	*	ATCC53516_07750	07T609_03110	08T492_02790	09T408_03220	09T494_03870	12T272_01600	12T273_06640	12T306_04830	CCUG54800_04020	*	T160124_03150
2	2	1	hypothetical protein	*	ATCC53516_07840	07T609_03010	*	*	*	*	*	*	*	*	*
2	2	1	hypothetical protein	*	ATCC53516_07950	*	*	09T408_02990	*	*	*	*	*	*	*
4	4	1	ABC-2 family transporter protein	*	ATCC53516_07970	*	08T492_13910	*	*	*	12T273_05800	*	*	*	T160124_02420
4	4	0.5	ABC-2 family transporter protein	*	ATCC53516_07980	*	08T492_13900	*	*	*	12T273_05790	*	*	*	T160124_02410
4	4	1	Sensory transduction protein regX3	*	ATCC53516_07990	*	08T492_13890	*	*	*	12T273_05780	*	*	*	T160124_02400
4	4	1	Sensor protein CzcS precursor	*	ATCC53516_08000	*	08T492_13880	*	*	*	12T273_05770	*	*	*	T160124_02390
6	6	1	hypothetical protein	*	ATCC53516_08180	*	*	*	*	*	12T273_05610	12T306_04180	CCUG54800_03560	T151023_13020	T160124_02230
1	1	1	Integrase core domain protein	*	ATCC53516_08340	*	*	*	*	*	*	*	*	*	*
1	1	1	Dipeptide transport system permease protein	*	ATCC53516_08470	*	*	*	*	*	*	*	*	*	*
3	3	1	hypothetical protein	*	ATCC53516_08630	*	*	*	*	*	*	*	CCUG54800_03140	*	T160124_01820
9	9	1	Na(+)/H(+) antiporter NhaC	*	ATCC53516_08920	*	*	09T408_02000	09T494_15470	12T272_12470	12T273_04940	12T306_03520	CCUG54800_02850	T151023_13710	T160124_01510
6	6	1	N-acetylmuramoyl-L-alanine amidase LytC pre	*	ATCC53516_08990	*	*	09T408_14600	*	*	*	12T306_03380	CCUG54800_16910	T151023_13780	T160124_14640
5	5	1	hypothetical protein	*	ATCC53516_09140	07T609_14950	08T492_15930	*	*	*	12T273_04670	*	*	T151023_13970	*

1	1		1	hypothetical protein	*	ATCC53516_09260	*	*	*	*	*	*	*	*	*	*
7	7		1	CRISPR-associated endoribonuclease Cas2	*	ATCC53516_09340	*	*	09T408_15000	09T494_17800	12T272_15320	*	12T306_03030	*	T151023_14640	T160124_15010
6	6		1	CRISPR-associated endonuclease Cas1	*	ATCC53516_09350	*	*	*	09T494_17810	12T272_15330	*	12T306_03020	*	T151023_14650	T160124_15020
6	6		1	hypothetical protein	*	ATCC53516_09360	*	*	*	09T494_17820	12T272_15340	*	12T306_03010	*	T151023_14660	T160124_15030
8	8		1	CRISPR-associated nuclease/helicase Cas3	*	ATCC53516_09370	*	08T492_16160	09T408_15010	09T494_17830	12T272_15350	*	12T306_03000	*	T151023_14670	T160124_15040
4	4		1	CRISPR-associated protein (Cas_Cas5)	*	ATCC53516_09380	*	08T492_16170	09T408_15020	09T494_17840	*	*	*	*	*	*
8	8		1	hypothetical protein	*	ATCC53516_09390	*	08T492_16180	09T408_15030	09T494_17850	12T272_15370	*	12T306_02980	*	T151023_14690	T160124_15060
4	4		1	CRISPR-associated protein Csx8 (Cas_Csx8)	*	ATCC53516_09400	*	08T492_16190	09T408_15040	09T494_17860	*	*	*	*	*	*
8	8		1	CRISPR associated protein Cas6	*	ATCC53516_09410	*	08T492_16200	09T408_15050	09T494_17870	12T272_15390	*	12T306_02960	*	T151023_14710	T160124_15080
9	9	0.667		hypothetical protein	*	ATCC53516_09890	*	*	09T408_00970	09T494_02740	12T272_00960	12T273_02110	12T306_01520	CCUG54800_01770	T151023_01660	T160124_00840
4	4		1	N-acetylmuramoyl-L-alanine amidase LytC pre	*	ATCC53516_09960	*	*	*	*	*	12T273_02040	*	CCUG54800_01700	*	T160124_00770
1	1		1	hypothetical protein	*	ATCC53516_10250	*	*	*	*	*	*	*	*	*	*
1	1		1	hypothetical protein	*	ATCC53516_10260	*	*	*	*	*	*	*	*	*	*
1	1		1	hypothetical protein	*	ATCC53516_10480	*	*	*	*	*	*	*	*	*	*
8	8		1	hypothetical protein	*	ATCC53516_10490	*	*	09T408_00350	09T494_02070	12T272_00200	12T273_01440	12T306_00900	CCUG54800_00530	T151023_01020	*
8	8		1	hypothetical protein	*	ATCC53516_10500	*	*	09T408_00340	09T494_02060	12T272_00190	12T273_01430	12T306_00890	CCUG54800_00520	T151023_01010	*
8	8		1	hypothetical protein	*	ATCC53516_10510	*	*	09T408_00330	09T494_02050	12T272_00180	12T273_01420	12T306_00880	CCUG54800_00510	T151023_01000	*
7	7		1	hypothetical protein	*	ATCC53516_10540	*	*	*	09T494_01890	12T272_00140	12T273_01370	12T306_00850	*	T151023_00950	T160124_00190
4	4		1	mRNA interferase YafQ	*	ATCC53516_10630	*	*	*	*	*	*	12T306_00760	CCUG54800_00380	T151023_00840	*
8	8		1	RelB antitoxin	*	ATCC53516_10640	07T609_00180	08T492_18110	09T408_00180	*	*	*	12T306_00750	CCUG54800_00370	T151023_00830	T160124_00100
1	1		1	hypothetical protein	*	ATCC53516_10720	*	*	*	*	*	*	*	*	*	*
1	1		1	hypothetical protein	*	ATCC53516_10830	*	*	*	*	*	*	*	*	*	*
1	1		1	hypothetical protein	*	ATCC53516_10840	*	*	*	*	*	*	*	*	*	*
8	8		1	hypothetical protein	*	ATCC53516_10880	*	*	*	09T494_01630	12T272_18010	12T273_01030	12T306_00450	CCUG54800_00070	T151023_00600	T160124_16860
3	3		1	Putative membrane protein insertion efficienc	*	ATCC53516_11330	*	*	*	*	*	*	12T306_00100	CCUG54800_18730	*	*
6	6		1	putative ABC transporter ATP-binding protein	*	ATCC53516_11360	07T609_16770	*	09T408_16570	*	12T272_17630	*	12T306_00070	*	T151023_00290	*
2	2		1	Toxin RelK	*	ATCC53516_11700	*	*	*	09T494_00880	*	*	*	*	*	*
5	5		1	hypothetical protein	*	ATCC53516_11730	*	*	09T408_16160	*	*	12T273_00180	12T306_17050	*	*	T160124_16190
1	1		1	hypothetical protein	*	ATCC53516_11750	*	*	*	*	*	*	*	*	*	*
8	8		1	Antitoxin RelJ	*	ATCC53516_11890	*	*	*	09T494_00720	12T272_16290	12T273_00030	12T306_16900	CCUG54800_18210	T151023_15600	T160124_16030
4	4		1	Plasmid encoded toxin Txe	*	ATCC53516_11900	*	*	*	09T494_00710	*	*	12T306_16890	CCUG54800_18200	*	*
3	3		1	hypothetical protein	*	ATCC53516_12040	*	*	*	*	12T272_16160	*	12T306_16780	*	*	*
3	3		1	PemK-like protein	*	ATCC53516_12050	*	*	*	*	12T272_16150	*	12T306_16770	*	*	*
7	7		1	sensory histidine kinase DcuS	*	ATCC53516_12180	*	*	09T408_15660	09T494_18510	12T272_16010	12T273_18460	*	CCUG54800_17880	*	T160124_15730
7	7		1	CAAX amino terminal protease self- immunity	*	ATCC53516_12190	*	*	09T408_15650	09T494_18500	12T272_16000	12T273_18450	*	CCUG54800_17870	*	T160124_15720
7	7		1	hypothetical protein	*	ATCC53516_12200	*	*	09T408_15640	09T494_18490	12T272_15990	12T273_18440	*	CCUG54800_17860	*	T160124_15710
6	8	0.175		Antilisterial bacteriocin subtilosin biosynthesis	*	ATCC53516_12210	*	*	09T408_15630	09T494_18480,09T494_	*	12T273_18430,12T273_	*	CCUG54800_17850	*	T160124_15700
5	5		1	hypothetical protein	*	ATCC53516_12220	*	*	*	09T494_18470	*	12T273_18420	*	CCUG54800_17840	*	T160124_15690
5	9		1	hypothetical protein	*	ATCC53516_12230,ATCC5351	*	*	*	09T494_18460	*	12T273_18410,12T273_	*	CCUG54800_17830,CCUG548	*	T160124_15680,T160124_15670
8	12	0.128		Putative multidrug export ATP-binding/permea	*	ATCC53516_12250	*	08T492_15950,08T492_15	*	09T494_18450	12T272_15040,12T272	12T273_18390,12T273_	*	CCUG54800_17810	T151023_13990,T151023_	T160124_15660
9	9	0.223		ABC-type transporter ATP-binding protein Ecs/	*	ATCC53516_12260	07T609_09370	*	09T408_15610	09T494_18440	12T272_15950	12T273_18380	12T306_02800	CCUG54800_17800	*	T160124_15650
7	7		1	ABC-2 family transporter protein	*	ATCC53516_12270	*	*	09T408_15600	09T494_18430	12T272_15940	12T273_18370	*	CCUG54800_17790	*	T160124_15640
5	5		1	Plasmid stabilisation system protein	*	ATCC53516_12330	07T609_15680	08T492_16700	*	*	12T272_15880	*	*	CCUG54800_17730	*	*
9	9		1	hypothetical protein	*	ATCC53516_12370	*	*	09T408_15480	09T494_18340	12T272_15840	12T273_18240	12T306_16560	CCUG54800_17690	T151023_15120	T160124_15560
1	1		1	Peptostreptococcal albumin-binding protein pr	*	ATCC53516_12510	*	*	*	*	*	*	*	*	*	*
1	1		1	MATE family multidrug exporter	*	ATCC53516_12580	*	*	*	*	*	*	*	*	*	*
8	8		1	putative 3-hydroxybutyryl-CoA dehydrogenase	*	ATCC53516_13460	*	*	*	09T494_06730	12T272_04500	12T273_09370	12T306_07590	CCUG54800_06830	T151023_06070	T160124_05990
9	11	0.285		Transposase	*	ATCC53516_13510	07T609_09210	*	*	09T494_18690,09T494_	12T272_13120	12T273_17650	12T306_08580	CCUG54800_19500,CCUG548	T151023_09330	T160124_13520
1	1		1	hypothetical protein	*	ATCC53516_14730	*	*	*	*	*	*	*	*	*	*
2	2		1	hypothetical protein	*	ATCC53516_15810	*	*	*	*	*	*	*	*	T151023_08410	*
1	1		1	Transposase	*	ATCC53516_15880	*	*	*	*	*	*	*	*	*	*
4	5	0.4		Transposase IS116/IS110/IS902 family protein	*	ATCC53516_15890	*	*	*	09T494_15570	12T272_12580	*	12T306_15080,12T306_	*	*	*
1	1		1	Peptidase family U32	*	ATCC53516_16420	*	*	*	*	*	*	*	*	*	*
8	8		1	GTPase HflX	*	ATCC53516_16750	*	*	*	09T494_09970	12T272_07730	12T273_12620	12T306_10850	CCUG54800_10070	T151023_09310	T160124_09240
7	7		1	Phosphoglycolate phosphatase	*	ATCC53516_16770	*	*	09T408_09030	09T494_09990	12T272_07750	12T273_12640	*	CCUG54800_10090	*	T160124_09260
7	7		1	hypothetical protein	*	ATCC53516_16780	*	*	09T408_09040	09T494_10000	12T272_07760	12T273_12650	*	CCUG54800_10100	*	T160124_09270
7	7		1	sn-glycerol-3-phosphate-binding periplasmic p	*	ATCC53516_16790	*	*	09T408_09060	09T494_10010	12T272_07770	12T273_12660	*	CCUG54800_10110	*	T160124_09280
1	1		1	Maltose/maltodextrin import ATP-binding pro	*	ATCC53516_16840	*	*	*	*	*	*	*	*	*	*
7	7		1	Glucitol operon repressor	*	ATCC53516_16850	*	*	09T408_09100	09T494_10050	12T272_07810	12T273_12710	*	CCUG54800_10150	*	T160124_09320
8	15	0.237		Alkaline phosphatase synthesis transcriptional	*	ATCC53516_16870,ATCC5351	07T609_10520,07T609_	*	09T408_09120,09T408_	09T494_10870,09T494_	12T272_08680,12T272	12T273_14120	*	CCUG54800_11740,CCUG548	*	T160124_10140,T160124_09340
5	5		1	IgA FC receptor precursor	*	ATCC53516_17020	*	*	09T408_09250	*	12T272_07960	12T273_12840	*	CCUG54800_10280	*	*
1	1		1	hypothetical protein	*	ATCC53516_17220	*	*	*	*	*	*	*	*	*	*
1	1		1	hypothetical protein	*	ATCC53516_17230	*	*	*	*	*	*	*	*	*	*
1	1		1	hypothetical protein	*	ATCC53516_17240	*	*	*	*	*	*	*	*	*	*
1	1		1	hypothetical protein	*	ATCC53516_17320	*	*	*	*	*	*	*	*	*	*
1	1		1	putative endopeptidase p60 precursor	*	ATCC53516_17350	*	*	*	*	*	*	*	*	*	*
2	2		1	Transposase, Mutator family	*	ATCC53516_17360	*	*	*	09T494_15890	*	*	*	*	*	*
1	1		1	Transposase, Mutator family	*	ATCC53516_17370	*	*	*	*	*	*	*	*	*	*
4	4		1	hypothetical protein	*	ATCC53516_17440	*	*	*	09T494_11050	*	12T273_13280	12T306_11380	*	*	*
8	9	0.556		hypothetical protein	*	ATCC53516_17490	07T609_09910	08T492_09900	09T408_14180	09T494_11100	*	12T273_13500	12T306_11610	CCUG54800_11120,CCUG548	*	*
8	16	0.25		Sensor protein KdpD	*	ATCC53516_18100,ATCC5351	07T609_10580,07T609_	*	09T408_10090,09T408_	09T494_11250,09T494_	12T272_08740,12T272	12T273_14180,12T273_	*	CCUG54800_11800,CCUG548	*	T160124_10130,T160124_10200
8	8		1	Potassium-transporting ATPase C chain	*	ATCC53516_18120	07T609_10530	*	09T408_10040	09T494_10880	12T272_08690	12T273_14130	*	CCUG54800_11750	*	T160124_10150
8	8		1	Potassium-transporting ATPase B chain	*	ATCC53516_18130	07T609_10540	*	09T408_10050	09T494_10890	12T272_08700	12T273_14140	*	CCUG54800_11760	*	T160124_10160
8	8		1	Potassium-transporting ATPase A chain	*	ATCC53516_18140	07T609_10550	*	09T408_10060	09T494_10900	12T272_08710	12T273_14150	*	CCUG54800_11770	*	T160124_10170
8	8		1	hypothetical protein	*	ATCC53516_18150	07T609_10560	*	09T408_10070	09T494_10910	12T272_08720	12T273_14160	*	CCUG54800_11780	*	T160124_10180
1	1		1	hypothetical protein	*	ATCC53516_18160	*	*	*	*	*	*	*	*	*	*
5	5		1	Hydroperoxy fatty acid reductase gpx1	*	ATCC53516_18220	*	*	*	09T494_11300	*	12T273_14240	*	CCUG54800_11850	*	T160124_10260
1	1		1	hypothetical protein	*	*	07T609_00310	*	*	*	*	*	*	*	*	*
1	1		1	hypothetical protein	*	*	07T609_00380	*	*	*	*	*	*	*	*	*
8	16		1	Elongation factor Tu-A	*	*	07T609_00450,07T609_	08T492_04400,08T492_0	09T408_04850,09T408_	*	12T272_03360,12T272	12T273_18790,12T273_	12T306_06470,12T306	CCUG54800_05720,CCUG548	T151023_15930,T151023_	*
1	1		1	N-acetylmuramoyl-L-alanine amidase LytC pre	*	*	07T609_00470	*	*	*	*	*	*	*	*	*
1	1		1	hypothetical protein	*	*	07T609_00640	*	*	*	*	*	*	*	*	*
6	6		1	alkanesulfonate transporter substrate-binding	*	*	07T609_00760	*	*	09T494_02430	12T272_00560	12T273_01840	*	CCUG54800_01500	T151023_01380	*
3	3		1	Arginine transport ATP-binding protein ArtM	*	*	07T609_00970	08T492_00500	09T408_00880	*	*	*	*	*	*	*
3	3		1	ABC-2 type transporter	*	*	07T609_00980	08T492_00510	09T408_00890	*	*	*	*	*	*	*
2	2		1	Collagen adhesin precursor	*	*	07T609_01210	*	*	*	12T272_01200	*	*	*	*	*
3	3		1	hypothetical protein	*	*	07T609_01590	*	*	*	*	*	*	CCUG54800_02420	*	T160124_13760
5	5		1	PemK-like protein	*	*	07T609_01700	*	09T408_01660	09T494_03510	12T272_13380	*	*	*	T151023_02310	*
1	1		1	Uroporphyrinogen decarboxylase (URO-D)	*	*	07T609_02030	*	*	*	*	*	*	*	*	*
2	2		1	hypothetical protein	*	*	07T609_02040	08T492_01770	*	*	*	*	*	*	*	*
1	1		1	hypothetical protein	*	*	07T609_02210	*	*	*	*	*	*	*	*	*
1	1		1	hypothetical protein	*	*	07T609_02790	*	*	*	*	*	*	*	*	*
1	1		1	HTH-type transcriptional regulator Xre	*	*	07T609_02800	*	*	*	*	*	*	*	*	*
1	1		1	Helix-turn-helix	*	*	07T609_02820	*	*	*	*	*	*	*	*	*
1	1		1	HpaII restriction endonuclease	*	*	07T609_02840	*	*	*	*	*	*	*	*	*
3	3		1	hypothetical protein	*	*	07T609_02850	*	*	*	*	12T273_03890	12T306_15040	*	*	*
6	6		1	Oleate hydratase	*	*	07T609_03090	08T492_02770	*	09T494_03850	12T272_01580	12T273_06620	12T306_04810	*	*	*
1	1		1	hypothetical protein	*	*	07T609_03130	*	*	*	*	*	*	*	*	*
2	3		1	Glycine/sarcosine/betaine reductase complex	*	*	07T609_04050,07T609_	08T492_03820	*	*	*	*	*	*	*	*
1	1		1	Dihydrolipoyl dehydrogenase	*	*	07T609_04440	*	*	*	*	*	*	*	*	*
1	1		1	hypothetical protein	*	*	07T609_06950	*	*	*	*	*	*	*	*	*
1	1		1	hypothetical protein	*	*	07T609_08030	*	*	*	*	*	*	*	*	*
1	1		1	hypothetical protein	*	*	07T609_09060	*	*	*	*	*	*	*	*	*
1	1		1	hypothetical protein	*	*	07T609_09160	*	*	*	*	*	*	*	*	*
3	5	0.401		Lipoprotein-releasing system ATP-binding prot	*	*	07T609_09250,07T609_	08T492_09110,08T492_10	*	*	*	*	12T306_12390	*	*	*
2	2		1	Macrolide export ATP-binding/permease prote	*	*	07T609_09260	08T492_09120	*	*	*	*	*	*	*	*
2	2		1	Macrolide export ATP-binding/permease prote	*	*	07T609_09270	08T492_09130	*	*	*	*	*	*	*	*
2	2		1	hypothetical protein	*	*	07T609_09280	08T492_09140	*	*	*	*	*	*	*	*
2	2		1	34 kDa membrane antigen precursor	*	*	07T609_09290	08T492_09150	*	*	*	*	*	*	*	*
2	2		1	Ferrous iron permease EfeU precursor	*	*	07T609_09300	08T492_09160	*	*	*	*	*	*	*	*
2	2		1	hypothetical protein	*	*	07T609_09320	08T492_09180	*	*	*	*	*	*	*	*
1	1		1	Ferrous iron transport protein B	*	*	07T609_09330	*	*	*	*	*	*	*	*	*
2	2		1	FeoA domain protein	*	*	07T609_09340	08T492_09210	*	*	*	*	*	*	*	*
1	1		1	hypothetical protein	*	*	07T609_09350	*	*	*	*	*	*	*	*	*
2	2		1	ABC-2 family transporter protein	*	*	07T609_09360	*	*	*	*	*	12T306_02810	*	*	*
1	1		1	hypothetical protein	*	*	07T609_09380	*	*	*	*	*	*	*	*	*
2	2		1	hypothetical protein	*	*	07T609_09390	*	*	*	*	*	12T306_02790	*	*	*
7	7		1	Transcriptional regulatory protein ComA	*	*	07T609_09400	08T492_13520	*	09T494_16200	12T272_17390	12T273_16770	12T306_02780	CCUG54800_19080	*	*
7	7		1	hypothetical protein	*	*	07T609_09410	08T492_13530	*	09T494_16210	12T272_17400	12T273_16780	12T306_02770	CCUG54800_19070	*	*
6	6		1	PrgI family protein	*	*	07T609_09520	08T492_13660	*	09T494_14040	12T272_17510	12T273_16900	*	CCUG54800_10690	*	*
1	1		1	hypothetical protein	*	*	07T609_09580	*	*	*	*	*	*	*	*	*
6	7	0.241		hypothetical protein	*	*	07T609_09640,07T609_	08T492_09770	09T408_14030	*	*	12T273_13350	12T306_11430	CCUG54800_10980	*	*
5	5		1	hypothetical protein	*	*	07T609_09660	08T492_09790	09T408_14050	*	*	12T273_13370	*	CCUG54800_11000	*	*
3	3		1	hypothetical protein	*	*	07T609_09680	08T492_09810	09T408_14070	*	*	*	*	*	*	*
1	1		1	hypothetical protein	*	*	07T609_09690	*	*	*	*	*	*	*	*	*
6	7	0.136		Energy-coupling factor transporter transmemb	*	*	07T609_09700	08T492_15420,08T492_09	*	09T494_15490	12T272_12490	*	12T306_16080	CCUG54800_10420	*	*
3	5	0.401		Putative HMP/thiamine import ATP-binding pr	*	*	07T609_09710,07T609_	08T492_15360	*	*	*	*	*	CCUG54800_15600,CCUG548	*	*
1	1		1	Putative multidrug export ATP-binding/permea	*	*	07T609_09720	*	*	*	*	*	*	*	*	*
1	1		1	putative HTH-type transcriptional regulator Ytt	*	*	07T609_09750	*	*	*	*	*	*	*	*	*
1	1		1	Type IV secretory system Conjugative DNA trar	*	*	07T609_09770	*	*	*	*	*	*	*	*	*
1	1		1	hypothetical protein	*	*	07T609_09780	*	*	*	*	*	*	*	*	*
1	1		1	hypothetical protein	*	*	07T609_09790	*	*	*	*	*	*	*	*	*
1	1		1	Choloylglycine hydrolase	*	*	07T609_09810	*	*	*	*	*	*	*	*	*
1	1		1	Mobilization protein A	*	*	07T609_09830	*	*	*	*	*	*	*	*	*
1	1		1	hypothetical protein	*	*	07T609_09840	*	*	*	*	*	*	*	*	*
1	1		1	putative ABC transporter ATP-binding protein '	*	*	07T609_09850	*	*	*	*	*	*	*	*	*
2	2		1	hypothetical protein	*	*	07T609_10570	*	09T408_10080	*	*	*	*	*	*	*
4	4		1	NADP-reducing hydrogenase subunit HndC	*	*	07T609_12800	08T492_12870	09T408_12250	*	*	*	*	*	*	T160124_12610
3	3		1	Heparinase II/III-like protein	*	*	07T609_13300	*	09T408_12730	*	*	*	12T306_14970	*	*	*
1	1		1	Extracellular matrix-binding protein ebh precu	*	*	07T609_13520	*	*	*	*	*	*	*	*	*
1	1		1	Serine-rich adhesin for platelets precursor	*	*	07T609_13530	*	*	*	*	*	*	*	*	*
1	1		1	hypothetical protein	*	*	07T609_13540	*	*	*	*	*	*	*	*	*
6	7	0.248		hypothetical protein	*	*	07T609_13900	08T492_15000	09T408_13400	09T494_16990	12T272_14420	*	*	CCUG54800_15450,CCUG548	*	*
4	4		1	hypothetical protein	*	*	07T609_13930	08T492_15030	*	09T494_17020	*	*	*	*	T151023_03110	*
3	3		1	Divergent AAA domain protein	*	*	07T609_13940	08T492_15040	*	*	*	*	*	*	T151023_03100	*
4	4		1	hypothetical protein	*	*	07T609_13950	08T492_15050	*	*	*	*	*	*	T151023_03090	T160124_13430
1	1		1	hypothetical protein	*	*	07T609_14000	*	*	*	*	*	*	*	*	*
1	1		1	hypothetical protein	*	*	07T609_14010	*	*	*	*	*	*	*	*	*
1	1		1	2-succinyl-6-hydroxy-2,4-cyclohexadiene-1-car	*	*	07T609_14020	*	*	*	*	*	*	*	*	*
1	1		1	putative methyltransferase YcgJ	*	*	07T609_14030	*	*	*	*	*	*	*	*	*
1	1		1	hypothetical protein	*	*	07T609_14040	*	*	*	*	*	*	*	*	*
1	1		1	Transposon Tn10 TetD protein	*	*	07T609_14250	*	*	*	*	*	*	*	*	*
1	1		1	hypothetical protein	*	*	07T609_14260	*	*	*	*	*	*	*	*	*
1	1		1	hypothetical protein	*	*	07T609_14270	*	*	*	*	*	*	*	*	*
2	2		1	hypothetical protein	*	*	07T609_14280	*	*	09T494_18580	*	*	*	*	*	*
1	1		1	DEAD/DEAH box helicase	*	*	07T609_14330	*	*	*	*	*	*	*	*	*
1	1		1	hypothetical protein	*	*	07T609_14340	*	*	*	*	*	*	*	*	*
1	1		1	hypothetical protein	*	*	07T609_14360	*	*	*	*	*	*	*	*	*
1	1		1	hypothetical protein	*	*	07T609_14390	*	*	*	*	*	*	*	*	*
1	1		1	Iron import ATP-binding/permease protein Irt/	*	*	07T609_14450	*	*	*	*	*	*	*	*	*
2	2		1	hypothetical protein	*	*	07T609_14490	08T492_18340	*	*	*	*	*	*	*	*
2	2		1	Arabinose operon regulatory protein	*	*	07T609_14520	*	*	*	*	*	*	CCUG54800_15800	*	*
1	1		1	hypothetical protein	*	*	07T609_14530	*	*	*	*	*	*	*	*	*
4	4		1	hypothetical protein	*	*	07T609_14540	08T492_09670	*	*	12T272_13840	*	12T306_11230	*	*	*
1	1		1	hypothetical protein	*	*	07T609_14570	*	*	*	*	*	*	*	*	*
3	3		1	anaerobic benzoate catabolism transcriptional	*	*	07T609_14810	08T492_15770	*	*	*	*	*	*	*	T160124_14650
2	2		1	hypothetical protein	*	*	07T609_14820	08T492_15780	*	*	*	*	*	*	*	*
1	1		1	Peptostreptococcal albumin-binding protein pr	*	*	07T609_15520	*	*	*	*	*	*	*	*	*
6	7	0.369		Abi-like protein	*	*	07T609_15770	08T492_13710,08T492_16	*	09T494_14090	12T272_17560	12T273_16950	*	CCUG54800_19400	*	*
2	2		1	taurine transporter substrate binding subunit	*	*	07T609_15900	*	*	*	*	*	*	*	T151023_15350	*
2	2		1	HTH-type transcriptional regulator CueR	*	*	07T609_15910	*	*	*	*	*	*	*	T151023_15360	*
2	2		1	hypothetical protein	*	*	07T609_15920	*	*	*	*	*	*	*	T151023_15370	*
2	2		1	putative permease	*	*	07T609_15930	*	*	*	*	*	*	*	T151023_15380	*
2	2		1	hypothetical protein	*	*	07T609_15940	*	*	*	*	*	*	*	T151023_15390	*
2	2		1	hypothetical protein	*	*	07T609_15950	*	*	*	*	*	*	*	T151023_15400	*
2	2		1	Alkylmercury lyase	*	*	07T609_15960	*	*	*	*	*	*	*	T151023_15410	*
2	2		1	Bicarbonate transport system permease prote	*	*	07T609_15980	*	*	*	*	*	*	*	T151023_15430	*
2	2		1	hypothetical protein	*	*	07T609_15990	*	*	*	*	*	*	*	T151023_15440	*
2	2		1	hypothetical protein	*	*	07T609_16000	*	*	*	*	*	*	*	T151023_15450	*
2	2		1	hypothetical protein	*	*	07T609_16010	*	*	*	*	*	*	*	T151023_15460	*
1	1		1	sn-glycerol-3-phosphate-binding periplasmic p	*	*	07T609_16080	*	*	*	*	*	*	*	*	*
1	1		1	putative glycerophosphoryl diester phosphodi	*	*	07T609_16090	*	*	*	*	*	*	*	*	*
1	1		1	Calcium-transporting ATPase	*	*	07T609_16100	*	*	*	*	*	*	*	*	*
1	1		1	Alpha/beta hydrolase family protein	*	*	07T609_16110	*	*	*	*	*	*	*	*	*
4	4		1	hypothetical protein	*	*	07T609_16230	*	*	*	12T272_18060	12T273_00090	*	*	*	T160124_16100
1	1		1	hypothetical protein	*	*	07T609_16270	*	*	*	*	*	*	*	*	*
1	1		1	hypothetical protein	*	*	07T609_16280	*	*	*	*	*	*	*	*	*
1	1		1	hypothetical protein	*	*	07T609_16400	*	*	*	*	*	*	*	*	*
2	2		1	hypothetical protein	*	*	07T609_16420	08T492_17220	*	*	*	*	*	*	*	*
2	2		1	hypothetical protein	*	*	07T609_16430	08T492_17230	*	*	*	*	*	*	*	*
7	7		1	hypothetical protein	*	*	07T609_16590	*	*	09T494_01090	12T272_16710	12T273_00440	12T306_17330	*	T151023_00130	T160124_16360
2	2		1	Arginine utilization regulatory protein RocR	*	*	07T609_16740	*	*	*	*	*	12T306_00040	*	*	*
2	2		1	Guanidinobutyrase	*	*	07T609_16750	*	*	*	*	*	12T306_00050	*	*	*
1	1		1	R3H domain protein	*	*	07T609_16780	*	*	*	*	*	*	*	*	*
2	2		1	Plasmid recombination enzyme	*	*	07T609_17190	08T492_18290	*	*	*	*	*	*	*	*
2	2		1	Type IV secretory system Conjugative DNA trar	*	*	07T609_17200	08T492_18300	*	*	*	*	*	*	*	*
2	2		1	hypothetical protein	*	*	07T609_17210	08T492_18310	*	*	*	*	*	*	*	*
5	6	0.554		Group II intron-encoded protein LtrA	*	*	07T609_17230	08T492_09680	*	*	*	12T273_13080	*	CCUG54800_19430,CCUG548	T151023_15960	*
6	6		1	hypothetical protein	*	*	07T609_17270	08T492_18390	*	*	12T272_18220	*	12T306_17560	CCUG54800_19530	T151023_15990	*
1	1		1	hypothetical protein	*	*	*	08T492_00190	*	*	*	*	*	*	*	*
2	2		1	Bacteriocin (Lactococcin_972)	*	*	*	08T492_00470	*	*	12T272_00870	*	*	*	*	*
1	1		1	Toxin Doc	*	*	*	08T492_01350	*	*	*	*	*	*	*	*
1	1		1	hypothetical protein	*	*	*	08T492_01480	*	*	*	*	*	*	*	*
1	1		1	Multidrug export protein MepA	*	*	*	08T492_01500	*	*	*	*	*	*	*	*
1	1		1	NADP-dependent isopropanol dehydrogenase	*	*	*	08T492_01540	*	*	*	*	*	*	*	*
1	1		1	ABC-2 family transporter protein	*	*	*	08T492_01820	*	*	*	*	*	*	*	*
1	1		1	ABC-2 family transporter protein	*	*	*	08T492_01830	*	*	*	*	*	*	*	*
1	1		1	hypothetical protein	*	*	*	08T492_02520	*	*	*	*	*	*	*	*
2	2		1	HTH-type transcriptional regulator PuuR	*	*	*	08T492_02530	*	*	*	*	12T306_15580	*	*	*
2	2		1	hypothetical protein	*	*	*	08T492_02540	*	*	*	*	12T306_15570	*	*	*

1	1	1	hypothetical protein	*	*	*	08T492_02690	*	*	*	*	*	*	*	*
1	1	1	Transposase from transposon Tn916	*	*	*	08T492_05150	*	*	*	*	*	*	*	*
1	1	1	hypothetical protein	*	*	*	08T492_05160	*	*	*	*	*	*	*	*
1	1	1	hypothetical protein	*	*	*	08T492_05170	*	*	*	*	*	*	*	*
1	1	1	SAP domain protein	*	*	*	08T492_05180	*	*	*	*	*	*	*	*
1	1	1	LexA repressor	*	*	*	08T492_05190	*	*	*	*	*	*	*	*
1	1	1	hypothetical protein	*	*	*	08T492_05200	*	*	*	*	*	*	*	*
1	1	1	hypothetical protein	*	*	*	08T492_05210	*	*	*	*	*	*	*	*
4	4	0.5	hypothetical protein	*	*	*	08T492_05220	*	09T494_14580	12T272_11550	*	12T306_14990	*	*	*
1	1	1	hypothetical protein	*	*	*	08T492_05230	*	*	*	*	*	*	*	*
1	1	1	hypothetical protein	*	*	*	08T492_07880	*	*	*	*	*	*	*	*
1	1	1	hypothetical protein	*	*	*	08T492_09070	*	*	*	*	*	*	*	*
1	1	1	hypothetical protein	*	*	*	08T492_09080	*	*	*	*	*	*	*	*
1	1	1	Ferrous iron transport protein B	*	*	*	08T492_09190	*	*	*	*	*	*	*	*
1	1	1	ferrous iron transport protein A	*	*	*	08T492_09200	*	*	*	*	*	*	*	*
1	1	1	sporulation sigma factor SigF	*	*	*	08T492_09260	*	*	*	*	*	*	*	*
1	1	1	Transposase, Mutator family	*	*	*	08T492_09370	*	*	*	*	*	*	*	*
1	1	1	hypothetical protein	*	*	*	08T492_09380	*	*	*	*	*	*	*	*
7	8	0.335	Putative HMP/thiamine import ATP-binding pr	*	*	*	08T492_09390,08T492_13	09T408_14080	09T494_15480	12T272_12480	12T273_13000	12T306_16070	CCUG54800_10410	*	*
1	1	1	hypothetical protein	*	*	*	08T492_09410	*	*	*	*	*	*	*	*
1	1	1	putative ABC transporter ATP-binding protein	*	*	*	08T492_09420	*	*	*	*	*	*	*	*
1	1	1	hypothetical protein	*	*	*	08T492_09430	*	*	*	*	*	*	*	*
1	1	1	Chromosome partition protein Smc	*	*	*	08T492_09490	*	*	*	*	*	*	*	*
1	1	1	hypothetical protein	*	*	*	08T492_09550	*	*	*	*	*	*	*	*
1	1	1	hypothetical protein	*	*	*	08T492_09560	*	*	*	*	*	*	*	*
1	1	1	hypothetical protein	*	*	*	08T492_09590	*	*	*	*	*	*	*	*
1	1	1	Glucose-1-phosphate thymidylyltransferase 2	*	*	*	08T492_09600	*	*	*	*	*	*	*	*
1	2	1	Putative HMP/thiamine import ATP-binding pr	*	*	*	08T492_09610,08T492_15	*	*	*	*	*	*	*	*
1	2	1	hypothetical protein	*	*	*	08T492_09630,08T492_15	*	*	*	*	*	*	*	*
1	1	1	Putative multidrug export ATP-binding/permea	*	*	*	08T492_09640	*	*	*	*	*	*	*	*
3	3	1	Lipid A export ATP-binding/permease protein I	*	*	*	08T492_09650	*	*	*	12T273_12980	12T306_11510	*	*	*
5	7	0.305	Diphtheria toxin repressor	*	*	*	08T492_09660,08T492_13	*	09T494_13910	12T272_17150	12T273_17860	*	CCUG54800_19240,CCUG548	*	*
1	1	1	Phosphoenolpyruvate synthase	*	*	*	08T492_09830	*	*	*	*	*	*	*	*
1	1	1	DNA-binding transcriptional repressor AcrR	*	*	*	08T492_09840	*	*	*	*	*	*	*	*
1	1	1	hypothetical protein	*	*	*	08T492_12120	*	*	*	*	*	*	*	*
2	2	1	hypothetical protein	*	*	*	08T492_12370	09T408_11760	*	*	*	*	*	*	*
1	1	1	Dipeptide-binding protein DppE precursor	*	*	*	08T492_12760	*	*	*	*	*	*	*	*
1	1	1	Antilisterial bacteriocin subtilosin biosynthesis	*	*	*	08T492_12930	*	*	*	*	*	*	*	*
6	6	0.5	putative multidrug resistance ABC transporter	*	*	*	08T492_12990	09T408_15620	09T494_13640	12T272_16940	12T273_16560	*	CCUG54800_14240	*	*
5	6	0.271	Recombinase	*	*	*	08T492_13010	*	09T494_13660	12T272_16960	12T273_16580	*	CCUG54800_14260,CCUG548	*	*
5	6	0.271	hypothetical protein	*	*	*	08T492_13020	*	09T494_13670	12T272_16970	12T273_16600	*	CCUG54800_14270,CCUG548	*	*
5	6	0.271	hypothetical protein	*	*	*	08T492_13030	*	09T494_13680	12T272_16980	12T273_16610	*	CCUG54800_14280,CCUG548	*	*
5	5	1	hypothetical protein	*	*	*	08T492_13040	*	09T494_13690	12T272_16990	12T273_16620	*	CCUG54800_14290	*	*
3	3	1	RNA polymerase sigma factor SigD	*	*	*	08T492_13050	*	*	*	12T273_16630	*	CCUG54800_14300	*	*
1	1	1	Cobalt transport protein	*	*	*	08T492_13110	*	*	*	*	*	*	*	*
1	1	1	Phenyloxazoline synthase MbtB	*	*	*	08T492_13130	*	*	*	*	*	*	*	*
2	2	1	hypothetical protein	*	*	*	08T492_13140	*	09T494_13830	*	*	*	*	*	*
2	2	1	Phosphopantetheine attachment site	*	*	*	08T492_13150	*	09T494_13840	*	*	*	*	*	*
3	3	1	Phenyloxazoline synthase MbtB	*	*	*	08T492_13160	*	09T494_13850	*	*	*	CCUG54800_14450	*	*
3	4	0.36	Oxidoreductase family, NAD-binding Rossmann	*	*	*	08T492_13170	*	09T494_13860,09T494_	*	*	*	CCUG54800_14400	*	*
4	6	0.219	Phenyloxazoline synthase MbtB	*	*	*	08T492_13180	*	09T494_13810,09T494_	12T272_17100	*	*	CCUG54800_15660,CCUG548	*	*
4	5	0.31	2,3-dihydroxybenzoate-AMP ligase	*	*	*	08T492_13190	*	09T494_13880	12T272_17120	*	*	CCUG54800_19360,CCUG548	*	*
4	4	1	4'-phosphopantetheinyl transferase sfp	*	*	*	08T492_13200	*	09T494_13890	12T272_17130	*	*	CCUG54800_19490	*	*
4	4	1	Linear gramicidin dehydrogenase LgrE	*	*	*	08T492_13210	*	09T494_13900	12T272_17140	*	*	CCUG54800_19480	*	*
1	1	1	Isochorismate synthase/isochorismate lyase	*	*	*	08T492_13280	*	*	*	*	*	*	*	*
5	5	1	hypothetical protein	*	*	*	08T492_13300	*	09T494_13920	12T272_17160	12T273_17870	*	CCUG54800_10560	*	*
3	3	1	ABC-2 family transporter protein	*	*	*	08T492_13320	*	*	12T272_17290	12T273_17890	*	*	*	*
3	3	1	ABC-2 family transporter protein	*	*	*	08T492_13330	*	*	12T272_17300	12T273_17900	*	*	*	*
3	3	1	Peptidase family M50	*	*	*	08T492_13350	*	*	12T272_17320	12T273_17920	*	*	*	*
4	5	0.465	Anaerobic sulfatase-maturating enzyme	*	*	*	08T492_13360,08T492_15	*	*	12T272_17330	12T273_17930	*	*	T151023_14010	*
3	3	1	hypothetical protein	*	*	*	08T492_13370	*	*	12T272_17340	12T273_17940	*	*	*	*
4	4	1	hypothetical protein	*	*	*	08T492_13380	*	09T494_16050	*	12T273_17950	*	CCUG54800_19320	*	*
4	5	1	hypothetical protein	*	*	*	08T492_13390	*	*	*	12T273_17960	12T306_16000,12T306_	CCUG54800_19330	*	*
5	6	0.271	hypothetical protein	*	*	*	08T492_13400	*	09T494_16060,09T494_	12T272_17260	12T273_17970	*	CCUG54800_19340	*	*
2	2	1	hypothetical protein	*	*	*	08T492_13410	*	*	*	12T273_17980	*	*	*	*
2	2	1	N5-glutamine S-adenosyl-L-methionine-depen	*	*	*	08T492_13420	*	*	*	12T273_17990	*	*	*	*
2	2	1	hypothetical protein	*	*	*	08T492_13430	*	*	*	12T273_18000	*	*	*	*
1	1	1	Cyclic pyranopterin monophosphate synthase	*	*	*	08T492_13440	*	*	*	*	*	*	*	*
2	2	1	Putative multidrug export ATP-binding/permea	*	*	*	08T492_13450	*	*	*	12T273_18020	*	*	*	*
2	2	1	hypothetical protein	*	*	*	08T492_13460	*	*	*	12T273_18040	*	*	*	*
5	6	0.271	putative ABC transporter ATP-binding protein	*	*	*	08T492_13470	*	09T494_16160,09T494_	12T272_17350	12T273_16730	*	CCUG54800_19120	*	*
5	5	1	hypothetical protein	*	*	*	08T492_13480	*	09T494_16170	12T272_17360	12T273_16740	*	CCUG54800_19110	*	*
5	5	1	Antilisterial bacteriocin subtilosin biosynthesis	*	*	*	08T492_13490	*	09T494_16180	12T272_17370	12T273_16750	*	CCUG54800_19100	*	*
5	5	1	hypothetical protein	*	*	*	08T492_13500	*	09T494_16190	12T272_17380	12T273_16760	*	CCUG54800_19090	*	*
1	1	1	transcriptional regulator FimZ	*	*	*	08T492_13510	*	*	*	*	*	*	*	*
5	5	1	hypothetical protein	*	*	*	08T492_13540	*	09T494_13940	12T272_17410	12T273_16790	*	CCUG54800_10580	*	*
5	5	1	hypothetical protein	*	*	*	08T492_13560	*	09T494_13960	12T272_17430	12T273_16810	*	CCUG54800_10610	*	*
3	3	1	hypothetical protein	*	*	*	08T492_13580	*	*	*	12T273_12910	*	*	*	T160124_09530
3	3	1	hypothetical protein	*	*	*	08T492_13620	*	*	12T272_17470	12T273_16860	*	*	*	*
5	5	1	putative endopeptidase p60 precursor	*	*	*	08T492_13630	*	09T494_14010	12T272_17480	12T273_16870	*	CCUG54800_10660	*	*
5	5	1	Modification methylase DpnIIB	*	*	*	08T492_13640	*	09T494_14020	12T272_17490	12T273_16880	*	CCUG54800_10670	*	*
5	5	1	hypothetical protein	*	*	*	08T492_13670	*	09T494_14050	12T272_17520	12T273_16910	*	CCUG54800_19460	*	*
1	1	1	hypothetical protein	*	*	*	08T492_13760	*	*	*	*	*	*	*	*
1	1	1	hypothetical protein	*	*	*	08T492_13780	*	*	*	*	*	*	*	*
1	1	1	RDD family protein	*	*	*	08T492_13790	*	*	*	*	*	*	*	*
1	1	1	ABC-2 family transporter protein	*	*	*	08T492_13820	*	*	*	*	*	*	*	*
1	1	1	ABC-2 family transporter protein	*	*	*	08T492_13830	*	*	*	*	*	*	*	*
1	1	1	Fluoroquinolones export ATP-binding protein/	*	*	*	08T492_13840	*	*	*	*	*	*	*	*
1	1	1	hypothetical protein	*	*	*	08T492_13860	*	*	*	*	*	*	*	*
1	1	1	hypothetical protein	*	*	*	08T492_13870	*	*	*	*	*	*	*	*
4	4	1	hypothetical protein	*	*	*	08T492_13950	*	09T494_14110	12T272_17580	*	*	CCUG54800_14480	*	*
5	7	0.286	hypothetical protein	*	*	*	08T492_13960	*	09T494_11080,09T494_	12T272_17590	12T273_16970,12T273_	*	CCUG54800_14490	*	*
5	5	1	hypothetical protein	*	*	*	08T492_13980	*	09T494_14140	12T272_17610	12T273_16990	*	CCUG54800_14510	*	*
6	6	0.333	hypothetical protein	*	*	*	08T492_13990	09T408_14190	09T494_14150	12T272_17620	12T273_17000	*	CCUG54800_14520	*	*
1	1	1	hypothetical protein	*	*	*	08T492_14570	*	*	*	*	*	*	*	*
1	1	1	Listeria-Bacteroides repeat domain (List_Bact_	*	*	*	08T492_14640	*	*	*	*	*	*	*	*
1	1	1	hypothetical protein	*	*	*	08T492_15250	*	*	*	*	*	*	*	*
1	1	1	hypothetical protein	*	*	*	08T492_15260	*	*	*	*	*	*	*	*
1	1	1	hypothetical protein	*	*	*	08T492_15270	*	*	*	*	*	*	*	*
1	1	1	hypothetical protein	*	*	*	08T492_15300	*	*	*	*	*	*	*	*
1	1	1	hypothetical protein	*	*	*	08T492_15310	*	*	*	*	*	*	*	*
1	1	1	Lipid A export ATP-binding/permease protein I	*	*	*	08T492_15330	*	*	*	*	*	*	*	*
1	1	1	Putative multidrug export ATP-binding/permea	*	*	*	08T492_15340	*	*	*	*	*	*	*	*
1	1	1	hypothetical protein	*	*	*	08T492_15350	*	*	*	*	*	*	*	*
1	1	1	multidrug efflux pump VmrA	*	*	*	08T492_15390	*	*	*	*	*	*	*	*
1	1	1	Multidrug export protein MepA	*	*	*	08T492_15400	*	*	*	*	*	*	*	*
1	1	1	Relaxase/Mobilisation nuclease domain protei	*	*	*	08T492_15480	*	*	*	*	*	*	*	*
2	2	1	hypothetical protein	*	*	*	08T492_15490	*	*	*	*	*	*	*	T160124_14040
1	1	1	hypothetical protein	*	*	*	08T492_15500	*	*	*	*	*	*	*	*
1	1	1	hypothetical protein	*	*	*	08T492_15510	*	*	*	*	*	*	*	*
1	1	1	hypothetical protein	*	*	*	08T492_15870	*	*	*	*	*	*	*	*
1	1	1	hypothetical protein	*	*	*	08T492_15980	*	*	*	*	*	*	*	*
1	1	1	Putative amidase domain protein	*	*	*	08T492_16020	*	*	*	*	*	*	*	*
1	1	1	Peptostreptococcal albumin-binding protein pr	*	*	*	08T492_16540	*	*	*	*	*	*	*	*
1	1	1	hypothetical protein	*	*	*	08T492_16890	*	*	*	*	*	*	*	*
5	6	0.212	Type I restriction enzyme EcoR124II R protein	*	*	*	08T492_16920	*	*	*	12T273_03130,12T273_	*	CCUG54800_16720	T151023_15480	T160124_15920
3	3	1	Putative type-1 restriction enzyme specificity p	*	*	*	08T492_16930	*	*	*	*	*	*	T151023_15490	T160124_15930
1	1	1	hypothetical protein	*	*	*	08T492_17330	*	*	*	*	*	*	*	*
5	5	1	Thiosulfate sulfurtransferase YnjE precursor	*	*	*	08T492_17700	*	09T494_01460	12T272_17870	12T273_00900	*	*	*	T160124_16740
5	5	1	hypothetical protein	*	*	*	08T492_17710	*	09T494_01470	12T272_17880	12T273_00910	*	*	*	T160124_16750
1	1	1	hypothetical protein	*	*	*	08T492_17830	*	*	*	*	*	*	*	*
1	1	1	hypothetical protein	*	*	*	08T492_17840	*	*	*	*	*	*	*	*
1	1	1	N-acetylmuramoyl-L-alanine amidase LytC pre	*	*	*	08T492_17850	*	*	*	*	*	*	*	*
1	1	1	ATP-dependent helicase HepA	*	*	*	08T492_18240	*	*	*	*	*	*	*	*
1	1	1	hypothetical protein	*	*	*	08T492_18250	*	*	*	*	*	*	*	*
2	2	1	putative methyltransferase	*	*	*	08T492_18260	09T408_15530	*	*	*	*	*	*	*
2	2	1	Type III restriction enzyme, res subunit	*	*	*	08T492_18270	09T408_15520	*	*	*	*	*	*	*
1	1	1	hypothetical protein	*	*	*	08T492_18380	*	*	*	*	*	*	*	*
1	1	1	hypothetical protein	*	*	*	*	09T408_00090	*	*	*	*	*	*	*
2	2	1	Type I restriction modification DNA specificity	*	*	*	*	09T408_00400	*	*	*	*	*	*	T160124_00290
2	2	1	Type I restriction enzyme EcoR124II R protein	*	*	*	*	09T408_00410	*	*	*	*	*	*	T160124_00300
1	1	1	N-acetylmuramoyl-L-alanine amidase LytC pre	*	*	*	*	09T408_00440	*	*	*	*	*	*	*
1	1	1	hypothetical protein	*	*	*	*	09T408_00590	*	*	*	*	*	*	*
1	1	1	hypothetical protein	*	*	*	*	09T408_01100	*	*	*	*	*	*	*
2	2	1	hypothetical protein	*	*	*	*	09T408_01460	*	*	*	12T306_01990	*	*	*
2	2	1	hypothetical protein	*	*	*	*	09T408_01550	09T494_03380	*	*	*	*	*	*
1	1	1	hypothetical protein	*	*	*	*	09T408_01650	*	*	*	*	*	*	*
2	2	1	hypothetical protein	*	*	*	*	09T408_02820	*	*	*	*	*	T151023_12870	*
3	3	1	hypothetical protein	*	*	*	*	09T408_02830	09T494_14650	*	*	*	*	T151023_12860	*
1	1	1	hypothetical protein	*	*	*	*	09T408_02840	*	*	*	*	*	*	*
1	1	1	CRISPR-associated nuclease/helicase Cas3	*	*	*	*	09T408_02850	*	*	*	*	*	*	*
1	1	1	CRISPR-associated protein Cse2 (CRISPR_cse2)	*	*	*	*	09T408_02870	*	*	*	*	*	*	*
1	1	1	CRISPR-associated endonuclease Cas1	*	*	*	*	09T408_02910	*	*	*	*	*	*	*
2	2	1	DNA polymerase III PolC-type	*	*	*	*	09T408_02920	09T494_00470	*	*	*	*	*	*
1	1	1	hypothetical protein	*	*	*	*	09T408_03010	*	*	*	*	*	*	*
1	1	1	hypothetical protein	*	*	*	*	09T408_03020	*	*	*	*	*	*	*
1	1	1	hypothetical protein	*	*	*	*	09T408_03030	*	*	*	*	*	*	*
1	1	1	ADP-ribosyltransferase exoenzyme	*	*	*	*	09T408_03040	*	*	*	*	*	*	*
1	1	1	hypothetical protein	*	*	*	*	09T408_03050	*	*	*	*	*	*	*
1	1	1	hypothetical protein	*	*	*	*	09T408_03060	*	*	*	*	*	*	*
4	4	0.5	hypothetical protein	*	*	*	*	09T408_03250	09T494_03910	12T272_01630	12T273_06670	*	*	*	*
2	2	1	hypothetical protein	*	*	*	*	09T408_03280	*	*	12T273_06710	*	*	*	*
1	1	1	hypothetical protein	*	*	*	*	09T408_03850	*	*	*	*	*	*	*
1	1	1	Alpha-amylase precursor	*	*	*	*	09T408_03860	*	*	*	*	*	*	*
2	4	1	UDP-4-amino-4-deoxy-L-arabinose--oxoglutara	*	*	*	*	09T408_05740,09T408_	*	12T272_14760,12T272_	*	*	*	*	*
3	3	1	3-hydroxybutyryl-CoA dehydrogenase	*	*	*	*	09T408_05810	*	*	12T273_09360	*	*	*	T160124_05980
1	1	1	DNA mismatch repair protein MutL	*	*	*	*	09T408_07480	*	*	*	*	*	*	*
1	1	1	hypothetical protein	*	*	*	*	09T408_08110	*	*	*	*	*	*	*
1	1	1	hypothetical protein	*	*	*	*	09T408_09050	*	*	*	*	*	*	*
1	2	1	Transposase IS200 like protein	*	*	*	*	09T408_09320,09T408_	*	*	*	*	*	*	*
1	1	1	Transposase IS200 like protein	*	*	*	*	09T408_09330	*	*	*	*	*	*	*
1	1	1	putative ABC transporter permease protein	*	*	*	*	09T408_09380	*	*	*	*	*	*	*
1	1	1	hypothetical protein	*	*	*	*	09T408_09700	*	*	*	*	*	*	*
1	1	1	Glycogen synthase	*	*	*	*	09T408_09910	*	*	*	*	*	*	*
1	1	1	hypothetical protein	*	*	*	*	09T408_11520	*	*	*	*	*	*	*
2	2	1	hypothetical protein	*	*	*	*	09T408_12760	09T494_16390	*	*	*	*	*	*
2	2	1	hypothetical protein	*	*	*	*	09T408_12980	*	*	*	12T306_15170	*	*	*
1	1	1	hypothetical protein	*	*	*	*	09T408_12990	*	*	*	*	*	*	*
1	1	1	hypothetical protein	*	*	*	*	09T408_13000	*	*	*	*	*	*	*
1	1	1	hypothetical protein	*	*	*	*	09T408_13010	*	*	*	*	*	*	*
1	1	1	hypothetical protein	*	*	*	*	09T408_13020	*	*	*	*	*	*	*
1	1	1	hypothetical protein	*	*	*	*	09T408_13030	*	*	*	*	*	*	*
2	2	1	hypothetical protein	*	*	*	*	09T408_13170	*	*	12T273_17400	*	*	*	*
4	4	1	Putative NAD(P)H nitroreductase YodC	*	*	*	*	09T408_13350	*	*	*	12T306_15540	CCUG54800_15400	*	T160124_13330
5	5	1	transcriptional regulator SlyA	*	*	*	*	09T408_13360	*	*	12T273_17550	12T306_15550	CCUG54800_15410	*	T160124_13340
2	2	1	hypothetical protein	*	*	*	*	09T408_13460	09T494_17030	*	*	*	*	*	*
1	1	1	hypothetical protein	*	*	*	*	09T408_13470	*	*	*	*	*	*	*
1	1	1	hypothetical protein	*	*	*	*	09T408_13520	*	*	*	*	*	*	*
3	3	1	hypothetical protein	*	*	*	*	09T408_13700	*	*	12T273_17730	12T306_02710	*	*	*
1	1	1	hypothetical protein	*	*	*	*	09T408_13720	*	*	*	*	*	*	*
1	1	1	hypothetical protein	*	*	*	*	09T408_13730	*	*	*	*	*	*	*
1	1	1	hypothetical protein	*	*	*	*	09T408_13740	*	*	*	*	*	*	*
1	1	1	hypothetical protein	*	*	*	*	09T408_13750	*	*	*	*	*	*	*
1	1	1	hypothetical protein	*	*	*	*	09T408_13770	*	*	*	*	*	*	*
1	1	1	Divergent AAA domain protein	*	*	*	*	09T408_13890	*	*	*	*	*	*	*
1	1	1	hypothetical protein	*	*	*	*	09T408_13910	*	*	*	*	*	*	*
1	1	1	hypothetical protein	*	*	*	*	09T408_13920	*	*	*	*	*	*	*
1	1	1	hypothetical protein	*	*	*	*	09T408_13950	*	*	*	*	*	*	*
4	5	0.31	hypothetical protein	*	*	*	*	09T408_14100	09T494_15500	12T272_12500	*	12T306_16090,12T306_	*	*	*
4	4	1	putative DNA-binding transcriptional regulator	*	*	*	*	09T408_14130	09T494_15530	12T272_12530	*	12T306_16120	*	*	*
2	2	1	UDP-glucose 6-dehydrogenase	*	*	*	*	09T408_14360	*	12T272_14650	*	*	*	*	*
2	2	1	hypothetical protein	*	*	*	*	09T408_14370	*	12T272_14660	*	*	*	*	*
2	2	1	hypothetical protein	*	*	*	*	09T408_14380	*	12T272_14670	*	*	*	*	*
2	2	1	O-Antigen ligase	*	*	*	*	09T408_14390	*	12T272_14680	*	*	*	*	*
2	2	1	putative glycosyltransferase EpsJ	*	*	*	*	09T408_14400	*	12T272_14690	*	*	*	*	*
3	3	1	Capsule biosynthesis protein CapA	*	*	*	*	09T408_14410	09T494_17230	12T272_14700	*	*	*	*	*
3	4	0.36	hypothetical protein	*	*	*	*	09T408_14420	09T494_17240,09T494_	12T272_14710	*	*	*	*	*
2	2	1	alanine dehydrogenase	*	*	*	*	09T408_14430	*	12T272_14720	*	*	*	*	*
3	6	0.105	carbamoyl phosphate synthase-like protein	*	*	*	*	09T408_14440,09T408_	09T494_17300,09T494_	12T272_14730,12T272_	*	*	*	*	*
3	3	1	Transcriptional regulator LytR	*	*	*	*	09T408_14480	09T494_17380	12T272_14770	*	*	*	*	*
3	3	1	Bacterial SH3 domain protein	*	*	*	*	09T408_14490	09T494_17390	12T272_14780	*	*	*	*	*
3	3	1	Capsular polysaccharide type 8 biosynthesis pr	*	*	*	*	09T408_14520	09T494_17420	12T272_14810	*	*	*	*	*
1	1	1	hypothetical protein	*	*	*	*	09T408_14680	*	*	*	*	*	*	*
1	2	1	hypothetical protein	*	*	*	*	09T408_14840,09T408_	*	*	*	*	*	*	*
1	1	1	Transposase	*	*	*	*	09T408_14850	*	*	*	*	*	*	*
1	1	1	hypothetical protein	*	*	*	*	09T408_14860	*	*	*	*	*	*	*
1	1	1	hypothetical protein	*	*	*	*	09T408_14880	*	*	*	*	*	*	*
1	1	1	hypothetical protein	*	*	*	*	09T408_14950	*	*	*	*	*	*	*
4	4	1	GTP pyrophosphokinase	*	*	*	*	09T408_14960	*	12T272_15300	*	12T306_03060	*	*	T160124_14970
4	4	1	hypothetical protein	*	*	*	*	09T408_14970	*	12T272_15310	*	12T306_03050	*	*	T160124_14980

1	1	1	hypothetical protein	*	*	*	*	09T408_14980	*	*	*	*	*	*	*
3	3	1	hypothetical protein	*	*	*	*	09T408_14990	*	*	*	12T306_03040	*	*	T160124_15000
2	2	1	Internalin-A precursor	*	*	*	*	09T408_15230	*	*	*	12T306_16250	*	*	*
1	1	1	Peptostreptococcal albumin-binding protein pr	*	*	*	*	09T408_15360	*	*	*	*	*	*	*
1	1	1	Reverse transcriptase (RNA-dependent DNA pc	*	*	*	*	09T408_15510	*	*	*	*	*	*	*
8	9	0.195	N-acetylmuramoyl-L-alanine amidase LytC pre	*	*	*	*	09T408_15770	09T494_00570	12T272_16100	12T273_18550	12T306_16710,12T306_	CCUG54800_17990	T151023_15280	T160124_15820
2	2	1	hypothetical protein	*	*	*	*	09T408_15850	*	*	*	*	CCUG54800_18070	*	*
3	3	1	hypothetical protein	*	*	*	*	09T408_15900	*	12T272_13700	*	*	CCUG54800_18120	*	*
3	3	1	hypothetical protein	*	*	*	*	09T408_15910	*	12T272_13710	*	*	CCUG54800_18130	*	*
2	2	1	hypothetical protein	*	*	*	*	09T408_15920	*	*	*	*	CCUG54800_18140	*	*
2	2	1	putative siderophore transport system ATP-bir	*	*	*	*	09T408_15980	*	*	12T273_05950	*	*	*	*
1	2	1	putative siderophore transport system permea	*	*	*	*	09T408_15990,09T408_1	*	*	*	*	*	*	*
2	2	1	Fe(3+)-citrate-binding protein YfmC precursor	*	*	*	*	09T408_16010	*	*	12T273_05920	*	*	*	*
1	1	1	D-alanine--poly(phosphoribitol) ligase subunit	*	*	*	*	09T408_16040	*	*	*	*	*	*	*
1	1	1	Peptidoglycan O-acetyltransferase	*	*	*	*	09T408_16050	*	*	*	*	*	*	*
1	1	1	D-alanine--poly(phosphoribitol) ligase subunit	*	*	*	*	09T408_16060	*	*	*	*	*	*	*
1	1	1	hypothetical protein	*	*	*	*	09T408_16070	*	*	*	*	*	*	*
2	2	1	N-acetylmuramoyl-L-alanine amidase LytC pre	*	*	*	*	09T408_16910	*	*	*	12T306_00380	*	*	*
1	1	1	Phage tail protein	*	*	*	*	*	09T494_00010	*	*	*	*	*	*
1	1	1	Chromosome partition protein Smc	*	*	*	*	*	09T494_00020	*	*	*	*	*	*
1	1	1	hypothetical protein	*	*	*	*	*	09T494_00030	*	*	*	*	*	*
1	1	1	hypothetical protein	*	*	*	*	*	09T494_00040	*	*	*	*	*	*
1	1	1	hypothetical protein	*	*	*	*	*	09T494_00050	*	*	*	*	*	*
1	1	1	hypothetical protein	*	*	*	*	*	09T494_00060	*	*	*	*	*	*
1	1	1	hypothetical protein	*	*	*	*	*	09T494_00070	*	*	*	*	*	*
1	1	1	hypothetical protein	*	*	*	*	*	09T494_00080	*	*	*	*	*	*
1	1	1	Phage capsid family protein	*	*	*	*	*	09T494_00090	*	*	*	*	*	*
1	1	1	Phage portal protein	*	*	*	*	*	09T494_00110	*	*	*	*	*	*
1	1	1	Phage Terminase	*	*	*	*	*	09T494_00120	*	*	*	*	*	*
1	1	1	hypothetical protein	*	*	*	*	*	09T494_00130	*	*	*	*	*	*
1	1	1	hypothetical protein	*	*	*	*	*	09T494_00140	*	*	*	*	*	*
1	1	1	HNH endonuclease	*	*	*	*	*	09T494_00150	*	*	*	*	*	*
1	1	1	hypothetical protein	*	*	*	*	*	09T494_00160	*	*	*	*	*	*
1	1	1	hypothetical protein	*	*	*	*	*	09T494_00170	*	*	*	*	*	*
2	2	1	hypothetical protein	*	*	*	*	*	09T494_00180	*	*	*	CCUG54800_01020	*	*
1	1	1	hypothetical protein	*	*	*	*	*	09T494_00190	*	*	*	*	*	*
2	2	1	hypothetical protein	*	*	*	*	*	09T494_00200	*	*	*	CCUG54800_01010	*	*
1	1	1	hypothetical protein	*	*	*	*	*	09T494_00210	*	*	*	*	*	*
1	1	1	hypothetical protein	*	*	*	*	*	09T494_00220	*	*	*	*	*	*
2	2	1	hypothetical protein	*	*	*	*	*	09T494_00230	*	*	*	CCUG54800_00960	*	*
1	1	1	hypothetical protein	*	*	*	*	*	09T494_00240	*	*	*	*	*	*
2	2	1	hypothetical protein	*	*	*	*	*	09T494_00250	*	*	*	CCUG54800_00940	*	*
1	1	1	hypothetical protein	*	*	*	*	*	09T494_00260	*	*	*	*	*	*
1	1	1	Endodeoxyribonuclease RusA	*	*	*	*	*	09T494_00270	*	*	*	*	*	*
1	1	1	hypothetical protein	*	*	*	*	*	09T494_00280	*	*	*	*	*	*
1	1	1	hypothetical protein	*	*	*	*	*	09T494_00290	*	*	*	*	*	*
1	1	1	hypothetical protein	*	*	*	*	*	09T494_00300	*	*	*	*	*	*
1	1	1	hypothetical protein	*	*	*	*	*	09T494_00310	*	*	*	*	*	*
1	1	1	hypothetical protein	*	*	*	*	*	09T494_00330	*	*	*	*	*	*
1	1	1	hypothetical protein	*	*	*	*	*	09T494_00340	*	*	*	*	*	*
1	1	1	hypothetical protein	*	*	*	*	*	09T494_00350	*	*	*	*	*	*
1	1	1	hypothetical protein	*	*	*	*	*	09T494_00360	*	*	*	*	*	*
2	2	1	hypothetical protein	*	*	*	*	*	09T494_00370	*	*	*	CCUG54800_00860	*	*
1	1	1	hypothetical protein	*	*	*	*	*	09T494_00380	*	*	*	*	*	*
2	2	1	hypothetical protein	*	*	*	*	*	09T494_00390	*	*	*	CCUG54800_00850	*	*
1	1	1	hypothetical protein	*	*	*	*	*	09T494_00400	*	*	*	*	*	*
1	1	1	Helix-turn-helix	*	*	*	*	*	09T494_00410	*	*	*	*	*	*
1	1	1	hypothetical protein	*	*	*	*	*	09T494_00420	*	*	*	*	*	*
1	1	1	hypothetical protein	*	*	*	*	*	09T494_00430	*	*	*	*	*	*
1	1	1	hypothetical protein	*	*	*	*	*	09T494_00450	*	*	*	*	*	*
1	1	1	hypothetical protein	*	*	*	*	*	09T494_00480	*	*	*	*	*	*
1	1	1	site-specific tyrosine recombinase XerD	*	*	*	*	*	09T494_00490	*	*	*	*	*	*
1	1	1	potassium/proton antiporter	*	*	*	*	*	09T494_01590	*	*	*	*	*	*
6	6	1	N-acetylmuramoyl-L-alanine amidase LytC pre	*	*	*	*	*	09T494_01830	12T272_00080	*	12T306_00780	CCUG54800_00400	T151023_00870	T160124_00130
1	1	1	hypothetical protein	*	*	*	*	*	09T494_01910	*	*	*	*	*	*
1	1	1	Cna protein B-type domain protein	*	*	*	*	*	09T494_01920	*	*	*	*	*	*
1	1	1	hypothetical protein	*	*	*	*	*	09T494_01930	*	*	*	*	*	*
1	1	1	Transposon Tn7 transposition protein TnsC	*	*	*	*	*	09T494_01940	*	*	*	*	*	*
1	1	1	Transposon Tn7 transposition protein TnsB	*	*	*	*	*	09T494_01950	*	*	*	*	*	*
1	1	1	Transposon Tn7 transposition protein TnsA	*	*	*	*	*	09T494_01960	*	*	*	*	*	*
1	1	1	hypothetical protein	*	*	*	*	*	09T494_01970	*	*	*	*	*	*
1	1	1	hypothetical protein	*	*	*	*	*	09T494_01980	*	*	*	*	*	*
1	1	1	Type IIS restriction enzyme Eco57I	*	*	*	*	*	09T494_01990	*	*	*	*	*	*
1	1	1	Modification methylase Eco57IB	*	*	*	*	*	09T494_02000	*	*	*	*	*	*
1	1	1	Competence protein ComM	*	*	*	*	*	09T494_02030	*	*	*	*	*	*
2	2	1	hypothetical protein	*	*	*	*	*	09T494_02110	12T272_00250	*	*	*	*	*
2	2	1	Eco57I restriction-modification methylase	*	*	*	*	*	09T494_02120	12T272_00260	*	*	*	*	*
2	2	1	Type III restriction enzyme, res subunit	*	*	*	*	*	09T494_02130	12T272_00270	*	*	*	*	*
1	1	1	hypothetical protein	*	*	*	*	*	09T494_02310	*	*	*	*	*	*
1	1	1	hypothetical protein	*	*	*	*	*	09T494_02320	*	*	*	*	*	*
1	1	1	hypothetical protein	*	*	*	*	*	09T494_02440	*	*	*	*	*	*
1	1	1	hypothetical protein	*	*	*	*	*	09T494_02570	*	*	*	*	*	*
1	1	1	sensor kinase CusS	*	*	*	*	*	09T494_02640	*	*	*	*	*	*
6	6	1	UDP-N-acetyl-alpha-D-glucosamine C6 dehydra	*	*	*	*	*	09T494_02750	12T272_00970	12T273_02120	12T306_01530	*	T151023_01670	T160124_00850
1	1	1	hypothetical protein	*	*	*	*	*	09T494_02980	*	*	*	*	*	*
1	1	1	DNA polymerase IV	*	*	*	*	*	09T494_03760	*	*	*	*	*	*
1	1	1	DNA polymerase IV	*	*	*	*	*	09T494_03770	*	*	*	*	*	*
1	1	1	DNA polymerase IV	*	*	*	*	*	09T494_03780	*	*	*	*	*	*
2	2	1	hypothetical protein	*	*	*	*	*	09T494_03810	*	*	12T306_02560	*	*	*
2	3	1	hypothetical protein	*	*	*	*	*	09T494_04060	12T272_01780,12T272_	*	*	*	*	*
2	3	1	Peptidase family M50	*	*	*	*	*	09T494_04070	*	*	*	CCUG54800_04220,CCUG548	*	*
4	4	1	hypothetical protein	*	*	*	*	*	09T494_04080	12T272_01810	*	*	CCUG54800_04230	T151023_03560	*
7	7	1	ribosomal-protein-alanine N-acetyltransferase	*	*	*	*	*	09T494_05170	12T272_02970	12T273_07910	12T306_06070	CCUG54800_05320	T151023_04680	T160124_04370
1	2	1	dTDP-3-amino-3,6-dideoxy-alpha-D-galactopyr	*	*	*	*	*	09T494_06690,09T494_	*	*	*	*	*	*
2	2	1	hypothetical protein	*	*	*	*	*	09T494_07300	*	*	*	*	*	T160124_06560
4	4	1	hypothetical protein	*	*	*	*	*	09T494_09070	12T272_06830	*	12T306_09940	CCUG54800_09170	*	*
2	2	1	hypothetical protein	*	*	*	*	*	09T494_10130	*	*	*	CCUG54800_13250	*	*
1	1	1	hypothetical protein	*	*	*	*	*	09T494_10920	*	*	*	*	*	*
1	1	1	hypothetical protein	*	*	*	*	*	09T494_11010	*	*	*	*	*	*
1	1	1	hypothetical protein	*	*	*	*	*	09T494_11030	*	*	*	*	*	*
1	1	1	hypothetical protein	*	*	*	*	*	09T494_11040	*	*	*	*	*	*
3	3	1	hypothetical protein	*	*	*	*	*	09T494_11120	12T272_12960	*	*	*	T151023_02720	*
3	3	1	hypothetical protein	*	*	*	*	*	09T494_11130	12T272_12970	*	*	*	T151023_02710	*
1	1	1	Transposase IS200 like protein	*	*	*	*	*	09T494_11430	*	*	*	*	*	*
1	1	1	SigmaK-factor processing regulatory protein Bc	*	*	*	*	*	09T494_12110	*	*	*	*	*	*
1	1	1	hypothetical protein	*	*	*	*	*	09T494_12670	*	*	*	*	*	*
1	1	1	putative ABC transporter ATP-binding protein	*	*	*	*	*	09T494_12680	*	*	*	*	*	*
2	3	1	hypothetical protein	*	*	*	*	*	09T494_12710	*	12T273_15660,12T273_	*	*	*	*
3	3	1	NADP-reducing hydrogenase subunit HndC	*	*	*	*	*	09T494_13460	12T272_10960	*	*	*	T151023_12310	*
3	3	1	Biotin synthase	*	*	*	*	*	09T494_13490	12T272_10990	*	*	*	T151023_12340	*
3	3	1	tRNA modification GTPase MnmE	*	*	*	*	*	09T494_13510	12T272_11010	*	*	*	T151023_12360	*
2	2	1	hypothetical protein	*	*	*	*	*	09T494_13590	12T272_11100	*	*	*	*	*
5	5	1	putative ABC transporter ATP-binding protein	*	*	*	*	*	09T494_13720	12T272_12860	12T273_13390	*	CCUG54800_15580	T151023_02850	*
6	6	0.333	Putative multidrug export ATP-binding/permea	*	*	*	*	*	09T494_13730	12T272_12870	12T273_13400	*	CCUG54800_15590	T151023_02840	T160124_13860
3	3	1	Energy-coupling factor transporter transmemb	*	*	*	*	*	09T494_13750	12T272_12890	12T273_13420	*	*	*	*
3	3	1	hypothetical protein	*	*	*	*	*	09T494_13770	12T272_12910	12T273_13440	*	*	*	*
1	1	1	Alpha/beta hydrolase family protein	*	*	*	*	*	09T494_13780	*	*	*	*	*	*
1	1	1	Polyketide synthase PksR	*	*	*	*	*	09T494_13790	*	*	*	*	*	*
1	1	1	hypothetical protein	*	*	*	*	*	09T494_13800	*	*	*	*	*	*
2	2	1	hypothetical protein	*	*	*	*	*	09T494_14000	*	*	*	CCUG54800_10650	*	*
1	1	1	putative multidrug resistance ABC transporter	*	*	*	*	*	09T494_14160	*	*	*	*	*	*
1	1	1	hypothetical protein	*	*	*	*	*	09T494_14320	*	*	*	*	*	*
1	1	1	hypothetical protein	*	*	*	*	*	09T494_14570	*	*	*	*	*	*
1	1	1	hypothetical protein	*	*	*	*	*	09T494_14590	*	*	*	*	*	*
2	2	1	hypothetical protein	*	*	*	*	*	09T494_14610	*	*	12T306_17040	*	*	*
1	1	1	hypothetical protein	*	*	*	*	*	09T494_14620	*	*	*	*	*	*
1	1	1	Sugar-specific transcriptional regulator TrmB	*	*	*	*	*	09T494_14630	*	*	*	*	*	*
1	1	1	hypothetical protein	*	*	*	*	*	09T494_14640	*	*	*	*	*	*
1	1	1	Spermidine N(1)-acetyltransferase	*	*	*	*	*	09T494_14660	*	*	*	*	*	*
1	1	1	hypothetical protein	*	*	*	*	*	09T494_14670	*	*	*	*	*	*
3	3	1	Iron import ATP-binding/permease protein Irt/	*	*	*	*	*	09T494_15520	12T272_12520	*	12T306_16110	*	*	*
2	2	1	DNA alkylation repair enzyme	*	*	*	*	*	09T494_15540	12T272_12540	*	*	*	*	*
2	2	1	Phage Terminase	*	*	*	*	*	09T494_15550	12T272_12550	*	*	*	*	*
1	1	1	Transposase	*	*	*	*	*	09T494_15560	*	*	*	*	*	*
4	6	0.461	hypothetical protein	*	*	*	*	*	09T494_15580,09T494_	12T272_12850,12T272_	*	12T306_16060	*	T151023_02870	*
1	1	1	hypothetical protein	*	*	*	*	*	09T494_15660	*	*	*	*	*	*
1	1	1	hypothetical protein	*	*	*	*	*	09T494_15670	*	*	*	*	*	*
2	2	1	flavodoxin	*	*	*	*	*	09T494_15690	12T272_12750	*	*	*	*	*
2	2	1	Tropinesterase	*	*	*	*	*	09T494_15700	12T272_12760	*	*	*	*	*
2	2	1	SpoVT / AbrB like domain protein	*	*	*	*	*	09T494_15710	12T272_12770	*	*	*	*	*
2	2	1	hypothetical protein	*	*	*	*	*	09T494_15720	12T272_12780	*	*	*	*	*
2	2	1	hypothetical protein	*	*	*	*	*	09T494_15730	12T272_12790	*	*	*	*	*
1	1	1	hypothetical protein	*	*	*	*	*	09T494_15740	*	*	*	*	*	*
1	1	1	hypothetical protein	*	*	*	*	*	09T494_15750	*	*	*	*	*	*
2	2	1	hypothetical protein	*	*	*	*	*	09T494_15760	12T272_12810	*	*	*	*	*
1	1	1	hypothetical protein	*	*	*	*	*	09T494_15770	*	*	*	*	*	*
1	1	1	hypothetical protein	*	*	*	*	*	09T494_15780	*	*	*	*	*	*
2	2	1	hypothetical protein	*	*	*	*	*	09T494_15820	*	*	*	CCUG54800_16800	*	*
2	2	1	hypothetical protein	*	*	*	*	*	09T494_15850	*	*	*	CCUG54800_16830	*	*
1	1	1	Transposase, Mutator family	*	*	*	*	*	09T494_15900	*	*	*	*	*	*
1	1	1	hypothetical protein	*	*	*	*	*	09T494_15930	*	*	*	*	*	*
1	1	1	molybdenum cofactor biosynthesis protein A	*	*	*	*	*	09T494_15940	*	*	*	*	*	*
1	2	1	hypothetical protein	*	*	*	*	*	09T494_15950,09T494_	*	*	*	*	*	*
1	1	1	hypothetical protein	*	*	*	*	*	09T494_15960	*	*	*	*	*	*
1	1	1	Putative multidrug export ATP-binding/permea	*	*	*	*	*	09T494_15970	*	*	*	*	*	*
1	1	1	FtsX-like permease family protein	*	*	*	*	*	09T494_15990	*	*	*	*	*	*
1	1	1	antitoxin HipB	*	*	*	*	*	09T494_16010	*	*	*	*	*	*
1	1	1	Peptidase family M50	*	*	*	*	*	09T494_16020	*	*	*	*	*	*
1	1	1	hypothetical protein	*	*	*	*	*	09T494_16030	*	*	*	*	*	*
1	1	1	hypothetical protein	*	*	*	*	*	09T494_16040	*	*	*	*	*	*
3	5	1	Integrase core domain protein	*	*	*	*	*	09T494_16070	*	*	*	CCUG54800_07050,CCUG548	*	T160124_17070
1	1	1	ABC-2 family transporter protein	*	*	*	*	*	09T494_16080	*	*	*	*	*	*
1	1	1	ABC-2 family transporter protein	*	*	*	*	*	09T494_16090	*	*	*	*	*	*
1	1	1	putative ABC transporter ATP-binding protein \	*	*	*	*	*	09T494_16100	*	*	*	*	*	*
1	1	1	hypothetical protein	*	*	*	*	*	09T494_16120	*	*	*	*	*	*
1	1	1	hypothetical protein	*	*	*	*	*	09T494_16130	*	*	*	*	*	*
1	1	1	hypothetical protein	*	*	*	*	*	09T494_16140	*	*	*	*	*	*
1	1	1	Chromosome partition protein Smc	*	*	*	*	*	09T494_16290	*	*	*	*	*	*
4	4	0.5	hypothetical protein	*	*	*	*	*	09T494_16350	*	*	12T306_04730	CCUG54800_03930	T151023_03300	*
1	1	1	hypothetical protein	*	*	*	*	*	09T494_16770	*	*	*	*	*	*
2	2	1	Argininosuccinate lyase 1	*	*	*	*	*	09T494_16940	12T272_14370	*	*	*	*	*
2	2	1	Argininosuccinate synthase	*	*	*	*	*	09T494_16950	12T272_14380	*	*	*	*	*
1	1	1	hypothetical protein	*	*	*	*	*	09T494_17000	*	*	*	*	*	*
1	1	1	hypothetical protein	*	*	*	*	*	09T494_17040	*	*	*	*	*	*
2	2	1	hypothetical protein	*	*	*	*	*	09T494_17050	*	*	*	*	*	T160124_13480
1	1	1	hypothetical protein	*	*	*	*	*	09T494_17060	*	*	*	*	*	*
1	1	1	HNH endonuclease	*	*	*	*	*	09T494_17100	*	*	*	*	*	*
2	2	1	Acetyltransferase (GNAT) family protein	*	*	*	*	*	09T494_17180	12T272_14620	*	*	*	*	*
2	2	1	RNA polymerase sigma factor	*	*	*	*	*	09T494_17190	12T272_14630	*	*	*	*	*
1	1	1	O-Antigen ligase	*	*	*	*	*	09T494_17210	*	*	*	*	*	*
1	1	1	Polysaccharide biosynthesis protein	*	*	*	*	*	09T494_17220	*	*	*	*	*	*
1	1	1	Capsule biosynthesis protein CapA	*	*	*	*	*	09T494_17250	*	*	*	*	*	*
1	1	1	N(5)-(carboxyethyl)ornithine synthase	*	*	*	*	*	09T494_17270	*	*	*	*	*	*
1	1	1	hypothetical protein	*	*	*	*	*	09T494_17280	*	*	*	*	*	*
1	1	1	Acetyltransferase (GNAT) family protein	*	*	*	*	*	09T494_17290	*	*	*	*	*	*
1	1	1	hypothetical protein	*	*	*	*	*	09T494_17320	*	*	*	*	*	*
1	1	1	hypothetical protein	*	*	*	*	*	09T494_17330	*	*	*	*	*	*
1	1	1	dTDP-glucose 4,6-dehydratase 2	*	*	*	*	*	09T494_17360	*	*	*	*	*	*
1	1	1	Peptostreptococcal albumin-binding protein pr	*	*	*	*	*	09T494_18200	*	*	*	*	*	*
1	1	1	hypothetical protein	*	*	*	*	*	09T494_18210	*	*	*	*	*	*
1	1	1	Inner membrane transport protein YajR	*	*	*	*	*	09T494_18240	*	*	*	*	*	*
1	1	1	hypothetical protein	*	*	*	*	*	09T494_18250	*	*	*	*	*	*
2	2	1	Inner membrane transporter YjeM	*	*	*	*	*	09T494_18370	*	*	*	*	T151023_15150	*
1	1	1	hypothetical protein	*	*	*	*	*	09T494_18520	*	*	*	*	*	*
1	1	1	hypothetical protein	*	*	*	*	*	09T494_18550	*	*	*	*	*	*
1	1	1	AP2 domain protein	*	*	*	*	*	09T494_18560	*	*	*	*	*	*
1	1	1	hypothetical protein	*	*	*	*	*	09T494_18570	*	*	*	*	*	*
2	2	1	hypothetical protein	*	*	*	*	*	09T494_18590	*	*	*	CCUG54800_01400	*	*
1	1	1	N-acetylmuramoyl-L-alanine amidase	*	*	*	*	*	09T494_18600	*	*	*	*	*	*
2	2	1	hypothetical protein	*	*	*	*	*	09T494_18610	*	*	*	CCUG54800_01380	*	*
1	1	1	hypothetical protein	*	*	*	*	*	09T494_18620	*	*	*	*	*	*
1	1	1	hypothetical protein	*	*	*	*	*	09T494_18630	*	*	*	*	*	*
1	1	1	hypothetical protein	*	*	*	*	*	09T494_18640	*	*	*	*	*	*
1	1	1	Nucleopolyhedrovirus P10 protein	*	*	*	*	*	09T494_18650	*	*	*	*	*	*
1	1	1	Putative acetyltransferase	*	*	*	*	*	*	12T272_00170	*	*	*	*	*
1	1	1	hypothetical protein	*	*	*	*	*	*	12T272_00220	*	*	*	*	*
1	1	1	hypothetical protein	*	*	*	*	*	*	12T272_00450	*	*	*	*	*
1	1	1	hypothetical protein	*	*	*	*	*	*	12T272_00500	*	*	*	*	*
2	2	1	Tyrosine recombinase XerD	*	*	*	*	*	*	12T272_00640	*	12T306_04390	*	*	*
1	1	1	hypothetical protein	*	*	*	*	*	*	12T272_00650	*	*	*	*	*

1	1	1	HTH-type transcriptional regulator SinR	*	*	*	*	*	*	12T272_00660	*	*	*	*	*
1	1	1	Helix-turn-helix	*	*	*	*	*	*	12T272_00670	*	*	*	*	*
1	1	1	ICEBs1 excisionase	*	*	*	*	*	*	12T272_00680	*	*	*	*	*
1	1	1	hypothetical protein	*	*	*	*	*	*	12T272_00690	*	*	*	*	*
1	1	1	hypothetical protein	*	*	*	*	*	*	12T272_00700	*	*	*	*	*
1	1	1	hypothetical protein	*	*	*	*	*	*	12T272_00710	*	*	*	*	*
1	1	1	hypothetical protein	*	*	*	*	*	*	12T272_00720	*	*	*	*	*
1	1	1	hypothetical protein	*	*	*	*	*	*	12T272_00730	*	*	*	*	*
1	1	1	hypothetical protein	*	*	*	*	*	*	12T272_00740	*	*	*	*	*
1	1	1	RNA polymerase sigma factor WhiG	*	*	*	*	*	*	12T272_00750	*	*	*	*	*
1	1	1	Major Facilitator Superfamily protein	*	*	*	*	*	*	12T272_00980	*	*	*	*	*
1	1	1	enterobactin exporter EntS	*	*	*	*	*	*	12T272_00990	*	*	*	*	*
3	3	1	D-alanyl-D-alanine-carboxypeptidase/endopep	*	*	*	*	*	*	12T272_01520	*	*	CCUG54800_02260	T151023_02110	*
1	1	1	hypothetical protein	*	*	*	*	*	*	12T272_01610	*	*	*	*	*
1	1	1	hypothetical protein	*	*	*	*	*	*	12T272_01790	*	*	*	*	*
1	1	1	putative HTH-type transcriptional regulator Yb	*	*	*	*	*	*	12T272_02030	*	*	*	*	*
1	1	1	Spermidine/putrescine import ATP-binding pro	*	*	*	*	*	*	12T272_02040	*	*	*	*	*
1	1	1	hypothetical protein	*	*	*	*	*	*	12T272_02060	*	*	*	*	*
1	1	1	2-aminoethylphosphonate--pyruvate transami	*	*	*	*	*	*	12T272_02070	*	*	*	*	*
1	1	1	Phosphonoacetaldehyde hydrolase	*	*	*	*	*	*	12T272_02080	*	*	*	*	*
1	1	1	hypothetical protein	*	*	*	*	*	*	12T272_02110	*	*	*	*	*
1	1	1	hypothetical protein	*	*	*	*	*	*	12T272_08030	*	*	*	*	*
1	1	1	hypothetical protein	*	*	*	*	*	*	12T272_08040	*	*	*	*	*
1	1	1	hypothetical protein	*	*	*	*	*	*	12T272_08280	*	*	*	*	*
1	1	1	hypothetical protein	*	*	*	*	*	*	12T272_08540	*	*	*	*	*
1	1	1	hypothetical protein	*	*	*	*	*	*	12T272_08730	*	*	*	*	*
1	1	1	hypothetical protein	*	*	*	*	*	*	12T272_08970	*	*	*	*	*
1	1	1	PglZ domain protein	*	*	*	*	*	*	12T272_09340	*	*	*	*	*
1	1	1	N-6 DNA Methylase	*	*	*	*	*	*	12T272_09350	*	*	*	*	*
1	1	1	hypothetical protein	*	*	*	*	*	*	12T272_09360	*	*	*	*	*
1	1	1	hypothetical protein	*	*	*	*	*	*	12T272_09370	*	*	*	*	*
1	1	1	hypothetical protein	*	*	*	*	*	*	12T272_09380	*	*	*	*	*
2	2	1	hypothetical protein	*	*	*	*	*	*	12T272_09770	*	*	*	*	T160124_11230
1	1	1	Chaperone protein DnaJ	*	*	*	*	*	*	12T272_10050	*	*	*	*	*
1	1	1	hypothetical protein	*	*	*	*	*	*	12T272_10840	*	*	*	*	*
2	2	1	ABC transporter transmembrane region	*	*	*	*	*	*	12T272_11090	*	*	*	*	T160124_12700
1	1	1	hypothetical protein	*	*	*	*	*	*	12T272_11160	*	*	*	*	*
1	1	1	MobA/MobL family protein	*	*	*	*	*	*	12T272_11580	*	*	*	*	*
1	1	1	HTH-type transcriptional regulator ImmR	*	*	*	*	*	*	12T272_11600	*	*	*	*	*
1	1	1	putative ABC transporter ATP-binding protein Y	*	*	*	*	*	*	12T272_12120	*	*	*	*	*
1	1	1	hypothetical protein	*	*	*	*	*	*	12T272_12130	*	*	*	*	*
1	1	1	hypothetical protein	*	*	*	*	*	*	12T272_12140	*	*	*	*	*
1	1	1	hypothetical protein	*	*	*	*	*	*	12T272_12150	*	*	*	*	*
1	1	1	hypothetical protein	*	*	*	*	*	*	12T272_12270	*	*	*	*	*
1	1	1	Transposase	*	*	*	*	*	*	12T272_12560	*	*	*	*	*
1	1	1	hypothetical protein	*	*	*	*	*	*	12T272_12570	*	*	*	*	*
2	3	1	hypothetical protein	*	*	*	*	*	*	12T272_12680	*	12T306_11160,12T306_	*	*	*
1	1	1	ABC-2 family transporter protein	*	*	*	*	*	*	12T272_12690	*	*	*	*	*
1	1	1	Lipopolysaccharide export system ATP-binding	*	*	*	*	*	*	12T272_12700	*	*	*	*	*
1	1	1	hypothetical protein	*	*	*	*	*	*	12T272_12710	*	*	*	*	*
1	1	1	hypothetical protein	*	*	*	*	*	*	12T272_12720	*	*	*	*	*
1	1	1	hypothetical protein	*	*	*	*	*	*	12T272_12730	*	*	*	*	*
1	1	1	hypothetical protein	*	*	*	*	*	*	12T272_12800	*	*	*	*	*
2	2	1	hypothetical protein	*	*	*	*	*	*	12T272_12820	12T273_13240	*	*	*	*
1	1	1	hypothetical protein	*	*	*	*	*	*	12T272_12930	*	*	*	*	*
1	1	1	Eco57I restriction-modification methylase	*	*	*	*	*	*	12T272_12940	*	*	*	*	*
1	1	1	hypothetical protein	*	*	*	*	*	*	12T272_13280	*	*	*	*	*
1	1	1	hypothetical protein	*	*	*	*	*	*	12T272_13680	*	*	*	*	*
1	1	1	hypothetical protein	*	*	*	*	*	*	12T272_13730	*	*	*	*	*
2	2	1	VanZ like family protein	*	*	*	*	*	*	12T272_13830	*	12T306_14790	*	*	*
1	1	1	Cna protein B-type domain protein	*	*	*	*	*	*	12T272_13990	*	*	*	*	*
2	2	1	hypothetical protein	*	*	*	*	*	*	12T272_14000	*	*	*	T151023_14150	*
1	1	1	hypothetical protein	*	*	*	*	*	*	12T272_14170	*	*	*	*	*
1	1	1	GIY-YIG catalytic domain protein	*	*	*	*	*	*	12T272_14450	*	*	*	*	*
1	1	1	hypothetical protein	*	*	*	*	*	*	12T272_14460	*	*	*	*	*
1	1	1	hypothetical protein	*	*	*	*	*	*	12T272_14480	*	*	*	*	*
1	1	1	hypothetical protein	*	*	*	*	*	*	12T272_14500	*	*	*	*	*
1	1	1	hypothetical protein	*	*	*	*	*	*	12T272_14530	*	*	*	*	*
1	1	1	hypothetical protein	*	*	*	*	*	*	12T272_14540	*	*	*	*	*
1	1	1	hypothetical protein	*	*	*	*	*	*	12T272_14550	*	*	*	*	*
1	1	1	hypothetical protein	*	*	*	*	*	*	12T272_14640	*	*	*	*	*
1	1	1	hypothetical protein	*	*	*	*	*	*	12T272_14820	*	*	*	*	*
1	1	1	putative cardiolipin synthase YwiE	*	*	*	*	*	*	12T272_14930	*	*	*	*	*
1	1	1	ABC transporter transmembrane region	*	*	*	*	*	*	12T272_15060	*	*	*	*	*
1	1	1	NYN domain protein	*	*	*	*	*	*	12T272_15220	*	*	*	*	*
1	1	1	hypothetical protein	*	*	*	*	*	*	12T272_15280	*	*	*	*	*
4	4	1	CRISPR-associated protein (Cas_Cas5)	*	*	*	*	*	*	12T272_15360	*	12T306_02990	*	T151023_14680	T160124_15050
4	4	1	CRISPR-associated protein (Cas_CXXC_CXXC)	*	*	*	*	*	*	12T272_15380	*	12T306_02970	*	T151023_14700	T160124_15070
1	1	1	Reverse transcriptase (RNA-dependent DNA po	*	*	*	*	*	*	12T272_15710	*	*	*	*	*
1	1	1	hypothetical protein	*	*	*	*	*	*	12T272_15720	*	*	*	*	*
1	1	1	Heterocyst differentiation ATP-binding protein	*	*	*	*	*	*	12T272_15960	*	*	*	*	*
1	1	1	ABC transporter transmembrane region	*	*	*	*	*	*	12T272_15970	*	*	*	*	*
1	1	1	hypothetical protein	*	*	*	*	*	*	12T272_15980	*	*	*	*	*
2	2	1	Toxin RTX-I translocation ATP-binding protein	*	*	*	*	*	*	12T272_16310	*	*	*	T151023_15620	*
1	1	1	hypothetical protein	*	*	*	*	*	*	12T272_16400	*	*	*	*	*
1	1	1	hypothetical protein	*	*	*	*	*	*	12T272_16410	*	*	*	*	*
1	1	1	Sodium:neurotransmitter symporter family pro	*	*	*	*	*	*	12T272_16660	*	*	*	*	*
3	3	1	Amidohydrolase	*	*	*	*	*	*	12T272_16720	12T273_00450	12T306_17340	*	*	*
3	3	1	L-fucose-proton symporter	*	*	*	*	*	*	12T272_16730	12T273_00460	12T306_17350	*	*	*
3	3	1	(2R)-sulfolactate sulfo-lyase subunit beta	*	*	*	*	*	*	12T272_16750	12T273_00480	12T306_17370	*	*	*
3	3	1	Altronate dehydratase	*	*	*	*	*	*	12T272_16760	12T273_00490	12T306_17380	*	*	*
3	3	1	putative 2-keto-3-deoxy-galactonate aldolase Y	*	*	*	*	*	*	12T272_16770	12T273_00500	12T306_17390	*	*	*
3	3	1	HTH-type transcriptional regulator SrpS	*	*	*	*	*	*	12T272_16780	12T273_00510	12T306_17400	*	*	*
1	1	1	Alpha/beta hydrolase family protein	*	*	*	*	*	*	12T272_17080	*	*	*	*	*
2	2	1	C factor cell-cell signaling protein	*	*	*	*	*	*	12T272_17090	*	*	CCUG54800_15650	*	*
1	1	1	Phenyloxazoline synthase MbtB	*	*	*	*	*	*	12T272_17110	*	*	*	*	*
1	1	1	hypothetical protein	*	*	*	*	*	*	12T272_17180	*	*	*	*	*
2	2	1	Lactococcin-G-processing and transport ATP-bi	*	*	*	*	*	*	12T272_17190	*	*	CCUG54800_10720	*	*
2	2	1	Lanthionine synthetase C-like protein	*	*	*	*	*	*	12T272_17200	*	*	CCUG54800_10730	*	*
1	1	1	hypothetical protein	*	*	*	*	*	*	12T272_17210	*	*	*	*	*
1	1	1	hypothetical protein	*	*	*	*	*	*	12T272_17220	*	*	*	*	*
1	1	1	ABC-2 family transporter protein	*	*	*	*	*	*	12T272_17230	*	*	*	*	*
1	1	1	ABC-2 family transporter protein	*	*	*	*	*	*	12T272_17240	*	*	*	*	*
1	1	1	hypothetical protein	*	*	*	*	*	*	12T272_17270	*	*	*	*	*
1	1	1	hypothetical protein	*	*	*	*	*	*	12T272_17280	*	*	*	*	*
5	5	1	hypothetical protein	*	*	*	*	*	*	12T272_18160	12T273_01170	*	CCUG54800_00270	T151023_00740	T160124_17000
1	2	1	hypothetical protein	*	*	*	*	*	*	*	12T273_00070,12T273_	*	*	*	*
2	2	1	hypothetical protein	*	*	*	*	*	*	*	12T273_00250	*	*	*	T160124_16260
1	1	1	hypothetical protein	*	*	*	*	*	*	*	12T273_00360	*	*	*	*
2	2	1	hypothetical protein	*	*	*	*	*	*	*	12T273_01200	*	*	*	T160124_00010
4	4	1	Divergent AAA domain protein	*	*	*	*	*	*	*	12T273_01390	12T306_11280	*	T151023_00970	T160124_00210
3	3	1	hypothetical protein	*	*	*	*	*	*	*	12T273_01410	*	*	T151023_00990	T160124_00240
1	2	1	Helix-turn-helix	*	*	*	*	*	*	*	12T273_01510,12T273_	*	*	*	*
1	2	1	Transcriptional regulatory protein CseB	*	*	*	*	*	*	*	12T273_01520,12T273_	*	*	*	*
1	2	1	Sensor histidine kinase YycG	*	*	*	*	*	*	*	12T273_01530,12T273_	*	*	*	*
1	1	1	hypothetical protein	*	*	*	*	*	*	*	12T273_01540	*	*	*	*
1	2	1	putative ABC transporter ATP-binding protein Y	*	*	*	*	*	*	*	12T273_01560,12T273_	*	*	*	*
1	2	1	ABC-2 family transporter protein	*	*	*	*	*	*	*	12T273_01570,12T273_	*	*	*	*
1	1	1	CAAX amino terminal protease self- immunity	*	*	*	*	*	*	*	12T273_01580	*	*	*	*
1	1	1	hypothetical protein	*	*	*	*	*	*	*	12T273_01590	*	*	*	*
1	1	1	hypothetical protein	*	*	*	*	*	*	*	12T273_01740	*	*	*	*
1	1	1	hypothetical protein	*	*	*	*	*	*	*	12T273_01970	*	*	*	*
2	2	1	Phosphate import ATP-binding protein PstB 3	*	*	*	*	*	*	*	12T273_01980	*	*	*	T160124_00710
1	1	1	hypothetical protein	*	*	*	*	*	*	*	12T273_02230	*	*	*	*
1	1	1	hypothetical protein	*	*	*	*	*	*	*	12T273_02260	*	*	*	*
1	1	1	Adenosine monophosphate-protein transferas	*	*	*	*	*	*	*	12T273_02840	*	*	*	*
1	1	1	Type I restriction modification DNA specificity	*	*	*	*	*	*	*	12T273_03120	*	*	*	*
1	1	1	Double zinc ribbon	*	*	*	*	*	*	*	12T273_03280	*	*	*	*
1	1	1	transcriptional repressor DicA	*	*	*	*	*	*	*	12T273_03290	*	*	*	*
1	1	1	hypothetical protein	*	*	*	*	*	*	*	12T273_03300	*	*	*	*
1	1	1	positive control sigma-like factor	*	*	*	*	*	*	*	12T273_03310	*	*	*	*
1	1	1	hypothetical protein	*	*	*	*	*	*	*	12T273_03320	*	*	*	*
2	2	1	DNA polymerase I, thermostable	*	*	*	*	*	*	*	12T273_03370	*	CCUG54800_16480	*	*
1	1	1	AP2 domain protein	*	*	*	*	*	*	*	12T273_03490	*	*	*	*
2	2	1	putative BsuMI modification methylase subuni	*	*	*	*	*	*	*	12T273_03500	*	CCUG54800_16340	*	*
2	2	1	hypothetical protein	*	*	*	*	*	*	*	12T273_03520	*	CCUG54800_16320	*	*
2	2	1	hypothetical protein	*	*	*	*	*	*	*	12T273_03530	*	CCUG54800_16310	*	*
2	2	1	hypothetical protein	*	*	*	*	*	*	*	12T273_03630	*	CCUG54800_16210	*	*
2	2	1	mRNA interferase RelE	*	*	*	*	*	*	*	12T273_03660	*	CCUG54800_16170	*	*
2	2	1	Phd_YefM	*	*	*	*	*	*	*	12T273_03670	*	CCUG54800_16160	*	*
3	3	1	Transposon Tn10 TetD protein	*	*	*	*	*	*	*	12T273_03930	12T306_15930	CCUG54800_15950	*	*
2	2	1	FRG domain protein	*	*	*	*	*	*	*	12T273_03950	12T306_02760	*	*	*
1	1	1	hypothetical protein	*	*	*	*	*	*	*	12T273_04000	*	*	*	*
1	1	1	Negative regulatory protein YxlE	*	*	*	*	*	*	*	12T273_04010	*	*	*	*
1	1	1	putative ABC transporter ATP-binding protein Y	*	*	*	*	*	*	*	12T273_04020	*	*	*	*
2	2	1	putative transmembrane protein YxlG	*	*	*	*	*	*	*	12T273_04030	*	CCUG54800_19200	*	*
1	1	1	transcriptional repressor DicA	*	*	*	*	*	*	*	12T273_04040	*	*	*	*
1	1	1	KAP family P-loop domain protein	*	*	*	*	*	*	*	12T273_04060	*	*	*	*
4	4	1	hypothetical protein	*	*	*	*	*	*	*	12T273_04320	*	CCUG54800_17260	T151023_14760	T160124_15140
1	1	1	hypothetical protein	*	*	*	*	*	*	*	12T273_04400	*	*	*	*
1	1	1	site-specific tyrosine recombinase XerC	*	*	*	*	*	*	*	12T273_04450	*	*	*	*
1	1	1	Helix-turn-helix domain protein	*	*	*	*	*	*	*	12T273_04460	*	*	*	*
1	1	1	RNA helicase	*	*	*	*	*	*	*	12T273_04470	*	*	*	*
1	1	1	hypothetical protein	*	*	*	*	*	*	*	12T273_04480	*	*	*	*
1	1	1	hypothetical protein	*	*	*	*	*	*	*	12T273_04490	*	*	*	*
1	1	1	hypothetical protein	*	*	*	*	*	*	*	12T273_04500	*	*	*	*
1	1	1	hypothetical protein	*	*	*	*	*	*	*	12T273_04510	*	*	*	*
1	1	1	hypothetical protein	*	*	*	*	*	*	*	12T273_04520	*	*	*	*
1	1	1	hypothetical protein	*	*	*	*	*	*	*	12T273_04530	*	*	*	*
1	1	1	Chaperone protein DnaJ	*	*	*	*	*	*	*	12T273_04550	*	*	*	*
1	1	1	Cobalt import ATP-binding protein CbiO	*	*	*	*	*	*	*	12T273_04850	*	*	*	*
1	1	1	hypothetical protein	*	*	*	*	*	*	*	12T273_05860	*	*	*	*
1	1	1	Transposase	*	*	*	*	*	*	*	12T273_05910	*	*	*	*
1	1	1	Ferric enterobactin transport system permease	*	*	*	*	*	*	*	12T273_05930	*	*	*	*
1	1	1	putative siderophore transport system permea	*	*	*	*	*	*	*	12T273_05940	*	*	*	*
1	1	1	Restriction enzyme BgcI subunit beta	*	*	*	*	*	*	*	12T273_05960	*	*	*	*
1	1	1	hypothetical protein	*	*	*	*	*	*	*	12T273_05970	*	*	*	*
3	3	1	Flavoredoxin	*	*	*	*	*	*	*	12T273_06100	12T306_14820	*	*	T160124_02660
2	2	1	hypothetical protein	*	*	*	*	*	*	*	12T273_06340	*	CCUG54800_03770	*	*
2	2	1	hypothetical protein	*	*	*	*	*	*	*	12T273_06350	*	CCUG54800_03780	*	*
1	1	1	2-keto-3-deoxygluconate permease	*	*	*	*	*	*	*	12T273_06470	*	*	*	*
1	1	1	hypothetical protein	*	*	*	*	*	*	*	12T273_06540	*	*	*	*
1	1	1	hypothetical protein	*	*	*	*	*	*	*	12T273_06700	*	*	*	*
2	2	1	hypothetical protein	*	*	*	*	*	*	*	12T273_07160	*	CCUG54800_04560	*	*
1	1	1	hypothetical protein	*	*	*	*	*	*	*	12T273_08300	*	*	*	*
3	3	1	Sortase family protein	*	*	*	*	*	*	*	12T273_08310	*	*	T151023_15980	T160124_04840
1	1	1	hypothetical protein	*	*	*	*	*	*	*	12T273_10620	*	*	*	*
1	1	1	hypothetical protein	*	*	*	*	*	*	*	12T273_10830	*	*	*	*
1	1	1	glycerol-3-phosphate transporter periplasmic I	*	*	*	*	*	*	*	12T273_12670	*	*	*	*
1	1	1	hypothetical protein	*	*	*	*	*	*	*	12T273_13020	*	*	*	*
1	1	1	HTH-type transcriptional activator Btr	*	*	*	*	*	*	*	12T273_13030	*	*	*	*
2	2	1	hypothetical protein	*	*	*	*	*	*	*	12T273_13070	*	CCUG54800_10500	*	*
2	2	1	hypothetical protein	*	*	*	*	*	*	*	12T273_13100	*	CCUG54800_10520	*	*
2	2	1	chromosome segregation protein	*	*	*	*	*	*	*	12T273_13160	*	CCUG54800_10830	*	*
1	1	1	Polyketide cyclase / dehydrase and lipid transp	*	*	*	*	*	*	*	12T273_13250	*	*	*	*
1	1	1	hypothetical protein	*	*	*	*	*	*	*	12T273_13270	*	*	*	*
1	1	1	putative acetyltransferase	*	*	*	*	*	*	*	12T273_13290	*	*	*	*
1	1	1	hypothetical protein	*	*	*	*	*	*	*	12T273_13300	*	*	*	*
1	1	1	hypothetical protein	*	*	*	*	*	*	*	12T273_14170	*	*	*	*
2	2	1	Cation efflux family protein	*	*	*	*	*	*	*	12T273_14230	*	*	*	T160124_10250
1	1	1	hypothetical protein	*	*	*	*	*	*	*	12T273_15600	*	*	*	*
1	1	1	hypothetical protein	*	*	*	*	*	*	*	12T273_16590	*	*	*	*
1	1	1	Putative multidrug export ATP-binding/permea	*	*	*	*	*	*	*	12T273_16680	*	*	*	*
1	1	1	4'-phosphopantetheinyl transferase sfp	*	*	*	*	*	*	*	12T273_16700	*	*	*	*
1	1	1	Peptidase family U32	*	*	*	*	*	*	*	12T273_16710	*	*	*	*
1	1	1	Linear gramicidin synthase subunit D	*	*	*	*	*	*	*	12T273_16720	*	*	*	*
1	1	1	hypothetical protein	*	*	*	*	*	*	*	12T273_16820	*	*	*	*
1	1	1	Platelet binding protein GspB precursor	*	*	*	*	*	*	*	12T273_17160	*	*	*	*
1	1	1	hypothetical protein	*	*	*	*	*	*	*	12T273_17360	*	*	*	*
2	2	1	Putative ABC transporter ATP-binding protein A	*	*	*	*	*	*	*	12T273_17380	*	*	*	T160124_13130
2	2	1	hypothetical protein	*	*	*	*	*	*	*	12T273_17390	*	*	*	T160124_13140
1	1	1	Lipoprotein-releasing system ATP-binding prot	*	*	*	*	*	*	*	12T273_17410	*	*	*	*
1	1	1	Pyrroline-5-carboxylate reductase	*	*	*	*	*	*	*	12T273_17510	*	*	*	*
1	1	1	Pyrroline-5-carboxylate reductase	*	*	*	*	*	*	*	12T273_17520	*	*	*	*
1	1	1	hypothetical protein	*	*	*	*	*	*	*	12T273_17540	*	*	*	*
2	2	1	hypothetical protein	*	*	*	*	*	*	*	12T273_17580	*	*	*	T160124_13380
3	3	1	hypothetical protein	*	*	*	*	*	*	*	12T273_17590	12T306_15610	*	*	T160124_13390
3	3	1	hypothetical protein	*	*	*	*	*	*	*	12T273_17600	12T306_15620	*	*	T160124_13400
2	2	1	Arsenate-mycothiol transferase ArsC1	*	*	*	*	*	*	*	12T273_17610	*	*	*	T160124_13410
2	3	1	Transcriptional repressor SdpR	*	*	*	*	*	*	*	12T273_17620	*	*	*	T160124_13420,T160124_13710

1	1	1	hypothetical protein	*	*	*	*	*	*	*	12T273_17630	*	*	*	*
3	3	1	Catalase HPII	*	*	*	*	*	*	*	12T273_17660	*	*	T151023_04300	T160124_13530
3	3	1	hypothetical protein	*	*	*	*	*	*	*	12T273_17720	12T306_15660	CCUG54800_15500	*	*
1	1	1	Polyketide synthase PksJ	*	*	*	*	*	*	*	12T273_17770	*	*	*	*
1	1	1	Linear gramicidin dehydrogenase LgrE	*	*	*	*	*	*	*	12T273_17780	*	*	*	*
1	1	1	Chorismate synthase	*	*	*	*	*	*	*	12T273_17810	*	*	*	*
1	1	1	Isochorismate synthase/isochorismate lyase	*	*	*	*	*	*	*	12T273_17840	*	*	*	*
1	1	1	hypothetical protein	*	*	*	*	*	*	*	12T273_17850	*	*	*	*
1	1	1	pyrroloquinoline quinone biosynthesis protein	*	*	*	*	*	*	*	12T273_18010	*	*	*	*
1	1	1	hypothetical protein	*	*	*	*	*	*	*	12T273_18030	*	*	*	*
1	1	1	hypothetical protein	*	*	*	*	*	*	*	12T273_18070	*	*	*	*
1	1	1	Peptostreptococcal albumin-binding protein pr	*	*	*	*	*	*	*	12T273_18110	*	*	*	*
1	1	1	hypothetical protein	*	*	*	*	*	*	*	12T273_18270	*	*	*	*
1	1	1	Type I restriction modification DNA specificity	*	*	*	*	*	*	*	12T273_18290	*	*	*	*
1	1	1	putative type I restriction enzymeP M protein	*	*	*	*	*	*	*	12T273_18300	*	*	*	*
1	1	1	Surface protein G precursor	*	*	*	*	*	*	*	12T273_18570	*	*	*	*
1	1	1	hypothetical protein	*	*	*	*	*	*	*	12T273_18660	*	*	*	*
1	1	1	Transposase IS116/IS110/IS902 family protein	*	*	*	*	*	*	*	*	12T306_00470	*	*	*
1	1	1	N-acetylmuramoyl-L-alanine amidase LytC pre	*	*	*	*	*	*	*	*	12T306_00490	*	*	*
2	2	1	hypothetical protein	*	*	*	*	*	*	*	*	12T306_00590	CCUG54800_00200	*	*
2	2	1	glutamine ABC transporter ATP-binding protei	*	*	*	*	*	*	*	*	12T306_00600	CCUG54800_00210	*	*
2	2	1	Inner membrane protein YbaN	*	*	*	*	*	*	*	*	12T306_00640	CCUG54800_00250	*	*
1	1	1	N-acetylmuramoyl-L-alanine amidase LytC pre	*	*	*	*	*	*	*	*	12T306_00660	*	*	*
1	1	1	hypothetical protein	*	*	*	*	*	*	*	*	12T306_01110	*	*	*
1	1	1	hypothetical protein	*	*	*	*	*	*	*	*	12T306_01120	*	*	*
1	1	1	2-ketoisovalerate ferredoxin oxidoreductase s	*	*	*	*	*	*	*	*	12T306_01210	*	*	*
1	1	1	Sec-independent protein translocase protein T	*	*	*	*	*	*	*	*	12T306_01220	*	*	*
1	1	1	chaperone protein TorD	*	*	*	*	*	*	*	*	12T306_01230	*	*	*
1	1	1	Anaerobic dimethyl sulfoxide reductase chain	*	*	*	*	*	*	*	*	12T306_01250	*	*	*
1	1	1	Sec-independent protein translocase protein T	*	*	*	*	*	*	*	*	12T306_01260	*	*	*
1	1	1	Cytosol non-specific dipeptidase	*	*	*	*	*	*	*	*	12T306_02420	*	*	*
2	2	1	Acetyltransferase (GNAT) family protein	*	*	*	*	*	*	*	*	12T306_02550	*	*	T160124_14250
1	1	1	hypothetical protein	*	*	*	*	*	*	*	*	12T306_02820	*	*	*
1	1	1	hypothetical protein	*	*	*	*	*	*	*	*	12T306_02830	*	*	*
1	1	1	ABC-2 family transporter protein	*	*	*	*	*	*	*	*	12T306_02850	*	*	*
1	1	1	FtsX-like permease family protein	*	*	*	*	*	*	*	*	12T306_02860	*	*	*
1	1	1	ABC 3 transport family protein	*	*	*	*	*	*	*	*	12T306_03490	*	*	*
1	1	1	hypothetical protein	*	*	*	*	*	*	*	*	12T306_04310	*	*	*
1	1	1	Multiple antibiotic resistance protein MarR	*	*	*	*	*	*	*	*	12T306_04350	*	*	*
1	1	1	hypothetical protein	*	*	*	*	*	*	*	*	12T306_04360	*	*	*
1	1	1	anaerobic benzoate catabolism transcriptional	*	*	*	*	*	*	*	*	12T306_04370	*	*	*
1	1	1	ICEBs1 excisionase	*	*	*	*	*	*	*	*	12T306_04380	*	*	*
1	1	1	hypothetical protein	*	*	*	*	*	*	*	*	12T306_04400	*	*	*
1	1	1	Virulence-associated protein E	*	*	*	*	*	*	*	*	12T306_04410	*	*	*
1	1	1	hypothetical protein	*	*	*	*	*	*	*	*	12T306_04420	*	*	*
1	1	1	hypothetical protein	*	*	*	*	*	*	*	*	12T306_04430	*	*	*
1	1	1	hypothetical protein	*	*	*	*	*	*	*	*	12T306_04440	*	*	*
1	1	1	hypothetical protein	*	*	*	*	*	*	*	*	12T306_04450	*	*	*
1	1	1	hypothetical protein	*	*	*	*	*	*	*	*	12T306_04460	*	*	*
1	1	1	hypothetical protein	*	*	*	*	*	*	*	*	12T306_04470	*	*	*
1	1	1	hypothetical protein	*	*	*	*	*	*	*	*	12T306_04480	*	*	*
1	1	1	FtsX-like permease family protein	*	*	*	*	*	*	*	*	12T306_04490	*	*	*
1	1	1	Lipoprotein-releasing system ATP-binding prot	*	*	*	*	*	*	*	*	12T306_04500	*	*	*
1	1	1	hypothetical protein	*	*	*	*	*	*	*	*	12T306_04670	*	*	*
1	1	1	hypothetical protein	*	*	*	*	*	*	*	*	12T306_04840	*	*	*
1	1	1	hypothetical protein	*	*	*	*	*	*	*	*	12T306_04850	*	*	*
1	1	1	O-Antigen ligase	*	*	*	*	*	*	*	*	12T306_05600	*	*	*
1	1	1	Putative metallo-hydrolase YycJ	*	*	*	*	*	*	*	*	12T306_06130	*	*	*
1	1	1	Dimethyl sulfoxide/trimethylamine N-oxide re	*	*	*	*	*	*	*	*	12T306_06720	*	*	*
1	1	1	Integrase core domain protein	*	*	*	*	*	*	*	*	12T306_08590	*	*	*
1	1	1	hypothetical protein	*	*	*	*	*	*	*	*	12T306_08840	*	*	*
1	1	1	ABC-2 family transporter protein	*	*	*	*	*	*	*	*	12T306_11170	*	*	*
1	1	1	ABC-2 family transporter protein	*	*	*	*	*	*	*	*	12T306_11180	*	*	*
1	1	1	ABC-2 family transporter protein	*	*	*	*	*	*	*	*	12T306_11200	*	*	*
1	1	1	Sensor histidine kinase YycG	*	*	*	*	*	*	*	*	12T306_11210	*	*	*
1	1	1	chromosome segregation protein	*	*	*	*	*	*	*	*	12T306_11300	*	*	*
1	1	1	hypothetical protein	*	*	*	*	*	*	*	*	12T306_11470	*	*	*
1	1	1	multidrug efflux pump VmrA	*	*	*	*	*	*	*	*	12T306_11480	*	*	*
1	1	1	multidrug efflux pump VmrA	*	*	*	*	*	*	*	*	12T306_11490	*	*	*
1	1	1	hypothetical protein	*	*	*	*	*	*	*	*	12T306_11860	*	*	*
1	1	1	SigmaK-factor processing regulatory protein Bc	*	*	*	*	*	*	*	*	12T306_13050	*	*	*
1	1	1	hypothetical protein	*	*	*	*	*	*	*	*	12T306_13670	*	*	*
2	3	1	hypothetical protein	*	*	*	*	*	*	*	*	12T306_13690	CCUG54800_13360,CCUG548	*	*
1	1	1	LD-carboxypeptidase	*	*	*	*	*	*	*	*	12T306_14770	*	*	*
1	1	1	hypothetical protein	*	*	*	*	*	*	*	*	12T306_14780	*	*	*
1	1	1	hypothetical protein	*	*	*	*	*	*	*	*	12T306_15050	*	*	*
1	1	1	hypothetical protein	*	*	*	*	*	*	*	*	12T306_15390	*	*	*
1	1	1	Subtilase family protein	*	*	*	*	*	*	*	*	12T306_15900	*	*	*
1	1	1	ATP-dependent zinc metalloprotease FtsH	*	*	*	*	*	*	*	*	12T306_15910	*	*	*
1	1	1	GIY-YIG catalytic domain protein	*	*	*	*	*	*	*	*	12T306_15950	*	*	*
1	1	1	hypothetical protein	*	*	*	*	*	*	*	*	12T306_15960	*	*	*
1	1	1	hypothetical protein	*	*	*	*	*	*	*	*	12T306_15970	*	*	*
1	1	1	hypothetical protein	*	*	*	*	*	*	*	*	12T306_15980	*	*	*
2	2	1	lysophospholipase L2	*	*	*	*	*	*	*	*	12T306_15990	*	T151023_03070	*
1	1	1	Peptostreptococcal albumin-binding protein pr	*	*	*	*	*	*	*	*	12T306_16410	*	*	*
1	1	1	Peptostreptococcal albumin-binding protein pr	*	*	*	*	*	*	*	*	12T306_16420	*	*	*
1	1	1	hypothetical protein	*	*	*	*	*	*	*	*	12T306_16430	*	*	*
1	1	1	hypothetical protein	*	*	*	*	*	*	*	*	12T306_17030	*	*	*
1	2	1	N-acetylmuramoyl-L-alanine amidase LytC pre	*	*	*	*	*	*	*	*	*	CCUG54800_00090,CCUG548	*	*
2	2	1	thiamine biosynthesis protein ThiH	*	*	*	*	*	*	*	*	*	CCUG54800_00420	T151023_00880	*
1	1	1	hypothetical protein	*	*	*	*	*	*	*	*	*	CCUG54800_00750	*	*
1	1	1	site-specific tyrosine recombinase XerC	*	*	*	*	*	*	*	*	*	CCUG54800_00800	*	*
1	1	1	hypothetical protein	*	*	*	*	*	*	*	*	*	CCUG54800_00830	*	*
1	1	1	hypothetical protein	*	*	*	*	*	*	*	*	*	CCUG54800_00840	*	*
1	1	1	hypothetical protein	*	*	*	*	*	*	*	*	*	CCUG54800_00870	*	*
1	1	1	chromosome segregation protein	*	*	*	*	*	*	*	*	*	CCUG54800_00880	*	*
1	1	1	recombination and repair protein RecT	*	*	*	*	*	*	*	*	*	CCUG54800_00890	*	*
1	1	1	Putative metallo-hydrolase YycJ	*	*	*	*	*	*	*	*	*	CCUG54800_00900	*	*
1	1	1	Replication initiation and membrane attachme	*	*	*	*	*	*	*	*	*	CCUG54800_00920	*	*
1	1	1	hypothetical protein	*	*	*	*	*	*	*	*	*	CCUG54800_00930	*	*
1	1	1	hypothetical protein	*	*	*	*	*	*	*	*	*	CCUG54800_00950	*	*
1	1	1	hypothetical protein	*	*	*	*	*	*	*	*	*	CCUG54800_00970	*	*
1	1	1	hypothetical protein	*	*	*	*	*	*	*	*	*	CCUG54800_00980	*	*
1	1	1	YopX protein	*	*	*	*	*	*	*	*	*	CCUG54800_00990	*	*
1	1	1	hypothetical protein	*	*	*	*	*	*	*	*	*	CCUG54800_01000	*	*
1	1	1	hypothetical protein	*	*	*	*	*	*	*	*	*	CCUG54800_01030	*	*
1	1	1	hypothetical protein	*	*	*	*	*	*	*	*	*	CCUG54800_01050	*	*
1	1	1	hypothetical protein	*	*	*	*	*	*	*	*	*	CCUG54800_01060	*	*
1	1	1	Phage terminase large subunit	*	*	*	*	*	*	*	*	*	CCUG54800_01070	*	*
1	1	1	Phage portal protein, SPP1 Gp6-like	*	*	*	*	*	*	*	*	*	CCUG54800_01080	*	*
1	1	1	NAD(+)--arginine ADP-ribosyltransferase EFV	*	*	*	*	*	*	*	*	*	CCUG54800_01090	*	*
1	1	1	hypothetical protein	*	*	*	*	*	*	*	*	*	CCUG54800_01110	*	*
1	1	1	hypothetical protein	*	*	*	*	*	*	*	*	*	CCUG54800_01120	*	*
1	1	1	hypothetical protein	*	*	*	*	*	*	*	*	*	CCUG54800_01130	*	*
1	1	1	hypothetical protein	*	*	*	*	*	*	*	*	*	CCUG54800_01140	*	*
1	1	1	hypothetical protein	*	*	*	*	*	*	*	*	*	CCUG54800_01150	*	*
1	1	1	hypothetical protein	*	*	*	*	*	*	*	*	*	CCUG54800_01160	*	*
1	1	1	hypothetical protein	*	*	*	*	*	*	*	*	*	CCUG54800_01170	*	*
1	1	1	Phage tail sheath protein	*	*	*	*	*	*	*	*	*	CCUG54800_01180	*	*
1	1	1	Phage-like element PBSX protein XkdM	*	*	*	*	*	*	*	*	*	CCUG54800_01190	*	*
1	1	1	Phage XkdN-like protein	*	*	*	*	*	*	*	*	*	CCUG54800_01200	*	*
1	1	1	hypothetical protein	*	*	*	*	*	*	*	*	*	CCUG54800_01210	*	*
1	1	1	hypothetical protein	*	*	*	*	*	*	*	*	*	CCUG54800_01220	*	*
1	1	1	LysM domain/BON superfamily protein	*	*	*	*	*	*	*	*	*	CCUG54800_01230	*	*
1	1	1	NlpC/P60 family protein	*	*	*	*	*	*	*	*	*	CCUG54800_01240	*	*
1	1	1	hypothetical protein	*	*	*	*	*	*	*	*	*	CCUG54800_01250	*	*
1	1	1	hypothetical protein	*	*	*	*	*	*	*	*	*	CCUG54800_01260	*	*
1	1	1	Baseplate J-like protein	*	*	*	*	*	*	*	*	*	CCUG54800_01270	*	*
1	1	1	hypothetical protein	*	*	*	*	*	*	*	*	*	CCUG54800_01280	*	*
1	1	1	hypothetical protein	*	*	*	*	*	*	*	*	*	CCUG54800_01290	*	*
1	1	1	hypothetical protein	*	*	*	*	*	*	*	*	*	CCUG54800_01300	*	*
1	1	1	hypothetical protein	*	*	*	*	*	*	*	*	*	CCUG54800_01310	*	*
1	1	1	hypothetical protein	*	*	*	*	*	*	*	*	*	CCUG54800_01320	*	*
1	2	1	hypothetical protein	*	*	*	*	*	*	*	*	*	CCUG54800_01330,CCUG548	*	*
1	1	1	Collagen triple helix repeat (20 copies)	*	*	*	*	*	*	*	*	*	CCUG54800_01350	*	*
1	1	1	hypothetical protein	*	*	*	*	*	*	*	*	*	CCUG54800_01360	*	*
1	1	1	hypothetical protein	*	*	*	*	*	*	*	*	*	CCUG54800_01370	*	*
1	1	1	Glycosyl hydrolases family 25	*	*	*	*	*	*	*	*	*	CCUG54800_01390	*	*
1	1	1	hypothetical protein	*	*	*	*	*	*	*	*	*	CCUG54800_01410	*	*
1	1	1	hypothetical protein	*	*	*	*	*	*	*	*	*	CCUG54800_01420	*	*
1	1	1	hypothetical protein	*	*	*	*	*	*	*	*	*	CCUG54800_01640	*	*
1	1	1	hypothetical protein	*	*	*	*	*	*	*	*	*	CCUG54800_02430	*	*
1	1	1	hypothetical protein	*	*	*	*	*	*	*	*	*	CCUG54800_02440	*	*
1	1	1	hypothetical protein	*	*	*	*	*	*	*	*	*	CCUG54800_02770	*	*
1	1	1	Helix-turn-helix	*	*	*	*	*	*	*	*	*	CCUG54800_03760	*	*
1	1	1	hypothetical protein	*	*	*	*	*	*	*	*	*	CCUG54800_04050	*	*
1	1	1	hypothetical protein	*	*	*	*	*	*	*	*	*	CCUG54800_04060	*	*
1	1	1	multidrug efflux pump VmrA	*	*	*	*	*	*	*	*	*	CCUG54800_04930	*	*
1	1	1	hypothetical protein	*	*	*	*	*	*	*	*	*	CCUG54800_05660	*	*
1	1	1	hypothetical protein	*	*	*	*	*	*	*	*	*	CCUG54800_08090	*	*
1	2	1	hypothetical protein	*	*	*	*	*	*	*	*	*	CCUG54800_10430,CCUG548	*	*
1	1	1	hypothetical protein	*	*	*	*	*	*	*	*	*	CCUG54800_10590	*	*
1	1	1	Bacitracin export permease protein BceB	*	*	*	*	*	*	*	*	*	CCUG54800_10700	*	*
1	1	1	hypothetical protein	*	*	*	*	*	*	*	*	*	CCUG54800_10740	*	*
1	1	1	hypothetical protein	*	*	*	*	*	*	*	*	*	CCUG54800_10910	*	*
1	1	1	hypothetical protein	*	*	*	*	*	*	*	*	*	CCUG54800_11010	*	*
1	1	1	hypothetical protein	*	*	*	*	*	*	*	*	*	CCUG54800_11020	*	*
1	1	1	hypothetical protein	*	*	*	*	*	*	*	*	*	CCUG54800_11040	*	*
1	1	1	hypothetical protein	*	*	*	*	*	*	*	*	*	CCUG54800_11050	*	*
1	1	1	Transcriptional repressor SdpR	*	*	*	*	*	*	*	*	*	CCUG54800_11060	*	*
1	1	1	hypothetical protein	*	*	*	*	*	*	*	*	*	CCUG54800_11790	*	*
1	1	1	hypothetical protein	*	*	*	*	*	*	*	*	*	CCUG54800_12680	*	*
1	2	1	hypothetical protein	*	*	*	*	*	*	*	*	*	CCUG54800_13080,CCUG548	*	*
1	1	1	hypothetical protein	*	*	*	*	*	*	*	*	*	CCUG54800_13300	*	*
1	1	1	hypothetical protein	*	*	*	*	*	*	*	*	*	CCUG54800_13310	*	*
1	1	1	hypothetical protein	*	*	*	*	*	*	*	*	*	CCUG54800_13350	*	*
1	1	1	Ferrous iron transport protein B	*	*	*	*	*	*	*	*	*	CCUG54800_13580	*	*
1	1	1	hypothetical protein	*	*	*	*	*	*	*	*	*	CCUG54800_14310	*	*
1	1	1	cobalt-precorrin-6Y C(15)-methyltransferase	*	*	*	*	*	*	*	*	*	CCUG54800_14390	*	*
1	1	1	hypothetical protein	*	*	*	*	*	*	*	*	*	CCUG54800_14410	*	*
1	1	1	Phosphopantetheine attachment site	*	*	*	*	*	*	*	*	*	CCUG54800_14430	*	*
1	1	1	Alpha/beta hydrolase family protein	*	*	*	*	*	*	*	*	*	CCUG54800_14440	*	*
1	1	1	Anguibactin system regulator	*	*	*	*	*	*	*	*	*	CCUG54800_14460	*	*
1	1	1	Histidine kinase-, DNA gyrase B-, and HSP90-lik	*	*	*	*	*	*	*	*	*	CCUG54800_14910	*	*
1	1	1	HaeIII restriction endonuclease	*	*	*	*	*	*	*	*	*	CCUG54800_14930	*	*
1	1	1	Rib/alpha-like repeat protein	*	*	*	*	*	*	*	*	*	CCUG54800_15040	*	*
1	1	1	hypothetical protein	*	*	*	*	*	*	*	*	*	CCUG54800_15240	*	*
1	1	1	hypothetical protein	*	*	*	*	*	*	*	*	*	CCUG54800_15250	*	*
1	1	1	hypothetical protein	*	*	*	*	*	*	*	*	*	CCUG54800_15480	*	*
1	1	1	hypothetical protein	*	*	*	*	*	*	*	*	*	CCUG54800_15520	*	*
1	1	1	2-succinyl-6-hydroxy-2,4-cyclohexadiene-1-car	*	*	*	*	*	*	*	*	*	CCUG54800_15640	*	*
1	1	1	Anguibactin system regulator	*	*	*	*	*	*	*	*	*	CCUG54800_15670	*	*
1	1	1	Multidrug export protein MepA	*	*	*	*	*	*	*	*	*	CCUG54800_15680	*	*
1	1	1	hypothetical protein	*	*	*	*	*	*	*	*	*	CCUG54800_15790	*	*
1	1	1	Relaxase/Mobilisation nuclease domain protei	*	*	*	*	*	*	*	*	*	CCUG54800_15900	*	*
1	1	1	ski2-like helicase	*	*	*	*	*	*	*	*	*	CCUG54800_15910	*	*
1	1	1	hypothetical protein	*	*	*	*	*	*	*	*	*	CCUG54800_15920	*	*
1	1	1	hypothetical protein	*	*	*	*	*	*	*	*	*	CCUG54800_15930	*	*
1	1	1	Metallo-beta-lactamase superfamily protein	*	*	*	*	*	*	*	*	*	CCUG54800_15960	*	*
1	1	1	cytidine deaminase	*	*	*	*	*	*	*	*	*	CCUG54800_15970	*	*
1	1	1	Phosphotransferase enzyme family protein	*	*	*	*	*	*	*	*	*	CCUG54800_15980	*	*
1	1	1	Ribosomal protein L11 methyltransferase	*	*	*	*	*	*	*	*	*	CCUG54800_16000	*	*
1	1	1	Malonyl-[acyl-carrier protein] O-methyltransfe	*	*	*	*	*	*	*	*	*	CCUG54800_16010	*	*
1	1	1	Nucleotidyltransferase domain protein	*	*	*	*	*	*	*	*	*	CCUG54800_16020	*	*
1	1	1	hypothetical protein	*	*	*	*	*	*	*	*	*	CCUG54800_16530	*	*
1	1	1	EcoKI restriction-modification system protein	*	*	*	*	*	*	*	*	*	CCUG54800_16730	*	*
1	1	1	putative type I restriction enzymeP M protein	*	*	*	*	*	*	*	*	*	CCUG54800_16740	*	*
1	1	1	hypothetical protein	*	*	*	*	*	*	*	*	*	CCUG54800_16780	*	*
1	1	1	Internalin-A precursor	*	*	*	*	*	*	*	*	*	CCUG54800_17400	*	*
1	1	1	Peptostreptococcal albumin-binding protein pr	*	*	*	*	*	*	*	*	*	CCUG54800_17560	*	*
1	1	1	hypothetical protein	*	*	*	*	*	*	*	*	*	CCUG54800_17960	*	*
1	1	1	hypothetical protein	*	*	*	*	*	*	*	*	*	CCUG54800_17970	*	*
1	1	1	HTH-type quorum sensing-dependent transcri	*	*	*	*	*	*	*	*	*	CCUG54800_19020	*	*
1	1	1	Transcriptional regulatory protein DegU	*	*	*	*	*	*	*	*	*	CCUG54800_19030	*	*
1	1	1	hypothetical protein	*	*	*	*	*	*	*	*	*	CCUG54800_19040	*	*
1	1	1	hypothetical protein	*	*	*	*	*	*	*	*	*	CCUG54800_19050	*	*
1	1	1	Antilisterial bacteriocin subtilosin biosynthesis	*	*	*	*	*	*	*	*	*	CCUG54800_19130	*	*
1	1	1	Antilisterial bacteriocin subtilosin biosynthesis	*	*	*	*	*	*	*	*	*	CCUG54800_19140	*	*
1	1	1	Antilisterial bacteriocin subtilosin biosynthesis	*	*	*	*	*	*	*	*	*	CCUG54800_19150	*	*
1	1	1	pyrroloquinoline quinone biosynthesis protein	*	*	*	*	*	*	*	*	*	CCUG54800_19160	*	*

1	1	1	Acetyltransferase YpeA	*	*	*	*	*	*	*	*	*	CCUG54800_19170	*	*
1	1	1	hypothetical protein	*	*	*	*	*	*	*	*	*	CCUG54800_19180	*	*
1	1	1	ABC-2 family transporter protein	*	*	*	*	*	*	*	*	*	CCUG54800_19220	*	*
1	1	1	hypothetical protein	*	*	*	*	*	*	*	*	*	CCUG54800_19230	*	*
1	1	1	putative ABC transporter ATP-binding protein	*	*	*	*	*	*	*	*	*	CCUG54800_19270	*	*
1	1	1	Bacitracin export permease protein BceB	*	*	*	*	*	*	*	*	*	CCUG54800_19280	*	*
1	1	1	hypothetical protein	*	*	*	*	*	*	*	*	*	CCUG54800_19310	*	*
1	1	1	PrgI family protein	*	*	*	*	*	*	*	*	*	CCUG54800_19470	*	*
1	1	1	hypothetical protein	*	*	*	*	*	*	*	*	*	CCUG54800_19520	*	*
1	1	1	hypothetical protein	*	*	*	*	*	*	*	*	*	*	T151023_01270	*
1	1	1	hypothetical protein	*	*	*	*	*	*	*	*	*	*	T151023_01280	*
1	1	1	Cna protein B-type domain protein	*	*	*	*	*	*	*	*	*	*	T151023_02180	*
1	1	1	hypothetical protein	*	*	*	*	*	*	*	*	*	*	T151023_02290	*
1	1	1	hypothetical protein	*	*	*	*	*	*	*	*	*	*	T151023_02760	*
1	1	1	ATP-dependent DNA helicase PcrA	*	*	*	*	*	*	*	*	*	*	T151023_02770	*
1	1	1	hypothetical protein	*	*	*	*	*	*	*	*	*	*	T151023_02900	*
1	1	1	hypothetical protein	*	*	*	*	*	*	*	*	*	*	T151023_03040	*
1	1	1	hypothetical protein	*	*	*	*	*	*	*	*	*	*	T151023_03050	*
1	1	1	Transposase	*	*	*	*	*	*	*	*	*	*	T151023_03540	*
1	1	1	Phosphate-binding protein PstS 2 precursor	*	*	*	*	*	*	*	*	*	*	T151023_06180	*
1	1	1	hypothetical protein	*	*	*	*	*	*	*	*	*	*	T151023_07330	*
1	1	1	SigmaK-factor processing regulatory protein Bc	*	*	*	*	*	*	*	*	*	*	T151023_10950	*
1	1	1	hypothetical protein	*	*	*	*	*	*	*	*	*	*	T151023_12440	*
1	1	1	hypothetical protein	*	*	*	*	*	*	*	*	*	*	T151023_12470	*
1	1	1	Sugar-specific transcriptional regulator TrmB	*	*	*	*	*	*	*	*	*	*	T151023_12850	*
2	4	1	hypothetical protein	*	*	*	*	*	*	*	*	*	*	T151023_13450,T151023_	T160124_01770,T160124_01760
1	1	1	Oligopeptide transport ATP-binding protein Op	*	*	*	*	*	*	*	*	*	*	T151023_13870	*
1	1	1	Oligopeptide transport ATP-binding protein Op	*	*	*	*	*	*	*	*	*	*	T151023_13880	*
1	1	1	hypothetical protein	*	*	*	*	*	*	*	*	*	*	T151023_14320	*
1	1	1	N-acetylmuramoyl-L-alanine amidase LytC pre	*	*	*	*	*	*	*	*	*	*	T151023_15270	*
1	1	1	hypothetical protein	*	*	*	*	*	*	*	*	*	*	T151023_15760	*
1	1	1	hypothetical protein	*	*	*	*	*	*	*	*	*	*	T151023_15920	*
1	1	1	hypothetical protein	*	*	*	*	*	*	*	*	*	*	*	T160124_00220
1	1	1	hypothetical protein	*	*	*	*	*	*	*	*	*	*	*	T160124_00250
1	1	1	hypothetical protein	*	*	*	*	*	*	*	*	*	*	*	T160124_00270
1	1	1	hypothetical protein	*	*	*	*	*	*	*	*	*	*	*	T160124_00500
1	1	1	hypothetical protein	*	*	*	*	*	*	*	*	*	*	*	T160124_00700
1	1	1	hypothetical protein	*	*	*	*	*	*	*	*	*	*	*	T160124_02450
1	1	1	Type I restriction enzyme EcoR124II R protein	*	*	*	*	*	*	*	*	*	*	*	T160124_03060
1	1	1	HTH-type transcriptional regulator CynR	*	*	*	*	*	*	*	*	*	*	*	T160124_04600
1	1	1	hypothetical protein	*	*	*	*	*	*	*	*	*	*	*	T160124_04620
1	1	1	hypothetical protein	*	*	*	*	*	*	*	*	*	*	*	T160124_04630
1	1	1	hypothetical protein	*	*	*	*	*	*	*	*	*	*	*	T160124_04830
1	1	1	hypothetical protein	*	*	*	*	*	*	*	*	*	*	*	T160124_07250
1	1	1	hypothetical protein	*	*	*	*	*	*	*	*	*	*	*	T160124_08650
1	1	1	Redox-sensing transcriptional repressor Rex	*	*	*	*	*	*	*	*	*	*	*	T160124_09060
1	1	1	Serine-aspartate repeat-containing protein F p	*	*	*	*	*	*	*	*	*	*	*	T160124_09450
1	1	1	hypothetical protein	*	*	*	*	*	*	*	*	*	*	*	T160124_09460
1	1	1	hypothetical protein	*	*	*	*	*	*	*	*	*	*	*	T160124_10190
1	1	1	ABC transporter ATP-binding protein YtrE	*	*	*	*	*	*	*	*	*	*	*	T160124_12680
1	1	1	cysteine/glutathione ABC transporter membra	*	*	*	*	*	*	*	*	*	*	*	T160124_12690
1	1	1	hypothetical protein	*	*	*	*	*	*	*	*	*	*	*	T160124_12710
1	1	1	hypothetical protein	*	*	*	*	*	*	*	*	*	*	*	T160124_12830
1	1	1	Extracellular matrix-binding protein ebh precu	*	*	*	*	*	*	*	*	*	*	*	T160124_12920
1	1	1	hypothetical protein	*	*	*	*	*	*	*	*	*	*	*	T160124_12930
1	1	1	hypothetical protein	*	*	*	*	*	*	*	*	*	*	*	T160124_13150
1	1	1	hypothetical protein	*	*	*	*	*	*	*	*	*	*	*	T160124_13440
1	1	1	hypothetical protein	*	*	*	*	*	*	*	*	*	*	*	T160124_13490
1	1	1	ABC transporter ATPase component	*	*	*	*	*	*	*	*	*	*	*	T160124_13590
1	1	1	Sodium Bile acid symporter family protein	*	*	*	*	*	*	*	*	*	*	*	T160124_13680
1	1	1	Arsenical pump-driving ATPase	*	*	*	*	*	*	*	*	*	*	*	T160124_13690
1	1	1	Arsenical resistance operon trans-acting repre	*	*	*	*	*	*	*	*	*	*	*	T160124_13700
1	1	1	Transcriptional repressor CopY	*	*	*	*	*	*	*	*	*	*	*	T160124_13720
1	1	1	Copper-exporting P-type ATPase B	*	*	*	*	*	*	*	*	*	*	*	T160124_13730
1	1	1	hypothetical protein	*	*	*	*	*	*	*	*	*	*	*	T160124_13740
1	1	1	Cadmium resistance transporter	*	*	*	*	*	*	*	*	*	*	*	T160124_13750
1	1	1	hypothetical protein	*	*	*	*	*	*	*	*	*	*	*	T160124_13770
1	1	1	hypothetical protein	*	*	*	*	*	*	*	*	*	*	*	T160124_13780
1	1	1	hypothetical protein	*	*	*	*	*	*	*	*	*	*	*	T160124_14060
1	1	1	osmolarity sensor protein	*	*	*	*	*	*	*	*	*	*	*	T160124_14070
1	1	1	hypothetical protein	*	*	*	*	*	*	*	*	*	*	*	T160124_14090
1	1	1	hypothetical protein	*	*	*	*	*	*	*	*	*	*	*	T160124_14960
1	1	1	hypothetical protein	*	*	*	*	*	*	*	*	*	*	*	T160124_14990
1	1	1	Peptostreptococcal albumin-binding protein pr	*	*	*	*	*	*	*	*	*	*	*	T160124_15430
1	1	1	hypothetical protein	*	*	*	*	*	*	*	*	*	*	*	T160124_16050
1	1	1	Double zinc ribbon	*	*	*	*	*	*	*	*	*	*	*	T160124_16060
1	1	1	hypothetical protein	*	*	*	*	*	*	*	*	*	*	*	T160124_16070

Table S2: CRISPR/cas systems in *Finegoldia* sp.

STRAIN NAME	SPECIES	# CRISPR	# SPACERS
**07T609**	*F. magna*	1	13
**08T492**	*F. magna*	2	6, 6
**09T408**	*F. magna*	3	17, 16, 12
**09T494**	*"F. nericia"*	1	34
**12T272**	*"F. nericia"*	1	41
**12T273**	*"F. nericia"*	0	0
**12T306**	*"F. nericia"*	1	82
**CCUG54800**	*"F. nericia"*	0	0
**T151023**	*"F. nericia"*	1	61
**T160124**	*"F. nericia"*	1	14
**ATCC 29328**	*F. magna*	4	14, 3, 6, 4
**ATCC 53516**	*"F. nericia"*	1	87
**GED7760A**	*F. magna*	0	0
**ACS-171-V-COL3**	*F. magna*	3	6, 17, 14
**BVS033A4**	*F. magna*	1	38
**SY403409CC001050417**	*F. magna*	1	11
**ALB8**	*F. magna*	0	0

F. magna **07T609: DR length: 36 Number of spacers: 13**

34785	**GTTTGAGAATGATGTAATTTCATATAGGTATTAAAC**	ACGCGATTCTTGAGAGTGGTGCCGCTGATA	34850
34851	**GTTTGAGAATGATGTAATTTCATATAGGTATTAAAC**	TTTCCCTGTGTTAGGTGGTCCTTGAATAAA	34916
34917	**GTTTGAGAATGATGTAATTTCATATAGGTATTAAAC**	AAGTATTCCTTATCATCTTTTGCGACTGTA	34982
34983	**GTTTGAGAATGATGTAATTTCATATAGGTATTAAAC**	CAAGTTGGAGGGGGAGACCGTTCGGAAGCTA	35049
35050	**GTTTGAGAATGATGTAATTTCATATAGGTATTAAAC**	AACTCAAAAAGACGATATCAACGAACAAAT	35115
35116	**GTTTGAGAATGATGTAATTTCATATAGGTATTAAAC**	TGGATAAAAAATTCATATACATCTACACAA	35181
35182	**GTTTGAGAATGATGTAATTTCATATAGGTATTAAAC**	TGCCATGAATGTCGCCATCGATTTCATCGA	35247
35248	**GTTTGAGAATGATGTAATTTCATATAGGTATTAAAC**	CACGGAAAACTAAAAGAGTTGTACGAAAAA	35313
35314	**GTTTGAGAATGATGTAATTTCATATAGGTATTAAAC**	GCTATACTGATATACAAAAACTACGATATC	35379
35380	**GTTTGAGAATGATGTAATTTCATATAGGTATTAAAC**	TTTGTTGCTATGTCGTTGTTGCTATCCAGGA	35446
35447	**GTTTGAGAATGATGTAATTTCATATAGGTATTAAAC**	TTATATTTGCTAAAATAATGGCGATCGGGG	35512
35513	**GTTTGAGAATGATGTAATTTCATATAGGTATTAAAC**	TTCAACTGCATTTCTCAAATTAAAAAATGT	35578
35579	**GTTTGAGAATGATGTAATTTCATATAGGTATTAAAC**	AAAACGGCGTATAGGGTCTCGGGGTTCCCT	35644
35645	**GTTTGAGAATGATGTAATTTCATATAGGTATTAAAC**		35680

F. magna **08T492: DR length: 30 Number of spacers: 6**

7270	**CTTTACATTTCAGTATGATTAGATTTTAAT**	TTTCGCAGGCGTTGCAATCCAAGCTGCTGCTAAATA	7335
7336	**ATTTACATTTCAGTATGAGTAGATTTTAAT**	TTGGGCATTGCTGGAGCATTCCTTGCTTGGAAATT	7400
7401	**ATTTACATTTCAGTATGAGTAAATTTTAAT**	GCTGGTAAATTTGCAGGTTGGGATAAGACAATGCA	7465
7466	**ATTTACATTTCAGTATGAGTAGATTTTAAT**	ACAAATCCTCAATATTCACAATATAGTCATCAATATC	7532
7533	**ATTTACATTCCAATATGAGTAGATTTTAAT**	GATTTGATTAAATGGAAAGACCATATTCCAGAATT	7597
7598	**ATTTACATTTCAATATGAGTAGATTTTAAT**	TGGAGCTGGTCTTTTAGGTGGAAACGACAAAGAAGA	7663
7664	**ATTTACATTTCAATATGAGTAGATTTTAAT**		7693

F. magna **08T492: DR length: 25 Number of spacers: 6**

63273	**TTTTTCTTATCTGTTGTAGTTTTTA**	CTGGTCTACTAACTTTGTTGTCTGAAGAA	63326
63327	**GTTTTATCTGCTTTTTCTGATTTTC**	TAACAAGAGTTACTTTCTTCTTTTCTGCA	63380
63381	**TTTTTCTTGAATTCTTCGGCTTTTT**	CCTTAGCTTTAGCTTTCATTTCTTCA	63431
63432	**GCCTTTTTTTCTTCATCGGCCTTTT**	TCTTTGCTTCTTCAGCT	63473
63474	**TTTTTCTTTGCTTCTTCAGCTTTTT**	TCTTTGCTTCTTCGGCTTTTTTTGCTTCTTCAGCT	63533
63534	**TTTTTCTTTGCTTCTTCGGCTTTTT**	GTTTTTCTGCCGAATTTTTCAAGCCATCT	63587
63588	**ACTTTCTTTTCTTTTTCTGCTTGTT**		63612

F. magna **09T408: DR length: 36 Number of spacers: 17**

25488	**GTTTGAGAATGATGTAATTTCATATAGGTATTAAAC**	AAAATAACATTGAACAGTTTATACAAGCAA	25553
25554	**GTTTGAGAATGATGTAATTTCATATAGGTATTAAAC**	ATTCAATTTTGAATATCCTTCTTCCGTGAT	25619
25620	**GTTTGAGAATGATGTAATTTCATATAGGTATTAAAC**	AACATCATATCCGAGAAGTAATATGGAATA	25685
25686	**GTTTGAGAATGATGTAATTTCATATAGGTATTAAAC**	GCGTATTGAAATATCATTCCACTGTATATG	25751
25752	**GTTTGAGAATGATGTAATTTCATATAGGTATTAAAC**	ATCATCGCTTTACTCAGAAAAATTATTTTG	25817
25818	**GTTTGAGAATGATGTAATTTCATATAGGTATTAAAC**	TTCTTGAGTTTCTATTTGCTCATAATCAGAA	25884
25885	**GTTTGAGAATGATGTAATTTCATATAGGTATTAAAC**	ATGATACGCTGAAATGGATAGCTCAGATAGG	25951
25952	**GTTTGAGAATGATGTAATTTCATATAGGTATTAAAC**	CTGTAGACTTGAAGAATCCCATACAGGATA	26017
26018	**GTTTGAGAATGATGTAATTTCATATAGGTATTAAAC**	TAATCAAACGATTAAAGAAAAAAGAGATAA	26083
26084	**GTTTGAGAATGATGTAATTTCATATAGGTATTAAAC**	TCTTTTGTGTCTTCTATCTCTTCACCAAGA	26149
26150	**GTTTGAGAATGATGTAATTTCATATAGGTATTAAAC**	AGGATATATATCCCCTATCGACTTTATCAAG	26216
26217	**GTTTGAGAATGATGTAATTTCATATAGGTATTAAAC**	AATCATAGCAAGTCTTTTAGTCTTAGTAGT	26282
26283	**GTTTGAGAATGATGTAATTTCATATAGGTATTAAAC**	GATATTGGAGATATTGAGGACAAGGAATAT	26348
26349	**GTTTGAGAATGATGTAATTTCATATAGGTATTAAAC**	AATGCTACAAAAGATAATGGTCAAAGAGTA	26414
26415	**GTTTGAGAATGATGTAATTTCATATAGGTATTAAAC**	TTATAGGATAAAGTATTTGGGTTAAATTGA	26480
26481	**GTTTGAGAATGATGTAAATTCATATAGGTTTTAAAC**	AAAAGACGGGCAAGGACTTATCACATACGA	26546
26547	**GTTTGAGAATGATGTAATTTCATATAGGTATTAAAC**	AATGGTTATAGAGATTTCAAAAAAGTTACCG	26613
26614	**GTTTGAGAATGATGTAATTTCATATAGGTATTAAAC**		26649

F. magna **09T408: DR length: 30 Number of spacers: 16**

55864	**TGTTATACCCGCACACGCGGGGATGATCCT**	ATTACCAAGAACAGTTGAAAAAATTGTATGA	55924
55925	**TGTTATACCCGCACACGCGGGGATGATCCT**	GCACGTTTACGGCTCACTGTATGTTGGTAGT	55985
55986	**AGTTATACCCGCACACGCGGGGATGATCCT**	AAAGAAAAGTAAAAGTTGAGTAAAGTTGAGT	56046
56047	**AGTTATACCCGCACACGCGGGGATGATCCT**	ATCATTACTTATTTCCATAAGCCTATTAATT	56107
56108	**CGTTATACCCGCACACGCGGGGATGATCCT**	GATATATTGTTTATGTCATGAAGTCCGTCAT	56168
56169	**TGTTATACCCGCACACGCGGGGATGATCCT**	GTATAAGTTTTATCTGTAACTAAATTATCTA	56229
56230	**CGTTATACCCGCACACGCGGGGATGATCCT**	GTATAAGTTTTATCTGTAACTAAATTATCTA	56290
56291	**CGTTATACCCGCACACGCGGGGATGATCCT**	GTATAAGTTTTATCTGTAACTAAATTATCTA	56351
56352	**CGTTATACCCGCACACGCGGGGATGATCCT**	GTATAAGTTTTATCTGTAACTAAATTATCTA	56412
56413	**CGTTATACCCGCACACGCGGGGATGATCCT**	ACTTTTAGAAACTGATGTACTAATTTTATCG	56473
56474	**CGTTATACCCGCACACGCGGGGATGATCCT**	GTATAAGTTTTATCTGTAACTAAATTATCTA	56534
56535	**CGTTATACCCGCACACGCGGGGATGATCCT**	ACTTTTAGAAACTGATGTACTAATTTTATCG	56595
56596	**CGTTATACCCGCACACGCGGGGATGATCCT**	GTATAAGTTTTATCTGTAACTAAATTATCTA	56656
56657	**CGTTATACCCGCACACGCGGGGATGATCCT**	ACTTTTAGAAACTGATGTACTAATTTTATCG	56717
56718	**CGTTATACCCGCACACGCGGGGATGATCCT**	TCATTAGATCATTTTACACGAATTTTTACAG	56778
56779	**AGTTATACCCGCACACGCGGGGATGATCCC**	TGATAACCAAAATAAGAAGTCGCAAAATCAA	56839
56840	**CGTTATACCCGCACACGCGGGGATAATATT**		56869

F. magna **09T408: DR length: 31 Number of spacers: 12**

8034	**GATTTACATTCCATTCTGGTTAGATTTTAAT**	GATTGTTTTTATCATAGACTTAACCCTCTGCGT	8097
8098	**TATTTACATTCCACTCTGGTTAGATTTTAAT**	GAAAAAAGCGACGAAGTAACAATTGAAACCTTGATGA	8165
8166	**CATTTACATTCCACTCTGGTTAGATTTTAAT**	AGCAGTAATAGGAGTTTACAAGCTGGTATTATCA	8230
8231	**AATTTACATTCCACTCTGGTTAGATTTTAAT**	ACAATAGCTTTCGTTTCTGTTGCTTTTTCAAGAA	8295
8296	**CATTTACATTCCACTATGGGTAGATTTTAAT**	TTATCTTCTGATTCTTCTTGTTCTTTTTTATCAT	8360
8361	**CATTTACATTCCACTATGGGTAGATTTTAAT**	TTTCTACGAACTCCTTGCTCGCCTTGTCATACG	8424
8425	**CATTTACATTCCACTATGGGTAGATTTTAAT**	GTAATTTAGATATTGATGTAAAAAACATTGTTGTAA	8491
8492	**TATTTACATTCCACTATGGTTAGATTTTAAT**	TATCTAGATACATATCTAATATATATATAATTCTT	8555
8556	**TATTTACATTCCACTATGGTTAGATTTTAAT**	TAAAGAAGAATTAAGACGTGCCGAAGTATCAGGTC	8621
8622	**AATTTACATTCCACTCTGATTAGATTTTAAT**	GAACTCAGGTGGTATTACTTTATTTAAAGAAAAGG	8687
8688	**TATTTACATTCCACTATGGGTAGATTTTAAT**	TAAGACAACAACCAACACTCGAAGTCGGAGAAA	8751
8752	**TATTTACATTCCACTATGGGTAGATTTTAAT**	CGGGTAAAGTGAGGAGTCTGCAAAGTCTGCTTTGT	8817
8818	**TATTTACATTCCACTATGAGTAGATTTTAAT**		8848

F. magna **ATCC29328: DR length: 36 Number of spacers: 14**

79845	**GTTTGAGAATGATGTAATTTCATATAGGTATTAAAC**	TTTATCCTCGTCAAACTCTTTTCCACACAC	79910
79911	**GTTTGAGAATGATGTAATTTCATATAGGTATTAAAC**	TATGGTGGAGATGAGGAGTATCGTACGAAC	79976
79977	**GTTTGAGAATGATGTAATTTCATATAGGTATTAAAC**	AGTACGAATTTGGATTTACTCCAAACGACG	80042
80043	**GTTTGAGAATGATGTAATTTCATATAGGTATTAAAC**	GGATTAGGTGAACATACAATTACAGTTACT	80108
80109	**GTTTGAGAATGATGTAATTTCATATAGGTATTAAAC**	TCAAACTTGATGAGGTCAAGGATTTGATTT	80174
80175	**GTTTGAGAATGATGTAATTTCATATAGGTATTAAAC**	ATTTCTACACGATTTGTTTCATCATTTGAA	80240
80241	**GTTTGAGAATGATGTAATTTCATATAGGTATTAAAC**	TTGCTATTTTTGTTTTAATCTTTAGTTCCC	80306
80307	**GTTTGAGAATGATGTAATTTCATATAGGTATTAAAC**	ATACTGATTAATCTGAATGGTTTGAGTGAT	80372
80373	**GTTTGAGAATGATGTAATTTCATATAGGTATTAAAC**	ATTGTTTACAAGCTCCCGAATATAAGCTTG	80438
80439	**GTTTGAGAATGATGTAATTTCATATAGGTATTAAAC**	TGGTTATAAAAAACCGAAAGCAGAAGTGAA	80504
80505	**GTTTGAGAATGATGTAATTTCATATAGGTATTAAAC**	TTTGATGACTACGAATTGCAAGCAGTGTATA	80571
80572	**GTTTGAGAATGATGTAATTTCATATAGGTATTAAAC**	AATGATAATTTAAAAGAATACTTAATTTATA	80638
80639	**GTTTGAGAATGATGTAATTTCATATAGGTATTAAAC**	GAGTAAAACATAAAAAGATTGAGTATGTTG	80704
80705	**GTTTGAGAATGATGTAATTTCATATAGGTATTAAAC**	CTGAAAGTTTAGGATTGACGTTCGAGGACA	80770
80771	**GTTTGAGAATGATGTAATTTCATATAGGTATTAAAC**		80806

F. magna **ATCC29328: DR length: 28** **Number of spacers: 3**

45598	**AGGAACACCCCCGCTATTGCTGGGAAAA**	ATTGTGCTGTTTTAGGGCCTCCAGTATCTTCCCT	45659
45660	**AGGAACACCCCCGCATGTGCGGGTAAAA**	CTCAACACCGTTGTTGTTATTTACATTATTAAC	45720
45721	**AGAAACACCCCTGCACTTGCGGGTAAAA**	CTGTGGACAAAATCTGTAACATAACAATCCTAT	45781
45782	**AGAAACACCCCCGCATGTGCGGGTAAAA**		45810

F. magna **ATCC29328: DR length: 28** **Number of spacers: 6**

45891	**TGGAACACCCCTGCTATTGCTGGTAAAA**	TAAGTATTTATTACGAAAAGTTCATAGATAAAA	45951
45952	**AGGAACACCCCCGCATGTGCGGGTAAAA**	CTTATTCGATTCTTCATTTTTTTAGTTCTCTT	46011
46012	**AGGAACACCCCCGCTTTTGCGGGTAAAA**	CTTATTGTTGAAATTATCTTTGTTTTTAGAGTT	46072
46073	**AGAAATACCCCCGCATGTGCGGGTAACA**	TGCTGTGTCAAATGCAAAAGAAGATGTTTTAAAT	46134
46135	**AGGAACACCCCCGCATGTGCGGGTAAAA**	CATAAAGTTATAATTATAATCTTTTTTTATAAC	46195
46196	**AGGAACACCCCCGCTTGTGCGGGTAAAA**	TCATAGATACTTTTTTCGCTCCTTGAAATAATC	46256
46257	**AGAAACACCCCCGCTTGTGCGGGTAAAC**		46284

F. magna **ATCC29328: DR length: 25** **Number of spacers: 4**

79988	**AACACCCCCGCTTGTGCGGGTAAAA**	TTTTATTTAGAACTAACTGAGTCAGGCAACAGG	80045
80046	**AACACCCTCGCTTGTGCGGGTAAAA**	GGGAACTAATATGAATTAAGGAATGGCAATATAAGA	80106
80107	**AACACCCCCGCCTGTGCGGGTAATA**	TTAATTAAGGAATAGTTTTCATCAGAAACGCCATCGCCATCAGG	80175
80176	**AACACACTCGCTTGTGCGAGTAAAA**	TAAAAAAAATTTAGGAAATGGTGTTATGAGACAGA	80235
80236	**AACACACTCGCCATTGCGAGAAATA**		80260

F. magna **ALB8: none**

F. magna **GED7760A: none**

F. magna **ACS-171-V-Col3: DR length: 30** **Number of spacers: 6**

28319	**CTTTTCATTCCACTCTAGTTAGATTTTAAT**	ATAATATCGTGTTTAACTTCTGAGTTAATATAAT	28382
28383	**ATTTACATTTCAGTATGAGTAGATTTTAAT**	CACGTGACTGGCAAGATTGTAAAAATCGTATGAAT	28447
28448	**ATTTACATTCCACTATGGTTAGATTTTAAT**	ACGACCTTGTCCTTGTATGATTCAGTCTTTTGAAT	28512
28513	**ATTTACATTCCACTCTAGTTAGATTTTAAT**	GCTTTTCCCTTGCTTCTACACCCGTATTAGCGTACGC	28579
28580	**ATTTACATTCCACTCTGGTTAGATTTTAAT**	GATAGTTTCAGATAAAGGAACCAGCTTTTCAAGAAA	28645
28646	**ATTTACATTCCACTCTGGTTAGATTTTAAC**	GTACATGCTCACTGTTGGAAAACTATGGCAAAAGG	28710
28711	**ATTTACATTTCAGTATGAGTAGATTTTAAT**		28741

F. magna **ACS-171-V-Col3: DR length: 30   Number of spacers: 17**

28841	**ATTTACATTCCACTATGGGTAGATTTTAAT**	ATAAACACAAAGTATCGAATTACAAAGATAGATGT	28905
28906	**ATTTACATTCCACTCCGGTTATATTTTAAT**	ACTTACTTGAATTCGGACACATTTCTGCAAACCAATA	28972
28973	**ATTTACATTCCACTCTGGTTAGATTTTAAT**	TTGAAGACATAATCGAGACGATTAAGGATGAGAC	29036
29037	**ATTTTCATTCCACTCTGGTTAGATTTTAAT**	TAAAGATACTACAAGAAGATACTTAACTAAGAACAT	29102
29103	**ATTTACATTCCACTCTGGTTAGATTTTAAT**	GAGGTGGTTATCTCATGATAGAAACTAGGTTTTCAAGA	29170
29171	**ATTTACATTCCACTCTGGTTAGATTTTAAT**	TTTGCTTTCGCAGTATCCACAGTGAGTGTCGCTCCAAT	29238
29239	**ATTTACATTTCAGTATGATTAGATTTTAAT**	TCATATGATAACCTTTTATAGCTAAATCAAATTT	29302
29303	**ATTTACATTTCAGTATGAGTAGATTTTAAT**	ATAATGATTATGTTGTGGATATTGACACACTACCAA	29368
29369	**ATTTACATTTCAGTATGAGTAGATTTTAAT**	TTGGATTAACTTACTTGCAATTGAAGTAACTTCCCC	29434
29435	**ATTTACATTTCAGTATGAGTAGATTTTAAT**	TTAAAAATTTAAATAATACGCTTGATAAAACTTTTTT	29501
29502	**ATTTACATTTCAGTATGAGTAGATTTTAAT**	CAGAGTTGAAAATAAAGAAACTAAGTTTACAACTTA	29567
29568	**ATTTACATTTCAGTATGAGTAGATTTTAAT**	CTTCAAGGAGCGATTCCTTATAACAAAGCTAAATA	29632
29633	**ATTTACATTTCAGTATGAGTAGATTTTAAT**	GAACGTATCAAGAAATACGATATTTGGATAGCTCAC	29698
29699	**ATTTACATTTCAGTATGAGTAGATTTTAAT**	TGCTTTCGCAGTAGACACAGTCAAGTATGCTCCGAT	29764
29765	**ATTTACATTTCAGTATGAGTAGATTTTAAT**	AACGGCCATGAAAAGTAAGTGAATGACCTTGTAAG	29829
29830	**ATTTACATTTCAGTATGAGTAGATTTTAAT**	GGATTGACCTTGTCAGACTTCAATGACCTAACCGT	29894
29895	**ATTTACATTTCAGTATGAGTAGATTTTAAT**	CGTTCATATCTTTTTCAAAGTCGTTTGATGAGCTATT	29961
29962	**ATTTACATTTCAGTATGAGTAGATTTTAAT**		29991

F. magna **ACS-171-V-Col3: DR length: 36   Number of spacers: 14**

11866	**ATTTAATACCTATATGAAATTACATCATTCTCAAAC**	TGATTGAGCAAATAACGAAAGAGACAAAAG	11931
11932	**GTTTAATACCTATATGAAATTACATCATTCTCAAAC**	GGCGGCATGTTGTTTCTACTAAACGTAACA	11997
11998	**GTTTAATACCTATATGAAATTACATCATTCTCAAAC**	CATAATAGTACCTGTTAAAATCTTCTATAAA	12064
12065	**GTTTAATACCTATATGAAATTACATCATTCTCAAAC**	ATTTGATGTTTGATTGTTTCTTTATCGACA	12130
12131	**GTTTAATACCTATATGAAATTACATCATTCTCAAAC**	TCCCAAATCCGAACTTATCTCTCAGTACCA	12196
12197	**GTTTAATACCTATATGAAATTACATCATTCTCAAAC**	GCAAAAAGATAATAAAAAAATACCCCCGTG	12262
12263	**GTTTAATACCTATATGAAATTACATCATTCTCAAAC**	TAAAACATTTGAATACAACTACAGAGAGTG	12328
12329	**GTTTAATACCTATATGAAATTACATCATTCTCAAAC**	TCATCATAGTTGCAAATCCTCAATGTTCACA	12395
12396	**GTTTAATACCTATATGAAATTACATCATTCTCAAAC**	ATTGATTTCTGAAAATATTCTGTTCTAATT	12461
12462	**GTTTAATACCTATATGAAATTACATCATTCTCAAAC**	ACTGAAAAAGAGATTTCAGATTATATAGCA	12527
12528	**GTTTAATACCTATATGAAATTACATCATTCTCAAAC**	CTCTAGTTTGTTCGTTAGAAACACGTTCTT	12593
12594	**GTTTAATACCTATATGAAATTACATCATTCTCAAAC**	TAAGTTCAAGCACCGCAGGTTCTTTGTCCT	12659
12660	**GTTTAATACCTATATGAAATTACATCATTCTCAAAC**	TTAAAATGTAATGGTATGAATTTTCTAATT	12725
12726	**GTTTAATACCTATATGAAATTACATCATTCTCAAAC**	CCAACTATGCAAATGTTAGCTGATTTATAC	12791
12792	**GTTTAATACCTATATGAAATTACATCATTCTCAAAC**		12827

***F. magna* BVS033A4: DR length: 30** **Number of spacers: 38**

106	**CTTTACATCCCACTCTAGTTAGATTTTAAT**	ATTGTTTTCAAATACACACCACTTATGTTAAAGGCT	171
172	**ATTTACATTCCACTATGGGTAGATTTTAAT**	CCAGTTGCTACATTTGTTGCTATTACTTATTGGTA	236
237	**ATTTACATTCCACTATGGTTAGATTTTAAT**	AGTGTTTTGCGGCTTATAATGTTGGTGTTGTTGGTGT	302
303	**ATTTACATTCCACTATGGGTAGATTTTAAT**	TTAATTGAATAGAATACTTTATTCGGATATTTATC	367
368	**ATTTACATTCCACTATGGTTAGATTTTAAT**	AGCGTATTTCCAAGAAGTGCTGCACTTCCTGTGCGT	433
434	**ATTTACATTCCACTCTGGTTAGATTTTAAT**	CCTCAAAGAACTAATTCTTTTTCGCTCCCTTGCCTC	499
500	**ATTTACATTCCACTCTGGTTAGATTTTAAT**	AGGGATAAAGATGGCAAAAACATCAAGGTTCCGCT	564
565	**ATTTACATTCCACTCTGGTTAGATTTTAAT**	GGATTTTATGATTTAAAATTTATTGATGAAGAAGAT	630
631	**ATTTACATTCCACTCTGGTTAGATTTTAAT**	TCCAAGCCAAAAAAGCTCCAGCAATTCCGAGACCCAC	697
698	**ATTTACATTCCACTCTGGTTAGATTTTAAT**	ATGTGACCTGCCTTGATTCTTAGTTGCATTAGCTCT	763
764	**ATTTACATTCCACTCTGGTTAGATTTTAAT**	ACAATTTTTATTGAAGTTGAACCTATTAAATCAAAA	829
830	**ATTTACATTCCACTCTGGTTAGATTTTAAT**	TTTAATTTAAAAAAAATTCTCTCAGTTTTTCATC	893
894	**ATTTACATTCCACTCTGGTTAGATTTTAAT**	TCAATCGACAGTTCAGATAAAGATTACTTCGAGGAT	959
960	**ATTTACATTCCACTCTGGTTAGATTTTAAT**	AAAGCTCCAATCAGATTTTTACTGTCTCGATTAAGACC	1027
1028	**ATTTACATTCCACTCTGGTTAGATTTTAAT**	AAAAAGATGCAATCAGATTATCTTGCATAGTACAA	1092
1093	**ATTTACATTCCACTCTGGTTAGATTTTAAT**	CGAAGACGGTGAAATCTTGTTTAAAGATATTACTCT	1158
1159	**ATTTACATTCCACTCTGGTTAGATTTTAAT**	CAAGCGTAAGGAGGGCTCACAATGAAAGAAAAAT	1222
1223	**ATTTACATTCCACTCTGGTTAGATTTTAAT**	TTACTATTGTGATGATTTCCAGCTAATGGATTGTC	1287
1288	**ATTTACATTCCACTCTGGTTAGATTTTAAT**	TGTTTTATATCCAGTAACTAAATCTTTGATATCATG	1353
1354	**ATTTACATTCCACTCTGGTTAGATTTTAAT**	GTCAATCCTTGAGTTGAACCTTGCGTGAAGCTTA	1417
1418	**ATTTACATTCCACTCTGGTTAGATTTTAAT**	AAGCAAATTATACCAAAGTTCGACTTAGCTATAAA	1482
1483	**ATTTACATTCCACTCTGGTTAGATTTTAAT**	TGATGCAGTGGGAGGACAAATTGAACGTGTTAATAT	1548
1549	**ATTTACATTCCACTCTGGTTAGATTTTAAT**	CTTGTAATTGCACCTAAAAAAGTGGCAGAAGCTACT	1614
1615	**ATTTACATTCCACTCTGGTTAGATTTTAAT**	AAATCATAGTCACAATTTGATTTGATGTTAGAAAA	1679
1680	**ATTTACATTCCACTCTGGTTAGATTTTAAT**	TTTTAACTTTGCCTTGTGCTTCCTCTGCAAATCCCTC	1746
1747	**ATTTACATTCCACTCTGGTTAGATTTTAAT**	ATGTATTTTAGTACGTCATCGCTAAATGTTTTTTCTC	1813
1814	**ATTTACATTCCACTCTGGTTAGATTTTAAT**	TTAAACAGTTTTCTTCATAGCTAACATACTGTTTACT	1880
1881	**ATTTACATTCCACTCTGGTTAGATTTTAAT**	CGACAATATCTGTACCATTAGTCGGTACTATAGCAGC	1947
1948	**ATTTACATTCCACTCTGGTTAGATTTTAAT**	TATCAACTCATGTTCAATCATTGACATCTGATTATT	2013
2014	**ATTTACATTCCACTCTGGTTAGATTTTAAT**	AAAAGAATAAGTGCAAAATGAGTATTAATGAAACTAT	2080
2081	**ATTTACATTCCACTCTGGTTAGATTTTAAT**	TAGCATTCGGAACAGGAACTGGAAACTCTAATAGATT	2147
2148	**ATTTACATTCCACTCTGGTTAGATTTTAAT**	CTTACATGGTTCTTAAAAATTATCCAAATTTAAGT	2212
2213	**ATTTACATTCCACTCTGGTTAGATTTTAAT**	AAGAAAAGTTATTAAGAAGAGGTCGTAGTCTTTCTAC	2279
2280	**ATTTACATTCCACTCTGGTTAGATTTTAAT**	TTTTGTATTGATAATTTCATATTGTTGAAATCTTTAAT	2347
2348	**ATTTACATTCCACTCTGGTTAGATTTTAAT**	GAAGGGAAAGCAGGTAAGGTTTCAAGCATTCAAAG	2412
2413	**ATTTACATTCCACTCTGGTTAGATTTTAAT**	GGATTTGACGAAAATCCAACTTGGGAGGATGCGAC	2477
2478	**ATTTACATTCCACTCTGGTTAGATTTTAAT**	CCGTTAATTTCTTTATATTTTTGTATTAATGAAT	2541
2542	**ATTTACATTCCACTCTGGTTAGATTTTAAT**	TTTGTACTATATCCTCGAATAGTCAATTTAAAATT	2606
2607	**ATTTACATTCCACTCTGGTTAGATTTTAAT**		2636

F. magna **SY403409CC001050417: DR length: 36 Number of spacers: 11**

17675	**GTTTAATACCTATATGAAATTACATCATTTTCAAAC**	TCCATTTCTCCCATATCAGCATCTGCCAAT	17740
17741	**GTTTAATACCTATATGAAATTACATCATTCTCAAAC**	ACATTTTTTAATTTGAGAAATGCAGTTGAA	17806
17807	**GTTTAATACCTATATGAAATTACATCATTCTCAAAC**	TGTCCTTTTTCAGAATTATCTCTTTTTTCG	17872
17873	**GTTTAATACCTATATGAAATTACATCATTCTCAAAC**	TTTCTGTTAAGTCTTCATTGTCTAACAATT	17938
17939	**GTTTAATACCTATATGAAATTACATCATTCTCAAAC**	GAACGATAAGCTACCATATCCGTTTTTGTTC	18005
18006	**GTTTAATACCTATATGAAATTACATCATTCTCAAAC**	TGTCCTTTTTCAGAATTATCTCTTTTTTCG	18071
18072	**GTTTAATACCTATATGAAATTACATCATTCTCAAAC**	TTTCGCTTCGTACTTCTTTACCATCGAACA	18137
18138	**GTTTAATACCTATATGAAATTACATCATTCTCAAAC**	TGCTGTAAATACTATCGTACTCACTTTGTG	18203
18204	**GTTTAATACCTATATGAAATTACATCATTCTCAAAC**	ATCGATTTCGGCACTGATGTTCAAATAACT	18269
18270	**GTTTAATACCTATATGAAATTACATCATTCTCAAAC**	ACCTTTAGGACCAACAGGACCAGTTTTCCC	18335
18336	**GTTTAATACCTATATGAAATTACATCATTCTCAAAC**	TTAGCATGTAGTATCCACTGACGTAGTTTG	18401
18402	**GTTTAATACCTATATGAAATTACATCATTCTCAAAC**		18437

"*F. nericia*" 09T494: DR length: 30 Number of spacers: 34

36946	**ATTTACATTCCAATATGGGTGGATTTTAAT**	TTGGGCGTAGCTGGTGCTTTTTTAGCTTGGAAATT	37010
37011	**ATTTACATTCCACTTTGGGTAGATTTTAAT**	CAAGTCATATTCAAACTGGATCATGCGAAAAAGA	37074
37075	**ATTTACATTCCACTTTGGGTAGATTATTTT**	AATGAATCCTTGAGCGCCTTGAAGTCCGCGGTCTCCTT	37142
37143	**ATTTACATTTCAATATGAGTAGATTTTAAT**	AAGAGTTAAAAGAGGAGATGTGAGTCAATGTAGCTT	37208
37209	**ATTTACATTTCAGTATGATTAGATTTTAAT**	GGAGCAATACGAATTGCAAACTCAAAGAAGAGTGA	37273
37274	**ATTTACATTTCAGTATGAATAGTTTTAATG**	CTTCTGGACACCAACCTTTTAACCCTTTATTCCA	37337
37338	**ATTTACATTCCATTCTGGTTAGATTTTAAT**	GTCATGAGTGGACATATAACTTGAAGCTCAAAAA	37401
37402	**ATTTACATTCCACTCTGGTTAGATTTTAAT**	GAAGTTGTGGATAGCAGAAATCAAAGGGCATCTTA	37466
37467	**ATTTACATTCCACTCTGGGTAGATTTTAAT**	TAAATTGCTTTGTGGATATCAGAAAATGTTACGATA	37532
37533	**CTTTACATTCCATTCTGGTTAGATTTTAAT**	AGGAAGTTACCAGTTTAGGGAACAAGAATTTCACAT	37598
37599	**CTTTACATTCCATTCTGGTTAGATTTTAAT**	AAGTGGCAAAGACGGACAACTTCCATCTGGGTTAGT	37664
37665	**CTTTACATTCCATTCTGGTTAGATTTTAAT**	AAGTTAATCCCCCTTGACCATCTTCTTTATATAAAAA	37731
37732	**CTTTACATTCCATTCTGGTTAGATTTTAAT**	TTATCTTCAAGGTTTGAGGGGATATAATGCAAGTCT	37797
37798	**CTTTACATTCCATTCTGGTTAGATTTTAAT**	TTTACTTGAAATCTTTATCAACATCAATTAAATTAA	37863
37864	**CTTTACATTCCATTCTGGTTAGATTTTAAT**	TTCGTCTGAATGTGTTACCAACAATTGCAAAGATTT	37929
37930	**CTTTACATTCCATTCTGGTTAGATTTTAAT**	TTTAGGACTTGGTGCTAACATTATGAGTGGAATTTT	37995
37996	**CTTTACATTCCATTCTGGTTAGATTTTAAT**	GTTCCACTGAACGTACGTTCATTAACTGTATTAAGT	38061
38062	**CTTTACATTCCATTCTGGTTAGATTTTAAT**	TTTACTTGAAATCTTTATCAACATCAATTAAATTAA	38127
38128	**CTTTACATTCCATTCTGGTTAGATTTTAAT**	GAATAGAAATCAACAATTATTTCAAACAGAAAATAA	38193
38194	**CTTTACATTCCATTCTGGTTAGATTTTAAT**	TTCTCCAAAGTCAAAATTTTCAGTTTTGAAAATTGAT	38260
38261	**CTTTACATTCCATTCTGGTTAGATTTTAAT**	TCAATCATTTCGTAAAATTCAAATAATTTTAAAGT	38325

38326	**CTTTACATTCCATTCTGGTTAGATTTTAAT**	AACTTGAACAGAATATCCGTATCCCTCCTCTGTGAT	38391
38392	**CTTTACATTCCATTCTGGTTAGATTTTAAT**	CAAACAAGGTCAAAAGATAGGATTAAGTGGAAATAC	38457
38458	**CTTTACATTCCATTCTGGTTAGATTTTAAT**	TAACGGAAAAAATCAATTGGGAACAAGCCAAAAAATC	38523
38524	**CTTTACATTCCATTCTGGTTAGATTTTAAT**	TTCATTTGAGTGTGTTACCAATAATTGCAAAGATTT	38589
38590	**CTTTACATTCCATTCTGGTTAGATTTTAAT**	CTCTTTTTCTTTAGTGTAATCATATTCAAGTTTTCG	38655
38656	**CTTTACATTCCATTCTGGTTAGATTTTAAT**	TTAAATAGGACACAAAGAGATTATCTCACTACAAG	38720
38721	**CTTTACATTCCATTCTGGTTAGATTTTAAT**	CTTTTATACTAAAATCTTCAATATCAAAATATAT	38784
38785	**CTTTACATTCCATTCTGGTTAGATTTTAAT**	TTTGTCTTGTTCTTGTTTTTCTTCTTTTCGTAGATT	38850
38851	**CTTTACATTCCATTCTGGTTAGATTTTAAT**	TTTACCCCCATAATTTACATATGTTTTATCATAAAT	38916
38917	**CTTTACATTCCATTCTGGTTAGATTTTAAT**	CTCTTTTCCTTTACTATAACTTTATTATATCACCTT	38982
38983	**CTTTACATTCCATTCTGGTTAGATTTTAAT**	TCTAATTGCAAACGGTCTAAATTTTTTTGGTTTTGG	39048
39049	**CTTTACATTCCATTCTGGTTAGATTTTAAT**	CTTTGTTAAGCTTAAAGCTTGGAGAATATCTAATAAA	39115
39116	**CTTTACATTCCATTCTGGTTAGATTTTAAT**	AATAATTTTTTAATGTATAATGAACTTGATTATTAT	39181
39182	**CTTTACATTCCATTCTGGTTAGATTTTAAT**		39211

"*F. nericia*" 12T272: DR length: 30 Number of spacers: 41

68572	**ATTTACATTCCATTCTGGTTTGATTTTAAT**	TAAAAAATAGACAATGGCAAGAGGCAAGAGATCCTCA	68638
68639	**ATTTACATTCCATTTTGGTTTGATTTTGAT**	CAGTAAGTTGCGTTGTTAGTTACTTTTCTATGAAG	68703
68704	**ATTTAAATTTCATTATGAGTAGGTTTTAAT**	TGTCTTTTTAAGTCCATAGAACTCTGCTATCGTATATTTACATTC CACATAATATC	68789
68790	**ATTTACATTCCACTCTGGTTAGATTTTAAT**	TGAAGTATTGTATACTTTTAAATCTATTAGCTATCAC	68856
68857	**ATTTACATTCCACTTTGGTTAAATTTTAAT**	TATATTCTTGCCAAGTAAATACTTTAAAAGTTCAAA	68922
68923	**ATTCACATTCCATTCTGGTTAAATTTTAAT**	TCATAAAGAATTTACCAATATTTATATAATCATTA	68987
68988	**ATTTACATTCCACTCTGGTTAAATTTTAAT**	CCCTCATCTGTGATAGGGTAAATAAAATAACTGTA	69052
69053	**ATTTACATTCCACTCTGGTTAAATTTTAAT**	ATATTTACCACCATAATTTACATAAGTTTGATTATAGAT	69121
69122	**ATTTACATTCCACTCTGGTTAAATTTTAAT**	TCTACATTTAAAACCTCTTTATATATTTGCTCTTGTG	69188
69189	**ATTTACATTCCACTCTGGTTAAATTTTAAT**	ATGGTTATTGAAAAAGATGACGGTGGCTCTGGTGGT	69254
69255	**ATTTACATTCCACTCTGGTTAAATTTTAAT**	TGAGGATAAGGTAATGTCGAATTTAGTATTGATATT	69320
69321	**ATTTACATTCCACTCTGGTTAAATTTTAAT**	AACAATCGTGACGTACCCGCACAGCTTAGAGTGAA	69385
69386	**ATTTACATTCCACTCTGGTTAAATTTTAAT**	CCATATTCTGTCCCCTTCGCCATTATCCCAAGGAAG	69451
69452	**ATTTACATTCCACTCTGGTTAAATTTTAAT**	AATGAAAAAACAAAATCCTAGACGTTATCAAGTCGC	69517
69518	**ATTTACATTCCACTCTGGTTAAATTTTAAT**	TTGGAAGGTAAAAGCATTGCTAACATTACAAGAG	69581
69582	**ATTTACATTCCACTCTGGTTAAATTTTAAT**	AAAGCTAAAAGCGGAGCAATAACAGCCAAAATAAC	69646
69647	**ATTTACATTCCACTCTGGTTAAATTTTAAT**	TCTTTACCTGGCATACCATTTTCACCATCAGCACCAGG	69714
69715	**ATTTACATTCCACTCTGGTTAAATTTTAAT**	AATATAATCATAACTTGTTACTTTGTTAATTTCAAC	69780
69781	**ATTTACATTCCACTCTGGTTAAATTTTAAT**	TTTTTATATAAAGAAGACGGTCAAGGTGGTTTGAC	69845
69846	**ATTTACATTCCACTCTGGTTAAATTTTAAT**	CAAACTTTTAAAAAATTTGACCTTTTCGTATTGCCAT	69911

69912	**ATTTACATTCCACTCTGGTTAAATTTTAAT**	GTGTCTTGATAAATTTGTGCATAATCATTAACTAA	69976
69977	**ATTTACATTCCACTCTGGTTAAATTTTAAT**	TAAATTGTTTTATTATTTCATTAAATCCAGTTATTA	70042
70043	**ATTTACATTCCACTCTGGTTAAATTTTAAT**	TCACCTAAAAAATGTTATTTAAATCATCTATTCTT	70107
70108	**ATTTACATTCCACTCTGGTTAAATTTTAAT**	TATCAAGTTTTTTCATTGCCTATTTCCTTTTAGTAA	70173
70174	**ATTTACATTCCACTCTGGTTAAATTTTAAT**	TGGAACAAAAATTGGCAGTAGAAAATTTGACGGAACATTTAC	70245
70246	**ATTTACATTCCACTCTGGTTAAATTTTAAT**	TTTCCAGTTGGTAGTTTATAATTTAACATAATAGC	70310
70311	**ATTTACATTCCACTCTGGTTAAATTTTAAT**	CAATAAAATTGTCTATATGTGATATTTCGTTTACTAA	70377
70378	**ATTTACATTCCACTCTGGTTAAATTTTAAT**	ATTGTTATTAATTTCACCTTGCATTTTATCAATGAT	70443
70444	**ATTTACATTCCACTCTGGTTAAATTTTAAT**	TTATTTATAACAAGTTTTTTACTTAAATTTTCATA	70508
70509	**ATTTACATTCCACTCTGGTTAAATTTTAAT**	TAAGCCATTCTTTTAGGTCTTTGATGCCTCAAGAGA	70574
70575	**ATTTACATTCCACTCTGGTTAAATTTTAAT**	CTCATTATATGCAATGTCTATTTCTTGTTATGTAA	70639
70640	**ATTTACATTCCACTCTGGTTAAATTTTAAT**	CTGAACTCATAAATTGAAATAATAGGTCAACTTCGTT	70706
70707	**ATTTACATTCCACTCTGGTTAAATTTTAAT**	GTAGCTTTTCCATCTCTGTAGAGTTCTACTTCTATT	70772
70773	**ATTTACATTCCACTCTGGTTAAATTTTAAT**	CTTGGTTATTATCAGAAATACTTCCTCTAGCAACA	70837
70838	**ATTTACATTCCACTCTGGTTAAATTTTAAT**	TCATTTGCAATTGCTCAACTTTACGAAGAAAGTTTT	70903
70904	**ATTTACATTCCACTCTGGTTAAATTTTAAT**	TCAGTTTTAACAACTGCAAGAAAATCATCCCACATAAATTC	70974
70975	**ATTTACATTCCACTCTGGTTAAATTTTAAT**	AACATTTTCTCCAAGTAGATTGGGTCTATACTGTC	71039
71040	**ATTTACATTCCACTCTGGTTAAATTTTAAT**	GTTGTACTTTCTTCTACTGTATCAACAACTGTTTT	71104
71105	**ATTTACATTCCACTCTGGTTAAATTTTAAT**	CCCAATGTTTCGTGTGTCCTCCAACTGAATAATT	71168
71169	**ATTTACATTCCACTCTGGTTAAATTTTAAT**	ATTTATATTACCCTTGATTTCTTTATTTTGGAAAAC	71234
71235	**ATTTACATTCCACTCTGGTTAAATTTTAAT**	GTTGTACTTTCTTCTACTGTATCAACAACTGTTTT	71299
71300	**ATTTACATTCCACTCTGGTTAAATTTTAAT**		71329

"*F. nericia*" 12T273: none

"*F. nericia*" 12T306: DR length: 30 Number of spacers: 82

14108	**ATTAAAATCTATCCAGAGTGGAATGTAAAT**	TTGTCCCATATCGTTAATAAATGTTGAAAATTTTTG	14173
14174	**ATTAAAATCTATCCAGAGTGGAATGTAAAT**	GTACTAAAAAATGATGAAATAATGAGAATGGGATAC	14239
14240	**ATTAAAATCTATCCAGAGTGGAATGTAAAT**	CTTTGTTGTTGTTAATATCATTTTGCATTTTTTCAATAA	14308
14309	**ATTAAAATCTATCCAGAGTGGAATGTAAAT**	ATCTATTATTAGCAACTGTTAAAAGTTCTTCCTG	14372
14373	**ATTAAAATCTATCCAGAGTGGAATGTAAAT**	GAAACAAACAAAAATATTGTTTTTAATTTTGACAA	14437
14438	**ATTAAAATCTATCCAGAGTGGAATGTAAAT**	AACCTAACATTTGTTTGATAATTTGTATTAAAATAATC	14505
14506	**ATTAAAATCTATCCAGAGTGGAATGTAAAT**	AATGGTAATATTAGTGTCGATAATTTAAAGAAATTA	14571
14572	**ATTAAAATCTATCCAGAGTGGAATGTAAAT**	ACATAGTTACCTAGTTTTTCAATTTCTTTTAAATA	14636
14637	**ATTAAAATCTATCCAGAGTGGAATGTAAAT**	CTATATCAATTATATTATACATTATCTCAATTAACAAA	14704
14705	**ATTAAAATCTATCCAGAGTGGAATGTAAAT**	ACAGTAAATGTTTTTGAAAAACTTGAACTTTTACAACCA	14773
14774	**ATTAAAATCTATCCAGAGTGGAATGTAAAT**	AAATCAAGTGGTAATACAGATTGGCAAAAAGTTAAC	14839

14840	**ATTAAAATCTATCCAGAGTGGAATGTAAAT**	TTAGTAAATGAAATTAGTCACATAGATAATTTTGT	14904
14905	**ATTAAAATCTATCCAGAGTGGAATGTAAAT**	TTTAAATCTTTATAACAAGTATATAATACATTTATA	14970
14971	**ATTAAAATCTATCCAGAGTGGAATGTAAAT**	TACTACAAGTTAATGTATAATCTTGTATAGTTTATAG	15037
15038	**ATTAAAATCTATCCAGAGTGGAATGTAAAT**	ATGGCAATACGAAAAGGTCAAGTTTTCAAAAGTTTAAAT	15106
15107	**ATTAAAATCTATCCAGAGTGGAATGTAAAT**	TTTATTTAAGTGACCGTAAACTGATAAATATTTAT	15171
15172	**ATTAAAATCTATCCAGAGTGGAATGTAAAT**	GAACAACTTGAAAATGGATAATAAATATTACCAAATT	15238
15239	**ATTAAAATCTATCCAGAGTGGAATGTAAAT**	AATGGTAATATCAGTATCGATAATCTTAAAAAATTAA	15305
15306	**ATTAAAATCTATCCAGAGTGGAATGTAAAT**	TTACTCATTTTTTGAAAAGCTTGTAATTCTCTAAAC	15371
15372	**ATTAAAATCTATCCAGAGTGGAATGTAAAT**	TGAATATTAAACATATATAAAGTGAACCCATCTTTTAT	15439
15440	**ATTAAAATCTATCCAGAGTGGAATGTAAAT**	AAGAAATAATTTATTGAGGAATGGTGCTAAAAAAAGA	15506
15507	**ATTAAAATCTATCCAGAGTGGAATGTAAAT**	TTAGTAAACGAAATATCACATATAGACAATTTTAT	15571
15572	**ATTAAAATCTATCCAGAGTGGAATGTAAAT**	GATAGTATTGACCCTTTATATTTAGAAAAAATGTTAAA	15639
15640	**ATTAAAATCTATCCAGAGTGGAATGTAAAT**	ACAATAAAATTATTTGAATTTTATGATATGATTGA	15704
15705	**ATTAAAATCTATCCAGAGTGGAATGTAAAT**	ACGCCTCTTGCTCCAATAACCATATTTAACATAGA	15769
15770	**ATTAAAATCTATCCAGAGTGGAATGTAAAT**	CTAAATGTTCCGTCAAATTTACGACTTCCTAGTTTTGTT	15838
15839	**ATTAAAATCTATCCAGAGTGGAATGTAAAT**	GAATTTATGTGGGACGATTTCTTAGCAGTTGTTGA	15903
15904	**ATTAAAATCTATCCAGAGTGGAATGTAAAT**	ACACCAAAAGTAGCTTTTTTTTAAATCATATTCCC	15967
15968	**ATTAAAATCTATCCAGAGTGGAATGTAAAT**	TCTGGATTTGTTGGGTTAATATCGTCAGCTATTTCAC	16034
16035	**ATTAAAATCTATCCAGAGTGGAATGTAAAT**	ATTTATCTTGAAAAAAATGTTGAATTTAAATGGATAT	16100
16101	**ATTAAAATCTATCCAGAGTGGAATGTAAAT**	AATTTCCACGCCAAAAAAGCCCCTGCGATTCCAA	16164
16165	**ATTAAAATCTATCCAGAGTGGAATGTAAAT**	TTCCAATACCCAATAGTGAATTTGCAACGCCAAAAC	16230
16231	**ATTAAAATCTATCCAGAGTGGAATGTAAAT**	ATTTTAATTTGAATAGAGGGTAAATGTTCATCTTTAA	16297
16298	**ATTAAAATCTATCCAGAGTGGAATGTAAAT**	ATTTAATTATGTGTGCTACATCAACTATTTACTCATT	16364
16365	**ATTAAAATCTATCCAGAGTGGAATGTAAAT**	AGTTTTGTTCCAGATTTCATTACAAATACTAACCA	16429
16430	**ATTAAAATCTATCCAGAGTGGAATGTAAAT**	GTTTTAGCAAATCCTCCGTAAAATTGATAAAATTTTGTC	16498
16499	**ATTAAAATCTATCCAGAGTGGAATGTAAAT**	GAATATCAGCTAAAAGCGTCTAAGAAATGGTATAA	16563
16564	**ATTAAAATCTATCCAGAGTGGAATGTAAAT**	TAAATCTAAATAAATTTTCAAATTCATATACTCC	16627
16628	**ATTAAAATCTATCCAGAGTGGAATGTAAAT**	AAAGTTGAATATTATAACACGCTTGAGTTAATGAAC	16693
16694	**ATTAAAATCTATCCAGAGTGGAATGTAAAT**	ATATTAATGGTATATATTTGCTATTCACAACCTCTG	16759
16760	**ATTAAAATCTATCCAGAGTGGAATGTAAAT**	ATAAATCGTTATATTTTAACCCTAAATCAATACTTT	16825
16826	**ATTAAAATCTATCCAGAGTGGAATGTAAAT**	ATTGCACGTGAAATATTAGCACATAAGTTTTTCATC	16891
16892	**ATTAAAATCTATCCAGAGTGGAATGTAAAT**	CCTTTATAGTATAATTTTTATAAGTTAAAGTGAGTT	16957
16958	**ATTAAAATCTATCCAGAGTGGAATGTAAAT**	GAATTTTTGTTTTCGTTTTAAAATAATTTTAATTTA	17023
17024	**ATTAAAATCTATCCAGAGTGGAATGTAAAT**	ATAAGAAAAAATATTATACAACGTAATTATATCACG	17089
17090	**ATTAAAATCTATCCAGAGTGGAATGTAAAT**	GAATTTTTGTTTTCGTTTTAAAATAATTTTAATTTAG	17156
17157	**ATTAAAATCTATCCAGAGTGGAATGTAAAT**	CAACGAACAAAAAGAAGATGGAACAAGCAAGGTTAA	17222
17223	**ATTAAAATCTATCCAGAGTGGAATGTAAAT**	TTTTTTGGGAAAATGTGAATTTGTTTTGTTTTTTC	17287
17288	**ATTAAAATCTATCCAGAGTGGAATGTAAAT**	ATCTCTAGAATTAAACAAGATCTGACACTCATCAA	17352
17353	**ATTAAAATCTATCCAGAGTGGAATGTAAAT**	CAAGAAACACCATTTGGAAATAGTTATAGACGTTA	17417
17418	**ATTAAAATCTATCCAGAGTGGAATGTAAAT**	TAATTAGATACCACCTATCATATCATTTTCTTTATT	17483

17484	**ATTAAAATCTATCCAGAGTGGAATGTAAAT**	ATGAATATAAAGATTATAAAATAGTAATAAGAGAT	17548
17549	**ATTAAAATCTATCCAGAGTGGAATGTAAAT**	ATTTAACCCTAATACTTTATCTTATAACTATTAT	17612
17613	**ATTAAAATCTATCCAGAGTGGAATGTAAAT**	ATCGTTGCCCTTACGTGTTTTTGACAAGTGAAGAAA	17678
17679	**ATTAAAATCTATCCAGAGTGGAATGTAAAT**	GACGAAAAATACAAAGGTATTATATTTACTACTA	17742
17743	**ATTAAAATCTATCCAGAGTGGAATGTAAAT**	ATATATAATAAAACATATGTCAACTATGGGGGCAA	17807
17808	**ATTAAAATCTATCCAGAGTGGAATGTAAAT**	GAATTTTCGACTCTCATAATCTCTTTTAAAGTCTCAT	17874
17875	**ATTAAAATCTATCCAGAGTGGAATGTAAAT**	TTGTAAGTGTGTCCAGCTTTTAAGATATATTGAAATAA	17942
17943	**ATTAAAATCTATCCAGAGTGGAATGTAAAT**	TTTGTTATTAAATTTGTGGCGTAACTATGTCTTAA	18007
18008	**ATTAAAATCTATCCAGAGTGGAATGTAAAT**	CTTAAAAGACATTAAAGAGGGATTAATAATAGATGG	18073
18074	**ATTAAAATCTATCCAGAGTGGAATGTAAAT**	ATTAATGACATAATCAAATTTACACGAAGAACGTT	18138
18139	**ATTAAAATCTATCCAGAGTGGAATGTAAAT**	TTAAAAGTGAATGAAATGTTTGTGTTGCAAGATAT	18203
18204	**ATTAAAATCTATCCAGAGTGGAATGTAAAT**	ATGTTTTAATAGCGAATTCAAAAAGTTGTTTGTA	18267
18268	**ATTAAAATCTATCCAGAGTGGAATGTAAAT**	ACATATTTAAAAGTAAATAAGCAATTCTGTGAAAGA	18333
18334	**ATTAAAATCTATCCAGAGTGGAATGTAAAT**	GCTATTATGCTAAATTATCCTTTACCTTGTGGATA	18398
18399	**ATTAAAATCTATCCAGAGTGGAATGTAAAT**	ATAGACTCTTTTAAATACCTTGAAATATCTTCATT	18463
18464	**ATTAAAATCTATCCAGAGTGGAATGTAAAT**	TCCAAAACTCGTTTTGAAAATCAAGCAGGTGTCCCT	18529
18530	**ATTAAAATCTATCCAGAGTGGAATGTAAAT**	AGAACAGTTGTTGATACAGTAGAAGAAAGTACAAC	18594
18595	**ATTAAAATCTATCCAGAGTGGAATGTAAAT**	ATCATTGATAAAATGCAAAATGAAATTAATAACAA	18659
18660	**ATTAAAATCTATCCAGAGTGGAATGTAAAT**	ACAAATCCTGATGGCTCTTGTCCATCATTACCAC	18723
18724	**ATTAAAATCTATCCAGAGTGGAATGTAAAT**	AAAGACAGTAACACGATTTATTTCATTACGGAGTAG	18789
18790	**ATTAAAATCTATCCAGAGTGGAATGTAAAT**	ATTTTTGATTTCACATGTCATGGCAAGGTTACAAT	18854
18855	**ATTAAAATCTATCCAGAGTGGAATGTAAAT**	ATTTCTCCGACTTCCAATGTTGGTTGCTTACGAATAA	18921
18922	**ATTAAAATTTAACCAGAGTGGAATGTAAAT**	TTACACAGAAAAGTGACTAACAATGCGACTGACTGG	18987
18988	**ATTAAAATTTAACCAGAGTGGAATGTAAAT**	AAGAACAATTGTGTCGGAATAGGTAAGTTTCCTGATA	19054
19055	**ATTAAAATTTAACCAGAGTGGAATGTAAAT**	GATGGAGTGGAGGCTTTTATGTTTTTGATATATCTAT	19121
19122	**ATTAAAATTTAACCAGAGTGGAATGTAAAT**	ATTTAAACTAAAGGAAGTGAGGATATGCAACAAAC	19186
19187	**ATTAAAATTTAACCAGAATGGAATGTAAAT**	GTTGGCTTGGGAATTGCAGGTGCTTTTTTGGCTTGGAA	19254
19255	**ATTAAAATCTACCCAGAGTGGAATGTAAAT**	TTCTCAACATCAATCAAATTCACTGTAGCTTTGA	19318
19319	**ATTAAAATCTACCCAGAGTGGAATGTAAAT**	GGGAAAACTGGTGCAACTGGACCATAAGGACCACCAGGG	19387
19388	**ATTAAAATTTAACCATAGTGGAATGTAAAT**	AGTACTACATCTTCTACATCATAAAAATCTTTTGC	19452
19453	**ATTAAAATCTACTCATACTGAAATGTAAAT**	ACGATTAGTGGTATTGATAAGGATATGGGAGTAGT	19517
19518	**ATTAAAATTTAACCAAAGTGGAATGTAAAT**		19547

"*F. nericia*" CCUG54800: none

"*F. nericia*" T151023: DR length: 30 **Number of spacers: 61**

11514	**ATTTACATTCCAATATGGGTAAATTTTAAT**	CACAATATGAATTAGATCCTTTACTAGGTACAAGC	11578
11579	**ATTTACATTCCACTCTGGGTAAATTTTAAT**	CGTCGTTTGCTATCATAATAGCCCAATCTACATA	11642
11643	**TTTTACATTCCATTCTGGTTAAACTTTAAT**	ACAACACCGAACGCATTGTTCTTTTTTATGTCTCG	11707
11708	**ATTTACATTCCATTCTGGTTAAATTTTAAT**	AATAAGAGGTGTTAAATAGTGATATATCTTTTTAC	11772
11773	**ATTTACATTCCACTCTGGTTAAATTTTAAT**	ATATGAACAAGTATTACAGAAAACACAAAACAATGGT	11839
11840	**ATTTACATTCCATTCTGGTTAAATTTTAAT**	GACATAAAGGCAACGCTCGGAACAGTCGCTCCAGT	11904
11905	**ATTTACATTCCAATCTGGGTAAATTTTAAC**	GATATGGCTGAAAAGTATTTGGGAAATGGCAGACGA	11970
11971	**ATTTACATTTCAATATGAGTAGATTTTAAT**	GTAAATGATTGATAAGTAGTATCTAAAATATCATC	12035
12036	**CTTTACATTTCAATATGAGTAGATTTTAAT**	GCAATTATCCAGTCGCCTGGCTTGATGTCAATCTC	12100
12101	**TTTTACATTTCAATATGAGTAGATTTTAAT**	CTGATACGGGCACTTTTCTTTTCAGACTTCCAACGC	12166
12167	**TTTTACATTTCAGTATGAGTAGATTTTAAT**	TAATAAATTAATCCGCCAATACCTGCGAATATCAAA	12232
12233	**ATTTACATTTCAGTATGAGTAGATTTTAAT**	TACAGATAGTTCTAGTAATTCTCAAGTATTAAATTATAC	12301
12302	**ATTTACATTTCAGTATGAGTAGATTTTAAT**	CTTGTGCTATGATATAAGCTTTTTGGTATTCTTCATCA	12369
12370	**ATTTACATTTCAGTATGAGTAGATTTTAAT**	TAGAATAGAGGTAATAATATGATATATCTTTTTAC	12434
12435	**ATTTACATTTCAGTATGAGTAGATTTTAAT**	TTTAGCATGATTTCATCAAGTGTTGCATTTTTGCT	12499
12500	**ATTTACATTTCAGTATGAGTAGATTTTAAT**	GCAAGTGCTGAAGAAATCGCAGGAGTTGCAGAAGCGGC	12567
12568	**ATTTACATTTCAGTATGAGTAGATTTTAAT**	GAATTTTTGTACAAGTATTCGACAATATCGTATACCCT	12635
12636	**ATTTACATTTCAGTATGAGTAGATTTTAAT**	TACTACAAGATAAAAACGCAAAAAGTTTTCTGAACT	12701
12702	**ATTTACATTTCAGTATGAGTAGATTTTAAT**	AAGACAGTAACACGATTTATTTCATAACGGAGTAGT	12767
12768	**ATTTACATTTCAGTATGAGTAGATTTTAAT**	GGACTTGAAAAAGAGTTGACGTATTCAAGCACTAG	12832
12833	**ATTTACATTTCAGTATGAGTAGATTTTAAT**	AAACTAACAAGGTTGTCACTCTCAAGTGCAAGTGA	12897
12898	**ATTTACATTTCAGTATGAGTAGATTTTAAT**	GAGGTTAGATTTATGTTTAAGATTGAAAGAAAAGA	12962
12963	**ATTTACATTTCAGTATGAGTAGATTTTAAT**	TGAAGAAGATCATTACATCAAGCCAATCCAAAAAT	13027
13028	**ATTTACATTTCAGTATGAGTAGATTTTAAT**	GAGGTTAGATTTATGTTTAAGATTGAAAGAAAAGA	13092
13093	**ATTTACATTTCAGTATGAGTAGATTTTAAT**	CTTATTTGCATAAGTGTTCAATATCTTACCTACATAG	13159
13160	**ATTTACATTTCAGTATGAGTAGATTTTAAT**	CAAATAAACTTGTGAATTTGTAAGAGCCTTGTTCTTC	13226
13227	**ATTTACATTTCAGTATGAGTAGATTTTAAT**	CTCAATACTCAATGGGAGATGCAATGACTGCATCATC	13293
13294	**ATTTACATTTCAGTATGAGTAGATTTTAAT**	GATTGGATTAAATTCTTTACTCAGCACAGAAAAT	13357
13358	**ATTTACATTTCAGTATGAGTAGATTTTAAT**	AACAGATAATACCATGTTTTAAGTTACAGATCAA	13421
13422	**ATTTACATTTCAGTATGAGTAGATTTTAAT**	GTAGTTTCCCAAAAACTCGTCTATGTTTGCAAATGA	13487
13488	**ATTTACATTTCAGTATGAGTAGATTTTAAT**	CAGCCTTTCTGGCTATTAACTTTTGATTGTTGGA	13551
13552	**ATTTACATTTCAGTATGAGTAGATTTTAAT**	GCAATGAGTTCAGTAGGTATTGAAGCTGAAGCAGG	13616
13617	**ATTTACATTTCAGTATGAGTAGATTTTAAT**	TACCATCAAGTTACGAAGTCTCCAATGTAGTCGCTC	13682
13683	**ATTTATATTTCAGTATGAGTAGATTTTAAT**	CGTCTGAATCCCTTTGATTTATTGATAAAGAGTTG	13747
13748	**ATTTACATTTCAGTATGAGTAGATTTTAAT**	GAAAAGATATGGGTTCTATATATTGTTTCCGGGGGT	13813
13814	**ATTTACATTTCAGTATGAGTAGATTTTAAT**	AAATAATCAGTTTTCATTGTTCGAAGGTTTAATTTT	13879
13880	**ATTTACATTTCAGTATGAGTAGATTTTAAT**	CAGATAGTGTTTCTACATCTGTTTCTCAAAGCAT	13943
13944	**ATTTACATTTCAGTATGAGTAGATTTTAAT**	GTCAATTCCTTCATGAATTGCAAGTGGCGCTTCATA	14009
14010	**ATTTACATTTCAGTATGAGTAGATTTTAAT**	TGAGGATATGAGGGTCAAAGGAATTTCTGATTTAAC	14075

14076	**ATTTACATTTCAGTATGAGTAGATTTTAAT**	GCTGTGAGGTTTGTTGTTTCGCCCATGTCTATCA	14139
14140	**ATTTACATTTCAGTATGAGTAGATTTTAAT**	CGAGACACTGGTGGTCGTGGTGAAGTTACAATCAA	14204
14205	**ATTTACATTTCAGTATGAGTAGATTTTAAT**	ACACGAAGAGCCTAGTTTATAGGCTCTTTTTCTTAGG	14271
14272	**ATTTACATTTCAGTATGAGTAGATTTTAAT**	AGATATTATCATCATAATCGTCATCTTCCACGATGT	14337
14338	**ATTTACATTTCAGTATGAGTAGATTTTAAT**	ATGGCTGCTGTGGATACATTTTTGGGTACTATATT	14402
14403	**ATTTACATTTCAGTATGAGTAGATTTTAAT**	TATACAATATTTTGTAGATTTTGCAGTAAGTTATCT	14468
14469	**ATTTACATTTCAGTATGAGTAGATTTTAAT**	TGTTTAATTCTCGAGACTGGCAAGCATGTAAAAAC	14533
14534	**ATTTACATTTCAGTATGAGTAGATTTTAAT**	GCATAGAATAGAGGGAAAGATGATTTATTTATTTAC	14599
14600	**ATTTACATTTCAGTATGAGTAGATTTTAAT**	TTTAATAATCGTCTTCTTTTTAATTGTTATGAAAA	14664
14665	**ATTTACATTTCAGTATGAGTAGATTTTAAT**	CCCTGCTGCAATTAAAGACCAACTCACATCTTCTGC	14730
14731	**ATTTACATTTCAGTATGAGTAGATTTTAAT**	TTATTAACTTGATTGTGTAAGTTCTTTAACAGA	14793
14794	**ATTTACATTTCAGTATGAGTAGATTTTAAT**	GTGTGAAAGGAAGTTAATATGGAGAATTTATTAAA	14858
14859	**ATTTACATTTCAGTATGAGTAGATTTTAAT**	CCAACATCAGTTCTTTTAATCATTTGCTTGAACTG	14923
14924	**ATTTACATTTCAGTATGAGTAGATTTTAAT**	TGGCATTTGTTATAAGTTCGTGAGTCCTGGAAACTC	14989
14990	**ATTTACATTTCAGTATGAGTAGATTTTAAT**	GATGGATAAATTGTCCTGGTAGTGTGAGACTCGAAA	15055
15056	**ATTTACATTTCAGTATGAGTAGATTTTAAT**	TCACCTGATCATTTAGTTTTATATTATGATAGTAA	15120
15121	**ATTTACATTTCAGTATGAGTAGATTTTAAT**	AATTAGTAGTACTTTAGATAATAAACTACGAAATA	15185
15186	**ATTTACATTTCAGTATGAGTAGATTTTAAT**	TGCCTATATCTGAAATGGTGGAAATTTTGATTTTA	15250
15251	**ATTTACATTTCAGTATGAGTAGATTTTAAT**	TCTTCGGTGGTGGATTCAAAGAAAAACTATCGGCTAT	15317
15318	**ATTTACATTTCAGTATGAGTAGATTTTAAT**	TTGGAAAGCTTGGCAAGATTAAAGATGAAATTAA	15381
15382	**ATTTACATTTCAGTATGAGTAGATTTTAAT**	ATATGTTGGACAAAGGACACACTTTTGATTATATCTT	15448
15449	**ATTTACATTTCAGTATGAGTAGATTTTAAT**	TCGGCTTTGGATAAAACATTTCTCGGTTATATAAAC	15514
15515	**ATTTACATTTCAGTATGAGTAGATTTTAAT**		15544

"*F. nericia*" T160124: DR length: 31 **Number of spacers: 14**

49457	**GATTTACATTCCACTCTGGGTAGATTTTAAT**	AATAGTTGCGCCAACATACTCAACATTACTAATAC	49522
49523	**TATTTACATTCCACTCTGGGTAGATTTTAAT**	TTACTAGAACTATCTGTAACATAATTGTCAAGAT	49587
49588	**CATTTACATTCCATTCTGGTTAAATTTTAAT**	CATTTCCTGCTGCTGCTTGATTATCAGGTCTATAG	49653
49654	**TATTTACATTCCACTCTGGTTAAATTTTAAT**	GTTATAATAAATCCAGCTCTATCTTCCCTCCTATATG	49721
49722	**ACTTTACATTCCACTCTGGTTAAATTTTAAT**	TCAAATTGGATCATGGTTTGGAAGTAAATTCAAAA	49787
49788	**CCTTTACATTCCACTCTGGTTAAATTTTAAT**	CCGATTAATTCAAGTCCTTTGTTTACCTCTTTTG	49852
49853	**TATTTACATTCCAATATGGTTAGATTTTAAT**	AATGATATGCGGTGTACAAAGATGACAAGTTTGAGT	49919
49920	**TATTTACATTCCAATATGGTTAGATTTTAAT**	GAGAGGCGAAGAAAAGGAAGAGGTCCTTGTAAATG	49985
49986	**TATTTACATTCCAATATGGTTAGATTTTAAT**	TCGCTTTCGCAGTATCCACAGTCAAGTATGCTCCAA	50052
50053	**TATTTACATTCCAATATGGTTAGATTTTAAT**	CTAATTCTTCAAGTATTTCTGGGAGCTGTTCGA	50116
50117	**GATTTACATTCCAATATGGTTAGATTTTAAT**	TTCATGAGTGCAGCACATGTGTCACGAGTACTGT	50181
50182	**AATTTACATTCCAATATAGTTAGATTTTAAT**	GACAAAAACGCAAAAAGCTTTTTGAATCTAGATA	50246

50247	**CATTTACATTCCAATATGGTTAGATTTTAAT**	AATACACGTCCGATGTAGTTTTCTTCGGCGAAAA	50311
50312	**TATTTACATTCCAATATGGTTAGATTTTAAT**	AGAGCACCTTATAAGTTGATACTCAATTCAACTT	50376
50377	**AATTTACATTCCAATATGGTTAGATTTTAAT**		50407

"*F. nericia*" ATCC53516: DR length: 30 Number of spacers: 87

985861	**ATTTAAATTCCATTCTGGTAAGATTTTAAT**	CAATTGTAAACAGACTACAACGTATAGCTTTGATAA	985926
985927	**ATTTACATTCCACTCTGGGTAGATTTTAAT**	CCAATTTTTGTTTACCTTTTTCAAAAAAGTTACCTTT	985993
985994	**ATTTACATTCCACTCTGGATAGATTTTAAT**	CTGGACAAGATAAGTTAACCATATTAGCAGAAATATT	986060
986061	**ATTTACATTCCACTATGGTTAAATTTTAAT**	AACAGTGGATCTTTTTTCTTTACTTTTGTAAACAT	986125
986126	**ATTTACATTTCAGTATGAGTAGATTTTAAT**	TCCACCAAATACAACGCAGTGTCCCTCTTTTTCTTC	986191
986192	**ATTTACATTTCAGTATGAATAGATTTTAAT**	AGCGTATCAAGAAATACGATATTTGGATAGCTCAC	986256
986257	**ATTTACATTTCAGTATGAGTAGATTTTAAT**	TAGCGCGGGTAAGGCTATAATTGATGGATTCTTAAA	986322
986323	**ATTTACAATTCAGTATGAGTAGATTTTAAT**	CCTAAATTCTAACCCGTCAACGGTAACCGCCTTTAT	986388
986389	**ATTTACATTTCAGTATGAGTAGATTTTAAT**	AGACACTTGAGAAAAAATCAAGTGAAAGGAGAAATA	986454
986455	**ATTTACATTCCACTCTGGGTAGATTTTAAT**	ATTAATCGTGAAATTGACAAAGGCAAAACATTAAC	986519
986520	**ATTTACATTCCACTCTGGGTAGATTTTAAT**	TCTGTACTATAACCCCTGATCTGTAATTTAAAATT	986584
986585	**ATTTACATTCCACTCTGGGTAGATTTTAAT**	GGAATTGGAATGGGTAATATTCTAGGTGGAACACT	986649
986650	**ATTTACATTCCACTCTGGGTAGATTTTAAT**	TGATAGCAACGACTTGCAGAATATCACAATTGTGCA	986715
986716	**ATTTACATTCCACTCTGGGTAGATTTTAAT**	TAGGTCTTATTGTTCTAGAGATATCAGAATCGATATC	986782
986783	**ATTTACATTCCACTCTGGATAGATTTTAAT**	GTTTCGATACCTAATCCAACTACGCCGATGTTGTC	986847
986848	**ATTTACATTCCACTGTGGTTAAATTTTAAT**	TTTAGGAACAGTAGCACCTGTCGGCTTGGGAATTGC	986913
986914	**ATTTACATTTCAGTATGAGTAGATTTTAAT**	CAATTTGCAATACTAAGGCTTGGCAGCGGCTATGG	986978
986979	**ATTTACATTTCAGTATGAATAGATTTTAAT**	GCATAAGCTTCAGAATCCTCATAGTCTTCCAATGT	987043
987044	**CTTTACATTTCAGTATGAATAGATTTTAAT**	TCCAATCGCAACTTTTGTTGCGATCACGTATTGGTA	987109
987110	**CTTTACATTTCAGTATGAATAGATTTTAAT**	AACGAATATGAGATGGACAAAAAAGACGATGAGAA	987174
987175	**CTTTACATTTCAGTATGAATAGATTTTAAT**	TGCGTTAAAGCAATTTGCTATTACTTCTTCTTTGGA	987240
987241	**CTTTACATTTCAGTATGAATAGATTTTAAT**	ATTGAAGTAATCTTGTATGTTTTCTTGAGCTAATAT	987306
987307	**CTTTACATTTCAGTATGAATAGATTTTAAT**	GGACGAATTGTCAATATGCAATTAGCACTATATCAGA	987373
987374	**CTTTACATTTCAGTATGAATAGATTTTAAT**	GCCAAGGCTGTAATAAAAAAGGACGGTCTCTCAT	987437
987438	**CTTTACATTTCAGTATGAATAGATTTTAAT**	TCAATGAATCATCTAGCCCTTTTATATCCACTCTAA	987503
987504	**CTTTACATTTCAGTATGAATAGATTTTAAT**	GATATGAGCGATGAAAACTTCGGAGCAAATTTGAG	987568
987569	**CTTTACATTTCAGTATGAATAGATTTTAAT**	GGTTTTAGTTATGATTTATCTTTTTACTGGTACTCC	987634
987635	**CTTTACATTTCAGTATGAATAGATTTTAAT**	AATGGAAATTTTTCTTTTATAATCAAATTTTTTA	987698
987699	**ATTTACATTTCAGTATGAATAGATTTTAAT**	GCAGTGTTTCTTATACCTCTGCATACTCGGCTAACGT	987765
987766	**ATTTACATTTCAGTATGAATAGATTTTAAT**	GTTTTTGTGCTTTAATTTCAACAGTATCACATGCTAG	987832
987833	**ATTTACATTCCAGTATGATTAGATTTTAAT**	TTCTGATAGTGTTAGTACTTCACTTAGTAAAAGTAT	987898
987899	**ATTTACATTCCAGTATGATTAGATTTTAAT**	AGTGTCGTAAATAGTAGAGAAGCAATATTAAAGTT	987963

987964	**ATTTACATTCCAGTATGATTAGATTTTAAT**	ATAAACTCAGAATTTTTTACTTATAAAAAAAAGCT	988028
988029	**ATTTACATTCCAGTATGATTAGATTTTAAT**	GGTTTAAATTATGCTTTGAGATTATT	988084
988085	**TAAAACATTCCAGTATGATTAGATTTTAAT**	TTTTTGTGCTTTACTTGCTACAGTAAGCCATGCGAG	988150
988151	**ATTTACATTCCATTCTGGTTGGATTTTAAT**	AATGATTGGTGGAGGAGTTCTATTAATCGTAATGGC	988216
988217	**ATTTACATTCCATTCTGGTTGGATTTTAAT**	TCCTACAAAAAAAGTTGGTAGCACATCAACATTTTGT	988282
988283	**ATTTACATTCCATTCTGGTTGGATTTTAAT**	CTTTTTTAAGTGCAGATTGATATTCCTTCGGAACGTC	988349
988350	**ATTTACATTCCATTCTGGTTGGATTTTAAT**	TGCGACTGGTGCAGGATATTTCTATGAGTTGAACTC	988415
988416	**ATTTACATTCCATTCTGGTTGGATTTTAAT**	TATCAACATTAAAAGCGTGTTCGCTTACTACTTTTAG	988482
988483	**ATTTACATTCCATTCTGGTTGGATTTTAAT**	TATAATAGTTTGATAAAATTTCAAGAGGAGTATTCAA	988549
988550	**ATTTACATTCCATTCTGGTTGGATTTTAAT**	AATAAGTGATTCCGCAATGAAAGTCAGTGAGACAAC	988615
988616	**ATTTACATTCCATTCTGGTTGGATTTTAAT**	TTCTGATAATTTTACTTTATGTTGTTTCATAAAGGC	988681
988682	**ATTTACATTCCACTCTGGGTAGATTTTAAT**	TAATTTTTGCAAGTTGGTCTAAGTCTTTGGAATTTTT	988748
988749	**ATTTACATTCCACTCTGGGTAGATTTTAAT**	TATAAAAAACTTTAAATACATCATAAGAATAAAAA	988813
988814	**ATTTACATTCCACTCTGGGTAGATTTTAAT**	AGAATAGGAGGAAAACTTTGATATATCTTTTCAC	988877
988878	**ATTTACATTCCACTCTGGGTAGATTTTAAT**	ATGTGAACCTACCATCATCTTCTACGTCATAAGCCTC	988944
988945	**ATTTACATTCCACTCTGGGTAGATTTTAAT**	AAAGAAATACAGCCGTTAAAGTATGATTGGGAGTATA	989011
989012	**ATTTACATTCCACTCTGGGTAGATTTTAAT**	CATCCATCATTTGTTGAATTTTCAAAGCATTGATA	989076
989077	**ATTTACATTCCACTCTGGGTAGATTTTAAT**	TCCGTTTAGTCGATTAAAAGTAAACTCGCTGTTTTC	989142
989143	**ATTTACATTCCACTCTGGGTAGATTTTAAT**	TTCATCTTTTTCTTTTTTTAACTTATCCTTATAGT	989207
989208	**ATTTACATTCCACTCTGGGTAGATTTTAAT**	ATGTTTAGGTGGAGATAAATTTTCAGTTTATAATAA	989273
989274	**ATTTACATTCCACTCTGGGTAGATTTTAAT**	TTATAATTTTAAAAGCACATTTCTCTCAATCTTAGGT	989340
989341	**ATTTACATTCCACTCTGGGTAGATTTTAAT**	GTGAGGTTTTGTATGATTTATTTATTTACTGGAACTCC	989408
989409	**ATTTACATTCCACTCTGGGTAGATTTTAAT**	TCTGATGGAGGAGATAATGATATTTATTATTCTGA	989473
989474	**ATTTACATTCCACTCTGGGTAGATTTTAAT**	CCAAAGCGGCATTTAAAGACTTAAAAGGAGAATC	989537
989538	**ATTTACATTCCACTCTGGGTAGATTTTAAT**	AAAGAATCTCTTCGCATTTTCAAGTTAGAACAAAC	989602
989603	**ATTTACATTCCACTCTGGGTAGATTTTAAT**	CAAGCTAAAAAAGCTCCAGCAATTCCAAGTCCCAC	989667
989668	**ATTTACATTCCACTCTGGGTAGATTTTAAT**	CACATCTGCAAGATTTTATCTCTTGATAAATAATATC	989734
989735	**ATTTACATTCCACTCTGGGTAGATTTTAAT**	CCCATAAAACTAAAATTTCAACCATCTCAGATAT	989798
989799	**ATTTACATTCCACTCTGGGTAGATTTTAAT**	CCAAGCCAAGAAAGCCCCAGCAATTCCGAGTCCGAC	989864
989865	**ATTTACATTCCACTCTGGGTAGATTTTAAT**	AAATGAATCTCTTCGCATTTTAAGATTAGAACAAAC	989930
989931	**ATTTACATTCCACTCTGGGTAGATTTTAAT**	ATATCATACGGCGATAATGCTAGAGTTACTGAGCT	989995
989996	**ATTTACATTCCACTCTGGGTAGATTTTAAT**	GATGCAGTCGCGAGTTCAATAAAAAATTTCGGATTTA	990062
990063	**ATTTACATTCCACTCTGGGTAGATTTTAAT**	AGCAAGAAATGTCATTGGCTCACTTGGATTAAGTGT	990128
990129	**ATTTACATTCCACTCTGGGTAGATTTTAAT**	TTTACCGCATTTTGGAATGCTTGACCTACAGCCTG	990193
990194	**ATTTACATTCCACTCTGGGTAGATTTTAAT**	TTTTTCCATGTAATCATTTAACGTCTCTGCTGAATA	990259
990260	**ATTTACATTCCACTCTGGGTAGATTTTAAT**	AAACTCAAATTATCCCGATAAGTCATTCTTTACAAA	990325
990326	**ATTTACATTCCACTCTGGGTAGATTTTAAT**	CAATGAAAGAGAAAAAACCGTTCTATAAGAAAGTA	990390
990391	**ATTTACATTCCACTCTGGGTAGATTTTAAT**	ACGCTCTTTTTTATCTAAAATTGCGTTGAAATAAAT	990456
990457	**ATTTACATTCCACTCTGGGTAGATTTTAAT**	TACTACATCAACAGAAAAAGATTACAAAAATATTTGG	990523
990524	**ATTTACATTCCACTCTGGGTAGATTTTAAT**	TCCCTGCGTTGTTTACGTCTGAGTAAGGTACTCCG	990588

990589	**ATTTACATTCCACTCTGGGTAGATTTTAAT**	TTGAATAATATTTTTAAAGTGTTCAATCTTTTTGAT	990654
990655	**ATTTACATTCCACTCTGGGTAGATTTTAAT**	TTAACAGTTGGCGAAAAAAATACTATCGAAATTG	990718
990719	**ATTTACATTCCACTCTGGGTAGATTTTAAT**	TTAGATGTTAGTTACAACACATTAGCAAGCTATCGAACAA	990788
990789	**ATTTACATTCCACTCTGGGTAGATTTTAAT**	AATGAATCTCTACGCATTTTAAGATTTGAACATAT	990853
990854	**ATTTACATTCCACTCTGGGTAGATTTTAAT**	TGAATTTGTTGTATCTGAAACATAATTATCTAAATT	990919
990920	**ATTTACATTCCACTCTGGGTAGATTTTAAT**	AAGTCTTCATGGCAGTATAAGACGGAAGACGAACAA	990985
990986	**ATTTACATTCCACTCTGGGTAGATTTTAAT**	GATTTCATCAAAGATAAAGTATCTTTCAAATCAAA	991050
991051	**ATTTACATTCCACTCTGGGTAGATTTTAAT**	AATCAAAAAGCATTTTTTAACAACACGTTTGATGT	991115
991116	**ATTTACATTCCACTCTGGGTAGATTTTAAT**	GCACAACCTGAGGATTACTCTGTAAAAGCTAGAAAA	991181
991182	**ATTTACATTCCACTCTGGGTAGATTTTAAT**	TCATAATTAAATCTATCCATGCCGATCGCTTATCAAA	991248
991249	**ATTTACATTCCACTCTGGGTAGATTTTAAT**	TCAAGCTTTTTGCCAAGAAGTCTTTCCTGCTCTG	991312
991313	**ATTTACATTCCACTCTGGGTAGATTTTAAT**	CTCTACTTGCACCAGCAGCATTTAATCTTAATTT	991376
991377	**ATTTACATTCCACTCTGGGTAGATTTTAAT**	GTATGATCCAATGAGTTCTCTTTTACGAGTTCCGT	991441
991442	**ATTTACATTCCACTCTGGGTAGATTTTAAT**	TAGAGCCTTGTGTTCTTGTGCTATGATATACGCTTTT	991508
991509	**ATTTACATTCCACTCTGGGTAGATTTTAAT**	TTGGGTAAAAGAAGCGTTAGAAATAGCAATGAAAAA	991574
991575	**ATTTACATTCCACTCTGGGTAGATTTTAAT**		991604

Table S3A: Predicted genomic islands in the genome of *F. magna* 07T609

Shown are genes of genomic islands predicted by IslandViewer4

Island start	Island end	Length	Gene name	Locus	Gene start	Gene end	Strand	Product
65901	75199	9298	OXZ41700.1	B9N58_00255	65901	66770	1	hypothetical protein
65901	75199	9298	OXZ41701.1	B9N58_00260	66757	68745	1	exopolyphosphatase
65901	75199	9298	OXZ41702.1	B9N58_00265	68726	69172	1	50S ribosomal protein L9
65901	75199	9298	OXZ41703.1	B9N58_00270	69205	70542	1	replicative DNA helicase
65901	75199	9298	OXZ41704.1	B9N58_00275	70539	71102	1	N-acetyltransferase
65901	75199	9298	OXZ41705.1	B9N58_00280	71107	71865	1	DNA replication protein
65901	75199	9298	OXZ41706.1	B9N58_00285	71868	72845	1	DNA replication protein DnaC
65901	75199	9298	OXZ41707.1	B9N58_00290	72842	73486	1	NAD(P)H-hydrate epimerase
65901	75199	9298	OXZ41708.1	B9N58_00295	73987	74529	1	ECF transporter S component
65901	75199	9298	OXZ41709.1	B9N58_00300	74513	75199	1	radical SAM protein
185658	190851	5193	OXZ41632.1	B9N58_00840	185658	186299	-1	hemolysin III
185658	190851	5193	OXZ41633.1	B9N58_00845	186453	186956	1	acetyltransferase
185658	190851	5193	OXZ41634.1	B9N58_00850	186956	187690	1	hydrolase
185658	190851	5193	OXZ41524.1	B9N58_00855	190276	190851	1	cell filamentation protein Fic
313682	318134	4452	OXZ41489.1	B9N58_01405	313682	314023	1	plasmid mobilization relaxosome protein MobC
313682	318134	4452	OXZ41490.1	B9N58_01410	314128	314343	1	transcriptional regulator
313682	318134	4452	OXZ41491.1	B9N58_01415	314350	315393	1	DNA (cytosine-5-)-methyltransferase
313682	318134	4452	OXZ41492.1	B9N58_01420	315397	316479	1	restriction endonuclease
313682	318134	4452	OXZ41493.1	B9N58_01425	316524	318134	1	ABC transporter ATP-binding protein
733227	739719	6492	OXZ41083.1	B9N58_03590	733227	733925	1	DNA-binding response regulator
733227	739719	6492	OXZ41084.1	B9N58_03595	733966	734550	1	peptidylprolyl isomerase
733227	739719	6492	OXZ41085.1	B9N58_03600	734552	735184	1	thymidine kinase
733227	739719	6492	OXZ41086.1	B9N58_03605	735206	735676	-1	S-ribosylhomocysteine lyase
733227	739719	6492	OXZ41087.1	B9N58_03610	735685	736788	-1	rod shape-determining protein RodA
733227	739719	6492	OXZ41088.1	B9N58_03615	736863	737696	1	endonuclease IV
733227	739719	6492	OXZ41089.1	B9N58_03620	737677	738303	1	endonuclease III
733227	739719	6492	OXZ41090.1	B9N58_03625	738371	739291	1	aspartate carbamoyltransferase
733227	739719	6492	OXZ41091.1	B9N58_03630	739291	739719	1	aspartate carbamoyltransferase regulatory subunit
756103	762234	6131	OXZ41108.1	B9N58_03715	756103	757791	1	hypothetical protein
756103	762234	6131	OXZ41109.1	B9N58_03720	757802	758599	1	pyruvate, phosphate dikinase regulatory protein
756103	762234	6131	OXZ41110.1	B9N58_03725	758683	760116	1	serine/threonine protein kinase
756103	762234	6131	OXZ41111.1	B9N58_03730	760118	760792	1	protein phosphatase
756103	762234	6131	OXZ41112.1	B9N58_03735	760849	762234	1	MiaB
795264	799700	4436	OXZ41144.1	B9N58_03900	795264	795599	-1	histidine triad nucleotide-binding protein
795264	799700	4436	OXZ41145.1	B9N58_03905	795646	796938	-1	methylthiotransferase MtaB
795264	799700	4436	OXZ41146.1	B9N58_03910	796938	797663	-1	rRNA methyltransferase
795264	799700	4436	OXZ41147.1	B9N58_03915	797656	798582	-1	50S ribosomal protein L11 methyltransferase
795264	799700	4436	OXZ41148.1	B9N58_03920	798582	799700	-1	molecular chaperone DnaJ
954114	1006418	52304	OXZ40898.1	B9N58_04620	954114	955391	-1	ABC transporter permease
954114	1006418	52304	OXZ40899.1	B9N58_04625	955589	955876	-1	cupin
954114	1006418	52304	OXZ40900.1	B9N58_04630	955944	956516	-1	uracil-DNA glycosylase
954114	1006418	52304		B9N58_04635	956655	956857	-1	hypothetical protein
954114	1006418	52304		B9N58_04640	956885	957130	-1	hypothetical protein
954114	1006418	52304		B9N58_04645	957132	957936	-1	ABC transporter
954114	1006418	52304		B9N58_04650	957923	958642	-1	iron ABC transporter permease
954114	1006418	52304	OXZ40901.1	B9N58_04655	958702	959460	-1	SAM-dependent methyltransferase
954114	1006418	52304	OXZ40902.1	B9N58_04660	959555	960148	1	transcriptional regulator
954114	1006418	52304	OXZ40903.1	B9N58_04665	960299	960490	-1	hypothetical protein
954114	1006418	52304		B9N58_04670	960685	961231	1	transposase
954114	1006418	52304	OXZ40904.1	B9N58_04675	961267	962100	1	hypothetical protein
954114	1006418	52304	OXZ40905.1	B9N58_04680	962331	963533	-1	ABC transporter substrate-binding protein
954114	1006418	52304	OXZ40906.1	B9N58_04685	963533	963958	-1	FMN-binding domain-containing protein
954114	1006418	52304	OXZ40907.1	B9N58_04690	963955	964674	-1	GTPase
954114	1006418	52304	OXZ40908.1	B9N58_04695	964686	965834	-1	ABC transporter permease
954114	1006418	52304	OXZ40909.1	B9N58_04700	965824	967107	-1	ABC transporter permease
954114	1006418	52304	OXZ40910.1	B9N58_04705	967116	968378	-1	hypothetical protein
954114	1006418	52304	OXZ40911.1	B9N58_04710	968435	969100	-1	hypothetical protein
954114	1006418	52304	OXZ40912.1	B9N58_04715	969110	970447	-1	iron transporter
954114	1006418	52304	OXZ40913.1	B9N58_04720	970450	971127	-1	ABC transporter ATP-binding protein
954114	1006418	52304	OXZ40914.1	B9N58_04725	971127	971522	-1	hypothetical protein
954114	1006418	52304	OXZ40915.1	B9N58_04730	971537	971719	-1	ferrous iron transporter B
954114	1006418	52304	OXZ40916.1	B9N58_04735	971967	972197	-1	ferrous iron transport protein A
954114	1006418	52304	OXZ40917.1	B9N58_04740	972495	972602	-1	replication initiator domain protein A
954114	1006418	52304	OXZ40875.1	B9N58_04745	973855	974205	-1	hypothetical protein
954114	1006418	52304	OXZ40876.1	B9N58_04750	974230	974955	-1	ABC transporter permease
954114	1006418	52304	OXZ40877.1	B9N58_04755	974939	975823	-1	multidrug ABC transporter ATP-binding protein
954114	1006418	52304	OXZ40878.1	B9N58_04760	975801	976043	-1	hypothetical protein
954114	1006418	52304	OXZ40879.1	B9N58_04765	976036	976434	-1	hypothetical protein
954114	1006418	52304	OXZ40880.1	B9N58_04770	976565	977170	-1	DNA-binding response regulator
954114	1006418	52304	OXZ40881.1	B9N58_04775	977142	978689	-1	hypothetical protein
954114	1006418	52304	OXZ40882.1	B9N58_04780	978977	979633	-1	DNA-binding protein
954114	1006418	52304	OXZ40883.1	B9N58_04785	979677	987320	-1	DNA helicase
954114	1006418	52304	OXZ40884.1	B9N58_04790	987403	989616	-1	hypothetical protein
954114	1006418	52304	OXZ40885.1	B9N58_04795	989652	990614	-1	DNA (cytosine-5-)-methyltransferase

954114	1006418	52304	OXZ40886.1	B9N58_04800	990607	992328	-1	DNA topoisomerase III
954114	1006418	52304	OXZ40887.1	B9N58_04805	992392	993258	-1	bacteriocin
954114	1006418	52304	OXZ40888.1	B9N58_04810	993273	995696	-1	copper amine oxidase
954114	1006418	52304	OXZ40889.1	B9N58_04815	995683	995922	-1	hypothetical protein
954114	1006418	52304	OXZ40890.1	B9N58_04820	995937	998516	-1	hypothetical protein
954114	1006418	52304	OXZ40891.1	B9N58_04825	998524	1000947	-1	conjugal transfer protein TraE
954114	1006418	52304		B9N58_04830	1002140	1002496	-1	hypothetical protein
954114	1006418	52304	OXZ40837.1	B9N58_04835	1002498	1002788	-1	hypothetical protein
954114	1006418	52304	OXZ40838.1	B9N58_04840	1002798	1003661	-1	hypothetical protein
954114	1006418	52304	OXZ40839.1	B9N58_04845	1003672	1003887	-1	conjugal transfer protein
954114	1006418	52304	OXZ40840.1	B9N58_04850	1003889	1004200	-1	single-stranded DNA-binding protein
954114	1006418	52304		B9N58_04855	1004396	1005864	-1	hypothetical protein
954114	1006418	52304	OXZ40841.1	B9N58_04860	1005882	1006418	-1	ClbS/DfsB family four-helix bundle protein
956655	968378	11723		B9N58_04635	956655	956857	-1	hypothetical protein
956655	968378	11723		B9N58_04640	956885	957130	-1	hypothetical protein
956655	968378	11723		B9N58_04645	957132	957936	-1	ABC transporter
956655	968378	11723		B9N58_04650	957923	958642	-1	iron ABC transporter permease
956655	968378	11723	OXZ40901.1	B9N58_04655	958702	959460	-1	SAM-dependent methyltransferase
956655	968378	11723	OXZ40902.1	B9N58_04660	959555	960148	1	transcriptional regulator
956655	968378	11723	OXZ40903.1	B9N58_04665	960299	960490	-1	hypothetical protein
956655	968378	11723		B9N58_04670	960685	961231	1	transposase
956655	968378	11723	OXZ40904.1	B9N58_04675	961267	962100	1	hypothetical protein
956655	968378	11723	OXZ40905.1	B9N58_04680	962331	963533	-1	ABC transporter substrate-binding protein
956655	968378	11723	OXZ40906.1	B9N58_04685	963533	963958	-1	FMN-binding domain-containing protein
956655	968378	11723	OXZ40907.1	B9N58_04690	963955	964674	-1	GTPase
956655	968378	11723	OXZ40908.1	B9N58_04695	964686	965834	-1	ABC transporter permease
956655	968378	11723	OXZ40909.1	B9N58_04700	965824	967107	-1	ABC transporter permease
956655	968378	11723	OXZ40910.1	B9N58_04705	967116	968378	-1	hypothetical protein
970450	979633	9183	OXZ40913.1	B9N58_04720	970450	971127	-1	ABC transporter ATP-binding protein
970450	979633	9183	OXZ40914.1	B9N58_04725	971127	971522	-1	hypothetical protein
970450	979633	9183	OXZ40915.1	B9N58_04730	971537	971719	-1	ferrous iron transporter B
970450	979633	9183	OXZ40916.1	B9N58_04735	971967	972197	-1	ferrous iron transport protein A
970450	979633	9183	OXZ40917.1	B9N58_04740	972495	972602	-1	replication initiator domain protein A
970450	979633	9183	OXZ40875.1	B9N58_04745	973855	974205	-1	hypothetical protein
970450	979633	9183	OXZ40876.1	B9N58_04750	974230	974955	-1	ABC transporter permease
970450	979633	9183	OXZ40877.1	B9N58_04755	974939	975823	-1	multidrug ABC transporter ATP-binding protein
970450	979633	9183	OXZ40878.1	B9N58_04760	975801	976043	-1	hypothetical protein
970450	979633	9183	OXZ40879.1	B9N58_04765	976036	976434	-1	hypothetical protein
970450	979633	9183	OXZ40880.1	B9N58_04770	976565	977170	-1	DNA-binding response regulator
970450	979633	9183	OXZ40881.1	B9N58_04775	977142	978689	-1	hypothetical protein
970450	979633	9183	OXZ40882.1	B9N58_04780	978977	979633	-1	DNA-binding protein
1022942	1030690	7748	OXZ40859.1	B9N58_04950	1022942	1023106	-1	hypothetical protein
1022942	1030690	7748	OXZ40860.1	B9N58_04955	1023211	1023579	-1	hypothetical protein
1022942	1030690	7748	OXZ40874.1	B9N58_04960	1023590	1023787	-1	hypothetical protein
1022942	1030690	7748	OXZ40861.1	B9N58_04965	1023879	1025384	-1	cardiolipin synthase
1022942	1030690	7748	OXZ40862.1	B9N58_04970	1025449	1026423	-1	choloylglycine hydrolase
1022942	1030690	7748	OXZ40863.1	B9N58_04975	1026718	1027017	-1	hypothetical protein
1022942	1030690	7748	OXZ40864.1	B9N58_04980	1027079	1028539	-1	conjugal transfer protein TraA
1022942	1030690	7748	OXZ40865.1	B9N58_04985	1028618	1028806	-1	hypothetical protein
1022942	1030690	7748	OXZ40866.1	B9N58_04990	1028781	1029101	-1	hypothetical protein
1022942	1030690	7748	OXZ40867.1	B9N58_04995	1029212	1030690	-1	Lsa family ABC-F type ribosomal protection protein
1028781	1037345	8564	OXZ40865.1	B9N58_04985	1028618	1028806	-1	hypothetical protein
1028781	1037345	8564	OXZ40866.1	B9N58_04990	1028781	1029101	-1	hypothetical protein
1028781	1037345	8564	OXZ40867.1	B9N58_04995	1029212	1030690	-1	Lsa family ABC-F type ribosomal protection protein
1028781	1037345	8564	OXZ40868.1	B9N58_05000	1030738	1032408	-1	recombinase
1028781	1037345	8564	OXZ40869.1	B9N58_05005	1032451	1033029	-1	conjugal transfer protein TraG
1028781	1037345	8564	OXZ40870.1	B9N58_05010	1033022	1033756	-1	phage antirepressor Ant
1028781	1037345	8564	OXZ40871.1	B9N58_05015	1033753	1034238	-1	conjugal transfer protein
1028781	1037345	8564	OXZ40872.1	B9N58_05020	1034231	1035058	-1	nucleoside triphosphate hydrolase
1028781	1037345	8564	OXZ40873.1	B9N58_05025	1035055	1035828	-1	replication initiator protein A
1028781	1037345	8564	OXZ40720.1	B9N58_05030	1037160	1037345	-1	hypothetical protein
1033022	1037345	4323	OXZ40869.1	B9N58_05005	1032451	1033029	-1	conjugal transfer protein TraG
1033022	1037345	4323	OXZ40870.1	B9N58_05010	1033022	1033756	-1	phage antirepressor Ant
1033022	1037345	4323	OXZ40871.1	B9N58_05015	1033753	1034238	-1	conjugal transfer protein
1033022	1037345	4323	OXZ40872.1	B9N58_05020	1034231	1035058	-1	nucleoside triphosphate hydrolase
1033022	1037345	4323	OXZ40873.1	B9N58_05025	1035055	1035828	-1	replication initiator protein A
1033022	1037345	4323	OXZ40720.1	B9N58_05030	1037160	1037345	-1	hypothetical protein
1424435	1460901	36466	OXZ40485.1	B9N58_06810	1423846	1424445	-1	capsular biosynthesis protein
1424435	1460901	36466	OXZ40486.1	B9N58_06815	1424435	1425073	-1	capsular biosynthesis protein
1424435	1460901	36466	OXZ40487.1	B9N58_06820	1425075	1425854	-1	capsular biosynthesis protein
1424435	1460901	36466	OXZ40488.1	B9N58_06825	1425855	1426277	-1	enterotoxin
1424435	1460901	36466	OXZ40489.1	B9N58_06830	1426458	1426982	1	DNA topology modulation protein FlaR
1424435	1460901	36466	OXZ40490.1	B9N58_06835	1427098	1434705	-1	hypothetical protein
1424435	1460901	36466	OXZ40491.1	B9N58_06840	1435350	1440322	-1	hypothetical protein
1424435	1460901	36466	OXZ40350.1	B9N58_06845	1441323	1444408	-1	hypothetical protein
1424435	1460901	36466	OXZ40351.1	B9N58_06850	1444520	1449238	-1	hypothetical protein
1424435	1460901	36466	OXZ40352.1	B9N58_06855	1450091	1450546	-1	IS200/IS605 family transposase
1424435	1460901	36466	OXZ40353.1	B9N58_06860	1450748	1452637	-1	cell wall protein

1424435	1460901	36466	OXZ40354.1	B9N58_06865	1453409	1453840	-1	GNAT family N-acetyltransferase
1424435	1460901	36466	OXZ40355.1	B9N58_06870	1453866	1454564	-1	hypothetical protein
1424435	1460901	36466	OXZ40356.1	B9N58_06875	1454564	1455283	-1	peptidase S51
1424435	1460901	36466	OXZ40357.1	B9N58_06880	1456188	1459541	-1	ABC transporter permease
1424435	1460901	36466	OXZ40358.1	B9N58_06885	1459543	1460244	-1	macrolide ABC transporter ATP-binding protein
1424435	1460901	36466	OXZ40359.1	B9N58_06890	1460356	1460901	1	TetR family transcriptional regulator
1435350	1440322	4972	OXZ40491.1	B9N58_06840	1435350	1440322	-1	hypothetical protein
1494017	1575399	81382	OXZ40386.1	B9N58_07025	1494017	1494325	-1	exosortase
1494017	1575399	81382	OXZ40387.1	B9N58_07030	1494330	1495001	-1	ATPase
1494017	1575399	81382	OXZ40388.1	B9N58_07035	1495202	1496740	-1	hypothetical protein
1494017	1575399	81382	OXZ40389.1	B9N58_07040	1496916	1498424	-1	AAA family ATPase
1494017	1575399	81382	OXZ40390.1	B9N58_07045	1499168	1500058	-1	methionine sulfoxide reductase
1494017	1575399	81382	OXZ40391.1	B9N58_07050	1500214	1500705	-1	protein-S-isoprenylcysteine methyltransferase
1494017	1575399	81382	OXZ40392.1	B9N58_07055	1500719	1501657	-1	esterase
1494017	1575399	81382	OXZ40393.1	B9N58_07060	1501726	1502352	-1	hypothetical protein
1494017	1575399	81382	OXZ40394.1	B9N58_07065	1502380	1502988	-1	SAM-dependent methyltransferase
1494017	1575399	81382	OXZ40395.1	B9N58_07070	1503208	1504962	-1	hypothetical protein
1494017	1575399	81382	OXZ40396.1	B9N58_07075	1505329	1505835	-1	hypothetical protein
1494017	1575399	81382	OXZ40397.1	B9N58_07080	1505846	1506592	-1	alpha/beta hydrolase
1494017	1575399	81382	OXZ40398.1	B9N58_07085	1506627	1507460	-1	SAM-dependent methyltransferase
1494017	1575399	81382	OXZ40399.1	B9N58_07090	1507482	1508108	-1	hypothetical protein
1494017	1575399	81382	OXZ40400.1	B9N58_07095	1508325	1509689	-1	ABC transporter ATP-binding protein
1494017	1575399	81382	OXZ40401.1	B9N58_07100	1509683	1510372	-1	cobalt ABC transporter permease
1494017	1575399	81382	OXZ40402.1	B9N58_07105	1510372	1510956	-1	hypothetical protein
1494017	1575399	81382	OXZ40480.1	B9N58_07110	1511016	1512359	-1	MATE family efflux transporter
1494017	1575399	81382	OXZ40403.1	B9N58_07115	1512371	1514092	-1	ABC transporter ATP-binding protein
1494017	1575399	81382	OXZ40404.1	B9N58_07120	1514076	1515830	-1	ABC transporter ATP-binding protein
1494017	1575399	81382	OXZ40405.1	B9N58_07125	1515991	1516983	1	AraC family transcriptional regulator
1494017	1575399	81382		B9N58_07130	1517161	1517348	1	cell filamentation protein
1494017	1575399	81382	OXZ40406.1	B9N58_07135	1517574	1518278	-1	hypothetical protein
1494017	1575399	81382	OXZ40407.1	B9N58_07140	1518287	1519039	-1	hypothetical protein
1494017	1575399	81382	OXZ40408.1	B9N58_07145	1519041	1519934	-1	ABC transporter ATP-binding protein
1494017	1575399	81382	OXZ40409.1	B9N58_07150	1520063	1520758	1	DNA-binding response regulator
1494017	1575399	81382	OXZ40410.1	B9N58_07155	1520760	1522055	1	two-component sensor histidine kinase
1494017	1575399	81382		B9N58_07160	1522098	1522384	-1	hypothetical protein
1494017	1575399	81382		B9N58_07165	1522760	1523472	-1	hypothetical protein
1494017	1575399	81382	OXZ40411.1	B9N58_07170	1523525	1525261	-1	multidrug ABC transporter ATP-binding protein
1494017	1575399	81382	OXZ40412.1	B9N58_07175	1525254	1526975	-1	ABC transporter ATP-binding protein
1494017	1575399	81382	OXZ40413.1	B9N58_07180	1526987	1528438	-1	ABC transporter
1494017	1575399	81382	OXZ40414.1	B9N58_07185	1528440	1529111	-1	cobalt transporter
1494017	1575399	81382	OXZ40415.1	B9N58_07190	1529108	1529698	-1	hypothetical protein
1494017	1575399	81382	OXZ40416.1	B9N58_07195	1529765	1530742	-1	AraC family transcriptional regulator
1494017	1575399	81382	OXZ40417.1	B9N58_07200	1530752	1531336	-1	TetR family transcriptional regulator
1494017	1575399	81382		B9N58_07205	1531537	1531919	-1	AraC family transcriptional regulator
1494017	1575399	81382	OXZ40418.1	B9N58_07210	1532893	1533549	-1	hypothetical protein
1494017	1575399	81382	OXZ40419.1	B9N58_07215	1533798	1534394	-1	hypothetical protein
1494017	1575399	81382	OXZ40420.1	B9N58_07220	1534396	1534854	-1	hypothetical protein
1494017	1575399	81382	OXZ40421.1	B9N58_07225	1535164	1536900	-1	multidrug ABC transporter ATP-binding protein
1494017	1575399	81382	OXZ40422.1	B9N58_07230	1536893	1538611	-1	ABC transporter ATP-binding protein
1494017	1575399	81382	OXZ40423.1	B9N58_07235	1538616	1539212	-1	TetR family transcriptional regulator
1494017	1575399	81382	OXZ40424.1	B9N58_07240	1539506	1539811	1	plasmid mobilization relaxosome protein MobC
1494017	1575399	81382		B9N58_07245	1539864	1540352	1	relaxase
1494017	1575399	81382	OXZ40425.1	B9N58_07250	1540435	1542969	-1	DEAD/DEAH box helicase
1494017	1575399	81382	OXZ40426.1	B9N58_07255	1542972	1543805	-1	hypothetical protein
1494017	1575399	81382	OXZ40427.1	B9N58_07260	1543842	1544003	-1	transcriptional regulator
1494017	1575399	81382		B9N58_07265	1544226	1544567	-1	hypothetical protein
1494017	1575399	81382	OXZ40428.1	B9N58_07270	1544618	1544866	-1	hypothetical protein
1494017	1575399	81382	OXZ40429.1	B9N58_07275	1544863	1545450	-1	transcriptional regulator
1494017	1575399	81382	OXZ40430.1	B9N58_07280	1545949	1547625	-1	recombinase family protein
1494017	1575399	81382	OXZ40431.1	B9N58_07285	1548719	1549651	-1	AraC family transcriptional regulator
1494017	1575399	81382	OXZ40432.1	B9N58_07290	1549648	1551039	-1	ABC transporter ATP-binding protein
1494017	1575399	81382	OXZ40433.1	B9N58_07295	1551033	1551728	-1	ABC transporter permease
1494017	1575399	81382		B9N58_07300	1551728	1552309	-1	hypothetical protein
1494017	1575399	81382	OXZ40434.1	B9N58_07305	1552320	1554041	-1	ABC transporter ATP-binding protein
1494017	1575399	81382	OXZ40435.1	B9N58_07310	1554038	1555786	-1	ABC transporter ATP-binding protein
1494017	1575399	81382	OXZ40436.1	B9N58_07315	1556431	1556901	-1	replication protein
1494017	1575399	81382	OXZ40437.1	B9N58_07320	1556986	1557597	-1	SAM-dependent methyltransferase
1494017	1575399	81382	OXZ40438.1	B9N58_07325	1557801	1558328	-1	conjugal transfer protein
1494017	1575399	81382	OXZ40439.1	B9N58_07330	1558760	1560394	-1	MobA/MobL protein
1494017	1575399	81382	OXZ40440.1	B9N58_07335	1560740	1561030	-1	hypothetical protein
1494017	1575399	81382	OXZ40441.1	B9N58_07340	1561269	1561748	1	AraC family transcriptional regulator
1494017	1575399	81382	OXZ40442.1	B9N58_07345	1562009	1562503	-1	hypothetical protein
1494017	1575399	81382	OXZ40443.1	B9N58_07350	1562933	1563151	-1	transcriptional regulator
1494017	1575399	81382	OXZ40444.1	B9N58_07355	1563269	1563427	-1	hypothetical protein
1494017	1575399	81382	OXZ40445.1	B9N58_07360	1563429	1563740	-1	single-stranded DNA-binding protein
1494017	1575399	81382	OXZ40446.1	B9N58_07365	1563943	1564251	-1	hypothetical protein
1494017	1575399	81382	OXZ40447.1	B9N58_07370	1564280	1564468	-1	hypothetical protein
1494017	1575399	81382	OXZ40448.1	B9N58_07375	1564741	1566234	-1	23S rRNA (uracil(1939)-C(5))-methyltransferase RlmD

1494017	1575399	81382	OXZ40449.1	B9N58_07380	1566327	1568123	-1	ABC transporter
1494017	1575399	81382	OXZ40450.1	B9N58_07385	1568116	1569876	-1	multidrug ABC transporter ATP-binding protein
1494017	1575399	81382	OXZ40451.1	B9N58_07390	1569851	1570327	-1	MarR family transcriptional regulator
1494017	1575399	81382	OXZ40452.1	B9N58_07395	1570494	1571795	-1	phosphomethylpyrimidine synthase
1494017	1575399	81382	OXZ40453.1	B9N58_07400	1571976	1572890	-1	cyclic pyranopterin monophosphate synthase MoaC
1494017	1575399	81382	OXZ40454.1	B9N58_07405	1572892	1573839	-1	GTP 3',8-cyclase MoaA
1494017	1575399	81382	OXZ40455.1	B9N58_07410	1573927	1575102	-1	hypothetical protein
1494017	1575399	81382	OXZ40456.1	B9N58_07415	1575118	1575399	-1	hypothetical protein
1520063	1530742	10679	OXZ40409.1	B9N58_07150	1520063	1520758	1	DNA-binding response regulator
1520063	1530742	10679	OXZ40410.1	B9N58_07155	1520760	1522055	1	two-component sensor histidine kinase
1520063	1530742	10679		B9N58_07160	1522098	1522384	-1	hypothetical protein
1520063	1530742	10679		B9N58_07165	1522760	1523472	-1	hypothetical protein
1520063	1530742	10679	OXZ40411.1	B9N58_07170	1523525	1525261	-1	multidrug ABC transporter ATP-binding protein
1520063	1530742	10679	OXZ40412.1	B9N58_07175	1525254	1526975	-1	ABC transporter ATP-binding protein
1520063	1530742	10679	OXZ40413.1	B9N58_07180	1526987	1528438	-1	ABC transporter
1520063	1530742	10679	OXZ40414.1	B9N58_07185	1528440	1529111	-1	cobalt transporter
1520063	1530742	10679	OXZ40415.1	B9N58_07190	1529108	1529698	-1	hypothetical protein
1520063	1530742	10679	OXZ40416.1	B9N58_07195	1529765	1530742	-1	AraC family transcriptional regulator
1556431	1561748	5317	OXZ40436.1	B9N58_07315	1556431	1556901	-1	replication protein
1556431	1561748	5317	OXZ40437.1	B9N58_07320	1556986	1557597	-1	SAM-dependent methyltransferase
1556431	1561748	5317	OXZ40438.1	B9N58_07325	1557801	1558328	-1	conjugal transfer protein
1556431	1561748	5317	OXZ40439.1	B9N58_07330	1558760	1560394	-1	MobA/MobL protein
1556431	1561748	5317	OXZ40440.1	B9N58_07335	1560740	1561030	-1	hypothetical protein
1556431	1561748	5317	OXZ40441.1	B9N58_07340	1561269	1561748	1	AraC family transcriptional regulator
1717729	1724002	6273	OXZ39742.1	B9N58_08075	1716944	1717747	-1	ABC transporter ATP-binding protein
1717729	1724002	6273	OXZ39743.1	B9N58_08080	1717729	1717866	-1	nitrate ABC transporter substrate-binding protein
1717729	1724002	6273	OXZ39744.1	B9N58_08085	1717906	1718529	-1	ABC transporter permease
1717729	1724002	6273	OXZ39745.1	B9N58_08090	1718490	1718999	-1	hypothetical protein
1717729	1724002	6273	OXZ39746.1	B9N58_08095	1719051	1719266	-1	hypothetical protein
1717729	1724002	6273	OXZ39747.1	B9N58_08100	1719259	1719450	-1	hypothetical protein
1717729	1724002	6273		B9N58_08105	1719930	1720375	1	hypothetical protein
1717729	1724002	6273		B9N58_08110	1720544	1720825	-1	type I restriction endonuclease
1717729	1724002	6273	OXZ39748.1	B9N58_08115	1720890	1721873	-1	hypothetical protein
1717729	1724002	6273	OXZ39749.1	B9N58_08120	1721866	1722477	-1	hypothetical protein
1717729	1724002	6273	OXZ39750.1	B9N58_08125	1722803	1724002	-1	sugar ABC transporter ATP-binding protein

Table S3B: Predicted genomic islands in the genome of *F. magna* 08T492

Shown are genes of genomic islands predicted by IslandViewer4

Island start	Island end	Length	Gene name	Locus	Gene start	Gene end	Strand	Product
4466	13082	8616	OXZ39870.1	B9N56_00035	4466	5335	1	hypothetical protein
4466	13082	8616	OXZ39871.1	B9N56_00040	5322	7310	1	exopolyphosphatase
4466	13082	8616	OXZ39872.1	B9N56_00045	7291	7737	1	50S ribosomal protein L9
4466	13082	8616	OXZ39873.1	B9N56_00050	7771	9108	1	replicative DNA helicase
4466	13082	8616	OXZ39874.1	B9N56_00055	9105	9668	1	GNAT family N-acetyltransferase
4466	13082	8616	OXZ39875.1	B9N56_00060	9673	10431	1	DNA replication protein
4466	13082	8616	OXZ39876.1	B9N56_00065	10434	11411	1	DNA replication protein DnaC
4466	13082	8616	OXZ39877.1	B9N56_00070	11408	12052	1	NAD(P)H-hydrate epimerase
4466	13082	8616	OXZ39878.1	B9N56_00075	12540	13082	1	ECF transporter S component
4466	13082	8616	OXZ39879.1	B9N56_00080	13066	13752	1	radical SAM protein
89839	96411	6572	OXZ39971.1	B9N56_00550	89839	91074	1	peptidoglycan-binding protein
89839	96411	6572	OXZ39695.1	B9N56_00615	95728	96003	-1	hypothetical protein
89839	96411	6572		B9N56_00620	96091	96411	-1	hypothetical protein
117178	124321	7143	OXZ39693.1	B9N56_00665	117178	117819	-1	hemolysin III
117178	124321	7143	OXZ39697.1	B9N56_00670	117972	118475	1	acetyltransferase
117178	124321	7143	OXZ39694.1	B9N56_00675	118475	119209	1	hydrolase
117178	124321	7143	OXZ39623.1	B9N56_00680	121747	122322	1	cell filamentation protein Fic
117178	124321	7143	OXZ39624.1	B9N56_00685	123705	124082	-1	death-on-curing protein
117178	124321	7143	OXZ39625.1	B9N56_00690	124079	124321	-1	prevent-host-death protein
675820	682312	6492	OXZ38460.1	B9N56_03450	675820	676518	1	DNA-binding response regulator
675820	682312	6492	OXZ38461.1	B9N56_03455	676559	677143	1	peptidylprolyl isomerase
675820	682312	6492	OXZ38462.1	B9N56_03460	677145	677777	1	thymidine kinase
675820	682312	6492	OXZ38463.1	B9N56_03465	677799	678269	-1	S-ribosylhomocysteine lyase
675820	682312	6492	OXZ38464.1	B9N56_03470	678278	679381	-1	rod shape-determining protein RodA
675820	682312	6492	OXZ38465.1	B9N56_03475	679456	680289	1	endonuclease
675820	682312	6492	OXZ38466.1	B9N56_03480	680270	680896	1	endonuclease III
675820	682312	6492	OXZ38467.1	B9N56_03485	680964	681884	1	aspartate carbamoyltransferase
675820	682312	6492	OXZ38468.1	B9N56_03490	681884	682312	1	subunit
698698	704829	6131	OXZ38485.1	B9N56_03575	698698	700386	1	hypothetical protein
698698	704829	6131	OXZ38486.1	B9N56_03580	700397	701194	1	pyruvate, phosphate dikinase regulatory protein
698698	704829	6131	OXZ38487.1	B9N56_03585	701278	702711	1	serine/threonine protein kinase
698698	704829	6131	OXZ38488.1	B9N56_03590	702713	703387	1	protein phosphatase
698698	704829	6131	OXZ38489.1	B9N56_03595	703444	704829	1	methylthiotransferase MiaB
737859	742292	4433	OXZ38521.1	B9N56_03760	737859	738194	-1	histidine triad nucleotide-binding protein
737859	742292	4433	OXZ38522.1	B9N56_03765	738241	739533	-1	C(2))- methylthiotransferase MtaB
737859	742292	4433	OXZ38523.1	B9N56_03770	739533	740258	-1	rRNA methyltransferase
737859	742292	4433	OXZ38524.1	B9N56_03775	740251	741174	-1	50S ribosomal protein L11 methyltransferase
737859	742292	4433	OXZ38525.1	B9N56_03780	741174	742292	-1	molecular chaperone DnaJ
892629	969377	76748	OXZ38269.1	B9N56_04475	892629	893906	-1	ABC transporter permease
892629	969377	76748	OXZ38270.1	B9N56_04480	894104	894391	-1	cupin
892629	969377	76748	OXZ38271.1	B9N56_04485	894459	895031	-1	uracil-DNA glycosylase
892629	969377	76748		B9N56_04490	895170	895373	-1	hypothetical protein
892629	969377	76748		B9N56_04495	895401	895646	-1	hypothetical protein
892629	969377	76748	OXZ38277.1	B9N56_04500	895648	896451	-1	ABC transporter
892629	969377	76748		B9N56_04505	896438	897157	-1	iron ABC transporter permease
892629	969377	76748	OXZ38272.1	B9N56_04510	897217	897975	-1	SAM-dependent methyltransferase
892629	969377	76748	OXZ38273.1	B9N56_04515	898070	898663	1	transcriptional regulator
892629	969377	76748	OXZ38274.1	B9N56_04520	899200	899384	1	transposase
892629	969377	76748	OXZ38150.1	B9N56_04525	900561	900788	1	transposase
892629	969377	76748	OXZ38148.1	B9N56_04530	900860	901066	1	hypothetical protein
892629	969377	76748	OXZ38149.1	B9N56_04535	901178	901694	1	IS3 family transposase
892629	969377	76748	OXZ38116.1	B9N56_04540	902695	902868	1	transposase
892629	969377	76748	OXZ38117.1	B9N56_04545	903099	904301	-1	ABC transporter substrate-binding protein
892629	969377	76748	OXZ38118.1	B9N56_04550	904301	904726	-1	FMN-binding domain-containing protein
892629	969377	76748	OXZ38119.1	B9N56_04555	904723	905442	-1	GTPase
892629	969377	76748	OXZ38120.1	B9N56_04560	905454	906602	-1	ABC transporter permease
892629	969377	76748	OXZ38121.1	B9N56_04565	906592	907875	-1	ABC transporter permease
892629	969377	76748	OXZ38122.1	B9N56_04570	907884	909146	-1	hypothetical protein
892629	969377	76748	OXZ38123.1	B9N56_04575	909203	909868	-1	hypothetical protein
892629	969377	76748	OXZ38124.1	B9N56_04580	909878	911215	-1	iron transporter
892629	969377	76748	OXZ38125.1	B9N56_04585	911218	911895	-1	ABC transporter ATP-binding protein
892629	969377	76748	OXZ38126.1	B9N56_04590	911895	912290	-1	hypothetical protein
892629	969377	76748		B9N56_04595	912333	912488	-1	hypothetical protein
892629	969377	76748	OXZ38127.1	B9N56_04600	912490	912732	-1	FeoA family protein
892629	969377	76748	OXZ38128.1	B9N56_04605	912736	912966	-1	ferrous iron transport protein A
892629	969377	76748	OXZ38129.1	B9N56_04610	913264	913371	-1	replication initiator domain protein A
892629	969377	76748	OXZ38130.1	B9N56_04615	913454	913726	-1	hypothetical protein
892629	969377	76748	OXZ38131.1	B9N56_04620	913799	914245	-1	methionine--tRNA ligase subunit beta
892629	969377	76748	OXZ38132.1	B9N56_04625	914402	915637	-1	site-specific integrase
892629	969377	76748	OXZ38133.1	B9N56_04630	915715	915924	-1	excisionase
892629	969377	76748		B9N56_04635	916360	916769	-1	RNA polymerase subunit sigma
892629	969377	76748	OXZ38134.1	B9N56_04640	916727	916840	-1	RNA polymerase subunit sigma-70

892629	969377	76748	OXZ38135.1	B9N56_04645	917089	918438	-1	MATE family efflux transporter
892629	969377	76748	OXZ38136.1	B9N56_04650	918475	920208	-1	multidrug ABC transporter ATP-binding protein
892629	969377	76748	OXZ38137.1	B9N56_04655	920205	921947	-1	ABC transporter ATP-binding protein
892629	969377	76748	OXZ38138.1	B9N56_04660	921983	923374	-1	ABC transporter ATP-binding protein
892629	969377	76748	OXZ38139.1	B9N56_04665	923367	924068	-1	cobalt ABC transporter permease
892629	969377	76748	OXZ38140.1	B9N56_04670	924093	924674	-1	hypothetical protein
892629	969377	76748	OXZ38141.1	B9N56_04675	924692	925303	-1	TetR family transcriptional regulator
892629	969377	76748	OXZ38142.1	B9N56_04680	925615	925971	1	plasmid mobilization relaxosome protein MobC
892629	969377	76748	OXZ38143.1	B9N56_04685	925973	927304	1	endonuclease
892629	969377	76748		B9N56_04690	927390	928475	1	IS256 family transposase
892629	969377	76748	OXZ38144.1	B9N56_04695	928580	930070	-1	hypothetical protein
892629	969377	76748	OXZ38145.1	B9N56_04700	930073	930783	-1	hypothetical protein
892629	969377	76748	OXZ38146.1	B9N56_04705	930784	931362	-1	hypothetical protein
892629	969377	76748	OXZ38147.1	B9N56_04710	931377	933107	-1	hypothetical protein
892629	969377	76748		B9N56_04715	934372	936342	-1	DNA helicase
892629	969377	76748	OXZ37671.1	B9N56_04720	936329	937294	-1	DNA (cytosine-5-)-methyltransferase
892629	969377	76748	OXZ37672.1	B9N56_04725	937287	939008	-1	DNA topoisomerase III
892629	969377	76748	OXZ37673.1	B9N56_04730	939097	940080	-1	hypothetical protein
892629	969377	76748	OXZ37674.1	B9N56_04735	940073	940684	-1	hypothetical protein
892629	969377	76748	OXZ37675.1	B9N56_04740	940758	941630	-1	bacteriocin
892629	969377	76748	OXZ37676.1	B9N56_04745	941645	943228	-1	copper amine oxidase
892629	969377	76748	OXZ37677.1	B9N56_04750	943215	943454	-1	hypothetical protein
892629	969377	76748	OXZ37678.1	B9N56_04755	943469	946048	-1	hypothetical protein
892629	969377	76748	OXZ37679.1	B9N56_04760	946056	948479	-1	conjugal transfer protein TraE
892629	969377	76748	OXZ37680.1	B9N56_04765	948370	948780	-1	PrgI family protein
892629	969377	76748	OXZ37681.1	B9N56_04770	948782	949072	-1	hypothetical protein
892629	969377	76748	OXZ37682.1	B9N56_04775	949082	949945	-1	hypothetical protein
892629	969377	76748		B9N56_04780	949956	950039	-1	hypothetical protein
892629	969377	76748	OXZ37683.1	B9N56_04785	950043	951689	-1	recombinase RecB
892629	969377	76748	OXZ37684.1	B9N56_04790	951693	951917	-1	hypothetical protein
892629	969377	76748	OXZ37685.1	B9N56_04795	952071	952772	-1	replisome organizer
892629	969377	76748	OXZ37686.1	B9N56_04800	952860	953855	-1	DNA-binding protein
892629	969377	76748	OXZ37687.1	B9N56_04805	953989	954186	-1	hypothetical protein
892629	969377	76748	OXZ37640.1	B9N56_04810	955228	955558	-1	nickase
892629	969377	76748	OXZ37641.1	B9N56_04815	955872	956186	-1	hypothetical protein
892629	969377	76748		B9N56_04820	956305	956403	-1	hypothetical protein
892629	969377	76748	OXZ37642.1	B9N56_04825	956449	957852	-1	ABC transporter
892629	969377	76748	OXZ37643.1	B9N56_04830	957857	958549	-1	hypothetical protein
892629	969377	76748	OXZ37644.1	B9N56_04835	958530	959117	-1	hypothetical protein
892629	969377	76748	OXZ37645.1	B9N56_04840	959141	960874	-1	ABC transporter ATP-binding protein
892629	969377	76748	OXZ37646.1	B9N56_04845	960862	962601	-1	ABC transporter ATP-binding protein
892629	969377	76748	OXZ37647.1	B9N56_04850	962906	963277	-1	transcriptional regulator
892629	969377	76748		B9N56_04855	963398	963598	-1	hypothetical protein
892629	969377	76748	OXZ37648.1	B9N56_04860	963602	963820	-1	transcriptional regulator
892629	969377	76748	OXZ37649.1	B9N56_04865	964132	965991	-1	group II intron reverse transcriptase/maturase
892629	969377	76748	OXZ37650.1	B9N56_04870	966714	966845	-1	hypothetical protein
892629	969377	76748	OXZ37651.1	B9N56_04875	966847	967158	-1	single-stranded DNA-binding protein
892629	969377	76748	OXZ37652.1	B9N56_04880	967354	968823	-1	hypothetical protein
892629	969377	76748	OXZ37653.1	B9N56_04885	968841	969377	-1	ClbS/DfsB family four-helix bundle protein
895170	899384	4214		B9N56_04490	895170	895373	-1	hypothetical protein
895170	899384	4214		B9N56_04495	895401	895646	-1	hypothetical protein
895170	899384	4214	OXZ38277.1	B9N56_04500	895648	896451	-1	ABC transporter
895170	899384	4214		B9N56_04505	896438	897157	-1	iron ABC transporter permease
895170	899384	4214	OXZ38272.1	B9N56_04510	897217	897975	-1	SAM-dependent methyltransferase
895170	899384	4214	OXZ38273.1	B9N56_04515	898070	898663	1	transcriptional regulator
895170	899384	4214	OXZ38274.1	B9N56_04520	899200	899384	1	transposase
902695	909146	6451	OXZ38116.1	B9N56_04540	902695	902868	1	transposase
902695	909146	6451	OXZ38117.1	B9N56_04545	903099	904301	-1	ABC transporter substrate-binding protein
902695	909146	6451	OXZ38118.1	B9N56_04550	904301	904726	-1	FMN-binding domain-containing protein
902695	909146	6451	OXZ38119.1	B9N56_04555	904723	905442	-1	GTPase
902695	909146	6451	OXZ38120.1	B9N56_04560	905454	906602	-1	ABC transporter permease
902695	909146	6451	OXZ38121.1	B9N56_04565	906592	907875	-1	ABC transporter permease
902695	909146	6451	OXZ38122.1	B9N56_04570	907884	909146	-1	hypothetical protein
921983	941630	19647	OXZ38138.1	B9N56_04660	921983	923374	-1	ABC transporter ATP-binding protein
921983	941630	19647	OXZ38139.1	B9N56_04665	923367	924068	-1	cobalt ABC transporter permease
921983	941630	19647	OXZ38140.1	B9N56_04670	924093	924674	-1	hypothetical protein
921983	941630	19647	OXZ38141.1	B9N56_04675	924692	925303	-1	TetR family transcriptional regulator
921983	941630	19647	OXZ38142.1	B9N56_04680	925615	925971	1	plasmid mobilization relaxosome protein MobC
921983	941630	19647	OXZ38143.1	B9N56_04685	925973	927304	1	endonuclease
921983	941630	19647		B9N56_04690	927390	928475	1	IS256 family transposase
921983	941630	19647	OXZ38144.1	B9N56_04695	928580	930070	-1	hypothetical protein
921983	941630	19647	OXZ38145.1	B9N56_04700	930073	930783	-1	hypothetical protein
921983	941630	19647	OXZ38146.1	B9N56_04705	930784	931362	-1	hypothetical protein
921983	941630	19647	OXZ38147.1	B9N56_04710	931377	933107	-1	hypothetical protein
921983	941630	19647		B9N56_04715	934372	936342	-1	DNA helicase
921983	941630	19647	OXZ37671.1	B9N56_04720	936329	937294	-1	DNA (cytosine-5-)-methyltransferase

921983	941630	19647	OXZ37672.1	B9N56_04725	937287	939008	-1	DNA topoisomerase III
921983	941630	19647	OXZ37673.1	B9N56_04730	939097	940080	-1	hypothetical protein
921983	941630	19647	OXZ37674.1	B9N56_04735	940073	940684	-1	hypothetical protein
921983	941630	19647	OXZ37675.1	B9N56_04740	940758	941630	-1	bacteriocin
953989	963820	9831	OXZ37687.1	B9N56_04805	953989	954186	-1	hypothetical protein
953989	963820	9831	OXZ37640.1	B9N56_04810	955228	955558	-1	nickase
953989	963820	9831	OXZ37641.1	B9N56_04815	955872	956186	-1	hypothetical protein
953989	963820	9831		B9N56_04820	956305	956403	-1	hypothetical protein
953989	963820	9831	OXZ37642.1	B9N56_04825	956449	957852	-1	ABC transporter
953989	963820	9831	OXZ37643.1	B9N56_04830	957857	958549	-1	hypothetical protein
953989	963820	9831	OXZ37644.1	B9N56_04835	958530	959117	-1	hypothetical protein
953989	963820	9831	OXZ37645.1	B9N56_04840	959141	960874	-1	ABC transporter ATP-binding protein
953989	963820	9831	OXZ37646.1	B9N56_04845	960862	962601	-1	ABC transporter ATP-binding protein
953989	963820	9831	OXZ37647.1	B9N56_04850	962906	963277	-1	transcriptional regulator
953989	963820	9831		B9N56_04855	963398	963598	-1	hypothetical protein
953989	963820	9831	OXZ37648.1	B9N56_04860	963602	963820	-1	transcriptional regulator
974719	979692	4973	OXZ37660.1	B9N56_04920	974719	975048	-1	hypothetical protein
974719	979692	4973	OXZ37661.1	B9N56_04925	975133	975546	-1	RNA polymerase subunit sigma-70
974719	979692	4973	OXZ37662.1	B9N56_04930	975834	977216	-1	MATE family efflux transporter
974719	979692	4973	OXZ37663.1	B9N56_04935	977218	979692	-1	phosphoenolpyruvate synthase
1190641	1202413	11772	OXZ37235.1	B9N56_05920	1190641	1190955	1	conjugal transfer protein
1190641	1202413	11772	OXZ37236.1	B9N56_05925	1190971	1191357	1	conjugal transfer protein
1190641	1202413	11772		B9N56_05930	1191386	1192771	1	ATP-binding protein
1190641	1202413	11772		B9N56_05935	1192774	1192927	1	conjugal transfer protein
1190641	1202413	11772	OXZ37237.1	B9N56_05940	1192950	1194155	1	Cro/Cl family transcriptional regulator
1190641	1202413	11772	OXZ37238.1	B9N56_05945	1194198	1194419	1	hypothetical protein
1190641	1202413	11772	OXZ37239.1	B9N56_05950	1194536	1195033	1	antirestriction protein ArdA
1190641	1202413	11772	OXZ37240.1	B9N56_05955	1195008	1195514	1	conjugal transfer protein
1190641	1202413	11772	OXZ37241.1	B9N56_05960	1195498	1197945	1	ATP/GTP-binding protein
1190641	1202413	11772	OXZ37242.1	B9N56_05965	1197948	1200125	1	hypothetical protein
1190641	1202413	11772	OXZ37243.1	B9N56_05970	1200122	1201123	1	peptidase P60
1190641	1202413	11772	OXZ37244.1	B9N56_05975	1201120	1202052	1	conjugal transfer protein
1190641	1202413	11772	OXZ37245.1	B9N56_05980	1202297	1202413	1	peptide
1317420	1321668	4248	OXZ37344.1	B9N56_06535	1317420	1318223	-1	DNA-binding protein
1317420	1321668	4248	OXZ37345.1	B9N56_06540	1318413	1319933	-1	conjugal transfer protein
1317420	1321668	4248	OXZ37346.1	B9N56_06545	1319926	1321668	-1	recombinase
1317420	1321668	4248	OXZ37347.1	B9N56_06550	1321661	1323379	-1	recombinase
1318413	1431755	113342	OXZ37345.1	B9N56_06540	1318413	1319933	-1	conjugal transfer protein
1318413	1431755	113342	OXZ37346.1	B9N56_06545	1319926	1321668	-1	recombinase
1318413	1431755	113342	OXZ37347.1	B9N56_06550	1321661	1323379	-1	recombinase
1318413	1431755	113342	OXZ37348.1	B9N56_06555	1323478	1323645	-1	aminoacyl-tRNA hydrolase
1318413	1431755	113342	OXZ37349.1	B9N56_06560	1324114	1324521	-1	RNA polymerase subunit sigma
1318413	1431755	113342	OXZ37350.1	B9N56_06565	1325321	1326664	-1	MATE family efflux transporter
1318413	1431755	113342	OXZ37351.1	B9N56_06570	1326676	1328424	-1	ABC transporter ATP-binding protein
1318413	1431755	113342	OXZ37352.1	B9N56_06575	1328378	1330132	-1	ABC transporter ATP-binding protein
1318413	1431755	113342	OXZ37353.1	B9N56_06580	1330281	1331240	1	AraC family transcriptional regulator
1318413	1431755	113342	OXZ37354.1	B9N56_06585	1331302	1332690	-1	ABC transporter ATP-binding protein
1318413	1431755	113342	OXZ37355.1	B9N56_06590	1332701	1333357	-1	cobalt transporter
1318413	1431755	113342	OXZ37356.1	B9N56_06595	1333359	1333940	-1	hypothetical protein
1318413	1431755	113342	OXZ37357.1	B9N56_06600	1333951	1337238	-1	hypothetical protein
1318413	1431755	113342	OXZ37358.1	B9N56_06605	1337255	1338298	-1	hypothetical protein
1318413	1431755	113342	OXZ37359.1	B9N56_06610	1338295	1339740	-1	hypothetical protein
1318413	1431755	113342	OXZ37360.1	B9N56_06615	1339749	1341164	-1	hypothetical protein
1318413	1431755	113342	OXZ37361.1	B9N56_06620	1341167	1342213	-1	hypothetical protein
1318413	1431755	113342	OXZ37362.1	B9N56_06625	1342235	1345675	-1	hypothetical protein
1318413	1431755	113342	OXZ37363.1	B9N56_06630	1345688	1347274	-1	2,3-dihydroxybenzoate-AMP ligase
1318413	1431755	113342	OXZ37364.1	B9N56_06635	1347271	1347957	-1	4-phosphopantetheinyl transferase
1318413	1431755	113342	OXZ37365.1	B9N56_06640	1347959	1348699	-1	thioesterase
1318413	1431755	113342	OXZ37366.1	B9N56_06645	1348820	1349788	-1	3-deoxy-7-phosphoheptulonate synthase
1318413	1431755	113342	OXZ37367.1	B9N56_06650	1349804	1350232	-1	type II 3-dehydroquinate dehydratase
1318413	1431755	113342	OXZ37368.1	B9N56_06655	1350210	1351451	-1	shikimate dehydrogenase
1318413	1431755	113342	OXZ37369.1	B9N56_06660	1351474	1352553	-1	chorismate synthase
1318413	1431755	113342	OXZ37426.1	B9N56_06665	1352540	1353832	-1	3-phosphoshikimate 1-carboxyvinyltransferase
1318413	1431755	113342	OXZ37370.1	B9N56_06670	1353829	1354875	-1	3-dehydroquinate synthase
1318413	1431755	113342	OXZ37371.1	B9N56_06675	1354879	1356240	-1	salicylate synthase
1318413	1431755	113342	OXZ37372.1	B9N56_06680	1356436	1356828	-1	transcriptional regulator
1318413	1431755	113342	OXZ37373.1	B9N56_06685	1357265	1357633	1	plasmid mobilization relaxosome protein MobC
1318413	1431755	113342	OXZ37374.1	B9N56_06690	1357633	1359003	1	endonuclease
1318413	1431755	113342	OXZ37375.1	B9N56_06695	1359062	1359808	-1	ABC transporter permease
1318413	1431755	113342	OXZ37376.1	B9N56_06700	1359823	1360515	-1	ABC transporter permease
1318413	1431755	113342	OXZ37377.1	B9N56_06705	1360517	1361431	-1	bacitracin ABC transporter ATP-binding protein
1318413	1431755	113342	OXZ37378.1	B9N56_06710	1361446	1362603	-1	hypothetical protein
1318413	1431755	113342	OXZ37379.1	B9N56_06715	1362607	1363914	-1	hypothetical protein
1318413	1431755	113342	OXZ37380.1	B9N56_06720	1364553	1364984	-1	hypothetical protein
1318413	1431755	113342	OXZ37381.1	B9N56_06725	1364997	1365260	-1	hypothetical protein
1318413	1431755	113342	OXZ37382.1	B9N56_06730	1365254	1365823	-1	hypothetical protein

1318413	1431755	113342		B9N56_06735	1366162	1366485	1	relaxase
1318413	1431755	113342	OXZ37383.1	B9N56_06740	1366606	1366953	-1	DUF3784 domain-containing protein
1318413	1431755	113342	OXZ37384.1	B9N56_06745	1366961	1368337	-1	hypothetical protein
1318413	1431755	113342	OXZ37385.1	B9N56_06750	1368347	1369141	-1	hypothetical protein
1318413	1431755	113342	OXZ37386.1	B9N56_06755	1369131	1370177	-1	hypothetical protein
1318413	1431755	113342	OXZ37387.1	B9N56_06760	1370179	1371825	-1	hypothetical protein
1318413	1431755	113342	OXZ37388.1	B9N56_06765	1372083	1372292	-1	hypothetical protein
1318413	1431755	113342	OXZ37389.1	B9N56_06770	1372602	1374263	-1	ABC transporter permease
1318413	1431755	113342	OXZ37390.1	B9N56_06775	1374256	1374522	-1	protein
1318413	1431755	113342	OXZ37391.1	B9N56_06780	1374524	1376713	-1	radical SAM protein
1318413	1431755	113342	OXZ37392.1	B9N56_06785	1376700	1376882	-1	hypothetical protein
1318413	1431755	113342		B9N56_06790	1377012	1377617	-1	DNA-binding response regulator
1318413	1431755	113342	OXZ37393.1	B9N56_06795	1377589	1379136	-1	hypothetical protein
1318413	1431755	113342	OXZ37394.1	B9N56_06800	1379450	1380544	-1	single-stranded DNA-binding protein
1318413	1431755	113342	OXZ37395.1	B9N56_06805	1380577	1390998	-1	conjugal transfer protein
1318413	1431755	113342	OXZ37396.1	B9N56_06810	1391191	1391517	-1	terminase large subunit
1318413	1431755	113342	OXZ37397.1	B9N56_06815	1391859	1393565	-1	DNA topoisomerase III
1318413	1431755	113342	OXZ37398.1	B9N56_06820	1393635	1394474	-1	abortive phage infection protein
1318413	1431755	113342	OXZ37399.1	B9N56_06825	1394467	1395060	-1	abortive phage infection protein
1318413	1431755	113342	OXZ37400.1	B9N56_06830	1395375	1396253	-1	bacteriocin
1318413	1431755	113342	OXZ37401.1	B9N56_06835	1396269	1398623	-1	copper amine oxidase
1318413	1431755	113342	OXZ37402.1	B9N56_06840	1398620	1398877	-1	hypothetical protein
1318413	1431755	113342	OXZ37403.1	B9N56_06845	1398897	1401860	-1	hypothetical protein
1318413	1431755	113342	OXZ37404.1	B9N56_06850	1401870	1402835	-1	site-specific DNA-methyltransferase
1318413	1431755	113342	OXZ37405.1	B9N56_06855	1402807	1405302	-1	DUF87 domain-containing protein
1318413	1431755	113342	OXZ37406.1	B9N56_06860	1405299	1405637	-1	conjugal transfer protein
1318413	1431755	113342	OXZ37407.1	B9N56_06865	1405637	1405894	-1	hypothetical protein
1318413	1431755	113342	OXZ37408.1	B9N56_06870	1405912	1406775	-1	hypothetical protein
1318413	1431755	113342	OXZ37409.1	B9N56_06875	1406777	1406992	-1	conjugal transfer protein
1318413	1431755	113342	OXZ37410.1	B9N56_06880	1406993	1407316	-1	single-stranded DNA-binding protein
1318413	1431755	113342	OXZ37411.1	B9N56_06885	1407507	1408370	-1	CAAX protease
1318413	1431755	113342		B9N56_06890	1408522	1409337	-1	conjugal transfer protein TraG
1318413	1431755	113342	OXZ37412.1	B9N56_06895	1409349	1409534	-1	hypothetical protein
1318413	1431755	113342	OXZ37413.1	B9N56_06900	1409693	1410406	-1	replisome organizer
1318413	1431755	113342	OXZ37414.1	B9N56_06905	1410478	1411551	-1	hypothetical protein
1318413	1431755	113342	OXZ37415.1	B9N56_06910	1411701	1412180	-1	replication protein
1318413	1431755	113342	OXZ36991.1	B9N56_06915	1413247	1413577	-1	nickase
1318413	1431755	113342	OXZ36992.1	B9N56_06920	1413911	1414201	-1	hypothetical protein
1318413	1431755	113342	OXZ36993.1	B9N56_06925	1414300	1414521	-1	hypothetical protein
1318413	1431755	113342	OXZ36994.1	B9N56_06930	1414542	1415051	-1	RDD family protein
1318413	1431755	113342	OXZ36995.1	B9N56_06935	1415221	1416297	-1	two-component sensor histidine kinase
1318413	1431755	113342	OXZ36996.1	B9N56_06940	1416287	1416967	-1	DNA-binding response regulator
1318413	1431755	113342	OXZ36997.1	B9N56_06945	1416979	1417746	-1	hypothetical protein
1318413	1431755	113342	OXZ36998.1	B9N56_06950	1417755	1418480	-1	hypothetical protein
1318413	1431755	113342	OXZ36999.1	B9N56_06955	1418461	1419204	-1	ABC transporter ATP-binding protein
1318413	1431755	113342	OXZ37000.1	B9N56_06960	1419437	1419736	-1	hypothetical protein
1318413	1431755	113342	OXZ37001.1	B9N56_06965	1419839	1420276	-1	hypothetical protein
1318413	1431755	113342	OXZ37002.1	B9N56_06970	1420397	1420729	-1	hypothetical protein
1318413	1431755	113342	OXZ37003.1	B9N56_06975	1421029	1422471	-1	histidine kinase
1318413	1431755	113342	OXZ37004.1	B9N56_06980	1422462	1423124	-1	DNA-binding response regulator
1318413	1431755	113342	OXZ37005.1	B9N56_06985	1423249	1423989	-1	lantibiotic ABC transporter permease
1318413	1431755	113342	OXZ37006.1	B9N56_06990	1423995	1424744	-1	lantibiotic ABC transporter permease
1318413	1431755	113342	OXZ37007.1	B9N56_06995	1424737	1425447	-1	lantibiotic ABC transporter ATP-binding protein
1318413	1431755	113342		B9N56_07000	1425648	1427437	-1	recombinase TnpX
1318413	1431755	113342	OXZ37008.1	B9N56_07005	1427477	1428535	-1	conjugal transfer protein TraG
1318413	1431755	113342	OXZ37009.1	B9N56_07010	1428580	1428786	-1	hypothetical protein
1318413	1431755	113342	OXZ37010.1	B9N56_07015	1428984	1429475	-1	hypothetical protein
1318413	1431755	113342	OXZ37011.1	B9N56_07020	1429490	1430347	-1	DNA replication protein
1318413	1431755	113342	OXZ37012.1	B9N56_07025	1430362	1431339	-1	replication initiator RepA
1318413	1431755	113342	OXZ37013.1	B9N56_07030	1431465	1431755	-1	hypothetical protein
1323478	1372292	48814	OXZ37348.1	B9N56_06555	1323478	1323645	-1	aminoacyl-tRNA hydrolase
1323478	1372292	48814	OXZ37349.1	B9N56_06560	1324114	1324521	-1	RNA polymerase subunit sigma
1323478	1372292	48814	OXZ37350.1	B9N56_06565	1325321	1326664	-1	MATE family efflux transporter
1323478	1372292	48814	OXZ37351.1	B9N56_06570	1326676	1328424	-1	ABC transporter ATP-binding protein
1323478	1372292	48814	OXZ37352.1	B9N56_06575	1328378	1330132	-1	ABC transporter ATP-binding protein
1323478	1372292	48814	OXZ37353.1	B9N56_06580	1330281	1331240	1	AraC family transcriptional regulator
1323478	1372292	48814	OXZ37354.1	B9N56_06585	1331302	1332690	-1	ABC transporter ATP-binding protein
1323478	1372292	48814	OXZ37355.1	B9N56_06590	1332701	1333357	-1	cobalt transporter
1323478	1372292	48814	OXZ37356.1	B9N56_06595	1333359	1333940	-1	hypothetical protein
1323478	1372292	48814	OXZ37357.1	B9N56_06600	1333951	1337238	-1	hypothetical protein
1323478	1372292	48814	OXZ37358.1	B9N56_06605	1337255	1338298	-1	hypothetical protein
1323478	1372292	48814	OXZ37359.1	B9N56_06610	1338295	1339740	-1	hypothetical protein
1323478	1372292	48814	OXZ37360.1	B9N56_06615	1339749	1341164	-1	hypothetical protein
1323478	1372292	48814	OXZ37361.1	B9N56_06620	1341167	1342213	-1	hypothetical protein
1323478	1372292	48814	OXZ37362.1	B9N56_06625	1342235	1345675	-1	hypothetical protein
1323478	1372292	48814	OXZ37363.1	B9N56_06630	1345688	1347274	-1	2,3-dihydroxybenzoate-AMP ligase

1323478	1372292	48814	OXZ37364.1	B9N56_06635	1347271	1347957	-1	4-phosphopantetheinyl transferase
1323478	1372292	48814	OXZ37365.1	B9N56_06640	1347959	1348699	-1	thioesterase
1323478	1372292	48814	OXZ37366.1	B9N56_06645	1348820	1349788	-1	3-deoxy-7-phosphoheptulonate synthase
1323478	1372292	48814	OXZ37367.1	B9N56_06650	1349804	1350232	-1	type II 3-dehydroquinate dehydratase
1323478	1372292	48814	OXZ37368.1	B9N56_06655	1350210	1351451	-1	shikimate dehydrogenase
1323478	1372292	48814	OXZ37369.1	B9N56_06660	1351474	1352553	-1	chorismate synthase
1323478	1372292	48814	OXZ37426.1	B9N56_06665	1352540	1353832	-1	3-phosphoshikimate 1-carboxyvinyltransferase
1323478	1372292	48814	OXZ37370.1	B9N56_06670	1353829	1354875	-1	3-dehydroquinate synthase
1323478	1372292	48814	OXZ37371.1	B9N56_06675	1354879	1356240	-1	salicylate synthase
1323478	1372292	48814	OXZ37372.1	B9N56_06680	1356436	1356828	-1	transcriptional regulator
1323478	1372292	48814	OXZ37373.1	B9N56_06685	1357265	1357633	1	plasmid mobilization relaxosome protein MobC
1323478	1372292	48814	OXZ37374.1	B9N56_06690	1357633	1359003	1	endonuclease
1323478	1372292	48814	OXZ37375.1	B9N56_06695	1359062	1359808	-1	ABC transporter permease
1323478	1372292	48814	OXZ37376.1	B9N56_06700	1359823	1360515	-1	ABC transporter permease
1323478	1372292	48814	OXZ37377.1	B9N56_06705	1360517	1361431	-1	bacitracin ABC transporter ATP-binding protein
1323478	1372292	48814	OXZ37378.1	B9N56_06710	1361446	1362603	-1	hypothetical protein
1323478	1372292	48814	OXZ37379.1	B9N56_06715	1362607	1363914	-1	hypothetical protein
1323478	1372292	48814	OXZ37380.1	B9N56_06720	1364553	1364984	-1	hypothetical protein
1323478	1372292	48814	OXZ37381.1	B9N56_06725	1364997	1365260	-1	hypothetical protein
1323478	1372292	48814	OXZ37382.1	B9N56_06730	1365254	1365823	-1	hypothetical protein
1323478	1372292	48814		B9N56_06735	1366162	1366485	1	relaxase
1323478	1372292	48814	OXZ37383.1	B9N56_06740	1366606	1366953	-1	DUF3784 domain-containing protein
1323478	1372292	48814	OXZ37384.1	B9N56_06745	1366961	1368337	-1	hypothetical protein
1323478	1372292	48814	OXZ37385.1	B9N56_06750	1368347	1369141	-1	hypothetical protein
1323478	1372292	48814	OXZ37386.1	B9N56_06755	1369131	1370177	-1	hypothetical protein
1323478	1372292	48814	OXZ37387.1	B9N56_06760	1370179	1371825	-1	hypothetical protein
1323478	1372292	48814	OXZ37388.1	B9N56_06765	1372083	1372292	-1	hypothetical protein
1374256	1401860	27604	OXZ37389.1	B9N56_06770	1372602	1374263	-1	ABC transporter permease
1374256	1401860	27604	OXZ37390.1	B9N56_06775	1374256	1374522	-1	protein
1374256	1401860	27604	OXZ37391.1	B9N56_06780	1374524	1376713	-1	radical SAM protein
1374256	1401860	27604	OXZ37392.1	B9N56_06785	1376700	1376882	-1	hypothetical protein
1374256	1401860	27604		B9N56_06790	1377012	1377617	-1	DNA-binding response regulator
1374256	1401860	27604	OXZ37393.1	B9N56_06795	1377589	1379136	-1	hypothetical protein
1374256	1401860	27604	OXZ37394.1	B9N56_06800	1379450	1380544	-1	single-stranded DNA-binding protein
1374256	1401860	27604	OXZ37395.1	B9N56_06805	1380577	1390998	-1	conjugal transfer protein
1374256	1401860	27604	OXZ37396.1	B9N56_06810	1391191	1391517	-1	terminase large subunit
1374256	1401860	27604	OXZ37397.1	B9N56_06815	1391859	1393565	-1	DNA topoisomerase III
1374256	1401860	27604	OXZ37398.1	B9N56_06820	1393635	1394474	-1	abortive phage infection protein
1374256	1401860	27604	OXZ37399.1	B9N56_06825	1394467	1395060	-1	abortive phage infection protein
1374256	1401860	27604	OXZ37400.1	B9N56_06830	1395375	1396253	-1	bacteriocin
1374256	1401860	27604	OXZ37401.1	B9N56_06835	1396269	1398623	-1	copper amine oxidase
1374256	1401860	27604	OXZ37402.1	B9N56_06840	1398620	1398877	-1	hypothetical protein
1374256	1401860	27604	OXZ37403.1	B9N56_06845	1398897	1401860	-1	hypothetical protein
1405299	1420729	15430	OXZ37405.1	B9N56_06855	1402807	1405302	-1	DUF87 domain-containing protein
1405299	1420729	15430	OXZ37406.1	B9N56_06860	1405299	1405637	-1	conjugal transfer protein
1405299	1420729	15430	OXZ37407.1	B9N56_06865	1405637	1405894	-1	hypothetical protein
1405299	1420729	15430	OXZ37408.1	B9N56_06870	1405912	1406775	-1	hypothetical protein
1405299	1420729	15430	OXZ37409.1	B9N56_06875	1406777	1406992	-1	conjugal transfer protein
1405299	1420729	15430	OXZ37410.1	B9N56_06880	1406993	1407316	-1	single-stranded DNA-binding protein
1405299	1420729	15430	OXZ37411.1	B9N56_06885	1407507	1408370	-1	CAAX protease
1405299	1420729	15430		B9N56_06890	1408522	1409337	-1	conjugal transfer protein TraG
1405299	1420729	15430	OXZ37412.1	B9N56_06895	1409349	1409534	-1	hypothetical protein
1405299	1420729	15430	OXZ37413.1	B9N56_06900	1409693	1410406	-1	replisome organizer
1405299	1420729	15430	OXZ37414.1	B9N56_06905	1410478	1411551	-1	hypothetical protein
1405299	1420729	15430	OXZ37415.1	B9N56_06910	1411701	1412180	-1	replication protein
1405299	1420729	15430	OXZ36991.1	B9N56_06915	1413247	1413577	-1	nickase
1405299	1420729	15430	OXZ36992.1	B9N56_06920	1413911	1414201	-1	hypothetical protein
1405299	1420729	15430	OXZ36993.1	B9N56_06925	1414300	1414521	-1	hypothetical protein
1405299	1420729	15430	OXZ36994.1	B9N56_06930	1414542	1415051	-1	RDD family protein
1405299	1420729	15430	OXZ36995.1	B9N56_06935	1415221	1416297	-1	two-component sensor histidine kinase
1405299	1420729	15430	OXZ36996.1	B9N56_06940	1416287	1416967	-1	DNA-binding response regulator
1405299	1420729	15430	OXZ36997.1	B9N56_06945	1416979	1417746	-1	hypothetical protein
1405299	1420729	15430	OXZ36998.1	B9N56_06950	1417755	1418480	-1	hypothetical protein
1405299	1420729	15430	OXZ36999.1	B9N56_06955	1418461	1419204	-1	ABC transporter ATP-binding protein
1405299	1420729	15430	OXZ37000.1	B9N56_06960	1419437	1419736	-1	hypothetical protein
1405299	1420729	15430	OXZ37001.1	B9N56_06965	1419839	1420276	-1	hypothetical protein
1405299	1420729	15430	OXZ37002.1	B9N56_06970	1420397	1420729	-1	hypothetical protein
1557581	1566142	8561		B9N56_07590	1557581	1558190	-1	SAM-dependent methyltransferase
1557581	1566142	8561	OXZ36941.1	B9N56_07595	1558406	1559155	-1	phosphorylase
1557581	1566142	8561	OXZ36942.1	B9N56_07600	1559285	1560886	-1	macrolide ABC transporter ATP-binding protein
1557581	1566142	8561	OXZ36943.1	B9N56_07605	1561291	1561905	-1	hypothetical protein
1557581	1566142	8561	OXZ36944.1	B9N56_07610	1562111	1563475	-1	ABC transporter ATP-binding protein
1557581	1566142	8561	OXZ36945.1	B9N56_07615	1563469	1564158	-1	cobalt ABC transporter permease
1557581	1566142	8561	OXZ36946.1	B9N56_07620	1564158	1564739	-1	hypothetical protein
1557581	1566142	8561	OXZ36960.1	B9N56_07625	1564799	1566142	-1	MATE family efflux transporter
1575901	1586348	10447		B9N56_07675	1575901	1576179	-1	AraC family transcriptional regulator

1575901	1586348	10447		B9N56_07680	1576714	1576923	-1	hypothetical protein
1575901	1586348	10447	OXZ36956.1	B9N56_07685	1577181	1577573	-1	hypothetical protein
1575901	1586348	10447	OXZ36961.1	B9N56_07690	1578112	1578495	-1	hypothetical protein
1575901	1586348	10447	OXZ36957.1	B9N56_07695	1579994	1580644	-1	hypothetical protein
1575901	1586348	10447	OXZ36958.1	B9N56_07700	1580950	1581436	-1	phosphorylase
1575901	1586348	10447	OXZ36826.1	B9N56_07705	1582716	1585109	-1	hypothetical protein
1575901	1586348	10447		B9N56_07710	1585102	1585322	-1	transcriptional regulator
1575901	1586348	10447	OXZ36827.1	B9N56_07715	1585737	1586348	-1	SAM-dependent methyltransferase
1604844	1611673	6829	OXZ36840.1	B9N56_07790	1604844	1605143	1	plasmid mobilization relaxosome protein MobC
1604844	1611673	6829		B9N56_07795	1605202	1605936	1	relaxase
1604844	1611673	6829		B9N56_07800	1606003	1606533	1	hypothetical protein
1604844	1611673	6829	OXZ36841.1	B9N56_07805	1606842	1609601	-1	hypothetical protein
1604844	1611673	6829	OXZ36842.1	B9N56_07810	1609861	1610073	-1	DNA-binding protein
1604844	1611673	6829		B9N56_07815	1610249	1611089	-1	abortive phage infection protein
1604844	1611673	6829	OXZ36843.1	B9N56_07820	1611086	1611673	-1	transcriptional regulator

Table S3C: Predicted genomic islands in the genome of *F. magna* 09T408

Shown are genes of genomic islands predicted by IslandViewer4

Island start	Island end	Length	Gene name	Locus	Gene start	Gene end	Strand	Product
7930	14062	6132	OXZ32095.1	B9N57_00015	7930	8017	1	hypothetical protein
7930	14062	6132	OXZ32096.1	B9N57_00020	8081	8533	-1	small-multidrug export protein
7930	14062	6132	OXZ32097.1	B9N57_00025	8542	9192	-1	phosphatase
7930	14062	6132	OXZ32098.1	B9N57_00030	9354	10586	1	aminopeptidase
7930	14062	6132	OXZ32099.1	B9N57_00035	10586	12382	1	oligoendopeptidase F
7930	14062	6132	OXZ32100.1	B9N57_00040	12433	13317	1	antibiotic ABC transporter ATP-binding protein
7930	14062	6132	OXZ32101.1	B9N57_00045	13310	14062	1	antibiotic ABC transporter permease
7930	14062	6132	OXZ32102.1	B9N57_00050	14052	14723	1	antibiotic ABC transporter permease
72169	82578	10409	OXZ31830.1	B9N57_00255	72169	73038	1	hypothetical protein
72169	82578	10409	OXZ31831.1	B9N57_00260	73025	75013	1	exopolyphosphatase
72169	82578	10409	OXZ31832.1	B9N57_00265	74994	75440	1	50S ribosomal protein L9
72169	82578	10409	OXZ31833.1	B9N57_00270	75474	76811	1	replicative DNA helicase
72169	82578	10409	OXZ31834.1	B9N57_00275	76808	77371	1	N-acetyltransferase
72169	82578	10409	OXZ31835.1	B9N57_00280	77376	78134	1	DNA replication protein
72169	82578	10409	OXZ31836.1	B9N57_00285	78137	79114	1	DNA replication protein DnaC
72169	82578	10409	OXZ31837.1	B9N57_00290	79111	79755	1	NAD(P)H-hydrate epimerase
72169	82578	10409	OXZ31838.1	B9N57_00295	80595	81137	1	ECF transporter S component
72169	82578	10409	OXZ31839.1	B9N57_00300	81121	81807	1	radical SAM protein
72169	82578	10409	OXZ31840.1	B9N57_00305	81874	82578	1	hypothetical protein
182046	186690	4644	OXZ31460.1	B9N57_00845	182046	182687	-1	hemolysin III
182046	186690	4644	OXZ31461.1	B9N57_00850	182841	183344	1	acetyltransferase
182046	186690	4644	OXZ31462.1	B9N57_00855	183344	184078	1	hydrolase
182046	186690	4644	OXZ31379.1	B9N57_00860	185725	186096	1	hypothetical protein
182046	186690	4644	OXZ31450.1	B9N57_00865	186343	186690	-1	toxin MazF
315622	325971	10349		B9N57_01460	315622	315807	1	DUF4926 domain-containing protein
315622	325971	10349	OXZ31235.1	B9N57_01465	316623	319379	1	Cas3
315622	325971	10349	OXZ31236.1	B9N57_01470	319372	321093	1	hypothetical protein
315622	325971	10349	OXZ31237.1	B9N57_01475	321105	321710	1	type I-E CRISPR-associated protein Cse2/CasB
315622	325971	10349	OXZ31238.1	B9N57_01480	321694	322776	1	Cas7/Cse4/CasC
315622	325971	10349	OXZ31239.1	B9N57_01485	322786	323490	1	type I-E CRISPR-associated protein Cas5/CasD
315622	325971	10349	OXZ31240.1	B9N57_01490	323494	324147	1	Cas6/Cse3/CasE
315622	325971	10349	OXZ31241.1	B9N57_01495	324144	325082	1	Cas1
315622	325971	10349	OXZ31242.1	B9N57_01500	325084	325971	1	Cas2
750468	761175	10707	OXZ30582.1	B9N57_03715	750468	751166	1	DNA-binding response regulator
750468	761175	10707	OXZ30583.1	B9N57_03720	751207	751791	1	peptidylprolyl isomerase
750468	761175	10707	OXZ30584.1	B9N57_03725	751793	752425	1	thymidine kinase
750468	761175	10707	OXZ30585.1	B9N57_03730	752447	752917	-1	S-ribosylhomocysteine lyase
750468	761175	10707	OXZ30586.1	B9N57_03735	752926	754029	-1	rod shape-determining protein RodA
750468	761175	10707	OXZ30587.1	B9N57_03740	754104	754937	1	endonuclease IV
750468	761175	10707	OXZ30588.1	B9N57_03745	754918	755544	1	endonuclease III
750468	761175	10707	OXZ30589.1	B9N57_03750	755612	756532	1	aspartate carbamoyltransferase
750468	761175	10707	OXZ30590.1	B9N57_03755	756532	756960	1	subunit
750468	761175	10707	OXZ30591.1	B9N57_03760	756960	758156	1	dihydroorotase
750468	761175	10707	OXZ30592.1	B9N57_03765	758143	758994	1	orotidine-5'-phosphate decarboxylase
750468	761175	10707	OXZ30593.1	B9N57_03770	758987	759703	1	subunit
750468	761175	10707	OXZ30594.1	B9N57_03775	759696	760592	1	subunit
750468	761175	10707	OXZ30595.1	B9N57_03780	760603	761175	1	orotate phosphoribosyltransferase
775045	779501	4456	OXZ30608.1	B9N57_03845	775045	775842	1	hypothetical protein
775045	779501	4456	OXZ30609.1	B9N57_03850	775926	777383	1	serine/threonine protein kinase
775045	779501	4456	OXZ30610.1	B9N57_03855	777385	778059	1	protein phosphatase
775045	779501	4456	OXZ30611.1	B9N57_03860	778116	779501	1	methylthiotransferase MiaB
812488	816921	4433	OXZ30642.1	B9N57_04025	812488	812823	-1	histidine triad nucleotide-binding protein
812488	816921	4433	OXZ30643.1	B9N57_04030	812870	814162	-1	C(2))- methylthiotransferase MtaB
812488	816921	4433	OXZ30644.1	B9N57_04035	814165	814887	-1	rRNA methyltransferase
812488	816921	4433	OXZ30645.1	B9N57_04040	814880	815803	-1	50S ribosomal protein L11 methyltransferase
812488	816921	4433	OXZ30646.1	B9N57_04045	815803	816921	-1	molecular chaperone DnaJ
983705	989288	5583		B9N57_04800	983705	984169	-1	IS200/IS605 family transposase
983705	989288	5583	OXZ30387.1	B9N57_04805	984389	984676	-1	cupin
983705	989288	5583	OXZ30388.1	B9N57_04810	984743	985315	-1	uracil-DNA glycosylase
983705	989288	5583		B9N57_04815	985454	985657	-1	hypothetical protein
983705	989288	5583		B9N57_04820	985647	985930	-1	hypothetical protein
983705	989288	5583		B9N57_04825	985932	986735	-1	ABC transporter
983705	989288	5583	OXZ30389.1	B9N57_04830	987500	988258	-1	SAM-dependent methyltransferase
983705	989288	5583	OXZ30390.1	B9N57_04835	988353	988946	1	transcriptional regulator
983705	989288	5583	OXZ30391.1	B9N57_04840	989097	989288	-1	hypothetical protein
1455800	1460804	5004		B9N57_07000	1455800	1456095	-1	AraC family transcriptional regulator
1455800	1460804	5004		B9N57_07005	1456206	1456360	-1	recombinase
1455800	1460804	5004	OXZ29994.1	B9N57_07010	1456581	1457231	-1	hypothetical protein
1455800	1460804	5004	OXZ29995.1	B9N57_07015	1457537	1458286	-1	phosphorylase
1455800	1460804	5004	OXZ29996.1	B9N57_07020	1458439	1458867	-1	hypothetical protein
1455800	1460804	5004	OXZ29997.1	B9N57_07025	1459220	1460248	-1	carboxypeptidase
1455800	1460804	5004		B9N57_07030	1460566	1460804	1	hypothetical protein

1473159	1506658	33499	OXZ30007.1	B9N57_07090	1473159	1474130	1	AraC family transcriptional regulator
1473159	1506658	33499	OXZ30008.1	B9N57_07095	1474325	1474681	1	plasmid mobilization relaxosome protein MobC
1473159	1506658	33499	OXZ30009.1	B9N57_07100	1474684	1476015	1	endonuclease
1473159	1506658	33499	OXZ30010.1	B9N57_07105	1476084	1476743	-1	DNA-binding protein
1473159	1506658	33499	OXZ30011.1	B9N57_07110	1476785	1485514	-1	helicase
1473159	1506658	33499	OXZ30088.1	B9N57_07115	1485598	1487133	-1	AAA family ATPase
1473159	1506658	33499	OXZ30089.1	B9N57_07120	1487215	1488927	-1	DNA topoisomerase III
1473159	1506658	33499	OXZ30012.1	B9N57_07125	1489022	1490557	-1	cell surface protein
1473159	1506658	33499	OXZ30013.1	B9N57_07130	1490532	1490771	-1	conjugal transfer protein
1473159	1506658	33499	OXZ30014.1	B9N57_07135	1490783	1492915	-1	CHAP domain-containing protein
1473159	1506658	33499	OXZ30015.1	B9N57_07140	1492919	1495348	-1	conjugal transfer protein TraE
1473159	1506658	33499	OXZ30016.1	B9N57_07145	1495260	1495649	-1	conjugal transfer protein
1473159	1506658	33499	OXZ30017.1	B9N57_07150	1495653	1495916	-1	conjugal transfer protein
1473159	1506658	33499	OXZ30018.1	B9N57_07155	1495934	1496797	-1	hypothetical protein
1473159	1506658	33499	OXZ30019.1	B9N57_07160	1496817	1497032	-1	conjugal transfer protein
1473159	1506658	33499	OXZ30020.1	B9N57_07165	1497036	1497347	-1	single-stranded DNA-binding protein
1473159	1506658	33499		B9N57_07170	1497513	1498325	-1	conjugal transfer protein TraG
1473159	1506658	33499	OXZ30021.1	B9N57_07175	1498337	1498522	-1	hypothetical protein
1473159	1506658	33499	OXZ30022.1	B9N57_07180	1498634	1499641	-1	DNA-binding protein
1473159	1506658	33499	OXZ30023.1	B9N57_07185	1499663	1499986	-1	hypothetical protein
1473159	1506658	33499	OXZ30024.1	B9N57_07190	1500160	1500912	-1	replisome organizer
1473159	1506658	33499	OXZ30025.1	B9N57_07195	1500915	1502567	-1	MobA/MobL protein
1473159	1506658	33499	OXZ30026.1	B9N57_07200	1502814	1503143	-1	hypothetical protein
1473159	1506658	33499	OXZ30027.1	B9N57_07205	1503228	1503644	-1	RNA polymerase subunit sigma-70
1473159	1506658	33499	OXZ30028.1	B9N57_07210	1503958	1505343	-1	ABC transporter
1473159	1506658	33499	OXZ30029.1	B9N57_07215	1505340	1506068	-1	transporter
1473159	1506658	33499	OXZ30030.1	B9N57_07220	1506068	1506658	-1	hypothetical protein
1535253	1542597	7344		B9N57_07355	1535253	1535486	-1	protein CapI
1535253	1542597	7344	OXZ30056.1	B9N57_07360	1535491	1536513	-1	hypothetical protein
1535253	1542597	7344	OXZ30057.1	B9N57_07365	1536548	1538062	-1	hypothetical protein
1535253	1542597	7344	OXZ30058.1	B9N57_07370	1538077	1539279	-1	hypothetical protein
1535253	1542597	7344	OXZ30059.1	B9N57_07375	1539314	1540381	-1	glycosyl transferase family 2
1535253	1542597	7344	OXZ30060.1	B9N57_07380	1540374	1541537	-1	capsule biosynthesis protein
1535253	1542597	7344	OXZ30061.1	B9N57_07385	1541551	1542597	-1	hypothetical protein
1535253	1542597	7344	OXZ30062.1	B9N57_07390	1542594	1543589	-1	ornithine cyclodeaminase
1638880	1660212	21332	OXZ29911.1	B9N57_07815	1637061	1638896	-1	multidrug ABC transporter
1638880	1660212	21332	OXZ29912.1	B9N57_07820	1638880	1640682	-1	multidrug ABC transporter
1638880	1660212	21332	OXZ29913.1	B9N57_07825	1640762	1641427	-1	histidine kinase
1638880	1660212	21332	OXZ29914.1	B9N57_07830	1641435	1642130	-1	DNA-binding response regulator
1638880	1660212	21332	OXZ29915.1	B9N57_07835	1642213	1643076	-1	UTP--glucose-1-phosphate uridylyltransferase
1638880	1660212	21332	OXZ29916.1	B9N57_07840	1643801	1646386	-1	ATP-dependent chaperone ClpB
1638880	1660212	21332	OXZ29917.1	B9N57_07845	1646405	1647349	-1	heat-shock protein
1638880	1660212	21332	OXZ29918.1	B9N57_07850	1647523	1648188	-1	nitroreductase
1638880	1660212	21332	OXZ29919.1	B9N57_07855	1648434	1649201	-1	hypothetical protein
1638880	1660212	21332	OXZ29920.1	B9N57_07860	1650005	1650556	1	hypothetical protein
1638880	1660212	21332	OXZ29921.1	B9N57_07865	1650617	1650829	1	hypothetical protein
1638880	1660212	21332	OXZ29922.1	B9N57_07870	1650909	1652057	1	MFS transporter
1638880	1660212	21332	OXZ29923.1	B9N57_07875	1652136	1652633	-1	hypothetical protein
1638880	1660212	21332	OXZ29924.1	B9N57_07880	1652669	1653142	-1	peptidase M50
1638880	1660212	21332	OXZ29925.1	B9N57_07885	1653629	1654051	-1	hypothetical protein
1638880	1660212	21332	OXZ29926.1	B9N57_07890	1654096	1655031	-1	TIGR01212 family radical SAM protein
1638880	1660212	21332	OXZ29927.1	B9N57_07895	1655024	1655464	-1	peroxiredoxin
1638880	1660212	21332	OXZ29928.1	B9N57_07900	1655457	1656293	-1	hydrolase
1638880	1660212	21332	OXZ29929.1	B9N57_07905	1656283	1656594	-1	DUF1292 domain-containing protein
1638880	1660212	21332	OXZ29930.1	B9N57_07910	1656607	1657998	-1	aminoacyl-histidine dipeptidase
1638880	1660212	21332	OXZ29931.1	B9N57_07915	1658063	1659181	-1	hypothetical protein
1638880	1660212	21332	OXZ29932.1	B9N57_07920	1659223	1660212	-1	peptide chain release factor 2
1720515	1725038	4523	OXZ29867.1	B9N57_08165	1720515	1720637	1	hypothetical protein
1720515	1725038	4523	OXZ29771.1	B9N57_08170	1721099	1721893	-1	protein
1720515	1725038	4523	OXZ29772.1	B9N57_08175	1721905	1722924	-1	iron ABC transporter
1720515	1725038	4523	OXZ29773.1	B9N57_08180	1722926	1723972	-1	iron ABC transporter
1720515	1725038	4523	OXZ29774.1	B9N57_08185	1723989	1725038	-1	iron ABC transporter permease
1816636	1830054	13418	OXZ29865.1	B9N57_08675	1816636	1822018	1	peptidase S8
1816636	1830054	13418		B9N57_08720	1823055	1823882	-1	N-acetylmuramoyl-L-alanine amidase
1816636	1830054	13418	OXZ29745.1	B9N57_08715	1824883	1825979	1	elongation factor Tu
1816636	1830054	13418	OXZ29746.1	B9N57_08695	1826980	1827071	-1	hypothetical protein
1816636	1830054	13418	OXZ29747.1	B9N57_08700	1827266	1827805	-1	transposase
1816636	1830054	13418	OXZ29748.1	B9N57_08705	1828247	1828466	1	hypothetical protein
1816636	1830054	13418	OXZ29740.1	B9N57_08750	1829467	1830054	-1	hypothetical protein

Table S3D: Predicted genomic islands in the genome of *"F. nericia"* 09T494

Shown are genes of genomic islands predicted by IslandViewer4

Island start	Island end	Length	Gene name	Locus	Gene start	Gene end	Strand	Product
4923	16807	11884	OXZ39974.1	B9N50_00015	4923	9083	-1	phage tail tape measure protein
4923	16807	11884	OXZ39975.1	B9N50_00020	9307	9636	-1	hypothetical protein
4923	16807	11884	OXZ39976.1	B9N50_00025	9646	10290	-1	hypothetical protein
4923	16807	11884	OXZ39977.1	B9N50_00030	10302	10745	-1	hypothetical protein
4923	16807	11884	OXZ39978.1	B9N50_00035	10742	11089	-1	hypothetical protein
4923	16807	11884	OXZ39979.1	B9N50_00040	11086	11427	-1	hypothetical protein
4923	16807	11884	OXZ39980.1	B9N50_00045	11427	11723	-1	hypothetical protein
4923	16807	11884	OXZ39981.1	B9N50_00050	11723	12904	-1	phage major capsid protein
4923	16807	11884	OXZ39982.1	B9N50_00055	12907	13638	-1	hypothetical protein
4923	16807	11884	OXZ39983.1	B9N50_00060	13711	14841	-1	hypothetical protein
4923	16807	11884	OXZ39984.1	B9N50_00065	14829	16514	-1	hypothetical protein
4923	16807	11884	OXZ39985.1	B9N50_00070	16511	16807	-1	hypothetical protein
4923	16807	11884	OXZ39986.1	B9N50_00075	16794	17708	-1	hypothetical protein
173877	193778	19901	OXZ40153.1	B9N50_00950	173877	174989	-1	hypothetical protein
173877	193778	19901	OXZ40154.1	B9N50_00955	175269	179723	-1	hypothetical protein
173877	193778	19901	OXZ40155.1	B9N50_00960	180154	181461	-1	hypothetical protein
173877	193778	19901	OXZ40156.1	B9N50_00965	181485	183122	-1	hypothetical protein
173877	193778	19901	OXZ40157.1	B9N50_00970	183132	185336	-1	hypothetical protein
173877	193778	19901	OXZ40158.1	B9N50_00975	185254	185979	-1	hypothetical protein
173877	193778	19901	OXZ40159.1	B9N50_00980	185960	187936	-1	hypothetical protein
173877	193778	19901	OXZ40160.1	B9N50_00985	188155	188784	1	hypothetical protein
173877	193778	19901	OXZ40161.1	B9N50_00990	189230	192190	1	restriction endonuclease
173877	193778	19901	OXZ40162.1	B9N50_00995	192183	193778	1	hypothetical protein
335804	339866	4062	OXZ39656.1	B9N50_01695	335804	336445	-1	hemolysin III
335804	339866	4062	OXZ39657.1	B9N50_01700	336598	337101	1	acetyltransferase
335804	339866	4062	OXZ39658.1	B9N50_01705	337101	337835	1	hydrolase
335804	339866	4062	OXZ39659.1	B9N50_01710	338290	338865	1	cell filamentation protein Fic
335804	339866	4062	OXZ39685.1	B9N50_01715	339519	339866	-1	toxin MazF
1341275	1417222	75947	OXZ37862.1	B9N50_06755	1341275	1342078	-1	DNA-binding protein
1341275	1417222	75947	OXZ37863.1	B9N50_06760	1342268	1343788	-1	conjugal transfer protein
1341275	1417222	75947	OXZ37864.1	B9N50_06765	1343781	1345526	-1	recombinase
1341275	1417222	75947	OXZ37865.1	B9N50_06770	1345519	1347237	-1	recombinase
1341275	1417222	75947	OXZ37866.1	B9N50_06775	1347336	1347503	-1	aminoacyl-tRNA hydrolase
1341275	1417222	75947	OXZ37867.1	B9N50_06780	1347921	1348331	-1	RNA polymerase subunit sigma
1341275	1417222	75947	OXZ37868.1	B9N50_06785	1348569	1349933	-1	MATE family efflux transporter
1341275	1417222	75947	OXZ37869.1	B9N50_06790	1350018	1351760	-1	ABC transporter ATP-binding protein
1341275	1417222	75947	OXZ37870.1	B9N50_06795	1351762	1353474	-1	ABC transporter ATP-binding protein
1341275	1417222	75947	OXZ37871.1	B9N50_06800	1353478	1354893	-1	ABC transporter ATP-binding protein
1341275	1417222	75947	OXZ37872.1	B9N50_06805	1354906	1355592	-1	cobalt transporter
1341275	1417222	75947	OXZ37873.1	B9N50_06810	1355593	1356186	-1	hypothetical protein
1341275	1417222	75947	OXZ37874.1	B9N50_06815	1356358	1356969	-1	TetR family transcriptional regulator
1341275	1417222	75947	OXZ37875.1	B9N50_06820	1357080	1357736	-1	hypothetical protein
1341275	1417222	75947	OXZ37876.1	B9N50_06825	1357760	1358518	-1	hypothetical protein
1341275	1417222	75947	OXZ37877.1	B9N50_06830	1358540	1359931	-1	hypothetical protein
1341275	1417222	75947	OXZ37878.1	B9N50_06835	1359999	1365371	-1	non-ribosomal peptide synthetase
1341275	1417222	75947	OXZ37879.1	B9N50_06840	1365391	1366440	-1	oxidoreductase
1341275	1417222	75947	OXZ37880.1	B9N50_06845	1366442	1367515	-1	hypothetical protein
1341275	1417222	75947	OXZ37881.1	B9N50_06850	1367512	1368888	-1	hypothetical protein
1341275	1417222	75947	OXZ37882.1	B9N50_06855	1368913	1370370	-1	peptide synthetase
1341275	1417222	75947	OXZ37883.1	B9N50_06860	1370367	1371431	-1	oxidoreductase
1341275	1417222	75947	OXZ37884.1	B9N50_06865	1371454	1377627	-1	non-ribosomal peptide synthetase
1341275	1417222	75947	OXZ37885.1	B9N50_06870	1377644	1379233	-1	2,3-dihydroxybenzoate-AMP ligase
1341275	1417222	75947	OXZ37886.1	B9N50_06875	1379252	1379926	-1	4'-phosphopantetheinyl transferase
1341275	1417222	75947	OXZ37887.1	B9N50_06880	1379904	1380662	-1	thioesterase
1341275	1417222	75947	OXZ37888.1	B9N50_06885	1380819	1381190	-1	transcriptional regulator
1341275	1417222	75947	OXZ37889.1	B9N50_06890	1381661	1381801	-1	diacylglyceryl transferase
1341275	1417222	75947	OXZ37890.1	B9N50_06895	1381850	1382218	1	plasmid mobilization relaxosome protein MobC
1341275	1417222	75947	OXZ37891.1	B9N50_06900	1382218	1383588	1	endonuclease
1341275	1417222	75947	OXZ37892.1	B9N50_06905	1383719	1384861	-1	single-stranded DNA-binding protein
1341275	1417222	75947	OXZ37893.1	B9N50_06910	1384895	1395817	-1	conjugal transfer protein
1341275	1417222	75947	OXZ37894.1	B9N50_06915	1396011	1396337	-1	terminase large subunit
1341275	1417222	75947	OXZ37895.1	B9N50_06920	1396678	1398384	-1	DNA topoisomerase III
1341275	1417222	75947	OXZ37896.1	B9N50_06925	1398801	1399679	-1	bacteriocin
1341275	1417222	75947	OXZ37897.1	B9N50_06930	1399695	1402049	-1	copper amine oxidase
1341275	1417222	75947	OXZ37898.1	B9N50_06935	1402046	1402303	-1	hypothetical protein
1341275	1417222	75947	OXZ37899.1	B9N50_06940	1402324	1405290	-1	hypothetical protein
1341275	1417222	75947	OXZ37900.1	B9N50_06945	1405300	1406265	-1	site-specific DNA-methyltransferase
1341275	1417222	75947	OXZ37901.1	B9N50_06950	1406237	1408732	-1	conjugal transfer protein TraE
1341275	1417222	75947	OXZ37902.1	B9N50_06955	1408729	1409067	-1	conjugal transfer protein
1341275	1417222	75947	OXZ37903.1	B9N50_06960	1409067	1409324	-1	hypothetical protein
1341275	1417222	75947	OXZ37904.1	B9N50_06965	1409342	1410205	-1	hypothetical protein
1341275	1417222	75947	OXZ37905.1	B9N50_06970	1410207	1410422	-1	conjugal transfer protein
1341275	1417222	75947	OXZ37906.1	B9N50_06975	1410423	1410746	-1	single-stranded DNA-binding protein
1341275	1417222	75947	OXZ37907.1	B9N50_06980	1410937	1411803	-1	CAAX protease
1341275	1417222	75947	OXZ37908.1	B9N50_06985	1411955	1413757	-1	conjugal transfer protein TraG
1341275	1417222	75947	OXZ37909.1	B9N50_06990	1413802	1414008	-1	hypothetical protein
1341275	1417222	75947	OXZ37910.1	B9N50_06995	1414207	1414698	-1	hypothetical protein
1341275	1417222	75947	OXZ38105.1	B9N50_07000	1414713	1415570	-1	DNA replication protein
1341275	1417222	75947	OXZ37911.1	B9N50_07005	1415585	1416562	-1	replication initiator RepA
1341275	1417222	75947	OXZ37912.1	B9N50_07010	1416649	1416939	-1	hypothetical protein
1341275	1417222	75947	OXZ38106.1	B9N50_07015	1416983	1417222	-1	hypothetical protein
1382218	1406265	24047	OXZ37891.1	B9N50_06900	1382218	1383588	1	endonuclease
1382218	1406265	24047	OXZ37892.1	B9N50_06905	1383719	1384861	-1	single-stranded DNA-binding protein

1382218	1406265	24047	OXZ37893.1	B9N50_06910	1384895	1395817	-1	conjugal transfer protein
1382218	1406265	24047	OXZ37894.1	B9N50_06915	1396011	1396337	-1	terminase large subunit
1382218	1406265	24047	OXZ37895.1	B9N50_06920	1396678	1398384	-1	DNA topoisomerase III
1382218	1406265	24047	OXZ37896.1	B9N50_06925	1398801	1399679	-1	bacteriocin
1382218	1406265	24047	OXZ37897.1	B9N50_06930	1399695	1402049	-1	copper amine oxidase
1382218	1406265	24047	OXZ37898.1	B9N50_06935	1402046	1402303	-1	hypothetical protein
1382218	1406265	24047	OXZ37899.1	B9N50_06940	1402324	1405290	-1	hypothetical protein
1382218	1406265	24047	OXZ37900.1	B9N50_06945	1405300	1406265	-1	site-specific DNA-methyltransferase
1382218	1406265	24047	OXZ37901.1	B9N50_06950	1406237	1408732	-1	conjugal transfer protein TraE
1408729	1416562	7833	OXZ37901.1	B9N50_06950	1406237	1408732	-1	conjugal transfer protein TraE
1408729	1416562	7833	OXZ37902.1	B9N50_06955	1408729	1409067	-1	conjugal transfer protein
1408729	1416562	7833	OXZ37903.1	B9N50_06960	1409067	1409324	-1	hypothetical protein
1408729	1416562	7833	OXZ37904.1	B9N50_06965	1409342	1410205	-1	hypothetical protein
1408729	1416562	7833	OXZ37905.1	B9N50_06970	1410207	1410422	-1	conjugal transfer protein
1408729	1416562	7833	OXZ37906.1	B9N50_06975	1410423	1410746	-1	single-stranded DNA-binding protein
1408729	1416562	7833	OXZ37907.1	B9N50_06980	1410937	1411803	-1	CAAX protease
1408729	1416562	7833	OXZ37908.1	B9N50_06985	1411955	1413757	-1	conjugal transfer protein TraG
1408729	1416562	7833	OXZ37909.1	B9N50_06990	1413802	1414008	-1	hypothetical protein
1408729	1416562	7833	OXZ37910.1	B9N50_06995	1414207	1414698	-1	hypothetical protein
1408729	1416562	7833	OXZ38105.1	B9N50_07000	1414713	1415570	-1	DNA replication protein
1408729	1416562	7833	OXZ37911.1	B9N50_07005	1415585	1416562	-1	replication initiator RepA
1551711	1651998	100287	OXZ38035.1	B9N50_07665	1551711	1553102	-1	ABC transporter
1551711	1651998	100287	OXZ38036.1	B9N50_07670	1553099	1553827	-1	transporter
1551711	1651998	100287	OXZ38037.1	B9N50_07675	1553827	1554417	-1	hypothetical protein
1551711	1651998	100287	OXZ38038.1	B9N50_07680	1554448	1556169	-1	ABC transporter permease
1551711	1651998	100287	OXZ38039.1	B9N50_07685	1556169	1557935	-1	ABC transporter permease
1551711	1651998	100287	OXZ38040.1	B9N50_07690	1558046	1558660	-1	TetR family transcriptional regulator
1551711	1651998	100287	OXZ38041.1	B9N50_07695	1558956	1559576	-1	phosphomethylpyrimidine kinase
1551711	1651998	100287		B9N50_07700	1559626	1560121	-1	terminase
1551711	1651998	100287		B9N50_07705	1560312	1561474	1	IS110 family transposase
1551711	1651998	100287	OXZ38042.1	B9N50_07710	1562235	1563971	-1	multidrug ABC transporter ATP-binding protein
1551711	1651998	100287	OXZ38043.1	B9N50_07715	1563971	1565686	-1	ABC transporter ATP-binding protein
1551711	1651998	100287	OXZ38044.1	B9N50_07720	1565690	1567171	-1	ABC transporter
1551711	1651998	100287	OXZ38111.1	B9N50_07725	1567156	1567836	-1	ABC transporter permease
1551711	1651998	100287	OXZ38045.1	B9N50_07730	1567851	1568480	-1	hypothetical protein
1551711	1651998	100287	OXZ38046.1	B9N50_07735	1568470	1569051	-1	TetR family transcriptional regulator
1551711	1651998	100287	OXZ38047.1	B9N50_07740	1569738	1570094	1	plasmid mobilization relaxosome protein MobC
1551711	1651998	100287	OXZ38048.1	B9N50_07745	1570283	1570792	-1	hypothetical protein
1551711	1651998	100287		B9N50_07750	1570932	1571213	-1	hypothetical protein
1551711	1651998	100287	OXZ38049.1	B9N50_07755	1571216	1571587	-1	transcriptional regulator
1551711	1651998	100287	OXZ38050.1	B9N50_07760	1571877	1572338	-1	flavodoxin
1551711	1651998	100287	OXZ38112.1	B9N50_07765	1572742	1573860	-1	alpha/beta hydrolase
1551711	1651998	100287	OXZ38051.1	B9N50_07770	1573866	1574123	-1	AbrB family transcriptional regulator
1551711	1651998	100287	OXZ38052.1	B9N50_07775	1574772	1575140	-1	EXLDI protein
1551711	1651998	100287		B9N50_07780	1575327	1575736	-1	recombinase family protein
1551711	1651998	100287	OXZ38113.1	B9N50_07785	1575991	1576698	-1	hypothetical protein
1551711	1651998	100287	OXZ38053.1	B9N50_07790	1577036	1577653	-1	hypothetical protein
1551711	1651998	100287		B9N50_07795	1577792	1578732	-1	recombinase family protein
1551711	1651998	100287	OXZ38114.1	B9N50_07800	1579119	1579574	-1	hypothetical protein
1551711	1651998	100287		B9N50_07805	1579830	1580329	-1	hypothetical protein
1551711	1651998	100287	OXZ38054.1	B9N50_07810	1580757	1582511	-1	recombinase family protein
1551711	1651998	100287	OXZ38055.1	B9N50_07815	1583242	1583652	-1	RNA polymerase subunit sigma
1551711	1651998	100287	OXZ38115.1	B9N50_07820	1583922	1584329	-1	hypothetical protein
1551711	1651998	100287	OXZ38056.1	B9N50_07825	1584632	1585033	1	transcriptional regulator
1551711	1651998	100287	OXZ38057.1	B9N50_07830	1585042	1585590	1	toxin
1551711	1651998	100287	OXZ38058.1	B9N50_07835	1585599	1586081	1	hypothetical protein
1551711	1651998	100287	OXZ38059.1	B9N50_07840	1586074	1587147	1	cell filamentation protein Fic
1551711	1651998	100287	OXZ38060.1	B9N50_07845	1587318	1587674	1	plasmid mobilization relaxosome protein MobC
1551711	1651998	100287	OXZ38061.1	B9N50_07850	1587676	1589007	1	endonuclease
1551711	1651998	100287		B9N50_07855	1589224	1590455	-1	IS256 family transposase
1551711	1651998	100287		B9N50_07860	1590580	1591127	-1	hypothetical protein
1551711	1651998	100287	OXZ38062.1	B9N50_07865	1591257	1591448	-1	hypothetical protein
1551711	1651998	100287	OXZ38063.1	B9N50_07870	1591634	1592872	-1	hypothetical protein
1551711	1651998	100287	OXZ38064.1	B9N50_07875	1592885	1593934	-1	hypothetical protein
1551711	1651998	100287	OXZ38065.1	B9N50_07880	1593943	1594962	-1	hypothetical protein
1551711	1651998	100287	OXZ38066.1	B9N50_07885	1594946	1596610	-1	hypothetical protein
1551711	1651998	100287	OXZ38067.1	B9N50_07890	1596603	1597754	-1	hypothetical protein
1551711	1651998	100287	OXZ38068.1	B9N50_07895	1597757	1600087	-1	ABC transporter permease
1551711	1651998	100287	OXZ38069.1	B9N50_07900	1600087	1600767	-1	ABC transporter ATP-binding protein
1551711	1651998	100287	OXZ38070.1	B9N50_07905	1601124	1601330	1	hypothetical protein
1551711	1651998	100287	OXZ38071.1	B9N50_07910	1601736	1602029	-1	hypothetical protein
1551711	1651998	100287	OXZ38072.1	B9N50_07915	1602246	1602566	-1	hypothetical protein
1551711	1651998	100287	OXZ38073.1	B9N50_07920	1602697	1603128	-1	hypothetical protein
1551711	1651998	100287	OXZ38074.1	B9N50_07925	1603141	1603407	-1	hypothetical protein
1551711	1651998	100287	OXZ38075.1	B9N50_07930	1603401	1603970	-1	hypothetical protein
1551711	1651998	100287		B9N50_07935	1604957	1605466	1	transposase
1551711	1651998	100287	OXZ38076.1	B9N50_07940	1605602	1605955	1	hypothetical protein
1551711	1651998	100287	OXZ38077.1	B9N50_07945	1606029	1606667	-1	hypothetical protein
1551711	1651998	100287	OXZ38078.1	B9N50_07950	1606664	1607827	-1	hypothetical protein
1551711	1651998	100287	OXZ38079.1	B9N50_07955	1607838	1608710	-1	hypothetical protein
1551711	1651998	100287	OXZ38080.1	B9N50_07960	1608697	1610373	-1	hypothetical protein
1551711	1651998	100287	OXZ38081.1	B9N50_07965	1610357	1611238	-1	hypothetical protein
1551711	1651998	100287	OXZ38082.1	B9N50_07970	1611328	1611522	-1	hypothetical protein
1551711	1651998	100287	OXZ38083.1	B9N50_07975	1611561	1612568	-1	hypothetical protein
1551711	1651998	100287	OXZ38084.1	B9N50_07980	1612572	1613831	-1	hypothetical protein
1551711	1651998	100287	OXZ38085.1	B9N50_07985	1614036	1615673	-1	ABC transporter permease

1551711	1651998	100287	OXZ38086.1	B9N50_07990	1615666	1615932	-1	CXXX repeat peptide modification system protein
1551711	1651998	100287	OXZ38087.1	B9N50_07995	1615934	1618120	-1	radical SAM protein
1551711	1651998	100287	OXZ38088.1	B9N50_08000	1618402	1619007	-1	DNA-binding response regulator
1551711	1651998	100287	OXZ38089.1	B9N50_08005	1618979	1620526	-1	hypothetical protein
1551711	1651998	100287	OXZ38090.1	B9N50_08010	1620826	1621482	-1	DNA-binding protein
1551711	1651998	100287	OXZ38091.1	B9N50_08015	1621526	1629169	-1	DNA helicase
1551711	1651998	100287	OXZ38092.1	B9N50_08020	1629156	1630121	-1	DNA (cytosine-5-)-methyltransferase
1551711	1651998	100287	OXZ38093.1	B9N50_08025	1630114	1631835	-1	DNA topoisomerase III
1551711	1651998	100287	OXZ38094.1	B9N50_08030	1631928	1632911	-1	hypothetical protein
1551711	1651998	100287	OXZ38095.1	B9N50_08035	1632904	1633515	-1	hypothetical protein
1551711	1651998	100287	OXZ38096.1	B9N50_08040	1633589	1634446	-1	bacteriocin
1551711	1651998	100287	OXZ38097.1	B9N50_08045	1634461	1635746	-1	copper amine oxidase
1551711	1651998	100287	OXZ38297.1	B9N50_05400	1636747	1637171	-1	copper amine oxidase
1551711	1651998	100287	OXZ38298.1	B9N50_05405	1637158	1637397	-1	hypothetical protein
1551711	1651998	100287	OXZ38299.1	B9N50_05410	1637412	1639991	-1	hypothetical protein
1551711	1651998	100287	OXZ38300.1	B9N50_05415	1639999	1642422	-1	conjugal transfer protein TraE
1551711	1651998	100287	OXZ38301.1	B9N50_05420	1642313	1642723	-1	conjugal transfer protein
1551711	1651998	100287	OXZ38302.1	B9N50_05425	1642725	1643015	-1	hypothetical protein
1551711	1651998	100287	OXZ38303.1	B9N50_05430	1643025	1643888	-1	hypothetical protein
1551711	1651998	100287	OXZ38304.1	B9N50_05435	1643899	1644114	-1	conjugal transfer protein
1551711	1651998	100287	OXZ38305.1	B9N50_05440	1644116	1644427	-1	single-stranded DNA-binding protein
1551711	1651998	100287	OXZ38306.1	B9N50_05445	1644616	1645071	-1	hypothetical protein
1551711	1651998	100287	OXZ38307.1	B9N50_05450	1645068	1645676	-1	bacteriocin ABC transporter ATP-binding protein
1551711	1651998	100287	OXZ38308.1	B9N50_05455	1645673	1647649	-1	hypothetical protein
1551711	1651998	100287	OXZ38309.1	B9N50_05460	1647814	1648152	-1	hypothetical protein
1551711	1651998	100287		B9N50_05465	1648403	1648661	-1	conjugal transfer protein TraG
1551711	1651998	100287	OXZ38310.1	B9N50_05470	1648699	1648974	-1	hypothetical protein
1551711	1651998	100287	OXZ38311.1	B9N50_05475	1648993	1650789	-1	conjugal transfer protein TraG
1551711	1651998	100287	OXZ38312.1	B9N50_05480	1650782	1651516	-1	phage antirepressor Ant
1551711	1651998	100287	OXZ38313.1	B9N50_05485	1651513	1651998	-1	conjugal transfer protein
1551711	1651998	100287	OXZ38314.1	B9N50_05490	1651991	1652818	-1	nucleoside triphosphate hydrolase
1591257	1596610	5353	OXZ38062.1	B9N50_07865	1591257	1591448	-1	hypothetical protein
1591257	1596610	5353	OXZ38063.1	B9N50_07870	1591634	1592872	-1	hypothetical protein
1591257	1596610	5353	OXZ38064.1	B9N50_07875	1592885	1593934	-1	hypothetical protein
1591257	1596610	5353	OXZ38065.1	B9N50_07880	1593943	1594962	-1	hypothetical protein
1591257	1596610	5353	OXZ38066.1	B9N50_07885	1594946	1596610	-1	hypothetical protein
1591257	1596610	5353	OXZ38067.1	B9N50_07890	1596603	1597754	-1	hypothetical protein
1611561	1619007	7446	OXZ38083.1	B9N50_07975	1611561	1612568	-1	hypothetical protein
1611561	1619007	7446	OXZ38084.1	B9N50_07980	1612572	1613831	-1	hypothetical protein
1611561	1619007	7446	OXZ38085.1	B9N50_07985	1614036	1615673	-1	ABC transporter permease
1611561	1619007	7446	OXZ38086.1	B9N50_07990	1615666	1615932	-1	CXXX repeat peptide modification system protein
1611561	1619007	7446	OXZ38087.1	B9N50_07995	1615934	1618120	-1	radical SAM protein
1611561	1619007	7446	OXZ38088.1	B9N50_08000	1618402	1619007	-1	DNA-binding response regulator
1611561	1619007	7446	OXZ38089.1	B9N50_08005	1618979	1620526	-1	hypothetical protein
1644116	1651998	7882	OXZ38305.1	B9N50_05440	1644116	1644427	-1	single-stranded DNA-binding protein
1644116	1651998	7882	OXZ38306.1	B9N50_05445	1644616	1645071	-1	hypothetical protein
1644116	1651998	7882	OXZ38307.1	B9N50_05450	1645068	1645676	-1	bacteriocin ABC transporter ATP-binding protein
1644116	1651998	7882	OXZ38308.1	B9N50_05455	1645673	1647649	-1	hypothetical protein
1644116	1651998	7882	OXZ38309.1	B9N50_05460	1647814	1648152	-1	hypothetical protein
1644116	1651998	7882		B9N50_05465	1648403	1648661	-1	conjugal transfer protein TraG
1644116	1651998	7882	OXZ38310.1	B9N50_05470	1648699	1648974	-1	hypothetical protein
1644116	1651998	7882	OXZ38311.1	B9N50_05475	1648993	1650789	-1	conjugal transfer protein TraG
1644116	1651998	7882	OXZ38312.1	B9N50_05480	1650782	1651516	-1	phage antirepressor Ant
1644116	1651998	7882	OXZ38313.1	B9N50_05485	1651513	1651998	-1	conjugal transfer protein
1644116	1651998	7882	OXZ38314.1	B9N50_05490	1651991	1652818	-1	nucleoside triphosphate hydrolase
1771690	1776504	4814	OXZ37464.1	B9N50_08530	1770150	1771697	-1	hypothetical protein
1771690	1776504	4814	OXZ37465.1	B9N50_08535	1771690	1772916	-1	hypothetical protein
1771690	1776504	4814	OXZ37466.1	B9N50_08540	1772947	1774119	-1	hypothetical protein
1771690	1776504	4814	OXZ37467.1	B9N50_08545	1774194	1775369	-1	hypothetical protein
1771690	1776504	4814	OXZ37468.1	B9N50_08550	1775362	1776504	-1	hypothetical protein

Table S3E: Predicted genomic islands in the genome of *"F. nericia"* 12T272

Shown are genes of genomic islands predicted by IslandViewer4

Island start	Island end	Length	Gene name	Locus	Gene start	Gene end	Strand	Product
34527	44047	9520	OXZ36018.1	B9N53_00160	34527	35396	1	hypothetical protein
34527	44047	9520	OXZ36019.1	B9N53_00165	35383	37371	1	exopolyphosphatase
34527	44047	9520	OXZ36020.1	B9N53_00170	37352	37798	1	50S ribosomal protein L9
34527	44047	9520	OXZ36021.1	B9N53_00175	37832	39169	1	replicative DNA helicase
34527	44047	9520	OXZ36022.1	B9N53_00180	39166	39729	1	GNAT family N-acetyltransferase
34527	44047	9520	OXZ36023.1	B9N53_00185	39734	40492	1	DNA replication protein
34527	44047	9520	OXZ36024.1	B9N53_00190	40495	41472	1	DNA replication protein DnaC
34527	44047	9520	OXZ36025.1	B9N53_00195	41469	42113	1	NAD(P)H-hydrate epimerase
34527	44047	9520	OXZ36026.1	B9N53_00200	42835	43377	1	ECF transporter S component
34527	44047	9520	OXZ36027.1	B9N53_00205	43361	44047	1	radical SAM protein
604530	616700	12170	OXZ35396.1	B9N53_03100	604530	605510	-1	recombinase XerC
604530	616700	12170	OXZ35397.1	B9N53_03105	605613	606788	-1	aspartate aminotransferase
604530	616700	12170	OXZ35398.1	B9N53_03110	606769	607596	-1	metal-dependent phosphoesterase
604530	616700	12170	OXZ35399.1	B9N53_03115	607665	609206	-1	ribonuclease Y
604530	616700	12170	OXZ35400.1	B9N53_03120	609325	609987	-1	chromosome partitioning protein ParA
604530	616700	12170	OXZ35401.1	B9N53_03125	609974	610213	-1	hypothetical protein
604530	616700	12170	OXZ35402.1	B9N53_03130	610213	611796	-1	exonuclease SbcC
604530	616700	12170	OXZ35420.1	B9N53_03135	611796	612746	-1	phosphoesterase
604530	616700	12170	OXZ35403.1	B9N53_03140	612777	613868	-1	recombinase RecA
604530	616700	12170	OXZ35404.1	B9N53_03145	613937	614473	-1	CDP-diacylglycerol--glycerol-3-phosphate 3-phosphatidyltransferase
604530	616700	12170	OXZ35405.1	B9N53_03150	614475	616700	-1	cell division protein FtsK
1277699	1282020	4321		B9N53_06220	1277699	1278187	-1	terminase
1277699	1282020	4321		B9N53_06225	1278379	1279523	1	IS110 family transposase
1277699	1282020	4321	OXZ34114.1	B9N53_06230	1280284	1282020	-1	multidrug ABC transporter ATP-binding protein
1294955	1300767	5812	OXZ34125.1	B9N53_06305	1293840	1294958	-1	alpha/beta hydrolase
1294955	1300767	5812	OXZ34126.1	B9N53_06310	1294955	1295212	-1	AbrB family transcriptional regulator
1294955	1300767	5812	OXZ34127.1	B9N53_06315	1295862	1296230	-1	EXLDI protein
1294955	1300767	5812		B9N53_06320	1296417	1296826	-1	recombinase family protein
1294955	1300767	5812	OXZ34201.1	B9N53_06325	1297080	1297787	-1	hypothetical protein
1294955	1300767	5812	OXZ34128.1	B9N53_06330	1298153	1298761	-1	hypothetical protein
1294955	1300767	5812		B9N53_06335	1298777	1299649	-1	recombinase family protein
1294955	1300767	5812	OXZ34129.1	B9N53_06340	1300150	1300767	-1	hypothetical protein
1359973	1364035	4062	OXZ34202.1	B9N53_06615	1359973	1360320	1	toxin MazF
1359973	1364035	4062	OXZ34181.1	B9N53_06620	1360974	1361549	-1	cell filamentation protein Fic
1359973	1364035	4062	OXZ34182.1	B9N53_06625	1362004	1362738	-1	hydrolase
1359973	1364035	4062	OXZ34183.1	B9N53_06630	1362738	1363241	-1	acetyltransferase
1359973	1364035	4062	OXZ34184.1	B9N53_06635	1363394	1364035	1	hemolysin III
1496002	1504337	8335	OXZ33200.1	B9N53_07260	1496002	1496184	-1	hypothetical protein
1496002	1504337	8335	OXZ33201.1	B9N53_07265	1496240	1497262	-1	hypothetical protein
1496002	1504337	8335	OXZ33202.1	B9N53_07270	1497300	1498811	-1	hypothetical protein
1496002	1504337	8335	OXZ33203.1	B9N53_07275	1498825	1500027	-1	hypothetical protein
1496002	1504337	8335	OXZ33204.1	B9N53_07280	1500062	1501129	-1	glycosyl transferase family 2
1496002	1504337	8335	OXZ33205.1	B9N53_07285	1501122	1502285	-1	capsule biosynthesis protein
1496002	1504337	8335	OXZ33206.1	B9N53_07290	1502299	1503345	-1	hypothetical protein
1496002	1504337	8335	OXZ33207.1	B9N53_07295	1503342	1504337	-1	hypothetical protein
1736754	1830377	93623	OXZ32959.1	B9N53_08375	1736754	1737218	-1	hypothetical protein
1736754	1830377	93623	OXZ32960.1	B9N53_08380	1737303	1738106	-1	DNA-binding protein
1736754	1830377	93623	OXZ32961.1	B9N53_08385	1738296	1739816	-1	conjugal transfer protein
1736754	1830377	93623	OXZ32962.1	B9N53_08390	1739809	1741551	-1	recombinase
1736754	1830377	93623	OXZ32963.1	B9N53_08395	1741544	1743262	-1	recombinase
1736754	1830377	93623	OXZ32964.1	B9N53_08400	1743361	1743528	-1	aminoacyl-tRNA hydrolase
1736754	1830377	93623	OXZ32965.1	B9N53_08405	1743945	1744355	-1	RNA polymerase subunit sigma
1736754	1830377	93623	OXZ32966.1	B9N53_08410	1744638	1746374	-1	multidrug ABC transporter ATP-binding protein
1736754	1830377	93623	OXZ32967.1	B9N53_08415	1746367	1748088	-1	ABC transporter ATP-binding protein
1736754	1830377	93623	OXZ32968.1	B9N53_08420	1748126	1749478	-1	MATE family efflux transporter
1736754	1830377	93623	OXZ32969.1	B9N53_08425	1749491	1750939	-1	ABC transporter
1736754	1830377	93623	OXZ32970.1	B9N53_08430	1750936	1751616	-1	ABC transporter permease
1736754	1830377	93623	OXZ32971.1	B9N53_08435	1751616	1752194	-1	hypothetical protein
1736754	1830377	93623	OXZ32972.1	B9N53_08440	1752246	1752812	-1	TetR family transcriptional regulator
1736754	1830377	93623	OXZ32973.1	B9N53_08445	1752898	1753650	-1	hypothetical protein
1736754	1830377	93623	OXZ32974.1	B9N53_08450	1753651	1754706	-1	saccharopine dehydrogenase
1736754	1830377	93623	OXZ32975.1	B9N53_08455	1754796	1760315	-1	non-ribosomal peptide synthetase
1736754	1830377	93623	OXZ32976.1	B9N53_08460	1760336	1762276	-1	condensation protein
1736754	1830377	93623	OXZ32977.1	B9N53_08465	1762289	1763875	-1	2,3-dihydroxybenzoate-AMP ligase
1736754	1830377	93623	OXZ32978.1	B9N53_08470	1763875	1764567	-1	4-phosphopantetheinyl transferase
1736754	1830377	93623	OXZ32979.1	B9N53_08475	1764557	1765309	-1	thioesterase
1736754	1830377	93623	OXZ32980.1	B9N53_08480	1765470	1765841	-1	transcriptional regulator
1736754	1830377	93623	OXZ32981.1	B9N53_08485	1766366	1766734	1	plasmid mobilization relaxosome protein MobC
1736754	1830377	93623	OXZ32982.1	B9N53_08490	1766734	1768104	1	endonuclease
1736754	1830377	93623		B9N53_08495	1768445	1769660	-1	IS256 family transposase
1736754	1830377	93623	OXZ32983.1	B9N53_08500	1769853	1770317	-1	hypothetical protein
1736754	1830377	93623	OXZ32984.1	B9N53_08505	1770441	1772564	-1	bacteriocin/lantibiotic ABC transporter ATP-binding protein
1736754	1830377	93623	OXZ32985.1	B9N53_08510	1772654	1775869	-1	type 2 lantipeptide synthetase LanM
1736754	1830377	93623	OXZ32986.1	B9N53_08515	1775957	1776184	-1	type 2 lantibiotic, mersacidin/lichenicidin family protein
1736754	1830377	93623	OXZ32987.1	B9N53_08520	1776259	1776981	-1	ABC transporter permease
1736754	1830377	93623	OXZ32988.1	B9N53_08525	1777001	1777735	-1	hypothetical protein
1736754	1830377	93623	OXZ32989.1	B9N53_08530	1777728	1778645	-1	ABC transporter ATP-binding protein
1736754	1830377	93623		B9N53_08535	1778911	1779289	-1	hypothetical protein

1736754	1830377	93623	OXZ32990.1	B9N53_08540	1779302	1779568	-1	hypothetical protein
1736754	1830377	93623	OXZ32991.1	B9N53_08545	1779562	1780131	-1	hypothetical protein
1736754	1830377	93623		B9N53_08550	1780470	1780774	1	relaxase
1736754	1830377	93623	OXZ32992.1	B9N53_08555	1780793	1781224	-1	hypothetical protein
1736754	1830377	93623		B9N53_08560	1781178	1782119	1	endonuclease
1736754	1830377	93623	OXZ32993.1	B9N53_08565	1782137	1782883	-1	ABC transporter permease
1736754	1830377	93623	OXZ32994.1	B9N53_08570	1782898	1783590	-1	ABC transporter permease
1736754	1830377	93623	OXZ32995.1	B9N53_08575	1783592	1784506	-1	bacitracin ABC transporter ATP-binding protein
1736754	1830377	93623	OXZ32996.1	B9N53_08580	1784521	1785678	-1	hypothetical protein
1736754	1830377	93623	OXZ32997.1	B9N53_08585	1785682	1786989	-1	hypothetical protein
1736754	1830377	93623	OXZ32998.1	B9N53_08590	1787575	1789212	-1	ABC transporter permease
1736754	1830377	93623	OXZ32999.1	B9N53_08595	1789205	1789471	-1	CXXX repeat peptide modification system protein
1736754	1830377	93623	OXZ33000.1	B9N53_08600	1789473	1791662	-1	radical SAM protein
1736754	1830377	93623	OXZ33001.1	B9N53_08605	1791649	1791831	-1	hypothetical protein
1736754	1830377	93623	OXZ33002.1	B9N53_08610	1791961	1792566	-1	DNA-binding response regulator
1736754	1830377	93623	OXZ33003.1	B9N53_08615	1792538	1794085	-1	hypothetical protein
1736754	1830377	93623	OXZ33004.1	B9N53_08620	1794400	1795494	-1	single-stranded DNA-binding protein
1736754	1830377	93623	OXZ33005.1	B9N53_08625	1795527	1805945	-1	conjugal transfer protein
1736754	1830377	93623	OXZ33006.1	B9N53_08630	1806140	1806466	-1	terminase large subunit
1736754	1830377	93623	OXZ33007.1	B9N53_08635	1806808	1808514	-1	DNA topoisomerase III
1736754	1830377	93623	OXZ33008.1	B9N53_08640	1808931	1809809	-1	bacteriocin
1736754	1830377	93623	OXZ33009.1	B9N53_08645	1809825	1812134	-1	copper amine oxidase
1736754	1830377	93623	OXZ33010.1	B9N53_08650	1812131	1812388	-1	hypothetical protein
1736754	1830377	93623	OXZ33011.1	B9N53_08655	1812408	1815371	-1	hypothetical protein
1736754	1830377	93623	OXZ33012.1	B9N53_08660	1815381	1816346	-1	site-specific DNA-methyltransferase
1736754	1830377	93623	OXZ33013.1	B9N53_08665	1816318	1818813	-1	conjugal transfer protein TraE
1736754	1830377	93623	OXZ33014.1	B9N53_08670	1818810	1819148	-1	conjugal transfer protein
1736754	1830377	93623	OXZ33015.1	B9N53_08675	1819148	1819405	-1	hypothetical protein
1736754	1830377	93623	OXZ33016.1	B9N53_08680	1819423	1820286	-1	hypothetical protein
1736754	1830377	93623	OXZ33017.1	B9N53_08685	1820288	1820503	-1	conjugal transfer protein
1736754	1830377	93623	OXZ33018.1	B9N53_08690	1820504	1820827	-1	single-stranded DNA-binding protein
1736754	1830377	93623	OXZ33019.1	B9N53_08695	1821017	1821880	-1	CAAX protease
1736754	1830377	93623	OXZ33020.1	B9N53_08700	1822032	1823834	-1	conjugal transfer protein TraG
1736754	1830377	93623	OXZ33021.1	B9N53_08705	1823879	1824085	-1	hypothetical protein
1736754	1830377	93623	OXZ33022.1	B9N53_08710	1824282	1824773	-1	DUF3801 domain-containing protein
1736754	1830377	93623	OXZ33083.1	B9N53_08715	1824788	1825645	-1	DNA replication protein
1736754	1830377	93623	OXZ33023.1	B9N53_08720	1825660	1826637	-1	replication initiator RepA
1736754	1830377	93623	OXZ33024.1	B9N53_08725	1826724	1827014	-1	hypothetical protein
1736754	1830377	93623		B9N53_08730	1827163	1828239	-1	ABC transporter ATP-binding protein
1736754	1830377	93623	OXZ33025.1	B9N53_08735	1828498	1829421	-1	RNA-binding protein
1736754	1830377	93623	OXZ33026.1	B9N53_08740	1829418	1830149	-1	insertase
1736754	1830377	93623	OXZ33027.1	B9N53_08745	1830165	1830377	-1	membrane protein insertion efficiency factor YidD
1736754	1830377	93623	OXZ33028.1	B9N53_08750	1830365	1830706	-1	ribonuclease P protein component
1736754	1741551	4797	OXZ32959.1	B9N53_08375	1736754	1737218	-1	hypothetical protein
1736754	1741551	4797	OXZ32960.1	B9N53_08380	1737303	1738106	-1	DNA-binding protein
1736754	1741551	4797	OXZ32961.1	B9N53_08385	1738296	1739816	-1	conjugal transfer protein
1736754	1741551	4797	OXZ32962.1	B9N53_08390	1739809	1741551	-1	recombinase
1736754	1741551	4797	OXZ32963.1	B9N53_08395	1741544	1743262	-1	recombinase
1743361	1754706	11345	OXZ32964.1	B9N53_08400	1743361	1743528	-1	aminoacyl-tRNA hydrolase
1743361	1754706	11345	OXZ32965.1	B9N53_08405	1743945	1744355	-1	RNA polymerase subunit sigma
1743361	1754706	11345	OXZ32966.1	B9N53_08410	1744638	1746374	-1	multidrug ABC transporter ATP-binding protein
1743361	1754706	11345	OXZ32967.1	B9N53_08415	1746367	1748088	-1	ABC transporter ATP-binding protein
1743361	1754706	11345	OXZ32968.1	B9N53_08420	1748126	1749478	-1	MATE family efflux transporter
1743361	1754706	11345	OXZ32969.1	B9N53_08425	1749491	1750939	-1	ABC transporter
1743361	1754706	11345	OXZ32970.1	B9N53_08430	1750936	1751616	-1	ABC transporter permease
1743361	1754706	11345	OXZ32971.1	B9N53_08435	1751616	1752194	-1	hypothetical protein
1743361	1754706	11345	OXZ32972.1	B9N53_08440	1752246	1752812	-1	TetR family transcriptional regulator
1743361	1754706	11345	OXZ32973.1	B9N53_08445	1752898	1753650	-1	hypothetical protein
1743361	1754706	11345	OXZ32974.1	B9N53_08450	1753651	1754706	-1	saccharopine dehydrogenase
1760336	1776981	16645	OXZ32976.1	B9N53_08460	1760336	1762276	-1	condensation protein
1760336	1776981	16645	OXZ32977.1	B9N53_08465	1762289	1763875	-1	2,3-dihydroxybenzoate-AMP ligase
1760336	1776981	16645	OXZ32978.1	B9N53_08470	1763875	1764567	-1	4-phosphopantetheinyl transferase
1760336	1776981	16645	OXZ32979.1	B9N53_08475	1764557	1765309	-1	thioesterase
1760336	1776981	16645	OXZ32980.1	B9N53_08480	1765470	1765841	-1	transcriptional regulator
1760336	1776981	16645	OXZ32981.1	B9N53_08485	1766366	1766734	1	plasmid mobilization relaxosome protein MobC
1760336	1776981	16645	OXZ32982.1	B9N53_08490	1766734	1768104	1	endonuclease
1760336	1776981	16645		B9N53_08495	1768445	1769660	-1	IS256 family transposase
1760336	1776981	16645	OXZ32983.1	B9N53_08500	1769853	1770317	-1	hypothetical protein
1760336	1776981	16645	OXZ32984.1	B9N53_08505	1770441	1772564	-1	bacteriocin/lantibiotic ABC transporter ATP-binding protein
1760336	1776981	16645	OXZ32985.1	B9N53_08510	1772654	1775869	-1	type 2 lantipeptide synthetase LanM
1760336	1776981	16645	OXZ32986.1	B9N53_08515	1775957	1776184	-1	type 2 lantibiotic, mersacidin/lichenicidin family protein
1760336	1776981	16645	OXZ32987.1	B9N53_08520	1776259	1776981	-1	ABC transporter permease
1778911	1816346	37435		B9N53_08535	1778911	1779289	-1	hypothetical protein
1778911	1816346	37435	OXZ32990.1	B9N53_08540	1779302	1779568	-1	hypothetical protein
1778911	1816346	37435	OXZ32991.1	B9N53_08545	1779562	1780131	-1	hypothetical protein
1778911	1816346	37435		B9N53_08550	1780470	1780774	1	relaxase
1778911	1816346	37435	OXZ32992.1	B9N53_08555	1780793	1781224	-1	hypothetical protein
1778911	1816346	37435		B9N53_08560	1781178	1782119	1	endonuclease
1778911	1816346	37435	OXZ32993.1	B9N53_08565	1782137	1782883	-1	ABC transporter permease
1778911	1816346	37435	OXZ32994.1	B9N53_08570	1782898	1783590	-1	ABC transporter permease

1778911	1816346	37435	OXZ32995.1	B9N53_08575	1783592	1784506	-1	bacitracin ABC transporter ATP-binding protein
1778911	1816346	37435	OXZ32996.1	B9N53_08580	1784521	1785678	-1	hypothetical protein
1778911	1816346	37435	OXZ32997.1	B9N53_08585	1785682	1786989	-1	hypothetical protein
1778911	1816346	37435	OXZ32998.1	B9N53_08590	1787575	1789212	-1	ABC transporter permease
1778911	1816346	37435	OXZ32999.1	B9N53_08595	1789205	1789471	-1	CXXX repeat peptide modification system protein
1778911	1816346	37435	OXZ33000.1	B9N53_08600	1789473	1791662	-1	radical SAM protein
1778911	1816346	37435	OXZ33001.1	B9N53_08605	1791649	1791831	-1	hypothetical protein
1778911	1816346	37435	OXZ33002.1	B9N53_08610	1791961	1792566	-1	DNA-binding response regulator
1778911	1816346	37435	OXZ33003.1	B9N53_08615	1792538	1794085	-1	hypothetical protein
1778911	1816346	37435	OXZ33004.1	B9N53_08620	1794400	1795494	-1	single-stranded DNA-binding protein
1778911	1816346	37435	OXZ33005.1	B9N53_08625	1795527	1805945	-1	conjugal transfer protein
1778911	1816346	37435	OXZ33006.1	B9N53_08630	1806140	1806466	-1	terminase large subunit
1778911	1816346	37435	OXZ33007.1	B9N53_08635	1806808	1808514	-1	DNA topoisomerase III
1778911	1816346	37435	OXZ33008.1	B9N53_08640	1808931	1809809	-1	bacteriocin
1778911	1816346	37435	OXZ33009.1	B9N53_08645	1809825	1812134	-1	copper amine oxidase
1778911	1816346	37435	OXZ33010.1	B9N53_08650	1812131	1812388	-1	hypothetical protein
1778911	1816346	37435	OXZ33011.1	B9N53_08655	1812408	1815371	-1	hypothetical protein
1778911	1816346	37435	OXZ33012.1	B9N53_08660	1815381	1816346	-1	site-specific DNA-methyltransferase
1778911	1816346	37435	OXZ33013.1	B9N53_08665	1816318	1818813	-1	conjugal transfer protein TraE
1818810	1827014	8204	OXZ33013.1	B9N53_08665	1816318	1818813	-1	conjugal transfer protein TraE
1818810	1827014	8204	OXZ33014.1	B9N53_08670	1818810	1819148	-1	conjugal transfer protein
1818810	1827014	8204	OXZ33015.1	B9N53_08675	1819148	1819405	-1	hypothetical protein
1818810	1827014	8204	OXZ33016.1	B9N53_08680	1819423	1820286	-1	hypothetical protein
1818810	1827014	8204	OXZ33017.1	B9N53_08685	1820288	1820503	-1	conjugal transfer protein
1818810	1827014	8204	OXZ33018.1	B9N53_08690	1820504	1820827	-1	single-stranded DNA-binding protein
1818810	1827014	8204	OXZ33019.1	B9N53_08695	1821017	1821880	-1	CAAX protease
1818810	1827014	8204	OXZ33020.1	B9N53_08700	1822032	1823834	-1	conjugal transfer protein TraG
1818810	1827014	8204	OXZ33021.1	B9N53_08705	1823879	1824085	-1	hypothetical protein
1818810	1827014	8204	OXZ33022.1	B9N53_08710	1824282	1824773	-1	DUF3801 domain-containing protein
1818810	1827014	8204	OXZ33083.1	B9N53_08715	1824788	1825645	-1	DNA replication protein
1818810	1827014	8204	OXZ33023.1	B9N53_08720	1825660	1826637	-1	replication initiator RepA
1818810	1827014	8204	OXZ33024.1	B9N53_08725	1826724	1827014	-1	hypothetical protein
1885694	1892257	6563	OXZ32597.1	B9N53_09040	1885694	1886158	1	IS200/IS605 family transposase
1885694	1892257	6563	OXZ32598.1	B9N53_09035	1891243	1892257	1	elongation factor Tu

Table S3F: Predicted genomic islands in the genome of *"F. nericia"* 12T273

Shown are genes of genomic islands predicted by IslandViewer4

Island start	Island end	Length	Gene name	Locus	Gene start	Gene end	Strand	Product
97479	122315	24836	OXZ35127.1	B9N55_00530	97479	97877	-1	hypothetical protein
97479	122315	24836	OXZ35128.1	B9N55_00535	97946	98398	-1	small-multidrug export protein
97479	122315	24836	OXZ35129.1	B9N55_00540	98407	99057	-1	phosphatase
97479	122315	24836	OXZ35130.1	B9N55_00545	99219	100451	1	aminopeptidase
97479	122315	24836	OXZ35131.1	B9N55_00550	100451	102247	1	oligoendopeptidase F
97479	122315	24836	OXZ35132.1	B9N55_00555	102300	103184	1	antibiotic ABC transporter ATP-binding protein
97479	122315	24836	OXZ35133.1	B9N55_00560	103177	103929	1	antibiotic ABC transporter permease
97479	122315	24836	OXZ35134.1	B9N55_00565	103919	104590	1	antibiotic ABC transporter permease
97479	122315	24836	OXZ35135.1	B9N55_00570	104590	105891	1	MFS transporter
97479	122315	24836	OXZ35136.1	B9N55_00575	105948	106307	1	superoxide reductase
97479	122315	24836	OXZ35137.1	B9N55_00580	106460	107821	1	hypothetical protein
97479	122315	24836	OXZ35138.1	B9N55_00585	107859	108398	1	transposase
97479	122315	24836	OXZ35172.1	B9N55_00590	108593	109255	1	transposase
97479	122315	24836	OXZ35139.1	B9N55_00595	109420	109656	1	hypothetical protein
97479	122315	24836	OXZ35140.1	B9N55_00600	109674	110216	1	LemA family protein
97479	122315	24836	OXZ35141.1	B9N55_00605	110340	111248	1	coproporphyrinogen III oxidase
97479	122315	24836	OXZ35142.1	B9N55_00610	111248	111976	1	biotin--[acetyl-CoA-carboxylase] ligase
97479	122315	24836	OXZ35143.1	B9N55_00615	111989	112216	-1	hypothetical protein
97479	122315	24836	OXZ35144.1	B9N55_00620	112308	112709	1	transcriptional regulator
97479	122315	24836	OXZ35145.1	B9N55_00625	112747	112962	1	heavy metal transporter
97479	122315	24836	OXZ35146.1	B9N55_00630	112962	114773	1	cadmium-translocating P-type ATPase
97479	122315	24836	OXZ35147.1	B9N55_00635	114820	115131	1	hypothetical protein
97479	122315	24836	OXZ35148.1	B9N55_00640	115179	115445	-1	cytotoxin
97479	122315	24836	OXZ35149.1	B9N55_00645	115445	115702	-1	AbrB family transcriptional regulator
97479	122315	24836	OXZ35150.1	B9N55_00650	115806	116378	-1	riboflavin transporter
97479	122315	24836	OXZ35151.1	B9N55_00655	116449	117591	-1	2-iminoacetate synthase ThiH
97479	122315	24836	OXZ35152.1	B9N55_00660	117599	118363	-1	thiazole synthase
97479	122315	24836	OXZ35153.1	B9N55_00665	118353	118949	-1	thiamine biosynthesis protein ThiF
97479	122315	24836	OXZ35154.1	B9N55_00670	118951	119148	-1	thiamine biosynthesis protein ThiS
97479	122315	24836	OXZ35155.1	B9N55_00675	119369	119980	1	glutathione S-transferase
97479	122315	24836	OXZ35156.1	B9N55_00680	120077	120778	1	SAM-dependent methyltransferase
97479	122315	24836	OXZ35157.1	B9N55_00685	120795	122315	1	magnesium chelatase
138212	146541	8329	OXZ34581.1	B9N55_00760	138212	138409	1	transcriptional regulator
138212	146541	8329	OXZ34582.1	B9N55_00765	138461	139141	1	hypothetical protein
138212	146541	8329	OXZ34583.1	B9N55_00770	139134	140561	1	hypothetical protein
138212	146541	8329	OXZ34584.1	B9N55_00775	141104	142303	1	hypothetical protein
138212	146541	8329	OXZ34585.1	B9N55_00780	142319	143197	1	hypothetical protein
138212	146541	8329	OXZ34586.1	B9N55_00785	143175	143927	1	hypothetical protein
138212	146541	8329	OXZ34587.1	B9N55_00790	143928	144536	1	hypothetical protein
138212	146541	8329	OXZ34588.1	B9N55_00795	144536	144958	1	hypothetical protein
138212	146541	8329	OXZ34589.1	B9N55_00800	144961	146541	1	hypothetical protein
138461	157045	18584	OXZ34582.1	B9N55_00765	138461	139141	1	hypothetical protein
138461	157045	18584	OXZ34583.1	B9N55_00770	139134	140561	1	hypothetical protein
138461	157045	18584	OXZ34584.1	B9N55_00775	141104	142303	1	hypothetical protein
138461	157045	18584	OXZ34585.1	B9N55_00780	142319	143197	1	hypothetical protein
138461	157045	18584	OXZ34586.1	B9N55_00785	143175	143927	1	hypothetical protein
138461	157045	18584	OXZ34587.1	B9N55_00790	143928	144536	1	hypothetical protein
138461	157045	18584	OXZ34588.1	B9N55_00795	144536	144958	1	hypothetical protein
138461	157045	18584	OXZ34589.1	B9N55_00800	144961	146541	1	hypothetical protein
138461	157045	18584	OXZ34590.1	B9N55_00805	146635	147177	1	hypothetical protein
138461	157045	18584	OXZ34591.1	B9N55_00810	147277	148146	1	hypothetical protein
138461	157045	18584	OXZ34592.1	B9N55_00815	148133	150121	1	exopolyphosphatase
138461	157045	18584	OXZ34593.1	B9N55_00820	150102	150548	1	50S ribosomal protein L9
138461	157045	18584	OXZ34594.1	B9N55_00825	150582	151919	1	replicative DNA helicase
138461	157045	18584	OXZ34595.1	B9N55_00830	151916	152479	1	GNAT family N-acetyltransferase
138461	157045	18584	OXZ34596.1	B9N55_00835	152484	153242	1	DNA replication protein
138461	157045	18584	OXZ34597.1	B9N55_00840	153245	154222	1	DNA replication protein DnaC
138461	157045	18584	OXZ34598.1	B9N55_00845	154219	154863	1	NAD(P)H-hydrate epimerase
138461	157045	18584	OXZ34599.1	B9N55_00850	155059	155238	1	hypothetical protein
138461	157045	18584	OXZ34600.1	B9N55_00855	155833	156375	1	ECF transporter S component
138461	157045	18584	OXZ34601.1	B9N55_00860	156359	157045	1	radical SAM protein
253324	260267	6943	OXZ34217.1	B9N55_01390	253324	254190	1	class C sortase
253324	260267	6943	OXZ34218.1	B9N55_01395	254177	255016	1	class C sortase
253324	260267	6943	OXZ34219.1	B9N55_01400	255003	255857	1	class C sortase
253324	260267	6943	OXZ34220.1	B9N55_01405	255917	256558	-1	hemolysin III
253324	260267	6943	OXZ34221.1	B9N55_01410	256712	257215	1	acetyltransferase
253324	260267	6943	OXZ34222.1	B9N55_01415	257215	257952	1	hydrolase
253324	260267	6943		B9N55_01420	259693	260267	1	cell filamentation protein Fic
310452	354643	44191	OXZ34265.1	B9N55_01640	310452	310676	1	hypothetical protein
310452	354643	44191	OXZ34266.1	B9N55_01645	310938	311525	1	RNA polymerase subunit sigma-24
310452	354643	44191	OXZ34267.1	B9N55_01650	311611	311862	1	hypothetical protein
310452	354643	44191	OXZ34268.1	B9N55_01655	311804	312103	1	hypothetical protein
310452	354643	44191	OXZ34269.1	B9N55_01660	312103	313215	1	nuclease
310452	354643	44191	OXZ34270.1	B9N55_01665	313181	313768	1	hypothetical protein
310452	354643	44191	OXZ34271.1	B9N55_01670	313778	313978	1	hypothetical protein
310452	354643	44191	OXZ34272.1	B9N55_01675	313953	316811	1	hypothetical protein
310452	354643	44191	OXZ34273.1	B9N55_01680	316824	317024	1	hypothetical protein
310452	354643	44191	OXZ34274.1	B9N55_01685	317017	317403	1	DUF4406 domain-containing protein
310452	354643	44191	OXZ34275.1	B9N55_01690	317404	319605	1	DNA primase
310452	354643	44191	OXZ34276.1	B9N55_01695	319870	320127	1	hypothetical protein
310452	354643	44191	OXZ34277.1	B9N55_01700	320085	320366	1	nuclease
310452	354643	44191	OXZ34278.1	B9N55_01705	320367	321713	1	DEAD/DEAH box helicase

310452	354643	44191	OXZ34279.1	B9N55_01710	321806	322234	1	hypothetical protein
310452	354643	44191	OXZ34280.1	B9N55_01715	322334	322699	1	HNH endonuclease
310452	354643	44191	OXZ34281.1	B9N55_01720	322922	323389	1	terminase
310452	354643	44191	OXZ34282.1	B9N55_01725	323395	324177	1	S-adenosylmethionine synthetase
310452	354643	44191	OXZ34344.1	B9N55_01730	324177	325427	1	DNA methylase
310452	354643	44191	OXZ34283.1	B9N55_01735	325420	326016	1	endonuclease
310452	354643	44191	OXZ34284.1	B9N55_01740	326018	327460	1	DNA (cytosine-5-)-methyltransferase
310452	354643	44191	OXZ34285.1	B9N55_01745	327504	327728	1	DUF4314 domain-containing protein
310452	354643	44191	OXZ34286.1	B9N55_01750	327814	328146	1	hypothetical protein
310452	354643	44191	OXZ34287.1	B9N55_01755	328147	328377	1	hypothetical protein
310452	354643	44191	OXZ34288.1	B9N55_01760	328470	330059	1	terminase
310452	354643	44191	OXZ34289.1	B9N55_01765	330104	330523	1	conjugal transfer protein
310452	354643	44191	OXZ34290.1	B9N55_01770	330563	331837	1	phage portal protein
310452	354643	44191	OXZ34291.1	B9N55_01775	331827	332513	1	peptidase
310452	354643	44191	OXZ34292.1	B9N55_01780	332518	333708	1	phage major capsid protein
310452	354643	44191	OXZ34293.1	B9N55_01785	333719	333991	1	AraC family transcriptional regulator
310452	354643	44191	OXZ34294.1	B9N55_01790	333991	334323	1	phage head-tail adapter protein
310452	354643	44191	OXZ34295.1	B9N55_01795	334320	334700	1	hypothetical protein
310452	354643	44191	OXZ34296.1	B9N55_01800	334693	335019	1	hypothetical protein
310452	354643	44191	OXZ34297.1	B9N55_01805	335012	335191	1	hypothetical protein
310452	354643	44191	OXZ34298.1	B9N55_01810	335181	335777	1	phage tail protein
310452	354643	44191	OXZ34299.1	B9N55_01815	335780	336136	1	hypothetical protein
310452	354643	44191	OXZ34300.1	B9N55_01820	336315	336644	-1	addiction module toxin RelE
310452	354643	44191	OXZ34301.1	B9N55_01825	336641	336964	-1	prevent-host-death protein
310452	354643	44191	OXZ34302.1	B9N55_01830	337054	340119	1	phage tail tape measure protein
310452	354643	44191	OXZ34303.1	B9N55_01835	340122	340484	1	hypothetical protein
310452	354643	44191	OXZ34304.1	B9N55_01840	340484	342355	1	hypothetical protein
310452	354643	44191	OXZ34305.1	B9N55_01845	342342	343721	1	hypothetical protein
310452	354643	44191	OXZ34306.1	B9N55_01850	343723	344124	1	holin
310452	354643	44191	OXZ34307.1	B9N55_01855	344125	344934	1	lysozyme
310452	354643	44191	OXZ34308.1	B9N55_01860	345111	345701	1	TetR family transcriptional regulator
310452	354643	44191	OXZ34309.1	B9N55_01865	345706	347424	1	ABC transporter ATP-binding protein
310452	354643	44191	OXZ34310.1	B9N55_01870	347417	349153	1	multidrug ABC transporter ATP-binding protein
310452	354643	44191	OXZ34311.1	B9N55_01875	349418	349627	1	hypothetical protein
310452	354643	44191	OXZ34312.1	B9N55_01880	349687	351249	1	recombinase family protein
310452	354643	44191	OXZ34313.1	B9N55_01885	351242	351658	1	integrase
310452	354643	44191	OXZ34314.1	B9N55_01890	351651	353213	1	recombinase family protein
310452	354643	44191	OXZ34345.1	B9N55_01895	353285	353923	1	TetR family transcriptional regulator
310452	354643	44191	OXZ34315.1	B9N55_01900	354047	354643	1	hypothetical protein
310452	354643	44191	OXZ34316.1	B9N55_01905	354633	355346	1	ABC transporter permease
322334	331837	9503	OXZ34280.1	B9N55_01715	322334	322699	1	HNH endonuclease
322334	331837	9503	OXZ34281.1	B9N55_01720	322922	323389	1	terminase
322334	331837	9503	OXZ34282.1	B9N55_01725	323395	324177	1	S-adenosylmethionine synthetase
322334	331837	9503	OXZ34344.1	B9N55_01730	324177	325427	1	DNA methylase
322334	331837	9503	OXZ34283.1	B9N55_01735	325420	326016	1	endonuclease
322334	331837	9503	OXZ34284.1	B9N55_01740	326018	327460	1	DNA (cytosine-5-)-methyltransferase
322334	331837	9503	OXZ34285.1	B9N55_01745	327504	327728	1	DUF4314 domain-containing protein
322334	331837	9503	OXZ34286.1	B9N55_01750	327814	328146	1	hypothetical protein
322334	331837	9503	OXZ34287.1	B9N55_01755	328147	328377	1	hypothetical protein
322334	331837	9503	OXZ34288.1	B9N55_01760	328470	330059	1	terminase
322334	331837	9503	OXZ34289.1	B9N55_01765	330104	330523	1	conjugal transfer protein
322334	331837	9503	OXZ34290.1	B9N55_01770	330563	331837	1	phage portal protein
322334	331837	9503	OXZ34291.1	B9N55_01775	331827	332513	1	peptidase
340122	345701	5579	OXZ34303.1	B9N55_01835	340122	340484	1	hypothetical protein
340122	345701	5579	OXZ34304.1	B9N55_01840	340484	342355	1	hypothetical protein
340122	345701	5579	OXZ34305.1	B9N55_01845	342342	343721	1	hypothetical protein
340122	345701	5579	OXZ34306.1	B9N55_01850	343723	344124	1	holin
340122	345701	5579	OXZ34307.1	B9N55_01855	344125	344934	1	lysozyme
340122	345701	5579	OXZ34308.1	B9N55_01860	345111	345701	1	TetR family transcriptional regulator
365736	388679	22943	OXZ34323.1	B9N55_01945	365736	366485	1	phosphorylase
365736	388679	22943	OXZ34324.1	B9N55_01950	366790	367440	1	hypothetical protein
365736	388679	22943	OXZ34325.1	B9N55_01955	368197	369045	1	AraC family transcriptional regulator
365736	388679	22943	OXZ34346.1	B9N55_01960	369129	369554	1	abortive infection protein
365736	388679	22943		B9N55_01965	369808	370324	1	FRG domain-containing protein
365736	388679	22943	OXZ34326.1	B9N55_01970	370372	370926	1	hypothetical protein
365736	388679	22943		B9N55_01975	370978	371348	1	hypothetical protein
365736	388679	22943		B9N55_01980	371494	371690	1	transcriptional regulator
365736	388679	22943	OXZ34327.1	B9N55_01985	371742	372425	1	hypothetical protein
365736	388679	22943	OXZ34328.1	B9N55_01990	372418	373839	1	hypothetical protein
365736	388679	22943	OXZ34329.1	B9N55_01995	373991	374878	1	hypothetical protein
365736	388679	22943	OXZ34330.1	B9N55_02000	374862	375614	1	hypothetical protein
365736	388679	22943		B9N55_02005	375964	376360	1	hypothetical protein
365736	388679	22943	OXZ34331.1	B9N55_02010	376681	376884	1	hypothetical protein
365736	388679	22943	OXZ34332.1	B9N55_02015	376884	377798	1	ABC transporter ATP-binding protein
365736	388679	22943	OXZ34333.1	B9N55_02020	377795	378562	1	hypothetical protein
365736	388679	22943	OXZ34334.1	B9N55_02025	378576	379679	1	transcriptional regulator
365736	388679	22943	OXZ34335.1	B9N55_02030	379851	380051	1	transcriptional regulator
365736	388679	22943	OXZ34336.1	B9N55_02035	380210	380791	1	hypothetical protein
365736	388679	22943	OXZ34337.1	B9N55_02040	381213	382211	-1	AraC family transcriptional regulator
365736	388679	22943	OXZ34338.1	B9N55_02045	382372	384126	1	ABC transporter ATP-binding protein
365736	388679	22943	OXZ34339.1	B9N55_02050	384110	385831	1	ABC transporter ATP-binding protein
365736	388679	22943	OXZ34340.1	B9N55_02055	385843	387186	1	MATE family efflux transporter
365736	388679	22943	OXZ34341.1	B9N55_02060	387338	387591	-1	IS110 family transposase
365736	388679	22943	OXZ33675.1	B9N55_02065	388592	388679	1	hypothetical protein
365736	376884	11148	OXZ34323.1	B9N55_01945	365736	366485	1	phosphorylase
365736	376884	11148	OXZ34324.1	B9N55_01950	366790	367440	1	hypothetical protein

365736	376884	11148	OXZ34325.1	B9N55_01955	368197	369045	1	AraC family transcriptional regulator
365736	376884	11148	OXZ34346.1	B9N55_01960	369129	369554	1	abortive infection protein
365736	376884	11148		B9N55_01965	369808	370324	1	FRG domain-containing protein
365736	376884	11148	OXZ34326.1	B9N55_01970	370372	370926	1	hypothetical protein
365736	376884	11148		B9N55_01975	370978	371348	1	hypothetical protein
365736	376884	11148		B9N55_01980	371494	371690	1	transcriptional regulator
365736	376884	11148	OXZ34327.1	B9N55_01985	371742	372425	1	hypothetical protein
365736	376884	11148	OXZ34328.1	B9N55_01990	372418	373839	1	hypothetical protein
365736	376884	11148	OXZ34329.1	B9N55_01995	373991	374878	1	hypothetical protein
365736	376884	11148	OXZ34330.1	B9N55_02000	374862	375614	1	hypothetical protein
365736	376884	11148		B9N55_02005	375964	376360	1	hypothetical protein
365736	376884	11148	OXZ34331.1	B9N55_02010	376681	376884	1	hypothetical protein
379851	384126	4275	OXZ34335.1	B9N55_02030	379851	380051	1	transcriptional regulator
379851	384126	4275	OXZ34336.1	B9N55_02035	380210	380791	1	hypothetical protein
379851	384126	4275	OXZ34337.1	B9N55_02040	381213	382211	-1	AraC family transcriptional regulator
379851	384126	4275	OXZ34338.1	B9N55_02045	382372	384126	1	ABC transporter ATP-binding protein
379851	384126	4275	OXZ34339.1	B9N55_02050	384110	385831	1	ABC transporter ATP-binding protein
569426	590255	20829	OXZ33831.1	B9N55_02880	568056	569435	-1	two-component sensor histidine kinase
569426	590255	20829	OXZ33832.1	B9N55_02885	569426	570085	-1	DNA-binding response regulator
569426	590255	20829	OXZ33833.1	B9N55_02890	570118	570858	-1	mutacin I biosynthesis protein MutG
569426	590255	20829	OXZ33834.1	B9N55_02895	570858	571598	-1	antibiotic ABC transporter permease
569426	590255	20829	OXZ33835.1	B9N55_02900	571602	572303	-1	lantibiotic ABC transporter ATP-binding protein
569426	590255	20829	OXZ33836.1	B9N55_02905	572915	574204	1	hypothetical protein
569426	590255	20829	OXZ33837.1	B9N55_02910	574703	575230	1	transcriptional regulator
569426	590255	20829		B9N55_02915	575250	575632	1	conjugal transfer protein TraA
569426	590255	20829	OXZ33838.1	B9N55_02920	575768	576340	1	uracil-DNA glycosylase
569426	590255	20829	OXZ33839.1	B9N55_02925	576408	576695	1	cupin
569426	590255	20829	OXZ33840.1	B9N55_02930	577333	579390	1	elongation factor G
569426	590255	20829	OXZ33841.1	B9N55_02935	579752	580417	-1	hypothetical protein
569426	590255	20829	OXZ33842.1	B9N55_02940	580526	581422	1	cysteine synthase A
569426	590255	20829	OXZ33843.1	B9N55_02945	581409	581933	1	serine O-acetyltransferase
569426	590255	20829	OXZ33844.1	B9N55_02950	582150	583034	-1	hypothetical protein
569426	590255	20829		B9N55_02955	583083	583187	-1	hypothetical protein
569426	590255	20829	OXZ33845.1	B9N55_02960	583681	584769	1	iron ABC transporter permease
569426	590255	20829	OXZ33846.1	B9N55_02965	584759	585766	1	iron ABC transporter
569426	590255	20829	OXZ33847.1	B9N55_02970	585766	586773	1	iron ABC transporter
569426	590255	20829	OXZ33848.1	B9N55_02975	586773	587567	1	iron ABC transporter ATP-binding protein
569426	590255	20829	OXZ33849.1	B9N55_02980	587970	588371	1	hypothetical protein
569426	590255	20829		B9N55_02985	588429	589043	1	hypothetical protein
569426	590255	20829	OXZ33850.1	B9N55_02990	589164	590255	1	glycerol dehydrogenase
1088002	1095275	7273	OXZ32287.1	B9N55_05575	1088002	1088982	-1	recombinase XerC
1088002	1095275	7273	OXZ32288.1	B9N55_05580	1089092	1090267	-1	aspartate aminotransferase
1088002	1095275	7273	OXZ32289.1	B9N55_05585	1090254	1091075	-1	metal-dependent phosphoesterase
1088002	1095275	7273	OXZ32290.1	B9N55_05590	1091144	1092685	-1	ribonuclease Y
1088002	1095275	7273	OXZ32291.1	B9N55_05595	1092804	1093466	-1	chromosome partitioning protein ParA
1088002	1095275	7273	OXZ32292.1	B9N55_05600	1093453	1093692	-1	hypothetical protein
1088002	1095275	7273	OXZ32293.1	B9N55_05605	1093692	1095275	-1	exonuclease SbcC
1293167	1324490	31323	OXZ32025.1	B9N55_06545	1293167	1294498	1	endonuclease
1293167	1324490	31323	OXZ32026.1	B9N55_06550	1294609	1295265	-1	DNA-binding protein
1293167	1324490	31323	OXZ32027.1	B9N55_06555	1295309	1296637	-1	helicase
1293167	1324490	31323	OXZ32028.1	B9N55_06560	1296771	1298441	-1	group II intron reverse transcriptase/maturase
1293167	1324490	31323	OXZ32029.1	B9N55_06565	1298997	1305293	-1	DNA helicase
1293167	1324490	31323	OXZ32030.1	B9N55_06570	1305377	1306879	-1	SIR2 family protein
1293167	1324490	31323	OXZ32031.1	B9N55_06575	1306936	1307874	-1	DNA (cytosine-5-)-methyltransferase
1293167	1324490	31323	OXZ32032.1	B9N55_06580	1307867	1309588	-1	DNA topoisomerase III
1293167	1324490	31323	OXZ32033.1	B9N55_06585	1309678	1310661	-1	hypothetical protein
1293167	1324490	31323	OXZ32034.1	B9N55_06590	1310654	1311265	-1	hypothetical protein
1293167	1324490	31323	OXZ32035.1	B9N55_06595	1311384	1312199	-1	bacteriocin
1293167	1324490	31323	OXZ32036.1	B9N55_06600	1312214	1313797	-1	copper amine oxidase
1293167	1324490	31323		B9N55_06605	1313787	1314023	-1	hypothetical protein
1293167	1324490	31323	OXZ32037.1	B9N55_06610	1314038	1316617	-1	hypothetical protein
1293167	1324490	31323	OXZ32038.1	B9N55_06615	1316625	1319048	-1	conjugal transfer protein TraE
1293167	1324490	31323	OXZ32039.1	B9N55_06620	1318939	1319349	-1	conjugal transfer protein
1293167	1324490	31323	OXZ32040.1	B9N55_06625	1319351	1319641	-1	hypothetical protein
1293167	1324490	31323	OXZ32041.1	B9N55_06630	1319651	1320514	-1	hypothetical protein
1293167	1324490	31323	OXZ32042.1	B9N55_06635	1320525	1320740	-1	conjugal transfer protein
1293167	1324490	31323	OXZ32043.1	B9N55_06640	1320742	1321053	-1	single-stranded DNA-binding protein
1293167	1324490	31323	OXZ32093.1	B9N55_06645	1321276	1321893	-1	hypothetical protein
1293167	1324490	31323	OXZ32044.1	B9N55_06650	1321961	1322383	-1	polyketide cyclase
1293167	1324490	31323	OXZ32045.1	B9N55_06655	1322421	1322939	-1	cytoplasmic protein
1293167	1324490	31323		B9N55_06660	1323001	1323468	-1	conjugal transfer protein TraG
1293167	1324490	31323	OXZ32046.1	B9N55_06665	1323506	1323781	-1	hypothetical protein
1293167	1324490	31323	OXZ32047.1	B9N55_06670	1324050	1324490	-1	GNAT family N-acetyltransferase
1305377	1312199	6822	OXZ32030.1	B9N55_06570	1305377	1306879	-1	SIR2 family protein
1305377	1312199	6822	OXZ32031.1	B9N55_06575	1306936	1307874	-1	DNA (cytosine-5-)-methyltransferase
1305377	1312199	6822	OXZ32032.1	B9N55_06580	1307867	1309588	-1	DNA topoisomerase III
1305377	1312199	6822	OXZ32033.1	B9N55_06585	1309678	1310661	-1	hypothetical protein
1305377	1312199	6822	OXZ32034.1	B9N55_06590	1310654	1311265	-1	hypothetical protein
1305377	1312199	6822	OXZ32035.1	B9N55_06595	1311384	1312199	-1	bacteriocin
1320742	1325827	5085	OXZ32043.1	B9N55_06640	1320742	1321053	-1	single-stranded DNA-binding protein
1320742	1325827	5085	OXZ32093.1	B9N55_06645	1321276	1321893	-1	hypothetical protein
1320742	1325827	5085	OXZ32044.1	B9N55_06650	1321961	1322383	-1	polyketide cyclase
1320742	1325827	5085	OXZ32045.1	B9N55_06655	1322421	1322939	-1	cytoplasmic protein
1320742	1325827	5085		B9N55_06660	1323001	1323468	-1	conjugal transfer protein TraG
1320742	1325827	5085	OXZ32046.1	B9N55_06665	1323506	1323781	-1	hypothetical protein
1320742	1325827	5085	OXZ32047.1	B9N55_06670	1324050	1324490	-1	GNAT family N-acetyltransferase

1320742	1325827	5085	OXZ32048.1	B9N55_06675	1324550	1324753	-1	hypothetical protein
1320742	1325827	5085	OXZ32049.1	B9N55_06680	1324815	1325600	-1	conjugal transfer protein TraG
1320742	1325827	5085	OXZ32050.1	B9N55_06685	1325642	1325827	-1	hypothetical protein
1331207	1344342	13135	OXZ32057.1	B9N55_06720	1331207	1332949	-1	ABC transporter ATP-binding protein
1331207	1344342	13135	OXZ32058.1	B9N55_06725	1332951	1334663	-1	ABC transporter ATP-binding protein
1331207	1344342	13135	OXZ32059.1	B9N55_06730	1334667	1336082	-1	ABC transporter ATP-binding protein
1331207	1344342	13135	OXZ32060.1	B9N55_06735	1336095	1336781	-1	cobalt transporter
1331207	1344342	13135	OXZ32061.1	B9N55_06740	1336782	1337375	-1	hypothetical protein
1331207	1344342	13135	OXZ32062.1	B9N55_06745	1337547	1338158	-1	TetR family transcriptional regulator
1331207	1344342	13135	OXZ32063.1	B9N55_06750	1338259	1340085	-1	recombinase TnpX
1331207	1344342	13135	OXZ32064.1	B9N55_06755	1340125	1341177	-1	conjugal transfer protein TraG
1331207	1344342	13135	OXZ32065.1	B9N55_06760	1341170	1341904	-1	phage antirepressor Ant
1331207	1344342	13135	OXZ32066.1	B9N55_06765	1341901	1342386	-1	conjugal transfer protein
1331207	1344342	13135	OXZ32067.1	B9N55_06770	1342379	1343206	-1	nucleoside triphosphate hydrolase
1331207	1344342	13135	OXZ32068.1	B9N55_06775	1343203	1343976	-1	replication initiator protein A
1331207	1344342	13135	OXZ32069.1	B9N55_06780	1344061	1344342	-1	hypothetical protein
1684263	1728424	44161	OXZ31633.1	B9N55_08365	1684263	1685537	-1	hypothetical protein
1684263	1728424	44161	OXZ31634.1	B9N55_08370	1685632	1687314	-1	hypothetical protein
1684263	1728424	44161	OXZ31344.1	B9N55_08375	1688746	1690407	-1	ABC transporter permease
1684263	1728424	44161	OXZ31345.1	B9N55_08380	1690400	1690666	-1	CXXX repeat peptide modification system protein
1684263	1728424	44161	OXZ31346.1	B9N55_08385	1690668	1692857	-1	radical SAM protein
1684263	1728424	44161	OXZ31347.1	B9N55_08390	1692844	1693026	-1	hypothetical protein
1684263	1728424	44161	OXZ31348.1	B9N55_08395	1693156	1693761	-1	DNA-binding response regulator
1684263	1728424	44161	OXZ31349.1	B9N55_08400	1693733	1695280	-1	hypothetical protein
1684263	1728424	44161	OXZ31350.1	B9N55_08405	1695594	1696688	-1	single-stranded DNA-binding protein
1684263	1728424	44161	OXZ31351.1	B9N55_08410	1696721	1707151	-1	conjugal transfer protein
1684263	1728424	44161		B9N55_08415	1707346	1707905	-1	antibiotic ABC transporter
1684263	1728424	44161	OXZ31352.1	B9N55_08420	1708184	1709890	-1	DNA topoisomerase III
1684263	1728424	44161	OXZ31353.1	B9N55_08425	1710307	1711185	-1	bacteriocin
1684263	1728424	44161	OXZ31354.1	B9N55_08430	1711201	1713555	-1	copper amine oxidase
1684263	1728424	44161	OXZ31355.1	B9N55_08435	1713552	1713809	-1	hypothetical protein
1684263	1728424	44161	OXZ31356.1	B9N55_08440	1713829	1716792	-1	hypothetical protein
1684263	1728424	44161	OXZ31357.1	B9N55_08445	1716802	1717767	-1	site-specific DNA-methyltransferase
1684263	1728424	44161	OXZ31358.1	B9N55_08450	1717739	1720234	-1	conjugal transfer protein TraE
1684263	1728424	44161	OXZ31359.1	B9N55_08455	1720231	1720569	-1	conjugal transfer protein
1684263	1728424	44161	OXZ31360.1	B9N55_08460	1720569	1720826	-1	hypothetical protein
1684263	1728424	44161	OXZ31361.1	B9N55_08465	1720844	1721707	-1	hypothetical protein
1684263	1728424	44161	OXZ31362.1	B9N55_08470	1721709	1721924	-1	conjugal transfer protein
1684263	1728424	44161	OXZ31363.1	B9N55_08475	1721925	1722248	-1	single-stranded DNA-binding protein
1684263	1728424	44161	OXZ31364.1	B9N55_08480	1722438	1723301	-1	CAAX protease
1684263	1728424	44161	OXZ31365.1	B9N55_08485	1723453	1725255	-1	conjugal transfer protein TraG
1684263	1728424	44161	OXZ31366.1	B9N55_08490	1725300	1725503	-1	hypothetical protein
1684263	1728424	44161	OXZ31367.1	B9N55_08495	1725692	1726183	-1	hypothetical protein
1684263	1728424	44161	OXZ31368.1	B9N55_08500	1726198	1727055	-1	DNA replication protein
1684263	1728424	44161	OXZ31369.1	B9N55_08505	1727070	1728047	-1	replication initiator RepA
1684263	1728424	44161	OXZ31370.1	B9N55_08510	1728134	1728424	-1	hypothetical protein
1690400	1716792	26392	OXZ31344.1	B9N55_08375	1688746	1690407	-1	ABC transporter permease
1690400	1716792	26392	OXZ31345.1	B9N55_08380	1690400	1690666	-1	CXXX repeat peptide modification system protein
1690400	1716792	26392	OXZ31346.1	B9N55_08385	1690668	1692857	-1	radical SAM protein
1690400	1716792	26392	OXZ31347.1	B9N55_08390	1692844	1693026	-1	hypothetical protein
1690400	1716792	26392	OXZ31348.1	B9N55_08395	1693156	1693761	-1	DNA-binding response regulator
1690400	1716792	26392	OXZ31349.1	B9N55_08400	1693733	1695280	-1	hypothetical protein
1690400	1716792	26392	OXZ31350.1	B9N55_08405	1695594	1696688	-1	single-stranded DNA-binding protein
1690400	1716792	26392	OXZ31351.1	B9N55_08410	1696721	1707151	-1	conjugal transfer protein
1690400	1716792	26392		B9N55_08415	1707346	1707905	-1	antibiotic ABC transporter
1690400	1716792	26392	OXZ31352.1	B9N55_08420	1708184	1709890	-1	DNA topoisomerase III
1690400	1716792	26392	OXZ31353.1	B9N55_08425	1710307	1711185	-1	bacteriocin
1690400	1716792	26392	OXZ31354.1	B9N55_08430	1711201	1713555	-1	copper amine oxidase
1690400	1716792	26392	OXZ31355.1	B9N55_08435	1713552	1713809	-1	hypothetical protein
1690400	1716792	26392	OXZ31356.1	B9N55_08440	1713829	1716792	-1	hypothetical protein
1720231	1728424	8193	OXZ31358.1	B9N55_08450	1717739	1720234	-1	conjugal transfer protein TraE
1720231	1728424	8193	OXZ31359.1	B9N55_08455	1720231	1720569	-1	conjugal transfer protein
1720231	1728424	8193	OXZ31360.1	B9N55_08460	1720569	1720826	-1	hypothetical protein
1720231	1728424	8193	OXZ31361.1	B9N55_08465	1720844	1721707	-1	hypothetical protein
1720231	1728424	8193	OXZ31362.1	B9N55_08470	1721709	1721924	-1	conjugal transfer protein
1720231	1728424	8193	OXZ31363.1	B9N55_08475	1721925	1722248	-1	single-stranded DNA-binding protein
1720231	1728424	8193	OXZ31364.1	B9N55_08480	1722438	1723301	-1	CAAX protease
1720231	1728424	8193	OXZ31365.1	B9N55_08485	1723453	1725255	-1	conjugal transfer protein TraG
1720231	1728424	8193	OXZ31366.1	B9N55_08490	1725300	1725503	-1	hypothetical protein
1720231	1728424	8193	OXZ31367.1	B9N55_08495	1725692	1726183	-1	hypothetical protein
1720231	1728424	8193	OXZ31368.1	B9N55_08500	1726198	1727055	-1	DNA replication protein
1720231	1728424	8193	OXZ31369.1	B9N55_08505	1727070	1728047	-1	replication initiator RepA
1720231	1728424	8193	OXZ31370.1	B9N55_08510	1728134	1728424	-1	hypothetical protein
1799490	1811368	11878	OXZ31185.1	B9N55_08875	1799490	1800239	-1	phosphorylase
1799490	1811368	11878		B9N55_08880	1800273	1800602	-1	hypothetical protein
1799490	1811368	11878	OXZ31186.1	B9N55_08885	1800703	1801284	-1	hypothetical protein
1799490	1811368	11878	OXZ31187.1	B9N55_08890	1801737	1802165	-1	hypothetical protein
1799490	1811368	11878	OXZ31188.1	B9N55_08895	1802314	1803678	-1	ABC transporter ATP-binding protein
1799490	1811368	11878	OXZ31189.1	B9N55_08900	1803672	1804361	-1	cobalt ABC transporter permease
1799490	1811368	11878	OXZ31190.1	B9N55_08905	1804361	1804942	-1	hypothetical protein
1799490	1811368	11878	OXZ31024.1	B9N55_09060	1806353	1808938	-1	ATP-dependent chaperone ClpB
1799490	1811368	11878	OXZ31025.1	B9N55_09065	1808957	1809901	-1	heat-shock protein
1799490	1811368	11878	OXZ31026.1	B9N55_09070	1810010	1810675	-1	nitroreductase
1799490	1811368	11878	OXZ31027.1	B9N55_09075	1810829	1811368	-1	RNA polymerase subunit sigma
1897911	1902891	4980	OXZ30960.1	B9N55_09420	1897911	1897998	-1	hypothetical protein

1897911	1902891	4980	OXZ30822.1	B9N55_09480	1901495	1902157	-1	hypothetical protein
1897911	1902891	4980	OXZ30823.1	B9N55_09485	1902352	1902891	-1	transposase
1914240	1959903	45663	OXZ30881.1	B9N55_09450	1914240	1914451	-1	translation elongation factor Tu
1914240	1959903	45663	OXZ31113.1	B9N55_08910	1915452	1917072	-1	hypothetical protein
1914240	1959903	45663	OXZ31114.1	B9N55_08915	1917069	1918730	-1	hypothetical protein
1914240	1959903	45663	OXZ31115.1	B9N55_08920	1918731	1920428	-1	hypothetical protein
1914240	1959903	45663	OXZ31116.1	B9N55_08925	1920448	1921431	-1	3-deoxy-7-phosphoheptulonate synthase
1914240	1959903	45663	OXZ31117.1	B9N55_08930	1921436	1921774	-1	dehydroquinase class II
1914240	1959903	45663		B9N55_08935	1921773	1922009	-1	chorismate synthase
1914240	1959903	45663		B9N55_08940	1921996	1923290	-1	3-phosphoshikimate 1-carboxyvinyltransferase
1914240	1959903	45663	OXZ31118.1	B9N55_08945	1923287	1924333	-1	3-dehydroquinate synthase
1914240	1959903	45663	OXZ31119.1	B9N55_08950	1924337	1924723	-1	salicylate synthase
1914240	1959903	45663	OXZ31120.1	B9N55_08955	1924888	1925148	-1	hypothetical protein
1914240	1959903	45663	OXZ31121.1	B9N55_08960	1925141	1925569	-1	hypothetical protein
1914240	1959903	45663	OXZ31122.1	B9N55_08965	1925892	1926284	-1	transcriptional regulator
1914240	1959903	45663	OXZ31123.1	B9N55_08970	1926721	1927089	1	plasmid mobilization relaxosome protein MobC
1914240	1959903	45663	OXZ31124.1	B9N55_08975	1927089	1928459	1	endonuclease
1914240	1959903	45663	OXZ31125.1	B9N55_08980	1928518	1929264	-1	ABC transporter permease
1914240	1959903	45663	OXZ31126.1	B9N55_08985	1929279	1929971	-1	ABC transporter permease
1914240	1959903	45663	OXZ31127.1	B9N55_08990	1929973	1930887	-1	bacitracin ABC transporter ATP-binding protein
1914240	1959903	45663	OXZ31128.1	B9N55_08995	1930902	1932059	-1	hypothetical protein
1914240	1959903	45663	OXZ31129.1	B9N55_09000	1932063	1933370	-1	hypothetical protein
1914240	1959903	45663	OXZ31130.1	B9N55_09005	1934009	1934440	-1	hypothetical protein
1914240	1959903	45663	OXZ31131.1	B9N55_09010	1934453	1934716	-1	hypothetical protein
1914240	1959903	45663	OXZ31132.1	B9N55_09015	1934710	1935279	-1	hypothetical protein
1914240	1959903	45663		B9N55_09020	1935618	1935941	1	relaxase
1914240	1959903	45663	OXZ31133.1	B9N55_09025	1936062	1936409	-1	DUF3784 domain-containing protein
1914240	1959903	45663	OXZ31134.1	B9N55_09030	1936417	1937793	-1	hypothetical protein
1914240	1959903	45663	OXZ31135.1	B9N55_09035	1937803	1938597	-1	hypothetical protein
1914240	1959903	45663	OXZ31136.1	B9N55_09040	1938587	1939453	-1	hypothetical protein
1914240	1959903	45663	OXZ31137.1	B9N55_09045	1939635	1940819	-1	hypothetical protein
1914240	1959903	45663	OXZ31138.1	B9N55_09050	1940947	1941279	-1	hypothetical protein
1914240	1959903	45663	OXZ31139.1	B9N55_09055	1941537	1941746	-1	hypothetical protein
1914240	1959903	45663	OXZ30824.1	B9N55_09470	1948165	1948863	-1	hypothetical protein
1914240	1959903	45663	OXZ30825.1	B9N55_09475	1949022	1949561	-1	transposase
1914240	1959903	45663	OXZ30779.1	B9N55_09500	1950607	1951809	-1	hypothetical protein
1914240	1959903	45663	OXZ30778.1	B9N55_09505	1952810	1953858	-1	IS110 family transposase
1914240	1959903	45663	OXZ30943.1	B9N55_09440	1955001	1959903	-1	hypothetical protein
1915452	1948863	33411	OXZ31113.1	B9N55_08910	1915452	1917072	-1	hypothetical protein
1915452	1948863	33411	OXZ31114.1	B9N55_08915	1917069	1918730	-1	hypothetical protein
1915452	1948863	33411	OXZ31115.1	B9N55_08920	1918731	1920428	-1	hypothetical protein
1915452	1948863	33411	OXZ31116.1	B9N55_08925	1920448	1921431	-1	3-deoxy-7-phosphoheptulonate synthase
1915452	1948863	33411	OXZ31117.1	B9N55_08930	1921436	1921774	-1	dehydroquinase class II
1915452	1948863	33411		B9N55_08935	1921773	1922009	-1	chorismate synthase
1915452	1948863	33411		B9N55_08940	1921996	1923290	-1	3-phosphoshikimate 1-carboxyvinyltransferase
1915452	1948863	33411	OXZ31118.1	B9N55_08945	1923287	1924333	-1	3-dehydroquinate synthase
1915452	1948863	33411	OXZ31119.1	B9N55_08950	1924337	1924723	-1	salicylate synthase
1915452	1948863	33411	OXZ31120.1	B9N55_08955	1924888	1925148	-1	hypothetical protein
1915452	1948863	33411	OXZ31121.1	B9N55_08960	1925141	1925569	-1	hypothetical protein
1915452	1948863	33411	OXZ31122.1	B9N55_08965	1925892	1926284	-1	transcriptional regulator
1915452	1948863	33411	OXZ31123.1	B9N55_08970	1926721	1927089	1	plasmid mobilization relaxosome protein MobC
1915452	1948863	33411	OXZ31124.1	B9N55_08975	1927089	1928459	1	endonuclease
1915452	1948863	33411	OXZ31125.1	B9N55_08980	1928518	1929264	-1	ABC transporter permease
1915452	1948863	33411	OXZ31126.1	B9N55_08985	1929279	1929971	-1	ABC transporter permease
1915452	1948863	33411	OXZ31127.1	B9N55_08990	1929973	1930887	-1	bacitracin ABC transporter ATP-binding protein
1915452	1948863	33411	OXZ31128.1	B9N55_08995	1930902	1932059	-1	hypothetical protein
1915452	1948863	33411	OXZ31129.1	B9N55_09000	1932063	1933370	-1	hypothetical protein
1915452	1948863	33411	OXZ31130.1	B9N55_09005	1934009	1934440	-1	hypothetical protein
1915452	1948863	33411	OXZ31131.1	B9N55_09010	1934453	1934716	-1	hypothetical protein
1915452	1948863	33411	OXZ31132.1	B9N55_09015	1934710	1935279	-1	hypothetical protein
1915452	1948863	33411		B9N55_09020	1935618	1935941	1	relaxase
1915452	1948863	33411	OXZ31133.1	B9N55_09025	1936062	1936409	-1	DUF3784 domain-containing protein
1915452	1948863	33411	OXZ31134.1	B9N55_09030	1936417	1937793	-1	hypothetical protein
1915452	1948863	33411	OXZ31135.1	B9N55_09035	1937803	1938597	-1	hypothetical protein
1915452	1948863	33411	OXZ31136.1	B9N55_09040	1938587	1939453	-1	hypothetical protein
1915452	1948863	33411	OXZ31137.1	B9N55_09045	1939635	1940819	-1	hypothetical protein
1915452	1948863	33411	OXZ31138.1	B9N55_09050	1940947	1941279	-1	hypothetical protein
1915452	1948863	33411	OXZ31139.1	B9N55_09055	1941537	1941746	-1	hypothetical protein
1915452	1948863	33411	OXZ30824.1	B9N55_09470	1948165	1948863	-1	hypothetical protein

Table S3G: Predicted genomic islands in the genome of *"F. nericia"* 12T306

Shown are genes of genomic islands predicted by IslandViewer4

Island start	Island end	Length	Gene name	Locus	Gene start	Gene end	Strand	Product
125213	131694	6481	OXZ36396.1	B9N54_00540	125213	126550	1	replicative DNA helicase
125213	131694	6481	OXZ36397.1	B9N54_00545	126547	127110	1	GNAT family N-acetyltransferase
125213	131694	6481	OXZ36398.1	B9N54_00550	127115	127873	1	DNA replication protein
125213	131694	6481	OXZ36399.1	B9N54_00555	127876	128853	1	DNA replication protein DnaC
125213	131694	6481	OXZ36400.1	B9N54_00560	128850	129494	1	NAD(P)H-hydrate epimerase
125213	131694	6481	OXZ36401.1	B9N54_00565	130482	131024	1	ECF transporter S component
125213	131694	6481	OXZ36402.1	B9N54_00570	131008	131694	1	radical SAM protein
229146	236132	6986	OXZ36325.1	B9N54_01130	229146	229787	-1	hemolysin III
229146	236132	6986	OXZ36326.1	B9N54_01135	229940	230443	1	acetyltransferase
229146	236132	6986	OXZ36327.1	B9N54_01140	230443	231177	1	hydrolase
229146	236132	6986	OXZ36328.1	B9N54_01145	232657	233232	1	cell filamentation protein Fic
229146	236132	6986	OXZ36329.1	B9N54_01150	234109	234351	-1	prevent-host-death protein
229146	236132	6986	OXZ36330.1	B9N54_01155	234514	235122	-1	hypothetical protein
229146	236132	6986	OXZ36331.1	B9N54_01160	235206	236132	-1	hypothetical protein
280334	298282	17948	OXZ36371.1	B9N54_01370	280334	281362	1	carboxypeptidase
280334	298282	17948	OXZ36372.1	B9N54_01375	281715	282143	1	hypothetical protein
280334	298282	17948	OXZ36373.1	B9N54_01380	282297	283046	1	phosphorylase
280334	298282	17948	OXZ36374.1	B9N54_01385	283352	284002	1	hypothetical protein
280334	298282	17948	OXZ36375.1	B9N54_01390	284842	285633	1	CPBP family intramembrane metalloprotease
280334	298282	17948	OXZ36376.1	B9N54_01395	285695	286162	1	abortive infection protein
280334	298282	17948		B9N54_01400	286395	286911	1	FRG domain-containing protein
280334	298282	17948	OXZ36377.1	B9N54_01405	286959	287513	1	hypothetical protein
280334	298282	17948		B9N54_01410	287548	287942	1	hypothetical protein
280334	298282	17948	OXZ36378.1	B9N54_01415	288232	289779	1	hypothetical protein
280334	298282	17948	OXZ36379.1	B9N54_01420	289751	290356	1	DNA-binding response regulator
280334	298282	17948	OXZ36380.1	B9N54_01425	290535	290939	1	hypothetical protein
280334	298282	17948	OXZ36381.1	B9N54_01430	290994	291878	1	multidrug ABC transporter ATP-binding protein
280334	298282	17948	OXZ36382.1	B9N54_01435	291862	292587	1	hypothetical protein
280334	298282	17948		B9N54_01440	292680	292882	1	hypothetical protein
280334	298282	17948	OXZ36383.1	B9N54_01445	293661	294560	1	bacitracin ABC transporter ATP-binding protein
280334	298282	17948	OXZ36384.1	B9N54_01450	294557	295399	1	ABC transporter permease
280334	298282	17948		B9N54_01455	295465	295956	1	ABC transporter permease
280334	298282	17948	OXZ36385.1	B9N54_01460	295970	296236	1	hypothetical protein
280334	298282	17948	OXZ36386.1	B9N54_01465	296310	296704	-1	relaxase
280334	298282	17948	OXZ36136.1	B9N54_01470	297705	297829	-1	transposase
280334	298282	17948	OXZ36137.1	B9N54_01475	297893	298282	-1	hypothetical protein
281715	298282	16567	OXZ36372.1	B9N54_01375	281715	282143	1	hypothetical protein
281715	298282	16567	OXZ36373.1	B9N54_01380	282297	283046	1	phosphorylase
281715	298282	16567	OXZ36374.1	B9N54_01385	283352	284002	1	hypothetical protein
281715	298282	16567	OXZ36375.1	B9N54_01390	284842	285633	1	CPBP family intramembrane metalloprotease
281715	298282	16567	OXZ36376.1	B9N54_01395	285695	286162	1	abortive infection protein
281715	298282	16567		B9N54_01400	286395	286911	1	FRG domain-containing protein
281715	298282	16567	OXZ36377.1	B9N54_01405	286959	287513	1	hypothetical protein
281715	298282	16567		B9N54_01410	287548	287942	1	hypothetical protein
281715	298282	16567	OXZ36378.1	B9N54_01415	288232	289779	1	hypothetical protein
281715	298282	16567	OXZ36379.1	B9N54_01420	289751	290356	1	DNA-binding response regulator
281715	298282	16567	OXZ36380.1	B9N54_01425	290535	290939	1	hypothetical protein
281715	298282	16567	OXZ36381.1	B9N54_01430	290994	291878	1	multidrug ABC transporter ATP-binding protein
281715	298282	16567	OXZ36382.1	B9N54_01435	291862	292587	1	hypothetical protein
281715	298282	16567		B9N54_01440	292680	292882	1	hypothetical protein
281715	298282	16567	OXZ36383.1	B9N54_01445	293661	294560	1	bacitracin ABC transporter ATP-binding protein
281715	298282	16567	OXZ36384.1	B9N54_01450	294557	295399	1	ABC transporter permease
281715	298282	16567		B9N54_01455	295465	295956	1	ABC transporter permease
281715	298282	16567	OXZ36385.1	B9N54_01460	295970	296236	1	hypothetical protein
281715	298282	16567	OXZ36386.1	B9N54_01465	296310	296704	-1	relaxase
281715	298282	16567	OXZ36136.1	B9N54_01470	297705	297829	-1	transposase
281715	298282	16567	OXZ36137.1	B9N54_01475	297893	298282	-1	hypothetical protein
454821	459306	4485	OXZ36294.1	B9N54_02190	454821	455048	1	mobilization protein
454821	459306	4485	OXZ36273.1	B9N54_02195	455377	455802	-1	MarR family transcriptional regulator
454821	459306	4485	OXZ36274.1	B9N54_02200	455804	456178	-1	hypothetical protein
454821	459306	4485		B9N54_02205	456629	456724	-1	glutamyl-tRNA amidotransferase
454821	459306	4485	OXZ36275.1	B9N54_02210	456938	457369	-1	transcriptional regulator
454821	459306	4485	OXZ36276.1	B9N54_02215	457631	457825	1	hypothetical protein
454821	459306	4485	OXZ36277.1	B9N54_02220	457827	458951	1	site-specific integrase
454821	459306	4485	OXZ36278.1	B9N54_02225	458944	459306	1	hypothetical protein
455377	468722	13345	OXZ36273.1	B9N54_02195	455377	455802	-1	MarR family transcriptional regulator
455377	468722	13345	OXZ36274.1	B9N54_02200	455804	456178	-1	hypothetical protein
455377	468722	13345		B9N54_02205	456629	456724	-1	glutamyl-tRNA amidotransferase
455377	468722	13345	OXZ36275.1	B9N54_02210	456938	457369	-1	transcriptional regulator
455377	468722	13345	OXZ36276.1	B9N54_02215	457631	457825	1	hypothetical protein
455377	468722	13345	OXZ36277.1	B9N54_02220	457827	458951	1	site-specific integrase
455377	468722	13345	OXZ36278.1	B9N54_02225	458944	459306	1	hypothetical protein
455377	468722	13345	OXZ36279.1	B9N54_02230	459424	460914	1	virulence-associated protein E
455377	468722	13345	OXZ36280.1	B9N54_02235	461243	461605	1	plasmid mobilization relaxosome protein MobC
455377	468722	13345	OXZ36281.1	B9N54_02240	461612	461860	1	hypothetical protein
455377	468722	13345	OXZ36295.1	B9N54_02245	461947	462342	1	hypothetical protein
455377	468722	13345	OXZ36282.1	B9N54_02250	462352	463794	1	hypothetical protein
455377	468722	13345	OXZ36283.1	B9N54_02255	463886	464056	1	XRE family transcriptional regulator
455377	468722	13345	OXZ36284.1	B9N54_02260	464147	464470	1	mobilization protein

455377	468722	13345	OXZ36285.1	B9N54_02265	464472	464711	1	hypothetical protein
455377	468722	13345	OXZ36286.1	B9N54_02270	464735	467233	-1	ABC transporter permease
455377	468722	13345	OXZ36287.1	B9N54_02275	467220	467909	-1	peptide ABC transporter ATP-binding protein
455377	468722	13345	OXZ36288.1	B9N54_02280	468185	468722	1	IS110 family transposase
461243	468722	7479	OXZ36280.1	B9N54_02235	461243	461605	1	plasmid mobilization relaxosome protein MobC
461243	468722	7479	OXZ36281.1	B9N54_02240	461612	461860	1	hypothetical protein
461243	468722	7479	OXZ36295.1	B9N54_02245	461947	462342	1	hypothetical protein
461243	468722	7479	OXZ36282.1	B9N54_02250	462352	463794	1	hypothetical protein
461243	468722	7479	OXZ36283.1	B9N54_02255	463886	464056	1	XRE family transcriptional regulator
461243	468722	7479	OXZ36284.1	B9N54_02260	464147	464470	1	mobilization protein
461243	468722	7479	OXZ36285.1	B9N54_02265	464472	464711	1	hypothetical protein
461243	468722	7479	OXZ36286.1	B9N54_02270	464735	467233	-1	ABC transporter permease
461243	468722	7479	OXZ36287.1	B9N54_02275	467220	467909	-1	peptide ABC transporter ATP-binding protein
461243	468722	7479	OXZ36288.1	B9N54_02280	468185	468722	1	IS110 family transposase
872345	880377	8032	OXZ35246.1	B9N54_04320	872345	873178	1	hypothetical protein
872345	880377	8032	OXZ35247.1	B9N54_04325	873299	873718	-1	flavodoxin
872345	880377	8032	OXZ35248.1	B9N54_04330	873722	874267	-1	hypothetical protein
872345	880377	8032	OXZ35249.1	B9N54_04335	874473	874697	1	preprotein translocase subunit SecG
872345	880377	8032	OXZ35250.1	B9N54_04340	874722	876734	1	sodium-translocating pyrophosphatase
872345	880377	8032	OXZ35251.1	B9N54_04345	876871	876995	1	transposase
872345	880377	8032	OXZ34999.1	B9N54_04350	877996	878159	1	hypothetical protein
872345	880377	8032	OXZ35000.1	B9N54_04355	878282	879610	1	hemolysin
872345	880377	8032	OXZ35001.1	B9N54_04360	879622	880377	1	type I methionyl aminopeptidase
895090	901022	5932	OXZ35013.1	B9N54_04435	895090	895281	1	50S ribosomal protein L35
895090	901022	5932	OXZ35014.1	B9N54_04440	895300	895653	1	50S ribosomal protein L20
895090	901022	5932	OXZ35015.1	B9N54_04445	895685	895960	-1	hypothetical protein
895090	901022	5932	OXZ35016.1	B9N54_04450	895960	896577	-1	recombinase RecX
895090	901022	5932	OXZ35017.1	B9N54_04455	896766	897005	1	hypothetical protein
895090	901022	5932	OXZ35018.1	B9N54_04460	897014	898090	1	phosphoesterase
895090	901022	5932	OXZ35019.1	B9N54_04465	898087	900339	1	DNA repair protein Rad50
895090	901022	5932	OXZ35020.1	B9N54_04470	900342	901022	1	serine/threonine protein phosphatase
949309	953561	4252	OXZ34685.1	B9N54_04705	949309	949935	-1	LexA repressor
949309	953561	4252	OXZ34686.1	B9N54_04710	950110	950421	1	peptidoglycan-binding protein LysM
949309	953561	4252	OXZ34687.1	B9N54_04715	950497	951477	-1	recombinase XerC
949309	953561	4252	OXZ34688.1	B9N54_04720	951587	952753	-1	aspartate aminotransferase
949309	953561	4252	OXZ34689.1	B9N54_04725	952740	953561	-1	metal-dependent phosphoesterase
1135201	1177204	42003	OXZ33900.1	B9N54_05575	1135201	1136436	-1	site-specific integrase
1135201	1177204	42003	OXZ33901.1	B9N54_05580	1136514	1136723	-1	excisionase
1135201	1177204	42003	OXZ33902.1	B9N54_05585	1137159	1137569	-1	RNA polymerase subunit sigma
1135201	1177204	42003	OXZ33903.1	B9N54_05590	1137791	1139212	-1	ABC transporter
1135201	1177204	42003	OXZ33910.1	B9N54_05595	1139200	1139889	-1	ABC transporter permease
1135201	1177204	42003	OXZ33904.1	B9N54_05600	1139882	1140472	-1	hypothetical protein
1135201	1177204	42003	OXZ33905.1	B9N54_05605	1140483	1142216	-1	multidrug ABC transporter ATP-binding protein
1135201	1177204	42003	OXZ33906.1	B9N54_05610	1142218	1143948	-1	ABC transporter ATP-binding protein
1135201	1177204	42003	OXZ33907.1	B9N54_05615	1144006	1144641	-1	TetR family transcriptional regulator
1135201	1177204	42003	OXZ33673.1	B9N54_05620	1146067	1146423	1	plasmid mobilization relaxosome protein MobC
1135201	1177204	42003	OXZ33674.1	B9N54_05625	1146425	1147488	1	endonuclease
1135201	1177204	42003	OXZ33647.1	B9N54_05630	1148554	1148883	1	relaxase
1135201	1177204	42003	OXZ33581.1	B9N54_05635	1148928	1149680	-1	ABC transporter permease
1135201	1177204	42003	OXZ33582.1	B9N54_05640	1149681	1150412	-1	ABC transporter permease
1135201	1177204	42003	OXZ33583.1	B9N54_05645	1150405	1151328	-1	bacitracin ABC transporter ATP-binding protein
1135201	1177204	42003	OXZ33584.1	B9N54_05650	1151343	1152110	-1	ABC transporter permease
1135201	1177204	42003	OXZ33585.1	B9N54_05655	1152230	1153138	-1	two-component sensor histidine kinase
1135201	1177204	42003	OXZ33586.1	B9N54_05660	1153142	1153840	-1	DNA-binding response regulator
1135201	1177204	42003	OXZ33587.1	B9N54_05665	1153861	1154073	-1	transcriptional regulator
1135201	1177204	42003	OXZ33588.1	B9N54_05670	1154397	1155056	-1	DNA-binding protein
1135201	1177204	42003	OXZ33589.1	B9N54_05675	1155100	1162743	-1	DNA helicase
1135201	1177204	42003	OXZ33590.1	B9N54_05680	1162730	1163695	-1	DNA (cytosine-5-)-methyltransferase
1135201	1177204	42003	OXZ33591.1	B9N54_05685	1163688	1165409	-1	DNA topoisomerase III
1135201	1177204	42003	OXZ33592.1	B9N54_05690	1165516	1166934	-1	AAA family ATPase
1135201	1177204	42003	OXZ33593.1	B9N54_05695	1167015	1167884	-1	bacteriocin
1135201	1177204	42003	OXZ33594.1	B9N54_05700	1167899	1169482	-1	copper amine oxidase
1135201	1177204	42003	OXZ33595.1	B9N54_05705	1169469	1169708	-1	hypothetical protein
1135201	1177204	42003	OXZ33596.1	B9N54_05710	1169723	1172302	-1	hypothetical protein
1135201	1177204	42003	OXZ33648.1	B9N54_05715	1172310	1174553	-1	conjugal transfer protein TraE
1135201	1177204	42003	OXZ33597.1	B9N54_05720	1174624	1175034	-1	conjugal transfer protein
1135201	1177204	42003	OXZ33598.1	B9N54_05725	1175036	1175326	-1	hypothetical protein
1135201	1177204	42003	OXZ33599.1	B9N54_05730	1175336	1176199	-1	hypothetical protein
1135201	1177204	42003	OXZ33600.1	B9N54_05735	1176210	1176425	-1	conjugal transfer protein
1135201	1177204	42003	OXZ33601.1	B9N54_05740	1176427	1176738	-1	single-stranded DNA-binding protein
1135201	1177204	42003	OXZ33602.1	B9N54_05745	1176929	1177204	-1	hypothetical protein
1136514	1146423	9909	OXZ33901.1	B9N54_05580	1136514	1136723	-1	excisionase
1136514	1146423	9909	OXZ33902.1	B9N54_05585	1137159	1137569	-1	RNA polymerase subunit sigma
1136514	1146423	9909	OXZ33903.1	B9N54_05590	1137791	1139212	-1	ABC transporter
1136514	1146423	9909	OXZ33910.1	B9N54_05595	1139200	1139889	-1	ABC transporter permease
1136514	1146423	9909	OXZ33904.1	B9N54_05600	1139882	1140472	-1	hypothetical protein
1136514	1146423	9909	OXZ33905.1	B9N54_05605	1140483	1142216	-1	multidrug ABC transporter ATP-binding protein
1136514	1146423	9909	OXZ33906.1	B9N54_05610	1142218	1143948	-1	ABC transporter ATP-binding protein
1136514	1146423	9909	OXZ33907.1	B9N54_05615	1144006	1144641	-1	TetR family transcriptional regulator
1136514	1146423	9909	OXZ33673.1	B9N54_05620	1146067	1146423	1	plasmid mobilization relaxosome protein MobC
1162730	1167884	5154	OXZ33589.1	B9N54_05675	1155100	1162743	-1	DNA helicase
1162730	1167884	5154	OXZ33590.1	B9N54_05680	1162730	1163695	-1	DNA (cytosine-5-)-methyltransferase
1162730	1167884	5154	OXZ33591.1	B9N54_05685	1163688	1165409	-1	DNA topoisomerase III
1162730	1167884	5154	OXZ33592.1	B9N54_05690	1165516	1166934	-1	AAA family ATPase

1162730	1167884	5154	OXZ33593.1	B9N54_05695	1167015	1167884	-1	bacteriocin
1185429	1198486	13057	OXZ33611.1	B9N54_05795	1185429	1187150	-1	ABC transporter ATP-binding protein
1185429	1198486	13057	OXZ33612.1	B9N54_05800	1187140	1188897	-1	ABC transporter ATP-binding protein
1185429	1198486	13057	OXZ33613.1	B9N54_05805	1188915	1190297	-1	ABC transporter ATP-binding protein
1185429	1198486	13057	OXZ33614.1	B9N54_05810	1190290	1191003	-1	cobalt transporter
1185429	1198486	13057	OXZ33615.1	B9N54_05815	1191003	1191599	-1	hypothetical protein
1185429	1198486	13057	OXZ33616.1	B9N54_05820	1191680	1192285	-1	TetR family transcriptional regulator
1185429	1198486	13057	OXZ33617.1	B9N54_05825	1192402	1194231	-1	recombinase TnpX
1185429	1198486	13057	OXZ33618.1	B9N54_05830	1194271	1195323	-1	conjugal transfer protein TraG
1185429	1198486	13057	OXZ33619.1	B9N54_05835	1195316	1196050	-1	phage antirepressor Ant
1185429	1198486	13057	OXZ33620.1	B9N54_05840	1196047	1196532	-1	conjugal transfer protein
1185429	1198486	13057	OXZ33621.1	B9N54_05845	1196525	1197352	-1	nucleoside triphosphate hydrolase
1185429	1198486	13057	OXZ33622.1	B9N54_05850	1197349	1198122	-1	replication initiator protein A
1185429	1198486	13057	OXZ33623.1	B9N54_05855	1198205	1198486	-1	hypothetical protein
1631909	1691704	59795	OXZ32456.1	B9N54_07905	1631909	1632202	-1	hypothetical protein
1631909	1691704	59795	OXZ32457.1	B9N54_07910	1632337	1632645	-1	exosortase
1631909	1691704	59795		B9N54_07915	1632651	1633321	-1	ATPase
1631909	1691704	59795	OXZ32458.1	B9N54_07920	1633701	1634243	-1	hypothetical protein
1631909	1691704	59795	OXZ32459.1	B9N54_07925	1634540	1636144	-1	macrolide ABC transporter ATP-binding protein
1631909	1691704	59795		B9N54_07930	1636624	1637011	-1	hypothetical protein
1631909	1691704	59795	OXZ32460.1	B9N54_07935	1637071	1637562	-1	protein-S-isoprenylcysteine methyltransferase
1631909	1691704	59795	OXZ32461.1	B9N54_07940	1637576	1638514	-1	esterase
1631909	1691704	59795	OXZ32462.1	B9N54_07945	1638583	1639209	-1	hypothetical protein
1631909	1691704	59795	OXZ32463.1	B9N54_07950	1639237	1639845	-1	SAM-dependent methyltransferase
1631909	1691704	59795	OXZ32464.1	B9N54_07955	1640060	1640809	-1	phosphorylase
1631909	1691704	59795	OXZ32465.1	B9N54_07960	1640944	1641558	-1	hypothetical protein
1631909	1691704	59795	OXZ32466.1	B9N54_07965	1641767	1643131	-1	ABC transporter ATP-binding protein
1631909	1691704	59795	OXZ32467.1	B9N54_07970	1643125	1643814	-1	cobalt ABC transporter permease
1631909	1691704	59795	OXZ32468.1	B9N54_07975	1643814	1644398	-1	hypothetical protein
1631909	1691704	59795	OXZ32469.1	B9N54_07980	1644458	1645801	-1	MATE family efflux transporter
1631909	1691704	59795	OXZ32470.1	B9N54_07985	1645813	1647534	-1	ABC transporter ATP-binding protein
1631909	1691704	59795	OXZ32471.1	B9N54_07990	1647518	1649272	-1	ABC transporter ATP-binding protein
1631909	1691704	59795	OXZ32472.1	B9N54_07995	1649433	1650425	1	AraC family transcriptional regulator
1631909	1691704	59795		B9N54_08000	1650854	1651616	-1	hypothetical protein
1631909	1691704	59795	OXZ32473.1	B9N54_08005	1651609	1653345	-1	multidrug ABC transporter ATP-binding protein
1631909	1691704	59795	OXZ32474.1	B9N54_08010	1653338	1655059	-1	ABC transporter ATP-binding protein
1631909	1691704	59795	OXZ32475.1	B9N54_08015	1655059	1656522	-1	ABC transporter
1631909	1691704	59795	OXZ32476.1	B9N54_08020	1656524	1657195	-1	cobalt transporter
1631909	1691704	59795	OXZ32477.1	B9N54_08025	1657192	1657782	-1	hypothetical protein
1631909	1691704	59795	OXZ32478.1	B9N54_08030	1657849	1658826	-1	AraC family transcriptional regulator
1631909	1691704	59795	OXZ32479.1	B9N54_08035	1658836	1659420	-1	TetR family transcriptional regulator
1631909	1691704	59795	OXZ32480.1	B9N54_08040	1659858	1660199	1	plasmid mobilization relaxosome protein MobC
1631909	1691704	59795	OXZ32481.1	B9N54_08045	1660574	1662907	-1	peptidase S8
1631909	1691704	59795	OXZ32482.1	B9N54_08050	1662910	1664007	-1	ATPase
1631909	1691704	59795	OXZ32483.1	B9N54_08055	1664488	1664934	-1	abortive infection protein
1631909	1691704	59795	OXZ32484.1	B9N54_08060	1665018	1665866	-1	AraC family transcriptional regulator
1631909	1691704	59795		B9N54_08065	1666434	1667498	-1	23S rRNA (uracil(1939)-C(5))-methyltransferase RlmD
1631909	1691704	59795	OXZ32485.1	B9N54_08070	1667999	1669765	-1	hypothetical protein
1631909	1691704	59795	OXZ32493.1	B9N54_08075	1669780	1670097	-1	nucleotide pyrophosphohydrolase
1631909	1691704	59795	OXZ32486.1	B9N54_08080	1670110	1671900	-1	hypothetical protein
1631909	1691704	59795		B9N54_08085	1672610	1673406	-1	lysophospholipase
1631909	1691704	59795	OXZ32487.1	B9N54_08090	1673763	1674020	-1	hypothetical protein
1631909	1691704	59795	OXZ32488.1	B9N54_08095	1674041	1676476	-1	ABC transporter permease
1631909	1691704	59795	OXZ32489.1	B9N54_08100	1676469	1677149	-1	peptide ABC transporter ATP-binding protein
1631909	1691704	59795	OXZ32490.1	B9N54_08105	1677202	1678062	-1	two-component sensor histidine kinase
1631909	1691704	59795	OXZ32491.1	B9N54_08110	1678063	1678713	-1	DNA-binding response regulator
1631909	1691704	59795	OXZ32492.1	B9N54_08115	1678899	1679023	1	transposase
1631909	1691704	59795	OXZ32171.1	B9N54_08120	1680024	1680187	1	hypothetical protein
1631909	1691704	59795	OXZ32172.1	B9N54_08125	1680267	1682039	-1	recombinase family protein
1631909	1691704	59795	OXZ32173.1	B9N54_08130	1682620	1684011	-1	ABC transporter
1631909	1691704	59795	OXZ32174.1	B9N54_08135	1684008	1684736	-1	transporter
1631909	1691704	59795	OXZ32175.1	B9N54_08140	1684736	1685326	-1	hypothetical protein
1631909	1691704	59795	OXZ32176.1	B9N54_08145	1685352	1687076	-1	ABC transporter permease
1631909	1691704	59795	OXZ32177.1	B9N54_08150	1687073	1688842	-1	ABC transporter permease
1631909	1691704	59795	OXZ32178.1	B9N54_08155	1688950	1689564	-1	TetR family transcriptional regulator
1631909	1691704	59795	OXZ32179.1	B9N54_08160	1689968	1691704	-1	multidrug ABC transporter ATP-binding protein
1631909	1691704	59795	OXZ32180.1	B9N54_08165	1691697	1693415	-1	ABC transporter ATP-binding protein
1653338	1665866	12528	OXZ32473.1	B9N54_08005	1651609	1653345	-1	multidrug ABC transporter ATP-binding protein
1653338	1665866	12528	OXZ32474.1	B9N54_08010	1653338	1655059	-1	ABC transporter ATP-binding protein
1653338	1665866	12528	OXZ32475.1	B9N54_08015	1655059	1656522	-1	ABC transporter
1653338	1665866	12528	OXZ32476.1	B9N54_08020	1656524	1657195	-1	cobalt transporter
1653338	1665866	12528	OXZ32477.1	B9N54_08025	1657192	1657782	-1	hypothetical protein
1653338	1665866	12528	OXZ32478.1	B9N54_08030	1657849	1658826	-1	AraC family transcriptional regulator
1653338	1665866	12528	OXZ32479.1	B9N54_08035	1658836	1659420	-1	TetR family transcriptional regulator
1653338	1665866	12528	OXZ32480.1	B9N54_08040	1659858	1660199	1	plasmid mobilization relaxosome protein MobC
1653338	1665866	12528	OXZ32481.1	B9N54_08045	1660574	1662907	-1	peptidase S8
1653338	1665866	12528	OXZ32482.1	B9N54_08050	1662910	1664007	-1	ATPase
1653338	1665866	12528	OXZ32483.1	B9N54_08055	1664488	1664934	-1	abortive infection protein
1653338	1665866	12528	OXZ32484.1	B9N54_08060	1665018	1665866	-1	AraC family transcriptional regulator
1722538	1824911	102373	OXZ32116.1	B9N54_08255	1720719	1722554	-1	multidrug ABC transporter
1722538	1824911	102373	OXZ32117.1	B9N54_08260	1722538	1724340	-1	multidrug ABC transporter
1722538	1824911	102373	OXZ32118.1	B9N54_08265	1724420	1725085	-1	histidine kinase

1722538	1824911	102373	OXZ32119.1	B9N54_08270	1725093	1725788	-1	DNA-binding response regulator
1722538	1824911	102373	OXZ32120.1	B9N54_08275	1725872	1726735	-1	UTP--glucose-1-phosphate uridylyltransferase
1722538	1824911	102373	OXZ32121.1	B9N54_08280	1727463	1730048	-1	ATP-dependent chaperone ClpB
1722538	1824911	102373	OXZ32122.1	B9N54_08285	1730067	1731011	-1	heat-shock protein
1722538	1824911	102373	OXZ32123.1	B9N54_08290	1731172	1731837	-1	nitroreductase
1722538	1824911	102373	OXZ32124.1	B9N54_08295	1731989	1732528	-1	RNA polymerase subunit sigma
1722538	1824911	102373	OXZ32125.1	B9N54_08300	1732767	1733222	1	hypothetical protein
1722538	1824911	102373	OXZ32126.1	B9N54_08305	1733323	1737573	-1	hypothetical protein
1722538	1824911	102373		B9N54_08310	1738533	1738844	-1	hypothetical protein
1722538	1824911	102373	OXZ32127.1	B9N54_08315	1738878	1739078	-1	hypothetical protein
1722538	1824911	102373	OXZ32128.1	B9N54_08320	1739665	1740216	1	hypothetical protein
1722538	1824911	102373	OXZ32129.1	B9N54_08325	1740277	1740489	1	hypothetical protein
1722538	1824911	102373	OXZ32130.1	B9N54_08330	1740569	1741717	1	MFS transporter
1722538	1824911	102373	OXZ32131.1	B9N54_08335	1741802	1742299	-1	hypothetical protein
1722538	1824911	102373	OXZ32132.1	B9N54_08340	1742333	1742806	-1	peptidase M50
1722538	1824911	102373	OXZ32133.1	B9N54_08345	1743311	1744294	-1	albumin-binding protein
1722538	1824911	102373	OXZ32134.1	B9N54_08350	1744957	1745379	-1	hypothetical protein
1722538	1824911	102373	OXZ32135.1	B9N54_08355	1745424	1746359	-1	TIGR01212 family radical SAM protein
1722538	1824911	102373	OXZ32136.1	B9N54_08360	1746352	1746792	-1	peroxiredoxin
1722538	1824911	102373	OXZ32137.1	B9N54_08365	1746785	1747621	-1	hydrolase
1722538	1824911	102373	OXZ32138.1	B9N54_08370	1747611	1747934	-1	DUF1292 domain-containing protein
1722538	1824911	102373	OXZ32139.1	B9N54_08375	1747949	1749340	-1	aminoacyl-histidine dipeptidase
1722538	1824911	102373	OXZ32140.1	B9N54_08380	1749405	1750523	-1	hypothetical protein
1722538	1824911	102373	OXZ32141.1	B9N54_08385	1750577	1751566	-1	peptide chain release factor 2
1722538	1824911	102373	OXZ32142.1	B9N54_08390	1751598	1751777	-1	hypothetical protein
1722538	1824911	102373	OXZ32143.1	B9N54_08395	1752103	1754832	-1	preprotein translocase subunit SecA
1722538	1824911	102373	OXZ32144.1	B9N54_08400	1755433	1756143	-1	transaldolase
1722538	1824911	102373	OXZ32145.1	B9N54_08405	1756171	1757529	-1	PTS ascorbate transporter subunit IIC
1722538	1824911	102373	OXZ32146.1	B9N54_08410	1757547	1757822	-1	PTS maltose transporter subunit IIBC
1722538	1824911	102373	OXZ32147.1	B9N54_08415	1757815	1758285	-1	PTS sugar transporter subunit IIA
1722538	1824911	102373	OXZ32148.1	B9N54_08420	1758393	1759310	-1	malate transporter
1722538	1824911	102373	OXZ32149.1	B9N54_08425	1759819	1761279	-1	peptide-binding protein
1722538	1824911	102373	OXZ32150.1	B9N54_08430	1761468	1762613	-1	hypothetical protein
1722538	1824911	102373	OXZ32151.1	B9N54_08435	1763296	1764732	-1	UDP-N-acetylmuramoyl-L-alanyl-D-glutamate--2, 6-diaminopimelate ligase
1722538	1824911	102373	OXZ32152.1	B9N54_08440	1764988	1765842	-1	beta-carotene 15,15'-monooxygenase
1722538	1824911	102373	OXZ32153.1	B9N54_08445	1765862	1766728	-1	polyphosphate--nucleotide phosphotransferase
1722538	1824911	102373	OXZ32154.1	B9N54_08450	1766927	1767913	1	alkane 1-monooxygenase
1722538	1824911	102373	OXZ32155.1	B9N54_08455	1768168	1769046	-1	ribokinase
1722538	1824911	102373	OXZ32156.1	B9N54_08460	1769195	1773166	-1	hypothetical protein
1722538	1824911	102373	OXZ32157.1	B9N54_08465	1773446	1779070	-1	hypothetical protein
1722538	1824911	102373	OXZ32158.1	B9N54_08470	1779344	1780198	1	hypothetical protein
1722538	1824911	102373	OXZ32159.1	B9N54_08475	1780318	1788978	-1	hypothetical protein
1722538	1824911	102373	OXZ32106.1	B9N54_08480	1790559	1791107	1	hypothetical protein
1722538	1824911	102373	OXZ32107.1	B9N54_08485	1791143	1792447	1	N-acetylmuramoyl-L-alanine amidase
1722538	1824911	102373	OXZ32108.1	B9N54_08490	1792594	1792701	1	transposase
1722538	1824911	102373	OXZ31954.1	B9N54_08495	1793975	1794298	-1	hypothetical protein
1722538	1824911	102373	OXZ31955.1	B9N54_08500	1794364	1794858	-1	hypothetical protein
1722538	1824911	102373	OXZ31956.1	B9N54_08505	1794926	1795753	-1	hypothetical protein
1722538	1824911	102373	OXZ31957.1	B9N54_08510	1795918	1796565	1	sugar ABC transporter ATP-binding protein
1722538	1824911	102373	OXZ31958.1	B9N54_08515	1796549	1797193	1	sugar ABC transporter permease
1722538	1824911	102373	OXZ31959.1	B9N54_08520	1797194	1797712	1	hypothetical protein
1722538	1824911	102373	OXZ31857.1	B9N54_08525	1799044	1799571	1	hypothetical protein
1722538	1824911	102373	OXZ31919.1	B9N54_08530	1800427	1803114	-1	calcium-translocating P-type ATPase, SERCA-type
1722538	1824911	102373	OXZ31858.1	B9N54_08535	1803284	1804042	-1	metal-dependent hydrolase
1722538	1824911	102373	OXZ31859.1	B9N54_08540	1804168	1804638	1	aminoacyl-tRNA deacylase
1722538	1824911	102373	OXZ31860.1	B9N54_08545	1804641	1805945	1	glutamate--cysteine ligase
1722538	1824911	102373	OXZ31861.1	B9N54_08550	1805938	1807248	1	glutathionylspermidine synthase
1722538	1824911	102373	OXZ31862.1	B9N54_08555	1807345	1807614	-1	Txe/YoeB family addiction module toxin
1722538	1824911	102373	OXZ31863.1	B9N54_08560	1807608	1807856	-1	prevent-host-death protein
1722538	1824911	102373	OXZ31864.1	B9N54_08565	1808039	1808458	1	CMP deaminase
1722538	1824911	102373	OXZ31865.1	B9N54_08570	1808470	1811889	1	pyruvate carboxylase
1722538	1824911	102373	OXZ31866.1	B9N54_08575	1811926	1812330	-1	DUF3139 domain-containing protein
1722538	1824911	102373	OXZ31867.1	B9N54_08580	1812461	1812703	1	cell division protein FtsW
1722538	1824911	102373	OXZ31868.1	B9N54_08585	1812806	1813213	1	hypothetical protein
1722538	1824911	102373	OXZ31869.1	B9N54_08590	1813229	1813735	-1	thiol reductase thioredoxin
1722538	1824911	102373	OXZ31870.1	B9N54_08595	1813840	1814184	-1	hypothetical protein
1722538	1824911	102373	OXZ31871.1	B9N54_08600	1814249	1814938	-1	hypothetical protein
1722538	1824911	102373	OXZ31872.1	B9N54_08605	1814935	1819629	-1	FMN-binding domain-containing protein
1722538	1824911	102373	OXZ31920.1	B9N54_08610	1819874	1820827	-1	2-dehydropantoate 2-reductase
1722538	1824911	102373	OXZ31873.1	B9N54_08615	1820847	1822496	-1	inorganic pyrophosphatase
1722538	1824911	102373	OXZ31874.1	B9N54_08620	1822661	1823182	1	N-carbamoylsarcosine amidohydrolase
1722538	1824911	102373		B9N54_08625	1823405	1823742	-1	RNA helicase
1722538	1824911	102373	OXZ31875.1	B9N54_08630	1824068	1824496	-1	hypothetical protein
1722538	1824911	102373	OXZ31876.1	B9N54_08635	1824555	1824911	-1	hypothetical protein
1867992	1874956	6964	OXZ31643.1	B9N54_08875	1867992	1868531	1	transposase
1867992	1874956	6964	OXZ31644.1	B9N54_08880	1868726	1869351	1	IS3 family transposase
1867992	1874956	6964	OXZ31452.1	B9N54_08900	1872118	1872655	-1	IS110 family transposase
1867992	1874956	6964	OXZ31645.1	B9N54_08870	1873742	1874956	1	IS256 family transposase

Table S3H: Predicted genomic islands in the genome of *"F. nericia"* CCUG54800
Shown are genes of genomic islands predicted by IslandViewer4

Island start	Island end	Length	Gene name	Locus	Gene start	Gene end	Strand	Product
68253	77535	9282	OXZ29102.1	B9N49_00325	68253	69122	1	hypothetical protein
68253	77535	9282	OXZ29103.1	B9N49_00330	69109	71097	1	exopolyphosphatase
68253	77535	9282	OXZ29104.1	B9N49_00335	71078	71524	1	50S ribosomal protein L9
68253	77535	9282	OXZ29105.1	B9N49_00340	71558	72895	1	replicative DNA helicase
68253	77535	9282	OXZ29106.1	B9N49_00345	72892	73455	1	GNAT family N-acetyltransferase
68253	77535	9282	OXZ29107.1	B9N49_00350	73460	74218	1	DNA replication protein
68253	77535	9282	OXZ29108.1	B9N49_00355	74221	75198	1	DNA replication protein DnaC
68253	77535	9282	OXZ29109.1	B9N49_00360	75195	75839	1	NAD(P)H-hydrate epimerase
68253	77535	9282	OXZ29110.1	B9N49_00365	76323	76865	1	ECF transporter S component
68253	77535	9282	OXZ29111.1	B9N49_00370	76849	77535	1	radical SAM protein
1015853	1107796	91943	OXZ27717.1	B9N49_05145	1015853	1016062	-1	excisionase
1015853	1107796	91943	OXZ27718.1	B9N49_05150	1016498	1016908	-1	RNA polymerase subunit sigma
1015853	1107796	91943	OXZ27361.1	B9N49_05155	1018276	1018815	1	transposase
1015853	1107796	91943	OXZ27362.1	B9N49_05160	1018803	1019684	1	hypothetical protein
1015853	1107796	91943	OXZ27348.1	B9N49_05165	1020784	1021207	-1	MATE family efflux transporter
1015853	1107796	91943	OXZ27349.1	B9N49_05170	1021249	1022712	-1	ABC transporter
1015853	1107796	91943	OXZ27350.1	B9N49_05175	1022715	1023449	-1	transporter
1015853	1107796	91943	OXZ27351.1	B9N49_05180	1023449	1024030	-1	hypothetical protein
1015853	1107796	91943	OXZ27352.1	B9N49_05185	1024111	1025844	-1	multidrug ABC transporter ATP-binding protein
1015853	1107796	91943	OXZ27353.1	B9N49_05190	1025838	1027583	-1	ABC transporter ATP-binding protein
1015853	1107796	91943	OXZ27354.1	B9N49_05195	1027677	1028300	-1	TetR family transcriptional regulator
1015853	1107796	91943	OXZ27355.1	B9N49_05200	1028609	1028965	1	plasmid mobilization relaxosome protein MobC
1015853	1107796	91943	OXZ27356.1	B9N49_05205	1028967	1030298	1	endonuclease
1015853	1107796	91943	OXZ27357.1	B9N49_05210	1030454	1031110	-1	DNA-binding protein
1015853	1107796	91943		B9N49_05215	1031154	1038797	-1	DNA helicase
1015853	1107796	91943	OXZ27360.1	B9N49_05220	1038882	1040384	-1	SIR2 family protein
1015853	1107796	91943	OXZ27358.1	B9N49_05225	1040441	1041379	-1	DNA (cytosine-5-)-methyltransferase
1015853	1107796	91943	OXZ27359.1	B9N49_05230	1041372	1043093	-1	DNA topoisomerase III
1015853	1107796	91943	OXZ27256.1	B9N49_05235	1044146	1044516	-1	thioesterase
1015853	1107796	91943	OXZ27257.1	B9N49_05240	1044673	1045044	-1	transcriptional regulator
1015853	1107796	91943		B9N49_05245	1045511	1045651	-1	diacylglyceryl transferase
1015853	1107796	91943	OXZ27258.1	B9N49_05250	1045700	1046068	1	plasmid mobilization relaxosome protein MobC
1015853	1107796	91943	OXZ27259.1	B9N49_05255	1046068	1047438	1	endonuclease
1015853	1107796	91943	OXZ27260.1	B9N49_05260	1047599	1048474	-1	single-stranded DNA-binding protein
1015853	1107796	91943	OXZ27261.1	B9N49_05265	1048775	1059184	-1	conjugal transfer protein
1015853	1107796	91943	OXZ27262.1	B9N49_05270	1059379	1059819	-1	terminase large subunit
1015853	1107796	91943	OXZ27263.1	B9N49_05275	1060047	1061753	-1	DNA topoisomerase III
1015853	1107796	91943	OXZ27264.1	B9N49_05280	1062170	1063048	-1	bacteriocin
1015853	1107796	91943	OXZ27265.1	B9N49_05285	1063064	1065418	-1	copper amine oxidase
1015853	1107796	91943	OXZ27266.1	B9N49_05290	1065415	1065672	-1	hypothetical protein
1015853	1107796	91943	OXZ27267.1	B9N49_05295	1065692	1068655	-1	hypothetical protein
1015853	1107796	91943	OXZ27268.1	B9N49_05300	1068665	1069630	-1	site-specific DNA-methyltransferase
1015853	1107796	91943	OXZ27269.1	B9N49_05305	1069602	1072097	-1	conjugal transfer protein TraE
1015853	1107796	91943		B9N49_05310	1072094	1072288	-1	conjugal transfer protein
1015853	1107796	91943	OXZ27270.1	B9N49_05315	1072332	1072470	1	transposase
1015853	1107796	91943	OXZ27234.1	B9N49_05320	1073471	1073609	-1	transposase
1015853	1107796	91943	OXZ27235.1	B9N49_05325	1073690	1075696	-1	hypothetical protein
1015853	1107796	91943	OXZ27236.1	B9N49_05330	1075697	1076452	-1	bacitracin ABC transporter ATP-binding protein
1015853	1107796	91943	OXZ27237.1	B9N49_05335	1076436	1078589	-1	lantibiotic transporter
1015853	1107796	91943	OXZ27238.1	B9N49_05340	1078573	1080699	-1	hypothetical protein
1015853	1107796	91943	OXZ27239.1	B9N49_05345	1080671	1081477	-1	hypothetical protein
1015853	1107796	91943	OXZ27240.1	B9N49_05350	1081750	1082406	-1	DNA-binding protein
1015853	1107796	91943	OXZ27241.1	B9N49_05355	1082450	1090093	-1	DNA helicase
1015853	1107796	91943	OXZ27242.1	B9N49_05360	1090176	1092389	-1	hypothetical protein
1015853	1107796	91943	OXZ27243.1	B9N49_05365	1092425	1093387	-1	DNA (cytosine-5-)-methyltransferase
1015853	1107796	91943	OXZ27244.1	B9N49_05370	1093380	1095101	-1	DNA topoisomerase III
1015853	1107796	91943	OXZ27245.1	B9N49_05375	1095190	1096173	-1	hypothetical protein
1015853	1107796	91943	OXZ27246.1	B9N49_05380	1096166	1096777	-1	hypothetical protein
1015853	1107796	91943	OXZ27247.1	B9N49_05385	1096851	1097711	-1	bacteriocin
1015853	1107796	91943	OXZ27248.1	B9N49_05390	1097726	1099309	-1	copper amine oxidase
1015853	1107796	91943	OXZ27249.1	B9N49_05395	1099296	1099535	-1	hypothetical protein
1015853	1107796	91943	OXZ27250.1	B9N49_05400	1099550	1102129	-1	hypothetical protein
1015853	1107796	91943	OXZ27251.1	B9N49_05405	1102137	1104560	-1	conjugal transfer protein TraE
1015853	1107796	91943	OXZ27252.1	B9N49_05410	1104451	1104861	-1	conjugal transfer protein
1015853	1107796	91943	OXZ27253.1	B9N49_05415	1104863	1105153	-1	hypothetical protein
1015853	1107796	91943	OXZ27254.1	B9N49_05420	1105163	1106026	-1	hypothetical protein
1015853	1107796	91943	OXZ27255.1	B9N49_05425	1106037	1106183	-1	hypothetical protein
1015853	1107796	91943	OXZ27208.1	B9N49_05430	1107352	1107483	-1	hypothetical protein
1015853	1107796	91943	OXZ27209.1	B9N49_05435	1107485	1107796	-1	single-stranded DNA-binding protein
1038882	1068655	29773	OXZ27360.1	B9N49_05220	1038882	1040384	-1	SIR2 family protein
1038882	1068655	29773	OXZ27358.1	B9N49_05225	1040441	1041379	-1	DNA (cytosine-5-)-methyltransferase
1038882	1068655	29773	OXZ27359.1	B9N49_05230	1041372	1043093	-1	DNA topoisomerase III
1038882	1068655	29773	OXZ27256.1	B9N49_05235	1044146	1044516	-1	thioesterase
1038882	1068655	29773	OXZ27257.1	B9N49_05240	1044673	1045044	-1	transcriptional regulator
1038882	1068655	29773		B9N49_05245	1045511	1045651	-1	diacylglyceryl transferase
1038882	1068655	29773	OXZ27258.1	B9N49_05250	1045700	1046068	1	plasmid mobilization relaxosome protein MobC
1038882	1068655	29773	OXZ27259.1	B9N49_05255	1046068	1047438	1	endonuclease
1038882	1068655	29773	OXZ27260.1	B9N49_05260	1047599	1048474	-1	single-stranded DNA-binding protein
1038882	1068655	29773	OXZ27261.1	B9N49_05265	1048775	1059184	-1	conjugal transfer protein
1038882	1068655	29773	OXZ27262.1	B9N49_05270	1059379	1059819	-1	terminase large subunit
1038882	1068655	29773	OXZ27263.1	B9N49_05275	1060047	1061753	-1	DNA topoisomerase III
1038882	1068655	29773	OXZ27264.1	B9N49_05280	1062170	1063048	-1	bacteriocin

1038882	1068655	29773	OXZ27265.1	B9N49_05285	1063064	1065418	-1	copper amine oxidase
1038882	1068655	29773	OXZ27266.1	B9N49_05290	1065415	1065672	-1	hypothetical protein
1038882	1068655	29773	OXZ27267.1	B9N49_05295	1065692	1068655	-1	hypothetical protein
1073690	1082406	8716	OXZ27235.1	B9N49_05325	1073690	1075696	-1	hypothetical protein
1073690	1082406	8716	OXZ27236.1	B9N49_05330	1075697	1076452	-1	bacitracin ABC transporter ATP-binding protein
1073690	1082406	8716	OXZ27237.1	B9N49_05335	1076436	1078589	-1	lantibiotic transporter
1073690	1082406	8716	OXZ27238.1	B9N49_05340	1078573	1080699	-1	hypothetical protein
1073690	1082406	8716	OXZ27239.1	B9N49_05345	1080671	1081477	-1	hypothetical protein
1073690	1082406	8716	OXZ27240.1	B9N49_05350	1081750	1082406	-1	DNA-binding protein
1090176	1097711	7535	OXZ27242.1	B9N49_05360	1090176	1092389	-1	hypothetical protein
1090176	1097711	7535	OXZ27243.1	B9N49_05365	1092425	1093387	-1	DNA (cytosine-5-)-methyltransferase
1090176	1097711	7535	OXZ27244.1	B9N49_05370	1093380	1095101	-1	DNA topoisomerase III
1090176	1097711	7535	OXZ27245.1	B9N49_05375	1095190	1096173	-1	hypothetical protein
1090176	1097711	7535	OXZ27246.1	B9N49_05380	1096166	1096777	-1	hypothetical protein
1090176	1097711	7535	OXZ27247.1	B9N49_05385	1096851	1097711	-1	bacteriocin
1107485	1112210	4725	OXZ27209.1	B9N49_05435	1107485	1107796	-1	single-stranded DNA-binding protein
1107485	1112210	4725		B9N49_05440	1107992	1109461	-1	hypothetical protein
1107485	1112210	4725	OXZ27210.1	B9N49_05445	1109479	1110015	-1	ClbS/DfsB family four-helix bundle protein
1107485	1112210	4725	OXZ27211.1	B9N49_05450	1110077	1110856	-1	conjugal transfer protein TraG
1107485	1112210	4725	OXZ27175.1	B9N49_05455	1112025	1112210	-1	hypothetical protein
1116939	1128106	11167	OXZ27181.1	B9N49_05485	1116939	1117214	-1	hypothetical protein
1116939	1128106	11167	OXZ27182.1	B9N49_05490	1117268	1118815	-1	hypothetical protein
1116939	1128106	11167	OXZ27183.1	B9N49_05495	1118812	1119522	-1	ABC transporter
1116939	1128106	11167	OXZ27184.1	B9N49_05500	1119589	1119897	-1	lysis protein
1116939	1128106	11167	OXZ27185.1	B9N49_05505	1119878	1120477	-1	hypothetical protein
1116939	1128106	11167	OXZ27186.1	B9N49_05510	1120467	1120742	-1	transcriptional regulator
1116939	1128106	11167	OXZ27187.1	B9N49_05515	1120873	1122723	-1	recombinase TnpX
1116939	1128106	11167	OXZ27034.1	B9N49_05520	1123890	1124942	-1	conjugal transfer protein TraG
1116939	1128106	11167	OXZ27035.1	B9N49_05525	1124935	1125669	-1	phage antirepressor Ant
1116939	1128106	11167	OXZ27036.1	B9N49_05530	1125666	1126151	-1	conjugal transfer protein
1116939	1128106	11167	OXZ27037.1	B9N49_05535	1126144	1126971	-1	nucleoside triphosphate hydrolase
1116939	1128106	11167	OXZ27038.1	B9N49_05540	1126968	1127741	-1	replication initiator protein A
1116939	1128106	11167	OXZ27039.1	B9N49_05545	1127825	1128106	-1	hypothetical protein
1464214	1499122	34908	OXZ26914.1	B9N49_07075	1464214	1465734	-1	conjugal transfer protein
1464214	1499122	34908	OXZ26915.1	B9N49_07080	1465727	1467469	-1	recombinase
1464214	1499122	34908	OXZ26916.1	B9N49_07085	1467462	1469180	-1	recombinase
1464214	1499122	34908	OXZ26917.1	B9N49_07090	1469279	1469446	-1	aminoacyl-tRNA hydrolase
1464214	1499122	34908	OXZ26918.1	B9N49_07095	1469899	1470372	-1	RNA polymerase subunit sigma
1464214	1499122	34908	OXZ26919.1	B9N49_07100	1471116	1472459	-1	MATE family efflux transporter
1464214	1499122	34908	OXZ26920.1	B9N49_07105	1472491	1474236	-1	ABC transporter permease
1464214	1499122	34908	OXZ26921.1	B9N49_07110	1474229	1476019	-1	ABC transporter ATP-binding protein
1464214	1499122	34908	OXZ26922.1	B9N49_07115	1476009	1477475	-1	ABC transporter
1464214	1499122	34908	OXZ26923.1	B9N49_07120	1477460	1478161	-1	ABC transporter permease
1464214	1499122	34908	OXZ26924.1	B9N49_07125	1478164	1478760	-1	hypothetical protein
1464214	1499122	34908	OXZ26925.1	B9N49_07130	1478902	1479540	-1	TetR family transcriptional regulator
1464214	1499122	34908	OXZ26926.1	B9N49_07135	1479648	1480358	-1	hypothetical protein
1464214	1499122	34908	OXZ26927.1	B9N49_07140	1480375	1481460	-1	hypothetical protein
1464214	1499122	34908	OXZ26928.1	B9N49_07145	1481471	1482445	-1	hypothetical protein
1464214	1499122	34908	OXZ26929.1	B9N49_07150	1482438	1487345	-1	hypothetical protein
1464214	1499122	34908	OXZ26930.1	B9N49_07155	1487345	1487614	-1	hypothetical protein
1464214	1499122	34908	OXZ26931.1	B9N49_07160	1487619	1488635	-1	hypothetical protein
1464214	1499122	34908	OXZ26932.1	B9N49_07165	1488653	1490071	-1	hypothetical protein
1464214	1499122	34908	OXZ26933.1	B9N49_07170	1490082	1493816	-1	hypothetical protein
1464214	1499122	34908	OXZ26780.1	B9N49_07175	1494883	1495941	-1	conjugal transfer protein TraG
1464214	1499122	34908	OXZ26781.1	B9N49_07180	1495986	1496192	-1	hypothetical protein
1464214	1499122	34908	OXZ26782.1	B9N49_07185	1496390	1496881	-1	hypothetical protein
1464214	1499122	34908	OXZ26783.1	B9N49_07190	1496896	1497753	-1	DNA replication protein
1464214	1499122	34908	OXZ26784.1	B9N49_07195	1497768	1498745	-1	replication initiator RepA
1464214	1499122	34908	OXZ26785.1	B9N49_07200	1498832	1499122	-1	hypothetical protein
1487619	1499122	11503	OXZ26931.1	B9N49_07160	1487619	1488635	-1	hypothetical protein
1487619	1499122	11503	OXZ26932.1	B9N49_07165	1488653	1490071	-1	hypothetical protein
1487619	1499122	11503	OXZ26933.1	B9N49_07170	1490082	1493816	-1	hypothetical protein
1487619	1499122	11503	OXZ26780.1	B9N49_07175	1494883	1495941	-1	conjugal transfer protein TraG
1487619	1499122	11503	OXZ26781.1	B9N49_07180	1495986	1496192	-1	hypothetical protein
1487619	1499122	11503	OXZ26782.1	B9N49_07185	1496390	1496881	-1	hypothetical protein
1487619	1499122	11503	OXZ26783.1	B9N49_07190	1496896	1497753	-1	DNA replication protein
1487619	1499122	11503	OXZ26784.1	B9N49_07195	1497768	1498745	-1	replication initiator RepA
1487619	1499122	11503	OXZ26785.1	B9N49_07200	1498832	1499122	-1	hypothetical protein
1535913	1544549	8636	OXZ26766.1	B9N49_07380	1535913	1536200	-1	cupin
1535913	1544549	8636	OXZ26767.1	B9N49_07385	1536268	1536840	-1	uracil-DNA glycosylase
1535913	1544549	8636	OXZ26768.1	B9N49_07390	1537611	1538900	-1	hypothetical protein
1535913	1544549	8636	OXZ26769.1	B9N49_07395	1539274	1540941	-1	DNA mismatch repair protein
1535913	1544549	8636	OXZ26770.1	B9N49_07400	1540951	1541985	-1	DNA (cytosine-5-)-methyltransferase
1535913	1544549	8636	OXZ26771.1	B9N49_07405	1542042	1542998	-1	restriction endonuclease
1535913	1544549	8636	OXZ26772.1	B9N49_07410	1543008	1544030	-1	DNA (cytosine-5-)-methyltransferase
1535913	1544549	8636	OXZ26773.1	B9N49_07415	1544017	1544253	-1	DNA-binding protein
1535913	1544549	8636	OXZ26774.1	B9N49_07420	1544361	1544549	-1	hypothetical protein
1612738	1655078	42340	OXZ26698.1	B9N49_07720	1612738	1613161	-1	MATE family efflux transporter
1612738	1655078	42340	OXZ26699.1	B9N49_07725	1613183	1614928	-1	ABC transporter ATP-binding protein
1612738	1655078	42340	OXZ26700.1	B9N49_07730	1614929	1616641	-1	ABC transporter ATP-binding protein
1612738	1655078	42340	OXZ26701.1	B9N49_07735	1616658	1618166	-1	ABC transporter ATP-binding protein
1612738	1655078	42340	OXZ26702.1	B9N49_07740	1618154	1618828	-1	hypothetical protein
1612738	1655078	42340	OXZ26703.1	B9N49_07745	1618846	1619448	-1	hypothetical protein
1612738	1655078	42340	OXZ26707.1	B9N49_07750	1619590	1620561	-1	AraC family transcriptional regulator
1612738	1655078	42340	OXZ26704.1	B9N49_07755	1620591	1621352	-1	alpha/beta hydrolase
1612738	1655078	42340	OXZ26705.1	B9N49_07760	1621336	1622415	-1	semialdehyde dehydrogenase
1612738	1655078	42340	OXZ26706.1	B9N49_07765	1622464	1627974	-1	non-ribosomal peptide synthetase

1612738	1655078	42340	OXZ26708.1	B9N49_07770	1627994	1629048	-1	hypothetical protein
1612738	1655078	42340	OXZ26697.1	B9N49_07775	1630103	1631170	-1	MATE family efflux transporter
1612738	1655078	42340	OXZ26589.1	B9N49_07780	1632171	1632409	1	IS30 family transposase
1612738	1655078	42340	OXZ26590.1	B9N49_07785	1632518	1633267	-1	phosphorylase
1612738	1655078	42340	OXZ26591.1	B9N49_07790	1633527	1635131	-1	macrolide ABC transporter ATP-binding protein
1612738	1655078	42340		B9N49_07795	1635454	1635747	-1	transposase
1612738	1655078	42340	OXZ26592.1	B9N49_07800	1635859	1636473	-1	hypothetical protein
1612738	1655078	42340	OXZ26593.1	B9N49_07805	1636678	1638042	-1	ABC transporter ATP-binding protein
1612738	1655078	42340	OXZ26594.1	B9N49_07810	1638030	1638725	-1	cobalt ABC transporter permease
1612738	1655078	42340	OXZ26595.1	B9N49_07815	1638725	1639309	-1	hypothetical protein
1612738	1655078	42340	OXZ26596.1	B9N49_07820	1639369	1640712	-1	MATE family efflux transporter
1612738	1655078	42340	OXZ26597.1	B9N49_07825	1640724	1642445	-1	ABC transporter ATP-binding protein
1612738	1655078	42340	OXZ26598.1	B9N49_07830	1642429	1644183	-1	ABC transporter ATP-binding protein
1612738	1655078	42340		B9N49_07835	1644344	1645335	1	AraC family transcriptional regulator
1612738	1655078	42340		B9N49_07840	1645700	1646460	-1	hypothetical protein
1612738	1655078	42340	OXZ26599.1	B9N49_07845	1646453	1648189	-1	multidrug ABC transporter ATP-binding protein
1612738	1655078	42340	OXZ26600.1	B9N49_07850	1648182	1649903	-1	ABC transporter ATP-binding protein
1612738	1655078	42340	OXZ26601.1	B9N49_07855	1649903	1651366	-1	ABC transporter
1612738	1655078	42340	OXZ26602.1	B9N49_07860	1651368	1652039	-1	cobalt transporter
1612738	1655078	42340	OXZ26603.1	B9N49_07865	1652036	1652626	-1	hypothetical protein
1612738	1655078	42340	OXZ26604.1	B9N49_07870	1652693	1653670	-1	AraC family transcriptional regulator
1612738	1655078	42340	OXZ26605.1	B9N49_07875	1653680	1654264	-1	TetR family transcriptional regulator
1612738	1655078	42340	OXZ26606.1	B9N49_07880	1654722	1655078	1	plasmid mobilization relaxosome protein MobC
1612738	1618828	6090	OXZ26698.1	B9N49_07720	1612738	1613161	-1	MATE family efflux transporter
1612738	1618828	6090	OXZ26699.1	B9N49_07725	1613183	1614928	-1	ABC transporter ATP-binding protein
1612738	1618828	6090	OXZ26700.1	B9N49_07730	1614929	1616641	-1	ABC transporter ATP-binding protein
1612738	1618828	6090	OXZ26701.1	B9N49_07735	1616658	1618166	-1	ABC transporter ATP-binding protein
1612738	1618828	6090	OXZ26702.1	B9N49_07740	1618154	1618828	-1	hypothetical protein
1635454	1640712	5258		B9N49_07795	1635454	1635747	-1	transposase
1635454	1640712	5258	OXZ26592.1	B9N49_07800	1635859	1636473	-1	hypothetical protein
1635454	1640712	5258	OXZ26593.1	B9N49_07805	1636678	1638042	-1	ABC transporter ATP-binding protein
1635454	1640712	5258	OXZ26594.1	B9N49_07810	1638030	1638725	-1	cobalt ABC transporter permease
1635454	1640712	5258	OXZ26595.1	B9N49_07815	1638725	1639309	-1	hypothetical protein
1635454	1640712	5258	OXZ26596.1	B9N49_07820	1639369	1640712	-1	MATE family efflux transporter
1663712	1668015	4303	OXZ26611.1	B9N49_07920	1663029	1663721	-1	MBL fold metallo-hydrolase
1663712	1668015	4303	OXZ26612.1	B9N49_07925	1663712	1664101	-1	cytidine deaminase
1663712	1668015	4303	OXZ26613.1	B9N49_07930	1664147	1664671	-1	hypothetical protein
1663712	1668015	4303	OXZ26614.1	B9N49_07935	1664888	1665619	-1	23S rRNA (adenine(2058)-N(6))-methyltransferase Erm(A)
1663712	1668015	4303	OXZ26615.1	B9N49_07940	1666115	1666396	-1	hypothetical protein
1663712	1668015	4303		B9N49_07945	1666428	1667165	-1	SAM-dependent methyltransferase
1663712	1668015	4303	OXZ26616.1	B9N49_07950	1667146	1668015	-1	hypothetical protein
1666115	1722368	56253	OXZ26615.1	B9N49_07940	1666115	1666396	-1	hypothetical protein
1666115	1722368	56253		B9N49_07945	1666428	1667165	-1	SAM-dependent methyltransferase
1666115	1722368	56253	OXZ26616.1	B9N49_07950	1667146	1668015	-1	hypothetical protein
1666115	1722368	56253	OXZ26617.1	B9N49_07955	1668719	1670290	-1	recombinase family protein
1666115	1722368	56253	OXZ26618.1	B9N49_07960	1670283	1670699	-1	integrase
1666115	1722368	56253	OXZ26619.1	B9N49_07965	1670692	1672254	-1	recombinase family protein
1666115	1722368	56253	OXZ26620.1	B9N49_07970	1672314	1672523	-1	hypothetical protein
1666115	1722368	56253	OXZ26621.1	B9N49_07975	1672887	1674623	-1	multidrug ABC transporter ATP-binding protein
1666115	1722368	56253	OXZ26622.1	B9N49_07980	1674616	1676334	-1	ABC transporter ATP-binding protein
1666115	1722368	56253	OXZ26623.1	B9N49_07985	1676339	1676929	-1	TetR family transcriptional regulator
1666115	1722368	56253	OXZ26624.1	B9N49_07990	1677106	1677915	-1	lysozyme
1666115	1722368	56253	OXZ26625.1	B9N49_07995	1677916	1678317	-1	holin
1666115	1722368	56253	OXZ26626.1	B9N49_08000	1678319	1679698	-1	hypothetical protein
1666115	1722368	56253	OXZ26627.1	B9N49_08005	1679685	1681556	-1	hypothetical protein
1666115	1722368	56253	OXZ26628.1	B9N49_08010	1681556	1681918	-1	hypothetical protein
1666115	1722368	56253	OXZ26629.1	B9N49_08015	1681918	1684983	-1	phage tail tape measure protein
1666115	1722368	56253	OXZ26630.1	B9N49_08020	1685073	1685396	1	prevent-host-death protein
1666115	1722368	56253	OXZ26631.1	B9N49_08025	1685393	1685722	1	addiction module toxin RelE
1666115	1722368	56253	OXZ26632.1	B9N49_08030	1685901	1686257	-1	hypothetical protein
1666115	1722368	56253	OXZ26633.1	B9N49_08035	1686260	1686856	-1	phage tail protein
1666115	1722368	56253	OXZ26634.1	B9N49_08040	1686846	1687025	-1	hypothetical protein
1666115	1722368	56253	OXZ26635.1	B9N49_08045	1687018	1687344	-1	hypothetical protein
1666115	1722368	56253	OXZ26636.1	B9N49_08050	1687337	1687717	-1	hypothetical protein
1666115	1722368	56253	OXZ26637.1	B9N49_08055	1687714	1688046	-1	phage head-tail adapter protein
1666115	1722368	56253	OXZ26638.1	B9N49_08060	1688046	1688318	-1	AraC family transcriptional regulator
1666115	1722368	56253	OXZ26639.1	B9N49_08065	1688329	1689519	-1	phage major capsid protein
1666115	1722368	56253	OXZ26640.1	B9N49_08070	1689522	1690208	-1	peptidase
1666115	1722368	56253	OXZ26641.1	B9N49_08075	1690198	1691472	-1	phage portal protein
1666115	1722368	56253	OXZ26642.1	B9N49_08080	1691499	1692395	-1	cell filamentation protein Fic
1666115	1722368	56253	OXZ26643.1	B9N49_08085	1692496	1694085	-1	terminase
1666115	1722368	56253	OXZ26644.1	B9N49_08090	1694178	1694408	-1	hypothetical protein
1666115	1722368	56253	OXZ26645.1	B9N49_08095	1694409	1694741	-1	hypothetical protein
1666115	1722368	56253	OXZ26646.1	B9N49_08100	1694822	1695052	-1	DUF4314 domain-containing protein
1666115	1722368	56253	OXZ26647.1	B9N49_08105	1695096	1696538	-1	DNA (cytosine-5-)-methyltransferase
1666115	1722368	56253	OXZ26648.1	B9N49_08110	1696531	1697766	-1	DNA modification methylase
1666115	1722368	56253	OXZ26649.1	B9N49_08115	1697763	1698545	-1	S-adenosylmethionine synthetase
1666115	1722368	56253	OXZ26650.1	B9N49_08120	1698551	1699198	-1	terminase
1666115	1722368	56253	OXZ26651.1	B9N49_08125	1699252	1699608	-1	HNH endonuclease
1666115	1722368	56253	OXZ26652.1	B9N49_08130	1699798	1700055	1	AbrB family transcriptional regulator
1666115	1722368	56253	OXZ26653.1	B9N49_08135	1700055	1700321	1	cytotoxin
1666115	1722368	56253	OXZ26654.1	B9N49_08140	1700398	1700799	-1	hypothetical protein
1666115	1722368	56253	OXZ26655.1	B9N49_08145	1700847	1702190	-1	DEAD/DEAH box helicase
1666115	1722368	56253	OXZ26656.1	B9N49_08150	1702191	1702472	-1	nuclease

1666115	1722368	56253	OXZ26657.1	B9N49_08155	1702430	1702687	-1	hypothetical protein
1666115	1722368	56253	OXZ26658.1	B9N49_08160	1702950	1705151	-1	DNA primase
1666115	1722368	56253	OXZ26659.1	B9N49_08165	1705152	1705538	-1	DUF4406 domain-containing protein
1666115	1722368	56253	OXZ26660.1	B9N49_08170	1705531	1705731	-1	hypothetical protein
1666115	1722368	56253	OXZ26661.1	B9N49_08175	1705745	1708603	-1	hypothetical protein
1666115	1722368	56253	OXZ26662.1	B9N49_08180	1708578	1708778	-1	hypothetical protein
1666115	1722368	56253	OXZ26663.1	B9N49_08185	1708788	1709375	-1	hypothetical protein
1666115	1722368	56253	OXZ26664.1	B9N49_08190	1709341	1710453	-1	nuclease
1666115	1722368	56253	OXZ26665.1	B9N49_08195	1710453	1710752	-1	hypothetical protein
1666115	1722368	56253	OXZ26666.1	B9N49_08200	1710694	1711065	-1	hypothetical protein
1666115	1722368	56253	OXZ26667.1	B9N49_08205	1711053	1711571	-1	RNA polymerase subunit sigma-70
1666115	1722368	56253	OXZ26668.1	B9N49_08210	1712143	1712928	-1	hypothetical protein
1666115	1722368	56253	OXZ26669.1	B9N49_08215	1712931	1713302	-1	transcriptional regulator
1666115	1722368	56253	OXZ26670.1	B9N49_08220	1713553	1714767	-1	23S rRNA (uracil(1939)-C(5))-methyltransferase RlmD
1666115	1722368	56253	OXZ26671.1	B9N49_08225	1714860	1716656	-1	ABC transporter
1666115	1722368	56253	OXZ26672.1	B9N49_08230	1716649	1718409	-1	multidrug ABC transporter ATP-binding protein
1666115	1722368	56253	OXZ26673.1	B9N49_08235	1718384	1718860	-1	MarR family transcriptional regulator
1666115	1722368	56253	OXZ26674.1	B9N49_08240	1719023	1720324	-1	phosphomethylpyrimidine synthase
1666115	1722368	56253	OXZ26675.1	B9N49_08245	1720505	1721419	-1	cyclic pyranopterin monophosphate synthase MoaC
1666115	1722368	56253	OXZ26676.1	B9N49_08250	1721421	1722368	-1	GTP 3',8-cyclase MoaA
1904216	1927243	23027	OXZ26463.1	B9N49_09015	1904216	1904974	-1	metal-dependent hydrolase
1904216	1927243	23027	OXZ26464.1	B9N49_09020	1905100	1905570	1	aminoacyl-tRNA deacylase
1904216	1927243	23027	OXZ26465.1	B9N49_09025	1905573	1906877	1	glutamate--cysteine ligase
1904216	1927243	23027	OXZ26466.1	B9N49_09030	1906870	1908180	1	glutathionylspermidine synthase
1904216	1927243	23027	OXZ26467.1	B9N49_09035	1908279	1908548	-1	Txe/YoeB family addiction module toxin
1904216	1927243	23027	OXZ26468.1	B9N49_09040	1908542	1908790	-1	prevent-host-death protein
1904216	1927243	23027	OXZ26469.1	B9N49_09045	1908972	1909391	1	CMP deaminase
1904216	1927243	23027	OXZ26470.1	B9N49_09050	1909403	1912822	1	pyruvate carboxylase
1904216	1927243	23027	OXZ26471.1	B9N49_09055	1912883	1913277	-1	hypothetical protein
1904216	1927243	23027	OXZ26373.1	B9N49_09060	1914278	1914416	-1	transposase
1904216	1927243	23027	OXZ26374.1	B9N49_09065	1914481	1914885	-1	DUF3139 domain-containing protein
1904216	1927243	23027	OXZ26375.1	B9N49_09070	1915016	1915258	1	cell division protein FtsW
1904216	1927243	23027	OXZ26376.1	B9N49_09075	1915362	1915769	1	hypothetical protein
1904216	1927243	23027	OXZ26377.1	B9N49_09080	1915786	1916292	-1	thiol reductase thioredoxin
1904216	1927243	23027	OXZ26378.1	B9N49_09085	1916397	1916741	-1	hypothetical protein
1904216	1927243	23027	OXZ26379.1	B9N49_09090	1916809	1917498	-1	hypothetical protein
1904216	1927243	23027	OXZ26380.1	B9N49_09095	1917495	1922597	-1	FMN-binding domain-containing protein
1904216	1927243	23027	OXZ26446.1	B9N49_09100	1922842	1923795	-1	2-dehydropantoate 2-reductase
1904216	1927243	23027	OXZ26381.1	B9N49_09105	1923815	1925464	-1	inorganic pyrophosphatase
1904216	1927243	23027	OXZ26382.1	B9N49_09110	1925629	1926150	1	N-carbamoylsarcosine amidohydrolase
1904216	1927243	23027	OXZ26383.1	B9N49_09115	1926192	1926818	1	hypothetical protein
1904216	1927243	23027	OXZ26384.1	B9N49_09120	1926887	1927243	-1	hypothetical protein
1978424	2050817	72393	OXZ26444.1	B9N49_09450	1978424	1978642	1	hypothetical protein
1978424	2050817	72393	OXZ26445.1	B9N49_09455	1978652	1978798	1	YvrJ family protein
1978424	2050817	72393	OXZ26339.1	B9N49_09620	1982645	1984502	-1	hypothetical protein
1978424	2050817	72393	OXZ26340.1	B9N49_09625	1984526	1985835	-1	2,3-dihydroxybenzoate-AMP ligase
1978424	2050817	72393	OXZ26306.1	B9N49_09730	1990594	1990988	-1	IS3 family transposase
1978424	2050817	72393	OXZ26307.1	B9N49_09735	1991183	1991626	-1	transposase
1978424	2050817	72393	OXZ26272.1	B9N49_09745	1992786	1993660	1	IS30 family transposase
1978424	2050817	72393	OXZ26341.1	B9N49_09560	1994661	1995031	-1	thioesterase
1978424	2050817	72393	OXZ26342.1	B9N49_09565	1995194	1995565	-1	transcriptional regulator
1978424	2050817	72393	OXZ26343.1	B9N49_09570	1995941	1996297	1	plasmid mobilization relaxosome protein MobC
1978424	2050817	72393	OXZ26344.1	B9N49_09575	1996299	1997630	1	endonuclease
1978424	2050817	72393	OXZ26345.1	B9N49_09580	1998089	1999705	-1	ABC transporter-like protein
1978424	2050817	72393	OXZ26346.1	B9N49_09585	1999720	2001807	-1	ABC transporter permease
1978424	2050817	72393	OXZ26347.1	B9N49_09590	2001809	2002483	-1	ABC transporter ATP-binding protein
1978424	2050817	72393	OXZ26348.1	B9N49_09595	2002493	2003836	-1	radical SAM domain-containing protein
1978424	2050817	72393	OXZ26349.1	B9N49_09600	2004202	2004630	-1	hypothetical protein
1978424	2050817	72393	OXZ26350.1	B9N49_09605	2004643	2004906	-1	hypothetical protein
1978424	2050817	72393	OXZ26351.1	B9N49_09610	2004900	2005469	-1	hypothetical protein
1978424	2050817	72393	OXZ26352.1	B9N49_09615	2005823	2006014	1	hypothetical protein
1978424	2050817	72393	OXZ26328.1	B9N49_09680	2007188	2007334	1	hypothetical protein
1978424	2050817	72393	OXZ26329.1	B9N49_09685	2007336	2008199	1	hypothetical protein
1978424	2050817	72393	OXZ26330.1	B9N49_09690	2008217	2008474	1	hypothetical protein
1978424	2050817	72393		B9N49_09695	2008474	2008623	1	conjugal transfer protein
1978424	2050817	72393	OXZ26331.1	B9N49_09700	2008662	2008749	-1	hypothetical protein
1978424	2050817	72393	OXZ26178.1	B9N49_09775	2009750	2010348	1	hypothetical protein
1978424	2050817	72393	OXZ26337.1	B9N49_09630	2011349	2013215	-1	non-ribosomal peptide synthetase
1978424	2050817	72393	OXZ26338.1	B9N49_09635	2013228	2014531	-1	2,3-dihydroxybenzoate-AMP ligase
1978424	2050817	72393	OXZ26273.1	B9N49_09740	2015532	2016545	-1	elongation factor Tu
1978424	2050817	72393	OXZ26181.1	B9N49_09755	2017546	2017915	-1	phage antirepressor Ant
1978424	2050817	72393	OXZ26182.1	B9N49_09760	2017912	2018226	-1	hypothetical protein
1978424	2050817	72393	OXZ26308.1	B9N49_09720	2019290	2019847	1	transposase
1978424	2050817	72393	OXZ26309.1	B9N49_09725	2019874	2020704	1	hypothetical protein
1978424	2050817	72393	OXZ26332.1	B9N49_09670	2021838	2023118	-1	group II intron reverse transcriptase/maturase
1978424	2050817	72393		B9N49_09650	2024585	2025400	1	conjugal transfer protein TraG
1978424	2050817	72393	OXZ26333.1	B9N49_09655	2025552	2026418	1	CAAX protease
1978424	2050817	72393	OXZ26334.1	B9N49_09660	2026609	2026932	1	single-stranded DNA-binding protein
1978424	2050817	72393	OXZ26335.1	B9N49_09665	2026933	2027064	1	hypothetical protein
1978424	2050817	72393	OXZ26336.1	B9N49_09640	2028341	2030200	-1	group II intron reverse transcriptase/maturase
1978424	2050817	72393	OXZ26179.1	B9N49_09765	2031943	2032029	1	hypothetical protein
1978424	2050817	72393	OXZ26180.1	B9N49_09770	2032256	2032620	-1	hypothetical protein
1978424	2050817	72393	OXZ26353.1	B9N49_09460	2033621	2033706	1	hypothetical protein
1978424	2050817	72393	OXZ26354.1	B9N49_09465	2033784	2034179	-1	hypothetical protein
1978424	2050817	72393	OXZ26355.1	B9N49_09470	2034121	2034435	-1	hypothetical protein

1978424	2050817	72393	OXZ26356.1	B9N49_09475	2034641	2034817	-1	lantibiotic salivaricin M precursor
1978424	2050817	72393	OXZ26357.1	B9N49_09480	2034941	2035561	1	DNA-binding protein
1978424	2050817	72393	OXZ26358.1	B9N49_09485	2035862	2037409	1	hypothetical protein
1978424	2050817	72393	OXZ26359.1	B9N49_09490	2037381	2037986	1	DNA-binding response regulator
1978424	2050817	72393	OXZ26360.1	B9N49_09495	2038268	2040454	1	radical SAM protein
1978424	2050817	72393	OXZ26361.1	B9N49_09500	2040456	2040722	1	CXXX repeat peptide modification system protein
1978424	2050817	72393	OXZ26362.1	B9N49_09505	2040715	2042352	1	ABC transporter permease
1978424	2050817	72393	OXZ26363.1	B9N49_09510	2042936	2044330	1	hypothetical protein
1978424	2050817	72393	OXZ26364.1	B9N49_09515	2044333	2045364	1	hypothetical protein
1978424	2050817	72393	OXZ26365.1	B9N49_09520	2045515	2046402	1	hypothetical protein
1978424	2050817	72393	OXZ26366.1	B9N49_09525	2046419	2046700	1	hypothetical protein
1978424	2050817	72393	OXZ26367.1	B9N49_09530	2046712	2047179	1	hypothetical protein
1978424	2050817	72393	OXZ26368.1	B9N49_09535	2047188	2047391	1	hypothetical protein
1978424	2050817	72393	OXZ26369.1	B9N49_09540	2047388	2048299	1	hypothetical protein
1978424	2050817	72393	OXZ26370.1	B9N49_09545	2048325	2049098	1	hypothetical protein
1978424	2050817	72393	OXZ26371.1	B9N49_09550	2049095	2050033	1	hypothetical protein
1978424	2050817	72393	OXZ26372.1	B9N49_09555	2050026	2050817	1	hypothetical protein
1978652	1985835	7183	OXZ26445.1	B9N49_09455	1978652	1978798	1	YvrJ family protein
1978652	1985835	7183	OXZ26339.1	B9N49_09620	1982645	1984502	-1	hypothetical protein
1978652	1985835	7183	OXZ26340.1	B9N49_09625	1984526	1985835	-1	2,3-dihydroxybenzoate-AMP ligase
1991183	2004906	13723	OXZ26307.1	B9N49_09735	1991183	1991626	-1	transposase
1991183	2004906	13723	OXZ26272.1	B9N49_09745	1992786	1993660	1	IS30 family transposase
1991183	2004906	13723	OXZ26341.1	B9N49_09560	1994661	1995031	-1	thioesterase
1991183	2004906	13723	OXZ26342.1	B9N49_09565	1995194	1995565	-1	transcriptional regulator
1991183	2004906	13723	OXZ26343.1	B9N49_09570	1995941	1996297	1	plasmid mobilization relaxosome protein MobC
1991183	2004906	13723	OXZ26344.1	B9N49_09575	1996299	1997630	1	endonuclease
1991183	2004906	13723	OXZ26345.1	B9N49_09580	1998089	1999705	-1	ABC transporter-like protein
1991183	2004906	13723	OXZ26346.1	B9N49_09585	1999720	2001807	-1	ABC transporter permease
1991183	2004906	13723	OXZ26347.1	B9N49_09590	2001809	2002483	-1	ABC transporter ATP-binding protein
1991183	2004906	13723	OXZ26348.1	B9N49_09595	2002493	2003836	-1	radical SAM domain-containing protein
1991183	2004906	13723	OXZ26349.1	B9N49_09600	2004202	2004630	-1	hypothetical protein
1991183	2004906	13723	OXZ26350.1	B9N49_09605	2004643	2004906	-1	hypothetical protein
1991183	2004906	13723	OXZ26351.1	B9N49_09610	2004900	2005469	-1	hypothetical protein
2017546	2026418	8872	OXZ26181.1	B9N49_09755	2017546	2017915	-1	phage antirepressor Ant
2017546	2026418	8872	OXZ26182.1	B9N49_09760	2017912	2018226	-1	hypothetical protein
2017546	2026418	8872	OXZ26308.1	B9N49_09720	2019290	2019847	1	transposase
2017546	2026418	8872	OXZ26309.1	B9N49_09725	2019874	2020704	1	hypothetical protein
2017546	2026418	8872	OXZ26332.1	B9N49_09670	2021838	2023118	-1	group II intron reverse transcriptase/maturase
2017546	2026418	8872		B9N49_09650	2024585	2025400	1	conjugal transfer protein TraG
2017546	2026418	8872	OXZ26333.1	B9N49_09655	2025552	2026418	1	CAAX protease
2046419	2056025	9606	OXZ26366.1	B9N49_09525	2046419	2046700	1	hypothetical protein
2046419	2056025	9606	OXZ26367.1	B9N49_09530	2046712	2047179	1	hypothetical protein
2046419	2056025	9606	OXZ26368.1	B9N49_09535	2047188	2047391	1	hypothetical protein
2046419	2056025	9606	OXZ26369.1	B9N49_09540	2047388	2048299	1	hypothetical protein
2046419	2056025	9606	OXZ26370.1	B9N49_09545	2048325	2049098	1	hypothetical protein
2046419	2056025	9606	OXZ26371.1	B9N49_09550	2049095	2050033	1	hypothetical protein
2046419	2056025	9606	OXZ26372.1	B9N49_09555	2050026	2050817	1	hypothetical protein
2046419	2056025	9606	OXZ26325.1	B9N49_09705	2052644	2053152	1	thioesterase
2046419	2056025	9606	OXZ26326.1	B9N49_09710	2053142	2053834	1	4-phosphopantetheinyl transferase
2046419	2056025	9606	OXZ26327.1	B9N49_09715	2053834	2054243	1	2,3-dihydroxybenzoate-AMP ligase
2046419	2056025	9606	OXZ26271.1	B9N49_09750	2055244	2056025	-1	hypothetical protein

Table S3I: Predicted genomic islands in the genome of *"F. nericia"* T151023

Shown are genes of genomic islands predicted by IslandViewer4

Island start	Island end	Length	Gene name	Locus	Gene start	Gene end	Strand	Product
81511	88758	7247	OXZ29531.1	B9N51_00605	79542	81530	1	exopolyphosphatase
81511	88758	7247	OXZ29532.1	B9N51_00610	81511	81957	1	50S ribosomal protein L9
81511	88758	7247	OXZ29533.1	B9N51_00615	81991	83328	1	replicative DNA helicase
81511	88758	7247	OXZ29534.1	B9N51_00620	83325	83888	1	GNAT family N-acetyltransferase
81511	88758	7247	OXZ29535.1	B9N51_00625	83893	84651	1	DNA replication protein
81511	88758	7247	OXZ29536.1	B9N51_00630	84654	85631	1	DNA replication protein DnaC
81511	88758	7247	OXZ29537.1	B9N51_00635	85628	86272	1	NAD(P)H-hydrate epimerase
81511	88758	7247	OXZ29538.1	B9N51_00640	86777	87319	1	ECF transporter S component
81511	88758	7247	OXZ29539.1	B9N51_00645	87303	87989	1	radical SAM protein
81511	88758	7247	OXZ29540.1	B9N51_00650	88054	88758	1	hypothetical protein
143045	188929	45884	OXZ29593.1	B9N51_00925	143045	143674	1	50S ribosomal protein L3
143045	188929	45884	OXZ29594.1	B9N51_00930	143690	144313	1	50S ribosomal protein L4
143045	188929	45884	OXZ29595.1	B9N51_00935	144313	144603	1	50S ribosomal protein L23
143045	188929	45884	OXZ29596.1	B9N51_00940	144620	145450	1	50S ribosomal protein L2
143045	188929	45884	OXZ29597.1	B9N51_00945	145463	145747	1	30S ribosomal protein S19
143045	188929	45884	OXZ29598.1	B9N51_00950	145765	146103	1	50S ribosomal protein L22
143045	188929	45884	OXZ29599.1	B9N51_00955	146115	146849	1	30S ribosomal protein S3
143045	188929	45884	OXZ29600.1	B9N51_00960	146871	147314	1	50S ribosomal protein L16
143045	188929	45884	OXZ29601.1	B9N51_00965	147304	147510	1	50S ribosomal protein L29
143045	188929	45884	OXZ29602.1	B9N51_00970	147516	147770	1	30S ribosomal protein S17
143045	188929	45884	OXZ29603.1	B9N51_00975	147795	148163	1	50S ribosomal protein L14
143045	188929	45884	OXZ29604.1	B9N51_00980	148182	148490	1	50S ribosomal protein L24
143045	188929	45884	OXZ29605.1	B9N51_00985	148509	149051	1	50S ribosomal protein L5
143045	188929	45884	OXZ29606.1	B9N51_00990	149064	149249	1	30S ribosomal protein S14 type Z
143045	188929	45884	OXZ29607.1	B9N51_00995	149271	149666	1	30S ribosomal protein S8
143045	188929	45884	OXZ29608.1	B9N51_01000	149693	150232	1	50S ribosomal protein L6
143045	188929	45884	OXZ29609.1	B9N51_01005	150247	150609	1	50S ribosomal protein L18
143045	188929	45884	OXZ29610.1	B9N51_01010	150630	151136	1	30S ribosomal protein S5
143045	188929	45884	OXZ29611.1	B9N51_01015	151150	151332	1	50S ribosomal protein L30
143045	188929	45884	OXZ29612.1	B9N51_01020	151344	151793	1	50S ribosomal protein L15
143045	188929	45884	OXZ29613.1	B9N51_01025	151795	153069	1	preprotein translocase subunit SecY
143045	188929	45884	OXZ29614.1	B9N51_01030	153080	153727	1	adenylate kinase
143045	188929	45884	OXZ29615.1	B9N51_01035	153733	154017	1	KOW domain-containing protein
143045	188929	45884	OXZ29616.1	B9N51_01040	153998	154213	1	translation initiation factor IF-1
143045	188929	45884	OXZ29617.1	B9N51_01045	154228	154341	1	50S ribosomal protein L36
143045	188929	45884	OXZ29618.1	B9N51_01050	154354	154704	1	30S ribosomal protein S13
143045	188929	45884	OXZ29619.1	B9N51_01055	154720	155127	1	30S ribosomal protein S11
143045	188929	45884	OXZ29620.1	B9N51_01060	155183	156136	1	DNA-directed RNA polymerase subunit alpha
143045	188929	45884	OXZ29621.1	B9N51_01065	156150	156545	1	50S ribosomal protein L17
143045	188929	45884	OXZ29622.1	B9N51_01070	156909	158750	1	hypothetical protein
143045	188929	45884	OXZ29623.1	B9N51_01075	159388	161361	1	N-acetylmuramoyl-L-alanine amidase
143045	188929	45884	OXZ29624.1	B9N51_01080	161458	162054	1	30S ribosomal protein S4
143045	188929	45884	OXZ29625.1	B9N51_01085	162158	163393	1	peptidoglycan-binding protein
143045	188929	45884	OXZ29524.1	B9N51_01130	167644	171711	1	hypothetical protein
143045	188929	45884	OXZ29525.1	B9N51_01135	171807	171895	-1	hypothetical protein
143045	188929	45884	OXZ29516.1	B9N51_01140	173176	182115	1	hypothetical protein
143045	188929	45884	OXZ29517.1	B9N51_01145	182248	182928	1	hypothetical protein
143045	188929	45884	OXZ29518.1	B9N51_01150	183357	185522	1	hypothetical protein
143045	188929	45884	OXZ29519.1	B9N51_01155	185644	187059	1	cell wall anchor protein
143045	188929	45884	OXZ29520.1	B9N51_01160	187237	188103	1	class C sortase
143045	188929	45884	OXZ29521.1	B9N51_01165	188090	188929	1	class C sortase
143045	188929	45884	OXZ29522.1	B9N51_01170	188916	189767	1	class C sortase
210413	218373	7960	OXZ29323.1	B9N51_01710	210413	210874	1	CtsR family transcriptional regulator
210413	218373	7960	OXZ29324.1	B9N51_01715	210885	211484	1	hypothetical protein
210413	218373	7960	OXZ29325.1	B9N51_01720	211477	212472	1	ATP--guanido phosphotransferase
210413	218373	7960	OXZ29326.1	B9N51_01725	212469	214889	1	ATP-dependent Clp protease ATP-binding protein ClpC
210413	218373	7960	OXZ29327.1	B9N51_01730	214891	216252	1	DNA repair protein RadA
210413	218373	7960	OXZ29328.1	B9N51_01735	216281	216586	-1	hypothetical protein
210413	218373	7960	OXZ29329.1	B9N51_01740	216688	218373	-1	M3 family oligoendopeptidase
598893	604825	5932	OXZ28763.1	B9N51_03760	598893	599084	1	50S ribosomal protein L35
598893	604825	5932	OXZ28764.1	B9N51_03765	599103	599456	1	50S ribosomal protein L20
598893	604825	5932	OXZ28765.1	B9N51_03770	599488	599763	-1	hypothetical protein
598893	604825	5932	OXZ28766.1	B9N51_03775	599763	600380	-1	recombinase RecX
598893	604825	5932	OXZ28767.1	B9N51_03780	600569	600808	1	hypothetical protein
598893	604825	5932	OXZ28768.1	B9N51_03785	600817	601893	1	phosphoesterase
598893	604825	5932	OXZ28769.1	B9N51_03790	601890	604142	1	DNA repair protein Rad50
598893	604825	5932	OXZ28770.1	B9N51_03795	604145	604825	1	serine/threonine protein phosphatase
614359	676348	61989	OXZ28780.1	B9N51_03845	614359	615057	1	DNA-binding response regulator
614359	676348	61989	OXZ28781.1	B9N51_03850	615099	615683	1	peptidylprolyl isomerase
614359	676348	61989	OXZ28782.1	B9N51_03855	615685	616296	1	thymidine kinase
614359	676348	61989	OXZ28783.1	B9N51_03860	616339	616809	-1	S-ribosylhomocysteine lyase
614359	676348	61989	OXZ28784.1	B9N51_03865	616818	617921	-1	rod shape-determining protein RodA
614359	676348	61989	OXZ28785.1	B9N51_03870	618006	618839	1	endonuclease IV
614359	676348	61989	OXZ28786.1	B9N51_03875	618820	619446	1	endonuclease III
614359	676348	61989	OXZ28787.1	B9N51_03880	619515	620435	1	aspartate carbamoyltransferase
614359	676348	61989	OXZ28788.1	B9N51_03885	620435	620863	1	aspartate carbamoyltransferase regulatory subunit
614359	676348	61989	OXZ28789.1	B9N51_03890	620863	622059	1	dihydroorotase
614359	676348	61989	OXZ28790.1	B9N51_03895	622046	622897	1	orotidine-5'-phosphate decarboxylase
614359	676348	61989	OXZ28791.1	B9N51_03900	622890	623606	1	dihydroorotate dehydrogenase electron transfer subunit

614359	676348	61989	OXZ28792.1	B9N51_03905	623599	624495	1	dihydroorotate dehydrogenase B catalytic subunit
614359	676348	61989	OXZ28793.1	B9N51_03910	624506	625078	1	orotate phosphoribosyltransferase
614359	676348	61989	OXZ28794.1	B9N51_03915	625207	625911	1	isoprenyl transferase
614359	676348	61989	OXZ28795.1	B9N51_03920	625908	626687	1	phosphatidate cytidylyltransferase
614359	676348	61989	OXZ28796.1	B9N51_03925	626700	627704	1	RIP metalloprotease RseP
614359	676348	61989	OXZ28797.1	B9N51_03930	627755	628804	1	4-hydroxy-3-methylbut-2-en-1-yl diphosphate synthase
614359	676348	61989	OXZ28798.1	B9N51_03935	628805	633070	1	PolC-type DNA polymerase III
614359	676348	61989	OXZ28799.1	B9N51_03940	633103	634086	1	phosphate starvation-inducible protein PhoH
614359	676348	61989	OXZ28800.1	B9N51_03945	634067	634528	1	rRNA maturation RNase YbeY
614359	676348	61989	OXZ28801.1	B9N51_03950	634512	635255	1	diacylglycerol kinase
614359	676348	61989	OXZ28802.1	B9N51_03955	635263	635658	1	cytidine deaminase
614359	676348	61989	OXZ28803.1	B9N51_03960	635651	636535	1	GTPase Era
614359	676348	61989	OXZ28804.1	B9N51_03965	636535	637239	1	DNA repair protein RecO
614359	676348	61989	OXZ28805.1	B9N51_03970	637249	638937	1	hypothetical protein
614359	676348	61989	OXZ28806.1	B9N51_03975	638948	639739	1	hypothetical protein
614359	676348	61989	OXZ28807.1	B9N51_03980	639829	641271	1	serine/threonine protein kinase
614359	676348	61989	OXZ28808.1	B9N51_03985	641273	641947	1	protein phosphatase
614359	676348	61989	OXZ28809.1	B9N51_03990	642004	643389	1	tRNA (N6-isopentenyl adenosine(37)-C2)-methylthiotransferase MiaB
614359	676348	61989	OXZ28810.1	B9N51_03995	643391	645970	1	DNA mismatch repair protein MutS
614359	676348	61989	OXZ28811.1	B9N51_04000	645963	647846	1	DNA mismatch repair protein MutL
614359	676348	61989	OXZ28812.1	B9N51_04005	647843	648787	1	tRNA (adenosine(37)-N6)-dimethylallyltransferase MiaA
614359	676348	61989	OXZ28813.1	B9N51_04010	648774	650048	1	hypothetical protein
614359	676348	61989	OXZ28814.1	B9N51_04015	650099	650725	-1	LexA repressor
614359	676348	61989	OXZ28815.1	B9N51_04020	650900	651211	1	peptidoglycan-binding protein LysM
614359	676348	61989	OXZ28816.1	B9N51_04025	651291	652271	-1	recombinase XerC
614359	676348	61989		B9N51_04030	652377	653553	-1	aspartate aminotransferase
614359	676348	61989	OXZ28817.1	B9N51_04035	653540	654361	-1	metal-dependent phosphoesterase
614359	676348	61989	OXZ28818.1	B9N51_04040	654430	655971	-1	ribonuclease Y
614359	676348	61989	OXZ28819.1	B9N51_04045	656090	656752	-1	chromosome partitioning protein ParA
614359	676348	61989	OXZ28820.1	B9N51_04050	656739	656978	-1	hypothetical protein
614359	676348	61989	OXZ28821.1	B9N51_04055	656978	658561	-1	exonuclease SbcC
614359	676348	61989	OXZ28892.1	B9N51_04060	658561	659511	-1	phosphoesterase
614359	676348	61989	OXZ28822.1	B9N51_04065	659542	660633	-1	recombinase RecA
614359	676348	61989	OXZ28823.1	B9N51_04070	660702	661238	-1	CDP-diacylglycerol--glycerol-3-phosphate 3-phosphatidyltransferase
614359	676348	61989	OXZ28824.1	B9N51_04075	661240	663465	-1	cell division protein FtsK
614359	676348	61989	OXZ28825.1	B9N51_04080	663468	663899	-1	deoxyuridine 5'-triphosphate nucleotidohydrolase
614359	676348	61989	OXZ28826.1	B9N51_04085	663951	666095	-1	polyribonucleotide nucleotidyltransferase
614359	676348	61989	OXZ28827.1	B9N51_04090	666142	666408	-1	30S ribosomal protein S15
614359	676348	61989	OXZ28828.1	B9N51_04095	666489	667415	-1	riboflavin biosynthesis protein RibF
614359	676348	61989	OXZ28829.1	B9N51_04100	667406	668269	-1	tRNA pseudouridine(55) synthase TruB
614359	676348	61989	OXZ28830.1	B9N51_04105	668253	669221	-1	exopolyphosphatase
614359	676348	61989	OXZ28831.1	B9N51_04110	669211	669576	-1	ribosome-binding factor A
614359	676348	61989	OXZ28832.1	B9N51_04115	669584	671884	-1	translation initiation factor IF-2
614359	676348	61989	OXZ28833.1	B9N51_04120	671903	672166	-1	RNA-binding protein
614359	676348	61989	OXZ28834.1	B9N51_04125	672192	673466	-1	transcription termination/antitermination protein NusA
614359	676348	61989	OXZ28835.1	B9N51_04130	673476	673940	-1	ribosome maturation factor
614359	676348	61989	OXZ28836.1	B9N51_04135	674047	674460	-1	hypothetical protein
614359	676348	61989	OXZ28837.1	B9N51_04140	674460	675443	-1	hypothetical protein
614359	676348	61989	OXZ28838.1	B9N51_04145	675445	676062	-1	nodulation efficiency protein D
614359	676348	61989	OXZ28839.1	B9N51_04150	676169	676348	-1	30S ribosomal protein S21
858783	869107	10324	OXZ28383.1	B9N51_04985	858783	860063	1	hypothetical protein
858783	869107	10324	OXZ28384.1	B9N51_04990	860056	860400	1	hypothetical protein
858783	869107	10324	OXZ28250.1	B9N51_04995	861555	861643	1	hypothetical protein
858783	869107	10324	OXZ28251.1	B9N51_05000	861771	862706	-1	sugar ABC transporter permease
858783	869107	10324	OXZ28252.1	B9N51_05005	862706	863806	-1	ABC transporter permease
858783	869107	10324	OXZ28253.1	B9N51_05010	863796	865328	-1	heme ABC transporter ATP-binding protein
858783	869107	10324	OXZ28254.1	B9N51_05015	865394	866470	-1	BMP family ABC transporter substrate-binding protein
858783	869107	10324	OXZ28255.1	B9N51_05020	866710	867735	1	NADH dehydrogenase subunit
858783	869107	10324	OXZ28256.1	B9N51_05025	867758	869107	-1	uracil permease
1340012	1346417	6405	OXZ27371.1	B9N51_07210	1340012	1340278	-1	hypothetical protein
1340012	1346417	6405	OXZ27372.1	B9N51_07215	1340262	1340555	-1	hypothetical protein
1340012	1346417	6405	OXZ27373.1	B9N51_07220	1340686	1341210	1	DNA topology modulation protein FlaR
1340012	1346417	6405		B9N51_07225	1341893	1343837	-1	cell wall protein
1340012	1346417	6405	OXZ27374.1	B9N51_07230	1344543	1344977	-1	GNAT family N-acetyltransferase
1340012	1346417	6405	OXZ27375.1	B9N51_07235	1345000	1345698	-1	hypothetical protein
1340012	1346417	6405	OXZ27376.1	B9N51_07240	1345698	1346417	-1	peptidase S51
1378722	1422726	44004	OXZ29512.1	B9N51_01610	1378722	1379030	-1	exosortase
1378722	1422726	44004	OXZ29511.1	B9N51_01605	1379036	1379707	-1	ATPase
1378722	1422726	44004	OXZ29510.1	B9N51_01600	1379912	1381450	-1	hypothetical protein
1378722	1422726	44004	OXZ29509.1	B9N51_01595	1381626	1383134	-1	AAA family ATPase
1378722	1422726	44004	OXZ29508.1	B9N51_01590	1383877	1384767	-1	methionine sulfoxide reductase
1378722	1422726	44004	OXZ29507.1	B9N51_01585	1385002	1386354	-1	hemolysin
1378722	1422726	44004		B9N51_01580	1386767	1387560	-1	lysophospholipase
1378722	1422726	44004	OXZ29506.1	B9N51_01575	1387770	1390295	-1	hypothetical protein
1378722	1422726	44004		B9N51_01570	1390527	1390706	-1	hypothetical protein
1378722	1422726	44004		B9N51_01565	1390676	1390825	-1	protein-S-isoprenylcysteine methyltransferase
1378722	1422726	44004	OXZ29505.1	B9N51_01560	1390839	1391777	-1	esterase
1378722	1422726	44004	OXZ29504.1	B9N51_01555	1391846	1392472	-1	hypothetical protein
1378722	1422726	44004	OXZ29503.1	B9N51_01550	1392500	1393108	-1	SAM-dependent methyltransferase

1378722	1422726	44004	OXZ29502.1	B9N51_01545	1393324	1394073	-1	phosphorylase
1378722	1422726	44004	OXZ29501.1	B9N51_01540	1394196	1395803	-1	macrolide ABC transporter ATP-binding protein
1378722	1422726	44004	OXZ29500.1	B9N51_01535	1396207	1396821	-1	hypothetical protein
1378722	1422726	44004	OXZ29499.1	B9N51_01530	1397160	1398557	-1	ABC transporter ATP-binding protein
1378722	1422726	44004	OXZ29498.1	B9N51_01525	1398550	1399266	-1	cobalt transporter
1378722	1422726	44004	OXZ29497.1	B9N51_01520	1399263	1399862	-1	hypothetical protein
1378722	1422726	44004	OXZ29496.1	B9N51_01515	1399872	1401593	-1	ABC transporter ATP-binding protein
1378722	1422726	44004	OXZ29495.1	B9N51_01510	1401577	1403331	-1	ABC transporter ATP-binding protein
1378722	1422726	44004	OXZ29494.1	B9N51_01505	1403492	1404484	1	AraC family transcriptional regulator
1378722	1422726	44004	OXZ29493.1	B9N51_01500	1404669	1405148	-1	mercury transporter
1378722	1422726	44004	OXZ29492.1	B9N51_01495	1405547	1405960	-1	hypothetical protein
1378722	1422726	44004	OXZ29491.1	B9N51_01490	1406031	1406336	-1	hypothetical protein
1378722	1422726	44004		B9N51_01485	1406504	1406635	-1	adenine methylase
1378722	1422726	44004	OXZ29514.1	B9N51_01480	1406849	1407139	-1	RNA methyltransferase
1378722	1422726	44004	OXZ29490.1	B9N51_01475	1407153	1408907	-1	recombinase family protein
1378722	1422726	44004	OXZ29489.1	B9N51_01470	1409399	1410751	-1	MATE family efflux transporter
1378722	1422726	44004	OXZ29488.1	B9N51_01465	1410773	1412518	-1	ABC transporter ATP-binding protein
1378722	1422726	44004	OXZ29487.1	B9N51_01460	1412519	1414231	-1	ABC transporter ATP-binding protein
1378722	1422726	44004	OXZ29486.1	B9N51_01455	1414247	1415755	-1	ABC transporter ATP-binding protein
1378722	1422726	44004	OXZ29485.1	B9N51_01450	1415743	1416417	-1	hypothetical protein
1378722	1422726	44004	OXZ29484.1	B9N51_01445	1416435	1417037	-1	hypothetical protein
1378722	1422726	44004	OXZ29483.1	B9N51_01440	1417179	1418150	-1	AraC family transcriptional regulator
1378722	1422726	44004	OXZ29482.1	B9N51_01435	1418470	1419747	-1	IS110 family transposase
1378722	1422726	44004	OXZ29481.1	B9N51_01430	1420159	1420500	1	plasmid mobilization relaxosome protein MobC
1378722	1422726	44004	OXZ29480.1	B9N51_01425	1420822	1422726	-1	DNA helicase UvrD
1378722	1422726	44004	OXZ29479.1	B9N51_01420	1422714	1424804	-1	ATP-dependent endonuclease
1393324	1399862	6538	OXZ29502.1	B9N51_01545	1393324	1394073	-1	phosphorylase
1393324	1399862	6538	OXZ29501.1	B9N51_01540	1394196	1395803	-1	macrolide ABC transporter ATP-binding protein
1393324	1399862	6538	OXZ29500.1	B9N51_01535	1396207	1396821	-1	hypothetical protein
1393324	1399862	6538	OXZ29499.1	B9N51_01530	1397160	1398557	-1	ABC transporter ATP-binding protein
1393324	1399862	6538	OXZ29498.1	B9N51_01525	1398550	1399266	-1	cobalt transporter
1393324	1399862	6538	OXZ29497.1	B9N51_01520	1399263	1399862	-1	hypothetical protein
1405547	1418150	12603	OXZ29492.1	B9N51_01495	1405547	1405960	-1	hypothetical protein
1405547	1418150	12603	OXZ29491.1	B9N51_01490	1406031	1406336	-1	hypothetical protein
1405547	1418150	12603		B9N51_01485	1406504	1406635	-1	adenine methylase
1405547	1418150	12603	OXZ29514.1	B9N51_01480	1406849	1407139	-1	RNA methyltransferase
1405547	1418150	12603	OXZ29490.1	B9N51_01475	1407153	1408907	-1	recombinase family protein
1405547	1418150	12603	OXZ29489.1	B9N51_01470	1409399	1410751	-1	MATE family efflux transporter
1405547	1418150	12603	OXZ29488.1	B9N51_01465	1410773	1412518	-1	ABC transporter ATP-binding protein
1405547	1418150	12603	OXZ29487.1	B9N51_01460	1412519	1414231	-1	ABC transporter ATP-binding protein
1405547	1418150	12603	OXZ29486.1	B9N51_01455	1414247	1415755	-1	ABC transporter ATP-binding protein
1405547	1418150	12603	OXZ29485.1	B9N51_01450	1415743	1416417	-1	hypothetical protein
1405547	1418150	12603	OXZ29484.1	B9N51_01445	1416435	1417037	-1	hypothetical protein
1405547	1418150	12603	OXZ29483.1	B9N51_01440	1417179	1418150	-1	AraC family transcriptional regulator
1420159	1426302	6143	OXZ29481.1	B9N51_01430	1420159	1420500	1	plasmid mobilization relaxosome protein MobC
1420159	1426302	6143	OXZ29480.1	B9N51_01425	1420822	1422726	-1	DNA helicase UvrD
1420159	1426302	6143	OXZ29479.1	B9N51_01420	1422714	1424804	-1	ATP-dependent endonuclease
1420159	1426302	6143	OXZ29478.1	B9N51_01415	1424882	1425718	-1	abortive phage infection protein
1420159	1426302	6143	OXZ29477.1	B9N51_01410	1425715	1426302	-1	transcriptional regulator
1469754	1474021	4267	OXZ29513.1	B9N51_01200	1469754	1470101	1	toxin MazF
1469754	1474021	4267	OXZ29435.1	B9N51_01195	1470680	1471255	-1	cell filamentation protein Fic
1469754	1474021	4267	OXZ29434.1	B9N51_01190	1471990	1472724	-1	hydrolase
1469754	1474021	4267	OXZ29433.1	B9N51_01185	1472724	1473227	-1	acetyltransferase
1469754	1474021	4267	OXZ29432.1	B9N51_01180	1473380	1474021	1	hemolysin III
1526090	1536080	9990	OXZ27316.1	B9N51_07615	1524271	1526106	-1	multidrug ABC transporter
1526090	1536080	9990	OXZ27317.1	B9N51_07620	1526090	1527892	-1	multidrug ABC transporter
1526090	1536080	9990	OXZ27318.1	B9N51_07625	1527972	1528637	-1	histidine kinase
1526090	1536080	9990	OXZ27319.1	B9N51_07630	1528645	1529340	-1	DNA-binding response regulator
1526090	1536080	9990	OXZ27320.1	B9N51_07635	1529422	1530285	-1	UTP--glucose-1-phosphate uridylyltransferase
1526090	1536080	9990	OXZ27321.1	B9N51_07640	1531007	1533592	-1	ATP-dependent chaperone ClpB
1526090	1536080	9990	OXZ27322.1	B9N51_07645	1533611	1534555	-1	heat-shock protein
1526090	1536080	9990	OXZ27347.1	B9N51_07650	1534724	1535368	-1	nitroreductase
1526090	1536080	9990	OXZ27323.1	B9N51_07655	1535541	1536080	-1	RNA polymerase subunit sigma
1677255	1704148	26893	OXZ29725.1	B9N51_00210	1677255	1677599	1	anti-sigma factor
1677255	1704148	26893	OXZ29726.1	B9N51_00215	1677604	1678380	1	B/F/G family RNA polymerase sigma-70 factor
1677255	1704148	26893	OXZ29727.1	B9N51_00220	1678381	1678992	1	glutamine ABC transporter ATP-binding protein
1677255	1704148	26893	OXZ29728.1	B9N51_00225	1678994	1679767	1	amino acid ABC transporter permease
1677255	1704148	26893	OXZ29729.1	B9N51_00230	1679781	1681139	1	fumarate hydratase, class II
1677255	1704148	26893	OXZ29738.1	B9N51_00235	1681262	1681438	1	transaldolase
1677255	1704148	26893	OXZ29730.1	B9N51_00240	1682099	1682743	-1	hypothetical protein
1677255	1704148	26893	OXZ29731.1	B9N51_00245	1682740	1683399	-1	ABC transporter
1677255	1704148	26893	OXZ29732.1	B9N51_00250	1683562	1683996	-1	hypothetical protein
1677255	1704148	26893	OXZ29733.1	B9N51_00255	1684006	1684404	-1	hypothetical protein
1677255	1704148	26893	OXZ29734.1	B9N51_00260	1684512	1684697	1	hypothetical protein
1677255	1704148	26893	OXZ29735.1	B9N51_00265	1684709	1684927	1	hypothetical protein
1677255	1704148	26893	OXZ29736.1	B9N51_00270	1684937	1685083	1	YvrJ family protein
1677255	1704148	26893	OXZ27007.1	B9N51_08160	1686267	1687624	-1	hypothetical protein
1677255	1704148	26893	OXZ27023.1	B9N51_08145	1688904	1690073	1	IS110 family transposase
1677255	1704148	26893	OXZ27032.1	B9N51_08100	1691294	1691947	-1	hypothetical protein
1677255	1704148	26893	OXZ27033.1	B9N51_08105	1692112	1692678	-1	transposase
1677255	1704148	26893	OXZ27008.1	B9N51_08150	1695626	1696225	-1	hypothetical protein
1677255	1704148	26893	OXZ27009.1	B9N51_08155	1696272	1696791	-1	hypothetical protein
1677255	1704148	26893		B9N51_08110	1697926	1702752	-1	hypothetical protein

1677255	1704148	26893		B9N51_08115	1703206	1703397	-1	transcriptional regulator
1677255	1704148	26893	OXZ27030.1	B9N51_08120	1703504	1704148	-1	hypothetical protein
1684937	1696791	11854	OXZ29736.1	B9N51_00270	1684937	1685083	1	YvrJ family protein
1684937	1696791	11854	OXZ27007.1	B9N51_08160	1686267	1687624	-1	hypothetical protein
1684937	1696791	11854	OXZ27023.1	B9N51_08145	1688904	1690073	1	IS110 family transposase
1684937	1696791	11854	OXZ27032.1	B9N51_08100	1691294	1691947	-1	hypothetical protein
1684937	1696791	11854	OXZ27033.1	B9N51_08105	1692112	1692678	-1	transposase
1684937	1696791	11854	OXZ27008.1	B9N51_08150	1695626	1696225	-1	hypothetical protein
1684937	1696791	11854	OXZ27009.1	B9N51_08155	1696272	1696791	-1	hypothetical protein
1703206	1716235	13029		B9N51_08115	1703206	1703397	-1	transcriptional regulator
1703206	1716235	13029	OXZ27030.1	B9N51_08120	1703504	1704148	-1	hypothetical protein
1703206	1716235	13029	OXZ27031.1	B9N51_08125	1704290	1704423	-1	hypothetical protein
1703206	1716235	13029	OXZ27029.1	B9N51_08140	1711588	1712868	1	group II intron reverse transcriptase/maturase
1703206	1716235	13029	OXZ26940.1	B9N51_08200	1715865	1716235	-1	IS200/IS605 family transposase

Table S3J: Predicted genomic islands in the genome of *"F. nericia"* T160124
Shown are genes of genomic islands predicted by IslandViewer4

Island start	Island end	Length	Gene name	Locus	Gene start	Gene end	Strand	Product
507	18470	17963	OXZ26312.1	B9N52_00015	507	1049	1	LemA family protein
507	18470	17963	OXZ26313.1	B9N52_00020	1173	2081	1	coproporphyrinogen III oxidase
507	18470	17963	OXZ26314.1	B9N52_00025	2081	2809	1	biotin--[acetyl-CoA-carboxylase] ligase
507	18470	17963	OXZ26315.1	B9N52_00030	2822	3049	-1	hypothetical protein
507	18470	17963	OXZ26316.1	B9N52_00035	3142	3543	1	transcriptional regulator
507	18470	17963	OXZ26317.1	B9N52_00040	3588	3803	1	heavy metal transporter
507	18470	17963	OXZ26318.1	B9N52_00045	3803	5614	1	cadmium-translocating P-type ATPase
507	18470	17963	OXZ26319.1	B9N52_00050	5661	5972	1	hypothetical protein
507	18470	17963	OXZ26320.1	B9N52_00055	6054	6332	1	damage-inducible protein J
507	18470	17963	OXZ26321.1	B9N52_00060	6431	6688	-1	AbrB family transcriptional regulator
507	18470	17963	OXZ26322.1	B9N52_00065	6792	7367	-1	riboflavin transporter
507	18470	17963	OXZ26323.1	B9N52_00070	7458	11297	1	hypothetical protein
507	18470	17963	OXZ26324.1	B9N52_00075	11319	11442	1	transposase
507	18470	17963	OXZ26274.1	B9N52_00080	12592	13746	-1	2-iminoacetate synthase ThiH
507	18470	17963	OXZ26275.1	B9N52_00085	13754	14518	-1	thiazole synthase
507	18470	17963	OXZ26276.1	B9N52_00090	14508	15104	-1	thiamine biosynthesis protein ThiF
507	18470	17963	OXZ26277.1	B9N52_00095	15106	15303	-1	thiamine biosynthesis protein ThiS
507	18470	17963	OXZ26278.1	B9N52_00100	15524	16135	1	glutathione S-transferase
507	18470	17963	OXZ26279.1	B9N52_00105	16232	16933	1	SAM-dependent methyltransferase
507	18470	17963	OXZ26280.1	B9N52_00110	16950	18470	1	magnesium chelatase
40249	48234	7985	OXZ26299.1	B9N52_00205	38280	40268	1	exopolyphosphatase
40249	48234	7985	OXZ26300.1	B9N52_00210	40249	40695	1	50S ribosomal protein L9
40249	48234	7985	OXZ26301.1	B9N52_00215	40729	42066	1	replicative DNA helicase
40249	48234	7985	OXZ26302.1	B9N52_00220	42063	42626	1	GNAT family N-acetyltransferase
40249	48234	7985	OXZ26303.1	B9N52_00225	42631	43389	1	DNA replication protein
40249	48234	7985	OXZ26304.1	B9N52_00230	43392	44369	1	DNA replication protein DnaC
40249	48234	7985	OXZ26305.1	B9N52_00235	44366	45010	1	NAD(P)H-hydrate epimerase
40249	48234	7985	OXZ26183.1	B9N52_00240	46139	46226	1	hypothetical protein
40249	48234	7985	OXZ26184.1	B9N52_00245	46466	46645	1	hypothetical protein
40249	48234	7985	OXZ26185.1	B9N52_00250	47022	47564	1	ECF transporter S component
40249	48234	7985	OXZ26186.1	B9N52_00255	47548	48234	1	radical SAM protein
498315	512770	14455	OXZ25905.1	B9N52_02425	498315	498785	1	30S ribosomal protein S7
498315	512770	14455	OXZ25906.1	B9N52_02430	498814	500901	1	elongation factor G
498315	512770	14455	OXZ25907.1	B9N52_02435	500913	502106	1	elongation factor Tu
498315	512770	14455	OXZ25908.1	B9N52_02440	502513	505694	1	hypothetical protein
498315	512770	14455	OXZ25792.1	B9N52_02445	506695	509981	1	hypothetical protein
498315	512770	14455	OXZ25793.1	B9N52_02450	510028	510627	1	hypothetical protein
498315	512770	14455	OXZ25794.1	B9N52_02455	511021	512190	-1	IS110 family transposase
498315	512770	14455	OXZ25795.1	B9N52_02460	512477	512770	1	hypothetical protein
506695	512770	6075	OXZ25792.1	B9N52_02445	506695	509981	1	hypothetical protein
506695	512770	6075	OXZ25793.1	B9N52_02450	510028	510627	1	hypothetical protein
506695	512770	6075	OXZ25794.1	B9N52_02455	511021	512190	-1	IS110 family transposase
506695	512770	6075	OXZ25795.1	B9N52_02460	512477	512770	1	hypothetical protein
717980	723912	5932	OXZ25469.1	B9N52_03640	717980	718171	1	50S ribosomal protein L35
717980	723912	5932	OXZ25470.1	B9N52_03645	718190	718543	1	50S ribosomal protein L20
717980	723912	5932	OXZ25471.1	B9N52_03650	718575	718850	-1	hypothetical protein
717980	723912	5932	OXZ25472.1	B9N52_03655	718850	719467	-1	recombinase RecX
717980	723912	5932	OXZ25473.1	B9N52_03660	719656	719895	1	hypothetical protein
717980	723912	5932	OXZ25474.1	B9N52_03665	719904	720980	1	phosphoesterase
717980	723912	5932	OXZ25475.1	B9N52_03670	720977	723229	1	DNA repair protein Rad50
717980	723912	5932	OXZ25476.1	B9N52_03675	723232	723912	1	serine/threonine protein phosphatase
769989	780323	10334	OXZ25521.1	B9N52_03900	769989	770300	1	peptidoglycan-binding protein LysM
769989	780323	10334	OXZ25522.1	B9N52_03905	770376	771356	-1	recombinase XerC
769989	780323	10334		B9N52_03910	771463	772639	-1	aspartate aminotransferase
769989	780323	10334	OXZ25523.1	B9N52_03915	772626	773447	-1	metal-dependent phosphoesterase
769989	780323	10334	OXZ25524.1	B9N52_03920	773516	775057	-1	ribonuclease Y
769989	780323	10334	OXZ25525.1	B9N52_03925	775176	775838	-1	chromosome partitioning protein ParA
769989	780323	10334	OXZ25526.1	B9N52_03930	775825	776064	-1	hypothetical protein
769989	780323	10334	OXZ25527.1	B9N52_03935	776064	777647	-1	exonuclease SbcC
769989	780323	10334	OXZ25684.1	B9N52_03940	777647	778597	-1	phosphoesterase
769989	780323	10334	OXZ25528.1	B9N52_03945	778628	779719	-1	recombinase RecA
769989	780323	10334	OXZ25529.1	B9N52_03950	779787	780323	-1	CDP-diacylglycerol--glycerol-3-phosphate 3-phosphatidyltransferase
1182570	1194616	12046	OXZ25283.1	B9N52_05820	1182570	1182686	-1	tetracycline resistance determinant leader peptide
1182570	1194616	12046	OXZ25284.1	B9N52_05825	1182931	1183866	-1	conjugal transfer protein
1182570	1194616	12046	OXZ25285.1	B9N52_05830	1183863	1184864	-1	peptidase P60
1182570	1194616	12046	OXZ25286.1	B9N52_05835	1184861	1187038	-1	hypothetical protein
1182570	1194616	12046	OXZ25287.1	B9N52_05840	1187041	1189488	-1	ATP/GTP-binding protein
1182570	1194616	12046	OXZ25288.1	B9N52_05845	1189472	1189978	-1	conjugal transfer protein
1182570	1194616	12046	OXZ25289.1	B9N52_05850	1189953	1190450	-1	antirestriction protein ArdA
1182570	1194616	12046	OXZ25290.1	B9N52_05855	1190567	1190788	-1	hypothetical protein
1182570	1194616	12046	OXZ25291.1	B9N52_05860	1190831	1192036	-1	replication initiation protein
1182570	1194616	12046	OXZ25292.1	B9N52_05865	1192059	1192211	-1	conjugal transfer protein
1182570	1194616	12046	OXZ25293.1	B9N52_05870	1192214	1193599	-1	ATP-binding protein
1182570	1194616	12046	OXZ25294.1	B9N52_05875	1193628	1194014	-1	conjugal transfer protein
1182570	1194616	12046	OXZ25295.1	B9N52_05880	1194030	1194344	-1	conjugal transfer protein
1182570	1194616	12046	OXZ25296.1	B9N52_05885	1194529	1194616	-1	hypothetical protein
1215800	1221814	6014	OXZ25202.1	B9N52_05970	1215800	1216666	-1	hypothetical protein
1215800	1221814	6014	OXZ25179.1	B9N52_05975	1217667	1218396	-1	hypothetical protein
1215800	1221814	6014	OXZ25180.1	B9N52_05980	1218662	1220110	-1	hypothetical protein
1215800	1221814	6014	OXZ25181.1	B9N52_05985	1220372	1221814	-1	hypothetical protein
1385415	1465266	79851	OXZ25081.1	B9N52_06785	1385415	1385762	-1	hypothetical protein

1385415	1465266	79851		B9N52_06790	1385852	1386129	-1	hypothetical protein
1385415	1465266	79851	OXZ25076.1	B9N52_06795	1386361	1386897	-1	Rossman fold protein, TIGR00730 family
1385415	1465266	79851	OXZ25077.1	B9N52_06800	1387008	1388005	-1	macrolide ABC transporter ATP-binding protein
1385415	1465266	79851	OXZ25019.1	B9N52_06805	1389211	1389657	-1	transposase
1385415	1465266	79851	OXZ25020.1	B9N52_06810	1389736	1391862	-1	catalase HPII
1385415	1465266	79851	OXZ25021.1	B9N52_06815	1392350	1392841	-1	protein-S-isoprenylcysteine methyltransferase
1385415	1465266	79851	OXZ25022.1	B9N52_06820	1392855	1393793	-1	esterase
1385415	1465266	79851	OXZ25023.1	B9N52_06825	1393862	1394488	-1	hypothetical protein
1385415	1465266	79851	OXZ25024.1	B9N52_06830	1394516	1395124	-1	SAM-dependent methyltransferase
1385415	1465266	79851	OXZ25025.1	B9N52_06835	1395340	1396089	-1	phosphorylase
1385415	1465266	79851	OXZ25026.1	B9N52_06840	1396219	1396576	-1	macrolide ABC transporter ATP-binding protein
1385415	1465266	79851	OXZ25018.1	B9N52_06845	1397577	1398145	-1	macrolide ABC transporter ATP-binding protein
1385415	1465266	79851	OXZ25017.1	B9N52_06850	1399146	1399876	-1	macrolide ABC transporter ATP-binding protein
1385415	1465266	79851	OXZ24933.1	B9N52_06855	1401405	1402019	-1	hypothetical protein
1385415	1465266	79851	OXZ24934.1	B9N52_06860	1402226	1403590	-1	ABC transporter ATP-binding protein
1385415	1465266	79851	OXZ24935.1	B9N52_06865	1403578	1404273	-1	cobalt ABC transporter permease
1385415	1465266	79851	OXZ24936.1	B9N52_06870	1404273	1404854	-1	hypothetical protein
1385415	1465266	79851	OXZ24937.1	B9N52_06875	1404914	1406257	-1	MATE family efflux transporter
1385415	1465266	79851	OXZ24938.1	B9N52_06880	1406269	1407990	-1	ABC transporter ATP-binding protein
1385415	1465266	79851	OXZ24939.1	B9N52_06885	1407974	1409728	-1	ABC transporter ATP-binding protein
1385415	1465266	79851	OXZ24940.1	B9N52_06890	1409889	1410881	1	AraC family transcriptional regulator
1385415	1465266	79851	OXZ24941.1	B9N52_06895	1411661	1412719	-1	arsenical-resistance protein
1385415	1465266	79851	OXZ24942.1	B9N52_06900	1412750	1414501	-1	arsenical pump-driving ATPase
1385415	1465266	79851	OXZ24943.1	B9N52_06905	1414520	1414885	-1	arsenical resistance operon transcriptional repressor ArsD
1385415	1465266	79851	OXZ24944.1	B9N52_06910	1414905	1415219	-1	transcriptional regulator
1385415	1465266	79851	OXZ24945.1	B9N52_06915	1415958	1416413	1	penicillinase repressor
1385415	1465266	79851		B9N52_06920	1416427	1418580	1	heavy metal translocating P-type ATPase
1385415	1465266	79851	OXZ24946.1	B9N52_06925	1419254	1419859	-1	cadmium resistance protein CadD
1385415	1465266	79851		B9N52_06930	1420153	1420370	-1	hypothetical protein
1385415	1465266	79851	OXZ24947.1	B9N52_06935	1420665	1421963	-1	AAA family ATPase
1385415	1465266	79851		B9N52_06940	1422588	1422925	-1	hypothetical protein
1385415	1465266	79851	OXZ24948.1	B9N52_06945	1423188	1423445	-1	hypothetical protein
1385415	1465266	79851	OXZ24949.1	B9N52_06950	1423468	1425903	-1	ABC transporter permease
1385415	1465266	79851	OXZ24950.1	B9N52_06955	1425896	1426576	-1	peptide ABC transporter ATP-binding protein
1385415	1465266	79851	OXZ24951.1	B9N52_06960	1426623	1427483	-1	two-component sensor histidine kinase
1385415	1465266	79851	OXZ24952.1	B9N52_06965	1427485	1428135	-1	DNA-binding response regulator
1385415	1465266	79851		B9N52_06970	1428709	1429124	-1	RNA polymerase subunit sigma
1385415	1465266	79851	OXZ24953.1	B9N52_06975	1429436	1430788	-1	MATE family efflux transporter
1385415	1465266	79851	OXZ24954.1	B9N52_06980	1430815	1432560	-1	ABC transporter permease
1385415	1465266	79851	OXZ24955.1	B9N52_06985	1432553	1434343	-1	ABC transporter ATP-binding protein
1385415	1465266	79851	OXZ24956.1	B9N52_06990	1434333	1435802	-1	ABC transporter
1385415	1465266	79851	OXZ24957.1	B9N52_06995	1435787	1436500	-1	ABC transporter permease
1385415	1465266	79851	OXZ24958.1	B9N52_07000	1436490	1437086	-1	hypothetical protein
1385415	1465266	79851	OXZ25016.1	B9N52_07005	1437210	1437848	-1	TetR family transcriptional regulator
1385415	1465266	79851	OXZ24959.1	B9N52_07010	1437920	1439482	-1	recombinase family protein
1385415	1465266	79851	OXZ24960.1	B9N52_07015	1439475	1439891	-1	integrase
1385415	1465266	79851	OXZ24961.1	B9N52_07020	1439884	1441446	-1	recombinase family protein
1385415	1465266	79851	OXZ24962.1	B9N52_07025	1441506	1441715	-1	hypothetical protein
1385415	1465266	79851	OXZ24963.1	B9N52_07030	1442081	1443817	-1	multidrug ABC transporter ATP-binding protein
1385415	1465266	79851	OXZ24964.1	B9N52_07035	1443810	1445531	-1	ABC transporter ATP-binding protein
1385415	1465266	79851	OXZ24965.1	B9N52_07040	1445569	1446921	-1	MATE family efflux transporter
1385415	1465266	79851	OXZ24966.1	B9N52_07045	1446934	1448382	-1	ABC transporter
1385415	1465266	79851	OXZ24967.1	B9N52_07050	1448379	1449068	-1	ABC transporter permease
1385415	1465266	79851	OXZ24968.1	B9N52_07055	1449068	1449646	-1	hypothetical protein
1385415	1465266	79851	OXZ24969.1	B9N52_07060	1449698	1450270	-1	TetR family transcriptional regulator
1385415	1465266	79851	OXZ24970.1	B9N52_07065	1450563	1450919	1	plasmid mobilization relaxosome protein MobC
1385415	1465266	79851		B9N52_07070	1450921	1452251	1	endonuclease
1385415	1465266	79851	OXZ24971.1	B9N52_07075	1452255	1453496	-1	DNA (cytosine-5-)-methyltransferase
1385415	1465266	79851	OXZ24972.1	B9N52_07080	1453493	1454986	-1	hypothetical protein
1385415	1465266	79851	OXZ24973.1	B9N52_07085	1454976	1457147	-1	hypothetical protein
1385415	1465266	79851	OXZ24974.1	B9N52_07090	1457198	1457419	-1	transcriptional regulator
1385415	1465266	79851		B9N52_07095	1457704	1458141	-1	hypothetical protein
1385415	1465266	79851	OXZ24975.1	B9N52_07100	1458874	1459461	-1	transcriptional regulator
1385415	1465266	79851	OXZ24976.1	B9N52_07105	1459959	1461173	-1	23S rRNA (uracil(1939)-C(5))-methyltransferase RlmD
1385415	1465266	79851	OXZ24977.1	B9N52_07110	1461266	1463062	-1	ABC transporter
1385415	1465266	79851	OXZ24978.1	B9N52_07115	1463055	1464815	-1	multidrug ABC transporter ATP-binding protein
1385415	1465266	79851	OXZ24979.1	B9N52_07120	1464790	1465266	-1	MarR family transcriptional regulator
1399146	1406257	7111	OXZ25017.1	B9N52_06850	1399146	1399876	-1	macrolide ABC transporter ATP-binding protein
1399146	1406257	7111	OXZ24933.1	B9N52_06855	1401405	1402019	-1	hypothetical protein
1399146	1406257	7111	OXZ24934.1	B9N52_06860	1402226	1403590	-1	ABC transporter ATP-binding protein
1399146	1406257	7111	OXZ24935.1	B9N52_06865	1403578	1404273	-1	cobalt ABC transporter permease
1399146	1406257	7111	OXZ24936.1	B9N52_06870	1404273	1404854	-1	hypothetical protein
1399146	1406257	7111	OXZ24937.1	B9N52_06875	1404914	1406257	-1	MATE family efflux transporter
1420153	1425903	5750		B9N52_06930	1420153	1420370	-1	hypothetical protein
1420153	1425903	5750	OXZ24947.1	B9N52_06935	1420665	1421963	-1	AAA family ATPase
1420153	1425903	5750		B9N52_06940	1422588	1422925	-1	hypothetical protein
1420153	1425903	5750	OXZ24948.1	B9N52_06945	1423188	1423445	-1	hypothetical protein
1420153	1425903	5750	OXZ24949.1	B9N52_06950	1423468	1425903	-1	ABC transporter permease

1420153	1425903	5750	OXZ24950.1	B9N52_06955	1425896	1426576	-1	peptide ABC transporter ATP-binding protein
1441506	1450919	9413	OXZ24962.1	B9N52_07025	1441506	1441715	-1	hypothetical protein
1441506	1450919	9413	OXZ24963.1	B9N52_07030	1442081	1443817	-1	multidrug ABC transporter ATP-binding protein
1441506	1450919	9413	OXZ24964.1	B9N52_07035	1443810	1445531	-1	ABC transporter ATP-binding protein
1441506	1450919	9413	OXZ24965.1	B9N52_07040	1445569	1446921	-1	MATE family efflux transporter
1441506	1450919	9413	OXZ24966.1	B9N52_07045	1446934	1448382	-1	ABC transporter
1441506	1450919	9413	OXZ24967.1	B9N52_07050	1448379	1449068	-1	ABC transporter permease
1441506	1450919	9413	OXZ24968.1	B9N52_07055	1449068	1449646	-1	hypothetical protein
1441506	1450919	9413	OXZ24969.1	B9N52_07060	1449698	1450270	-1	TetR family transcriptional regulator
1441506	1450919	9413	OXZ24970.1	B9N52_07065	1450563	1450919	1	plasmid mobilization relaxosome protein MobC
1771981	1789695	17714	OXZ24924.1	B9N52_08540	1771981	1773342	1	hypothetical protein
1771981	1789695	17714	OXZ24684.1	B9N52_08575	1774499	1775197	-1	hypothetical protein
1771981	1789695	17714	OXZ24685.1	B9N52_08580	1775356	1775895	-1	transposase
1771981	1789695	17714	OXZ24682.1	B9N52_08585	1784312	1784851	1	transposase
1771981	1789695	17714	OXZ24683.1	B9N52_08590	1784914	1785708	1	hypothetical protein
1771981	1789695	17714	OXZ24688.1	B9N52_08545	1788311	1789105	-1	transposase
1771981	1789695	17714	OXZ24689.1	B9N52_08550	1789129	1789695	-1	transposase
1774499	1789105	14606	OXZ24684.1	B9N52_08575	1774499	1775197	-1	hypothetical protein
1774499	1789105	14606	OXZ24685.1	B9N52_08580	1775356	1775895	-1	transposase
1774499	1789105	14606	OXZ24682.1	B9N52_08585	1784312	1784851	1	transposase
1774499	1789105	14606	OXZ24683.1	B9N52_08590	1784914	1785708	1	hypothetical protein
1774499	1789105	14606	OXZ24688.1	B9N52_08545	1788311	1789105	-1	transposase

Table S3K: Predicted genomic islands in the genome of *"F. nericia"* ATCC53516

Shown are genes of genomic islands predicted by IslandViewer4

Island start	Island end	Length	Gene name	Locus	Gene start	Gene end	Strand	Product
235768	246338	10570	EFH93375.1	HMPREF0391_11033	235768	236637	1	hypothetical protein
235768	246338	10570	EFH93374.1	HMPREF0391_11032	236624	238612	1	DHHA1 domain protein
235768	246338	10570	EFH93373.1	HMPREF0391_11031	238593	239039	1	ribosomal protein L9
235768	246338	10570	EFH93372.1	HMPREF0391_11030	239073	240410	1	replicative DNA helicase
235768	246338	10570	EFH93371.1	HMPREF0391_11029	240407	240970	1	acetyltransferase, GNAT family
235768	246338	10570	EFH93370.1	HMPREF0391_11028	240954	241733	1	DnaD domain protein
235768	246338	10570	EFH93369.1	HMPREF0391_11027	241736	242713	1	DNA replication protein DnaC
235768	246338	10570	EFH93368.1	HMPREF0391_11026	242710	243354	1	YjeF-like protein
235768	246338	10570	EFH93367.1	HMPREF0391_11025	244071	244613	1	hypothetical protein
235768	246338	10570	EFH93366.1	HMPREF0391_11024	244597	245283	1	hypothetical protein
235768	246338	10570	EFH93365.1	HMPREF0391_11023	245337	246053	1	putative phage head-tail adaptor
235768	246338	10570	EFH93364.1	HMPREF0391_11022	246111	246338	1	hypothetical protein
294189	302405	8216	EFH93316.1	HMPREF0391_10974	294189	294389	1	DNA-binding helix-turn-helix protein
294189	302405	8216	EFH93315.1	HMPREF0391_10973	294390	294839	1	hypothetical protein
294189	302405	8216	EFH93314.1	HMPREF0391_10972	294976	295641	1	hypothetical protein
294189	302405	8216	EFH93313.1	HMPREF0391_10971	295802	296746	1	DnaJ domain protein
294189	302405	8216	EFH93312.1	HMPREF0391_10970	296765	299350	1	ATP-dependent chaperone protein ClpB
294189	302405	8216	EFH93311.1	HMPREF0391_10969	299626	300015	-1	hypothetical protein
294189	302405	8216	EFH93310.1	HMPREF0391_10968	300091	300954	1	UTP--glucose-1-phosphate uridylyltransferase
294189	302405	8216	EFH93309.1	HMPREF0391_10967	301037	301732	1	response regulator receiver domain protein
294189	302405	8216	EFH93308.1	HMPREF0391_10966	301740	302405	1	hypothetical protein
349541	354034	4493	EFH93269.1	HMPREF0391_10927	349438	349557	1	hypothetical protein
349541	354034	4493	EFH93268.1	HMPREF0391_10926	349541	349756	1	hypothetical protein
349541	354034	4493	EFH93267.1	HMPREF0391_10925	352820	354034	-1	transposase, Mutator family
494767	499382	4615	EFH93134.1	HMPREF0391_10792	494767	494967	1	hypothetical protein
494767	499382	4615	EFH93133.1	HMPREF0391_10791	495132	496514	-1	domain protein
494767	499382	4615	EFH93132.1	HMPREF0391_10790	496505	497164	-1	response regulator receiver domain protein
494767	499382	4615	EFH93131.1	HMPREF0391_10789	497197	497964	-1	antibiotic ABC transporter protein MutG
494767	499382	4615	EFH93130.1	HMPREF0391_10788	497937	498677	-1	mutacin ABC transporter protein, MutE
494767	499382	4615	EFH93129.1	HMPREF0391_10787	498681	499382	-1	MutF
971218	980556	9338	EFH92519.1	HMPREF0391_11491	971218	972198	-1	site-specific tyrosine recombinase XerC
971218	980556	9338	EFH92520.1	HMPREF0391_11492	972301	973476	-1	aminotransferase, class I/II
971218	980556	9338	EFH92521.1	HMPREF0391_11493	973463	974284	-1	PHP domain protein
971218	980556	9338	EFH92522.1	HMPREF0391_11494	974353	975894	-1	YmdA/YtgF family protein
971218	980556	9338	EFH92523.1	HMPREF0391_11495	976013	976675	-1	hypothetical protein
971218	980556	9338	EFH92524.1	HMPREF0391_11496	976662	976901	-1	hypothetical protein
971218	980556	9338	EFH92525.1	HMPREF0391_11497	976901	978484	-1	hypothetical protein
971218	980556	9338	EFH92526.1	HMPREF0391_11498	978484	979437	-1	phosphodiesterase family protein
971218	980556	9338	EFH92527.1	HMPREF0391_11499	979465	980556	-1	RecA protein
1135806	1139907	4101	EFH92677.1	HMPREF0391_11649	1135806	1136735	-1	ABC transporter, permease protein
1135806	1139907	4101	EFH92678.1	HMPREF0391_11650	1136737	1137441	-1	TOBE domain protein
1135806	1139907	4101	EFH92679.1	HMPREF0391_11651	1137658	1138644	1	integrase core domain protein
1135806	1139907	4101	EFH92680.1	HMPREF0391_11652	1138613	1138924	-1	protein
1135806	1139907	4101	EFH92681.1	HMPREF0391_11653	1139134	1139907	1	transcriptional regulator, DeoR family
1172805	1217320	44515	EFH92708.1	HMPREF0391_11680	1172805	1173215	-1	hypothetical protein
1172805	1217320	44515	EFH92709.1	HMPREF0391_11681	1173535	1174884	-1	MATE efflux family protein
1172805	1217320	44515	EFH92710.1	HMPREF0391_11682	1174921	1176654	-1	ABC transporter, ATP-binding protein
1172805	1217320	44515	EFH92711.1	HMPREF0391_11683	1176651	1178393	-1	ABC transporter, ATP-binding protein
1172805	1217320	44515	EFH92712.1	HMPREF0391_11684	1178429	1179820	-1	ABC transporter, ATP-binding protein
1172805	1217320	44515	EFH92713.1	HMPREF0391_11685	1179813	1180514	-1	cobalt transport protein
1172805	1217320	44515	EFH92714.1	HMPREF0391_11686	1180539	1181120	-1	conserved hypothetical protein TIGR02185
1172805	1217320	44515	EFH92715.1	HMPREF0391_11687	1181138	1181749	-1	transcriptional regulator, TetR family
1172805	1217320	44515	EFH92716.1	HMPREF0391_11688	1181938	1182417	1	hypothetical protein
1172805	1217320	44515	EFH92717.1	HMPREF0391_11689	1182419	1183750	1	relaxase/mobilization nuclease domain protein
1172805	1217320	44515	EFH92718.1	HMPREF0391_11690	1183771	1184268	-1	hypothetical protein
1172805	1217320	44515	EFH92719.1	HMPREF0391_11691	1184269	1185690	-1	hypothetical protein
1172805	1217320	44515	EFH92720.1	HMPREF0391_11692	1185725	1185940	-1	hypothetical protein
1172805	1217320	44515	EFH92721.1	HMPREF0391_11693	1186041	1186274	-1	hypothetical protein
1172805	1217320	44515	EFH92722.1	HMPREF0391_11694	1186387	1187049	-1	hypothetical protein
1172805	1217320	44515	EFH92723.1	HMPREF0391_11695	1187065	1194735	-1	helicase C-terminal domain protein
1172805	1217320	44515	EFH92724.1	HMPREF0391_11696	1194818	1197031	-1	hypothetical protein
1172805	1217320	44515	EFH92725.1	HMPREF0391_11697	1197067	1198029	-1	DNA (cytosine-5-)-methyltransferase
1172805	1217320	44515	EFH92726.1	HMPREF0391_11698	1198022	1199755	-1	DNA topoisomerase
1172805	1217320	44515	EFH92727.1	HMPREF0391_11699	1199807	1200673	-1	hypothetical protein
1172805	1217320	44515	EFH92728.1	HMPREF0391_11700	1200688	1201758	-1	copper amine oxidase domain protein
1172805	1217320	44515	EFH92729.1	HMPREF0391_11701	1201839	1203038	-1	hypothetical protein
1172805	1217320	44515	EFH92730.1	HMPREF0391_11702	1203025	1203264	-1	hypothetical protein
1172805	1217320	44515	EFH92731.1	HMPREF0391_11703	1203354	1204529	-1	NlpC/P60 family protein
1172805	1217320	44515	EFH92732.1	HMPREF0391_11704	1204692	1204973	-1	hypothetical protein
1172805	1217320	44515	EFH92733.1	HMPREF0391_11705	1205024	1205908	-1	transposase, Mutator family
1172805	1217320	44515	EFH92734.1	HMPREF0391_11706	1205958	1207439	-1	hypothetical protein
1172805	1217320	44515	EFH92735.1	HMPREF0391_11707	1207447	1209870	-1	hypothetical protein
1172805	1217320	44515	EFH92736.1	HMPREF0391_11708	1209761	1210171	-1	hypothetical protein
1172805	1217320	44515	EFH92737.1	HMPREF0391_11709	1210173	1210463	-1	hypothetical protein
1172805	1217320	44515	EFH92738.1	HMPREF0391_11710	1210473	1211336	-1	hypothetical protein
1172805	1217320	44515	EFH92739.1	HMPREF0391_11711	1211347	1211562	-1	hypothetical protein
1172805	1217320	44515	EFH92740.1	HMPREF0391_11712	1211564	1211875	-1	hypothetical protein
1172805	1217320	44515	EFH92741.1	HMPREF0391_11713	1212066	1212341	-1	hypothetical protein

1172805	1217320	44515	EFH92742.1	HMPREF0391_11714	1212360	1214156	-1	TraG/TraD family protein
1172805	1217320	44515	EFH92743.1	HMPREF0391_11715	1214149	1214883	-1	BRO family, N-terminal domain protein
1172805	1217320	44515	EFH92744.1	HMPREF0391_11716	1214880	1215365	-1	hypothetical protein
1172805	1217320	44515	EFH92745.1	HMPREF0391_11717	1215358	1216185	-1	chromosomal replication initiator protein DnaA
1172805	1217320	44515	EFH92746.1	HMPREF0391_11718	1216182	1216955	-1	replication initiator protein A domain protein
1172805	1217320	44515	EFH92747.1	HMPREF0391_11719	1217039	1217320	-1	hypothetical protein
1203025	1207439	4414	EFH92729.1	HMPREF0391_11701	1201839	1203038	-1	hypothetical protein
1203025	1207439	4414	EFH92730.1	HMPREF0391_11702	1203025	1203264	-1	hypothetical protein
1203025	1207439	4414	EFH92731.1	HMPREF0391_11703	1203354	1204529	-1	NlpC/P60 family protein
1203025	1207439	4414	EFH92732.1	HMPREF0391_11704	1204692	1204973	-1	hypothetical protein
1203025	1207439	4414	EFH92733.1	HMPREF0391_11705	1205024	1205908	-1	transposase, Mutator family
1203025	1207439	4414	EFH92734.1	HMPREF0391_11706	1205958	1207439	-1	hypothetical protein
1211564	1217320	5756	EFH92740.1	HMPREF0391_11712	1211564	1211875	-1	hypothetical protein
1211564	1217320	5756	EFH92741.1	HMPREF0391_11713	1212066	1212341	-1	hypothetical protein
1211564	1217320	5756	EFH92742.1	HMPREF0391_11714	1212360	1214156	-1	TraG/TraD family protein
1211564	1217320	5756	EFH92743.1	HMPREF0391_11715	1214149	1214883	-1	BRO family, N-terminal domain protein
1211564	1217320	5756	EFH92744.1	HMPREF0391_11716	1214880	1215365	-1	hypothetical protein
1211564	1217320	5756	EFH92745.1	HMPREF0391_11717	1215358	1216185	-1	chromosomal replication initiator protein DnaA
1211564	1217320	5756	EFH92746.1	HMPREF0391_11718	1216182	1216955	-1	replication initiator protein A domain protein
1211564	1217320	5756	EFH92747.1	HMPREF0391_11719	1217039	1217320	-1	hypothetical protein
1349537	1383336	33799	EFH93638.1	HMPREF0391_10006	1348897	1349553	-1	chain length determinant protein
1349537	1383336	33799	EFH93639.1	HMPREF0391_10007	1349537	1350316	-1	hypothetical protein
1349537	1383336	33799	EFH93640.1	HMPREF0391_10008	1350317	1350739	-1	SH3 domain protein
1349537	1383336	33799	EFH93641.1	HMPREF0391_10009	1350920	1351444	1	topology modulation protein
1349537	1383336	33799	EFH93642.1	HMPREF0391_10010	1351534	1352748	1	transposase, Mutator family
1349537	1383336	33799	EFH93643.1	HMPREF0391_10011	1353139	1361259	-1	LPXTG-motif cell wall anchor domain protein
1349537	1383336	33799	EFH93644.1	HMPREF0391_10012	1362088	1363713	-1	LPXTG-motif cell wall anchor domain protein
1349537	1383336	33799	EFH93645.1	HMPREF0391_10013	1363706	1363873	1	hypothetical protein
1349537	1383336	33799	EFH93646.1	HMPREF0391_10014	1367492	1367641	-1	hypothetical protein
1349537	1383336	33799	EFH93647.1	HMPREF0391_10015	1367678	1371538	-1	hypothetical protein
1349537	1383336	33799	EFH93648.1	HMPREF0391_10016	1371646	1371777	-1	hypothetical protein
1349537	1383336	33799	EFH93649.1	HMPREF0391_10017	1373225	1375135	-1	LPXTG-motif cell wall anchor domain protein
1349537	1383336	33799	EFH93650.1	HMPREF0391_10018	1375655	1375777	-1	hypothetical protein
1349537	1383336	33799	EFH93651.1	HMPREF0391_10019	1375808	1376242	-1	acetyltransferase, GNAT family
1349537	1383336	33799	EFH93652.1	HMPREF0391_10020	1376265	1376963	-1	putative TIGR02206 family protein
1349537	1383336	33799	EFH93653.1	HMPREF0391_10021	1376963	1377682	-1	hypothetical protein
1349537	1383336	33799	EFH93654.1	HMPREF0391_10022	1378623	1381976	-1	efflux ABC transporter, permease protein
1349537	1383336	33799	EFH93655.1	HMPREF0391_10023	1381978	1382679	-1	ABC transporter, ATP-binding protein
1349537	1383336	33799	EFH93656.1	HMPREF0391_10024	1382791	1383336	1	transcriptional regulator, TetR family
1431249	1529242	97993	EFH93711.1	HMPREF0391_10079	1431249	1431857	-1	methyltransferase domain protein
1431249	1529242	97993	EFH93712.1	HMPREF0391_10080	1431960	1432244	-1	hypothetical protein
1431249	1529242	97993	EFH93713.1	HMPREF0391_10081	1432455	1432946	-1	hypothetical protein
1431249	1529242	97993	EFH93714.1	HMPREF0391_10082	1433194	1433943	-1	hypothetical protein
1431249	1529242	97993	EFH93715.1	HMPREF0391_10083	1434203	1435807	-1	ABC transporter, ATP-binding protein
1431249	1529242	97993	EFH93716.1	HMPREF0391_10084	1436535	1437149	-1	hypothetical protein
1431249	1529242	97993	EFH93717.1	HMPREF0391_10085	1437358	1438722	-1	ABC transporter, ATP-binding protein
1431249	1529242	97993	EFH93718.1	HMPREF0391_10086	1438716	1439405	-1	cobalt transport protein
1431249	1529242	97993	EFH93719.1	HMPREF0391_10087	1439405	1439989	-1	conserved hypothetical protein TIGR02185
1431249	1529242	97993	EFH93720.1	HMPREF0391_10088	1440049	1441407	-1	MATE efflux family protein
1431249	1529242	97993	EFH93721.1	HMPREF0391_10089	1441404	1443125	-1	ABC transporter, ATP-binding protein
1431249	1529242	97993	EFH93722.1	HMPREF0391_10090	1443109	1444869	-1	ABC transporter, ATP-binding protein
1431249	1529242	97993	EFH93723.1	HMPREF0391_10091	1445024	1446016	1	transcriptional regulator, AraC family
1431249	1529242	97993	EFH93724.1	HMPREF0391_10092	1446278	1446817	1	transposase
1431249	1529242	97993	EFH93725.1	HMPREF0391_10093	1447012	1447716	1	integrase core domain protein
1431249	1529242	97993	EFH93726.1	HMPREF0391_10094	1447946	1449160	-1	transposase, Mutator family
1431249	1529242	97993	EFH93727.1	HMPREF0391_10095	1449521	1450117	-1	hypothetical protein
1431249	1529242	97993	EFH93728.1	HMPREF0391_10096	1450260	1452002	-1	ABC transporter, ATP-binding protein
1431249	1529242	97993	EFH93729.1	HMPREF0391_10097	1451989	1453710	-1	ABC transporter, ATP-binding protein
1431249	1529242	97993	EFH93730.1	HMPREF0391_10098	1453710	1455173	-1	ABC transporter, ATP-binding protein
1431249	1529242	97993	EFH93731.1	HMPREF0391_10099	1455175	1455846	-1	cobalt transport protein
1431249	1529242	97993	EFH93732.1	HMPREF0391_10100	1455843	1456433	-1	conserved hypothetical protein TIGR02185
1431249	1529242	97993	EFH93733.1	HMPREF0391_10101	1456500	1457477	-1	transcriptional regulator, AraC family
1431249	1529242	97993	EFH93734.1	HMPREF0391_10102	1457487	1458089	-1	transcriptional regulator, TetR family
1431249	1529242	97993	EFH93735.1	HMPREF0391_10103	1458205	1458381	-1	hypothetical protein
1431249	1529242	97993	EFH93736.1	HMPREF0391_10104	1458528	1458791	1	hypothetical protein
1431249	1529242	97993	EFH93737.1	HMPREF0391_10105	1459309	1459572	-1	hypothetical protein
1431249	1529242	97993	EFH93738.1	HMPREF0391_10106	1459749	1460726	-1	caspase domain protein
1431249	1529242	97993	EFH93739.1	HMPREF0391_10107	1460746	1461123	-1	hypothetical protein
1431249	1529242	97993	EFH93740.1	HMPREF0391_10108	1461126	1461293	-1	hypothetical protein
1431249	1529242	97993	EFH93741.1	HMPREF0391_10109	1461517	1461984	-1	hypothetical protein
1431249	1529242	97993	EFH93742.1	HMPREF0391_10110	1462046	1462837	-1	CAAX amino terminal protease family protein
1431249	1529242	97993	EFH93743.1	HMPREF0391_10111	1463678	1464328	-1	hypothetical protein
1431249	1529242	97993	EFH93744.1	HMPREF0391_10112	1464635	1465384	-1	hypothetical protein
1431249	1529242	97993	EFH93745.1	HMPREF0391_10113	1465957	1467390	-1	hypothetical protein
1431249	1529242	97993	EFH93746.1	HMPREF0391_10114	1467431	1467751	-1	hypothetical protein
1431249	1529242	97993	EFH93747.1	HMPREF0391_10115	1467931	1468203	-1	hypothetical protein
1431249	1529242	97993	EFH93748.1	HMPREF0391_10116	1468489	1469475	1	integrase core domain protein
1431249	1529242	97993	EFH93749.1	HMPREF0391_10117	1469778	1471130	-1	MATE efflux family protein
1431249	1529242	97993	EFH93750.1	HMPREF0391_10118	1471157	1472902	-1	ABC transporter, ATP-binding protein
1431249	1529242	97993	EFH93751.1	HMPREF0391_10119	1472895	1474688	-1	ABC transporter, ATP-binding protein
1431249	1529242	97993	EFH93752.1	HMPREF0391_10120	1474675	1476144	-1	ABC transporter, ATP-binding protein
1431249	1529242	97993	EFH93753.1	HMPREF0391_10121	1476129	1476842	-1	cobalt transport protein

1431249	1529242	97993	EFH93754.1	HMPREF0391_10122	1476832	1477428	-1	conserved hypothetical protein TIGR02185
1431249	1529242	97993	EFH93755.1	HMPREF0391_10123	1477552	1478271	-1	transcriptional regulator, TetR family
1431249	1529242	97993	EFH93756.1	HMPREF0391_10124	1478262	1479827	-1	resolvase, N-terminal domain protein
1431249	1529242	97993	EFH93757.1	HMPREF0391_10125	1479817	1480233	-1	recombinase
1431249	1529242	97993	EFH93758.1	HMPREF0391_10126	1480226	1481788	-1	resolvase, N-terminal domain protein
1431249	1529242	97993	EFH93759.1	HMPREF0391_10127	1481848	1482057	-1	hypothetical protein
1431249	1529242	97993	EFH93760.1	HMPREF0391_10128	1482421	1484157	-1	ABC transporter, ATP-binding protein
1431249	1529242	97993	EFH93761.1	HMPREF0391_10129	1484150	1485889	-1	ABC transporter, ATP-binding protein
1431249	1529242	97993	EFH93762.1	HMPREF0391_10130	1485873	1486496	-1	transcriptional regulator, TetR family
1431249	1529242	97993	EFH93763.1	HMPREF0391_10131	1486640	1487449	-1	Cpl-7 lysozyme C-terminal domain protein
1431249	1529242	97993	EFH93764.1	HMPREF0391_10132	1487450	1487851	-1	toxin secretion/phage lysis holin
1431249	1529242	97993	EFH93765.1	HMPREF0391_10133	1487853	1489232	-1	hypothetical protein
1431249	1529242	97993	EFH93766.1	HMPREF0391_10134	1489219	1491090	-1	hypothetical protein
1431249	1529242	97993	EFH93767.1	HMPREF0391_10135	1491090	1491452	-1	hypothetical protein
1431249	1529242	97993	EFH93768.1	HMPREF0391_10136	1491452	1492948	-1	TMP repeat protein
1431249	1529242	97993	EFH93769.1	HMPREF0391_10137	1492818	1494518	-1	phage tail tape measure protein, TP901 family
1431249	1529242	97993	EFH93770.1	HMPREF0391_10138	1494581	1494907	-1	hypothetical protein
1431249	1529242	97993	EFH93771.1	HMPREF0391_10139	1494946	1495110	-1	hypothetical protein
1431249	1529242	97993	EFH93772.1	HMPREF0391_10140	1495128	1495484	-1	hypothetical protein
1431249	1529242	97993	EFH93773.1	HMPREF0391_10141	1495487	1496083	-1	phage major tail protein, phi13 family
1431249	1529242	97993	EFH93774.1	HMPREF0391_10142	1496073	1496420	-1	hypothetical protein
1431249	1529242	97993	EFH93775.1	HMPREF0391_10143	1496413	1496793	-1	hypothetical protein
1431249	1529242	97993	EFH93776.1	HMPREF0391_10144	1496790	1497122	-1	putative phage head-tail adaptor
1431249	1529242	97993	EFH93777.1	HMPREF0391_10145	1497122	1497394	-1	phage DNA packaging protein
1431249	1529242	97993	EFH93778.1	HMPREF0391_10146	1497405	1498595	-1	phage major capsid protein, HK97 family
1431249	1529242	97993	EFH93779.1	HMPREF0391_10147	1498598	1499284	-1	endopeptidase Clp
1431249	1529242	97993	EFH93780.1	HMPREF0391_10148	1499271	1500548	-1	phage portal protein, HK97 family
1431249	1529242	97993	EFH93781.1	HMPREF0391_10149	1500593	1501012	-1	hypothetical protein
1431249	1529242	97993	EFH93782.1	HMPREF0391_10150	1501057	1502646	-1	putative phage terminase, large subunit
1431249	1529242	97993	EFH93783.1	HMPREF0391_10151	1502742	1503062	-1	hypothetical protein
1431249	1529242	97993	EFH93784.1	HMPREF0391_10152	1503123	1503518	-1	hypothetical protein
1431249	1529242	97993	EFH93785.1	HMPREF0391_10153	1503563	1503778	-1	hypothetical protein
1431249	1529242	97993	EFH93786.1	HMPREF0391_10154	1503822	1505072	-1	DNA (cytosine-5-)-methyltransferase
1431249	1529242	97993	EFH93787.1	HMPREF0391_10155	1505072	1505854	-1	hypothetical protein
1431249	1529242	97993	EFH93788.1	HMPREF0391_10156	1505858	1506370	-1	HNH endonuclease domain protein
1431249	1529242	97993	EFH93789.1	HMPREF0391_10157	1506380	1507027	-1	hypothetical protein
1431249	1529242	97993	EFH93790.1	HMPREF0391_10158	1507081	1507479	-1	HNH endonuclease domain protein
1431249	1529242	97993	EFH93791.1	HMPREF0391_10159	1507626	1507883	1	transcriptional regulator, AbrB family
1431249	1529242	97993	EFH93792.1	HMPREF0391_10160	1507883	1508149	1	addiction module toxin, RelE/StbE family
1431249	1529242	97993	EFH93793.1	HMPREF0391_10161	1508226	1508627	-1	hypothetical protein
1431249	1529242	97993	EFH93794.1	HMPREF0391_10162	1508675	1510018	-1	type III restriction enzyme, res subunit
1431249	1529242	97993	EFH93795.1	HMPREF0391_10163	1510019	1510216	-1	VRR-NUC domain protein
1431249	1529242	97993	EFH93796.1	HMPREF0391_10164	1510258	1510497	-1	hypothetical protein
1431249	1529242	97993	EFH93797.1	HMPREF0391_10165	1510779	1512980	-1	protein
1431249	1529242	97993	EFH93798.1	HMPREF0391_10166	1512981	1513367	-1	hypothetical protein
1431249	1529242	97993	EFH93799.1	HMPREF0391_10167	1513360	1513602	-1	hypothetical protein
1431249	1529242	97993	EFH93800.1	HMPREF0391_10168	1513574	1515505	-1	putative DNA-directed DNA polymerase
1431249	1529242	97993	EFH93801.1	HMPREF0391_10169	1515480	1515680	-1	hypothetical protein
1431249	1529242	97993	EFH93802.1	HMPREF0391_10170	1515690	1516232	-1	hypothetical protein
1431249	1529242	97993	EFH93803.1	HMPREF0391_10171	1516222	1517346	-1	hypothetical protein
1431249	1529242	97993	EFH93804.1	HMPREF0391_10172	1517346	1517621	-1	hypothetical protein
1431249	1529242	97993	EFH93805.1	HMPREF0391_10173	1517584	1517748	-1	hypothetical protein
1431249	1529242	97993	EFH93806.1	HMPREF0391_10174	1517739	1517909	-1	hypothetical protein
1431249	1529242	97993	EFH93807.1	HMPREF0391_10175	1517943	1518461	-1	RNA polymerase sigma factor, sigma-70 family
1431249	1529242	97993	EFH93808.1	HMPREF0391_10176	1518796	1518960	-1	hypothetical protein
1431249	1529242	97993	EFH93809.1	HMPREF0391_10177	1519033	1519818	-1	hypothetical protein
1431249	1529242	97993	EFH93810.1	HMPREF0391_10178	1519821	1520192	-1	DNA-binding helix-turn-helix protein
1431249	1529242	97993	EFH93811.1	HMPREF0391_10179	1520443	1521657	-1	23S rRNA (uracil-5-)-methyltransferase RumA
1431249	1529242	97993	EFH93812.1	HMPREF0391_10180	1521740	1523536	-1	ABC transporter, ATP-binding protein
1431249	1529242	97993	EFH93813.1	HMPREF0391_10181	1523529	1525289	-1	ABC transporter, ATP-binding protein
1431249	1529242	97993	EFH93814.1	HMPREF0391_10182	1525264	1525740	-1	transcriptional regulator, MarR family
1431249	1529242	97993	EFH93815.1	HMPREF0391_10183	1525745	1525873	-1	hypothetical protein
1431249	1529242	97993	EFH93816.1	HMPREF0391_10184	1525897	1527198	-1	thiamine biosynthesis protein ThiC
1431249	1529242	97993	EFH93817.1	HMPREF0391_10185	1527379	1528293	-1	molybdenum cofactor biosynthesis protein C
1431249	1529242	97993	EFH93818.1	HMPREF0391_10186	1528295	1529242	-1	molybdenum cofactor biosynthesis protein A
1453710	1469475	15765	EFH93730.1	HMPREF0391_10098	1453710	1455173	-1	ABC transporter, ATP-binding protein
1453710	1469475	15765	EFH93731.1	HMPREF0391_10099	1455175	1455846	-1	cobalt transport protein
1453710	1469475	15765	EFH93732.1	HMPREF0391_10100	1455843	1456433	-1	conserved hypothetical protein TIGR02185
1453710	1469475	15765	EFH93733.1	HMPREF0391_10101	1456500	1457477	-1	transcriptional regulator, AraC family
1453710	1469475	15765	EFH93734.1	HMPREF0391_10102	1457487	1458089	-1	transcriptional regulator, TetR family
1453710	1469475	15765	EFH93735.1	HMPREF0391_10103	1458205	1458381	-1	hypothetical protein
1453710	1469475	15765	EFH93736.1	HMPREF0391_10104	1458528	1458791	1	hypothetical protein
1453710	1469475	15765	EFH93737.1	HMPREF0391_10105	1459309	1459572	-1	hypothetical protein
1453710	1469475	15765	EFH93738.1	HMPREF0391_10106	1459749	1460726	-1	caspase domain protein
1453710	1469475	15765	EFH93739.1	HMPREF0391_10107	1460746	1461123	-1	hypothetical protein
1453710	1469475	15765	EFH93740.1	HMPREF0391_10108	1461126	1461293	-1	hypothetical protein
1453710	1469475	15765	EFH93741.1	HMPREF0391_10109	1461517	1461984	-1	hypothetical protein
1453710	1469475	15765	EFH93742.1	HMPREF0391_10110	1462046	1462837	-1	CAAX amino terminal protease family protein
1453710	1469475	15765	EFH93743.1	HMPREF0391_10111	1463678	1464328	-1	hypothetical protein
1453710	1469475	15765	EFH93744.1	HMPREF0391_10112	1464635	1465384	-1	hypothetical protein
1453710	1469475	15765	EFH93745.1	HMPREF0391_10113	1465957	1467390	-1	hypothetical protein
1453710	1469475	15765	EFH93746.1	HMPREF0391_10114	1467431	1467751	-1	hypothetical protein

1453710	1469475	15765	EFH93747.1	HMPREF0391_10115	1467931	1468203	-1	hypothetical protein
1453710	1469475	15765	EFH93748.1	HMPREF0391_10116	1468489	1469475	1	integrase core domain protein
1570342	1579874	9532	EFH93858.1	HMPREF0391_10226	1570342	1571268	1	hypothetical protein
1570342	1579874	9532	EFH93859.1	HMPREF0391_10227	1571298	1571957	1	hypothetical protein
1570342	1579874	9532	EFH93860.1	HMPREF0391_10228	1572230	1572361	1	hypothetical protein
1570342	1579874	9532	EFH93861.1	HMPREF0391_10229	1572354	1573454	1	transposase
1570342	1579874	9532	EFH93862.1	HMPREF0391_10230	1573710	1573952	1	prevent-host-death family protein
1570342	1579874	9532	EFH93863.1	HMPREF0391_10231	1574033	1575247	1	transposase, Mutator family
1570342	1579874	9532	EFH93864.1	HMPREF0391_10232	1576090	1576665	-1	Fic family protein
1570342	1579874	9532	EFH93865.1	HMPREF0391_10233	1577843	1578577	-1	hydrolase, carbon-nitrogen family
1570342	1579874	9532	EFH93866.1	HMPREF0391_10234	1578577	1579080	-1	hypothetical protein
1570342	1579874	9532	EFH93867.1	HMPREF0391_10235	1579233	1579874	1	channel protein, hemolysin III family
1806496	1818737	12241	EFH94054.1	HMPREF0391_10422	1806496	1806795	-1	hypothetical protein
1806496	1818737	12241	EFH94055.1	HMPREF0391_10423	1807324	1807638	1	hypothetical protein
1806496	1818737	12241	EFH94056.1	HMPREF0391_10424	1807654	1808040	1	hypothetical protein
1806496	1818737	12241	EFH94057.1	HMPREF0391_10425	1808069	1809454	1	FtsK/SpoIIIE family protein
1806496	1818737	12241	EFH94058.1	HMPREF0391_10426	1809457	1809609	1	hypothetical protein
1806496	1818737	12241	EFH94059.1	HMPREF0391_10427	1809632	1810837	1	DNA-binding helix-turn-helix protein
1806496	1818737	12241	EFH94060.1	HMPREF0391_10428	1810880	1811101	1	hypothetical protein
1806496	1818737	12241	EFH94061.1	HMPREF0391_10429	1811218	1811715	1	antirestriction protein ArdA
1806496	1818737	12241	EFH94062.1	HMPREF0391_10430	1811690	1812196	1	hypothetical protein
1806496	1818737	12241	EFH94063.1	HMPREF0391_10431	1812180	1814627	1	hypothetical protein
1806496	1818737	12241	EFH94064.1	HMPREF0391_10432	1814630	1816807	1	hypothetical protein
1806496	1818737	12241	EFH94065.1	HMPREF0391_10433	1816804	1817805	1	NlpC/P60 family protein
1806496	1818737	12241	EFH94066.1	HMPREF0391_10434	1817802	1818737	1	hypothetical protein
1888259	1904566	16307	EFH94139.1	HMPREF0391_10507	1888259	1888393	-1	hypothetical protein
1888259	1904566	16307	EFH94140.1	HMPREF0391_10508	1889426	1889770	-1	hypothetical protein
1888259	1904566	16307	EFH94141.1	HMPREF0391_10509	1889757	1891727	-1	oligopeptide transporter, OPT family
1888259	1904566	16307	EFH94142.1	HMPREF0391_10510	1891902	1892507	1	hypothetical protein
1888259	1904566	16307	EFH94143.1	HMPREF0391_10511	1892871	1893791	-1	transposase, Mutator family
1888259	1904566	16307	EFH94144.1	HMPREF0391_10512	1893839	1894084	-1	hypothetical protein
1888259	1904566	16307	EFH94145.1	HMPREF0391_10513	1894683	1895966	-1	hypothetical protein
1888259	1904566	16307	EFH94146.1	HMPREF0391_10514	1895969	1897267	-1	hypothetical protein
1888259	1904566	16307	EFH94147.1	HMPREF0391_10515	1897280	1898029	-1	hypothetical protein
1888259	1904566	16307	EFH94148.1	HMPREF0391_10516	1898022	1898333	-1	hypothetical protein
1888259	1904566	16307	EFH94149.1	HMPREF0391_10517	1898350	1900155	-1	hypothetical protein
1888259	1904566	16307	EFH94150.1	HMPREF0391_10518	1900143	1900892	-1	hypothetical protein
1888259	1904566	16307	EFH94151.1	HMPREF0391_10519	1900918	1902234	-1	hypothetical protein
1888259	1904566	16307	EFH94152.1	HMPREF0391_10520	1902530	1904566	1	hypothetical protein
1892871	1900892	8021	EFH94143.1	HMPREF0391_10511	1892871	1893791	-1	transposase, Mutator family
1892871	1900892	8021	EFH94144.1	HMPREF0391_10512	1893839	1894084	-1	hypothetical protein
1892871	1900892	8021	EFH94145.1	HMPREF0391_10513	1894683	1895966	-1	hypothetical protein
1892871	1900892	8021	EFH94146.1	HMPREF0391_10514	1895969	1897267	-1	hypothetical protein
1892871	1900892	8021	EFH94147.1	HMPREF0391_10515	1897280	1898029	-1	hypothetical protein
1892871	1900892	8021	EFH94148.1	HMPREF0391_10516	1898022	1898333	-1	hypothetical protein
1892871	1900892	8021	EFH94149.1	HMPREF0391_10517	1898350	1900155	-1	hypothetical protein
1892871	1900892	8021	EFH94150.1	HMPREF0391_10518	1900143	1900892	-1	hypothetical protein
1904633	1910011	5378	EFH94153.1	HMPREF0391_10521	1904633	1906318	1	hypothetical protein
1904633	1910011	5378	EFH94154.1	HMPREF0391_10522	1906315	1907400	1	hypothetical protein
1904633	1910011	5378	EFH94155.1	HMPREF0391_10523	1907437	1908018	1	hypothetical protein
1904633	1910011	5378	EFH94156.1	HMPREF0391_10524	1909789	1910011	-1	hypothetical protein

Table S3L: Predicted genomic islands in the genome of *F. magna* ATCC29328

Shown are genes of genomic islands predicted by IslandViewer4

Island start	Island end	Length	Gene name	Locus	Gene start	Gene end	Strand	Product
777457	788164	10707	WP_002838404.1	FMG_RS03870	777457	778155	1	DNA-binding response regulator
777457	788164	10707	WP_012290581.1	FMG_RS03875	778196	778780	1	peptidyl-prolyl cis-trans isomerase
777457	788164	10707	WP_012290582.1	FMG_RS03880	778782	779414	1	thymidine kinase
777457	788164	10707	WP_002841719.1	FMG_RS03885	779436	779906	-1	S-ribosylhomocysteine lyase
777457	788164	10707	WP_012290583.1	FMG_RS03890	779915	781018	-1	rod shape-determining protein RodA
777457	788164	10707	WP_002841740.1	FMG_RS03895	781093	781926	1	endonuclease
777457	788164	10707	WP_002838210.1	FMG_RS03900	781907	782533	1	endonuclease III
777457	788164	10707	WP_002841720.1	FMG_RS03905	782601	783521	1	aspartate carbamoyltransferase
777457	788164	10707	WP_012290584.1	FMG_RS03910	783521	783949	1	aspartate carbamoyltransferase regulatory subunit
777457	788164	10707	WP_012290585.1	FMG_RS03915	783949	785145	1	dihydroorotase
777457	788164	10707	WP_012290586.1	FMG_RS03920	785132	785983	1	orotidine 5'-phosphate decarboxylase
777457	788164	10707	WP_012290587.1	FMG_RS03925	785976	786692	1	dihydroorotate dehydrogenase electron transfer subunit
777457	788164	10707	WP_012290588.1	FMG_RS03930	786685	787581	1	dihydroorotate dehydrogenase
777457	788164	10707	WP_012290589.1	FMG_RS03935	787592	788164	1	orotate phosphoribosyltransferase
800334	806465	6131	WP_012290598.1	FMG_RS03995	800334	802022	1	hypothetical protein
800334	806465	6131	WP_002838113.1	FMG_RS04000	802033	802830	1	bifunctional ([pyruvate, phosphate dikinase] phosphate) phosphotransferase/[pyruvate, phosphate diki
800334	806465	6131	WP_012290599.1	FMG_RS04005	802914	804347	1	serine/threonine protein kinase
800334	806465	6131	WP_012290600.1	FMG_RS04010	804349	805023	1	protein phosphatase
800334	806465	6131	WP_002838381.1	FMG_RS04015	805080	806465	1	tRNA (N6-isopentenyl adenosine(37)-C2)-methylthiotransferase MiaB
991864	1059050	67186	WP_012290711.1	FMG_RS04885	991864	993141	-1	ABC transporter permease
991864	1059050	67186	WP_012290712.1	FMG_RS04890	993339	993626	-1	cupin
991864	1059050	67186	WP_012290713.1	FMG_RS04895	993694	994266	-1	uracil-DNA glycosylase
991864	1059050	67186	WP_012290714.1	FMG_RS04900	994598	994882	-1	hypothetical protein
991864	1059050	67186		FMG_RS04905	994940	995686	-1	ABC transporter
991864	1059050	67186		FMG_RS04910	995673	996392	-1	iron ABC transporter permease
991864	1059050	67186	WP_002840481.1	FMG_RS04915	996452	997210	-1	methyltransferase
991864	1059050	67186	WP_002837989.1	FMG_RS04920	997305	997898	1	hypothetical protein
991864	1059050	67186	WP_012290720.1	FMG_RS04925	998668	998937	1	transposase
991864	1059050	67186	WP_041250650.1	FMG_RS04930	999050	999847	1	transposase
991864	1059050	67186	WP_002840495.1	FMG_RS04935	1000078	1001280	-1	ABC transporter substrate-binding protein
991864	1059050	67186	WP_012290722.1	FMG_RS04940	1001280	1001819	-1	FMN-binding domain-containing protein
991864	1059050	67186	WP_012290723.1	FMG_RS04945	1001925	1002371	-1	hypothetical protein
991864	1059050	67186	WP_012290724.1	FMG_RS04950	1002528	1003763	-1	site-specific integrase
991864	1059050	67186	WP_009345237.1	FMG_RS04955	1003841	1004050	-1	transposase
991864	1059050	67186	WP_009345230.1	FMG_RS04960	1004486	1004896	-1	sigma factor
991864	1059050	67186	WP_041250601.1	FMG_RS04965	1005216	1006565	-1	multidrug transporter MatE
991864	1059050	67186	WP_012290726.1	FMG_RS04970	1006602	1008335	-1	multidrug ABC transporter ATP-binding protein
991864	1059050	67186	WP_012290727.1	FMG_RS04975	1008332	1010074	-1	ABC transporter ATP-binding protein
991864	1059050	67186	WP_012290728.1	FMG_RS04980	1010110	1011501	-1	ABC transporter ATP-binding protein
991864	1059050	67186	WP_012290729.1	FMG_RS04985	1011494	1012195	-1	ABC transporter permease
991864	1059050	67186	WP_002836642.1	FMG_RS04990	1012220	1012801	-1	membrane protein
991864	1059050	67186	WP_012290730.1	FMG_RS04995	1012819	1013412	-1	TetR family transcriptional regulator
991864	1059050	67186	WP_012290731.1	FMG_RS05000	1013743	1014099	1	conjugal transfer protein
991864	1059050	67186	WP_041250602.1	FMG_RS05005	1014101	1015432	1	endonuclease
991864	1059050	67186	WP_012290734.1	FMG_RS05010	1015796	1018231	-1	ABC transporter permease
991864	1059050	67186	WP_012290735.1	FMG_RS05015	1018224	1018904	-1	peptide ABC transporter ATP-binding protein
991864	1059050	67186	WP_012290736.1	FMG_RS05020	1018957	1019817	-1	two-component sensor histidine kinase
991864	1059050	67186	WP_012290737.1	FMG_RS05025	1019819	1020469	-1	DNA-binding response regulator
991864	1059050	67186	WP_012290738.1	FMG_RS05030	1020651	1021754	-1	ATP-dependent DNA helicase
991864	1059050	67186	WP_012290739.1	FMG_RS05035	1021744	1023372	-1	DNA recombinase
991864	1059050	67186	WP_012290740.1	FMG_RS05040	1023395	1023601	-1	transcriptional regulator
991864	1059050	67186	WP_012290741.1	FMG_RS05045	1023780	1024436	-1	hypothetical protein
991864	1059050	67186	WP_012290742.1	FMG_RS05050	1024480	1032123	-1	DNA helicase
991864	1059050	67186	WP_002836656.1	FMG_RS05055	1032206	1034419	-1	hypothetical protein
991864	1059050	67186	WP_002836657.1	FMG_RS05060	1034455	1035417	-1	type II restriction endonuclease subunit M
991864	1059050	67186	WP_012290743.1	FMG_RS05065	1035410	1037131	-1	DNA topoisomerase III
991864	1059050	67186	WP_002836659.1	FMG_RS05070	1037195	1038061	-1	bacteriocin
991864	1059050	67186	WP_004817210.1	FMG_RS05075	1038076	1040427	-1	copper amine oxidase
991864	1059050	67186	WP_001080402.1	FMG_RS05080	1040414	1040653	-1	hypothetical protein
991864	1059050	67186	WP_012290744.1	FMG_RS05085	1040668	1043247	-1	hypothetical protein
991864	1059050	67186	WP_041250652.1	FMG_RS05090	1043255	1045498	-1	conjugal transfer protein TraE
991864	1059050	67186	WP_008902108.1	FMG_RS05095	1045569	1045979	-1	transposase
991864	1059050	67186	WP_002838520.1	FMG_RS05100	1045981	1046271	-1	hypothetical protein
991864	1059050	67186	WP_000466919.1	FMG_RS05105	1046281	1047144	-1	hypothetical protein
991864	1059050	67186	WP_000394203.1	FMG_RS05110	1047155	1047370	-1	conjugative transfer protein
991864	1059050	67186	WP_002839977.1	FMG_RS05115	1047372	1047683	-1	hypothetical protein
991864	1059050	67186	WP_001105422.1	FMG_RS05120	1047879	1049348	-1	hypothetical protein
991864	1059050	67186	WP_001258251.1	FMG_RS05125	1049366	1049902	-1	hypothetical protein
991864	1059050	67186	WP_002839985.1	FMG_RS05130	1049964	1050683	-1	conjugal transfer protein TraG
991864	1059050	67186	WP_002838479.1	FMG_RS05135	1050785	1050970	-1	transposase
991864	1059050	67186	WP_002838522.1	FMG_RS05140	1051072	1052061	-1	hypothetical protein
991864	1059050	67186	WP_012290746.1	FMG_RS05145	1052135	1052458	-1	hypothetical protein
991864	1059050	67186	WP_012290747.1	FMG_RS05150	1052641	1053354	-1	hypothetical protein
991864	1059050	67186	WP_012290748.1	FMG_RS05155	1053436	1055076	-1	nickase
991864	1059050	67186	WP_012290749.1	FMG_RS05160	1055391	1055765	-1	hypothetical protein
991864	1059050	67186	WP_002416386.1	FMG_RS05165	1055852	1056268	-1	hypothetical protein
991864	1059050	67186	WP_002416388.1	FMG_RS05170	1056555	1056788	-1	hypothetical protein
991864	1059050	67186	WP_012290750.1	FMG_RS05175	1056801	1058822	-1	ferrous iron transporter B
991864	1059050	67186	WP_012290751.1	FMG_RS05180	1058826	1059050	-1	iron transporter FeoA

991864	1059050	67186	WP_012290752.1	FMG_RS05185	1059037	1060785	-1	multidrug ABC transporter
994598	1001280	6682	WP_012290714.1	FMG_RS04900	994598	994882	-1	hypothetical protein
994598	1001280	6682		FMG_RS04905	994940	995686	-1	ABC transporter
994598	1001280	6682		FMG_RS04910	995673	996392	-1	iron ABC transporter permease
994598	1001280	6682	WP_002840481.1	FMG_RS04915	996452	997210	-1	methyltransferase
994598	1001280	6682	WP_002837989.1	FMG_RS04920	997305	997898	1	hypothetical protein
994598	1001280	6682	WP_012290720.1	FMG_RS04925	998668	998937	1	transposase
994598	1001280	6682	WP_041250650.1	FMG_RS04930	999050	999847	1	transposase
994598	1001280	6682	WP_002840495.1	FMG_RS04935	1000078	1001280	-1	ABC transporter substrate-binding protein
1018957	1024436	5479	WP_012290736.1	FMG_RS05020	1018957	1019817	-1	two-component sensor histidine kinase
1018957	1024436	5479	WP_012290737.1	FMG_RS05025	1019819	1020469	-1	DNA-binding response regulator
1018957	1024436	5479	WP_012290738.1	FMG_RS05030	1020651	1021754	-1	ATP-dependent DNA helicase
1018957	1024436	5479	WP_012290739.1	FMG_RS05035	1021744	1023372	-1	DNA recombinase
1018957	1024436	5479	WP_012290740.1	FMG_RS05040	1023395	1023601	-1	transcriptional regulator
1018957	1024436	5479	WP_012290741.1	FMG_RS05045	1023780	1024436	-1	hypothetical protein
1538370	1546110	7740	WP_012291054.1	FMG_RS07340	1538370	1539119	-1	phosphorylase
1538370	1546110	7740	WP_012291055.1	FMG_RS07345	1539249	1540850	-1	macrolide ABC transporter ATP-binding protein
1538370	1546110	7740	WP_012291056.1	FMG_RS07350	1541255	1541869	-1	hypothetical protein
1538370	1546110	7740	WP_012291057.1	FMG_RS07355	1542076	1543440	-1	ABC transporter ATP-binding protein
1538370	1546110	7740	WP_012291058.1	FMG_RS07360	1543428	1544123	-1	cobalt ABC transporter permease
1538370	1546110	7740	WP_012291059.1	FMG_RS07365	1544123	1544707	-1	membrane protein
1538370	1546110	7740	WP_041250667.1	FMG_RS07370	1544767	1546110	-1	MATE family efflux transporter
1553812	1571194	17382	WP_002836976.1	FMG_RS07405	1553812	1554507	1	DNA-binding response regulator
1553812	1571194	17382	WP_012291067.1	FMG_RS07410	1554509	1555804	1	two-component sensor histidine kinase
1553812	1571194	17382	WP_041250623.1	FMG_RS07415	1556528	1556734	-1	hypothetical protein
1553812	1571194	17382	WP_041250624.1	FMG_RS07420	1557057	1557284	-1	hypothetical protein
1553812	1571194	17382	WP_012291068.1	FMG_RS07425	1557281	1559017	-1	multidrug ABC transporter ATP-binding protein
1553812	1571194	17382	WP_012291069.1	FMG_RS07430	1559010	1560731	-1	ABC transporter ATP-binding protein
1553812	1571194	17382	WP_012291070.1	FMG_RS07435	1560769	1562121	-1	MATE family efflux transporter
1553812	1571194	17382	WP_012291071.1	FMG_RS07440	1562134	1563582	-1	ABC transporter
1553812	1571194	17382	WP_012291072.1	FMG_RS07445	1563579	1564259	-1	ABC transporter permease
1553812	1571194	17382	WP_005956598.1	FMG_RS07450	1564259	1564837	-1	hypothetical protein
1553812	1571194	17382	WP_041250625.1	FMG_RS07455	1564889	1565461	-1	TetR family transcriptional regulator
1553812	1571194	17382	WP_012291075.1	FMG_RS07460	1565754	1566110	1	conjugal transfer protein
1553812	1571194	17382	WP_004838629.1	FMG_RS07470	1566622	1568316	-1	hypothetical protein
1553812	1571194	17382	WP_041250668.1	FMG_RS07475	1568316	1569320	-1	hypothetical protein
1553812	1571194	17382		FMG_RS07480	1569768	1570610	-1	abortive phage infection protein
1553812	1571194	17382	WP_012291079.1	FMG_RS07485	1570607	1571194	-1	transcriptional regulator

Table S4A: Blast results showing protein L homologs in 12 *Finegoldia* genomes.
As query, protein L from strain 312 (locus ID: M86697) was taken. As Blast cutoff: e-value < e-10

Species	strain	gene	Prokka annotation	score	e-value
“F. nericia”	ATCC53516	12510	Peptostreptococcal albumin-binding protein precursor	1099	0.0
“F. nericia”	T160124	15430	Peptostreptococcal albumin-binding protein precursor	357	e-112
“F. nericia”	CCUG54800	17560	Peptostreptococcal albumin-binding protein precursor	334	e-104
“F. nericia”	12T273	18110	Peptostreptococcal albumin-binding protein precursor	286	7e-85
“F. nericia”	12T306	16410	Peptostreptococcal albumin-binding protein precursor	218	3e-59
“F. nericia”	CCUG54800	10280	IgA FC receptor precursor	90	1e-18
“F. nericia”	12T272	07960	IgA FC receptor precursor	89	3e-18
“F. nericia”	T160124	15840	Surface protein G precursor	71	1e-12
F. magna	08T492	16540	Peptostreptococcal albumin-binding protein precursor	69	1e-12
F. magna	07T609	13520	Extracellular matrix-binding protein ebh precursor	68	1e-11
“F. nericia”	12T273	17160	Platelet binding protein GspB precursor	65	5e-11
“F. nericia”	CCUG54800	15040	Rib/alpha-like repeat protein	65	5e-11

Table S4B: Blast results showing peptostreptococcal albumin-binding protein (PAB) homologs in 12 *Finegoldia* genomes. As query, the PAB protein of strain ALB8 (locus ID: X77864) was taken. As Blast cutoff: e-value < e-10.

**Species**	**strain**	**gene**	**Prokka annotation**	**score**	**e-value**
F. magna	08T492	16540	Peptostreptococcal albumin-binding protein precursor	149	1e-40
F. magna	07T609	15520	Peptostreptococcal albumin-binding protein precursor	140	1e-38
"F. nericia"	12T306	16410	Peptostreptococcal albumin-binding protein precursor	123	4e-30
"F. nericia"	T160124	15430	Peptostreptococcal albumin-binding protein precursor	99	8e-23
"F. nericia"	CCUG54800	17560	Peptostreptococcal albumin-binding protein precursor	99	1e-22
"F. nericia"	12T273	18110	Peptostreptococcal albumin-binding protein precursor	65	1e-11
"F. nericia"	CCUG54800	17400	Internalin-A precursor	64	6e-11

Table S4C: Blast results showing GA-module containing proteins in 12 *Finegoldia* genomes. As query, the GA module of the PAB protein of strain ALB8 (locus ID: X77864) was taken. As Blast cutoff: e-value < e-10.

Species	strain	gene	Prokka annotation	score	e-value
F. magna	07T609	15520	Peptostreptococcal albumin-binding protein precursor	78	9e-20
F. magna	08T492	16540	Peptostreptococcal albumin-binding protein precursor	78	5e-19
"F. nericia"	T160124	15430	Peptostreptococcal albumin-binding protein precursor	77	2e-18
"F. nericia"	CCUG54800	17560	Peptostreptococcal albumin-binding protein precursor	74	4e-17
"F. nericia"	12T306	16410	Peptostreptococcal albumin-binding protein precursor	74	5e-17
"F. nericia"	CCUG54800	17400	Internalin-A precursor	60	3e-12
"F. nericia"	12T273	18110	Peptostreptococcal albumin-binding protein precursor	59	6e-12
"F. nericia"	12T306	16250	Internalin-A precursor	58	1e-11
"F. nericia"	09T494	18050	N-acetylmuramoyl-L-alanine amidase LytC	58	2e-11
"F. nericia"	12T273	04210	N-acetylmuramoyl-L-alanine amidase LytC	58	2e-11
F. magna	07T609	15360	Internalin-A precursor	58	2e-11
"F. nericia"	ATCC53516	09600	N-acetylmuramoyl-L-alanine amidase LytC	58	2e-11
"F. nericia"	T160124	15270	Internalin-A precursor	57	2e-11
F. magna	08T492	16380	N-acetylmuramoyl-L-alanine amidase LytC	57	3e-11
"F. nericia"	12T272	15560	N-acetylmuramoyl-L-alanine amidase LytC	56	6e-11
F. magna	ATCC29328	15850	N-acetylmuramoyl-L-alanine amidase LytC	56	8e-11

Table S4D: Blast results showing SufA homologs in 12 *Finegoldia* genomes. As query, SufA of strain ALB8 (locus ID: DQ679960) was taken. As Blast cutoff: e-value < e-10.

Species	strain	gene	Prokka annotation	score	e-value
F. magna	08T492	17920	PII-type proteinase precursor	1971	0.0
"F. nericia"	12T273	01050	PIII-type proteinase precursor	1865	0.0
"F. nericia"	T160124	16880	PIII-type proteinase precursor	1827	0.0
"F. nericia"	ATCC53516	10860	PII-type proteinase precursor	1823	0.0
"F. nericia"	T151023	00620	PII-type proteinase precursor	1779	0.0
"F. nericia"	12T306	00480	PII-type proteinase precursor	1771	0.0
"F. nericia"	09T494	01650	PII-type proteinase precursor	1747	0.0
"F. nericia"	12T272	18030	PIII-type proteinase precursor	1725	0.0
F. magna	ATCC29328	00370	PII-type proteinase precursor	1678	0.0
F. magna	09T408	03220	PII-type proteinase precursor	157	5e-39
"F. nericia"	09T494	03870	PIII-type proteinase precursor	156	1e-38
F. magna	07T609	03110	PII-type proteinase precursor	154	9e-38
"F. nericia"	12T273	06640	PII-type proteinase precursor	153	1e-37
"F. nericia"	12T306	04830	PII-type proteinase precursor	153	1e-37
"F. nericia"	12T272	01600	PII-type proteinase precursor	153	1e-37
F. magna	08T492	02790	PII-type proteinase precursor	153	1e-37
"F. nericia"	CCUG54800	04020	PII-type proteinase precursor	153	2e-37
"F. nericia"	ATCC53516	07750	PII-type proteinase precursor	146	2e-35
"F. nericia"	T160124	03150	PII-type proteinase precursor	146	2e-35

Table S4E: Blast results showing FAF (*F. magna* adhesion factor) homologs in 12 *Finegoldia* genomes. As query, the FAF protein of strain ALB8 (locus ID: AAO46090) was taken. As Blast cutoff: e-value < e-10.

Species	strain	gene	Prokka annotation	score	e-value
F. magna	08T492	14650	Cna protein B-type domain protein	471	e-158
F. magna	ATCC29328	14170	Cna protein B-type domain protein	467	e-156
"F. nericia"	CCUG54800	15060	Cna protein B-type domain protein	451	e-150
"F. nericia"	T160124	12940	Cna protein B-type domain protein	436	e-144
F. magna	07T609	13570	Cna protein B-type domain protein	399	e-130
"F. nericia"	ATCC53516	00150	Cna protein B-type domain protein	314	9e-98
"F. nericia"	12T273	17180	Cna protein B-type domain protein	306	2e-94
"F. nericia"	T151023	14140	Cna protein B-type domain protein	239	6e-71
"F. nericia"	12T272	13990	Cna protein B-type domain protein	117	3e-29
"F. nericia"	12T272	14000	Hypothetical protein	111	4e-27
"F. nericia"	T151023	14150	Hypothetical protein	75	1e-15

Table S5: *Finegoldia* sp. strains tested in the RapID ANA II system

	strain	URE	BLTS	aARA	ONPG	aGLU	BGLU	aGAL	aFUC	NAG	PO4	LGY	GLY	PRO	PAL	ARG	SER	PYR	IND
F. magna	07T609	÷	÷	÷	÷	÷	÷	÷	÷	÷	÷	+	+	÷*	+	+	+	+	÷
F. magna	08T492	÷	÷	÷	÷	÷	÷	÷	÷	÷	÷	+	+	÷*	+	+	+	+	÷
F. magna	09T408	÷	÷	÷	÷	÷	÷	÷	÷	÷	÷	+	+	÷*	+	+	+	+	÷
"*F. nericia*"	09T494	÷	÷	÷	÷	÷	÷	÷	÷	÷	+	+	+	÷*	+	+	+	+	÷
"*F. nericia*"	12T272	÷	÷	÷	÷	÷	÷	÷	÷	÷	÷	+	+	÷*	+	+	+	+	÷
"*F. nericia*"	12T273	÷	÷	÷	÷	÷	÷	÷	÷	÷	÷	+	+	÷*	+	+	+	+	÷
"*F. nericia*"	12T306	÷	÷	÷	÷	÷	÷	÷	÷	÷	+	+	+	÷*	+	+	+	+	÷
"*F. nericia*"	CCUG54800	÷	÷	÷	÷	÷	÷	÷	÷	÷	+	+	+	÷*	+	+	+	+	÷
"*F. nericia*"	15T1023	÷	÷	÷	÷	÷	÷	÷	÷	÷	+	+	+	÷*	+	+	+	+	÷
"*F. nericia*"	16T0124	÷	÷	÷	÷	÷	÷	÷	÷	÷	+	+	+	÷*	+	+	+	+	÷

* very light pink

**Reaction**	**Substrate**
URE:	Urea
BLTS:	ρ-Nitrophenyl-β, D-disaccharide
aARA:	ρ-Nitrophenyl-α, L-arabinoside
ONPG:	σ-Nitrophenyl-β, D-galactoside
aGLU:	ρ-Nitrophenyl-α, D-glucoside
BGLU:	ρ-Nitrophenyl-β, D-glucoside
aGAL:	ρ-Nitrophenyl-α, D-galactoside
aFUC:	ρ-Nitrophenyl-α, L-fucoside
NAG:	ρ-Nitrophenyl-n-acetyl-β, D-glucosaminide
PO4:	ρ-Nitrophenylphosphate
LGY:	Leucyl-glycine-β-naphthylamide
GLY:	Glycine-β-naphthylamide
PRO:	Proline-β-naphthylamide
PAL:	Phenylalanine-β-naphthylamide
ARG:	Arginine-β-naphthylamide
SER:	Serine-β-naphthylamide
PYR:	Pyrrolidonyl-β-naphthylamide
IND:	Trytophane
